# Photoenzymatic Hydroalkylation Enables Streamlined Access to Aryl Glutarimide Precursors

**DOI:** 10.1002/anie.202600006

**Published:** 2026-03-19

**Authors:** Zhi Xu, Prasun Mukherjee, Steven Gossert, Stephen Thomas, Vasil H. Vasilev, Eric R. Welin, Yichen Tan, Shane M. McKenna, Megan A. Emmanuel, Todd K. Hyster

**Affiliations:** ^1^ Department of Chemistry Princeton University Princeton New Jersey USA; ^2^ Integrated Materials Engineering & Technology Bristol Myers Squibb New Brunswick New Jersey USA; ^3^ Discovery & Development Sciences Bristol Myers Squibb San Diego California USA; ^4^ Chemical Process Development Bristol Myers Squibb New Brunswick New Jersey USA; ^5^ Chemical Process Development Bristol Myers Squibb Company Wirral UK

**Keywords:** biocatalysis, directed evolution, glutarimide, photoenzymatic catalysis, targeted protein degradation

## Abstract

We describe a photoenzymatic hydroalkylation reaction that enables the efficient and stereocontrolled synthesis of aryl glutarimide precursors—chemically and configurationally robust entry points to bioactive agents for targeted protein degradation. Screening of flavin‐dependent “ene”‐reductases identified GluER HA*_rac_*, a *G. oxydans* variant, as an efficient and substrate‐tolerant catalyst, granting access to >30 (hetero)aryl glutarimide precursors. A directed evolution campaign then furnished a hexamutant, GluER HA*_ent_*, that delivers the products in up to 93:7 enantiomeric ratio. Mechanistic experiments revealed a pathway that departs from the hydrogen atom transfer mechanism previously established for related systems, proceeding instead via radical–polar crossover followed by enantioselective proton transfer from an active‐site tyrosine residue. Collectively, these studies establish a biocatalytic platform for advancing the synthesis and diversification of glutarimide‐containing degraders.

## Graphical abstract

We report a photoenzymatic hydroalkylation that enables streamlined, stereocontrolled access to aryl glutarimide precursors relevant to targeted protein degradation. Engineered flavin‐dependent “ene”‐reductases provide broad scope and high enantioselectivity through a distinct electron transfer–enantioselective proton transfer pathway.

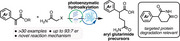

## 1 Introduction

**FIGURE 1:**
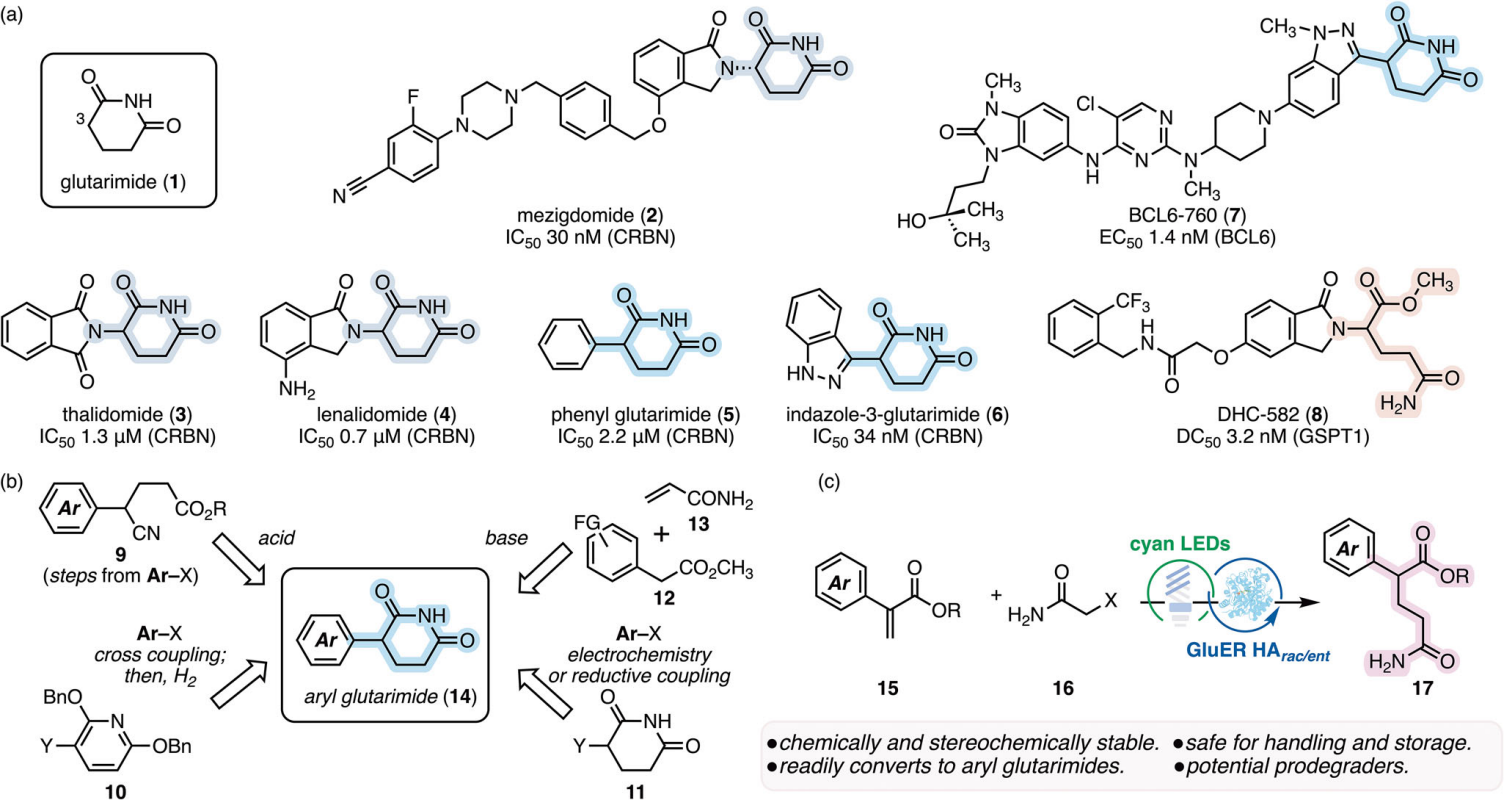
Significance and design. (a) structures of glutarimide (**1**) and representative glutarimide‐containing CELMoDs and heterobifunctional degraders **2**–**8**. IC_50_: half‐maximal inhibitory concentration. EC_50_: half‐maximal effective concentration. BCL6: B‐cell lymphoma 6 protein. GSPT1: G1 to S phase transition protein 1. DC_50_: half‐maximal degradation concentration. (b) reported synthetic strategies toward aryl glutarimide **14**. (c) photoenzymatic hydroalkylation strategy for accessing aryl glutarimide precursor **17** and its merits.

Targeted protein degradation (TPD) has emerged as a powerful therapeutic strategy [1, 2, 3, 4], as highlighted by the advancement of several agents into clinical trials (e.g., mezigdomide (**2**); Figure 1a) [5, 6]. A predominant mechanism relies on induced proximity between the protein of interest (POI) and the Cullin–RING ligase 4 (CRL4) [7], enabling POI polyubiquitination and proteasomal degradation [8]. Central to this mechanism is the recruitment of cereblon (CRBN), the substrate receptor of CRL4, by the active degrader. This interaction is typically mediated by a glutarimide pharmacophore (**1**), whose imide residue forms critical interactions with P352, S379, and H380 of CRBN, along with three additional tryptophan CH–π interactions [9, 10]. Consequently, both monovalent Cereblon E3‐Ligase Modulating Drugs (CELMoDs, e.g., **2**–**4**) [11, 12] and heterobifunctional degraders [13, 14, 15] incorporate C3–N‐linked glutarimide (in gray) as a conserved scaffold.

Despite their utility, C3–N‐linked glutarimides undergo rapid hydrolysis and racemization in aqueous media [16], motivating a shift toward more robust C3–Csp^2^‐linked aryl glutarimide derivatives (e.g., **5**–**7**, Figure 1a) [16, 17, 18, 19]. Although the C–C linkage (in blue) confers stability [16], its incorporation complicates synthesis (Figure 1b) [20]. Early strategies toward aryl glutarimides require either protic acid treatment of the nitrile **9** prepared through multiple steps [21, 22, 23], or hydrogenative deprotection of the 2,6‐bis(benzyloxy)pyridine precursor **10** following cross‐coupling with aryl halides [16, 18, 24, 25]. More recently, straightforward syntheses of **14** have been developed by the Baran [26], Reisman [27], and Jarvo [28] groups, leveraging electrochemical methods and nickel‐catalyzed reductive coupling strategies to directly incorporate the unprotected glutarimide precursor **11**. Base‐mediated coupling of **12** and **13** was also reported to assemble phenyl glutarimide analogs [29].

The pharmaceutical industry has increasingly embraced biocatalytic methodologies, recognizing their ability to mediate challenging transformations with exceptional efficiency, selectivity, and sustainability [30, 31, 32]. Over the past decade, our group has expanded this platform by developing non‐native, radical‐mediated transformations catalyzed by flavin‐dependent “ene”‐reductases (EREDs) [33]. With the goal of using this photoenzymatic platform to address pressing challenges in pharmaceutical synthesis, we sought to develop a biocatalytic process to streamline access to aryl glutarimides.

We recognized a compatibility issue at the outset of our study: despite their improved stability compared to the C3–N‐connected counterparts [16], aryl glutarimides may still not withstand the prolonged aqueous conditions typical of enzymatic catalysis. Specifically, Nilewski and co‐workers showed that the heteroaryl analogs undergo complete decomposition in DMEM buffer (pH 7.0–7.4) within 10–48 h [18], while Reisman and co‐workers [27] demonstrated that phenyl glutarimides display varying degrees of erosion in enantiomeric ratio (er) within 24 h at pH 7. Consequently, we selected for synthesis the uncyclized glutarimide precursor **17** (Figure 1c). We anticipated the protected, open‐chain residue (in pink) would resist hydrolysis [16], and the molecule would remain inert to racemization owing to its reduced acidity (calculated C3–H pKa values of 19.24 and 14.90 for **17** and **14**, respectively; Ar = Ph) [34]. In addition, the use of **17** circumvents the handling and storage hazards associated with pre‐cyclized glutarimides. Moreover, Crews and co‐workers recently reported potent prodegraders (e.g., **8**, Figure 1a) bearing C3–N‐connected, uncyclized glutamine warheads (in orange) [35]. They observed *in cellulo* cyclization of these prodegraders to form the active glutarimide pharmacophore, akin to the revelation of naturally occurring CRBN‐recruiting degrons [36]. Given the shared structures between **8** and **17**, our study will also provide a platform for investigating the bioactivities of C3–Csp^2^‐linked prodegraders.

In 2021, our group reported photoenzymatic intermolecular hydroalkylation of olefins mediated by enzyme‐templated charge‐transfer complexes [37]. Although free amides showed low reactivity and electron‐deficient alkenes displayed diminished stereoselectivity in this study, we anticipated that a related system could be developed to couple the aryl acrylate **15** with the 2‐haloacetamide **16**, providing a modular route to the aryl glutarimide precursor **17** (Figure 1c). Guided by our proposed mechanism involving a quaternary charge‐transfer complex [37], we expected that an engineered enzyme could provide high efficiency and stereoselectivity unparalleled by small‐molecule catalysts.

## 2 Results and Discussion

We first prioritized identifying an efficient, substrate‐tolerant biocatalyst, as early‐stage discovery research typically evaluates degraders using racemic mixtures [16, 19, 26]. Of note, the electron‐deficient α,β‐unsaturated ester **15** resembles the natural substrates of flavin‐dependent EREDs [38]. To disfavor the native pathway, we selected the encumbered *tert*‐butyl ester **18** for the model reaction. UV–vis studies revealed negligible dark reactivity of **18** (see Figures S14–S16 and related discussion), thereby validating this choice. In our previous report [37], coupling of the unalkylated 2‐chloroacetamide to α‐methylstyrene gave <10% conversion with an evolved *G. oxydans* variant GluER‐T36A [39]. Switching to the electrophilic, bulky substrate **18** further diminished the yield to <1% (Table 1, entry 1). Consequently, we screened an in‐house ERED library (Table S1) for the desired reactivity. We identified a point mutant, GluER‐T36A‐Y343W (hereafter GluER HA*_rac_*), as a substantially improved catalyst, affording the product **20** in 35% yield (entry 2). To better align with practical use, we designated the more valuable acrylate substrate as the limiting reagent. Systematic optimization (Table S2) identified conditions that delivered **20** in 91% with 0.75 mol% enzyme (entry 3). The reaction was successfully scaled to >1 mmol, furnishing **20** in 83% isolated yield (entry 4). The use of dialyzed cell‐free lysate was also tolerated, albeit with lower efficiency (47% yield, entry 5), presumably due to poorer light penetration. The product **20** was readily cyclized using methanesulfonic acid to give phenyl glutarimide (**5**) in 92% yield.

**TABLE 1:** Initial reaction development.

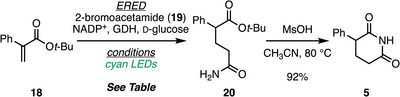		
Entry	ERED	yield
1^a^	1.00 mol% GluER‐T36A	<1%^b^
2^a^	1.00 mol% GluER HA*_rac_*	35%^b^
3^c^	0.75 mol% GluER HA*_rac_*	91%^b^
4^d^	0.75 mol% GluER HA*_rac_*	83%^e^
5^f^	1.00 mol% GluER HA*_rac_* dialyzed cell‐free lysate	47%^e^

^a^ Reaction conditions: **19** (20.0 µmol), **18** (2.00 equiv), ERED (1.00 mol%), NADP^+^ (5.00 mol%), GDH (1.50 mg), d‐glucose (6.00 equiv), KP_i_ buffer (pH 7.0, 100 mM, 900 µL), dimethyl sulfoxide (DMSO, 100 µL), cyan LEDs, 24 h.

^b^ Yield determined by LC‐MS using tribromobenzene as an internal standard and quantified against a standard calibration curve.

^c^ Reaction conditions: **18** (26.7 µmol), **19** (2.00 equiv), GluER HA*_rac_* (0.75 mol%), NADP^+^ (2.00 mol%), GDH (2.00 mg), d‐glucose (2.00 equiv), Tricine buffer (pH 9.0, 100 mM, 900 µL), DMSO (100 µL), cyan LEDs, 36 h, *vide infra*.

^d^ **18** (1.20 mmol) with GluER HA*_rac_* (0.75 mol%).

^e^ yield determined by isolation.

^f^ **18** (1.00 mmol) with GluER HA*_rac_* dialyzed cell‐free lysate (1.00 mol%).

**FIGURE 2:**
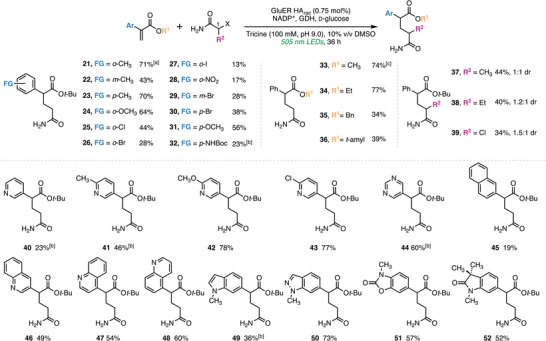
Scope of the photoenzymatic hydroalkylation reaction with GluER HA*_rac_*. [a] yields represent the average of three independent reactions, determined by ^1^H NMR spectroscopy using trimethoxybenzene as an internal standard. [b] 1.00 mol% GluER HA*_rac_* was used. [c] GluER HA*_ent_* was used instead of GluER HA*_rac_*.

With the optimal conditions in hand, we evaluated the scope and limitations of the reaction using (hetero)aryl acrylate substrates (Figure 2). Methyl substituents were well tolerated at all positions of the phenyl ring, delivering the corresponding products in uniformly good yields (**21**, 71%; **22**, 43%; **23**, 70%). For electron‐donating groups, *o*‐methoxy was compatible, with **24** formed in 64% yield. *Ortho*‐halogenated acrylates were accepted as well, although yields decreased in the order of *o*‐Cl > *o*‐Br > *o*‐I (**25**, 44%; **26**, 28%; **27**, 13%), consistent with increasingly disfavored substrate binding arising from steric congestion. Electron‐deficient analogs proved more challenging, owing to their lability under the strongly reducing conditions. The *o*‐NO_2_ analog (**28**) afforded 17% yield, with the remainder of the mass balance attributed to unreacted starting material and an over‐reduced byproduct (see Table S3). Other electron‐poor arenes, such as trifluoromethyl‐substituted phenyl acrylate (not shown), afforded <5% yield (Table S3). On the acetamide side, substrates bearing methyl, ethyl, or chloro substituents at the C1 positions were tolerated, delivering **37**–**39** as 1:1–1.5:1 diastereomeric mixtures in 34%–44% yields. Because substitution patterns are known to strongly influence the stereochemical stability of aryl glutarimides [27], **37**–**39** may enable access to more robust glutarimide analogs. Greater steric encumbrance—either from phenyl substitutions on the aromatic ring of the acrylate or the α‐position of the haloacetamide, or from a methyl substitution on the β‐position of the unsaturated ester (not shown)—entirely abolished reactivity (Table S3). Additionally, the method was found incompatible with the synthesis of C3–N‐ or C3–Csp^3^‐linked glutarimide precursors (Table S3).

To facilitate downstream pharmaceutical applications, we first demonstrated that aryl acrylate substrates bearing a bromo handle at all positions on the aryl ring were compatible, affording the products **26**, **29**, and **30** in 28%–38% yield. These intermediates are challenging to access via reductive coupling methodologies [26, 27, 28] and may serve as modular fragments for the synthesis of heterobifunctional degraders via cross‐coupling with POI binders. The *p*‐methoxy and *p*‐*tert‐*butoxycarbonyl amino analogs **31** and **32** were obtained in 56% and 23% yields. Under acidic conditions [29], these precursors can convert to the corresponding phenyl glutarimides, which are known CRBN binders (IC_50_ = 3.2 µM and 0.12 µM for *p*‐OCH_3_‐ and *p*‐NH_2_‐substituted phenyl glutarimide, respectively) [16]. In applying this method to study aryl‐substituted prodegraders [35], we demonstrated that both methyl and ethyl acrylates were viable substrates, furnishing the desired products in 74% (**33**) and 77% (**34**) yield. Based on the findings of Crews and co‐workers [35], we expect these esters to exhibit more favorable in vivo cyclization kinetics than the *tert*‐butyl ester **20**. Additionally, we found that benzyl (**35**, 34%) and *tert*‐amyl (**36**, 39%) acrylates were also tolerated. Finally, we thoroughly evaluated the compatibility of pharmaceutically relevant heterocyclic substrates [18, 19, 22, 23, 24, 25]. A broad range of systems were found to be competent, including pyridine (**40**–**43**, 23%–78%), pyrimidine (**44**, 60%), naphthalene (**45**, 19%), quinoline (**46**–**48**, 49%–60%), indole (**49**, 36%), indazole (**50**, 73%), oxazolidinone (**51**, 57%), and oxindole (**52**, 52%). These results highlight the broad functional‐group tolerance of the method.

We next directed our efforts toward developing an enzyme capable of catalyzing the reaction with high enantioselectivity, thereby enabling rigorous biological studies of each aryl glutarimide enantiomer [40] and facilitating lead‐compound characterization in process chemistry [41]. Guided by the crystal structure of GluER‐T36A (PDB: 6MYW) [39], we initiated an engineering campaign targeting residues lining the protein active site. To our surprise, a round of site‐saturation mutagenesis with GluER HA*_rac_* as the template failed to yield any mutant with improved enantioselectivity (Table S4). We therefore reevaluated other GluER mutants (Table S5) and found that 1.00 mol% GluER‐T36A‐Y177F furnished **20** in 14% yield with 65:35 er [42]. Despite its lower activity, we selected this variant as the new parent for directed evolution. To accelerate progress, we iteratively optimized reaction conditions in parallel with the mutagenesis efforts.

**FIGURE 3:**
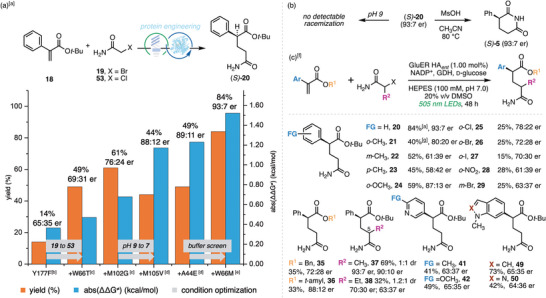
Development and scope of the enantioselective photoenzymatic hydroalkylation. (a) evolutionary campaign and condition optimization to engineer an enantioselective enzyme. (b) stereochemical stability of the enantio‐enriched **20**. (c) scope of the enantioselective hydroalkylation reaction with GluER HA*_ent_*. [a] yields represent the average of three independent reactions, determined by LC‐MS using tribromobenzene as an internal standard and quantified against a standard calibration curve; enantiomeric ratios determined by chiral HPLC analysis, *vide infra*. [b] reaction conditions: **19** (20.0 µmol), **18** (2.00 equiv), ERED (1.00 mol%), NADP^+^ (1.00 mol%), GDH (1.50 mg), d‐glucose (6.00 equiv), Tris buffer (pH 9.0, 100 mM, 900 µL), DMSO (100 µL), cyan LEDs. [c] reaction conditions: **53** (20.0 µmol), **18** (4.00 equiv), ERED (1.00 mol%), NADP^+^ (1.00 mol%), GDH (1.50 mg), d‐glucose (6.00 equiv), Tris buffer (pH 9.0, 100 mM, 900 µL), DMSO (100 µL), cyan LEDs. [d] reaction conditions: **18** (20.0 µmol), **53** (4.00 equiv), ERED (1.00 mol%), NADP^+^ (1.00 mol%), GDH (1.50 mg), d‐glucose (6.00 equiv), Tris buffer (pH 7.5, 100 mM, 900 µL), DMSO (100 µL), cyan LEDs. [e] Reaction conditions: **18** (20.0 µmol), **53** (4.00 equiv), ERED (1.00 mol%), NADP^+^ (1.00 mol%), GDH (1.50 mg), d‐glucose (6.00 equiv), HEPES buffer (pH 7.0, 100 mM, 800 µL), DMSO (200 µL), cyan LEDs. [f] absolute configurations depicted for products herein were assigned by analogy to **20**. (g) yields represent the average of three independent reactions, determined by ^1^H NMR spectroscopy using trimethoxybenzene as an internal standard; enantiomeric ratios determined by chiral HPLC analysis, *vide infra*.

The first round of evolution identified residue W66T as a beneficial mutation, and contemporaneous screening (Table S6) revealed that replacing 2‐bromoacetamide (**19**) with its chloride congener **53** further improved performance, together delivering **20** in 49% yield and 69:31 er (Figure 3a). In the second round, introduction of a glycine residue at position 102 (M102G) further enhanced catalytic performance (61% yield, 76:24 er). A subsequent round targeting eight active‐site residues delivered minimal improvements in stereoselectivity, prompting reevaluation of the reaction conditions. After some optimization, we found that lowering the pH to 7.5 significantly increased enantioselectivity (86:14 er with GluER‐T36A‐Y177F‐W66T‐M102G, Tables S10 and S11), albeit with reduced yield (40%). Two additional rounds of engineering combined with computational simulations (see Supporting Information section 9.3) identified mutations M105V and A44E, which marginally improved overall performance (49% yield, 89:11 er). Exhaustive screening of buffer and co‐solvent combinations (Table S14) found pH 7.0 HEPES (100 mM)–20% v/v dimethyl sulfoxide as optimal, increasing the yield to 75% with a slight decrease in er (85:15). A final round of engineering led to the replacement of the threonine mutation at position 66 with a methionine (W66T to W66M), affording product **20** in 84% yield and 93:7 er. Gratifyingly, **20** displayed high stereochemical integrity, showing no detectable loss of configuration at pH 9 or during the acidic cyclization to the corresponding glutarimide **5** (Figure 3b). Comparison of the optical rotation of enantio‐enriched **5** with the reported value [27] assigned its (*S*)‐configuration.

We subsequently evaluated the scope and limitations of the enantioselective hydroalkylation (Figure 3c) using the evolved GluER‐T36A‐Y177F‐M102G‐M105V‐A44E‐W66M variant (hereafter GluER HA*_ent_*). Reactions with methyl‐substituted phenyl acrylates gave products **21**–**23** in 40%–52% yield while revealing a positional effect, with enantioselectivity decreasing in the order *o*‐CH_3_> *m*‐CH_3_> *p*‐CH_3_ (80:20 er for **21**, 61:39 er for **22**, 58:42 er for **23**). This trend was attributed to the increased perturbation of the preferred substrate‐binding geometry in the active site, and was observed with other substituents (Table S17a). Despite compromised generality, a range of *ortho*‐substituents, including methoxy (**24**), chloro (**25**), bromo (**26**), iodo (**27**), and nitro (**28**) groups, as well as a *meta*‐substituted bromide (**29**), were tolerated, affording the corresponding enantioenriched products in 15%–59% yield and 61:39–87:13 er. On the ester side (R^1^), although methyl and ethyl acrylates exhibited low enantioselectivity (Table S17a), the benzyl‐ and *tert*‐amyl‐substituted products **35** and **36** were obtained in 35% and 33% yield, respectively, with 72:28 and 88:12 er. Use of α‐alkylated haloacetamide as the coupling partner furnished the 5‐alkyl‐substituted aryl glutarimide precursors **37** (69% yield, 1:1 dr) and **38** (32% yield, 1.2:1 dr) in good to excellent enantioselectivity (97:3 er and 90:10 er for **37**, 70:30 and 63:37 er for **38**), expanding the potential utility of this novel scaffold. For heterocyclic acrylates, 41–73% yield and 63:37–65:35 er were observed for pyridine‐ (**41** and **42**), indole‐ (**49**), and indazole‐derived (**50**) analogs. While the remaining substrates in Figure 2 displayed modest enantioselectivity or diminished yields under the optimized conditions (Tables S17a, b), these results nonetheless underscore the developability of this platform for stereocontrolled access to glutarimide precursors in response to prospective industrial needs.

**FIGURE 4:**
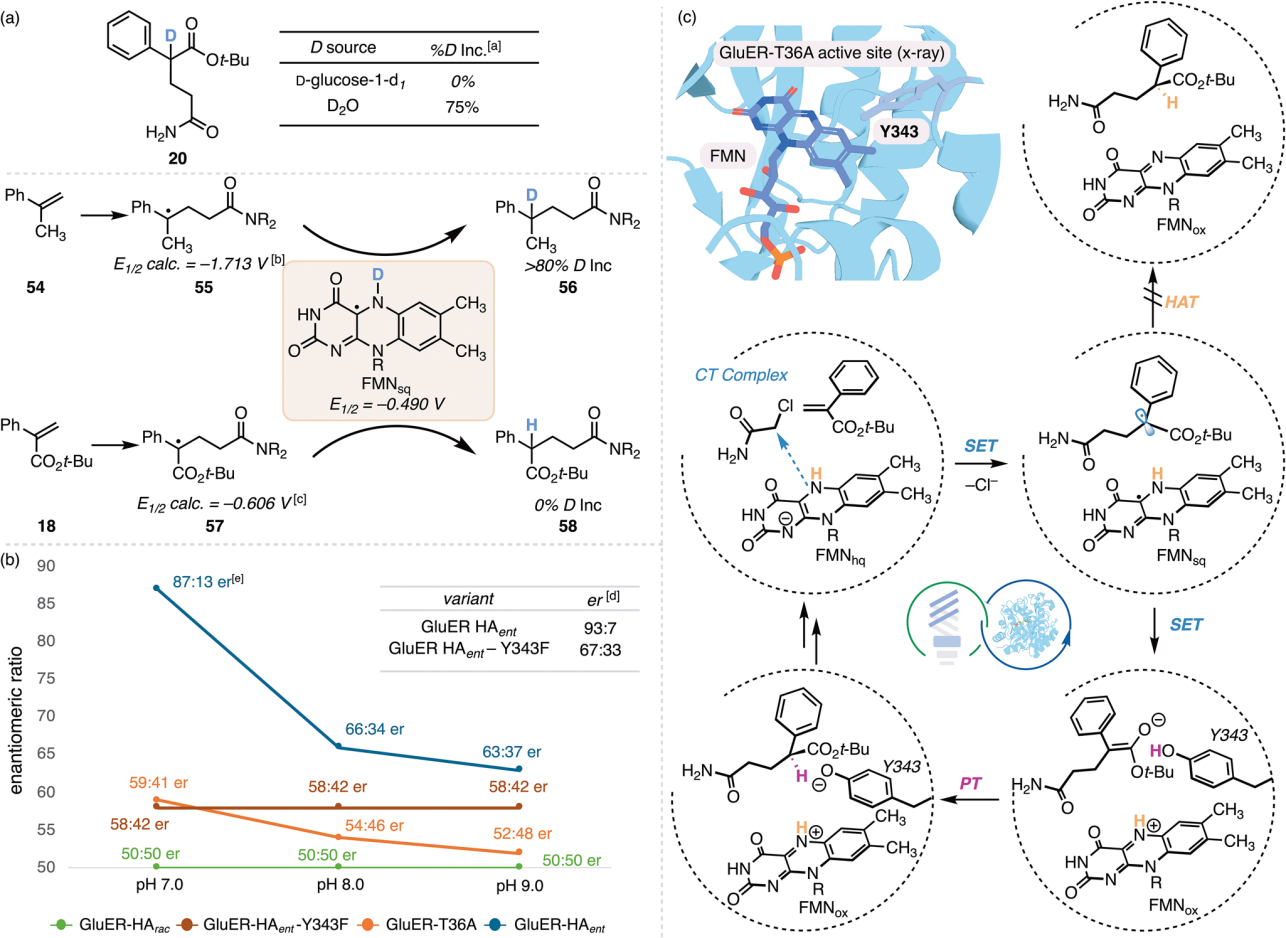
Mechanistic studies. (a) isotope incorporation studies. (b) site‐directed mutagenesis and pH dependency studies. (c) proposed mechanism. FMN_hq_: flavin hydroquinone. FMN_ox_: flavin quinone. [a] reaction conditions: **18** (20.0 µmol), **53** (4.00 equiv), GluER HA*_ent_* (1.00 mol%), NADP^+^ (1.00 mol%), GDH (1.50 mg), d‐glucose (6.00 equiv), Tris buffer (pH 9.0, 100 mM, 900 µL), DMSO (100 µL), cyan LEDs; with the corresponding d‐labelled reactants. [b] calculated for *R* = CH_3_. [c] calculated for *R* = H. [d] enantiomeric ratio determined for reactions under the optimal reaction conditions for GluER HA*_ent_*, *vide infra*. [e] Tris buffer (100 mM, pH 7.0–9.0) was used here for its suitable buffering range.

Having developed an enantioselective catalyst, we sought to investigate the reaction mechanism. Control experiments (Table S19) confirmed that the enzyme, light irradiation, and the cofactor turnover system (NADP^+^, GDH, and d‐glucose) are all essential for this reaction. Consumption of **53** was detected only when both **18** and the enzyme were present, consistent with the formation of an enzyme‐templated quaternary complex [37]. Based on our previous studies [37], we initially proposed that the stereocenter in **20** was established via selective hydrogen atom transfer (HAT) from flavin semiquinone (FMN_sq_) to the prochiral radical formed upon addition (Figure 4c, top). To probe this, we conducted an isotope incorporation experiment using d‐glucose‐1‐d*_1_* [39], which would lead to deuteration of the flavin N5‐position (Figure 4a). Surprisingly, no deuterium was incorporated into the product **20**. In contrast, when the reaction was run with unlabeled d‐glucose in deuterated buffer, 75% deuterium incorporation was observed [34]. Because solvent exchange of the flavin N5–H(D) is negligible at pH 9 [43], these results rule out the enantioselective HAT pathway.

To pinpoint the origin of the distinct mechanistic pathway, we compared deuterium incorporation for reactions with α‐methylstyrene (**54**) and the phenyl acrylate **18** (Figure 4a, bottom) [34]. Of note, *N*,*N*‐dimethyl chloroacetamide was used to achieve comparable reactivities [37]. With in situ‐generated N5–D flavin, the α‐methylstyrene‐derived product **56** incorporated >80% deuterium. In contrast, the acrylate‐derived product **58** showed none under identical conditions, indicating that the acrylate substrate **18** is primarily responsible for the mechanistic divergence. A key distinction between the two substrates lies in their electronic properties. Redox potential calculations [34] revealed that while the styrene‐derived radical **55** is difficult to reduce (calculated *E*_1/2_ = –1.713 V), the acrylate‐derived radical **57** (calculated *E*_1/2_ = –0.606 V) falls within the range for single‐electron transfer (SET) from FMN_sq_ (*E*_1/2_ = –0.490 V) [44]. We therefore propose that the radical intermediate undergoes a FMN_sq_‐mediated radical–polar crossover rather than stereoselective HAT, generating an enolate intermediate that was enantioselectively protonated (Figure 4c, bottom). The intermediacy of a charged species was supported by a strong linear correlation between the enantioselectivities of *o*‐substituted phenyl acrylates and the electronic nature of the substituents (see Table S24, Figure S13, and related discussion).

Considering the results from the isotope incorporation studies, we propose that the enolate intermediate is stereoselectively protonated by an active‐site residue bearing a solvent‐exchangeable proton. This model is consistent with the pH dependency of enantioselectivity observed during reaction optimization (Table S10): at higher pH, the key residue is predominantly deprotonated, thereby suppressing the enzyme‐mediated proton‐transfer (PT) pathway and leading to nonselective protonation by the solvent.

Analysis of the protein active site drew our attention to a tyrosine residue (side‐chain pKa 10.07) at position 343 (Figure 4c). Notably, GluER HA*_rac_*, which contains a Y343W mutation, catalyzed hydroalkylation of >30 distinct acrylates with negligible enantioselectivity. Moreover, every variant bearing a mutated Y343 gave diminished enantioselectivity in our engineering campaign (Table S8), in addition to an entirely unfruitful round using GluER HA*_rac_* as the parent (Table S4). These observations implied Y343 as a key residue in the enantio‐determining step. To further probe this hypothesis, we generated the Y343F variant of GluER HA*_ent_* via site‐directed mutagenesis (Figure 4b). As expected, this mutation resulted in a substantial drop in enantioselectivity (from 93:7 to 67:33 er) under optimized conditions, despite introducing minimal structural perturbation to the active site.

To validate the role of Y343 as a proton donor, we evaluated the dependence of enantioselectivity on buffer pH across a series of GluER variants (Figure 4b). Enzymes retaining the tyrosine at position 343 showed a pronounced inverse correlation between enantiomeric ratio and increasing pH. In contrast, GluER HA*_rac_* and GluER‐HA*_ent_*‐Y343F, in which this residue was mutated, exhibited no pH‐dependent change in selectivity. These results directly link reaction enantioselectivity to the protonation state of Y343, providing strong evidence that this residue serves as the proton source in the enantiodetermining step. Accordingly, we propose a revised mechanism involving sequential electron transfer and enantioselective proton transfer, shown in Figure 4c.

## 3 Conclusion

In conclusion, we developed a photoenzymatic hydroalkylation method that provides streamlined access to pharmaceutically relevant aryl glutarimide precursors. A GluER variant was identified to deliver a broad scope of (hetero)aryl glutarimide analogs with high efficiency. Through protein engineering, we further obtained an enzyme that enables stereoselective access to the products. The enantioselectivity was found to derive from a novel SET–PT mechanism, distinct from the HAT pathway established for related systems. Overall, this work demonstrates that non‐native biocatalytic mechanisms can provide intriguing solutions to outstanding challenges at the forefront of pharmaceutical research.

## Conflicts of Interest

The authors declare no conflicts of interest.

## Supporting information




**Supporting File**: The authors have cited additional references within the Supporting Information [45, 46, 47, 48, 49, 50, 51, 52, 53, 54, 55, 56, 57, 58, 59, 60, 61, 62, 63, 64, 65, 66, 67, 68, 69, 70, 71, 72.

## Data Availability

The data that support the findings of this study are available from the corresponding author upon reasonable request.
