# Supplementary material for: Photoenzymatic Hydroalkylation Enables Streamlined Access to Aryl Glutarimide Precursors
Source: Angew Chem Int Ed Engl. 2026 Mar 19;65(18):e00006. doi: 10.1002/anie.202600006 (PMC13021314; doi:10.1002/anie.202600006)
Supplement: Supplementary file 1 — Supporting File: The authors have cited additional references within the Supporting Information [45, 46, 47, 48, 49, 50, 51, 52, 53, 54, 55, 56, 57, 58, 59, 60, 61, 62, 63, 64, 65, 66, 67, 68, 69, 70, 71, 72. [file ANIE-65-e00006-s001.pdf]

Photoenzymatic Hydroalkylation Enables Streamlined Access to Aryl Glutarimide Precursors.

Zhi Xu, [‡,1] Prasun Mukherjee, [‡,1] Steven Gossert,[2,a] Stephen Thomas,[2] Vasil H. Vasilev,[3] Eric R. Welin,[3] Yichen Tan,[4,b] Shane M. McKenna,[5] Megan A. Emmanuel,[4] and Todd K. Hyster[*,1]

[1] Department of Chemistry, Princeton University, Princeton, New Jersey 08544, United States.

[2] Integrated Materials Engineering & Technology, Bristol Myers Squibb, New Brunswick, New Jersey 08901, United States.

[3] Discovery & Development Sciences, Bristol Myers Squibb, San Diego, California 92191, United States.

[4] Chemical Process Development, Bristol Myers Squibb, New Brunswick, New Jersey 08901, United States.

[5] Chemical Process Development, Bristol Myers Squibb Company, Reeds Lane, Moreton, Wirral CH46 1QW, United Kingdom

[a] Present address: SilcsBio, 1300 Bayard St, Baltimore, Maryland 21230, United States.

[b] Present address: Eli Lilly and Company, 1223 W Morris St, Indianapolis, Indiana 46221, United States.

[‡] Equal contribution.

Angewandte Chemie International Edition.

Supporting Information

Index:

1. General information S1
   1.1 General experimental procedures S1
   1.2 Materials S1
   1.3 Instrumentation S1
   1.4 LED Lamps S2
   1.5 Chromatography S2
   1.6 Cloning S2
   1.7 Protein Expression and Purification S2
   1.8 Preparation of dialyzed cell-free lysate S3
2. DNA and protein sequences S4
3. Development of the photoenzymatic hydroalkylation reaction. S7
   3.1 General procedures for photoenzymatic reactions S7
   3.2 Site-saturation mutagenesis and construction of site-saturation libraries. S7
   3.3 Screening procedure in 96-well plates S7

3.4 Site-directed mutagenesis S8
3.5 Supplementary Tables for the development of the photoenzymatic hydroalkylation reaction with GluER HA_{rac} S9
3.6 Development of the photoenzymatic hydroalkylation reaction with GluER HA_{ent}. S12
4. Synthetic Procedures S22
4.1 Synthetic procedures for the substrates **18**, **21-s**–**52-s**. S22
4.2 Synthetic procedure for the product standards **20** and **S11** S32
4.3 Synthetic procedure for the phenyl glutarimide (**5**) from the amide **20** S35
5. Photoenzymatic reaction procedures S36
5.1 Procedures for the synthesis of **20**–**52** via analytical-scale photoenzymatic hydroalkylation reactions S36
5.2 Procedures for the synthesis of **20** via preparative-scale photoenzymatic hydroalkylation reactions S57
6. Determination of the absolute configuration of the amide **20** S61
7. Racemization studies of the amide **20** S63
8. Mechanistic studies S64
8.1 Control experiments S64
8.2 Site-directed mutagenesis studies S65
8.3 Deuterium incorporation studies S66
8.4 Studies of the correlation between buffer pH and enantioselectivity S77
8.5 Studies of the correlation between electronic properties of the substrates and enantioselectivity S79
8.6 UV–Vis studies S80
9. Computational Studies S84
9.1 Calculation of pKa values S84
9.2 Calculation of redox potential values S85
9.3 Biocatalysis modeling studies S86
10. Cyclic Voltammetry Studies S89
11. Chiral HPLC traces S91
12. Catalog of nuclear magnetic resonance spectra S125
13. Bibliography S245

1. General Information.

1.1 General experimental procedures. All reactions were performed in single-neck, flame-dried, round-bottomed flasks fitted with rubber septa under a nitrogen atmosphere unless otherwise noted. Air- and moisture-sensitive liquids were transferred via syringe. Organic solutions were concentrated by rotary evaporation at 30–40 °C. Flash-column chromatography was performed as described by Still et al.,[1] employing silica gel ('SiliaFlash® P60', 60 Å, 40–63 μm particle size) purchased from SiliCycle. Analytical thin-layered chromatography (TLC) and preparative TLC were performed using glass plates pre-coated with silica gel (250 μm, 60 Å pore size) impregnated with a fluorescent indicator (254 nm). TLC plates were visualized by exposure to ultraviolet light (UV) and/or submersion in aqueous potassium permanganate ($KMnO_4$) or phosphomolybdic acid (PMA) stains, followed by brief heating with a heat gun (15–30 s).

1.2 Materials. Commercial solvents and reagents were used as received, with the following exceptions. Tetrahydrofuran was dried over molecular sieves. All organic solvents and aqueous buffer solutions employed in the enzymatic reactions were sparged with nitrogen for 18 h and stored in a nitrogen-filled MBraun Unilab^{pro} SP box (working O_2 level <5 ppm). 2-Chloroacetamide (Sigma-Aldrich) was recrystallized from water before use. The nicotinamide adenine dinucleotide phosphate(NADP)-dependent glucose dehydrogenase GDH-105 (GDH hereafter) was purchased as a cell-free lysate from Codexis and used as received. NADP-004660 ($NADP^+$ hereafter) was purchased from Codexis and used as received. Riboflavin-5′-phosphate sodium salt dihydrate (FMN-Na•$2H_2O$, FMN hereafter) was purchased from CHEM-IMPEX INT'L INC. and used as received. Zinc powder was activated as follows: the powder was stirred in 1 M aqueous hydrochloric acid for 2 h at 23 °C. The suspension was filtered, and the solid residue was washed sequentially with water, ethanol, acetone, and diethyl ether. The washed residue was collected and dried under reduced pressure for 18 h. Deuterated buffer solutions were prepared as follows: the buffer was flash-frozen in liquid nitrogen and lyophilized at room temperature for 24 h. The residue was resuspended in an equal volume of D_2O, then flash-frozen in liquid nitrogen and lyophilized at room temperature for 24 h. This procedure was repeated once, and the residue was transferred into a nitrogen-filled MBraun Unilab^{pro} SP box (working O_2 level <5 ppm), and resuspended in an equal volume of degassed D_2O prior to use.

1.3 Instrumentation. Proton nuclear magnetic resonance spectra (^{1}H NMR) were recorded at 400 and 500 megahertz (MHz) at 23 °C, unless otherwise noted. Chemical shifts are expressed in parts per million (ppm, δ scale) downfield from tetramethylsilane and are referenced to residual protium in the NMR solvent ($CHCl_3$, δ 7.26). Data are represented as follows: chemical shift, multiplicity (s = singlet, d = doublet, t = triplet, q = quartet, m = multiplet and/or multiple resonances), coupling constant in Hertz (Hz), assignment, and integration. Proton-decoupled carbon nuclear magnetic resonance spectra (^{13}C{^{1}H} NMR, hereafter ^{13}C NMR) were recorded at 125 MHz at 23 °C, unless otherwise noted. Chemical shifts are expressed in parts per million (ppm, δ scale) downfield from tetramethylsilane and are referenced to the carbon resonances of the solvent ($CDCl_3$, δ 77.0). High-resolution mass spectra (HRMS) were obtained on an Agilent 6230 High-Resolution ESI-TOF Mass Spectrometer. IR spectra were recorded on an Agilent Cary 630 FTIR spectrometer, and peaks were reported as the frequency of absorption (cm^{-1}). Optical rotations were recorded on a Jasco P-2000 polarimeter with a Jasco 1103-1105A cylindrical glass cell. Ultraviolet–visible spectra were recorded on a Horiba Scientific Duetta fluorescence and absorbance spectrometer.

1.4 LED lamps. The analytical-scale photoenzymatic hydroalkylation reactions employed a Lumidox® II LED array, which consists of a Lumidox® II controller (SKU: LUM2CON) and a Lumidox® II 96-well 505 nm cyan LED array with lens mat and active cooling base (SKU: LUM296LA505). The preparative-scale photoenzymatic hydroalkylation reactions employed cyan LED lamps constructed from Chanzon High Power 50W Cyan LED chips (497 nm/1500 mA/DC30-34 V/50 W) (Amazon 1DGL-JC-50W-490), powered by Mean Well HLG-320H-C1750A power supplies (320 W/183 V/1750 mA) by wiring two LED chips in series. Each LED chip was secured to a Nagulagu cooling aluminum LED heatsink equipped with a 12V fan (Amazon B01K1Z6VP6). The chemical synthesis of the product standard **20** employed a Lumidox® II LED array, which consists of a Lumidox® II controller (SKU: LUM2CON) and a Lumidox® II 96-well 470 nm cyan LED array with lens mat and active cooling base (SKU: LUM296LA470).

1.5 Chromatography. Analytical high-performance liquid chromatography (HPLC) and Electron Spray Ionization (ESI) mass spectrometry were carried out using an Agilent 1260 Infinity Liquid Chromatography-Mass Spectrometry (LC-MS) System. Yields and conversions were determined using a Waters XSelect™ Premier HS ST3 column (4.6 × 100 mm, 2.5 μm) with an internal standard 1,3,5-tribromobenzene (TBB) at 210 nm. Reverse-phase chiral HPLC analyses were conducted using a Shimadzu LC-2050C liquid chromatograph system with water and acetonitrile as mobile phases. CHIRALCEL® OX-3R column (4.6 × 250 mm, 3 μm) was used to separate enantiomers at 25–30 °C. Normal-phase chiral HPLC analyses were conducted using an Agilent 1260 Infinity chiral HPLC system with isopropanol and hexane as mobile phases. CHIRALPAK® IE, IG, and IH columns (4.6 × 250 mm, 5 μm) were used to separate enantiomers at 25–30 °C.

1.6 Cloning. pET22b(+) was used as a cloning and expression vector for all enzymes described herein. Genes for 'ene' reductase enzymes were purchased as gBlocks from IDT and cloned using the Gibson cloning method.[2] All genes were cloned between the NdeI and XhoI restriction sites and contained an N-terminal 6 × His tag. Cloning for each construct was carried out using BL21 *E. coli*.

1.7 Protein Expression and Purification. *Gluconobacter oxydans* enoate reductase (GluER, GenBank accession number: AAW60280) used in purified protein experiments was expressed in BL21 *E. coli* transformed with a plasmid encoding GluER variants. Mutants were expressed identically to the wild-type enzyme. Transformed glycerol stocks were used to initiate a 5 mL overnight culture in Luria–Bertani (LB) media with ampicillin (100 μg/mL) at 37 °C and 250 rpm. 500 mL of Terrific Broth™ (TB) media in a 2 L baffled shake flask containing ampicillin (100 μg/mL) and auto-inducing mix (sterile filtered mixture of 1% glucose, 4% lactose and 15% glycerol, 40 mL/L media) were inoculated with 1 mL of the overnight culture, then grown at 30 °C and 250 rpm for 24 h. Following expression, the cells were harvested by centrifugation (4000 × *g*, 15 min, 4 °C) and resuspended in GluER storage buffer (100 mM triethanolamine (TEOA), pH 7.0). The cells were then pelleted by centrifugation (4000 × *g*, 20 min, 4 °C). The supernatant was discarded, and the cell pellet was frozen at –20 °C for storage. For purification, frozen cells were thawed, resuspended in buffer A (20 mM TEOA, pH 7.0, 300 mM NaCl, 25 mM imidazole), and supplemented with lysozyme (1 mg/mL), Deoxyribonuclease (DNase) I (0.1 mg/mL), FMN (1 mg/mL), and phenylmethylsulfonyl fluoride (PMSF, 1 mM, added as a 35 mg/mL solution in absolute ethanol). The mixture was allowed to shake for 30 min at 37 °C. The cells were further

disrupted by sonication (2 × 4 min, output control 5, 35% duty cycle; Sonicator QSonica Q500 Ultra Sonicator). Lysates were centrifuged at 65,000 × *g* for 45 min at 4 °C to pellet insoluble materials. Protein dissolved in the supernatant was purified using a nickel-NTA resin column. Enzymes were eluted with 100% buffer B (20 mM TEOA, pH 7.0, 300 mM NaCl, 250 mM imidazole) over 5 column volumes. Yellow fractions containing enzyme were pooled, concentrated, and subjected to three exchanges with imidazole-free storage buffer (100 mM TEOA, pH 7.0) to remove excess salt and imidazole. Concentrated proteins were aliquoted, flash frozen in liquid N_2, and stored at –80 °C for later use. Protein concentration was determined using the reported[3] extinction coefficient (11.4 $mM^{-1}cm^{-1}$ at 464 nm). All proteins other than GluER were used as aliquots pre-expressed and purified according to the procedures detailed in previously published work from the Hyster lab.[3-4]

1.8 Preparation of dialyzed cell-free lysate. The cells expressed according to the above-mentioned procedure were pelleted and resuspended in GluER storage buffer (100 mM TEOA, pH 7.0) before being frozen at –20 °C for 14 h. The frozen suspension was then thawed and supplemented with lysozyme (1 mg/mL), DNase I (0.1 mg/mL), FMN (1 mg/mL), and PMSF (1 mM, added as a 35 mg/mL solution in absolute ethanol). The mixture was allowed to shake for 30 min at 37 °C. The cells were further disrupted by sonication (2 × 4 min, output control 5, 35% duty cycle; Sonicator QSonica Q500 Ultra Sonicator). The disrupted cells were centrifuged at 65,000 × *g* for 45 min at 4 °C twice to pellet insoluble materials. The cell pellets were discarded, and the supernatant was filtered through a 0.22 µm filter (Termo CH2225-CA) before being transferred into a dialysis bag. The bag was allowed to sit in a glass beaker with 1000 mL of GluER storage buffer (100 mM TEOA, pH 7.0) at 4 °C for 12 h before the exchanged buffer was decanted and the beaker was refilled with 1000 mL of freshly prepared GluER storage buffer (100 mM TEOA, pH 7.0). The procedure was performed three more times. The dialyzed lysate was then flash-frozen in liquid N_2. The frozen lysates were lyophilized for 48 h at room temperature. The lyophilized lysates were quantified by stain-free sodium dodecyl sulfate-polyacrylamide gel electrophoresis (SDS-PAGE) against a standard curve of purified GluER variants of a known concentration.

2. DNA and protein sequences.

N-terminal His$_6$-tagged **GluER T36A Y343W** DNA sequence

ATGCACCACCATCACCACCACCCGACCCTTTTCGACCCCATCGATTTCGGACCTATC
CACGCCAAGAATCGTATCGTCATGTCCCCCCTGACTCGCGGTCGCGCTGACAAAGAG
GCGGTTCCAGCCCCCATTATGGCTGAATACTACGCCCAACGCGCTTCGGCGGGTTTA
ATTATCACTGAAGCGACGGGGATTTCACGCGAAGGCTTAGGTTGGCCGTTTGCGCCG
GGAATTTGGTCCGATGCACAGGTTGAGGCGTGGAAACCTATCGTCGCGGGTGTCCAT
GCAAAGGGCGGCAAGATCGTATGTCAGCTTTGGCATATGGGCCGTATGGTACATTCT
TCAGTTACAGGGACGCAGCCCGTAAGCAGTTCCGCCACTACTGCTCCAGGTGAGGTT
CACACCTATGAGGGCAAGAAGCCCTTCGAACAAGCGCGTGCAATCGATGCTGCAGA
CATCTCCCGCATCCTTAACGATTACGAAAATGCAGCACGTAATGCAATCCGCGCGGG
TTTCGATGGAGTGCAGATCCACGCAGCCAATGGCTACCTTATCGATGAGTTTTTGCG
TAACGGAACCAATCATCGCACCGATGAGTATGGGGGGGTGCCGGAGAACCGTATTC
GTTTCTTGAAAGAGGTAACAGAACGCGTCATCGCGGCGATTGGCGCTGACCGTACG
GGTGTGCGTCTGAGTCCAAACGGTGACACACAGGGTTGTATCGACAGTGCTCCCGA
AACCGTTTTTGTTCCTGCCGCAAAGCTTTTGCAAGATTTAGGGGTAGCGTGGCTTGA
GCTGCGTGAACCTGGTCCGAATGGTACGTTTGGAAAGACGGATCAACCAAAATTAT
CTCCACAAATCCGTAAGGTATTCCTTCGTCCATTGGTCTTAAATCAAGACTATACTTT
TGAGGCCGCACAGACGGCCCTGGCTGAGGGCAAGGCGGACGCTATTGCGTTTGGCC
GTAAGTTCATTTCAAATCCAGACTTGCCTGAGCGCTTTGCCCGTGGCATCGCACTGC
AACCAGACGATATGAAAACATGGTGGTCCCAAGGCCCAGAGGGTTACACAGACTAT
CCATCCGCAACTTCTGGGCCGAACAAT

N-terminal His$_6$-tagged **GluER T36A Y343W** amino acid sequence

MHHHHHHPTLFDPIDFGPIHAKNRIVMSPLTRGRADKEAVPAPIMAEYYAQRASAGLIIT
EATGISREGLGWPFAPGIWSDAQVEAWKPIVAGVHAKGGKIVCQLWHMGRMVHSSVT
GTQPVSSSATTAPGEVHTYEGKKPFEQARAIDAADISRILNDYENAARNAIRAGFDGVQI
HAANGYLIDEFLRNGTNHRTDEYGGVPENRIRFLKEVTERVIAAIGADRTGVRLSPNGDT
QGCIDSAPETVFVPAAKLLQDLGVAWLELREPGPNGTFGKTDQPKLSPQIRKVFLRPLVL
NQDYTFEAAQTALAEGKADAIAFGRKFISNPDLPERFARGIALQPDDMKTWWSQGPEG
YTDYPSATSGPNN

N-terminal His$_6$-tagged **GluER T36A Y177F M102G M105V A44E W66M** DNA sequence

ATGCACCACCATCACCACCACCCGACCCTTTTCGACCCCATCGATTTCGGACCTATC
CACGCCAAGAATCGTATCGTCATGTCCCCCCTGACTCGCGGTCGCGCTGACAAAGAG
GCGGTTCCAGCCCCCATTATGGCTGAATACTACGAACAACGCGCTTCGGCGGGTTTA
ATTATCACTGAAGCGACGGGGATTTCACGCGAAGGCTTAGGTATGCCGTTTGCGCCG
GGAATTTGGTCCGATGCACAGGTTGAGGCGTGGAAACCTATCGTCGCGGGTGTCCAT
GCAAAGGGCGGCAAGATCGTATGTCAGCTTTGGCATGGTGGCCGTGTGGTACATTCT
TCAGTTACAGGGACGCAGCCCGTAAGCAGTTCCGCCACTACTGCTCCAGGTGAGGTT
CACACCTATGAGGGCAAGAAGCCCTTCGAACAAGCGCGTGCAATCGATGCTGCAGA
CATCTCCCGCATCCTTAACGATTACGAAAATGCAGCACGTAATGCAATCCGCGCGGG
TTTCGATGGAGTCCAGATCCACGCAGCCAATGGCTTTCTTATCGATGAGTTTTTGCGT
AACGGAACCAATCATCGCACCGATGAGTATGGGGGGGTGCCGGAGAACCGTATTCG
TTTCTTGAAAGAGGTAACAGAACGCGTCATCGCGGCGATTGGCGCTGACCGTACGG

GTGTGCGTCTGAGTCCAAACGGTGACACACAGGGTTGTATCGACAGTGCTCCCGAA
ACCGTTTTTGTTCCTGCCGCAAAGCTTTTGCAAGATTTAGGGGTAGCGTGGCTTGAG
CTGCGTGAACCTGGTCCGAATGGTACGTTTGGAAAGACGGATCAACCAAAATTATCT
CCACAAATCCGTAAGGTATTCCTTCGTCCATTGGTCTTAAATCAAGACTATACTTTTG
AGGCCGCACAGACGGCCCTGGCTGAGGGCAAGGCGGACGCTATTGCGTTTGGCCGT
AAGTTCATTTCAAATCCAGACTTGCCTGAGCGCTTTGCCCGTGGCATCGCACTGCAA
CCAGACGATATGAAAACATGGTACTCCCAAGGCCCAGAGGGTTACACAGACTATCC
ATCCGCAACTTCTGGGCCGAACAAT

N-terminal His$_6$-tagged **GluER T36A Y177F M102G M105V A44E W66M** amino acid sequence

MHHHHHHPTLFDPIDFGPIHAKNRIVMSPLTRGRADKEAVPAPIMAEYYEQRASAGLIIT
EATGISREGLGMPFAPGIWSDAQVEAWKPIVAGVHAKGGKIVCQLWHGGRVVHSSVTG
TQPVSSSATTAPGEVHTYEGKKPFEQARAIDAADISRILNDYENAARNAIRAGFDGVQIH
AANGFLIDEFLRNGTNHRTDEYGGVPENRIRFLKEVTERVIAAIGADRTGVRLSPNGDTQ
GCIDSAPETVFVPAAKLLQDLGVAWLELREPGPNGTFGKTDQPKLSPQIRKVFLRPLVLN
QDYTFEAAQTALAEGKADAIAFGRKFISNPDLPERFARGIALQPDDMKTWYSQGPEGYT
DYPSATSGPNN

N-terminal His$_6$-tagged **GluER T36A Y177F M102G M105V A44E W66M Y343F** DNA sequence

ATGCACCACCATCACCACCACCCGACCCTTTTCGACCCCATCGATTTCGGACCTATC
CACGCCAAGAATCGTATCGTCATGTCCCCCCTGACTCGCGGTCGCGCTGACAAAGAG
GCGGTTCCAGCCCCCATTATGGCTGAATACTACGAACAACGCGCTTCGGCGGGTTTA
ATTATCACTGAAGCGACGGGGATTTCACGCGAAGGCTTAGGTATGCCGTTTGCGCCG
GGAATTTGGTCCGATGCACAGGTTGAGGCGTGGAAACCTATCGTCGCGGGTGTCCAT
GCAAAGGGCGGCAAGATCGTATGTCAGCTTTGGCATGGTGGCCGTGTGGTACATTCT
TCAGTTACAGGGACGCAGCCCGTAAGCAGTTCCGCCACTACTGCTCCAGGTGAGGTT
CACACCTATGAGGGCAAGAAGCCCTTCGAACAAGCGCGTGCAATCGATGCTGCAGA
CATCTCCCGCATCCTTAACGATTACGAAAATGCAGCACGTAATGCAATCCGCGCGGG
TTTCGATGGAGTCCAGATCCACGCAGCCAATGGCTTTCTTATCGATGAGTTTTTGCGT
AACGGAACCAATCATCGCACCGATGAGTATGGGGGGGTGCCGGAGAACCGTATTCG
TTTCTTGAAAGAGGTAACAGAACGCGTCATCGCGGCGATTGGCGCTGACCGTACGG
GTGTGCGTCTGAGTCCAAACGGTGACACACAGGGTTGTATCGACAGTGCTCCCGAA
ACCGTTTTTGTTCCTGCCGCAAAGCTTTTGCAAGATTTAGGGGTAGCGTGGCTTGAG
CTGCGTGAACCTGGTCCGAATGGTACGTTTGGAAAGACGGATCAACCAAAATTATCT
CCACAAATCCGTAAGGTATTCCTTCGTCCATTGGTCTTAAATCAAGACTATACTTTTG
AGGCCGCACAGACGGCCCTGGCTGAGGGCAAGGCGGACGCTATTGCGTTTGGCCGT
AAGTTCATTTCAAATCCAGACTTGCCTGAGCGCTTTGCCCGTGGCATCGCACTGCAA
CCAGACGATATGAAAACATGGTTCTCCCAAGGCCCAGAGGGTTACACAGACTATCC
ATCCGCAACTTCTGGGCCGAACAAT

N-terminal His$_6$-tagged **GluER T36A Y177F M102G M105V A44E W66M Y343F** amino acid sequence

MHHHHHHPTLFDPIDFGPIHAKNRIVMSPLTRGRADKEAVPAPIMAEYYEQRASAGLIIT
EATGISREGLGMPFAPGIWSDAQVEAWKPIVAGVHAKGGKIVCQLWHGGRVVHSSVTG
TQPVSSSATTAPGEVHTYEGKKPFEQARAIDAADISRILNDYENAARNAIRAGFDGVQIH

AANGFLIDEFLRNGTNHRTDEYGGVPENRIRFLKEVTERVIAAIGADRTGVRLSPNGDTQ
GCIDSAPETVFVPAAKLLQDLGVAWLELREPGPNGTFGKTDQPKLSPQIRKVFLRPLVLN
QDYTFEAAQTALAEGKADAIAFGRKFISNPDLPERFARGIALQPDDMKTWFSQGPEGYT
DYPSATSGPNN

3. Development of the photoenzymatic hydroalkylation reaction.

3.1 General procedures for photoenzymatic reactions.

All analytical photoenzymatic reactions were carried out in 1.5-dram shell vials equipped with magnetic stir bars and sealed with rubber septa unless otherwise noted. Aqueous buffer solutions and organic solvents were sparged with N_2 for 14 h and subsequently stored in a nitrogen-filled MBraun Unilab^{pro} SP box (O_2 level < 5 ppm, glovebox hereafter). Known quantities of each reaction component were transferred into the glovebox in 1-dram vials, followed by the addition of a measured volume of solvent or buffer to prepare stock solutions at the calculated concentration. Enzyme aliquots, prepared according to the procedures described in Section 1.7, were thawed and brought into the glovebox immediately prior to use. Reactions were set up inside the glovebox. After sealing with a rubber septum, the reaction vials were removed from the glovebox and placed under the designated light conditions, with the appropriate cooling method applied.

3.2 Site-saturation mutagenesis and construction of site-saturation libraries.

Site-saturation mutagenesis primers were designed following the protocol described by Acevedo and co-workers.[5] Polymerase chain reaction (PCR) products generated using these primers were digested with DpnI and subsequently repaired using Gibson Mix™. The resulting mixtures were used directly to transform *E. coli* BL21 electrocompetent cells, which were plated on LB agar supplemented with ampicillin (100 µg/mL).

Single colonies for library construction were picked with sterile toothpicks and used to inoculate 500 µL overnight cultures in sterile deep-well 96-well plates containing LB medium with ampicillin (100 µg/mL). Cultures were grown at 30 °C and 250 rpm. Wells A1, B2, C4, D6, E8, F10, and G11 contained cells with DNA encoding the parent protein as internal controls, and well H12 served as a blank (no cells). A glycerol stock of the resulting library was prepared by mixing sterilized glycerol (50% v/v, 100 µL per well) with aliquots of the overnight cultures (100 µL per well). The plate was sealed and stored at –80 °C.

In parallel, expression cultures were prepared in sterile deep-well 96-well plates. Each well contained 960 µL TB (100 µg/mL ampicillin), 40 µL auto-inducing mix, and 50 µL of the corresponding overnight culture. Plates were incubated for 24 h at 30 °C and 250 rpm, then harvested by centrifugation (4000 × *g*, 15 min, 4 °C). Supernatants were discarded, and the resulting cell pellets were stored at –80 °C.

3.3 Screening procedure in 96-well plates.

The cell libraries were thawed at 23°C for 15 min. Lysis buffer was prepared by dissolving lysozyme (15 mg), DNase I (1.5 mg), and PMSF (1 mM, added as a 35 mg/mL solution in absolute ethanol, 150 µL) in 15 mL of the appropriate aqueous buffer (see Section 3.6). Thawed cell pellets were resuspended in lysis buffer (130 µL per well), and the deep-well 96-well plate was sealed with a reusable silicone mat and incubated for 1 h at 37 °C with shaking at 250 rpm. The unpurified lysates were clarified by centrifugation (4000 × *g*, 30 min, 4 °C), and supernatants (100 µL per well) were transferred immediately into a white optical 96-well plate with a clear flat bottom (Corning Costar® Assay Plate). Each well contained 100 µL of turnover mix solution, prepared by dissolving D-glucose (324 mg), GDH (22.5 mg), $NADP^+$ (2.25 mg), and FMN (1.5 mg) in 15 mL of the appropriate aqueous buffer.

The plate was transferred into the glovebox. Stock solutions of the substrates were prepared inside the glovebox by dissolving the appropriate amounts of 2-bromoacetamide (**19**) (or 2-chloroacetamide, **53**) and the ester **18** in dimethyl sulfoxide. Substrates were dispensed into each well using a repeat pipettor such that each reaction contained 5 μmol of the limiting reagent. The plate was sealed with adhesive polyester sealing film (BrandTech® 701390ES), placed on a plate shaker (400 rpm) under a fan, and irradiated at room temperature under anaerobic conditions for 16 h using a Lumidox® 96-well cyan LED array (505 nm, 70 mV per well).

Reactions were quenched by the addition of acetonitrile (600 μL per well). The plate was sealed with a reusable silicone mat, shaken for 30 min, and centrifuged (4500 × *g*, 20 min). Supernatants (220 μL per well) were filtered through a Millipore 96-well filter plate into a shallow-well collection plate (900 × *g*, 1 min).

For yield-based screenings, filtrates were analyzed by RapidFire 400 high-resolution mass spectrometry. For enantioselectivity-based screenings, filtrates were analyzed using a Shimadzu LC-2050C system equipped with a CHIRALCEL® OX-3R column (4.6 × 250 mm, 3 μm) and water/acetonitrile mobile phases. Promising hits were cultured in shaking flasks and used in analytical-scale photoenzymatic reactions under appropriate conditions for validation. Plasmids from validated hits were isolated and submitted for sequencing to determine the corresponding mutations.

3.4 Site-directed mutagenesis.

Site-directed mutagenesis primers were designed to introduce the desired mutation at the target site. PCR products generated using these primers were digested with DpnI and subsequently repaired using Gibson Mix™. The products were directly used to transform BL21 *E. coli* electrocompetent cells. Single colonies were inoculated into a 5 mL overnight culture in LB media with ampicillin (100 μg/mL) at 37 °C and 250 rpm with a sterile inoculation loop. A glycerol stock of the resulting variant was prepared by mixing 50% v/v sterilized glycerol (2 mL) with aliquots of the overnight cultures (2 mL) in a sterile Eppendorf tube. The tube was sealed and stored at –80 °C, and was used for the expression and purification of the mutated variant following the procedure detailed in Section 1.7.

3.5 Supplementary Tables for the development of the photoenzymatic hydroalkylation reaction with GluER HA$_{rac}$.

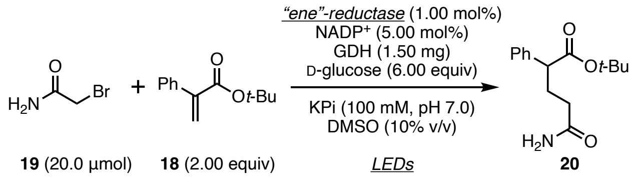

entry	"ene"-reductase	yield (with blue light)	yield (with cyan light)
1	Nostoc	5%	–
2	OYE3	6%	–
3	OYE2	13%	–
4	MORB	17%	18%
5	PhenER	4%	–
6	YersER	12%	–
7	GKOYE	26%	–
8	CsER	7%	6%
9	YqjM	8%	3%
10	TOYE	7%	2%
11	GluER-T36A	12%	<1%
12	GluER-T36A-Y343F	28%	12%
13	GluER-T36A-Y343W	15%	**35%**

Table S1. Initial screen of "ene"-reductases for the photoenzymatic hydroalkylation under blue or cyan LEDs irradiation. Yields were determined by LC-MS analysis using TBB as an internal standard and quantified against a standard calibration curve.

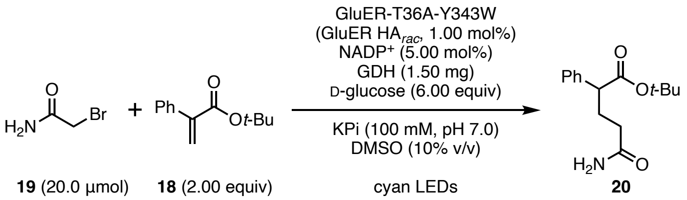

entry	deviation from the shown conditions	yield
1	–	35%
2	add $Ru(bpy)_3Cl_2$ (1.00 mol%)	33%
3	replace buffer with MES buffer (pH 6.0, 100 mM)	32%
4	replace buffer with MOPS buffer (pH 8.0, 100 mM)	21%
5	replace buffer with KPi buffer (pH 8.0, 100 mM)	8%
6	replace buffer with with Tricine buffer (pH 9.0, 100 mM)	84%
7	use 20 μmol **18** as the limiting reagent and 2.00 equiv of **19**	67%
8	entry 7, replace buffer with Tricine buffer (pH 9.0, 100 mM)	85%
9	entry 8, with 26.7 μmol **18**, 0.75 mol% enzyme, 2.00 mol% NADP$^+$, 2.00 mg GDH, and 2.00 equiv D-glucose	**91%**
10	entry 9, with 1.00 mol% GluER-T36A	75%

Table S2. Optimization of reaction conditions for the photoenzymatic hydroalkylation with GluER-T36A-Y343W (GluER HA$_{rac}$). Yields were determined by LC-MS analysis using TBB as an internal standard and quantified against a standard calibration curve.

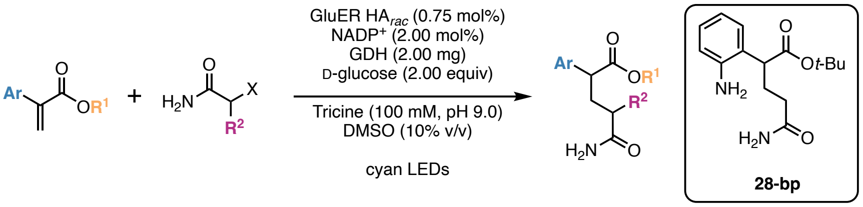

entry	substrate	result
1	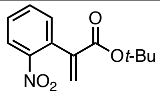 **28-s**	17% yield, 75% **28-s** recovered, ~5% **28-bp** detected by ^{1}H NMR.
2	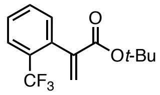 **S1**	<5% yield, **S1** was not recovered.
3	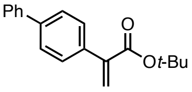 **S2a**	no reaction, >90% **S2a** recovered.
4	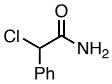 **S2b**	no reaction, >90% **S2b** recovered
5	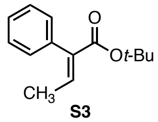 **S3**	no reaction, ~80% **S3** recovered
6	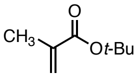 **S4a**	<5% yield, **S4a** was not recovered
7	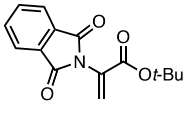 **S4b**	no reaction, >90% **S4b** recovered

Table S3. Limitations of the photoenzymatic hydroalkylation with GluER HA$_{rac}$. Results were determined by NMR analysis of the unpurified product mixture using 1,3,5-trimethoxybenzene (TMB) as an internal standard.

3.6 Development of the photoenzymatic hydroalkylation reaction with GluER HA*ent*.

Summary of the 1st round site-saturation mutagenesis.

All mutants tested in this round showed no improvement in enantioselectivity; selected data are shown in Table S4. Accordingly, screening of GluER mutants was conducted, and enzymes that catalyze the reaction with improved enantiomeric selectivity are listed in Table S5. Based on the screening result, **GluER-T36A-Y177F** was chosen as the template for the next round of engineering.

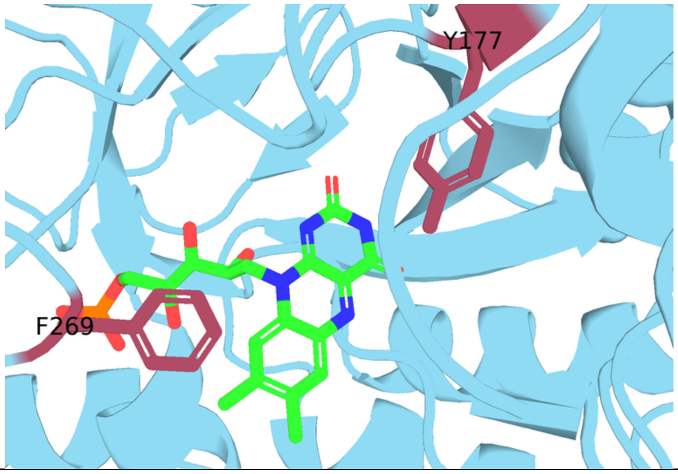

template	target sites	beneficial mutation
GluER-T36A-Y343W	Y177, F269	–

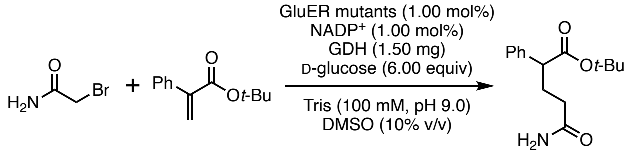

19 (20.0 µmol) **18** (2.00 equiv) cyan LEDs **20**

mutant	yield	er
GluER-T36A-Y343W	54%	50:50
GluER-T36A-Y343W-Y177A	62%	52:48
GluER-T36A-Y343W-Y177D	59%	50:50
GluER-T36A-Y343W-Y177G	53%	51:49
GluER-T36A-Y343W-Y177K	51%	50:50
GluER-T36A-Y343W-Y177V	53%	49:51

Table S4. First-round mutants showed no improvement in enantioselectivity. Yields were determined by LC-MS analysis using TBB as an internal standard and quantified against a standard calibration curve. Enantiomeric ratios were determined using a Shimadzu LC-2050C liquid chromatograph system with water and acetonitrile as mobile phases equipped with CHIRALCEL® OX-3R column (4.6 × 250 mm, 3 µm).

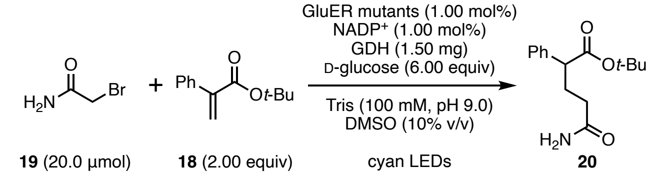

mutant	yield	er
GluER-T36A-K317M-Y343F	51%	51:49
**GluER-T36A-Y177F**	**14%**	**65:35**
GluER-T36A-Y177W	8%	57:43
GluER-T36A-F269A	5%	56:44
GluER-T36A-F269W	14%	57:43

Table S5. Reevaluation of GluER mutants found GluER-T36A-Y177F as the new parent enzyme. Yields were determined by LC-MS analysis using TBB as an internal standard and quantified against a standard calibration curve. Enantiomeric ratios were determined using a Shimadzu LC-2050C liquid chromatograph system with water and acetonitrile as mobile phases equipped with CHIRALCEL® OX-3R column (4.6 × 250 mm, 3 μm).

Summary of the 2nd round site-saturation mutagenesis.

The goal of the 2nd round of site-saturation mutagenesis was to identify mutants that catalyze the reaction with improved yield and minimal decrease in enantiomeric selectivity. Condition optimization found that the use of 2-chloroacetamide (**53**) improves enantioselectivity compared to 2-bromoacetamide (**19**; Table S6). Under the optimized conditions, **GluER-T36A-Y177F-W66T** was chosen as the template for the next round of engineering (Table S7). Additionally, we found that all variants with Y343 mutated showed significantly decreased enantioselectivity (Table S8).

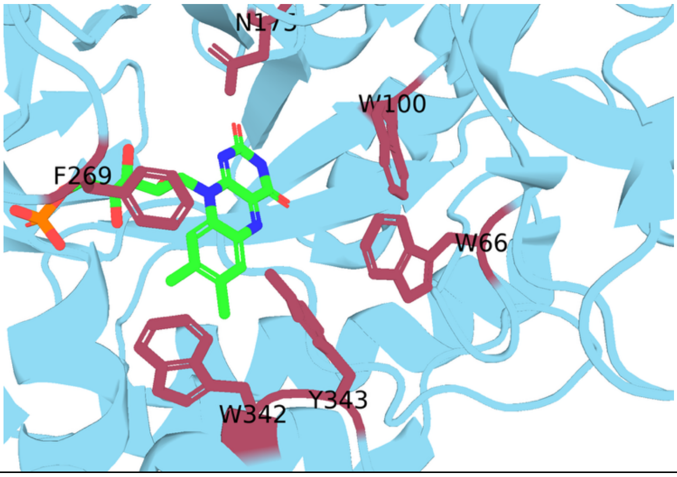

template	target sites	beneficial mutation
GluER-T36A-Y177F	W66, W100, N175, F269, W342, Y343	W66T

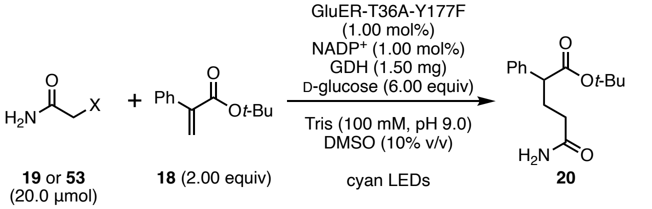

2-haloacetamide employed	yield	er
**19**, X = Br	14%	65:35
**53**, X = Cl	11%	78:22

Table S6. Investigation of 2-haloacetamide **19** and **53** for enantioselectivity. Yields were determined by LC-MS analysis using TBB as an internal standard and quantified against a standard calibration curve. Enantiomeric ratios were determined using a Shimadzu LC-2050C liquid chromatograph system with water and acetonitrile as mobile phases equipped with CHIRALCEL® OX-3R column (4.6 × 250 mm, 3 µm).

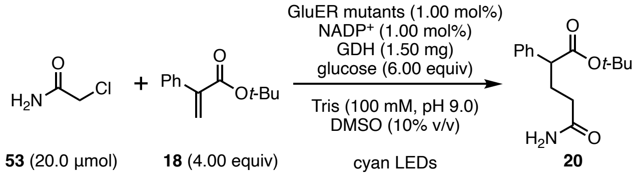

mutant	yield	er
GluER-T36A-Y177F	11%	78:22
**GluER-T36A-Y177F-W66T**	**49%**	**69:31**

Table S7. Analytical scale reaction conditions and results for 2nd round hits validation. Yields were determined by LC-MS analysis using TBB as an internal standard and quantified against a standard calibration curve. Enantiomeric ratios were determined using a Shimadzu LC-2050C liquid chromatograph system with water and acetonitrile as mobile phases equipped with CHIRALCEL® OX-3R column (4.6 × 250 mm, 3 μm).

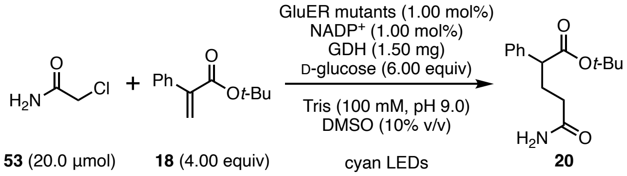

mutant	yield	er
GluER-T36A-Y177F	11%	78:22
GluER-T36A-Y177F-Y343T	21%	57:43
GluER-T36A-Y177F-Y343A	33%	55:45
GluER-T36A-Y177F-Y343G	36%	56:44
GluER-T36A-Y177F-Y343C	28%	57:43
GluER-T36A-Y177F-Y343K	29%	54:46

Table S8. All Y343 mutants gave low enantioselectivity. Yields were determined by LC-MS analysis using TBB as an internal standard and quantified against a standard calibration curve. Enantiomeric ratios were determined using a Shimadzu LC-2050C liquid chromatograph system with water and acetonitrile as mobile phases equipped with CHIRALCEL® OX-3R column (4.6 × 250 mm, 3 μm).

3rd Round of site-saturation mutagenesis.

Summary of the 3rd round site-saturation mutagenesis.

The goal of the 3rd round of site-saturation mutagenesis was to identify mutants that catalyze the reaction with both improved yield and improved enantiomeric selectivity. **GluER-T36A-Y177F-W66T-M102G** was chosen as the template for the next round of engineering (Table S9).

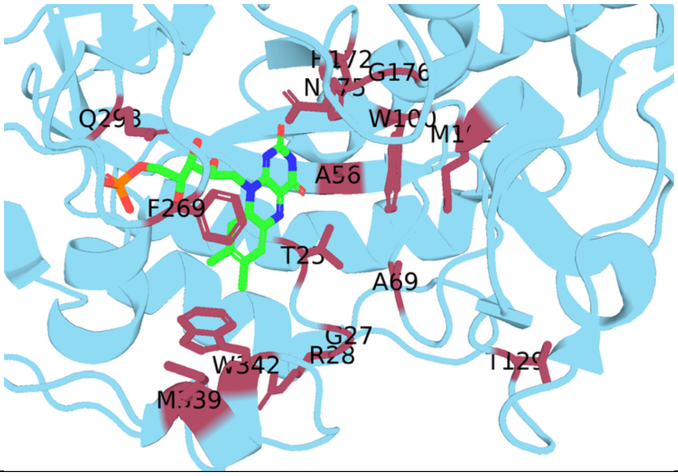

template	target sites	beneficial mutations
GluER-T36A-Y177F-W66T	T25, G27, R28, A56, A69, W100, M102, T129, H172, N175, G176, F269, Q293, M339, W342	F269A, M102G

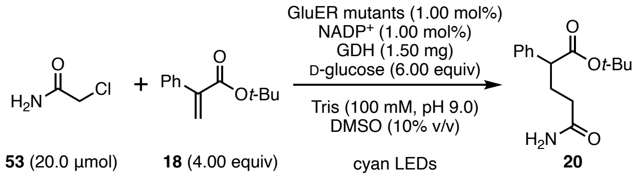

mutant	yield	er
GluER-T36A-Y177F-W66T	49%	69:31
GluER-T36A-Y177F-W66T-F269A	61%	65:35
**GluER-T36A-Y177F-W66T-M102G**	**61%**	**76:24**

Table S9. Analytical scale reaction conditions and results for 3rd round hits validation. Yields were determined by LC-MS analysis using TBB as an internal standard and quantified against a standard calibration curve. Enantiomeric ratios were determined using a Shimadzu LC-2050C liquid chromatograph system with water and acetonitrile as mobile phases equipped with CHIRALCEL® OX-3R column (4.6 × 250 mm, 3 µm).

4th Round of site-saturation mutagenesis.

Summary of the 4th round site-saturation mutagenesis.

The goal of the 4th round of site-saturation mutagenesis was to identify mutants that catalyze the reaction with either improved yield or improved enantiomeric selectivity. Condition optimization found that the use of pH 7.5 Tris buffer improves enantioselectivity (Table S10). For practical reasons, we designated the more precious acrylate **18** as the limiting reagent (Table S11). Under the optimized conditions, **GluER-T36A-Y177F-W66T-M102G-M105V** was chosen as the template for the next round of engineering (Table S12).

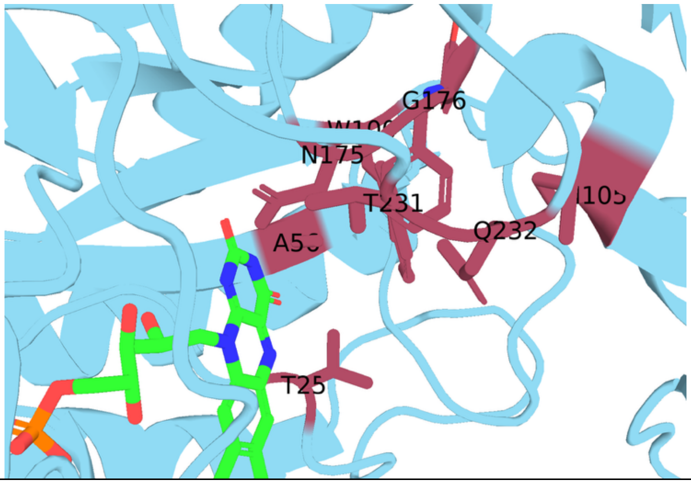

template	target sites	beneficial mutations
GluER-T36A-Y177F-W66T-M102G	T25, A56, T66, W100, M105, N175, G176, F177, T231, Q232	W66R, W66P, W100Y, M105V, M105I

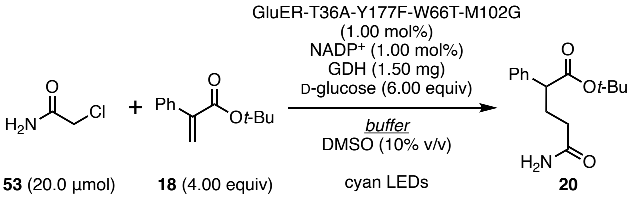

buffer (100 mM)	yield	er
MES pH 6.0	16%	90:10
MES pH 7.0	24%	88:12
Tris pH 9.0	72%	73:27
Tris pH 8.0	32%	83:17
**Tris pH 7.5**	**13%**	**90:10**
Tris pH 7.0	11%	88:12
KP_i pH 7.0	15%	88:12
CHES pH 7.0	8%	87:13
CAPS pH 7.0	7%	78:22
MOPS pH 7.0	17%	87:13

Table S10. Investigation of buffer and pH for enantioselectivity. Yields were determined by LC-MS analysis using TBB as an internal standard and quantified against a standard calibration curve. Enantiomeric ratios were determined using a Shimadzu LC-2050C liquid chromatograph system

with water and acetonitrile as mobile phases equipped with CHIRALCEL® OX-3R column (4.6 × 250 mm, 3 μm).

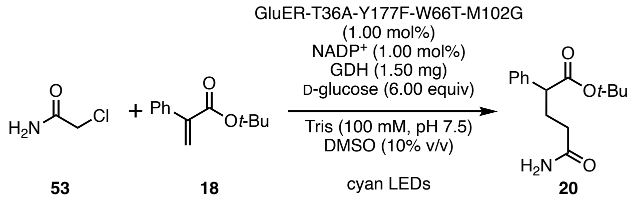

stoichiometry	yield	er
20.0 μmol of **53**, 4.00 equiv of **18**	13%	90:10
20.0 μmol of **18**, 4.00 equiv of **53**	40%	86:14

Table S11. Change of the limiting reagent from **53** to **18**. Yields were determined by LC-MS analysis using TBB as an internal standard and quantified against a standard calibration curve. Enantiomeric ratios were determined using a Shimadzu LC-2050C liquid chromatograph system with water and acetonitrile as mobile phases equipped with CHIRALCEL® OX-3R column (4.6 × 250 mm, 3 μm).

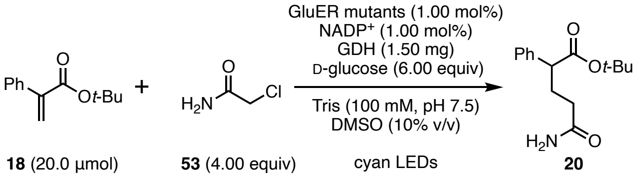

mutant	yield	er
GluER-T36A-Y177F-W66T-M102G	40%	86:14
GluER-T36A-Y177F-M102G-W66R	57%	82:18
GluER-T36A-Y177F-M102G-W66P	44%	84:16
GluER-T36A-Y177F-W66T-M102G-W100Y	58%	82:18
**GluER-T36A-Y177F-W66T-M102G-M105V**	**44%**	**88:12**
GluER-T36A-Y177F-W66T-M102G-M105I	55%	87:13

Table S12. Analytical scale reaction conditions and results for 4th round hits validation. Yields were determined by LC-MS analysis using TBB as an internal standard and quantified against a standard calibration curve. Enantiomeric ratios were determined using a Shimadzu LC-2050C liquid chromatograph system with water and acetonitrile as mobile phases equipped with CHIRALCEL® OX-3R column (4.6 × 250 mm, 3 μm).

5th Round of site-saturation mutagenesis and site-directed mutagenesis.

Summary of the 5th round site-saturation and site-directed mutagenesis.

The goal of the 5th round of site-saturation mutagenesis was to identify mutants that catalyze the reaction with either improved yield or improved enantiomeric selectivity. In conjunction with the site-saturation mutagenesis effort, we conducted computational simulations to predict beneficial mutations (for details, see Section 9.3). A point mutant **GluER-T36A-Y177F-W66T-M102G-M105V-A44E** was chosen as the template for the next round of engineering (Table S13).

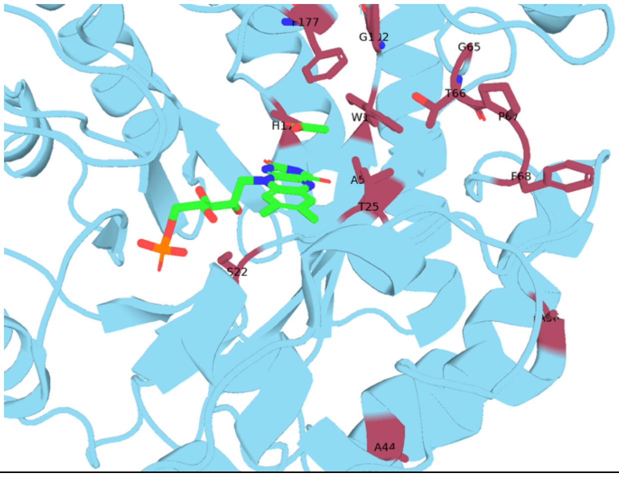

template	point mutations	target sites	beneficial mutations
GluER-T36A-Y177F-W66T-M102G-M105V	A36E, A44E, S22A, F68Y	T25, A56, G65, T66, P67, W100, G102, H172, F177	A44E, W100F

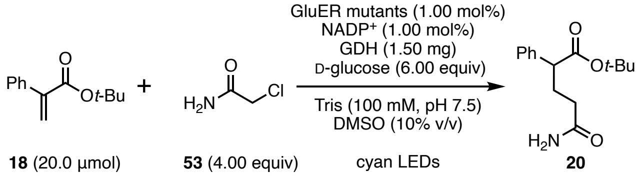

mutant	yield	er
GluER-T36A-Y177F-W66T-M102G-M105V	44%	88:12
**GluER-T36A-Y177F-W66T-M102G-M105V-A44E**	**49%**	**89:11**
GluER-T36A-Y177F-W66T-M102G-M105V-W100F	43%	88:12

Table S13. Analytical scale reaction conditions and results for 5th round hits validation. Yields were determined by LC-MS analysis using TBB as an internal standard and quantified against a standard calibration curve. Enantiomeric ratios were determined using a Shimadzu LC-2050C liquid chromatograph system with water and acetonitrile as mobile phases equipped with CHIRALCEL® OX-3R column (4.6 × 250 mm, 3 μm).

6th Round of site-saturation mutagenesis and site-directed mutagenesis.

<u>Summary of the 6th round site-saturation mutagenesis.</u>

The goal of the 6th round of site-saturation mutagenesis was to identify mutants that catalyze the reaction with improved enantiomeric selectivity. Condition optimization found that the use of pH 7.0 HEPES buffer with 20% v/v dimethyl sulfoxide (DMSO) improves enantioselectivity (Table S14). Under the optimized conditions, **GluER-T36A-Y177F-M102G-M105V- W66M** was chosen as the final enantioselective variant (Table S15). Yields and enantiomeric ratios under the optimal conditions with the mutants selected in each round are summarized in Table S16.

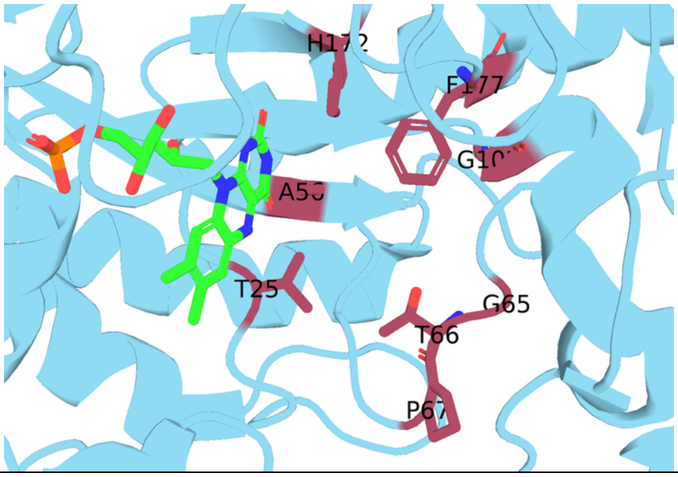

template	target sites and point mutations	beneficial mutation
GluER-T36A-Y177F-W66T-M102G-M105V-A44E	T25, A56, G65, T66, P67, G102, H172, F177	W66M

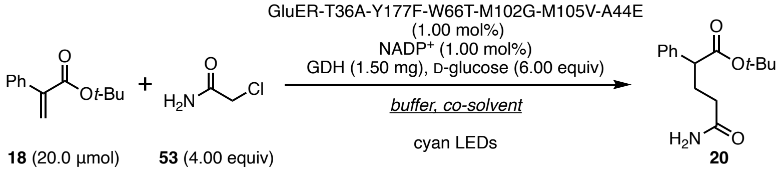

buffer (100 mM)	co-solvent	er
Tris pH 7.0	10% v/v DMSO	78:22
KPi pH 7.0	10% v/v DMSO	77:23
citrate pH 6.0	10% v/v DMSO	71:29
HEPES pH 7.0	10% v/v DMSO	82:18
HEPES pH 7.0	10% v/v CH_3OH	83:17
HEPES pH 7.0	10% v/v EtOH	86:14
**HEPES pH7.0**	**20% v/v DMSO**	**85:15**

Table S14. Optimization of buffer and co-solvent with GluER-T36A-Y177F-W66T-M102G-M105V-A44E. Enantiomeric ratio determined using a Shimadzu LC-2050C liquid chromatograph system with water and acetonitrile as mobile phases equipped with CHIRALCEL® OX-3R column (4.6 × 250 mm, 3 µm).

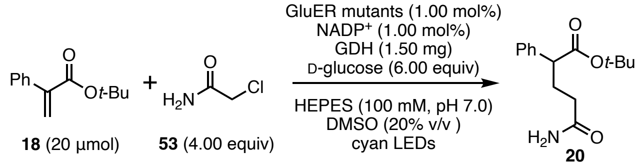

mutant	yield	er
GluER-T36A-Y177F-W66T-M102G-M105V-A44E	73%	85:15
**GluER-T36A-Y177F-M102G-M105V-A44E-W66M**	**84%**	**93:7**

Table S15. Analytical scale reaction conditions and results for 5th round hits validation. Yields were determined by LC-MS analysis using TBB as an internal standard and quantified against a standard calibration curve. Enantiomeric ratios were determined using a Shimadzu LC-2050C liquid chromatograph system with water and acetonitrile as mobile phases equipped with CHIRALCEL® OX-3R column (4.6 × 250 mm, 3 µm).

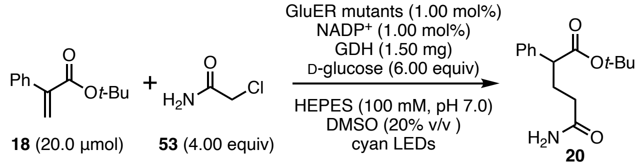

round	mutant	yield	er
1	GluER-T36A-Y177F	51%	83:17
2	GluER-T36A-Y177F-W66T	76%	78:22
3	GluER-T36A-Y177F-W66T-M102G	69%	81:19
4	GluER-T36A-Y177F-W66T-M102G-M105V	77%	82:18
5	GluER-T36A-Y177F-W66T-M102G-M105V-A44E	73%	85:15
6	GluER-T36A-Y177F-M102G-M105V-A44E-W66M	84%	93:7

Table S16. Yields and enantiomeric ratios of the photoenzymatic hydroalkylation reaction catalyzed by the chosen variants throughout the engineering campaign under the optimized conditions. Yields were determined by LC-MS analysis using TBB as an internal standard and quantified against a standard calibration curve. Enantiomeric ratio determined using a Shimadzu LC-2050C liquid chromatograph system with water and acetonitrile as mobile phases equipped with CHIRALCEL® OX-3R column (4.6 × 250 mm, 3 µm).

4. Synthetic procedures.

4.1 Synthetic procedures for the substrates 18, 21-s–52-s.

The substrates **18**, **21-s**–**52-s** for the enzymatic reactions were prepared according to General Procedures A, B, or reported procedures (see below). For compounds with no previous reports, ^{1}H NMR, ^{13}C NMR, HRMS, and IR data were recorded. For known compounds, ^{1}H NMR shifts were recorded to confirm consistency with the reported values. Due to the instability of the unsaturated ester residue under light and heat, the substrates were stored in an aluminum-foil-covered flask at –20 °C after preparation.

General Procedure A.

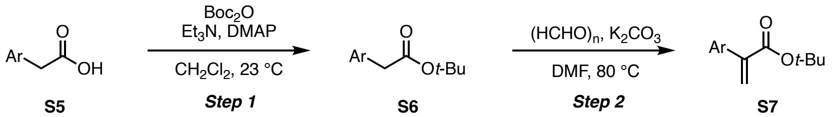

Step 1: Triethylamine (1.50 equiv) and 4-dimethylaminopyridine (DMAP, 0.10 equiv) were added in sequence to a solution of the corresponding carboxylic acid **S5** (1 equiv) in dichloromethane (0.30 M for **S5**) at 23 °C. Di-*tert*-butyl dicarbonate (Boc_2O, 1.10 equiv) was added dropwise to the reaction mixture at 23 °C over 2 min. The reaction mixture was stirred at 23 °C for 14 h. The product mixture was concentrated under reduced pressure. The residue obtained was purified by flash-column chromatography to provide the corresponding ester **S6**

Step 2: Paraformaldehyde (1.50 equiv) and potassium carbonate (1.10 equiv) were added in sequence to a solution of the corresponding ester **S6** (1 equiv) in *N*,*N*-dimethylformamide (0.15 M for **S6**) at 23 °C. The reaction vessel was placed on a metal block preheated to 80 °C, and the reaction mixture was stirred at this temperature for 4 h. The product mixture was cooled to 23 °C over 10 min, and diluted sequentially with ethyl acetate and water. The diluted product mixture was transferred to a separatory funnel, and the layers that formed were separated. The aqueous layer was extracted with ethyl acetate three times. The organic layers were combined, and the combined organic layers were dried over sodium sulfate. The dried solution was filtered, and the filtrate was concentrated. The residue obtained was purified by flash-column chromatography to provide the substrate **S7**.

General Procedure B.

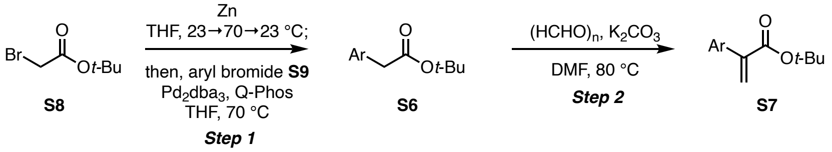

Step 1: This procedure is modified from that reported by Hartwig and co-workers.[6]

tert-Butyl 2-bromoacetate (**S8**, 2.00 equiv) was added dropwise over 2 min to a suspension of activated zinc powder (2.40 equiv) in tetrahydrofuran (0.50 M for the aryl bromide **S9**) at 23 °C.

The reaction vessel was placed on a metal block preheated to 70 °C, and the reaction mixture was stirred at this temperature for 5 min. The reaction mixture was then allowed to cool to 23 °C over 10 min. In a separate vial, the arylbromide **S9** (1 equiv), tris(dibenzylideneacetone)dipalladium(0) (Pd_2dba_3, 1.00 mol%), and 1,2,3,4,5-Pentaphenyl-1′-(di-*tert*-butylphosphino)ferrocene (Q Phos, 1.00 mol%) were dissolved in tetrahydrofuran (0.50 M for the arylbromide **S9**), and the solutionwas added to the reaction mixture at 23 °C. The reaction vessel was then placed on a metal block preheated to 70 °C, and the reaction mixture was stirred at this temperature for 6 h. The product mixture was allowed to cool to 23 °C, and was diluted sequentially with ethyl acetate and water. The diluted product mixture was transferred to a separatory funnel, and the layers that formed were separated. The aqueous layer was extracted with ethyl acetate three times. The organic layers were combined, and the combined organic layers were dried over sodium sulfate. The dried solution was filtered, and the filtrate was concentrated. The residue obtained was purified by flash-column chromatography to provide the ester **S6**.

<u>Step 2:</u> Identical to that of General Procedure A.

*Synthesis of the substrates **18** and **21-s–52-s**.*

tert-butyl 2-phenylacrylate (**18**)

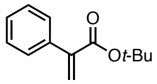

18

The ester **18** was prepared according to the procedure reported by Tan and co-workers.[7]
^{1}H NMR (500 MHz, $CDCl_3$): δ 7.44 – 7.39 (m, 2H), 7.37 – 7.29 (m, 3H), 6.24 (d, *J* = 1.4 Hz, 1H), 5.82 (d, *J* = 1.4 Hz, 1H), 1.53 (s, 9H).

tert-butyl 2-(*o*-tolyl)acrylate (**21-s**)

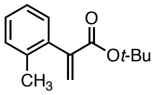

21-s

The ester **21-s** was prepared according to General Procedure A in 57% overall yield.
^{1}H NMR (500 MHz, $CDCl_3$): δ 7.25 – 7.21 (m, 1H), 7.19 – 7.16 (m, 2H), 7.14 (td, *J* = 7.7, 1.4 Hz, 1H), 6.39 (d, *J* = 1.8 Hz, 1H), 5.64 (d, *J* = 1.8 Hz, 1H), 2.22 (s, 3H), 1.48 (s, 9H). ^{13}C NMR (126 MHz, $CDCl_3$): δ 165.8, 143.6, 137.8, 135.9, 129.7, 129.5, 127.8, 127.0, 125.5, 80.9, 27.9, 19.8. IR (FTIR), cm^{-1}: 2980, 2933, 1735, 1718, 1370, 1254, 1144, 742.
*Note: Due to poor ionization, the calculated mass was not observed for **21-s** on HRMS. The calculated mass for the corresponding product **21** was observed (see Section 5.1).*

tert-butyl 2-(*m*-tolyl)acrylate (**22-s**)

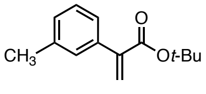

22-s

The ester **22-s** was prepared according to the procedure reported by Tan and co-workers.[7]
^{1}H NMR (500 MHz, $CDCl_3$): δ 7.27 – 7.21 (m, 3H), 7.16 – 7.13 (m, 1H), 6.23 (d, *J* = 1.4 Hz, 1H), 5.82 (d, *J* = 1.4 Hz, 1H), 2.38 (s, 3H), 1.55 (s, 9H).

tert-butyl 2-(*p*-tolyl)acrylate (**23-s**)

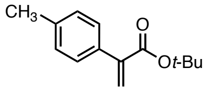

23-s

The ester **23-s** was prepared according to the procedure reported by Tan and co-workers.[7]
^{1}H NMR (500 MHz, $CDCl_3$): δ 7.31 (d, *J* = 8.2 Hz, 2H), 7.15 (d, *J* = 7.8 Hz, 2H), 6.19 (d, *J* = 1.5 Hz, 1H), 5.78 (d, *J* = 1.4 Hz, 1H), 2.36 (s, 3H), 1.53 (s, 9H).

tert-butyl 2-(2-methoxyphenyl)acrylate (**24-s**)

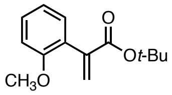

24-s

The ester **24-s** was prepared according to General Procedure B in 47% overall yield.

^{1}H NMR (300 MHz, $CDCl_3$): δ 7.34 – 7.27 (m, 1H), 7.21 (dd, *J* = 7.4, 1.8 Hz, 1H), 6.94 (td, *J* = 7.4, 1.1 Hz, 1H), 6.87 (dd, *J* = 8.3, 1.1 Hz, 1H), 6.17 (d, *J* = 1.7 Hz, 1H), 5.67 (d, *J* = 1.7 Hz, 1H), 3.80 (s, 3H), 1.48 (s, 9H). ^{13}C NMR (126 MHz, $CDCl_3$): δ 166.6, 156.8, 141.8, 130.1, 129.5, 127.6, 124.9, 120.5, 110.4, 80.5, 55.3, 28.0. IR (FTIR), cm^{-1}: 2980, 2937, 1716, 1492, 1368, 1246, 1161, 751. HRMS (ESI-TOF), m/z: $[M – C_4H_9O]^+$ calculated for $[C_{10}H_9O_2]^+$, 161.0597; found 161.0593.

tert-butyl 2-(2-chlorophenyl)acrylate (**25-s**)

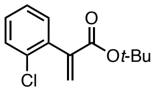

25-s

The ester **25-s** was prepared according to General Procedure A in 79% overall yield.
^{1}H NMR (500 MHz, $CDCl_3$): δ 7.41 – 7.34 (m, 1H), 7.31 – 7.22 (m, 3H), 6.42 (d, *J* = 1.5 Hz, 1H), 5.71 (d, *J* = 1.5 Hz, 1H), 1.48 (s, 9H). ^{13}C NMR (126 MHz, $CDCl_3$): δ 165.0, 142.1, 137.2, 133.2, 130.9, 129.1, 129.1, 127.7, 126.6, 81.3, 27.9. IR (FTIR), cm^{-1}: 2980, 2935, 1718, 1369, 1256, 1159, 1103, 757.

*Note: Due to poor ionization, the calculated mass was not observed for **25-s**. The calculated mass for the corresponding product **25** was observed and documented.*

tert-butyl 2-(2-bromophenyl)acrylate (**26-s**)

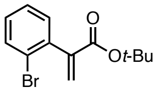

26-s

The ester **26-s** was prepared according to the procedure reported by Wang and co-workers.[8]
^{1}H NMR (300 MHz, $CDCl_3$): δ 7.56 (dd, *J* = 7.9, 1.3 Hz, 1H), 7.37 – 7.27 (m, 1H), 7.23 – 7.14 (m, 2H), 6.41 (d, *J* = 1.5 Hz, 1H), 5.68 (d, *J* = 1.5 Hz, 1H), 1.48 (s, 9H).

tert-butyl 2-(2-iodophenyl)acrylate (**27-s**)

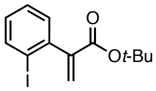

27-s

The ester **27-s** was prepared according to General Procedure A in 81% overall yield.
^{1}H NMR (500 MHz, $CDCl_3$): δ 7.83 (dd, *J* = 7.9, 1.2 Hz, 1H), 7.34 (td, *J* = 7.5, 1.2 Hz, 1H), 7.21 (dd, *J* = 7.6, 1.7 Hz, 1H), 7.00 (td, *J* = 7.7, 1.7 Hz, 1H), 6.44 (d, *J* = 1.5 Hz, 1H), 5.65 (d, *J* = 1.5 Hz, 1H), 1.49 (s, 9H). ^{13}C NMR (126 MHz, $CDCl_3$): δ 164.4, 146.3, 143.2, 138.7, 130.1, 129.1, 127.9, 127.7, 98.6, 81.3, 27.9. IR (FTIR), cm^{-1}: 2980, 2933, 1716, 1467, 1370, 1254, 1161,1016, 729. HRMS (ESI-TOF), m/z: $[M – C_4H_9O + H_2O]^+$ calculated for $[C_9H_8IO_2]^+$, 274.9564; found 274.9559.

tert-butyl 2-(2-nitrophenyl)acrylate (**28-s**)

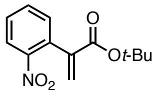

28-s

The ester **28-s** was prepared according to General Procedure A in 67% overall yield.

^{1}H NMR (500 MHz, $CDCl_3$): δ 8.08 (dd, J = 8.2, 1.3 Hz, 1H), 7.62 (td, J = 7.5, 1.3 Hz, 1H), 7.50 (td, J = 7.8, 1.5 Hz, 1H), 7.38 (dd, J = 7.5, 1.5 Hz, 1H), 6.47 (d, J = 1.1 Hz, 1H), 5.81 (d, J = 1.1 Hz, 1H), 1.42 (s, 9H). ^{13}C NMR (126 MHz, $CDCl_3$): δ 163.7, 148.2, 141.5, 133.5, 133.5, 132.1, 129.0, 126.5, 124.4, 82.1, 27.8. IR (FTIR), cm^{-1}: 2980, 2937, 1716, 1492, 1368, 1246, 1161, 751. HRMS (ESI-TOF), m/z: [M + Na]$^+$ calculated for $[C_{13}H_{15}NNaO_4]^+$, 272.0893; found 272.0885.

tert-butyl 2-(3-bromophenyl)acrylate (**29-s**)

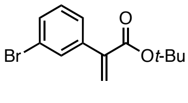

29-s

The ester **29-s** was prepared according to General Procedure A in 77% overall yield.
^{1}H NMR (500 MHz, $CDCl_3$): δ 7.57 (t, J = 1.8 Hz, 1H), 7.44 (ddd, J = 8.0, 2.0, 1.0 Hz, 1H), 7.34 (dt, J = 7.8, 1.3 Hz, 1H), 7.21 (t, J = 7.9 Hz, 1H), 6.29 (d, J = 1.2 Hz, 1H), 5.83 (d, J = 1.2 Hz, 1H), 1.53 (s, 9H). ^{13}C NMR (126 MHz, $CDCl_3$): δ 165.4, 141.6, 139.1, 131.4, 130.9, 129.4, 127.1, 126.8, 122.0, 81.6, 28.0. IR (FTIR), cm^{-1}: 2980, 2935, 1716, 1477, 1370, 1157, 851, 788. HRMS (ESI-TOF), m/z: [2M + Na]$^+$ calculated for $[C_{26}H_{30}Br_2NaO_4]^+$, 587.0403; found 587.0407.

tert-butyl 2-(4-bromophenyl)acrylate (**30-s**)

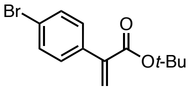

30-s

The ester **30-s** was prepared according to the procedure reported by Wang and co-workers.[8]
^{1}H NMR (500 MHz, $CDCl_3$): δ 7.47 (d, J = 8.5 Hz, 2H), 7.28 (d, J = 8.5 Hz, 2H), 6.27 (d, J = 1.3 Hz, 1H), 5.81 (d, J = 1.2 Hz, 1H), 1.52 (s, 9H).

tert-butyl 2-(4-methoxyphenyl)acrylate (**31-s**)

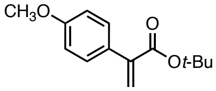

31-s

The ester **31-s** was prepared according to the procedure reported by Tan and co-workers.[7]
^{1}H NMR (500 MHz, $CDCl_3$): δ 7.36 (d, J = 8.8 Hz, 2H), 6.87 (d, J = 8.9 Hz, 2H), 6.15 (d, J = 1.4 Hz, 1H), 5.75 (d, J = 1.4 Hz, 1H), 3.81 (s, 3H), 1.53 (s, 9H).

tert-butyl 2-(4-((*tert*-butoxycarbonyl)amino)phenyl)acrylate (**32-s**)

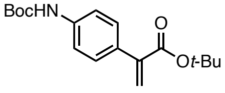

32-s

The ester **32-s** was prepared according to General Procedure A in 12% overall yield.
^{1}H NMR (500 MHz, $CDCl_3$): δ 7.39 – 7.30 (m, 4H), 6.17 (d, J = 1.4 Hz, 1H), 5.76 (d, J = 1.4 Hz, 1H), 1.54 – 1.50 (m, 18H). ^{13}C NMR (126 MHz, $CDCl_3$): δ 166.2, 152.6, 142.2, 138.1, 131.8, 129.0, 124.5, 117.8, 81.2, 80.6, 28.3, 28.1. IR (FTIR), cm^{-1}: 3399, 2984, 2935, 1705, 1524, 1504, 1368, 1226, 1155, 834, 762. HRMS (ESI-TOF), m/z: [M + Na]$^+$ calculated for $[C_{18}H_{25}NNaO_4]^+$, 342.1676; found 347.1678.

methyl 2-phenylacrylate (**33-s**)

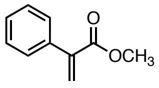

33-s

The ester **33-s** was prepared according to the procedure reported by Shu and co-workers.[9]
^{1}H NMR (300 MHz, $CDCl_3$): δ 7.43 – 7.39 (m, 2H), 7.38 – 7.32 (m, 3H), 6.37 (d, *J* = 1.2 Hz, 1H), 5.90 (d, *J* = 1.2 Hz, 1H), 3.83 (s, 3H).

ethyl 2-phenylacrylate (**34-s**)

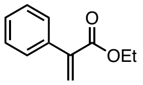

34-s

The ester **34-s** was prepared according to the procedure reported by Chang and co-workers.[10]
^{1}H NMR (300 MHz, $CDCl_3$): δ 7.48 – 7.27 (m, 5H), 6.34 (d, *J* = 1.3 Hz, 1H), 5.88 (d, *J* = 1.3 Hz, 1H), 4.29 (q, *J* = 7.1 Hz, 2H), 1.33 (t, *J* = 7.1 Hz, 3H).

benzyl 2-phenylacrylate (**35-s**)

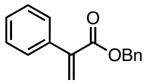

35-s

The ester **35-s** was prepared according to the procedure reported by Bower and co-workers.[11]
^{1}H NMR (300 MHz, $CDCl_3$): δ 7.48 – 7.33 (m, 10H), 6.40 (d, *J* = 1.2 Hz, 1H), 5.93 (d, *J* = 1.2 Hz, 1H), 5.28 (s, 2H).

tert-pentyl 2-phenylacrylate (**36-s**)

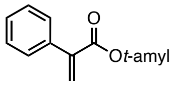

36-s

The ester **36-s** was prepared according to procedures reported by Yang, Zhou, and co-workers[12] using 2-phenylacryloyl chloride as starting material.
^{1}H NMR (300 MHz, $CDCl_3$): δ 7.42 – 7.29 (m, 5H), 6.25 (d, *J* = 1.4 Hz, 1H), 5.81 (d, *J* = 1.4 Hz, 1H), 1.84 (q, *J* = 7.5 Hz, 2H), 1.51 (s, 6H), 0.91 (t, *J* = 7.5 Hz, 3H). ^{13}C NMR (126 MHz, $CDCl_3$): δ 166.0, 142.9, 137.2, 128.3, 127.9, 127.9, 125.5, 83.7, 33.7, 25.5, 8.2. IR (FTIR), cm^{-1}: 2978, 2942, 1713, 1202, 1153, 1090, 773, 698.
*Note: Due to poor ionization, the calculated mass was not observed for **36-s**. The calculated mass for the corresponding product **36** was observed and documented.*

tert-butyl 2-(pyridin-3-yl)acrylate (**40-s**)

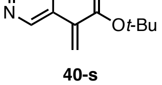

40-s

The ester **40-s** was prepared according to General Procedure A in 51% overall yield.
^{1}H NMR (500 MHz, $CDCl_3$): δ 8.63 (dd, *J* = 2.3, 0.9 Hz, 1H), 8.55 (dd, *J* = 4.8, 1.7 Hz, 1H), 7.77 (dt, *J* = 7.9, 2.0 Hz, 1H), 7.28 (ddd, *J* = 7.9, 4.9, 0.9 Hz, 1H), 6.39 (d, *J* = 1.1 Hz, 1H), 5.89 (d, *J* = 1.3 Hz, 1H), 1.53 (s, 9H). ^{13}C NMR (126 MHz, $CDCl_3$): δ 165.1, 149.0, 148.9, 139.8, 136.1,

133.1, 127.6, 122.8, 81.9, 28.1. IR (FTIR), cm^{-1}: 2981, 2935, 1720, 1370, 1220, 1149, 984, 844, 712. HRMS (ESI-TOF), m/z: $[M + H]^+$ calculated for $[C_{12}H_{16}NO_2]^+$, 206.1176; found 206.1175.

tert-butyl 2-(6-methylpyridin-3-yl)acrylate (**41-s**)

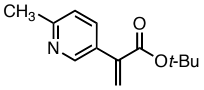

41-s

The ester **41-s** was prepared according to General Procedure A in 69% overall yield.
^{1}H NMR (500 MHz, $CDCl_3$): δ 8.50 (d, *J* = 2.5 Hz, 1H), 7.65 (dd, *J* = 8.1, 2.3 Hz, 1H), 7.13 (d, *J* = 8.0 Hz, 1H), 6.33 (d, *J* = 1.2 Hz, 1H), 5.85 (d, *J* = 1.2 Hz, 1H), 2.56 (s, 3H), 1.52 (s, 9H). ^{13}C NMR (126 MHz, $CDCl_3$): δ 165.3, 157.8, 148.3, 139.8, 136.3, 130.1, 126.7, 122.3, 81.7, 28.0, 24.1. IR (FTIR), cm^{-1}: 2980, 2932, 1713, 1615, 1491, 1370, 1159, 1094, 1027, 740. HRMS (ESI-TOF), m/z: $[M + H]^+$ calculated for $[C_{13}H_{18}NO_2]^+$, 220.1332; found 220.1332.

tert-butyl 2-(6-methoxypyridin-3-yl)acrylate (**42-s**)

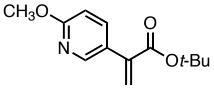

42-s

The ester **42-s** was prepared according to General Procedure A in 59% overall yield.
^{1}H NMR (400 MHz, $CDCl_3$): δ 8.19 (dd, *J* = 2.5, 0.8 Hz, 1H), 7.66 (dd, *J* = 8.6, 2.5 Hz, 1H), 6.72 (dd, *J* = 8.6, 0.8 Hz, 1H), 6.25 (d, *J* = 1.3 Hz, 1H), 5.80 (d, *J* = 1.3 Hz, 1H), 3.95 (s, 3H), 1.53 (s, 9H). ^{13}C NMR (101 MHz, $CDCl_3$): δ 165.6, 163.8, 146.1, 139.5, 138.9, 126.4, 125.5, 109.9, 81.6, 53.5, 28.1. IR (FTIR), cm^{-1}: 2980, 2948, 1716, 1603, 1495, 1370, 1286, 1160, 1025, 833. HRMS (ESI-TOF), m/z: $[M + H]^+$ calculated for $[C_{13}H_{18}NO_3]^+$, 236.1281; found 236.1287.

tert-butyl 2-(6-chloropyridin-3-yl)acrylate (**43-s**)

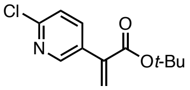

43-s

The ester **43-s** was prepared according to General Procedure A in 23% overall yield.
^{1}H NMR (500 MHz, $CDCl_3$): δ 8.40 (dd, *J* = 2.5, 0.7 Hz, 1H), 7.74 (dd, *J* = 8.3, 2.5 Hz, 1H), 7.31 (dd, *J* = 8.4, 0.7 Hz, 1H), 6.41 (d, *J* = 1.0 Hz, 1H), 5.89 (d, *J* = 1.0 Hz, 1H), 1.53 (s, 9H). ^{13}C NMR (126 MHz, $CDCl_3$): δ 164.7, 150.8, 149.0, 138.8, 138.6, 131.9, 128.0, 123.3, 82.1, 28.0. IR (FTIR), cm^{-1}: 2980, 2935, 1713, 1461, 1370, 1161, 1111, 852, 836, 811. HRMS (ESI-TOF), m/z: $[M + H]^+$ calculated for $[C_{12}H_{15}ClNO_2]^+$, 240.0786; found 240.0790.

tert-butyl 2-(pyrimidin-5-yl)acrylate (**44-s**)

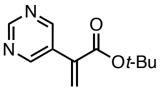

44-s

The ester **44-s** was prepared according to General Procedure A in 21% overall yield.
^{1}H NMR (500 MHz, $CDCl_3$): δ 9.16 (s, 1H), 8.80 (s, 2H), 6.50 (d, *J* = 1.0 Hz, 1H), 5.96 (d, *J* = 1.0 Hz, 1H), 1.54 (s, 9H). ^{13}C NMR (126 MHz, $CDCl_3$): δ 164.1, 157.7, 156.1, 136.9, 131.1, 129.2, 82.5, 28.0. IR (FTIR), cm^{-1}: 2982, 2935, 1716, 1552, 1422, 1317, 1234, 1165, 1133. HRMS (ESI-TOF), m/z: $[M + H]^+$ calculated for $[C_{11}H_{15}N_2O_2]^+$, 207.1128; found 207.1127.

tert-butyl 2-(naphthalen-2-yl)acrylate (**45-s**)

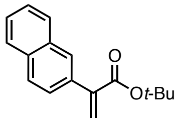

45-s

The ester **45-s** was prepared according to General Procedure A in 69% overall yield.
^{1}H NMR (300 MHz, $CDCl_3$): δ 7.93 – 7.76 (m, 4H), 7.58 – 7.42 (m, 3H), 6.33 (d, *J* = 1.3 Hz, 1H), 5.94 (d, *J* = 1.4 Hz, 1H), 1.56 (s, 9H). ^{13}C NMR (126 MHz, $CDCl_3$): δ 166.1, 142.9, 134.6, 133.1, 132.9, 128.2, 127.5, 127.4, 127.3, 126.3, 126.2, 126.1, 125.9, 81.4, 28.1. IR (FTIR), cm^{-1}: 2978, 2932, 1720, 1367, 1247, 1155, 851, 818, 749. HRMS (ESI-TOF), m/z: $[M + Na]^+$ calculated for $[C_{17}H_{18}NaO_2]^+$, 277.1199; found 277.1196.

tert-butyl 2-(quinolin-3-yl)acrylate (**46-s**)

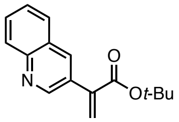

46-s

The ester **46-s** was prepared according to General Procedure B in 21% overall yield.
^{1}H NMR (500 MHz, $CDCl_3$): δ 8.94 (d, *J* = 2.2 Hz, 1H), 8.23 (s, 1H), 8.10 (d, *J* = 8.6 Hz, 1H), 7.83 (d, *J* = 8.3 Hz, 1H), 7.71 (t, *J* = 7.5 Hz, 1H), 7.55 (d, *J* = 7.5 Hz, 1H), 6.48 (d, *J* = 1.1 Hz, 1H), 6.01 (d, *J* = 1.2 Hz, 1H), 1.55 (s, 9H). ^{13}C NMR (126 MHz, $CDCl_3$): δ 165.2, 150.4, 147.4, 140.0, 135.2, 130.2, 129.7, 129.2, 128.1, 127.9, 127.5, 126.9, 82.0, 28.1. IR (FTIR), cm^{-1}: 2980, 2935, 1718, 1493, 1370, 1258, 1156, 851, 755. HRMS (ESI-TOF), m/z: $[M + H]^+$ calculated for $[C_{16}H_{18}NO_2]^+$, 256.1332; found 256.1335.

tert-butyl 2-(quinolin-4-yl)acrylate (**47-s**)

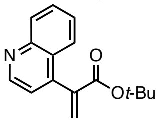

47-s

The ester **47-s** was prepared according to General Procedure B in 27% overall yield.
^{1}H NMR (500 MHz, $CDCl_3$): δ 8.90 (d, *J* = 4.4 Hz, 1H), 8.15 (d, *J* = 8.5 Hz, 1H), 7.79 (d, *J* = 8.4 Hz, 1H), 7.76 – 7.70 (m, 1H), 7.56 – 7.52 (m, 1H), 7.28 (d, *J* = 4.3 Hz, 1H), 6.69 (d, *J* = 1.4 Hz, 1H), 5.88 (d, *J* = 1.5 Hz, 1H), 1.40 (s, 9H). ^{13}C NMR (126 MHz, $CDCl_3$): δ 164.8, 149.6, 147.8, 144.8, 140.3, 136.0, 129.8, 129.5, 129.5, 126.6, 125.3, 121.3, 81.9, 27.8. IR (FTIR), cm^{-1}: 2980, 2935, 1716, 1367, 1236, 1159, 1144, 1127, 767. HRMS (ESI-TOF), m/z: $[M + H]^+$ calculated for $[C_{16}H_{18}NO_2]^+$, 256.1332; found 256.1336.

tert-butyl 2-(quinolin-5-yl)acrylate (**48-s**)

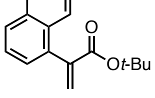

48-s

The ester **48-s** was prepared according to General Procedure B in 17% overall yield.

^{1}H NMR (500 MHz, $CDCl_3$): δ 8.89 (dd, *J* = 4.2, 1.7 Hz, 1H), 8.12 – 8.04 (m, 2H), 7.66 (dd, *J* = 8.6, 7.1 Hz, 1H), 7.40 (dd, *J* = 7.1, 1.2 Hz, 1H), 7.36 (dd, *J* = 8.5, 4.2 Hz, 1H), 6.62 (d, *J* = 1.7 Hz, 1H), 5.82 (d, *J* = 1.7 Hz, 1H), 1.39 (s, 9H). ^{13}C NMR (126 MHz, $CDCl_3$): δ 165.6, 150.1, 148.0, 141.3, 136.0, 133.8, 129.6, 129.3, 128.7, 127.2, 126.9, 120.8, 81.4, 27.8. IR (FTIR), cm^{-1}: 2980, 2933, 1715, 1500, 1370, 1236, 1153, 1118, 803. HRMS (ESI-TOF), m/z: $[M + H]^+$ calculated for $[C_{16}H_{18}NO_2]^+$, 256.1332; found 256.1335.

tert-butyl 2-(1-methyl-1*H*-indol-6-yl)acrylate (**49-s**)

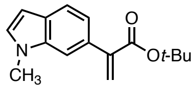

49-s

The ester **49-s** was prepared according to General Procedure B in 14% overall yield.
^{1}H NMR (500 MHz, $CDCl_3$): δ 7.57 (d, *J* = 8.3 Hz, 1H), 7.43 (s, 1H), 7.16 (dd, *J* = 8.2, 1.5 Hz, 1H), 7.06 (d, *J* = 3.0 Hz, 1H), 6.46 (dd, *J* = 3.0, 0.9 Hz, 1H), 6.21 (d, *J* = 1.5 Hz, 1H), 5.85 (d, *J* = 1.5 Hz, 1H), 3.80 (s, 3H), 1.55 (s, 9H). ^{13}C NMR (126 MHz, $CDCl_3$): δ 166.8, 143.8, 130.6, 129.6, 126.0, 124.2, 120.7, 120.2, 120.0, 109.2, 100.8, 81.1, 32.8, 28.2. IR (FTIR), cm^{-1}: 2980, 2935, 1728, 1513, 1474, 1370, 1247, 1142, 717. HRMS (ESI-TOF), m/z: $[M + H]^+$ calculated for $[C_{16}H_{20}NO_2]^+$, 258.1489; found 258.1487.

tert-butyl 2-(1-methyl-1*H*-indazol-6-yl)acrylate (**50-s**)

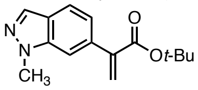

50-s

The ester **50-s** was prepared according to General Procedure B in 33% overall yield.
^{1}H NMR (500 MHz, $CDCl_3$): δ 7.96 (s, 1H), 7.68 (d, *J* = 8.4 Hz, 1H), 7.48 (s, 1H), 7.18 (dd, *J* = 8.4, 1.3 Hz, 1H), 6.33 (d, *J* = 1.3 Hz, 1H), 5.91 (d, *J* = 1.3 Hz, 1H), 4.08 (s, 3H), 1.55 (s, 9H). ^{13}C NMR (126 MHz, $CDCl_3$): δ 166.0, 143.1, 139.8, 135.4, 132.5, 126.3, 123.5, 121.5, 120.4, 108.7, 81.5, 35.5, 28.1. IR (FTIR), cm^{-1}: 2980, 2935, 1713, 1477, 1370, 1157, 945, 851. HRMS (ESI-TOF), m/z: $[M + H]^+$ calculated for $[C_{15}H_{19}N_2O_2]^+$, 259.1441; found 259.1444.

tert-butyl 2-(3-methyl-2-oxo-2,3-dihydrobenzo[*d*]oxazol-6-yl)acrylate (**51-s**)

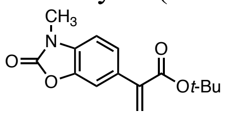

51-s

The ester **51-s** was prepared according to General Procedure B in 20% overall yield.
^{1}H NMR (500 MHz, $CDCl_3$): δ 7.30 (d, *J* = 1.6 Hz, 1H), 7.28 – 7.23 (m, 1H), 6.92 (d, *J* = 8.1 Hz, 1H), 6.26 (d, *J* = 1.3 Hz, 1H), 5.81 (d, *J* = 1.2 Hz, 1H), 3.41 (s, 3H), 1.53 (s, 9H). ^{13}C NMR: (126 MHz, $CDCl_3$): δ 165.7, 154.9, 142.3, 142.0, 132.1, 131.5, 125.8, 124.2, 110.2, 107.3, 81.6, 28.2, 28.1. IR (FTIR), cm^{-1}: 2980, 2937, 1782, 1713, 1508, 1370, 1154, 1161, 938, 751. HRMS (ESI-TOF), m/z: $[M + H]^+$ calculated for $[C_{15}H_{18}NO_4]^+$, 276.1230; found 276.1229.

tert-butyl 2-(1,3,3-trimethyl-2-oxoindolin-6-yl)acrylate (**52-s**)

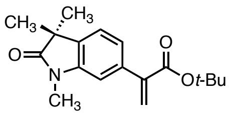

52-s

The ester **52-s** was prepared according to General Procedure B in 40% overall yield.
^{1}H NMR (500 MHz, $CDCl_3$): δ 7.17 (d, J = 7.5 Hz, 1H), 7.09 (dd, J = 7.6, 1.5 Hz, 1H), 6.92 (d, J = 1.5 Hz, 1H), 6.27 (d, J = 1.3 Hz, 1H), 5.85 (d, J = 1.4 Hz, 1H), 3.22 (s, 3H), 1.55 (s, 9H), 1.37 (s, 6H). ^{13}C NMR (126 MHz, $CDCl_3$): δ 181.4, 165.9, 142.7, 142.4, 136.9, 135.6, 125.9, 122.8, 121.8, 108.2, 81.5, 44.1, 28.1, 26.2, 24.3. IR (FTIR), cm^{-1}: 2975, 2933, 1715, 1621, 1450, 1370, 1160, 954. HRMS (ESI-TOF), m/z: $[M + H]^+$ calculated for $[C_{18}H_{24}NO_3]^+$, 302.1751; found 302.1758.

4.2 Synthetic procedures for the product standards 20 and S11.

*Synthesis of tert-butyl 5-amino-5-oxo-2-phenylpentanoate (**20**).*

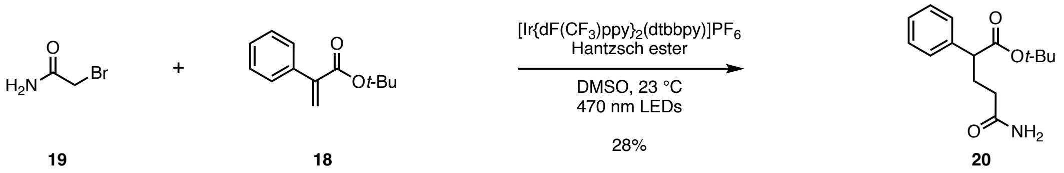

The ester **18** (81.7 mg, 400 µmol, 2.00 equiv), 2-bromoacetamide (**19**, 27.6 mg, 200 µmol, 1 equiv), Hantzsch ester (76.0 mg, 300 µmol, 1.50 equiv), and [Ir{dF(CF_3)ppy}$_2$(dtbbpy)]PF_6 (2.24 mg, 2.00 µmol, 1 mol%) were combined in a 4-mL glass shell vial. The vial was sealed with a rubber septum, evacuated under reduced pressure, and backfilled with nitrogen. This process was repeated three times. Dimethyl sulfoxide (DMSO, 1.50 mL, degassed by sparging with nitrogen) was added to the reaction mixture. The reaction vessel was then stirred under irradiation with 470 nm LEDs for 18 h. The product mixture was diluted sequentially with ethyl acetate (2 mL) and saturated aqueous sodium bicarbonate solution (2 mL). The diluted product mixture was transferred to a separatory funnel, and the layers that formed were separated. The aqueous layer was extracted with ethyl acetate (3 × 10 mL). The organic layers were combined, and the combined organic layers were dried over sodium sulfate. The dried solution was filtered, and the filtrate was concentrated. The residue obtained was purified by flash-column chromatography (eluting with 50% ethyl acetate–hexanes) to provide the amide **20** as a light-yellow powder (14.0 mg, 28%).

The NMR spectroscopic data obtained for amide **20** matched those of the amide **20** prepared via enzymatic reactions (see Section 5.1).

Standard Curve of the amide 20 for LC-MS analysis: All sample vials contain the corresponding amount of **20** and 100 µL of a 5 mg/mL solution of TBB, diluted with acetonitrile to a final volume of 2.20 mL. The x-axis measurement is the amount of **20** in milligrams. The y-axis measurement is the ratio of the LC-MS peak area for **20** to that of the standard.

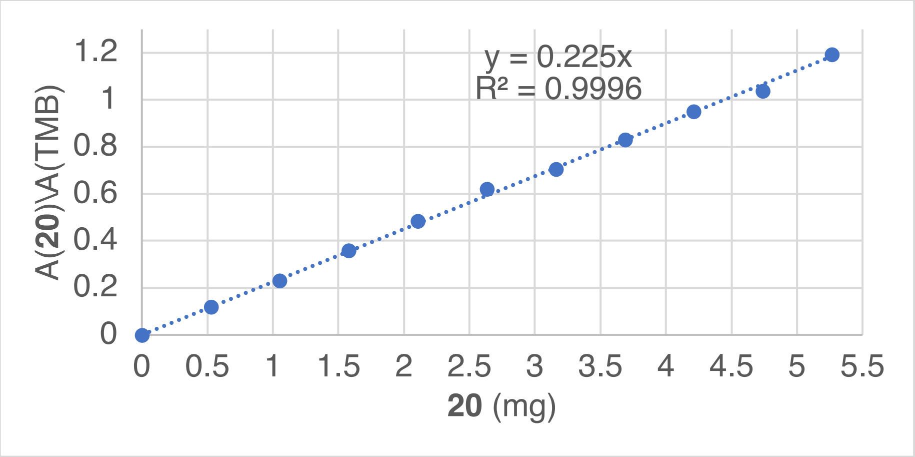

*Synthesis of tert-butyl 2-phenylpropanoate (**S11**).*

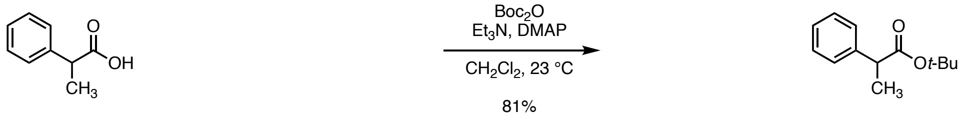

S10 **S11**

Triethylamine (1.05 mL, 7.50 mmol, 1.50 equiv) and 4-dimethylaminopyridine (DMAP, 61.1 mg, 500 µmol, 0.10 equiv) were added in sequence to a solution of 2-phenylpropionic acid (**S10**, 683 µL, 5.00 mmol, 1 equiv) in dichloromethane (17.0 mL) at 23 °C. Di-*tert*-butyl dicarbonate (Boc_2O, 1.26 mL, 5.50 mmol, 1.10 equiv) was added dropwise to the reaction mixture at 23 °C over 2 min. The reaction mixture was stirred for 3 h at 23 °C. The product mixture was concentrated under reduced pressure. The residue obtained was purified by flash-column chromatography (eluting with 2% ethyl acetate–hexanes) to provide the ester **S11** as a colorless oil (840 mg, 81%).

^{1}H NMR (500 MHz, $CDCl_3$) δ 7.35 – 7.20 (m, 5H), 3.61 (q, J = 7.2 Hz, 1H), 1.45 (d, J = 7.2 Hz, 3H), 1.39 (s, 9H).

The ^{1}H NMR shifts of **S11** so obtained matched the values reported by Hama and Hartwig.[13]

4.3 Synthetic procedure for phenyl glutarimide (5) from the amide 20.

Synthesis of 3-phenylpiperidine-2,6-dione (**5**).

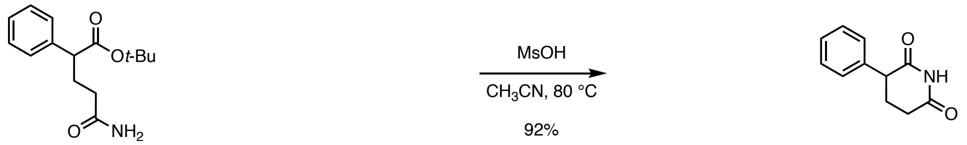

Methanesulfonic acid (32.5 µL, 500 µmol, 5.00 equiv) was added to a solution of the amide **20** (26.3 mg, 100 µmol, 1 equiv) in acetonitrile (300 µL) at 23 °C. The reaction mixture was stirred for 3 h at 80 °C. The product mixture was cooled to 23 °C over 10 min, and the cooled mixture was diluted sequentially with ethyl acetate (2 mL) and saturated aqueous sodium bicarbonate solution (2 mL). The diluted product mixture was transferred to a separatory funnel, and the layers that formed were separated. The aqueous layer was extracted with ethyl acetate (3 × 10 mL). The organic layers were combined, and the combined organic layers were dried over sodium sulfate. The dried solution was filtered, and the filtrate was concentrated. The residue obtained was purified by flash-column chromatography (eluting with 50% ethyl acetate–hexanes) to provide the glutarimide **5** as a white powder (17.5 mg, 92%).

^{1}H NMR (500 MHz, $CDCl_3$): δ 7.38 (t, *J* = 7.2 Hz, 2H), 7.33 (t, *J* = 7.3 Hz, 1H), 7.22 (d, *J* = 6.8 Hz, 2H), 3.80 (dd, *J* = 9.5, 5.2 Hz, 1H), 2.77 – 2.70 (m, 1H), 2.65 (ddd, *J* = 17.6, 9.6, 5.2 Hz, 1H), 2.41 – 2.21 (m, 2H). ^{13}C NMR (126 MHz, $CDCl_3$): δ 173.0, 172.1, 137.0, 129.0, 128.0, 127.8, 47.9, 30.8, 26.4. IR (FTIR), cm^{-1}: 3064, 2967, 2881, 1713, 1698, 1456, 1361, 1331, 1303, 1291, 1234, 1195, 844, 752, 695, 669. HRMS (ESI-TOF), m/z: $[M + H]^+$ calculated for $[C_{11}H_{12}NO_2]^+$, 190.0863; found 190.0861. Chiral HPLC method: CHIRALPAK® IH column, 20% *iso*-propanol, 80% hexane; t_R1 = 26.45 min, t_R2 = 34.74 min

The NMR spectroscopic data obtained for the glutarimide **5** matched those reported by Reisman and co-workers.[14]

5. Photoenzymatic reaction procedures.

5.1 Procedures for the synthesis of 20–52 via analytical-scale photoenzymatic hydroalkylation reactions.

The products **20–52** were prepared according to Photoenzymatic Hydroalkylation Procedures A and/or B. ^{1}H NMR, ^{13}C NMR, HRMS, IR, and chiral HPLC data were collected from samples isolated from the photoenzymatic reactions.

Photoenzymatic Hydroalkylation Procedure A.

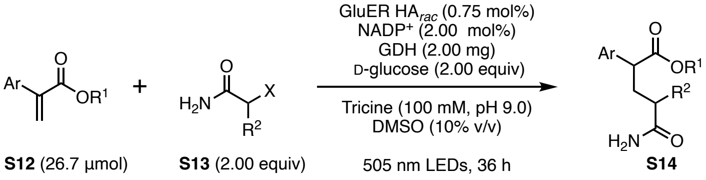

All reactions were run with 26.7 µmol of the acrylate substrate **S12** and 200 nmol of GluER HA$_{rac}$ (GluER-T36A-Y343W), unless otherwise noted. Reactions for all substrates were performed in triplicate. In the glovebox, 767 µL of Tricine buffer (100 mM, pH 9.0) was added to a 1.5-dram shell vial equipped with a magnetic stir bar containing 0.75 mol% of the aliquoted GluER HA$_{rac}$ (200 nmol) to provide an orange solution. 133 µL of a "master mix" solution containing 2.00 equivalents of D-glucose, 2.00 mg GDH, and 2.00 mol% of NADP$^+$ in Tricine buffer (100 mM, pH 9.0) was added to the reaction mixture. The mixture was allowed to sit for 2 min with gentle mixing until the orange color disappeared. Once the solution became colorless, 2.00 equiv of **S13** was added as a 50.0 µL solution in dimethyl sulfoxide. Finally, 26.7 µmol of **S12** was added as a 50.0 µL solution in dimethyl sulfoxide. The vial was sealed with a rubber septum and removed from the glovebox. The sealed vial was placed on a stir plate at 300 rpm under a fan, and irradiated with a Lumidox II 96-well 505 nm cyan LED array (75 mW per well) for 36 h.

For LC-MS determination of yields: After 36 h, 1.90 mL of acetonitrile was added to the product mixture, followed by the addition of 133 µL of 5 mg/mL TBB solution. The product mixture was stirred for 30 min at 500 rpm to denature the enzyme. The product mixture was then transferred to microcentrifuge tubes. The tubes were centrifuged at 16000 × *g* for 10 min. The supernatant was collected and analyzed by LC-MS. The products were then quantified against a standard calibration curve to calculate an effective yield. For NMR determination of reaction yields: After 36 h, 26.7 µmol of 1,3,5-trimethoxybenzene (TMB) was added as a 1 mL solution in ethyl acetate to the product mixture, followed by the addition of an additional 1 mL of ethyl acetate. The product mixture was stirred for 30 min at 500 rpm to denature the enzyme. The product mixture was then transferred to microcentrifuge tubes. The tubes were centrifuged at 8000 × *g* for 1 min to separate the aqueous and organic layers. The organic layer was collected. An equal volume of ethyl acetate was then added to the microcentrifuge tube containing the aqueous layer. The tube was agitated to mix the layers. The tube was then centrifuged at 8000 × *g* for 1 min to separate the aqueous and organic layers. The organic layer was collected. This procedure was repeated five times. The organic layers were combined and concentrated under reduced pressure. The concentrated product mixture was resuspended in $CDCl_3$ and analyzed by ^{1}H NMR for yield calculation, where the

signal at δ = 6.06 ppm was assigned an integration of 3.00. The integration of varying signals from different products can be used to calculate an effective yield.

For normal-phase HPLC analysis to determine the enantiomeric ratio: the concentrated, unpurified product mixture was dissolved in a 1:9 mixture of *iso*-propanol–hexane and analyzed under the corresponding conditions. For reverse-phase HPLC analysis to determine the enantiomeric ratio: the concentrated unpurified product mixture was dissolved in acetonitrile and analyzed with the corresponding conditions.

The product mixtures for the triplicates were combined after analysis, and the product was purified using preparative thin-layer chromatography. The purified products were characterized using NMR, HRMS, and IR spectroscopy.

Photoenzymatic Hydroalkylation Procedure B.

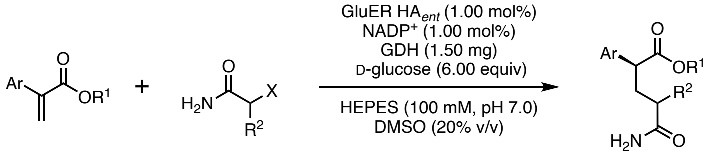

S12 (20.0 µmol) **S13** (4.00 equiv) **S14**

All reactions were run with 20.0 µmol of the acrylate substrate **S12** and 200 nmol of GluER HA$_{ent}$ (GluER-T36A-Y177F-M102G-M105V-A44E-W66M), unless otherwise noted. Reactions for all substrates were performed in triplicate. 700 µL of HEPES buffer (100 mM, pH 7.0) was added to a 1.5-dram shell vial equipped with a magnetic stir bar containing 1.00 mol% of the aliquoted GluER HA$_{ent}$ (GluER-T36A-Y177F-M102G-M105V-A44E-W66M, 200 nmol) to provide an orange solution. 100 µL of a "master mix" solution containing 6.00 equivalents of D-glucose, 1.50 mg GDH, and 1.00 mol% of NADP$^+$ in HEPES (100 mM, pH 7.0) was added to the reaction mixture. The mixture was allowed to sit for 2 min with gentle mixing until the orange color disappeared. Once the solution became colorless, 4.00 equiv of **S13** was added as a 100 µL solution in dimethyl sulfoxide. Finally, 20.0 µmol of **S12** was added as a 100 µL solution in dimethyl sulfoxide. The vial was sealed with a rubber septum and removed from the glovebox, then placed on a stir plate at 300 rpm under a fan and irradiated with a Lumidox II 96-well 505 nm cyan LED array (85 mW per well) for 48 h.

For LC-MS determination of yields: After 48 h, 1.90 mL of acetonitrile was added to the product mixture, followed by the addition of 100 µL of 5 mg/mL TBB solution. The product mixture was stirred for 30 min at 500 rpm to denature the enzyme. The product mixture was then transferred to microcentrifuge tubes. The tubes were centrifuged at 16000 × *g* for 10 min. The supernatant was collected and analyzed by LC-MS. The products were then quantified against a standard calibration curve to calculate an effective yield. For NMR determination of reaction yields: After 48 h, 20.0 µmol of TMB was added as a 1 mL solution in ethyl acetate to the product mixture, followed by the addition of an additional 1 mL of ethyl acetate. The product mixture was stirred for 30 min at 500 rpm to denature the enzyme. The product mixture was then transferred to microcentrifuge tubes. The tubes were centrifuged at 8000 × *g* for 1 min to separate the aqueous and organic layers. The organic layer was collected. An equal volume of ethyl acetate was then added to the microcentrifuge tube containing the aqueous layer. The tube was agitated to mix the layers. The tube was then centrifuged at 8000 × *g* for 1 min to separate the aqueous and organic layers. The organic layer was collected. This procedure was repeated five times. The organic layers were combined and concentrated under reduced pressure. The concentrated product mixture was resuspended in $CDCl_3$ and analyzed by ^1H NMR for yield calculation, where the signal at δ = 6.06 ppm was assigned an integration of 3.00. The integration of varying signals from different products can be used to calculate an effective yield.

For normal-phase HPLC analysis to determine the enantiomeric ratio: the concentrated, unpurified product mixture was dissolved in a 1:9 mixture of *iso*-propanol–hexane and analyzed under the corresponding conditions. For reverse-phase HPLC analysis to determine the enantiomeric ratio: the concentrated unpurified product mixture was dissolved in acetonitrile and analyzed with the corresponding conditions.

*Synthesis of the products **20–52** via **<u>Procedure A.</u>***

Yields for **20** were determined by LC-MS. Yields for **21–52** were determined by NMR. 2-Bromoacetamide (**19**) was used to synthesize **20–36** and **40–52**. The amides used for the synthesis of **37–39** are specified below. Syntheses of the corresponding substrates **18** and **21-s–52-s** are reported in the previous section.

tert-butyl 5-amino-5-oxo-2-phenylpentanoate (**20**):

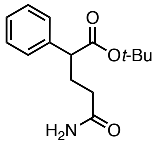

20

Yields: 94%, 88%, 91%. Enantiomeric ratio: 50:50.
^{1}H NMR (500 MHz, $CDCl_3$): δ 7.35 – 7.16 (m, 5H), 3.49 (t, *J* = 7.7 Hz, 1H), 2.36 – 2.23 (m, 1H), 2.13 (dd, *J* = 7.9, 6.9 Hz, 2H), 2.10 – 1.95 (m, 1H), 1.36 (s, 9H). ^{13}C NMR (126 MHz, $CDCl_3$): δ 174.6, 172.7, 138.8, 128.6, 127.8, 127.2, 80.9, 51.7, 33.3, 28.8, 27.9. IR (FTIR), cm^{-1}: 3394, 3181, 1717, 1649, 1364, 1332, 1152, 697. HRMS (ESI-TOF), m/z: $[M + Na]^+$ calculated for $[C_{15}H_{21}NNaO_3]^+$, 286.1414; found 286.1410. Chiral HPLC method: CHIRALCEL® OX-3R column, 55% acetonitrile, 45% water; t_R1 = 3.54 min, t_R2 = 3.89 min.

tert-butyl 5-amino-5-oxo-2-(*o*-tolyl)pentanoate (**21**):

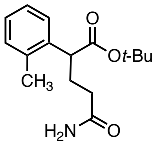

21

Yields: 72%, 73%, 68%. Enantiomeric ratio: 50:50.
^{1}H NMR (500 MHz, $CDCl_3$): δ 7.30 – 7.23 (m, 1H), 7.20 – 7.10 (m, 3H), 3.83 (t, *J* = 7.6 Hz, 1H), 2.37 (s, 3H), 2.36 – 2.30 (m, 1H), 2.20 (t, *J* = 7.4 Hz, 2H), 2.06 (dq, *J* = 14.6, 7.3 Hz, 1H), 1.37 (s, 9H). ^{13}C NMR (126 MHz, $CDCl_3$): δ 174.5, 173.0, 137.4, 136.3, 130.5, 126.9, 126.4, 126.3, 80.8, 46.8, 33.3, 28.1, 27.9, 19.8. IR (FTIR), cm^{-1}: 3349, 3198, 2978, 1725, 1670, 1370, 1150, 753. HRMS (ESI-TOF), m/z: $[M + Na]^+$ calculated for $[C_{16}H_{23}NNaO_3]^+$, 300.1570; found 300.1569. Chiral HPLC method: CHIRALCEL® OX-3R column, 55% acetonitrile, 45% water; t_R1 = 3.97 min, t_R2 = 4.88 min.

tert-butyl 5-amino-5-oxo-2-(*m*-tolyl)pentanoate (**22**):

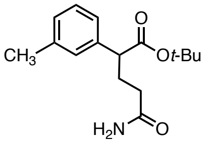

22

Yields: 43%, 41%, 44%. Enantiomeric ratio: 50:50.
^{1}H NMR (500 MHz, $CDCl_3$): δ 7.20 (t, *J* = 7.5 Hz, 1H), 7.10 – 7.04 (m, 3H), 3.47 (t, *J* = 7.7 Hz, 1H), 2.35 – 2.31 (m, 4H), 2.17 (t, *J* = 7.5 Hz, 2H), 2.10 – 2.03 (m, 1H), 1.39 (s, 9H). ^{13}C NMR (126 MHz, $CDCl_3$): δ 174.6, 172.8, 138.7, 138.2, 128.6, 128.4, 128.0, 124.9, 80.9, 51.6, 33.4, 28.9,

27.9, 21.4. IR (FTIR), cm^{-1}: 3351, 3193, 2980, 1726, 1668, 1608, 1370, 1148, 848, 770, 699. HRMS (ESI-TOF), m/z: $[M + Na]^+$ calculated for $[C_{16}H_{23}NNaO_3]^+$, 300.1570; found 300.1572. Chiral HPLC method: CHIRALCEL® OX-3R column, 45% acetonitrile, 55% water; t_R1 = 5.98 min, t_R2 = 6.68 min.

tert-butyl 5-amino-5-oxo-2-(*p*-tolyl)pentanoate (**23**):

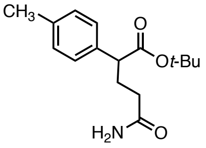

23

Yields: 68%, 69%, 72%. Enantiomeric ratio: 46:54.
^{1}H NMR (500 MHz, $CDCl_3$): δ 7.16 (d, *J* = 8.1 Hz, 2H), 7.12 (d, *J* = 8.1 Hz, 2H), 3.47 (t, *J* = 7.7 Hz, 1H), 2.34 – 2.31 (m, 4H), 2.21 – 2.09 (m, 2H), 2.05 (ddd, *J* = 14.9, 7.6, 6.2 Hz, 1H), 1.39 (s, 9H). ^{13}C NMR (126 MHz, $CDCl_3$): δ 174.4, 172.9, 136.8, 135.7, 129.3, 127.7, 80.8, 51.3, 33.4, 28.8, 27.9, 21.0. IR (FTIR), cm^{-1}: 3336, 3196, 2978, 2928, 1726, 1668, 1515, 1370, 1148, 822. HRMS (ESI-TOF), m/z: $[M + Na]^+$ calculated for $[C_{16}H_{23}NNaO_3]^+$, 300.1570; found 300.1572. Chiral HPLC method: CHIRALCEL® OX-3R column, 55% acetonitrile, 45% water; t_R1 = 4.12 min, t_R2 = 4.73 min.

tert-butyl 5-amino-2-(2-methoxyphenyl)-5-oxopentanoate (**24**):

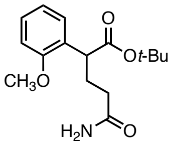

24

Yields: 68%, 62%, 63%. Enantiomeric ratio: 51:49.
^{1}H NMR (500 MHz, $CDCl_3$): δ 7.26 – 7.19 (m, 2H), 6.92 (td, *J* = 7.5, 1.1 Hz, 1H), 6.86 (d, *J* = 8.1 Hz, 1H), 3.92 (t, *J* = 7.5 Hz, 1H), 3.81 (s, 3H), 2.36 – 2.23 (m, 1H), 2.22 – 2.15 (m, 2H), 2.09 – 1.98 (m, 1H), 1.39 (s, 9H). ^{13}C NMR (126 MHz, $CDCl_3$) δ 174.8, 173.1, 156.8, 128.3, 128.1, 127.8, 120.7, 110.7, 80.6, 55.4, 44.8, 33.7, 28.0, 27.8. IR (FTIR), cm^{-1}: 3350, 3192, 2976, 2925, 1724, 1664, 1494, 1368, 1247, 1150, 1031, 755. HRMS (ESI-TOF), m/z: $[M + Na]^+$ calculated for $[C_{16}H_{23}NNaO_4]^+$, 316.1519; found 316.1519. Chiral HPLC method: CHIRALCEL® OX-3R column, 55% acetonitrile, 45% water; t_R1 = 3.67 min, t_R2 = 4.37 min.

tert-butyl 5-amino-2-(2-chlorophenyl)-5-oxopentanoate (**25**):

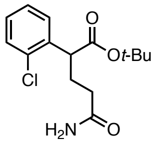

25

Yields: 44%, 42%, 46%. Enantiomeric ratio: 49:51.
^{1}H NMR (500 MHz, $CDCl_3$): δ 7.36 (ddd, *J* = 14.4, 7.8, 1.6 Hz, 2H), 7.24 (td, *J* = 7.5, 1.5 Hz, 1H), 7.19 (td, *J* = 7.6, 1.8 Hz, 1H), 4.09 (t, *J* = 7.6 Hz, 1H), 2.42 – 2.31 (m, 1H), 2.29 – 2.15 (m, 2H), 2.14 – 2.05 (m, 1H), 1.40 (s, 9H). ^{13}C NMR (126 MHz, $CDCl_3$) δ 174.2, 172.0, 136.8, 134.1, 129.7, 128.6, 128.3, 127.1, 81.3, 47.6, 33.3, 28.0, 27.9. IR (FTIR), cm^{-1}: 3324, 3196, 2980, 2933, 1728, 1668, 1476, 1370, 1152, 851, 755. HRMS (ESI-TOF), m/z: $[M + Na]^+$ calculated for

[$C_{15}H_{20}ClNNaO_3$]$^+$, 320.1024; found 320.1027. Chiral HPLC method: CHIRALCEL® OX-3R column, 55% acetonitrile, 45% water; t_R1 = 4.21 min, t_R2 = 5.15 min.

tert-butyl 5-amino-2-(2-bromophenyl)-5-oxopentanoate (**26**):

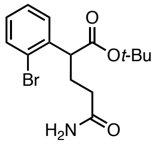

26

Yields: 27%, 30%, 28%. Enantiomeric ratio: 48:52.
^{1}H NMR (500 MHz, $CDCl_3$): δ 7.57 (dd, *J* = 8.1, 1.3 Hz, 1H), 7.34 (dd, *J* = 7.8, 1.8 Hz, 1H), 7.29 (td, *J* = 7.6, 1.3 Hz, 1H), 7.11 (td, *J* = 7.5, 1.7 Hz, 1H), 4.10 (t, *J* = 7.5 Hz, 1H), 2.39 – 2.31 (m, 1H), 2.26 – 2.16 (m, 2H), 2.14 – 2.07 (m, 1H), 1.40 (s, 9H). ^{13}C NMR (126 MHz, $CDCl_3$): δ 174.2, 172.0, 138.5, 133.0, 128.6, 128.5, 127.8, 125.0, 81.3, 50.2, 33.3, 28.3, 27.9. IR (FTIR), cm^{-1}: 3324, 3220, 2978, 2926, 1726, 1666, 1370, 1150, 1023, 751. HRMS (ESI-TOF), m/z: [M + Na]$^+$ calculated for [$C_{15}H_{20}BrNNaO_3$]$^+$, 364.0519; found 364.0519. Chiral HPLC method: CHIRALCEL® OX-3R column, 45% acetonitrile, 55% water; t_R1 = 7.77 min, t_R2 = 9.90 min.

tert-butyl 5-amino-2-(2-iodophenyl)-5-oxopentanoate (**27**):

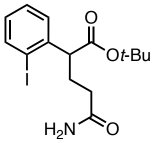

27

Yields: 10%, 13%, 15%. Enantiomeric ratio: 51:49.
^{1}H NMR (500 MHz, $CDCl_3$): δ 7.86 (d, *J* = 8.7 Hz, 1H), 7.36 – 7.28 (m, 2H), 6.99 – 6.90 (m, 1H), 3.98 (t, *J* = 7.5 Hz, 1H), 2.37 – 2.00 (m, 4H), 1.40 (s, 9H). ^{13}C NMR (126 MHz, $CDCl_3$): δ 174.1, 172.0, 141.9, 139.8, 128.9, 128.6, 127.6, 101.9, 81.3, 55.0, 33.3, 28.6, 27.9. IR (FTIR), cm^{-1}: 3198, 2976, 1722, 1666, 1368, 1148, 1012, 751. HRMS (ESI-TOF), m/z: [M + Na]$^+$ calculated for [$C_{15}H_{20}INNaO_3$]$^+$, 412.0380; found 412.0379. Chiral HPLC method: CHIRALCEL® OX-3R column, 45% acetonitrile, 55% water; t_R1 = 9.14 min, t_R2 = 11.60 min.

tert-butyl 5-amino-2-(2-nitrophenyl)-5-oxopentanoate (**28**):

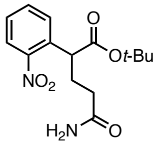

28

Yields: 18%, 17%, 17%. Enantiomeric ratio: 51:49.
^{1}H NMR (500 MHz, $CDCl_3$) δ 7.88 (d, *J* = 9.5 Hz, 1H), 7.59 (t, *J* = 7.5 Hz, 1H), 7.53 (d, *J* = 9.5 Hz, 1H), 7.42 (d, *J* = 7.8 Hz, 1H), 4.12 (t, *J* = 7.3 Hz, 1H), 2.49 (dddd, *J* = 13.8, 9.2, 7.4, 5.7 Hz, 1H), 2.38 – 2.15 (m, 3H), 1.37 (s, 9H). ^{13}C NMR (126 MHz, $CDCl_3$) δ 174.0, 171.1, 149.6, 133.5, 133.0, 129.9, 128.1, 124.7, 81.9, 46.6, 33.5, 28.0, 27.8. IR (FTIR), cm^{-1}: 3367, 3192, 2982, 2924, 2851, 1727, 1694, 1668, 1528, 1355, 1152, 855, 787, 745, 703. HRMS (ESI-TOF), m/z: [M + Na]$^+$ calculated for [$C_{15}H_{20}N_2NaO_5$]$^+$, 331.1264; found 331.1271. Chiral HPLC method: CHIRALCEL® OX-3R column, 45% acetonitrile, 55% water; t_R1 = 5.08 min, t_R2 = 5.73 min.

tert-butyl 5-amino-2-(3-bromophenyl)-5-oxopentanoate (**29**):

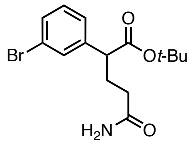

29

Yields: 27%, 27%, 29%. Enantiomeric ratio: 52:48.
^{1}H NMR (500 MHz, $CDCl_3$): δ 7.44 (t, *J* = 1.9 Hz, 1H), 7.39 (dt, *J* = 7.7, 1.6 Hz, 1H), 7.22 – 7.15 (m, 2H), 3.50 (t, *J* = 7.7 Hz, 1H), 2.32 (dt, *J* = 13.3, 7.5 Hz, 1H), 2.17 (td, *J* = 7.1, 1.1 Hz, 2H), 2.05 (dq, *J* = 12.9, 7.5 Hz, 1H), 1.40 (s, 9H). ^{13}C NMR (126 MHz, $CDCl_3$) δ 174.0, 172.1, 141.1, 131.0, 130.4, 130.2, 126.5, 122.6, 81.4, 51.3, 33.1, 28.7, 27.9. IR (FTIR), cm^{-1}: 3198, 2980, 1726, 1666, 1370, 1476, 1370, 1148, 768, 693. HRMS (ESI-TOF), m/z: $[M + Na]^+$ calculated for $[C_{15}H_{20}BrNNaO_3]^+$, 364.0519; found 364.0518. Chiral HPLC method: CHIRALCEL® OX-3R column, 45% acetonitrile, 55% water; t_R1 = 7.88 min, t_R2 = 8.56 min.

tert-butyl 5-amino-2-(4-bromophenyl)-5-oxopentanoate (**30**):

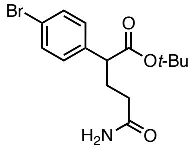

30

Yields: 39%, 37%, 39%. Enantiomeric ratio: 50:50.
^{1}H NMR (500 MHz, $CDCl_3$) δ 7.44 (d, *J* = 8.4 Hz, 2H), 7.16 (d, *J* = 8.4 Hz, 2H), 3.50 (t, *J* = 7.7 Hz, 1H), 2.31 (dt, *J* = 14.9, 7.3 Hz, 1H), 2.15 (td, *J* = 7.1, 1.7 Hz, 2H), 2.09 – 1.99 (m, 1H), 1.38 (s, 9H). ^{13}C NMR (126 MHz, $CDCl_3$) δ 174.2, 172.3, 137.9, 131.7, 129.6, 121.2, 81.2, 51.0, 33.1, 28.6, 27.9. IR (FTIR), cm^{-1}: 3345, 3196, 2980, 2935, 1726, 1668, 1489, 1369, 1148, 1012, 829. HRMS (ESI-TOF), m/z: $[M + Na]^+$ calculated for $[C_{15}H_{20}BrNNaO_3]^+$, 364.0519; found 364.0518. Chiral HPLC method: CHIRALCEL® OX-3R column, 45% acetonitrile, 55% water; t_R1 = 8.00 min, t_R2 = 9.27 min.

tert-butyl 5-amino-2-(4-methoxyphenyl)-5-oxopentanoate (**31**):

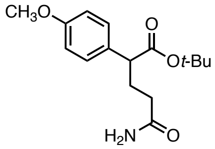

31

Yields: 55%, 56%, 58%. Enantiomeric ratio: 57:43.
^{1}H NMR (500 MHz, $CDCl_3$): δ 7.20 (d, *J* = 8.7 Hz, 2H), 6.85 (d, *J* = 8.7 Hz, 2H), 3.79 (s, 3H), 3.46 (t, *J* = 7.7 Hz, 1H), 2.31 (dq, *J* = 13.2, 7.3 Hz, 1H), 2.16 (td, *J* = 7.0, 1.8 Hz, 2H), 2.10 – 2.00 (m, 1H), 1.39 (s, 9H). ^{13}C NMR (126 MHz, $CDCl_3$) δ 174.6, 173.0, 158.8, 130.8, 128.9, 114.0, 80.8, 55.2, 50.8, 33.3, 28.8, 28.0. IR (FTIR), cm^{-1}: 2978, 2937, 1722, 1666, 1513, 1370, 1249, 1148, 1036, 834. HRMS (ESI-TOF), m/z: $[M + Na]^+$ calculated for $[C_{16}H_{23}NNaO_4]^+$, 316.1519; found 316.1520. Chiral HPLC method: CHIRALCEL® OX-3R column, 55% acetonitrile, 45% water; t_R1 = 3.46 min, t_R2 = 3.83 min.

tert-butyl 5-amino-2-(4-((*tert*-butoxycarbonyl)amino)phenyl)-5-oxopentanoate (**32**):

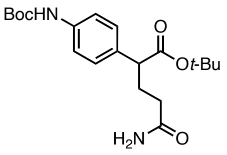

32

Note: 1 mol% GluER HA$_{rac}$ was used.

Yields: 21%, 22%, 25%. Enantiomeric ratio: 50:50.

^{1}H NMR (500 MHz, $CDCl_3$): δ 7.31 (d, *J* = 8.3 Hz, 2H), 7.20 (d, *J* = 8.6 Hz, 2H), 3.46 (t, *J* = 7.7 Hz, 1H), 2.37 – 2.26 (m, 1H), 2.18 – 2.11 (m, 2H), 2.06 – 2.00 (m, 1H), 1.51 (s, 9H), 1.38 (s, 9H). ^{13}C NMR (126 MHz, $CDCl_3$) δ 174.5, 172.8, 152.7, 137.4, 133.3, 128.5, 118.7, 80.9, 80.9, 51.0, 33.2, 28.7, 28.3, 27.9. IR (FTIR), cm^{-1}: 3004, 2980, 1726, 1672, 1527, 1370, 1157, 844. HRMS (ESI-TOF), m/z: [M + Na]$^+$ calculated for $[C_{20}H_{30}N_2NaO_5]^+$, 401.2047; found 401.2048. Chiral HPLC method: CHIRALCEL® OX-3R column, 55% acetonitrile, 45% water; t_R1 = 4.20 min, t_R2 = 4.79 min.

methyl-5-amino-5-oxo-2-phenylpentanoate (**33**):

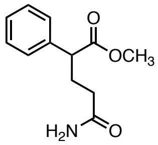

33

Note: 0.75 mol% of GluER HA$_{ent}$ was used instead of *GluER HA$_{rac}$*.

Yields: 72%, 75%, 75%. Enantiomeric ratio: 51:49.

^{1}H NMR (500 MHz, $CDCl_3$): δ 7.36 – 7.24 (m, 5H), 3.70 – 3.61 (m, 4H), 2.46 – 2.34 (m, 1H), 2.20 – 2.13 (m, 3H). ^{13}C NMR (126 MHz, $CDCl_3$): δ 174.1, 174.0, 138.2, 128.8, 128.0, 127.5, 52.1, 50.4, 33.1, 28.7. IR (FTIR), cm^{-1}: 3341, 3199, 2955, 1732, 1666, 1455, 1436, 1408, 1163, 736, 700. HRMS (ESI-TOF), m/z: [M + Na]$^+$ calculated for $[C_{12}H_{15}NNaO_3]^+$, 244.0944; found 244.0948. Chiral HPLC method: CHIRALPAK® IG column, 5% *iso*-propanol, 95% hexane; t_R1 = 36.82 min, t_R2 = 42.03 min.

ethyl-5-amino-5-oxo-2-phenylpentanoate (**34**):

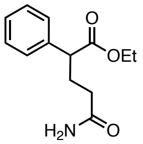

34

Yields: 75%, 77%, 79%. Enantiomeric ratio: 50:50.

^{1}H NMR (500 MHz, $CDCl_3$): δ 7.32 – 7.19 (m, 5H), 4.16 – 3.98 (m, 2H), 3.58 (t, *J* = 7.5 Hz, 1H), 2.35 – 2.03 (m, 1H), 2.19 – 2.03 (m, 3H), 1.16 (t, *J* = 7.1 Hz, 3H). ^{13}C NMR (126 MHz, $CDCl_3$): δ 174.5, 173.5, 138.4, 128.7, 127.9, 127.4, 60.9, 50.6, 33.2, 28.7, 14.1. IR (FTIR), cm^{-1}: 3332, 3201, 2981, 2939, 1726, 1662, 1372, 1155, 1021, 734, 699. HRMS (ESI-TOF), m/z: [M + Na]$^+$ calculated for $[C_{13}H_{17}NNaO_3]^+$, 258.1101; found 258.1101. Chiral HPLC method: CHIRALPAK® IG column, 5% *iso*-propanol, 95% hexane; t_R1 = 49.72 min, t_R2 = 55.32 min.

benzyl 5-amino-5-oxo-2-phenylpentanoate (**35**):

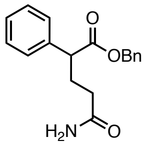

35

Yields: 34%, 35%, 34%. Enantiomeric ratio: 52:48.
^{1}H NMR (500 MHz, $CDCl_3$): δ 7.34 – 7.27 (m, 8H), 7.25 – 7.22 (m, 2H), 5.15 (d, *J* = 12.4 Hz, 1H), 5.07 (d, *J* = 12.4 Hz, 1H), 3.75 – 3.67 (m, 1H), 2.45 – 2.33 (m, 1H), 2.22 – 2.09 (m, 3H). ^{13}C NMR (126 MHz, $CDCl_3$): δ 174.0, 173.3, 138.1, 135.8, 128.7, 128.5, 128.2, 128.0, 128.0, 127.5, 66.5, 50.6, 33.0, 28.6. IR (FTIR), cm^{-1}: 3453, 3350, 3196, 2939, 1729, 1664, 1498, 1455, 1258, 1213, 1154, 736, 699. HRMS (ESI-TOF), m/z: [M + Na]$^+$ calculated for $[C_{18}H_{19}NNaO_3]^+$, 320.1257; found 320.1263. Chiral HPLC method: CHIRALCEL® OX-3R column, 45% acetonitrile, 55% water; t_R1 = 7.27 min, t_R2 = 8.69 min.

tert-pentyl 5-amino-5-oxo-2-phenylpentanoate (**36**):

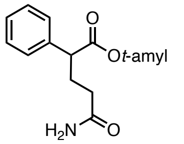

36

Yields: 38%, 37%, 41%. Enantiomeric ratio: 52:48.
^{1}H NMR (500 MHz, $CDCl_3$): δ 7.35 – 7.21 (m, 5H), 3.53 (t, *J* = 7.6 Hz, 1H), 2.41 – 2.30 (m, 1H), 2.22 – 2.15 (m, 2H), 2.14 – 2.04 (m, 1H), 1.70 (qd, *J* = 7.5, 1.1 Hz, 2H), 1.36 (s, 3H), 1.34 (s, 3H), 0.73 (t, *J* = 7.5 Hz, 3H). ^{13}C NMR (126 MHz, $CDCl_3$) δ 174.3, 172.7, 138.9, 128.6, 127.9, 127.2, 83.4, 51.7, 33.4, 33.3, 28.6, 25.5, 25.2, 8.0. IR (FTIR), cm^{-1}: 3431, 3347, 3194, 2977, 2935, 1723, 1664, 1455, 1146, 741, 699. HRMS (ESI-TOF), m/z: [M + Na]$^+$ calculated for $[C_{16}H_{23}NNaO_3]^+$, 300.1570; found 300.1566. Chiral HPLC method: CHIRALCEL® OX-3R column, 45% acetonitrile, 55% water; t_R1 = 7.19 min, t_R2 = 8.43 min.

tert-butyl 5-amino-4-methyl-5-oxo-2-phenylpentanoate (**37**):

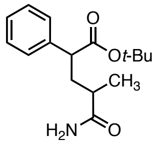

37

*Note: 2-Bromopropanamide (**S15a**) was used for the synthesis of **37**. Product was acquired as an inseparable 1:1 diastereomeric mixture; NMR data were acquired on a partially separated diastereomeric mixture (1.4:1 dr) to assign signals for each diastereomer. Relative configurations were not determined.*

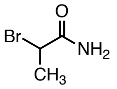

S15a

Yields: 44%, 44%, 45%. Enantiomeric ratio: 58:42; 39:61.
^{1}H NMR (500 MHz, $CDCl_3$, * denotes the other diastereomer): δ 7.35 – 7.21 (m, 5H, 5H*), 3.59 – 3.52 (m, 1H, 1H*), 2.36 (ddd, *J* = 13.8, 8.4, 6.9 Hz, 1H), 2.27 (qd, *J* = 6.5, 1.7 Hz, 1H*), 2.15 – 2.00 (m, 1H, 1H*), 1.90 – 1.80 (m, 1H, 1H*), 1.39 (s, 9H*), 1.36 (s, 9H), 1.18 (d, *J* = 6.8 Hz, 3H*),

1.16 (d, J = 6.9 Hz, 3H). ^{13}C NMR (126 MHz, $CDCl_3$, * denotes the other diastereomer) δ 178.2*, 178.1, 173.0*, 172.9, 139.0*, 138.9, 128.6, 128.6*, 127.9, 127.7*, 127.2, 127.2*, 81.0*, 80.9, 50.4*, 50.3, 38.5*, 38.0, 37.8*, 37.2, 27.9*, 27.9, 18.0, 17.9*. IR (FTIR), cm^{-1}: 3194, 2978, 2933, 1728, 1668, 1456, 1370, 1150, 846, 751, 699. HRMS (ESI-TOF), m/z: [M + Na]$^+$ calculated for $[C_{16}H_{23}NNaO_3]^+$, 300.1570; found 300.1576. Chiral HPLC method (* denotes the other diastereomer): CHIRALPAK® IG column, 5% *iso*-propanol, 95% hexane; t_R1 = 24.80 min, t_R2 = 26.82 min, t_R1^* = 29.70 min, t_R2^* = 32.09 min.

tert-butyl 4-carbamoyl-2-phenylhexanoate (**38**):

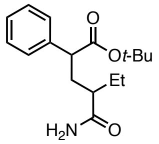

38

*Note: 2-Chlorobutanamide (**S16**) was used for the synthesis of **38**. The product was acquired as an inseparable 1.2:1 diastereomeric mixture, on which NMR data were acquired. Relative configurations were not determined.*

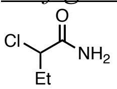

S16

Yields: 40%, 38%, 41%. Enantiomeric ratio: 50:50; 50:50.

^{1}H NMR (500 MHz, $CDCl_3$, * denotes minor diastereomer): δ 7.36 – 7.20 (m, 5H, 5H*), 3.58 – 3.49 (m, 1H, 1H*), 2.28 (ddd, J = 13.9, 9.7, 5.7 Hz, 1H), 2.14 – 2.07 (m, 1H, 1H*), 2.00 – 1.90 (m, 1H, 1H*), 1.79 (tt, J = 9.6, 4.9 Hz, 1H*), 1.70 – 1.60 (m, 1H, 1H*), 1.58 – 1.42 (m, 1H, 1H*), 1.40 (s, 9H*), 1.35 (s, 9H), 0.94 (t, J = 7.4 Hz, 3H*), 0.87 (t, J = 7.4 Hz, 3H). ^{13}C NMR (126 MHz, $CDCl_3$, * denotes minor diastereomer): δ 177.3*, 177.2, 173.0*, 172.9, 139.4*, 138.8, 128.7, 128.6*, 128.1, 127.7*, 127.3, 127.1*, 81.0*, 80.8, 50.6*, 50.3, 46.5*, 45.5, 36.5*, 35.2, 28.0*, 27.9, 26.1, 26.1*, 11.9*, 11.9. IR (FTIR), cm^{-1}: 2970, 2933, 1728, 1666, 1455, 1370, 1150, 753. HRMS (ESI-TOF), m/z: [M + Na]$^+$ calculated for $[C_{17}H_{25}NNaO_3]^+$, 314.1727; found 314.1728. Chiral HPLC method (* denotes the minor diastereomer): CHIRALPAK® IE column, 2% *iso*-propanol, 98% hexane; t_R1 = 72.52 min, t_R2 = 79.55 min, t_R1^* = 85.01 min, t_R2^* = 88.16 min.

tert-butyl 5-amino-4-chloro-5-oxo-2-phenylpentanoate (**39**):

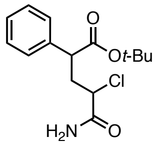

39

*Note: 2,2-Dichloroacetamide (**S17**) was used for the synthesis of **39**. The product was acquired as an inseparable 1.5:1 diastereomeric mixture, on which NMR data were acquired. Relative configurations were not determined.*

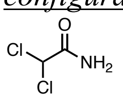

S17

Yields: 33%, 32%, 37%. Enantiomeric ratio: 52:48; 68:32.

^{1}H NMR (500 MHz, $CDCl_3$, * denotes minor diastereomer): δ 7.38 – 7.23 (m, 5H, 5H*), 4.33 (dd, *J* = 8.9, 5.2 Hz, 1H), 3.92 (dd, *J* = 11.1, 3.6 Hz, 1H*), 3.84 (dd, *J* = 9.5, 5.6 Hz, 1H), 3.79 (dd, *J* = 10.4, 5.1 Hz, 1H*), 2.92 (ddd, *J* = 14.6, 9.6, 5.2 Hz, 1H), 2.75 (ddd, *J* = 14.3, 10.5, 3.6 Hz, 1H*), 2.43 (ddd, *J* = 14.7, 11.1, 5.1 Hz, 1H*), 2.19 (ddd, *J* = 14.5, 9.0, 5.6 Hz, 1H), 1.39 (s, 9H), 1.38 (s, 9H*). ^{13}C NMR (126 MHz, $CDCl_3$, * denotes minor diastereomer): δ 171.9*, 171.8, 170.9*, 170.7, 138.5, 137.1*, 128.9*, 128.7, 128.2*, 127.9, 127.6*, 127.4, 81.3, 81.2*, 58.0*, 57.9, 49.5*, 49.3, 39.0, 38.4*, 27.9, 27.9*. IR (FTIR), cm^{-1}: 2981, 2926, 1722, 1669, 1455, 1395, 1370, 1150, 846, 751, 701. HRMS (ESI-TOF), m/z: $[M + Na]^+$ calculated for $[C_{15}H_{20}ClNNaO_3]^+$, 320.1024; found 320.1022. Chiral HPLC method (* denotes the minor diastereomer): CHIRALPAK® IG column, 2% *iso*-propanol, 98% hexane; t_R1 = 48.59 min, t_R2 = 53.57 min, t_R1^* = 57.31 min, t_R2^* = 61.23 min.

tert-butyl 5-amino-5-oxo-2-(pyridin-3-yl)pentanoate (**40**):

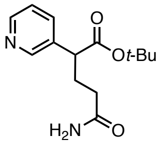

40

Note: 1 mol% GluER HA_{rac} was used.

Yields: 28%, 21%, 20%. Enantiomeric ratio: 51:49.

^{1}H NMR (500 MHz, $CDCl_3$): δ 8.60 (d, *J* = 23.7 Hz, 2H), 7.99 (d, *J* = 8.0 Hz, 1H), 7.53 (dd, *J* = 8.0, 5.1 Hz, 1H), 3.71 (t, *J* = 7.7 Hz, 1H), 2.41 (dq, *J* = 14.5, 7.2 Hz, 1H), 2.21 (td, *J* = 6.9, 1.6 Hz, 2H), 2.16 – 2.08 (m, 1H), 1.41 (s, 9H). ^{13}C NMR (126 MHz, $CDCl_3$) δ 173.3, 171.2, 145.9, 144.8, 139.5, 136.8, 124.9, 82.4, 49.0, 32.7, 28.6, 27.9. IR (FTIR), cm^{-1}: 3365, 2922, 2851, 1724, 1692, 1671, 1528, 1370, 1241, 1211, 1150, 713, 700. HRMS (ESI-TOF), m/z: $[M + H]^+$ calculated for $[C_{14}H_{21}N_2O_3]^+$, 265.1547; found 265.1546. Chiral HPLC method: CHIRALPAK® IG column, 20% *iso*-propanol, 80% hexane; t_R1 = 18.18 min, t_R2 = 32.38 min.

tert-butyl 5-amino-2-(6-methylpyridin-3-yl)-5-oxopentanoate (**41**):

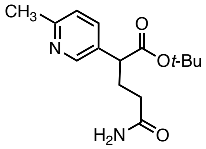

41

Note: 1 mol% GluER HA_{rac} was used.

Yields: 44%, 43%, 51%. Enantiomeric ratio: 52:48.

^{1}H NMR (500 MHz, $CDCl_3$) δ 8.39 (d, *J* = 2.3 Hz, 1H), 7.58 (dd, *J* = 8.0, 2.4 Hz, 1H), 7.14 (d, *J* = 8.1 Hz, 1H), 3.53 (t, *J* = 7.7 Hz, 1H), 2.55 (s, 3H), 2.36 (dq, *J* = 13.5, 7.5 Hz, 1H), 2.17 (dd, *J* = 8.0, 6.8 Hz, 2H), 2.05 (dq, *J* = 13.4, 7.5 Hz, 1H), 1.39 (s, 9H). ^{13}C NMR (126 MHz, $CDCl_3$) δ 173.9, 172.1, 157.3, 148.5, 135.8, 131.6, 123.4, 81.5, 48.7, 33.0, 28.5, 27.9, 23.9. IR (FTIR), cm^{-1}: 3414, 3311, 3190, 2978, 2935, 1723, 1672, 1396, 1370, 1299, 1251, 1152, 1032, 842. HRMS (ESI-TOF), m/z: $[M + H]^+$ calculated for $[C_{15}H_{23}N_2O_3]^+$, 279.1703; found 279.1708. Chiral HPLC method: CHIRALPAK® IG column, 20% *iso*-propanol, 80% hexane; t_R1 = 17.08 min, t_R2 = 19.87 min.

tert-butyl 5-amino-2-(6-methoxypyridin-3-yl)-5-oxopentanoate (**42**):

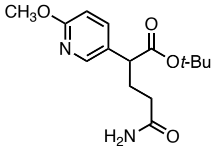

42

Yields: 78%, 79%, 77%. Enantiomeric ratio: 55:45.

^{1}H NMR (500 MHz, $CDCl_3$) δ 8.03 (d, J = 2.5 Hz, 1H), 7.56 (dd, J = 8.6, 2.5 Hz, 1H), 6.72 (d, J = 8.5 Hz, 1H), 3.92 (s, 3H), 3.48 (t, J = 7.8 Hz, 1H), 2.33 (dq, J = 13.4, 7.5 Hz, 1H), 2.16 (td, J = 7.2, 1.1 Hz, 2H), 2.08 – 1.98 (m, 1H), 1.39 (s, 9H). ^{13}C NMR (126 MHz, $CDCl_3$) δ 174.0, 172.4, 163.5, 146.2, 137.9, 127.2, 111.0, 81.3, 53.5, 48.3, 33.1, 28.5, 27.9. IR (FTIR), cm^{-1}: 3201, 2981, 2948, 1726, 1670, 1608, 1478, 1398, 1289, 1179, 1029, 839. HRMS (ESI-TOF), m/z: $[M + H]^+$ calculated for $[C_{15}H_{23}N_2O_4]^+$, 295.1652; found 295.1655. HPLC method: CHIRALCEL® OX-3R column, 30% acetonitrile, 70% water; t_R1 = 7.32 min, t_R2 = 7.93 min.

tert-butyl 5-amino-2-(6-chloropyridin-3-yl)-5-oxopentanoate (**43**):

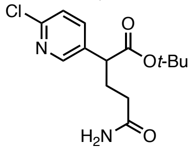

43

Yields: 77%, 77%, 78%. Enantiomeric ratio: 52:48.

^{1}H NMR (500 MHz, $CDCl_3$) δ 8.28 (d, J = 2.6 Hz, 1H), 7.64 (dd, J = 8.3, 2.6 Hz, 1H), 7.30 (d, J = 8.3 Hz, 1H), 3.57 (t, J = 7.8 Hz, 1H), 2.35 (dq, J = 13.5, 7.5 Hz, 1H), 2.17 (dd, J = 7.8, 6.9 Hz, 2H), 2.09 – 1.98 (m, 1H), 1.39 (s, 9H). ^{13}C NMR (126 MHz, $CDCl_3$) δ 173.7, 171.6, 150.5, 149.4, 138.0, 133.6, 124.3, 81.9, 48.3, 32.8, 28.5, 27.9. IR (FTIR), cm^{-1}: 3337, 3196, 2980, 1723, 1661, 1459, 1392, 1370, 1148, 1107, 840. HRMS (ESI-TOF), m/z: $[M + H]^+$ calculated for $[C_{14}H_{20}ClN_2O_3]^+$, 299.1157; found 299.1158. Chiral HPLC method: CHIRALPAK® IG column, 20% *iso*-propanol, 80% hexane; t_R1 = 12.68 min, t_R2 = 16.71 min.

tert-butyl 5-amino-5-oxo-2-(pyrimidin-5-yl)pentanoate (**44**):

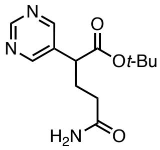

44

Note: 1 mol% GluER HA$_{rac}$ was used.

Yields: 59%, 56%, 64%. Enantiomeric ratio: 52:48.

^{1}H NMR (500 MHz, $CDCl_3$) δ 9.14 (s, 1H), 8.70 (s, 2H), 3.60 (dd, J = 8.3, 7.3 Hz, 1H), 2.41 (ddt, J = 13.8, 8.4, 7.2 Hz, 1H), 2.23 (td, J = 7.0, 1.3 Hz, 2H), 2.10 (dq, J = 14.4, 7.2 Hz, 1H), 1.41 (s, 9H). ^{13}C NMR (126 MHz, $CDCl_3$) δ 173.4, 171.0, 157.8, 156.4, 132.6, 82.3, 47.2, 32.7, 28.3, 27.9. IR (FTIR), cm^{-1}: 3334, 3198, 2980, 2937, 1728, 1671, 1564, 1414, 1370, 1150, 726. HRMS (ESI-TOF), m/z: $[M + H]^+$ calculated for $[C_{13}H_{20}N_3O_3]^+$, 266.1499; found 266.1492. Chiral HPLC method: CHIRALPAK® IE column, 50% *iso*-propanol, 50% hexane; t_R1 = 7.70 min, t_R2 = 9.81 min.

tert-butyl 5-amino-2-(naphthalen-2-yl)-5-oxopentanoate (**45**):

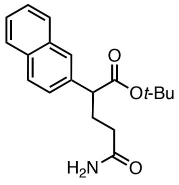

45

Yields: 19%, 19%, 20%. Enantiomeric ratio: 48:52.
^{1}H NMR (500 MHz, $CDCl_3$) δ 7.85 – 7.77 (m, 3H), 7.72 (s, 1H), 7.50 – 7.45 (m, 2H), 7.44 (dd, *J* = 8.5, 1.8 Hz, 1H), 3.70 (t, *J* = 7.2 Hz, 1H), 2.49 – 2.38 (m, 1H), 2.25 – 2.13 (m, 3H), 1.39 (s, 9H). ^{13}C NMR (126 MHz, $CDCl_3$) δ 174.3, 172.7, 136.3, 133.4, 132.6, 128.4, 127.8, 127.6, 126.9, 126.1, 125.8, 125.8, 81.1, 51.7, 33.2, 28.6, 27.9. IR (FTIR), cm^{-1}: 3190, 2978, 2933, 1724, 1666, 1370, 1148, 861, 820, 749. HRMS (ESI-TOF), m/z: [M + Na]$^{+}$ calculated for $[C_{19}H_{23}NNaO_3]^{+}$, 336.1570; found 336.1572. Chiral HPLC method: CHIRALCEL® OX-3R column, 30% acetonitrile, 70% water; t_R1 = 5.14 min, t_R2 = 6.04 min.

tert-butyl 5-amino-5-oxo-2-(quinolin-3-yl)pentanoate (**46**):

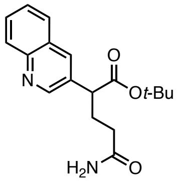

46

Yields: 53%, 46%, 48%. Enantiomeric ratio: 52:48.
^{1}H NMR (500 MHz, $CDCl_3$) δ 8.89 (d, *J* = 2.2 Hz, 1H), 8.23 – 8.18 (m, 2H), 7.85 (dd, *J* = 8.3, 1.4 Hz, 1H), 7.76 (ddd, *J* = 8.4, 6.8, 1.4 Hz, 1H), 7.61 (ddd, *J* = 8.1, 6.9, 1.2 Hz, 1H), 3.85 – 3.78 (m, 1H), 2.55 – 2.44 (m, 1H), 2.29 – 2.14 (m, 3H), 1.41 (s, 9H). ^{13}C NMR (126 MHz, $CDCl_3$) δ 173.6, 171.7, 149.5, 145.6, 136.3, 132.0, 130.4, 128.1, 127.9, 127.8, 127.6, 82.0, 49.1, 32.9, 28.7, 27.9. IR (FTIR), cm^{-1}: 3192, 2976, 1724, 1671, 1370, 1150, 905, 848, 757. HRMS (ESI-TOF), m/z: [M + H]$^{+}$ calculated for $[C_{18}H_{23}N_2O_3]^{+}$, 315.1703; found 315.1707. Chiral HPLC method: CHIRALCEL® OX-3R column, 20% acetonitrile, 80% water; t_R1 = 10.00 min, t_R2 = 10.93 min.

tert-butyl 5-amino-5-oxo-2-(quinolin-4-yl)pentanoate (**47**):

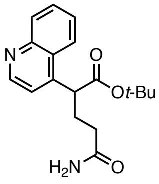

47

Yields: 56%, 54%, 52%. Enantiomeric ratio: 52:48.
^{1}H NMR (500 MHz, $CDCl_3$) δ 8.88 (d, *J* = 4.7 Hz, 1H), 8.22 (dd, *J* = 8.4, 1.5 Hz, 2H), 7.77 (ddd, *J* = 8.4, 6.8, 1.3 Hz, 1H), 7.64 (ddd, *J* = 8.2, 6.8, 1.3 Hz, 1H), 7.43 (d, *J* = 4.6 Hz, 1H), 4.48 (dd, *J* = 8.6, 5.9 Hz, 1H), 2.54 – 2.43 (m, 1H), 2.37 – 2.19 (m, 3H), 1.36 (s, 9H). ^{13}C NMR (126 MHz, $CDCl_3$) δ 173.8, 171.5, 149.0, 147.2, 146.9, 130.0, 129.4, 127.4, 127.1, 123.5, 119.2, 82.0, 46.5, 32.8, 28.0, 27.8. IR (FTIR), cm^{-1}: 3197, 2976, 2926, 1728, 1672, 1395, 1370, 1150, 848, 764. HRMS (ESI-TOF), m/z: [M + H]$^{+}$ calculated for $[C_{18}H_{23}N_2O_3]^{+}$, 315.1703; found 315.1708.

Chiral HPLC method: CHIRALCEL® OX-3R column, 20% acetonitrile, 80% water; t_R1 = 5.49 min, t_R2 = 6.04 min.

tert-butyl 5-amino-5-oxo-2-(quinolin-5-yl)pentanoate (**48**):

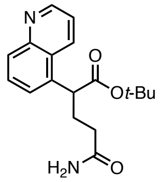

48

Yields: 63%, 60%, 58%. Enantiomeric ratio: 50:50.
^{1}H NMR (500 MHz, $CDCl_3$): δ 8.93 (dd, *J* = 4.1, 1.6 Hz, 1H), 8.63 (d, *J* = 8.7 Hz, 1H), 8.10 (d, *J* = 8.4 Hz, 1H), 7.71 (dd, *J* = 8.5, 7.2 Hz, 1H), 7.55 (dd, *J* = 7.2, 1.2 Hz, 1H), 7.49 (dd, *J* = 8.7, 4.2 Hz, 1H), 4.41 (dd, *J* = 8.6, 6.1 Hz, 1H), 2.54 – 2.43 (m, 1H), 2.35 – 2.17 (m, 3H), 1.34 (s, 9H). ^{13}C NMR (126 MHz, $CDCl_3$): δ 174.1, 172.5, 149.4, 147.7, 136.2, 133.1, 129.6, 128.4, 127.0, 125.4, 121.1, 81.5, 46.3, 32.9, 28.2, 27.9. IR (FTIR), cm^{-1}: 3359, 3196, 2978, 2933, 1722, 1670, 1370, 1149, 846, 805. HRMS (ESI-TOF), m/z: $[M + H]^+$ calculated for $[C_{18}H_{23}N_2O_3]^+$, 315.1703; found 315.1708. Chiral HPLC method: CHIRALCEL® OX-3R column, 20% acetonitrile, 80% water; t_R1 = 5.17 min, t_R2 = 5.68 min.

tert-butyl 5-amino-2-(1-methyl-1*H*-indol-6-yl)-5-oxopentanoate (**49**):

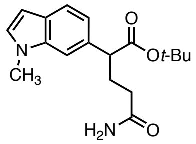

49

Note: 1 mol% GluER HA$_{rac}$ was used.
Yields: 33%, 36%, 40%. Enantiomeric ratio: 49:51.
^{1}H NMR (500 MHz, $CDCl_3$) δ 7.55 (dd, *J* = 8.1, 0.6 Hz, 1H), 7.26 – 7.23 (m, 1H), 7.06 – 6.99 (m, 2H), 6.45 (dd, *J* = 3.0, 0.9 Hz, 1H), 3.78 (s, 3H), 3.68 – 3.58 (m, 1H), 2.45 – 2.31 (m, 1H), 2.23 – 2.10 (m, 3H), 1.39 (s, 9H). ^{13}C NMR (126 MHz, $CDCl_3$) δ 174.6, 173.4, 132.3, 129.1, 127.6, 125.5, 120.9, 119.6, 108.5, 100.7, 80.7, 52.0, 33.4, 32.9, 29.3, 28.0. IR (FTIR), cm^{-1}: 2976, 2924, 1720, 1694, 1370, 1148. HRMS (ESI-TOF), m/z: $[M + Na]^+$ calculated for $[C_{18}H_{24}N_2NaO_3]^+$, 339.1679; found 339.1678. Chiral HPLC method: CHIRALCEL® OX-3R column, 45% acetonitrile, 55% water; t_R1 = 6.85 min, t_R2 = 8.62 min.

tert-butyl 5-amino-2-(1-methyl-1*H*-indazol-6-yl)-5-oxopentanoate (**50**):

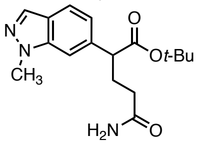

50

Yields: 70%, 74%, 74%. Enantiomeric ratio: 52:48.
^{1}H NMR (500 MHz, $CDCl_3$): δ 7.93 (d, *J* = 0.9 Hz, 1H), 7.65 (dd, *J* = 8.3, 0.8 Hz, 1H), 7.31 (s, 1H), 7.08 (dd, *J* = 8.3, 1.4 Hz, 1H), 4.06 (s, 3H), 3.72 – 3.63 (m, 1H), 2.46 – 2.36 (m, 1H), 2.22 –

2.10 (m, 3H), 1.39 (s, 9H). ^{13}C NMR (126 MHz, $CDCl_3$) δ 174.2, 172.7, 140.2, 137.4, 132.5, 123.3, 121.2, 120.9, 108.0, 81.1, 52.0, 35.5, 33.2, 29.0, 27.9. IR (FTIR), cm^{-1}: 3200, 2976, 2937, 1724, 1672, 1370, 1150, 844. HRMS (ESI-TOF), m/z: $[M + H]^+$ calculated for $[C_{17}H_{24}N_3O_3]^+$, 318.1812; found 318.1812. Chiral HPLC method: CHIRALCEL® OX-3R column, 30% acetonitrile, 70% water; t_R1 = 12.51 min, t_R2 = 14.49 min.

tert-butyl 5-amino-2-(3-methyl-2-oxo-2,3-dihydrobenzo[*d*]oxazol-6-yl)-5-oxopentanoate (**51**):

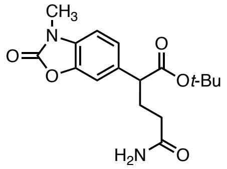

51

Yields: 56%, 54%, 62%. Enantiomeric ratio: 49:51.
^{1}H NMR (500 MHz, $CDCl_3$) δ 7.18 (d, *J* = 1.6 Hz, 1H), 7.12 (dd, *J* = 8.1, 1.6 Hz, 1H), 6.90 (d, *J* = 8.0 Hz, 1H), 3.55 (t, *J* = 7.7 Hz, 1H), 3.39 (s, 3H), 2.39 – 2.29 (m, 1H), 2.17 (t, *J* = 7.3 Hz, 2H), 2.12 – 2.01 (m, 1H), 1.39 (s, 9H). ^{13}C NMR (126 MHz, $CDCl_3$) δ 174.2, 172.5, 154.8, 142.8, 133.8, 131.0, 123.6, 109.5, 108.0, 81.3, 51.5, 33.1, 29.0, 28.2, 27.9. IR (FTIR), cm^{-1}: 3445, 3345, 3211, 2976, 2939, 1774, 1715, 1666, 1505, 1370, 1150, 937, 751. HRMS (ESI-TOF), m/z: $[M + Na]^+$ calculated for $[C_{17}H_{22}N_2NaO_5]^+$, 357.1421; found 357.1420. Chiral HPLC method: CHIRALCEL® OX-3R column, 45% acetonitrile, 55% water; t_R1 = 4.57 min, t_R2 = 5.07 min.

tert-butyl 5-amino-5-oxo-2-(1,3,3-trimethyl-2-oxoindolin-6-yl)pentanoate (**52**):

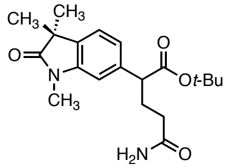

52

Yields: 51%, 55%, 50%. Enantiomeric ratio: 49:51.
^{1}H NMR (500 MHz, $CDCl_3$) δ 7.12 (d, *J* = 7.5 Hz, 1H), 6.95 (dd, *J* = 7.5, 1.6 Hz, 1H), 6.79 (d, *J* = 1.5 Hz, 1H), 3.54 (t, *J* = 7.7 Hz, 1H), 3.20 (s, 3H), 2.40 – 2.29 (m, 1H), 2.20 (d, *J* = 8.0 Hz, 2H), 2.14 – 2.02 (m, 1H), 1.42 (s, 9H), 1.35 (s, 6H). ^{13}C NMR (126 MHz, $CDCl_3$) δ 181.5, 174.3, 172.7, 143.0, 138.7, 134.8, 122.3, 122.1, 107.4, 81.2, 51.9, 44.0, 33.3, 29.2, 28.0, 26.3, 24.4. IR (FTIR), cm^{-1}: 3345, 3205, 2973, 2931, 2873, 1715, 1677, 1621, 1449, 1387, 1370, 1252, 1148, 844. HRMS (ESI-TOF), m/z: $[M + Na]^+$ calculated for $[C_{20}H_{28}N_2NaO_4]^+$, 383.1941; found 383.1939. Chiral HPLC method: CHIRALCEL® OX-3R column, 45% acetonitrile, 55% water; t_R1 = 4.96 min, t_R2 = 5.31 min.

*Synthesis of the enantioenriched products **20–29**, **35–38**, **41–42**, and **49–50** via* <u>Procedure B.</u>

Yields for enantioenriched **20** were determined by LC-MS. Yields for enantioenriched **21**–**29**, **35**–**38**, **41**–**42**, and **49**–**50** were determined by NMR. 2-Chloroacetamide (**53**) was used to synthesize **21**–**29**, **35**–**36**, **41**–**42**, and **49**–**50**. The amides used for the synthesis of **37**–**38** are specified below. The absolute stereochemistry of **20** was determined by procedures detailed in the next section. The absolute stereochemistry of all other products was assigned by analogy to **20**.

enantioenriched *tert*-butyl 5-amino-5-oxo-2-phenylpentanoate (**20**):

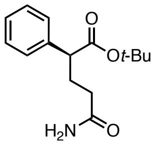

20

Yields: 84%, 86%, 82%. Enantiomeric ratio: 93:7.

Chiral HPLC method: CHIRALCEL® OX-3R column, 55% acetonitrile, 45% water; t_R1 = 3.57 min, t_R2 = 3.93 min.

enantioenriched *tert*-butyl 5-amino-5-oxo-2-(*o*-tolyl)pentanoate (**21**):

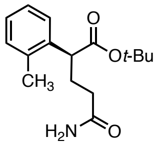

21

Yields: 43%, 38%, 38%. Enantiomeric ratio: 80:20.

Chiral HPLC method: CHIRALCEL® OX-3R column, 55% acetonitrile, 45% water; t_R1 = 3.99 min, t_R2 = 4.89 min.

enantioenriched *tert*-butyl 5-amino-5-oxo-2-(*m*-tolyl)pentanoate (**22**):

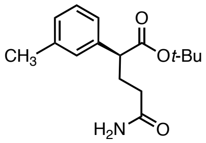

22

Yields: 49%, 50%, 56%. Enantiomeric ratio: 61:39.

Chiral HPLC method: CHIRALCEL® OX-3R column, 45% acetonitrile, 55% water; t_R1 = 5.98 min, t_R2 = 6.68 min.

enantioenriched *tert*-butyl 5-amino-5-oxo-2-(*p*-tolyl)pentanoate (**23**):

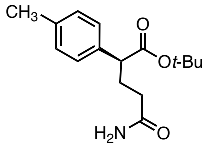

23

Yields: 41%, 47%, 47%. Enantiomeric ratio: 58:42.

Chiral HPLC method: CHIRALCEL® OX-3R column, 55% acetonitrile, 45% water; t_R1 = 4.11 min, t_R2 = 4.72 min.

enantioenriched *tert*-butyl 5-amino-2-(2-methoxyphenyl)-5-oxopentanoate (**24**):

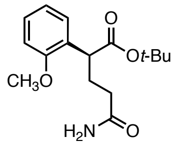

24

Yields: 56%, 60%, 62%. Enantiomeric ratio: 87:13.

Chiral HPLC method: CHIRALCEL® OX-3R column, 55% acetonitrile, 45% water; t_R1 = 3.67 min, t_R2 = 4.38 min.

enantioenriched *tert*-butyl 5-amino-2-(2-chlorophenyl)-5-oxopentanoate (**25**):

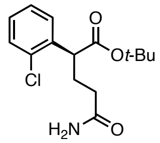

25

Yields: 24%, 25%, 25%. Enantiomeric ratio: 78:22.

Chiral HPLC method: CHIRALCEL® OX-3R column, 55% acetonitrile, 45% water; t_R1 = 4.22 min, t_R2 = 5.15 min.

enantioenriched *tert*-butyl 5-amino-2-(2-bromophenyl)-5-oxopentanoate (**26**):

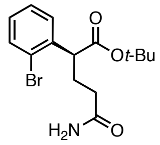

26

Yields: 24%, 22%, 28%. Enantiomeric ratio: 72:28.

Chiral HPLC method: CHIRALCEL® OX-3R column, 45% acetonitrile, 55% water; t_R1 = 7.77 min, t_R2 = 9.90 min.

enantioenriched *tert*-butyl 5-amino-2-(2-iodophenyl)-5-oxopentanoate (**27**):

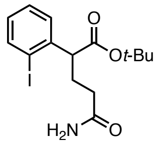

27

Yields: 15%, 15%, 15%. Enantiomeric ratio: 70:30.

Chiral HPLC method: CHIRALCEL® OX-3R column, 45% acetonitrile, 55% water; t_R1 = 9.13 min, t_R2 = 11.59 min.

enantioenriched *tert*-butyl 5-amino-2-(2-nitrophenyl)-5-oxopentanoate (**28**):

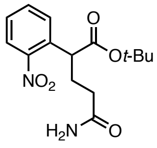

28

Yields: 29%, 25%, 29%. Enantiomeric ratio: 61:39.

Chiral HPLC method: CHIRALCEL® OX-3R column, 45% acetonitrile, 55% water; t_R1 = 5.09 min, t_R2 = 5.76 min.

enantioenriched *tert*-butyl 5-amino-2-(3-bromophenyl)-5-oxopentanoate (**29**):

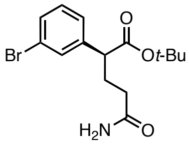

29

Yields: 24%, 25%, 25%. Enantiomeric ratio: 63:37.

Chiral HPLC method: CHIRALCEL® OX-3R column, 45% acetonitrile, 55% water; t_R1 = 7.88 min, t_R2 = 8.56 min.

enantioenriched benzyl 5-amino-5-oxo-2-phenylpentanoate (**35**):

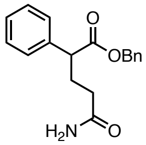

35

Yields: 34%, 38%, 38%. Enantiomeric ratio: 72:28.

Chiral HPLC method: CHIRALCEL® OX-3R column, 45% acetonitrile, 55% water; t_R1 = 7.28 min, t_R2 = 8.71 min.

enantioenriched *tert*-pentyl 5-amino-5-oxo-2-phenylpentanoate (**36**):

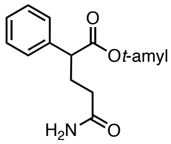

36

Yields: 33%, 33%, 34%. Enantiomeric ratio: 88:12.

Chiral HPLC method: CHIRALCEL® OX-3R column, 45% acetonitrile, 55% water; t_R1 = 7.19 min, t_R2 = 8.44 min.

enantioenriched *tert*-butyl 5-amino-4-methyl-5-oxo-2-phenylpentanoate (**37**):

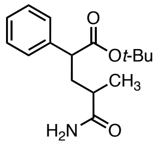

37

*Note: 2-Bromopropanamide (**S15a**) was used for the synthesis of enantioenriched **37**.*

Yields: 71%, 68%, 68%. Enantiomeric ratio: 93:7; 90:10.

Chiral HPLC method (* denotes the other diastereomer): CHIRALPAK® IG column, 5% *iso*-propanol, 95% hexane; t_R1 = 25.25 min, t_R2 = 27.52 min, t_R1^* = 29.69 min, t_R2^* = 32.63 min.

enantioenriched *tert*-butyl 4-carbamoyl-2-phenylhexanoate (**38**):

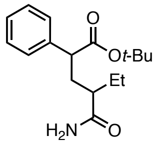

38

*Note: 2-Chlorobutanamide (**S16**) was used for the synthesis of enantioenriched **38**.*

Yields: 30%, 33%, 34%. Enantiomeric ratio: 63:37; 70:30.

Chiral HPLC method (* denotes the minor diastereomer): CHIRALPAK® IE column, 2% *iso*-propanol, 98% hexane; t_R1 = 71.93 min, t_R2 = 79.39 min, t_R1^* = 84.55 min, t_R2^* = 87.89 min.

enantioenriched *tert*-butyl 5-amino-2-(6-methylpyridin-3-yl)-5-oxopentanoate (**41**):

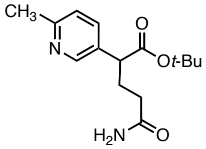

41

Yields: 40%, 41%, 42%. Enantiomeric ratio: 63:37.

Chiral HPLC method: CHIRALPAK® IG column, 20% *iso*-propanol, 80% hexane; t_R1 = 17.19 min, t_R2 = 19.77 min.

enantioenriched *tert*-butyl 5-amino-2-(6-methoxypyridin-3-yl)-5-oxopentanoate (**42**):

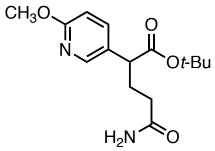

42

Yields: 46%, 48%, 52%. Enantiomeric ratio: 65:35.

HPLC method: CHIRALCEL® OX-3R column, 30% acetonitrile, 70% water; t_R1 = 7.30 min, t_R2 = 7.91 min.

enantioenriched *tert*-butyl 5-amino-2-(1-methyl-1*H*-indol-6-yl)-5-oxopentanoate (**49**):

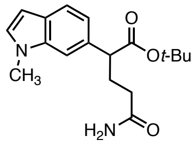

49

Yields: 70%, 72%, 77%. Enantiomeric ratio: 65:35.

Chiral HPLC method: CHIRALCEL® OX-3R column, 45% acetonitrile, 55% water; t_R1 = 6.85 min, t_R2 = 8.62 min.

enantioenriched *tert*-butyl 5-amino-2-(1-methyl-1*H*-indazol-6-yl)-5-oxopentanoate (**50**):

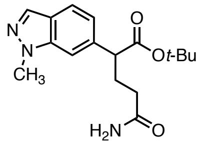

50

Yields: 42%, 43%, 42%. Enantiomeric ratio: 64:36.

Chiral HPLC method: CHIRALCEL® OX-3R column, 30% acetonitrile, 70% water; t_R1 = 12.35 min, t_R2 = 14.33 min.

product	er	product	er
**30**	62:38	**44**	50:50
**31**	61:39	**45**	60:40
**32**	67:33	**46**	61:39
**33**	50:50	**47**	53:47
**34**	53:47	**48**	58:42
**39**	99:1	**51**	52:48
**40**	n.d.[a]	**52**	52:48
**43**	52:48		

Table S17a. Enantiomeric ratios for the products **30**–**34**, **39**–**40**, **43**–**48**, and **51**–**52** obtained using Procedure B. [a]Enantiomeric ratios were not determined due to low yields.

	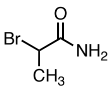 **S15a**	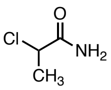 **S15b**	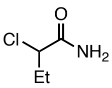 **S16**	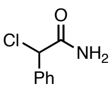 **S2b**	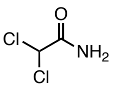 **S17**
Procedure B	69%<br>1:1 dr<br>93:7 er<br>90:10 er	44%<br>1:1 dr<br>95:5 er<br>92:8 er	32%<br>1.2:1 dr<br>70:30 er<br>63:37 er	n.d.	3%<br>1:1 dr<br>99:1 er<br>99:1 er

Table S17b. Summary of results using α-substituted carboxamide under Procedure B.

5.2 Procedures for the synthesis of 20 via preparative-scale photoenzymatic hydroalkylation reactions.

*Synthesis of **20** via preparative-scale photoenzymatic hydroalkylation reactions with purified enzyme.*

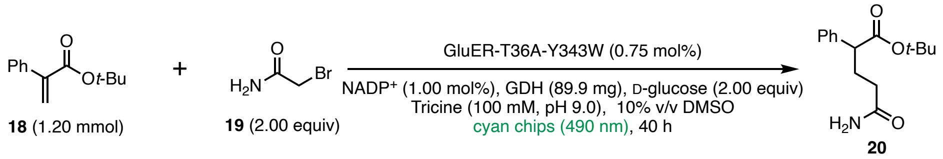

In the glovebox, 34.0 mL of Tricine buffer (100 mM, pH 9.0) was added to a 200 mL round-bottom flask with a magnetic stir bar containing 0.75 mol% of the aliquoted GluER HA_{rac} (900 µmol) to provide an orange solution. 6.00 mL of a "master mix" solution containing 2.00 equivalents of D-glucose, 89.9 mg GDH, and 2 mol% of $NADP^+$ in Tricine buffer (100 mM, pH 9.0) was added to the reaction mixture. After 5 min, the bromide **19** (331.2 mg, 2.00 equiv) was added as a 2.25 mL solution in dimethyl sulfoxide. Finally, the ester **18 (**244.8 mg 1.20 mmol, 1 equiv) was added as a 2.25 mL solution in dimethyl sulfoxide. The flask was sealed with a rubber septum and removed from the glovebox, then placed on a stir plate at 600 rpm in a water bath, and irradiated with two 490 nm cyan LED chips for 40 h. The reaction set up is shown in Photos S1–S3.

The product mixture was diluted with ethyl acetate (100 mL) and stirred vigorously for 1 h. The diluted product mixture was filtered through a pad of celite, and the residue was washed three times with ethyl acetate (50 mL). The filtrate was transferred to a separatory funnel, and the layers that formed were separated. The aqueous layer was extracted with ethyl acetate (5 × 100 mL). The organic layers were combined, and the combined organic layers were dried over sodium sulfate. The dried solution was filtered, and the filtrate was concentrated. The residue obtained was purified by flash-column chromatography (eluting with 50% ethyl acetate–hexanes) to provide the amide **20** as a white powder (262 mg, 83%).

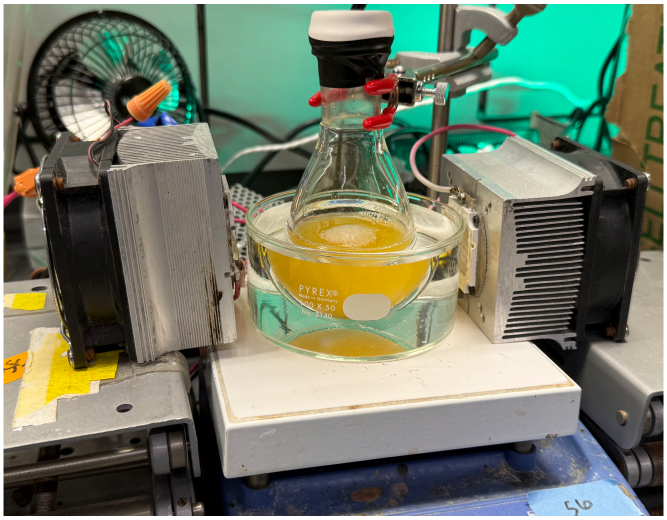

Photo S1. Reaction setup. Picture taken right before turning on the cyan LEDs.

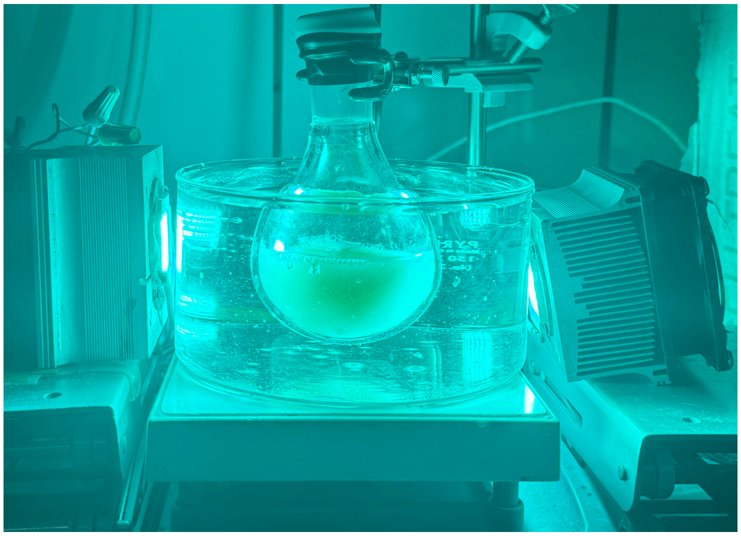

Photo S2. Reaction setup. Picture taken during the reaction, illuminated by cyan LEDs.

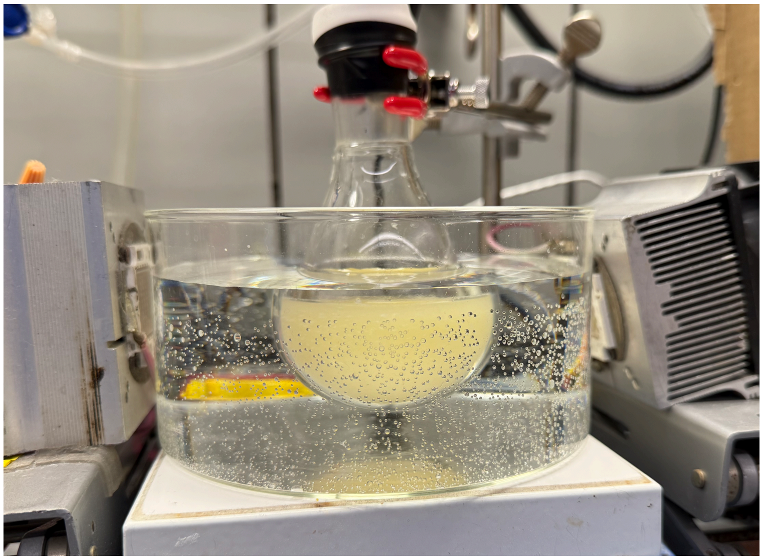

Photo S3. Reaction setup. Picture taken after completion.

*Synthesis of **20** via preparative-scale photoenzymatic hydroalkylation reactions with dialyzed cell-free lysate.*

Quantification of the dialyzed cell-free lysate.

Dialyzed cell-free lysate of GluER HA_{rac} was prepared and quantified according to the procedure detailed in Section 1.8. The gel image of the stain-free SDS-PAGE and the standard curve of purified GluER HA_{rac} are shown in Fig. S1.

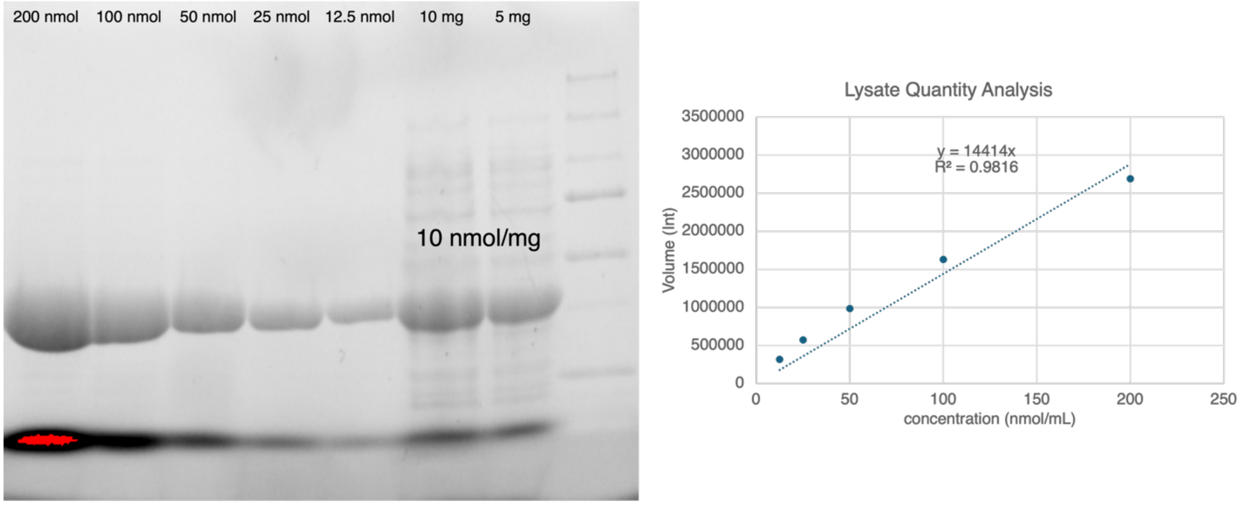

Figure S1. Stain-free SDS page gel and the standard curve of purified GluER HA_{rac}.

Preparative-scale photoenzymatic hydroalkylation reactions with dialyzed cell-free lysate.

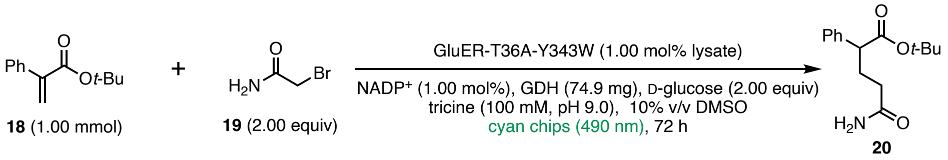

In the glovebox, 35.0 mL of Tricine buffer (100 mM, pH 9.0) was added to a 200 mL round-bottom flask with a magnetic stir bar containing 1.00 mol% of the GluER HA_{rac} dialyzed cell-free lysate (1.00 g) to provide an orange solution. 5.00 mL of a "master mix" solution containing 2.00 equivalents of D-glucose, 74.9 mg GDH, and 2 mol% of $NADP^+$ in Tricine buffer (100 mM, pH 9.0) was added to the reaction mixture. After 5 min, the bromide **19** (276.0 mg, 2.00 equiv) was added as a 2.25 mL solution in dimethyl sulfoxide. Finally, the ester **18** **(**204.0 mg 1.00 mmol, 1 equiv) was added as a 2.25 mL solution in dimethyl sulfoxide. The flask was sealed with a rubber septum and removed from the glovebox, then placed on a stir plate at 600 rpm in a water bath, and irradiated with two 490 nm cyan LED chips for 72 h. The reaction set up is shown in Photo S1–S3.

The product mixture was diluted with ethyl acetate (100 mL) and stirred vigorously for 1 h. The diluted product mixture was filtered through celite, and the residue was washed three times with ethyl acetate (50 mL). The filtrate was transferred to a separatory funnel, and the layers that formed were separated. The aqueous layer was extracted with ethyl acetate (5 × 100 mL). The organic layers were combined, and the combined organic layers were dried over sodium sulfate.

The dried solution was filtered, and the filtrate was concentrated. The residue obtained was purified by flash-column chromatography (eluting with 50% ethyl acetate–hexanes) to provide the amide **20** as a white powder (123 mg, 47%).

6. Determination of the absolute configuration of the amide 20.

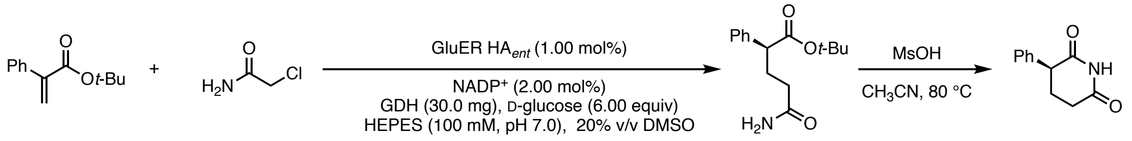

18 (400 µmol) **53** (4.00 equiv) **20** (93:7 er) **5** (93:7 er)

In the glovebox, 14.0mL of HEPES buffer (100 mM, pH 7.0) was added to a 100 mL round-bottom flask with a magnetic stir bar containing 1.00 mol% of purified GluER HA$_{ent}$ (4.00 µmol) to provide an orange solution. 2.00 mL of a "master mix" solution containing 6.00 equivalents of D-glucose, 30.0 mg GDH, and 2 mol% of $NADP^+$ in HEPES buffer (100 mM, pH 7.0) was added to the reaction mixture. After 5 min, the chloride **53** (149.6 mg, 4.00 equiv) was added as a 2.00 mL solution in dimethyl sulfoxide. Finally, the ester **18 (**81.6 mg, 400 µmol, 1 equiv) was added as a 2.00 mL solution in dimethyl sulfoxide. The flask was sealed with a rubber septum and removed from the glovebox, then placed on a stir plate at 600 rpm in a water bath, and irradiated with two 490 nm cyan LED chips for 72 h.

The product mixture was diluted with ethyl acetate (50 mL) and stirred vigorously for 1 h. The diluted product mixture was filtered through celite, and the residue was washed three times with ethyl acetate (20 mL). The filtrate was transferred to a separatory funnel, and the layers that formed were separated. The aqueous layer was extracted with ethyl acetate (5 × 20 mL). The organic layers were combined, and the combined organic layers were dried over sodium sulfate. The dried solution was filtered, and the filtrate was concentrated. The residue obtained was purified by flash-column chromatography (eluting with 50% ethyl acetate–hexanes) to provide the amide **20** admixed with the chloride **53**. The mixture was not further separated and was used directly in the next step. Analysis of the reaction mixture showed that **20** was formed with 93:7 er.

Methanesulfonic acid (130 µL, 2.00 µmol, 5.00 equiv) was added to a solution of the amide **20** (nominally 400 µmol, 1 equiv) in acetonitrile (1.20 mL) at 23 °C. The reaction mixture was stirred for 3 h at 80 °C. The product mixture was cooled to 23 °C over 10 min and diluted sequentially with ethyl acetate (5 mL) and saturated aqueous sodium bicarbonate solution (5 mL). The diluted product mixture was transferred to a separatory funnel, and the layers that formed were separated. The aqueous layer was extracted with ethyl acetate (3 × 20 mL). The organic layers were combined, and the combined organic layers were dried over sodium sulfate. The dried solution was filtered, and the filtrate was concentrated. The residue obtained was purified by flash-column chromatography (eluting with 50% ethyl acetate–hexanes) to provide the enantioenriched glutarimide **5** as a white powder (21.0 mg, 28% over two steps). Chiral HPLC analysis of the purified product showed that **5** was formed with 93:7 er. Chiral HPLC method: CHIRALPAK® IH column, 20% *iso*-propanol, 80% hexane; t_R1 = 27.04 min, t_R2 = 33.98 min

Optical rotation values were obtained for the enantioenriched **5**. Comparison with values reported by Reisman and co-workers[14] assigned enantio-enriched **5** as the (*S*)-enantiomer (Table S18).

	experimental	literature[14]
er	93:7	97:3
$[\alpha]_D^{22}$	–3.16 (c = 1.0, $CHCl_3$)	–4.44 (c = 1.0, $CHCl_3$)

Table S18. Experimental and literature-reported optical rotation values of the enatio-enriched **5**.

7. Racemization studies of the amide 20.

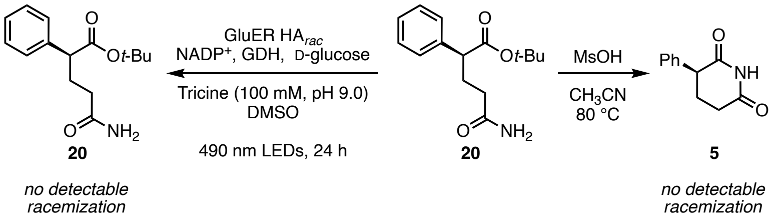

A small amount of racemic **20** (chiral HPLC trace shown in Fig. S2a) was subjected to preparative chiral HPLC purification, and the enantiomers were partially separated (chiral HPLC traces shown in Fig. S2b). 2.00 mg of enantiomerically pure **20** was added to a reaction mixture of 200 nmol GluER HA$_{rac}$, 1.50 mg GDH, 0.30 mg NADP$^+$, and 21.6 mg D-glucose dissolved in 666 µL Tricine buffer (pH 9.0, 100 mM) and dimethyl sulfoxide (74 µL). The mixture was irradiated with 490 nm cyan chips for 24h while stirring. Chiral HPLC analysis of the mixture after irradiation showed no racemization of the enantiomerically pure **20** (chiral HPLC traces shown in Fig. S2c). 2.00 mg of enantiomerically pure **20** was converted to **5** following procedures detailed in Section 4.3. Chiral HPLC analysis showed that **5** so obtained remained enantiomerically pure (Fig. S2d and Fig. S2e), indicating that there was no racemization of **20** under the acidic cyclization conditions.

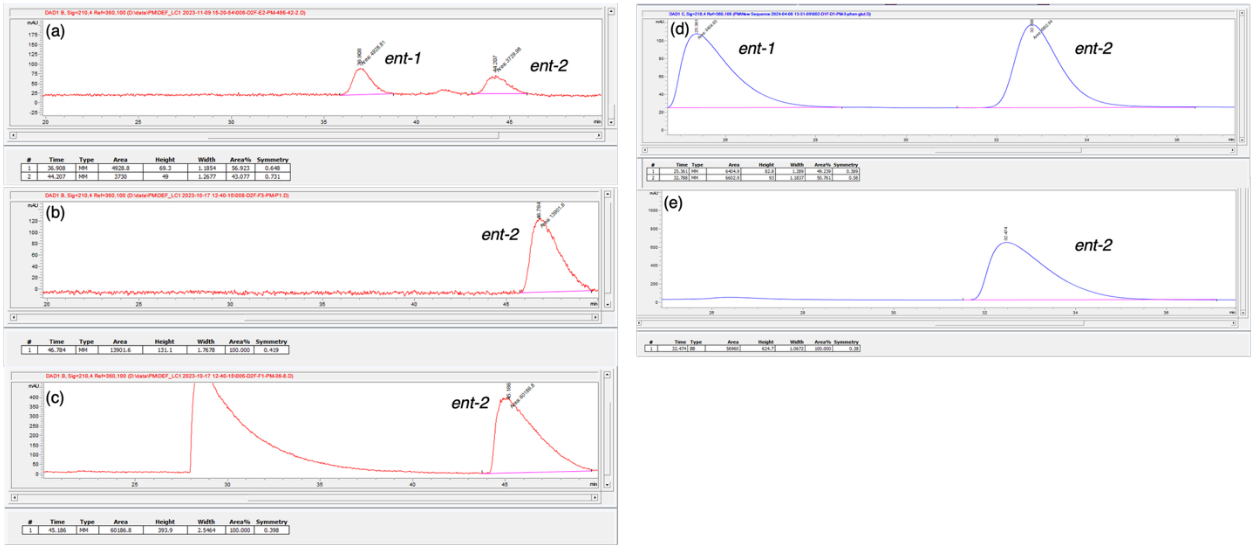

Figure S2. (a) Chiral HPLC trace of racemic **20**. CHIRALPAK® OJ column, 3% *iso*-propanol, 97% hexane; $t_R1 = 36.91$ min, $t_R2 = 44.21$ min. (b) Chiral HPLC trace of the enantiopure **20** after separation. CHIRALPAK® OJ column, 3% *iso*-propanol, 97% hexane; $t_R2 = 44.78$ min. (c) Chiral HPLC trace of the pH 9.0 mixture containing enantiopure **20**. CHIRALPAK® OJ column, 3% *iso*-propanol, 97% hexane; $t_R2 = 44.19$ min. (d) Chiral HPLC trace of racemic **5**. CHIRALPAK® IH column, 20% *iso*-propanol, 80% hexane; $t_R1 = 25.36$ min, $t_R2 = 32.79$ min. (e) Chiral HPLC trace of **5** synthesized from enantiopure **20**. CHIRALPAK® IH column, 20% *iso*-propanol, 80% hexane; $t_R2 = 32.47$ min.

8. Mechanistic studies.

8.1 Control experiments.

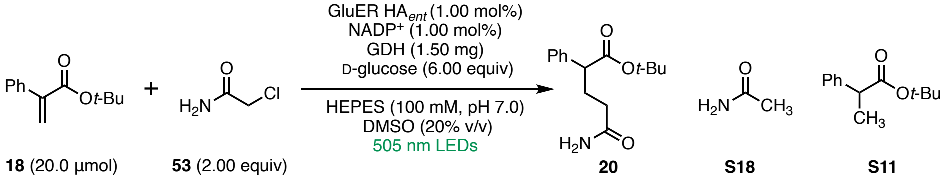

entry	deviation from standard conditions	result
1	no enzyme	**20** not detected by LC-MS analysis
2	no turnover mix	~1% **20** by LC-MS analysis
3	5% FMN instead of enzyme	0.2% **20** by LC-MS analysis
4	no **18**	1.5% **S18** by NMR analysis
5	no **53**	**S11** not detected by NMR or LC-MS analysis
6	no light	**20** or **S11** not detected by NMR or LC-MS analysis
7	no light, no **18**	**S18** not detected by NMR or LC-MS analysis
8	no light, no **53**	**S11** not detected by NMR or LC-MS analysis

Table S19. Tabulated results of the control experiments. The standard conditions are detailed in Section 5.1, Procedure B.

8.2 Site-directed mutagenesis studies.

GluER-T36A-Y177F-M102G-M105V-A44E-W66M-Y343F was generated following procedures detailed in Section 3.4, and was used in the photoenzymatic hydroalkylation following Procedure B. Tabulated reaction yields and enantiomeric ratios with this mutant and related enzymes are shown in Table S20.

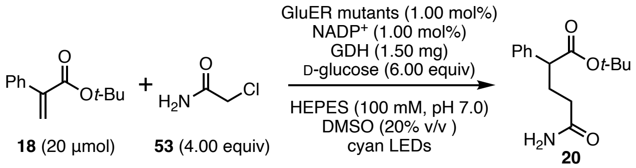

mutant	yield	er
GluER-T36A	24%	57:43
GluER-T36A-Y343W	42%	50:50
GluER-T36A-Y177F-M102G-M105V-A44E-W66M	84%	93:7
GluER-T36A-Y177F-M102G-M105V-A44E-W66M-Y343W	54%	67:33

Table S20. Tabulated yields and enantiomeric ratios of reactions with selected GluER mutants.

8.3 Deuterium incorporation studies.

Deuterium incorporation studies at pH 9

Two series of isotopic labeling experiments were conducted at 1.00 mol% GluER loading on a 20-µmol scale with the ester **18** and the chloride **53**. Reactions were conducted at pH 9.0 to suppress solvent exchange of the flavin N5–H(D).[15] Each series comprised nine independent experiments, each carried out with either a GluER mutant identified during the engineering campaign, GluER HA$_{rac}$, or GluER HA$_{ent}$-Y343F (see below).

The two reaction series were performed simultaneously for 48 h. One series containing 6.00 equivalents of D-glucose-1-d_1 in 100 mM Tris buffer(pH = 9.0) prepared in H_2O, while the other containing non-deuterated D-glucose at the same loading in an identical buffer prepared in D_2O. Each reaction was conducted in duplicate. Upon completion of the reaction, reaction mixtures were processed for LC-MS and NMR analyses as described in Section 5.1. Yields and enantiomeric ratios were determined accordingly.

The extent of deuterium incorporation was determined by quantitative ^{13}C NMR spectroscopy. ^{13}C signals corresponding to C4 (glutarimide numbering) in the deuterated and non-deuterated **20** were identified and integrated using MestReNova (version 15.1.0) employing the fit region tool (lower and upper boundaries set to 0.1 and 100 Hz; simulated annealing enabled; coarse iterations = 500, fine iterations = 200). Deuterium incorporation was calculated using Equation S1. Representative ^{1}H and ^{13}C NMR spectra are shown in Figs. S4–S6, and tabulated deuterium incorporation data are shown in Table S21.

In a control experiment (Fig. S3), non-deuterated compound **20** was resubjected to the reaction conditions at pH 9.0, and no deuterium incorporation was detected. This result suggests that deuterium incorporation occurs during the process of product formation rather than through post-reaction exchange.

At pH 9.0, no deuterium incorporation was observed in Series A, whereas Series B consistently exhibited ≥46% deuterium incorporation. These results indicate that the C3–H(D) proton in **20** is not derived from the flavin N5–H(D).

Equation S1: $$\%\boldsymbol{D}_{\mathbf{Inc.}} = \left[1 - \left(\frac{A_1}{A_1 + A_2}\right)\right] \times 100$$

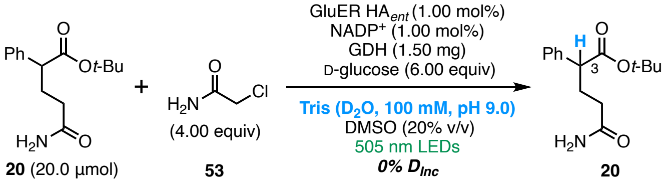

Figure S3. A control experiment that ruled out potential solvent exchange of C3–H in **20** at pH 9.0.

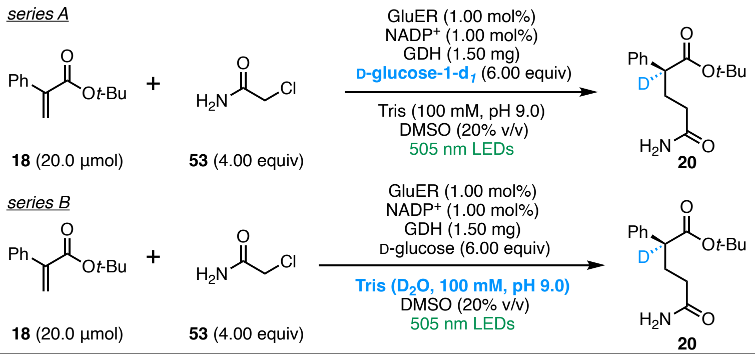

GluER mutant	series A			series B		
	%D_{Inc}	yield	er	%D_{Inc}	yield	er
T36A	0	44%	55:45	46.3	50%	53:47
T36A-Y177F	0	47%	57:43	63.4	62%	54:46
T36A-Y177F-W66T	0	45%	54:46	55.5	47%	53:47
T36A-Y177F-W66T-M102G	0	47%	55:45	59.1	54%	54:46
T36A-Y177F-W66T-M102G-M105V	0	77%	61:39	49.4	61%	63:37
T36A-Y177F-W66T-M102G-M105V-A44E	0	56%	58:42	56.1	63%	56:44
T36A-Y177F-M102G-M105V-A44E-W66M	0	62%	61:39	75.2	72%	58:42
T36A-Y343W	0	48%	50:50	55.2	55%	50:50
T36A-Y177F-M102G-M105V-A44E-W66M-Y343F	0	64%	58:42	67.5	67%	58:42

Table S21. Tabulated %D_{Inc}, yield, and er data for deuterium incorporation studies at pH 9.0.

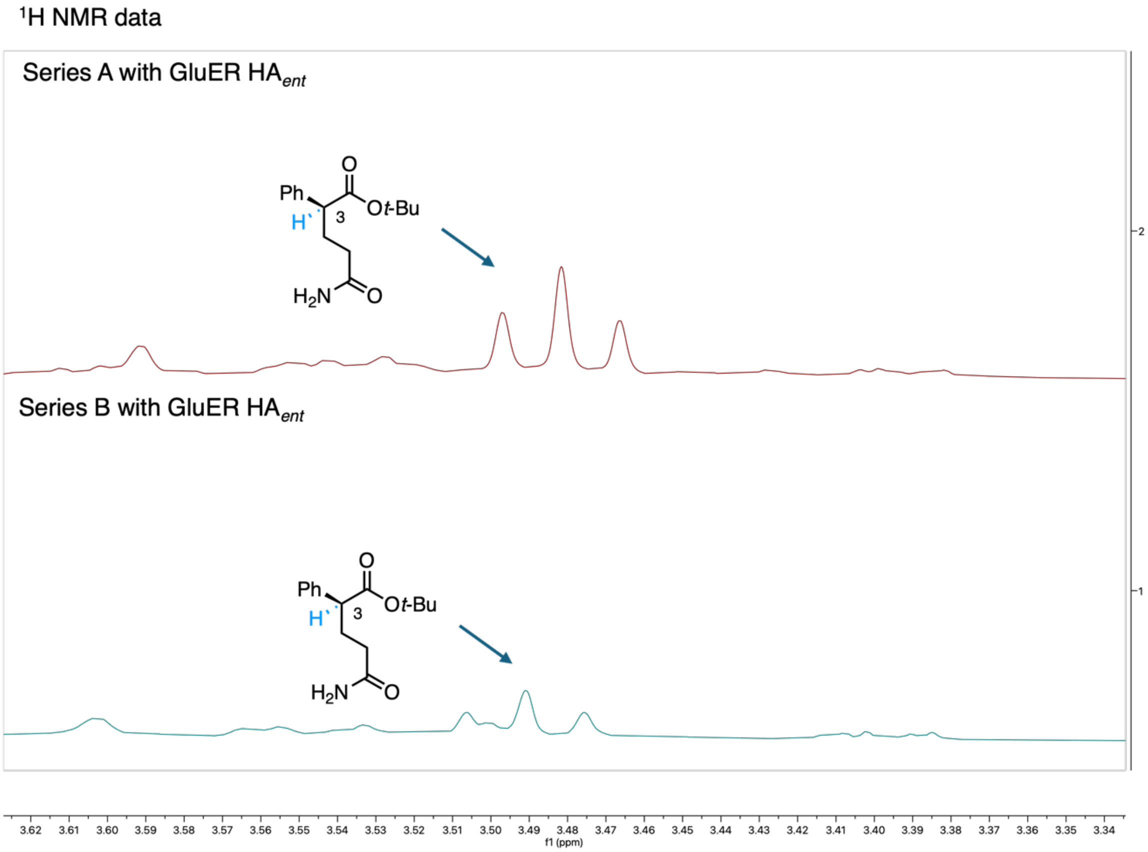

Figure S4. Representative ^{1}H NMR spectra with GluER HA$_{ent}$.

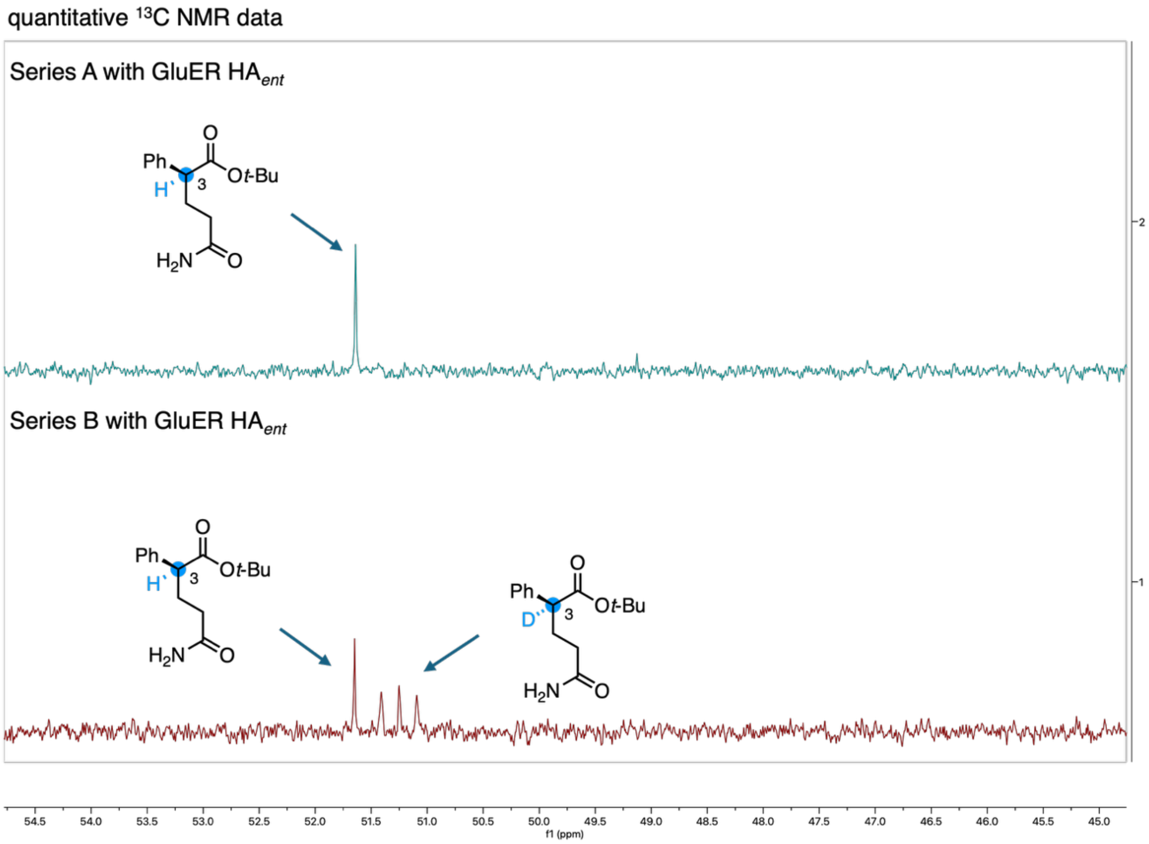

Figure S5. Representative quantitative ^{13}C NMR spectra with GluER HA*ent*.

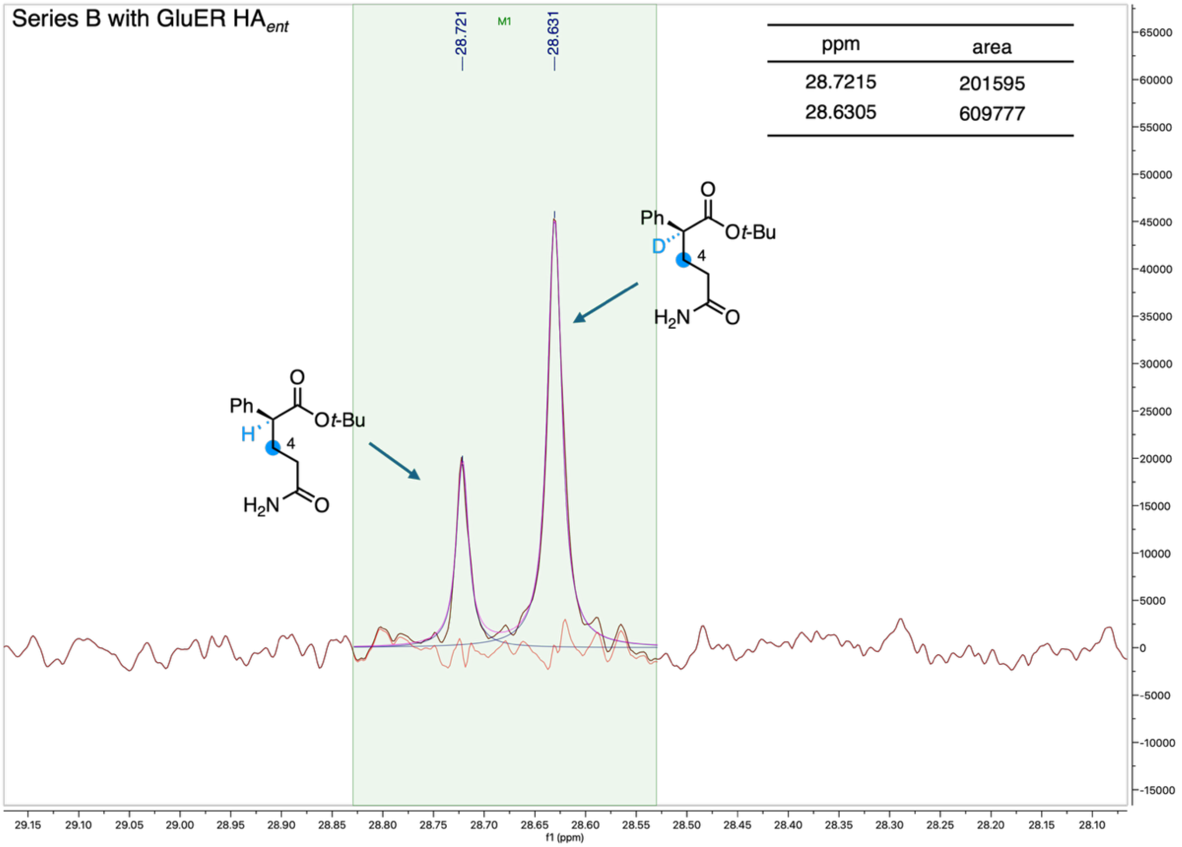

Figure S6. Representative quantitative ^{13}C NMR spectra with GluER HA$_{ent}$.

Deuterium incorporation studies at pH 7

Because the optimized conditions are conducted at pH 7.0 (see Procedure B), deuterium incorporation studies were performed using D-glucose-1-d_1 in 100 mM HEPES buffer (pH=7.0) prepared in H_2O, and non-deuterated D-glucose at the same loading in an identical buffer prepared in D_2O, following the same procedures as at pH 9. Given the rapid solvent exchange of the flavin N5–H(D) at pH 7.0,[15] conclusions drawn from these experiments are **necessarily tentative**. Tabulated deuterium incorporation data are provided in Table S22.

At pH 7.0, increased deuterium incorporation was observed in Series A relative to experiments conducted at pH 9.0. Because we previously established that C3–H in **20** is not directly derived from flavin N5–H, we attribute this increase to the solvent exchange of flavin N5–D within the active site. Consistent with our proposed mechanism, we tentatively hypothesize that, in Series A, flavin N5–D partially regenerates the deprotonated phenolic oxygen of Y343 at the end of a catalytic cycle (Fig. S7). This deuterium atom is subsequently incorporated into **20** in the next catalytic turnover. This is supported by the 0% deuterium incorporation observed for GluER HA_{rac} and GluER HA_{ent}-Y343F, which lack the tyrosine residue at position 343.

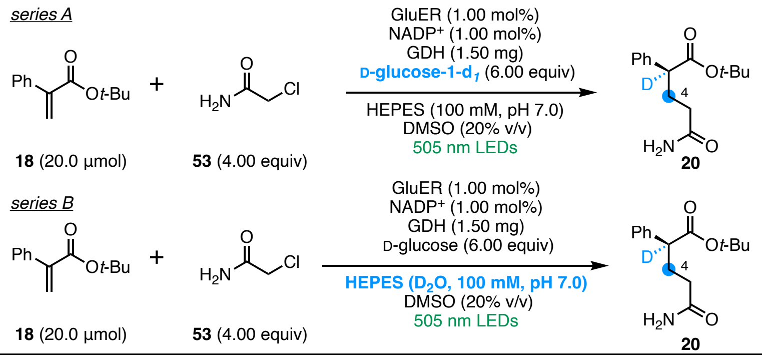

GluER mutant	series A			series B		
	$\%D_{Inc}$	yield	er	$\%D_{Inc}$	yield	er
T36A	0.0	30%	57:43	71.4[a]	5%	53:47
T36A-Y177F	18.7	54%	81:19	19.4	10%	83:17
T36A-Y177F-W66T	29.5	68%	68:32	60.9	58%	85:15
T36A-Y177F-W66T-M102G	33.0	71%	76:24	51.3	54%	82:18
T36A-Y177F-W66T-M102G-M105V	30.6	74%	77:23	55.4	54%	86:14
T36A-Y177F-W66T-M102G-M105V-A44E	37.7	74%	78:22	48.7	65%	88:12
T36A-Y177F-M102G-M105V-A44E-W66M	33.8	89%	86:14	28.0	70%	94:6
T36A-Y343W	0.0	28%	50:50	59.8	51%	50:50
T36A-Y177F-M102G-M105V-A44E-W66M-Y343F	0.0	26%	54:46	50.3	61%	70:30

Table S22. Tabulated $\%D_{Inc}$, yield, and er data for deuterium incorporation studies at pH 7.0. [a]Determined by LC-MS due to low yield.

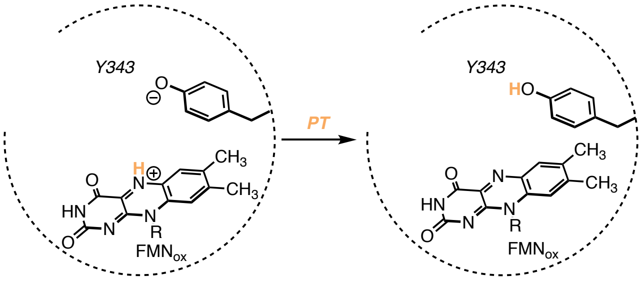

Figure S7. A possible proton transfer between FMN_{ox} and Y343 to regenerate the phenolic alcohol of Y343.

*Comparative deuterium incorporation analysis of reactions with **54** and **18**.*

Two parallel isotopic labeling experiments were conducted at 1.00 mol% GluER-T36A loading on a 20-μmol scale with the styrene **54** and the ester **18** (Fig. S8). The reaction conditions were modified from the report by Hyster and co-workers,[16] and 2-chloro-*N*,*N*-dimethylacetamide (**S19**) was used to allow for comparable reactivities with the two substrates.[16] The reactions were performed simultaneously for 24 h and were both conducted with 6.00 equivalents of D-glucose-1-d_1 in 100 mM Tris buffer (pH=9.0) prepared in H_2O. Upon completion of the reaction, reaction mixtures were processed for NMR analyses. ^{1}H and ^{13}C NMR spectra are shown in Figs. S9–S11, and tabulated deuterium incorporation data are discussed below.

Under identical conditions, reaction with **54** afforded the product **56** with 82% deuterium incorporation, while reaction with **18** afforded **58** with no deuterium incorporation. These results suggested that **18** is responsible for the mechanistic difference.

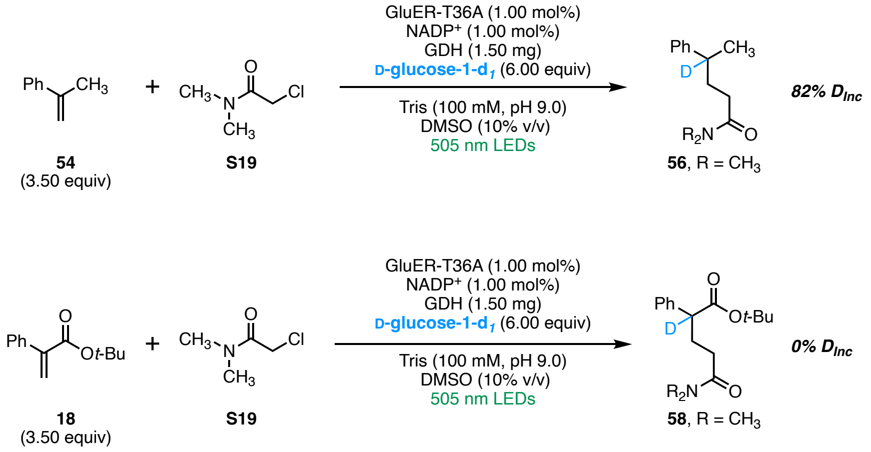

Figure S8. Comparative deuterium incorporation analysis of reactions with **54** and **18**.

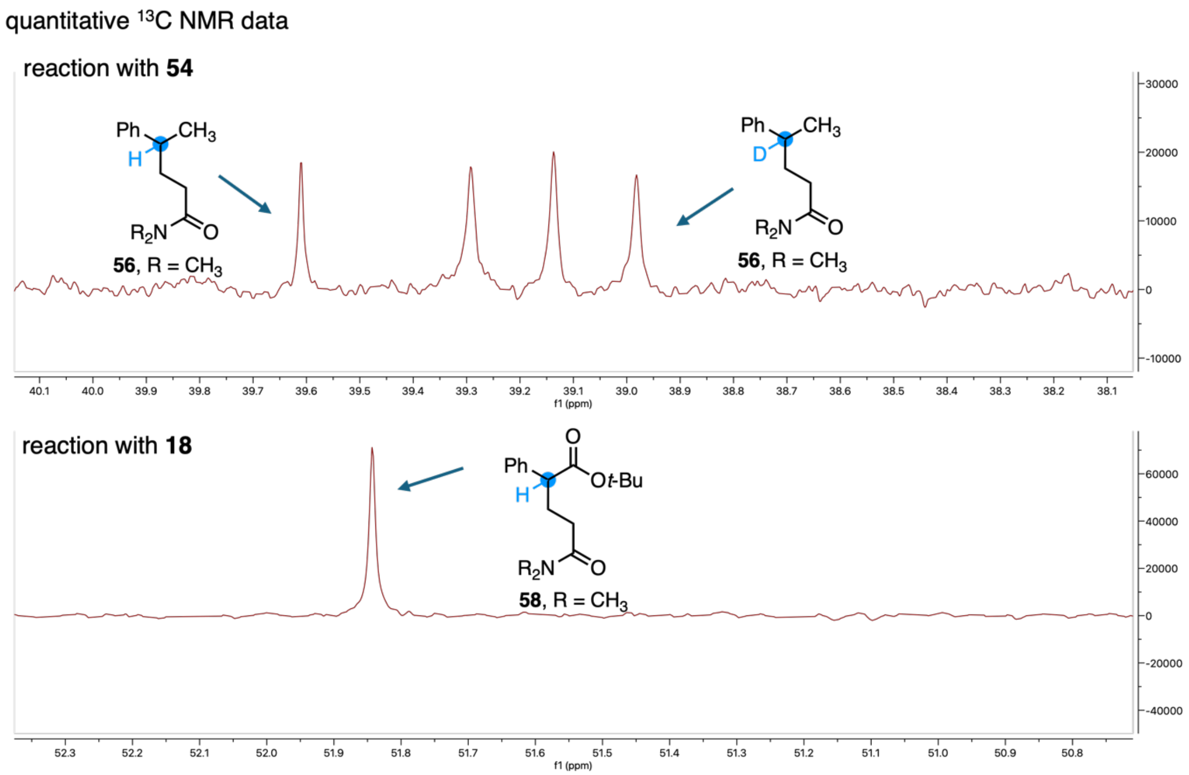

Figure S9. Quantitative ^{13}C NMR spectra for reactions with **54** and **18**.

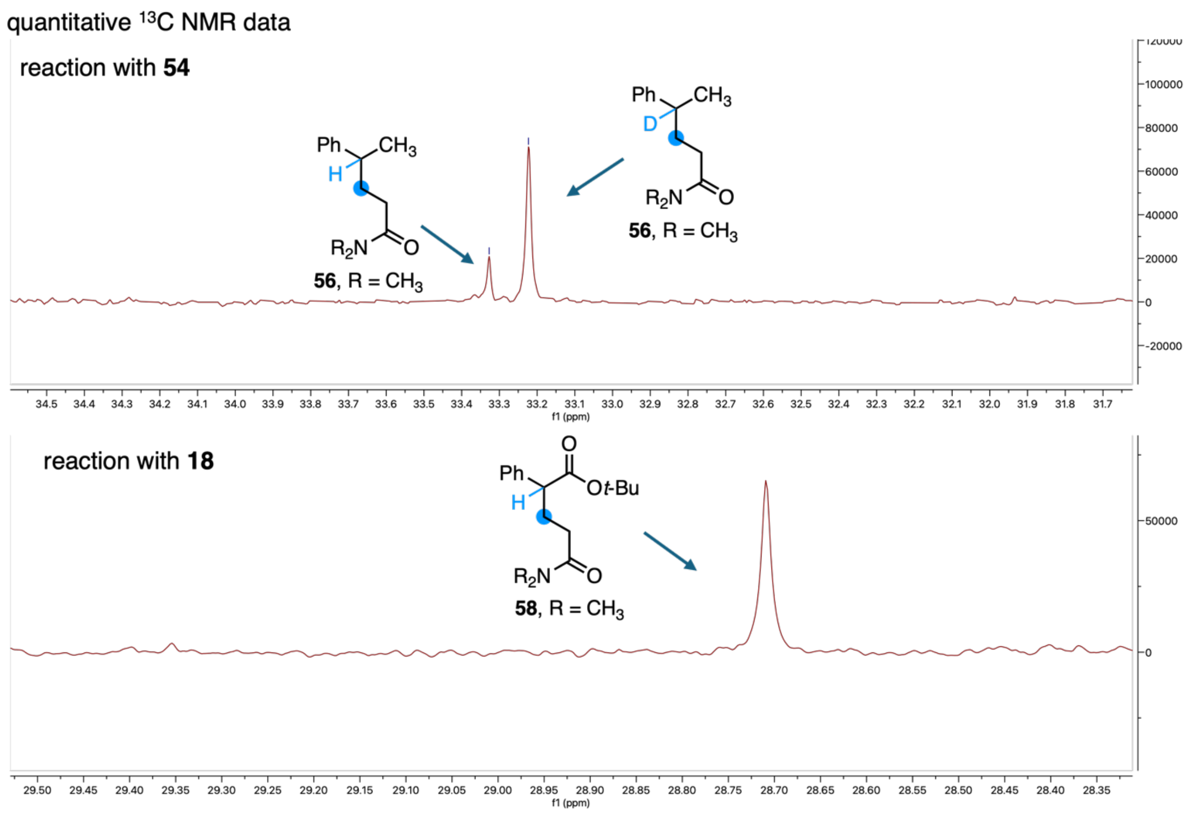

Figure S10. Quantitative ^{13}C NMR spectra for reactions with **54** and **18**.

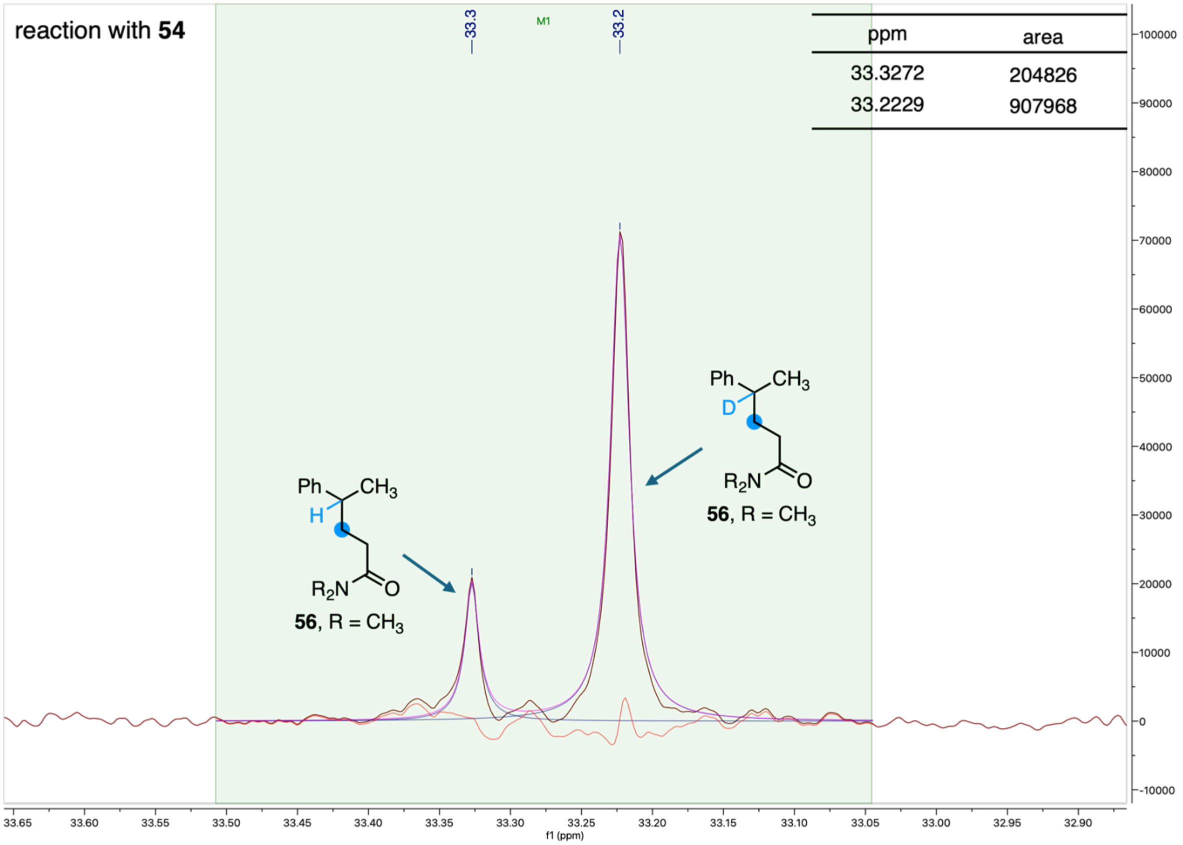

Figure S11. Quantitative ^{13}C NMR spectra for reactions with **54**.

8.4 Studies of the correlation between buffer pH and enantioselectivity.

Seven series of experiments were conducted at 1.00 mol % GluER catalyst loading on a 20 µmol scale using the ester **18** and the chloride **53** with selected GluER variants following Section 5.1 Procedure B. Each series comprised three independent experiments conducted at pH 9.0, 8.0, and 7.0 in 100 mM Tris buffer, otherwise following Procedure B. Enantiomeric ratios are tabulated in Table S23, and a corresponding line graph is shown in Fig. S12.

Among all GluER variants examined, only the two mutants lacking Y343 (GluER HA_{rac} and GluER HA_{ent}-Y343F) showed no correlation between buffer pH and enantioselectivity.

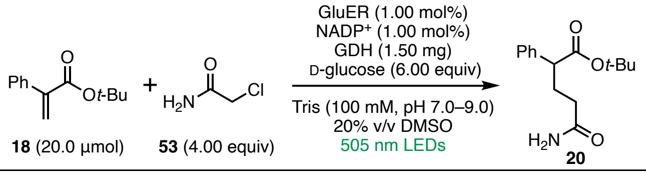

GluER variants	enantiomeric ratio		
	pH 7.0	pH 8.0	pH 9.0
T36A	59:41	54:46	52:48
T36A-Y177F	60:40	55:45	53:47
T36A-Y177F-M102G-M105V-A44E-W66M	87:13	66:34	63:37
T36A-Y343W	50:50	50:50	50:50
T36A-Y177F-M102G-M105V-A44E-W66M-Y343F	58:42	58:42	58:42
T36A-Y177F-W66T-M102G-M105V	79:21	68:32	62:38
T36A-Y177F-W66T-M102G-M105V-A44E	81:19	64:36	61:39

Table S23. Tabulated enantiomeric ratio of reactions with selected GluER variants at different pH values.

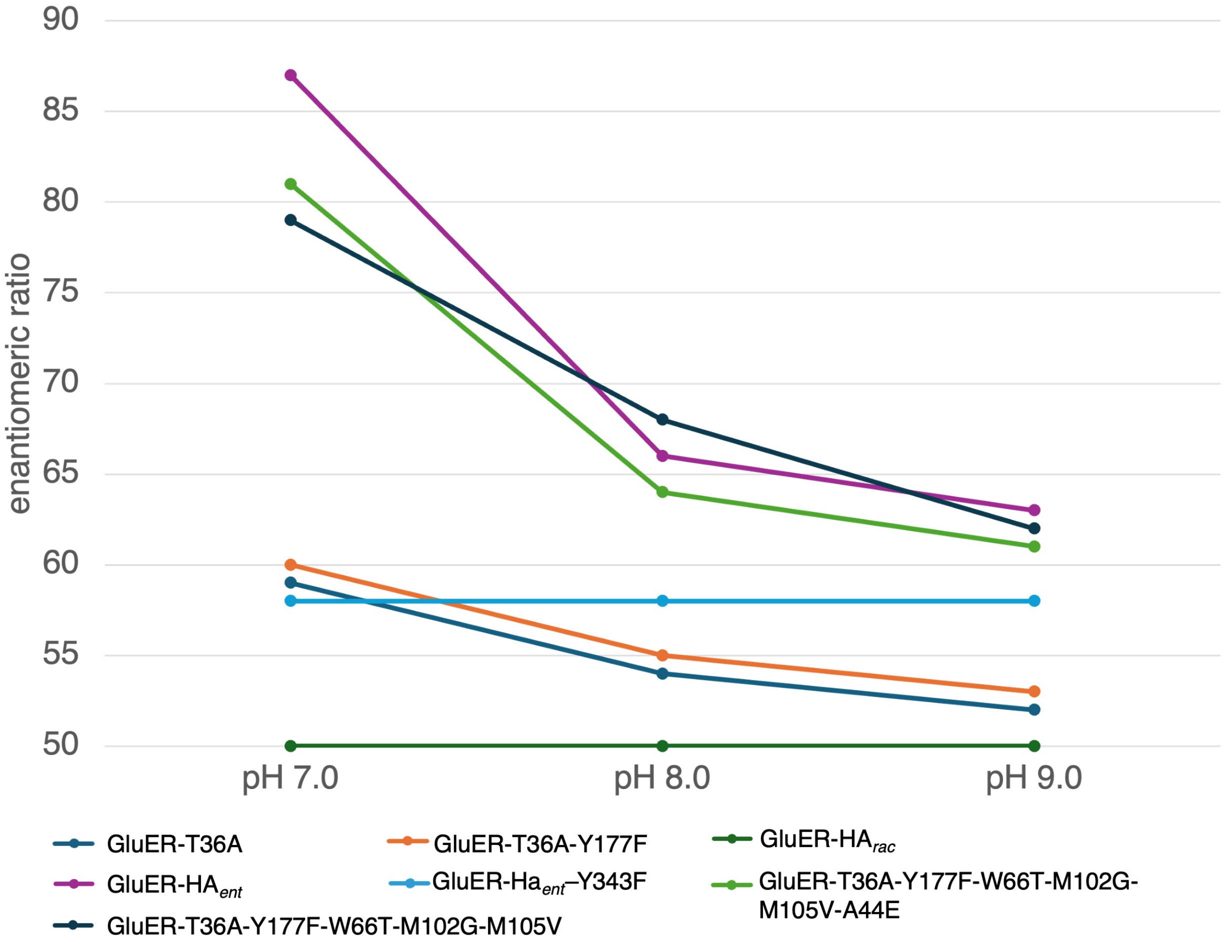

Figure S12. Line graph depicting enantiomeric ratio of reactions with selected GluER variants at different pH values.

8.5 Studies of the correlation between electronic properties of the substrates and enantioselectivity.

Enantioselectivity for *ortho*-substituted phenyl acrylates **21** and **24–28** were converted to the corresponding absolute $\Delta\Delta G^{\neq}$ values using Equation **S2**. These energy values were then correlated with Hammett substituent constants[17] to evaluate the relationship between electronic properties of the substrates and enantioselectivity. Because Hammett parameters are not formally defined for *ortho* substitution, σ values for the corresponding *meta*- and *para*-substituents were used. Notably, *para*-substituent constants are generally considered to more reliably reflect intrinsic electronic effects.

The resulting data are summarized in Table S24 and plotted in Figure S13 with linear regression trendlines and associated R^2 values shown. A strong linear correlation was observed using *para*-substituent Hammett parameters ($R^2 = 0.8309$), whereas a moderate correlation was obtained using *meta*-substituent parameters ($R^2 = 0.6457$).

Equation S2: $\Delta\Delta G^{\neq} = -0.592\ \ln(er)\ kcal \cdot mol^{-1}$

product	substituent	σ *para*	σ *meta*	er	$\lvert\Delta\Delta G^{\neq}\rvert$ (kcal mol^{-1})
**21**	*o*-CH_3	–0.17	–0.07	80:20	0.821
**24**	*o*-OCH_3	–0.27	0.12	87:13	1.125
**25**	*o*-Cl	0.23	0.37	78:22	0.749
**26**	*o*-Br	0.23	0.39	72:28	0.559
**27**	*o*-I	0.18	0.35	70:30	0.501
**28**	*o*-NO_2	0.78	0.71	61:39	0.265

Table S24. Tabulated data for studies of the correlation between electronic properties of the substrates and enantioselectivity.

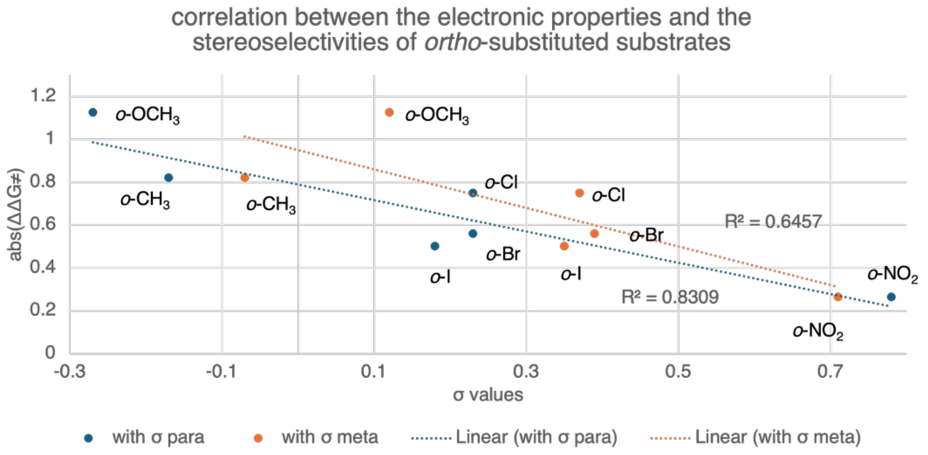

Figure S13. Scatter plot and linear trendlines depicting the enantiomeric ratio of reactions with correlation to Hammett substitution constants.

8.6 UV–Vis studies.

All samples were prepared in an MBraun® Labstar^{Pro} (working O2 level < 1.0 ppm, glovebox hereafter). Custom-designed quartz cuvettes were used to preserve anaerobic conditions throughout the experiments. Spectroscopic measurements were collected on a HORIBA Duetta fluorescence and absorption spectrometer housed inside the glovebox.

A blank spectrum was obtained using degassed HEPES buffer (100 mM, pH 7.0). A 200 µM enzyme solution was prepared by dissolving GluER HA$_{ent}$ (200 nmol, 1.00 equiv) in degassed HEPES buffer (100 mM, pH 7.0; total volume = 1.00 mL), and a spectrum of the oxidized FMN cofactor was recorded. Reduction of the FMN cofactor was achieved by the addition of sodium dithionite (30.0 µL of a 20.0 mM solution in HEPES buffer, pH 7.0; 600 nmol, 3.00 equiv), after which a spectrum of the reduced FMN cofactor was recorded.

Substrate solutions of ester **18** (200 equiv) or chloride **53** (800 equiv) prepared in degassed dimethyl sulfoxide (50.0 µL) were then added to the reduced GluER HA$_{ent}$ solution and passed through a syringe filter prior to spectral acquisition. Spectra of reduced GluER HA$_{ent}$ in the presence of each substrate were recorded, and data are shown in Fig. S13. Next, four parallel experiments were conducted using the same protocol, with spectra recorded at 0 min, 30 min, 1h, and 2 h following the addition of sodium dithionite, **18**, **53**, or **18** and **53** together. The corresponding spectra are shown in Fig. S15. Finally, the above procedure was repeated with **33-s** in place of **18**, and the spectrum was recorded at 0 min, 15 min, and 30 min following the addition of sodium dithionite and **33-s (**Fig. S16).

In the UV–Vis spectra collected in the first experiment, no characteristic spectral features associated with an enzyme-templated charge-transfer complex were observed (Fig. S14).[18] In the second experiment, slow oxidation of flavin hydroquinone (FMN$_{hq}$) was detected over approximately 2 h in the presence of both reactants (Fig. S15). A comparable rate of flavin oxidation was observed when only **18** was present, suggesting that this process does not require the haloamide coupling partner. In the third experiment, a substantially more rapid oxidation of FMN$_{hq}$ was observed when methyl acrylate **33-s** was used in place of **18** (Fig.S16). Collectively, these results suggest that the absence of observable charge-transfer features in the first experiment arises from the resting-state reactivity of the reduced enzyme in the dark, which slowly reduces **18** via the native ERED pathway. Consistent with this interpretation, the use of **18** markedly suppresses this background reduction relative to the less sterically hindered methyl acrylate **33-s**.

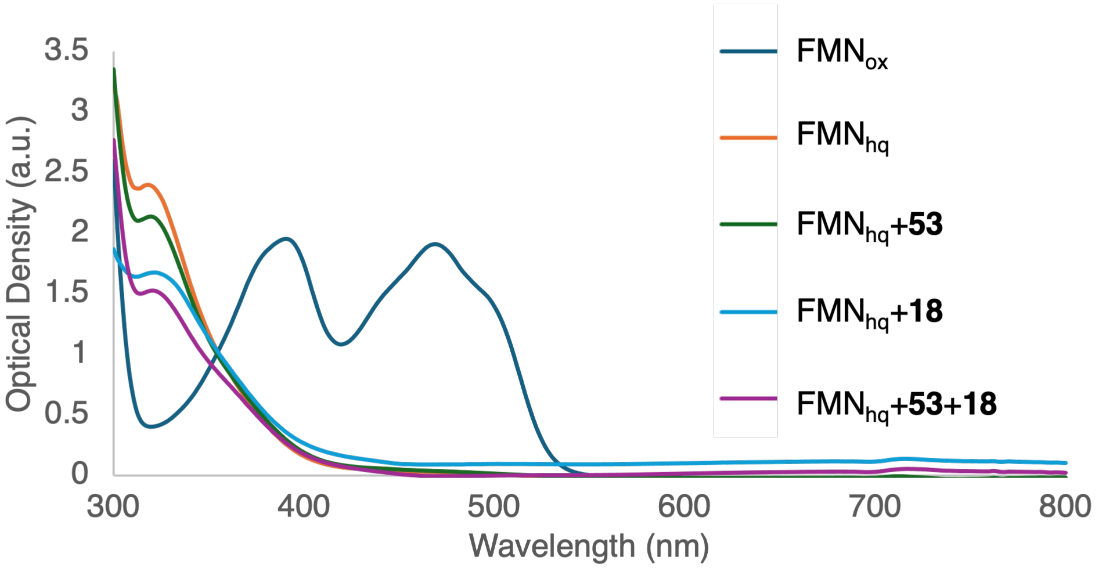

Figure S14. UV–Vis spectra of GluER HA*ent* (FMN_{ox}), GluER HA*ent* (FMN_{hq}), GluER HA*ent* (FMN_{hq}) with **53**, GluER HA*ent* (FMN_{hq}) with **18**, and GluER HA*ent* (FMN_{hq}) with **18** and **53** together.

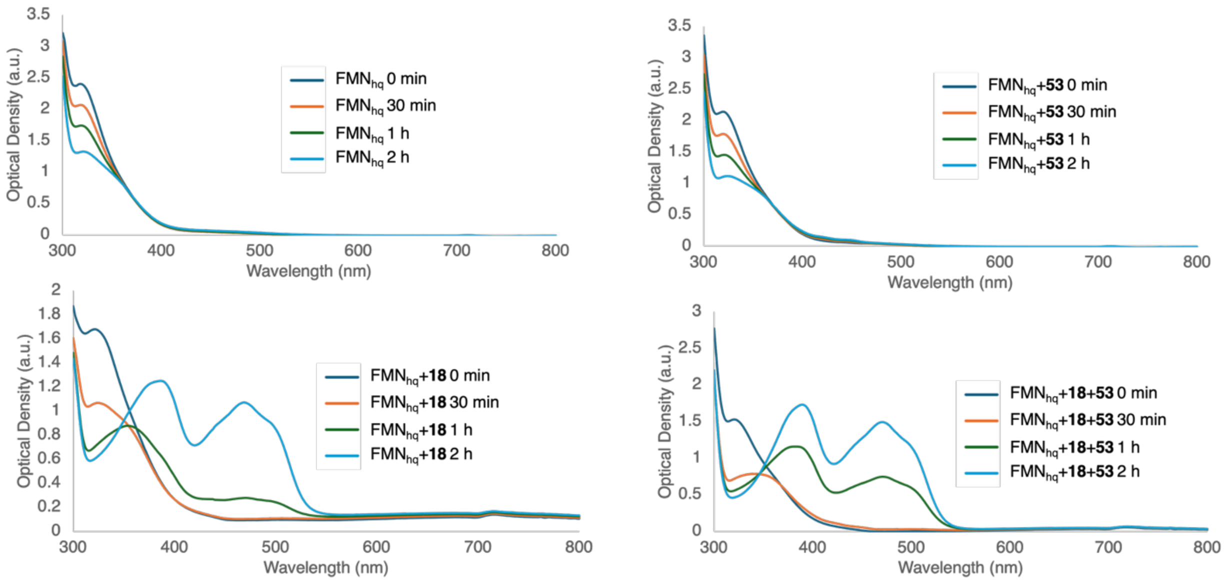

Figure S15. UV–Vis spectra of GluER HA$_{ent}$ (FMN$_{hq}$), GluER HA$_{ent}$ (FMN$_{hq}$) with **53**, GluER HA$_{ent}$ (FMN$_{hq}$) with **18**, and GluER HA$_{ent}$ (FMN$_{hq}$) with **18** and **53** together measured across four time points over 2h.

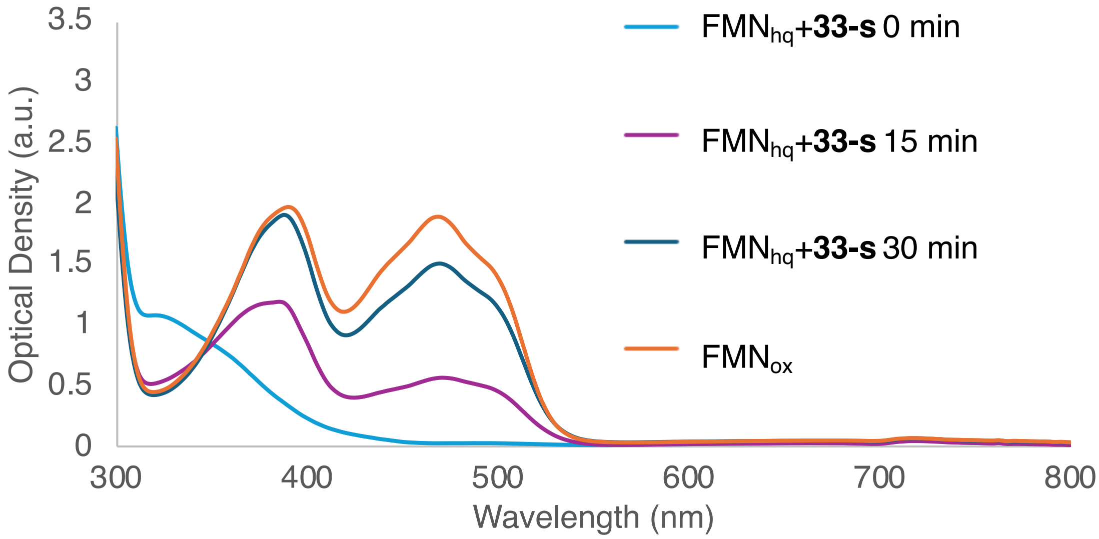

Figure S16. UV–Vis spectra of GluER HA*ent* (FMN_{hq}) with **33-s** measured across three time points over 30 min. The spectrum of GluER HA*ent* (FMN_{ox}) was incorporated as a reference.

9. Computational studies.

9.1 Calculation of pKa values.

Computational method.

Microscopic pK_a values were computed using the Rowan pK_a prediction workflow[19] (Rowan Scientific, https://www.rowansci.com).

Calculations were performed in microscopic mode, wherein individual ionizable sites on each molecule are evaluated separately. Geometry generation and conformational sampling were carried out using the ETKDG algorithm [20] followed by force-field pre-optimization. Low-energy conformers were further refined using the semiempirical method GFN2-xTB[21] before final energy evaluation. Single-point energies and geometry optimizations were performed using the AIMNet2 [22]machine-learned potential. Solvation free energies were obtained from GFN2-xTB calculations using the CPCM-X[23] implicit solvation model, with water used as the solvent. For each ionizable site, conjugate acid–base pairs were enumerated automatically, and Boltzmann-weighted free energies were computed across all relevant conformers. Microscopic pK_a values were then derived from calculated free-energy differences using Rowan's site-specific calibration protocol, which incorporates atom- and valence-dependent corrections.

Calculations were performed using Rowan Scientific's web platform (accessed 2025-11-21), and the results are shown in Table S25. We acknowledge Rowan Scientific for providing the pK_a prediction workflow used in this study.

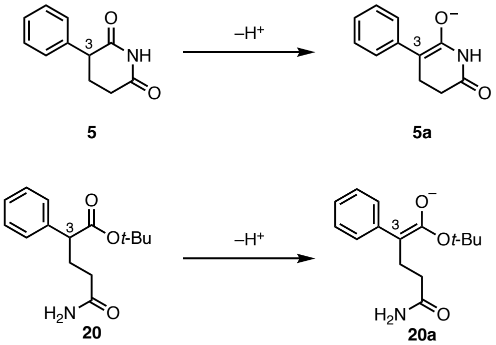

compound	site label	atom	calculated microscopic pKa (in H_2O)
**5**	3	C	14.90
**20**	3	C	19.24

Table S25. Predicted microscopic pK_a values for **5** and **20** in water.

9.2 Calculation of redox potential values.

Computational method.

Redox potentials were calculated using the Rowan redox-potential prediction workflow (Rowan Scientific, https://www.rowansci.com).

Calculations were performed in *Rapid* mode with implicit solvent treatment enabled. For each compound, the species was first geometry-optimized using GFN2-xTB.[21] A subsequent higher-level single-point energy calculation was then performed using r^2SCAN-3c/COSMO(CH_3CN).[24] Reduction or oxidation events were modeled by adding or removing one electron from the designated species. The resulting species was subsequently geometry-optimized and re-evaluated using the same computational protocol.

The difference in electronic energies between the neutral and charged species was converted from Hartree to electronvolts. Redox potentials were then referenced to the saturated calomel electrode (SCE) by subtracting a constant offset of 4.422 V, as implemented in the Rowan redox workflow.

All calculations were performed using Rowan Scientific's web platform (accessed 2025-12-9 and 2025-11-5), and the results are shown in Table S26. We acknowledge Rowan Scientific for providing the redox-potential prediction workflow used in this study.

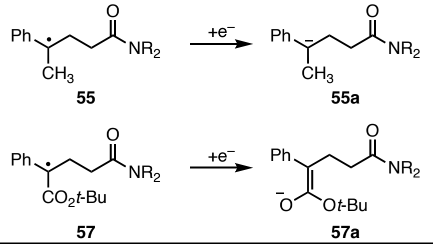

redox pair		calculated $E_{1/2}$ (vs SCE)
**55/55a**	R = CH_3	–1.713 V
**57/57a**	R = H	–0.606 V
	R = CH_3	–0.797 V

Table S26. Predicted redox potential values for **55/55a** and **57/57a**.

9.3 Biocatalysis modeling studies.

Computational method.

SILCS-Bio Molecular Dynamics-Monte Carlo Methodology

A novel in-silico approach – SILCS-Biologics was used to understand the binding affinity of the reactants on the enzyme surface, including the binding pocket. SILCS enables modeling of interactions of the reactants at an atomistic level. SILCS simulations with GROMACS[25] were performed using the SILCSBio package (provided by SILCSBio, LLC), where the model of the GluER-T36A molecule (ID: 6MYW) was obtained from the RCSB database and prepared using Molecular Operating Environment (MOE)[26] at pH 7.5. The FMN molecule was removed from the binding pocket prior to the SILCS simulations. The reactants were drawn using ChemDraw and energy-minimized using MOE. A combination of p3.2×large AWS with 1 NVIDIA Tesla V100 GPU and m5.16×large with x86_64 CPU architecture was used to run the SILCS simulations.

The protocol of SILCS simulations is described in Somani et al.[27] Briefly, 10 independent simulation systems involving the enzyme, water, and eight probe molecules that represent diverse functional groups that are common in small organic molecule to protein interaction, such as hydrophobic or nonpolar (benzene, propane), hydrogen bond acceptor and donor (methanol, formamide, acetaldehyde, imidazole), positive (methylammonium) and negative (acetate) charge interactions, were prepared. The choice of probe molecules and the validation of the SILCS approach in replicating protein–ligand interactions have been investigated in prior works.[28-31] Each system was minimized and equilibrated using 1 ns of MD simulation followed by 100 cycles of grand canonical Monte Carlo (GCMC)/MD simulation.[27] During each cycle, 200,000 steps of GCMC simulation were performed that drive the sampling of water and probe molecules, followed by 1 ns of MD simulation of the entire system, resulting in an aggregated simulation time of 1 μs (10 × 100 ns). The protein, probe, and water molecules were described using the CHARMM36m protein force field,[31-32] the CHARMM general force field (CGenFF),[33] and the TIP3P water model modified for the CHARMM force field.[34]

The GCMC/MD-based approach resulted in the generation of fragment maps (FragMaps) for the enzyme by binding the selected probe atoms into the voxels of a 1Å spaced grid spanning the simulation system. The voxel occupancy was used to measure a grid-based free energy (GFE, ΔG) using the equation $\Delta G_i = -RT\ln\left(\frac{N_i}{N_{bulk}}\right)$ where R is the universal gas constant, T is the system temperature, N_I is the observed voxel occupancy of the probe at grid point i and N_{bulk} is the expected voxel occupancy of the probe alone in the bulk solution. Therefore, GFE is a measure of the free energy change of moving an atom from the bulk state to the grid point i. For instance, if the GFE of a voxel near protein is −1.5 kcal/mol, then the probability of probe atoms is about 12 times more likely to be found in the voxels than in the voxels that are far away from the protein ("bulk") at room temperature.

Individual systems for each reactant were generated using the docking and screening algorithm (SILCS-MC), which involves MC sampling of the ligand (reactant) in translational, rotational, and torsional space.[29] The primary purpose is to sample ligand binding affinity in the field of FragMaps, along with the SILCS exclusion map. The exclusion map prevents the sampling of the ligand in the interior region of the protein where no water or probe molecules visited during the SILCS GCMC/MD step. The energy associated with ligand conformation is based on CGenFF intramolecular forces along with the LGFE score that is an approximate representation of the minimum free energy binding conformation, defined as the sum of atomic GFEs.[28-29] LGFEs have been shown to correlate well with the binding affinities of small, drug-like molecules to a range of proteins.[28]

A surface map of GluER-T36A is shown in Fig. S17. SILCSBio simulations identified a series of potential binding sites for **53**. Several non-specific surface binding sites (highlighted in orange) were detected; these sites are considered detrimental to reaction efficiency, as they may sequester **53** and compete with productive binding. In addition, SILCSBio analysis revealed a continuous series of binding sites leading into the active site, which we interpret as a putative diffusion pathway for **53** (yellow arrow). The binding GFEs of each site along this pathway were calculated. Efficient diffusion requires a progressive decrease in binding energy, and therefore, the energetically unfavorable "bottleneck" positions along the trajectory were identified (shown in purple).

Guided by these data, four point mutations were predicted to enhance catalytic efficiency. Of note, residues situated near well-defined secondary structural elements were excluded to minimize structural perturbation. The selected mutations, the rationale for their selection, and the experimental validation of their reactivity are summarized in Table S27.

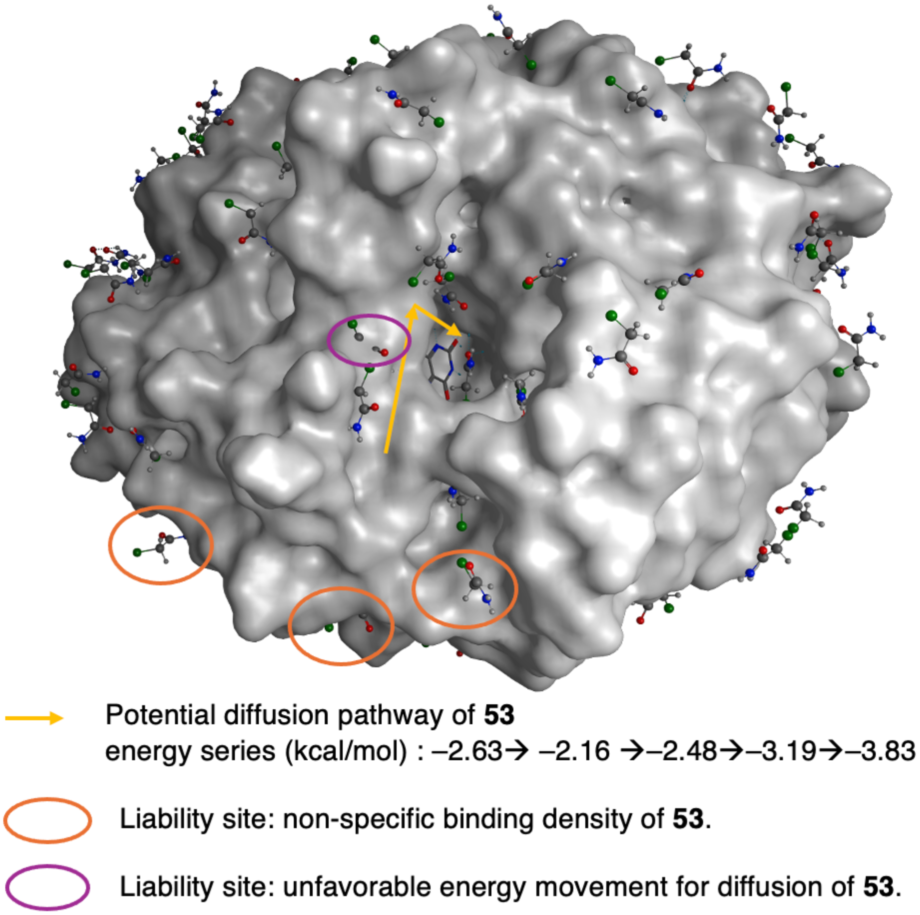

Figure S17. Surface map of GluER-T36A with **53** binding sites identified by SILCSBio simulations.

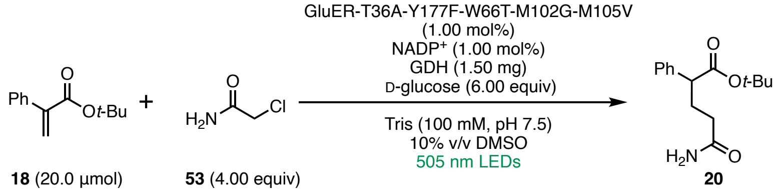

mutation	rationale	yield	er
–	–	44%	88:12
S22A	facilitate effective diffusion	39%	91:9
T36E	suppress non-specific binding	36%	84:16
**A44E**	**suppress non-specific binding**	**49%**	**89:11**
F68Y	suppress non-specific binding	32%	72:28

Table S27. Analytical scale reaction conditions and results for the predicted point mutants. Parent enzyme: GluER-T36A-Y177F-W66T-M102G-M105V. Yields were determined by LC-MS analysis using TBB as an internal standard and quantified against a standard calibration curve. Enantiomeric ratios were determined using a Shimadzu LC-2050C liquid chromatograph system with water and acetonitrile as mobile phases equipped with CHIRALCEL® OX-3R column (4.6 × 250 mm, 3 µm).

10. Cyclic voltammetry studies.

All electrochemical experiments were executed with a Bio-Logic VSP potentiostat. Cyclic voltammetry measurements were conducted in 0.1 M $TBAPF_6$ solution in acetonitrile with a glassy carbon working electrode, a Pt mesh counter electrode, and an Ag/AgCl wire pseudoreference electrode. The glassy carbon electrode was cleaned by polishing with 1, 0.3, and 0.05 μm diameter alumina powder from BASI. Pt electrode was cleaned by sonicating in dimethyl sulfoxide for 15 min followed by rinsing with acetone. The Ag/AgCl electrode was made by dipping Ag wire in Clorox bleach, followed by rinsing with MiliQ water and acetone. The system was purged with N_2 for 15 mins.

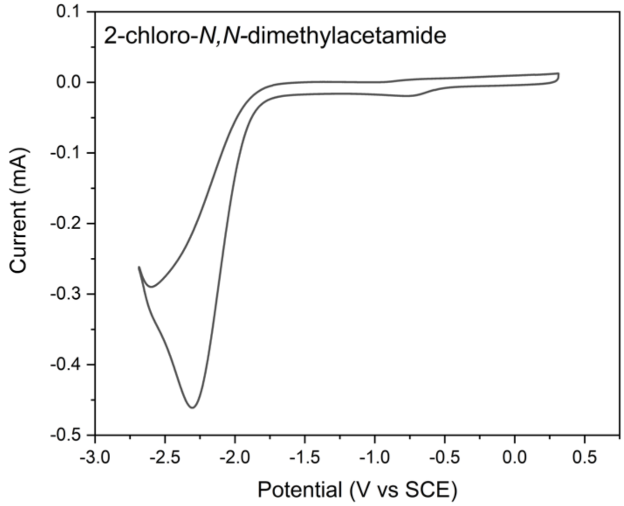

Figure S18. Cyclic voltammetry graph of 2-chloro-*N*,*N*-dimethylacetamide (**S19**).

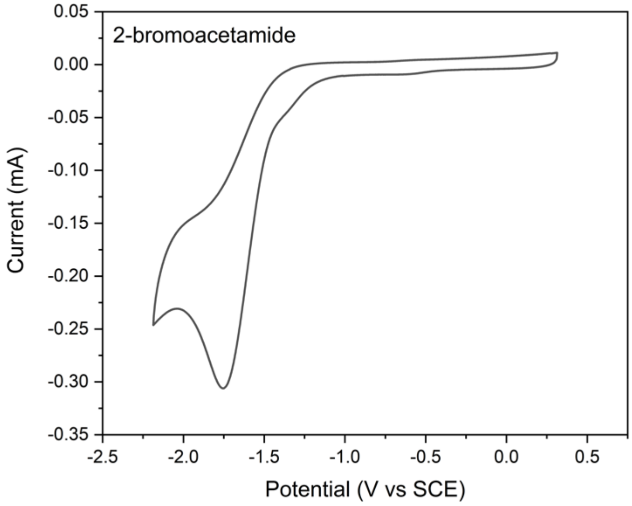

Figure S19. Cyclic voltammetry graph of 2-bromoacetamide (**19**).

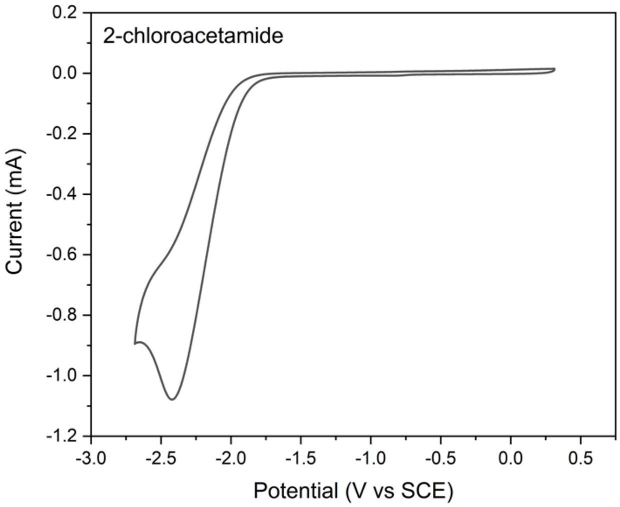

Figure S20. Cyclic voltammetry graph of 2-chloroacetamide (**53**).

compound	$E_{1/2}$ vs SCE
2-chloro-*N*,*N*-dimethylacetamide (**S19**)	–2.303 V
2-bromoacetamide (**19**)	–1.753 V
2-chloroacetamide (**53**)	–2.418 V

Table S28. Tabulated redox potentials determined by cyclic voltametry.

11. Chiral HPLC traces.

tert-butyl 5-amino-5-oxo-2-phenylpentanoate (**20**):

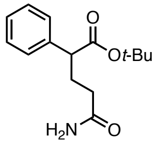

20

Procedure A:

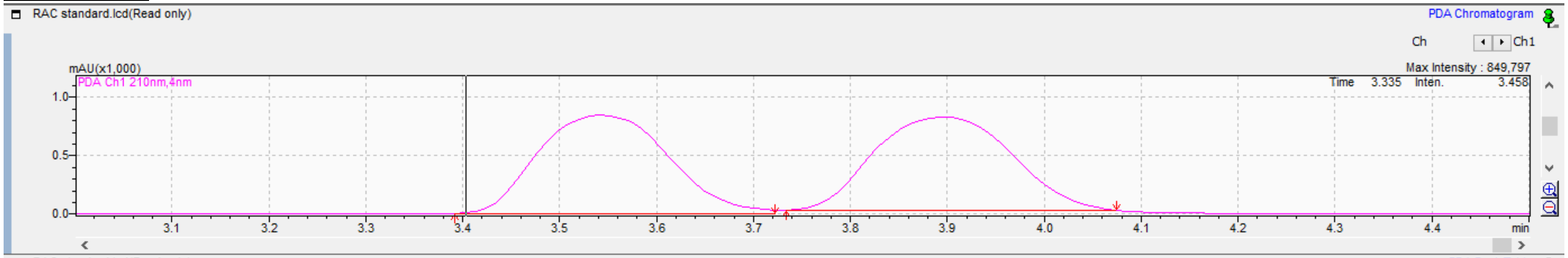

RAC standard.lcd(Read only) PDA Peak Table

Peak#	Ret. Time	Area	Area%
1	3.544	7993256	50.325
2	3.894	7889932	49.675
Total		15883188	100.000

CHIRALCEL® OX-3R column, 55% acetonitrile, 45% water; t_R1 = 3.54 min, t_R2 = 3.89 min.

Procedure B:

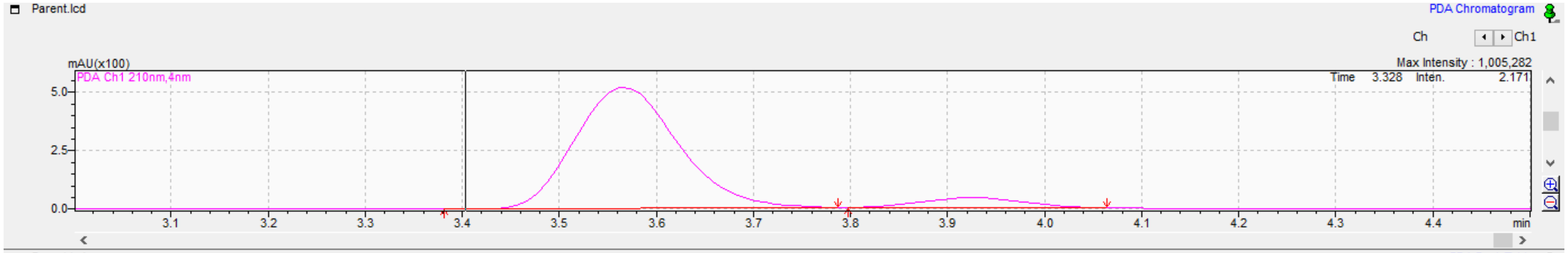

Parent.lcd PDA Peak Table

Peak#	Ret. Time	Area	Area%
1	3.567	3820430	92.746
2	3.927	298800	7.254
Total		4119230	100.000

CHIRALCEL® OX-3R column, 55% acetonitrile, 45% water; t_R1 = 3.57 min, t_R2 = 3.93 min.

tert-butyl 5-amino-5-oxo-2-(*o*-tolyl)pentanoate (**21**):

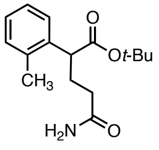

21

Procedure A:

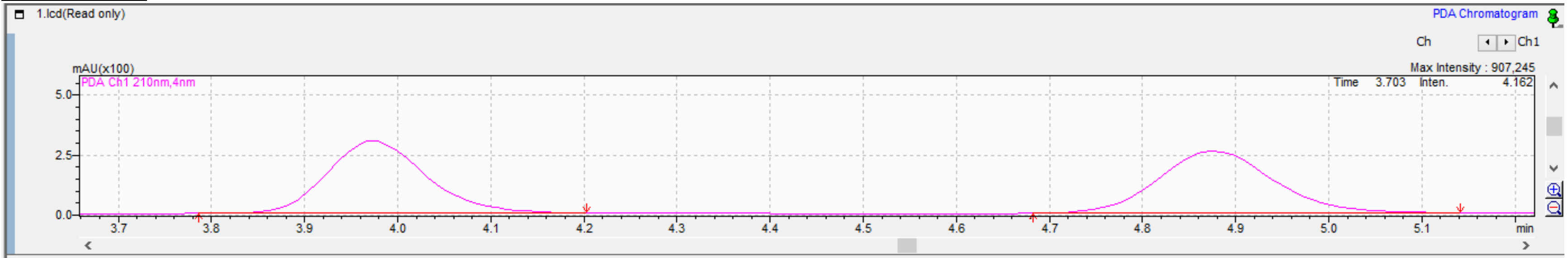

1.lcd(Read only) PDA Peak Table

Peak#	Ret. Time	Area	Area%
1	3.973	2222977	49.814
2	4.876	2239597	50.186
Total		4462574	100.000

CHIRALCEL® OX-3R column, 55% acetonitrile, 45% water; t_R1 = 3.97 min, t_R2 = 4.88 min.

Procedure B:

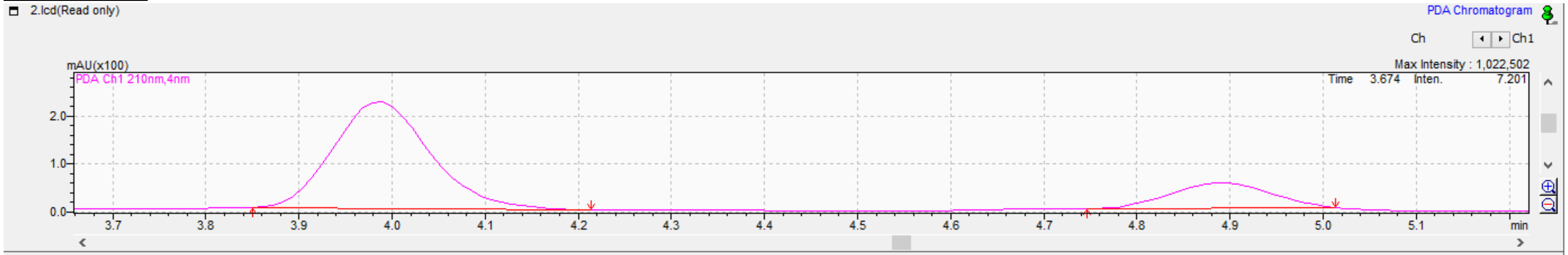

2.lcd(Read only) PDA Peak Table

Peak#	Ret. Time	Area	Area%
1	3.985	1608565	80.340
2	4.890	393637	19.660
Total		2002202	100.000

CHIRALCEL® OX-3R column, 55% acetonitrile, 45% water; t_R1 = 3.99 min, t_R2 = 4.89 min.

tert-butyl 5-amino-5-oxo-2-(*m*-tolyl)pentanoate (**22**):

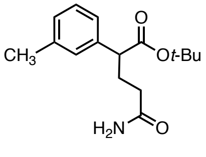

22

Procedure A:

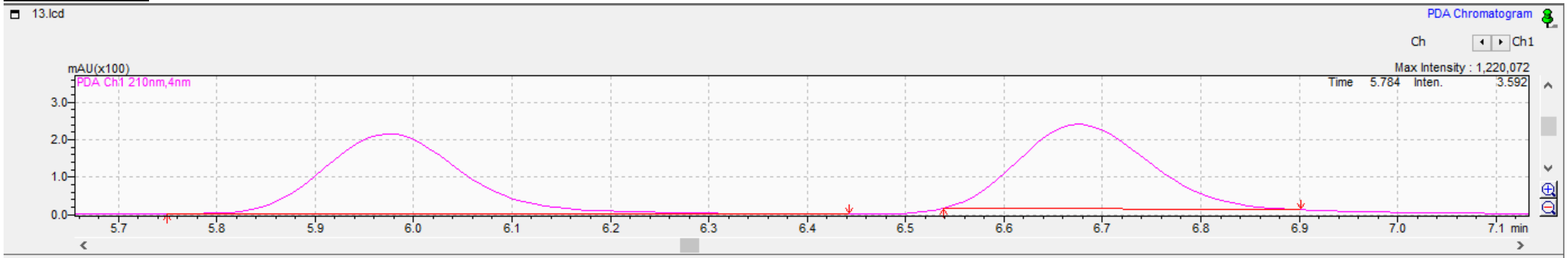

13.lcd PDA Peak Table

Peak#	Ret. Time	Area	Area%
1	5.975	2099163	50.390
2	6.676	2066638	49.610
Total		4165801	100.000

CHIRALCEL® OX-3R column, 45% acetonitrile, 55% water; t_R1 = 5.98 min, t_R2 = 6.68 min.

Procedure B:

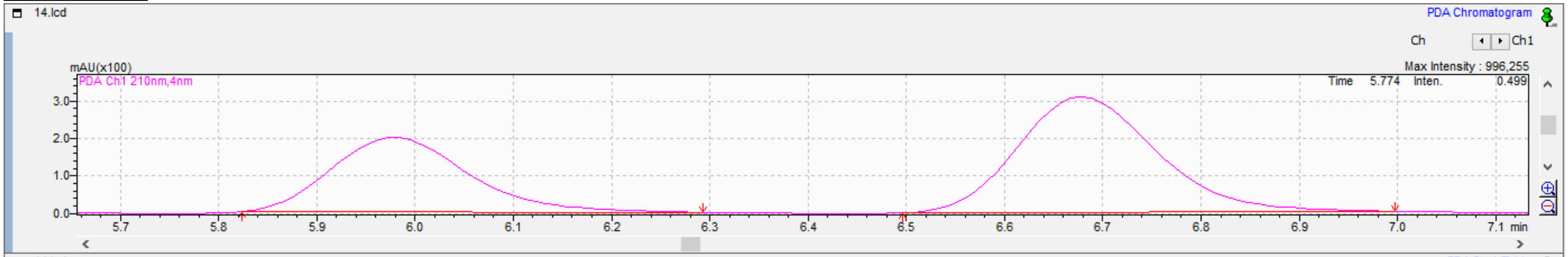

14.lcd PDA Peak Table

Peak#	Ret. Time	Area	Area%
1	5.979	1915526	38.731
2	6.678	3030216	61.269
Total		4945742	100.000

CHIRALCEL® OX-3R column, 45% acetonitrile, 55% water; t_R1 = 5.98 min, t_R2 = 6.68 min.

tert-butyl 5-amino-5-oxo-2-(*p*-tolyl)pentanoate (**23**):

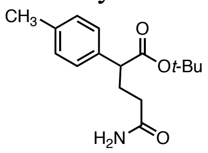

23

Procedure A:

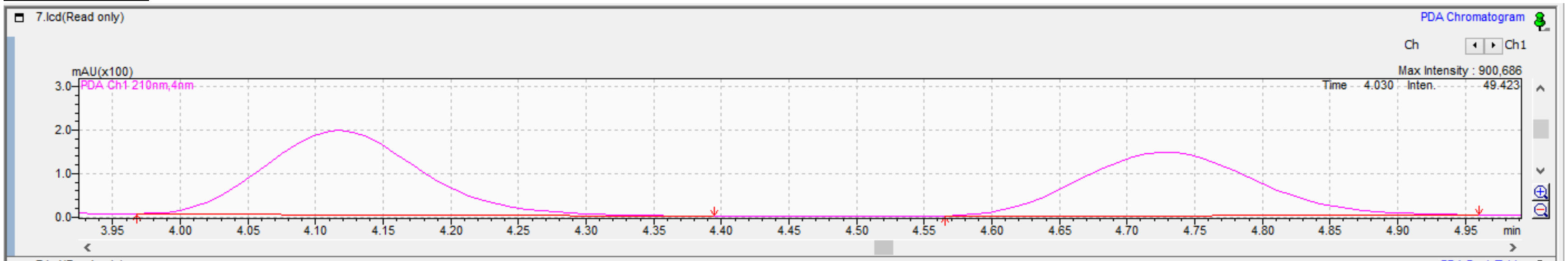

7.lcd(Read only) PDA Peak Table

Peak#	Ret. Time	Area	Area%
1	4.117	1553188	54.248
2	4.729	1309915	45.752
Total		2863102	100.000

CHIRALCEL® OX-3R column, 55% acetonitrile, 45% water; t_R1 = 4.12 min, t_R2 = 4.73 min.

Procedure B:

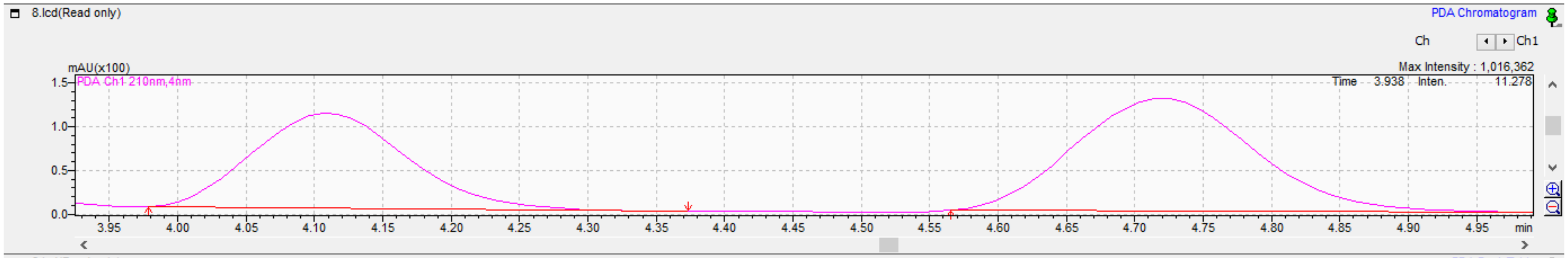

8.lcd(Read only) PDA Peak Table

Peak#	Ret. Time	Area	Area%
1	4.109	853788	42.090
2	4.720	1174714	57.910
Total		2028502	100.000

CHIRALCEL® OX-3R column, 55% acetonitrile, 45% water; t_R1 = 4.11 min, t_R2 = 4.72 min.

tert-butyl 5-amino-2-(2-methoxyphenyl)-5-oxopentanoate (**24**):

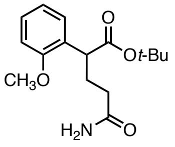

24

Procedure A:

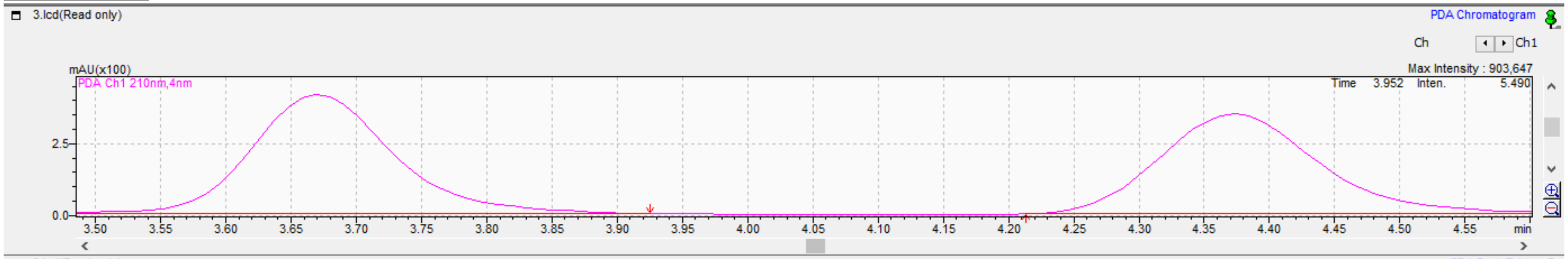

3.lcd(Read only) PDA Peak Table

Peak#	Ret. Time	Area	Area%
1	3.670	3107002	51.409
2	4.373	2936748	48.591
Total		6043750	100.000

CHIRALCEL® OX-3R column, 55% acetonitrile, 45% water; t_R1 = 3.67 min, t_R2 = 4.37 min.

Procedure B:

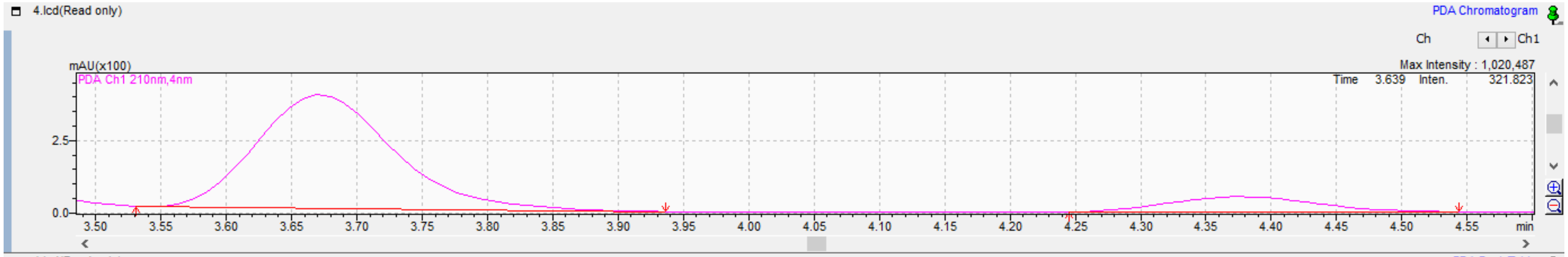

4.lcd(Read only) PDA Peak Table

Peak#	Ret. Time	Area	Area%
1	3.672	2864778	87.485
2	4.377	409812	12.515
Total		3274590	100.000

CHIRALCEL® OX-3R column, 55% acetonitrile, 45% water; t_R1 = 3.67 min, t_R2 = 4.38 min.

tert-butyl 5-amino-2-(2-chlorophenyl)-5-oxopentanoate (**25**):

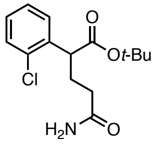

25

Procedure A:

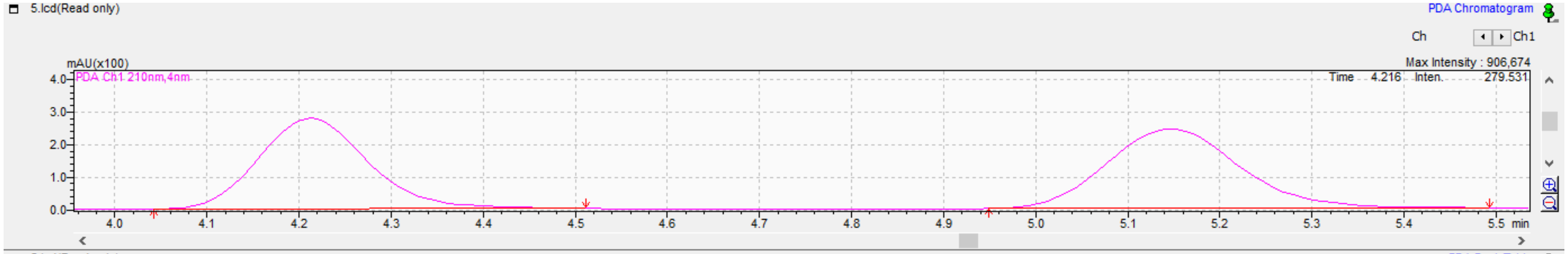

5.lcd(Read only) PDA Peak Table

Peak#	Ret. Time	Area	Area%
1	4.213	2281857	48.531
2	5.147	2419975	51.469
Total		4701832	100.000

CHIRALCEL® OX-3R column, 55% acetonitrile, 45% water; t_R1 = 4.21 min, t_R2 = 5.15 min.

Procedure B:

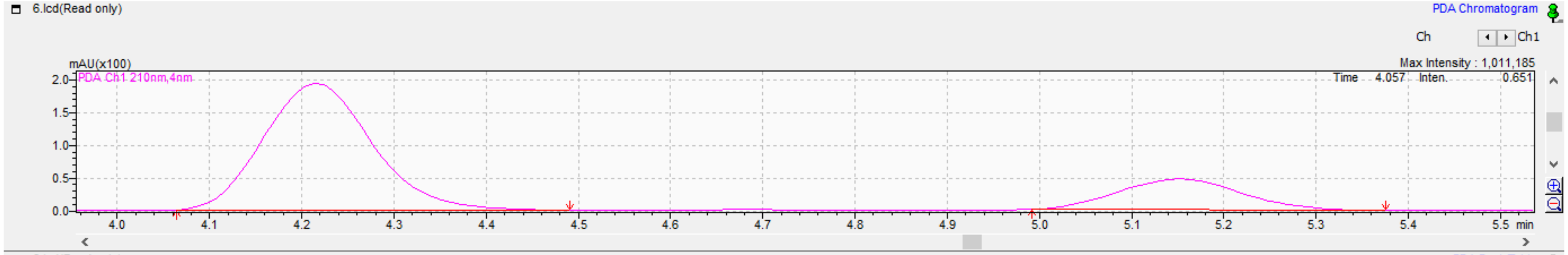

6.lcd(Read only) PDA Peak Table

Peak#	Ret. Time	Area	Area%
1	4.215	1582376	78.419
2	5.152	435459	21.581
Total		2017836	100.000

CHIRALCEL® OX-3R column, 55% acetonitrile, 45% water; t_R1 = 4.22 min, t_R2 = 5.15 min.

tert-butyl 5-amino-2-(2-bromophenyl)-5-oxopentanoate (**26**):

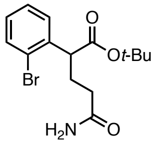

26

<u>Procedure A:</u>

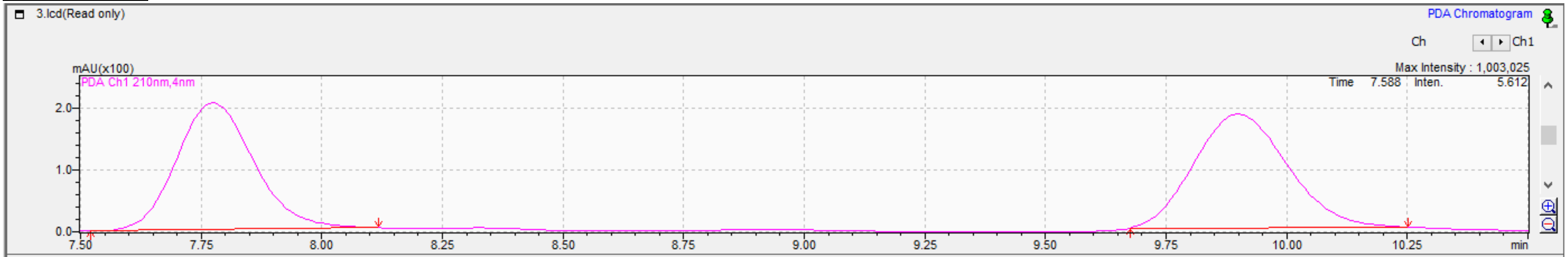

3.lcd(Read only) — PDA Peak Table

Peak#	Ret. Time	Area	Area%
1	7.774	2289815	48.131
2	9.899	2467694	51.869
Total		4757509	100.000

CHIRALCEL® OX-3R column, 45% acetonitrile, 55% water; t_R1 = 7.77 min, t_R2 = 9.90 min.

<u>Procedure B:</u>

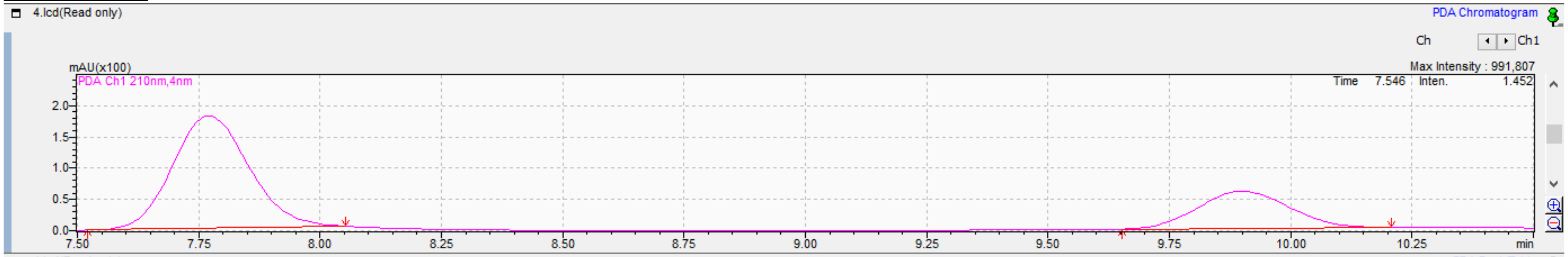

4.lcd(Read only) — PDA Peak Table

Peak#	Ret. Time	Area	Area%
1	7.771	1968355	71.852
2	9.901	771091	28.148
Total		2739445	100.000

CHIRALCEL® OX-3R column, 45% acetonitrile, 55% water; t_R1 = 7.77 min, t_R2 = 9.90 min.

tert-butyl 5-amino-2-(2-iodophenyl)-5-oxopentanoate (**27**):

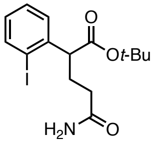

27

<u>Procedure A</u>:

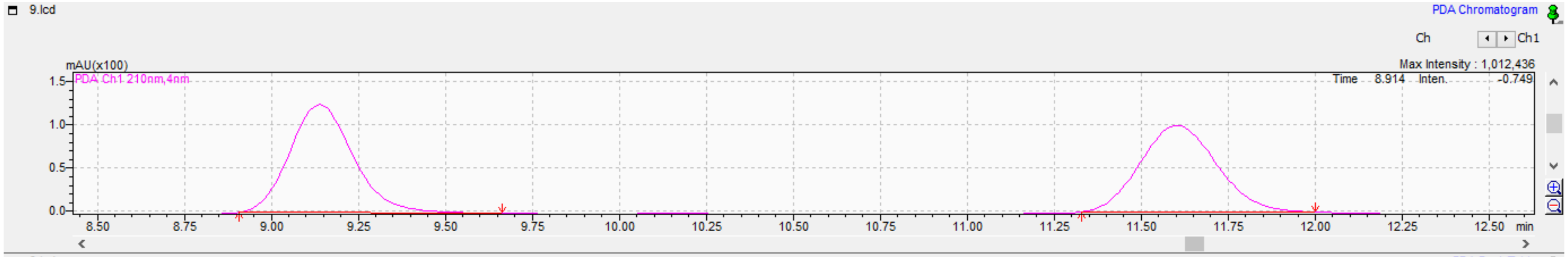

Peak#	Ret. Time	Area	Area%
1	9.139	1596604	50.904
2	11.603	1539915	49.096
Total		3136519	100.000

CHIRALCEL® OX-3R column, 45% acetonitrile, 55% water; t_R1 = 9.14 min, t_R2 = 11.60 min.

<u>Procedure B</u>:

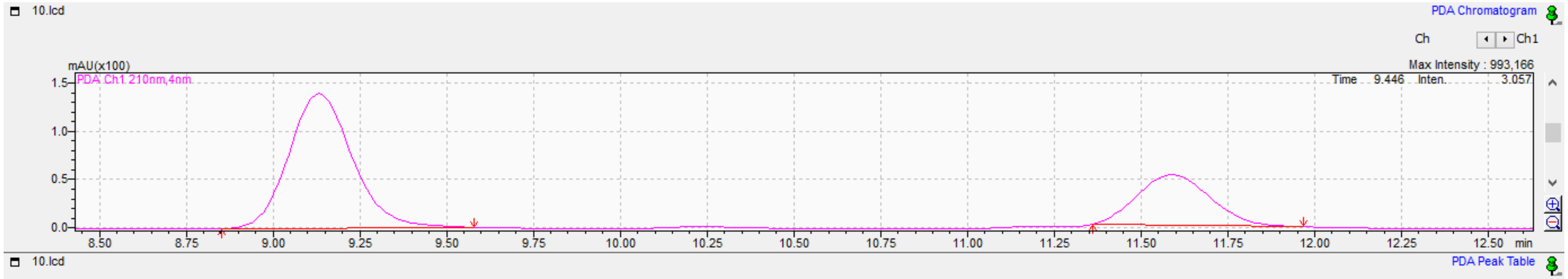

Peak#	Ret. Time	Area	Area%
1	9.132	1779528	69.919
2	11.588	765593	30.081
Total		2545121	100.000

CHIRALCEL® OX-3R column, 45% acetonitrile, 55% water; t_R1 = 9.13 min, t_R2 = 11.59 min.

tert-butyl 5-amino-2-(2-nitrophenyl)-5-oxopentanoate (**28**):

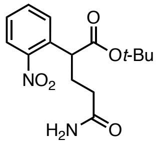

28

Procedure A:

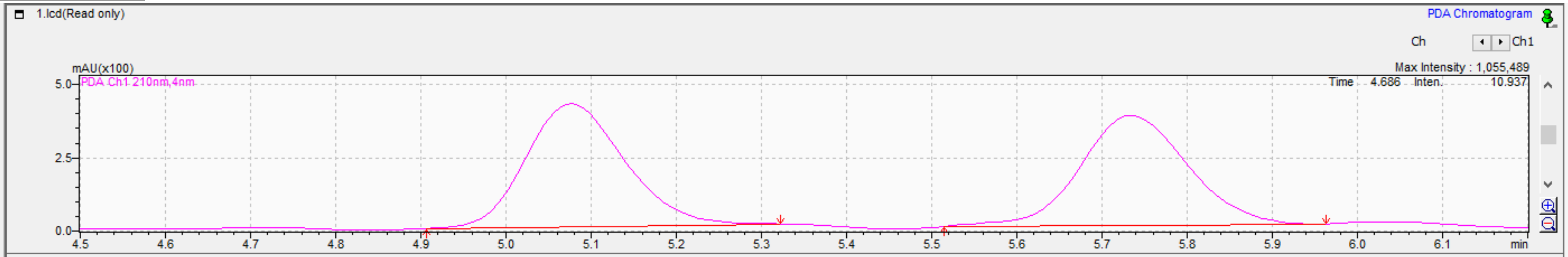

1.lcd(Read only) PDA Peak Table

Peak#	Ret. Time	Area	Area%
1	5.076	3374915	50.615
2	5.734	3292960	49.385
Total		6667875	100.000

CHIRALCEL® OX-3R column, 45% acetonitrile, 55% water; t_R1 = 5.08 min, t_R2 = 5.73 min.

Procedure B:

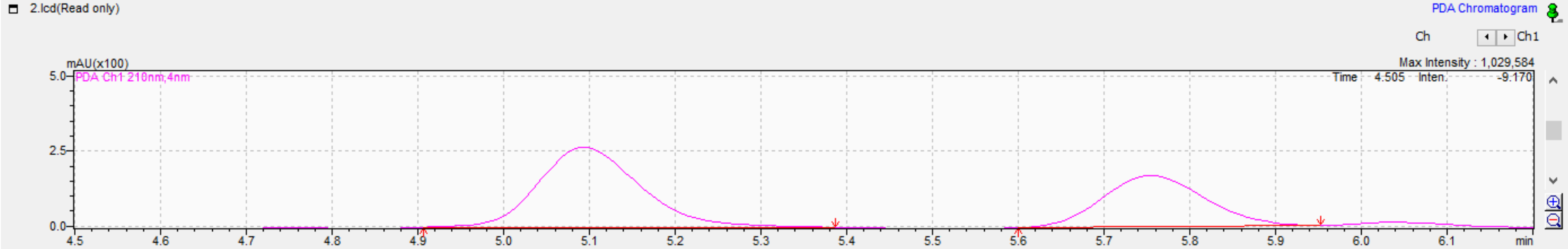

2.lcd(Read only) PDA Peak Table

Peak#	Ret. Time	Area	Area%
1	5.094	2206134	61.026
2	5.755	1408943	38.974
Total		3615077	100.000

CHIRALCEL® OX-3R column, 45% acetonitrile, 55% water; t_R1 = 5.09 min, t_R2 = 5.76 min.

tert-butyl 5-amino-2-(3-bromophenyl)-5-oxopentanoate (**29**):

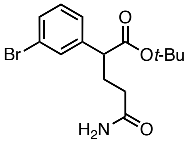

29

Procedure A:

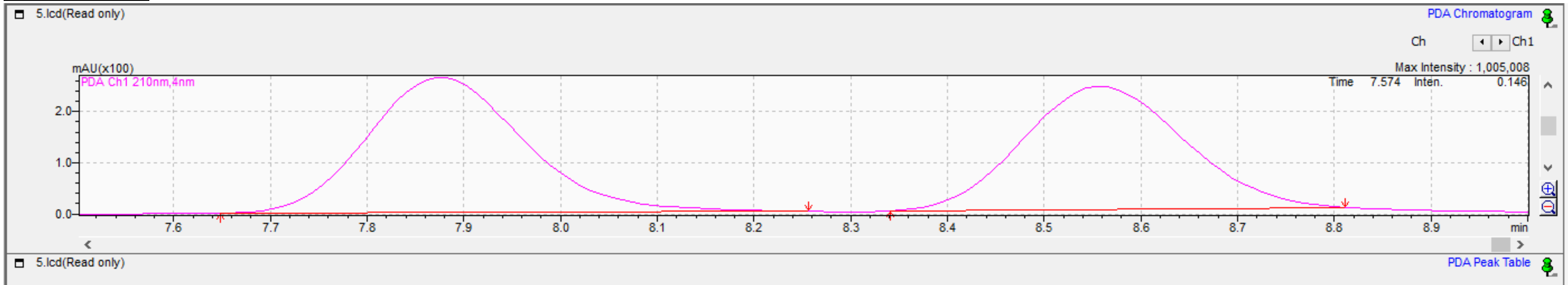

Peak#	Ret. Time	Area	Area%
1	7.875	2995272	51.698
2	8.558	2798523	48.302
Total		5793795	100.000

CHIRALCEL® OX-3R column, 45% acetonitrile, 55% water; t_R1 = 7.88 min, t_R2 = 8.56 min.

Procedure B:

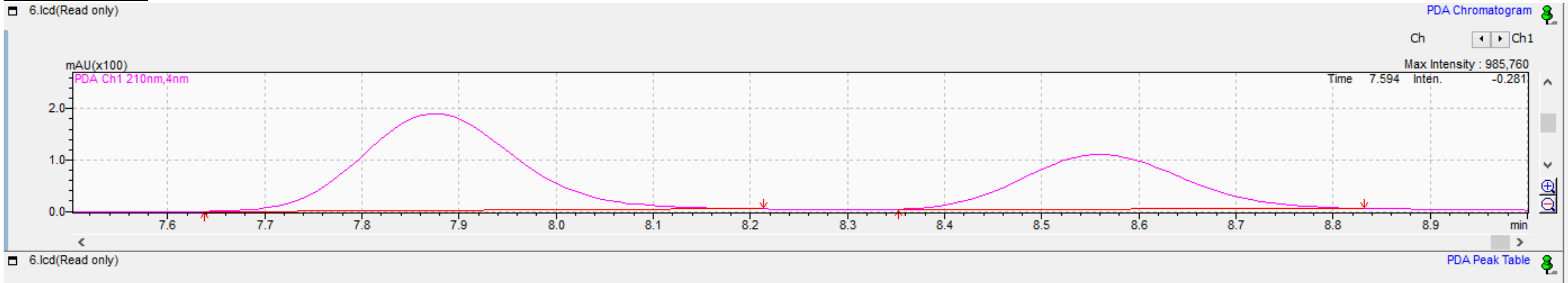

Peak#	Ret. Time	Area	Area%
1	7.875	2100473	63.243
2	8.560	1220791	36.757
Total		3321264	100.000

CHIRALCEL® OX-3R column, 45% acetonitrile, 55% water; t_R1 = 7.88 min, t_R2 = 8.56 min.

tert-butyl 5-amino-2-(4-bromophenyl)-5-oxopentanoate (**30**):

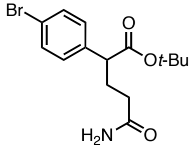

30

<u>Procedure A:</u>

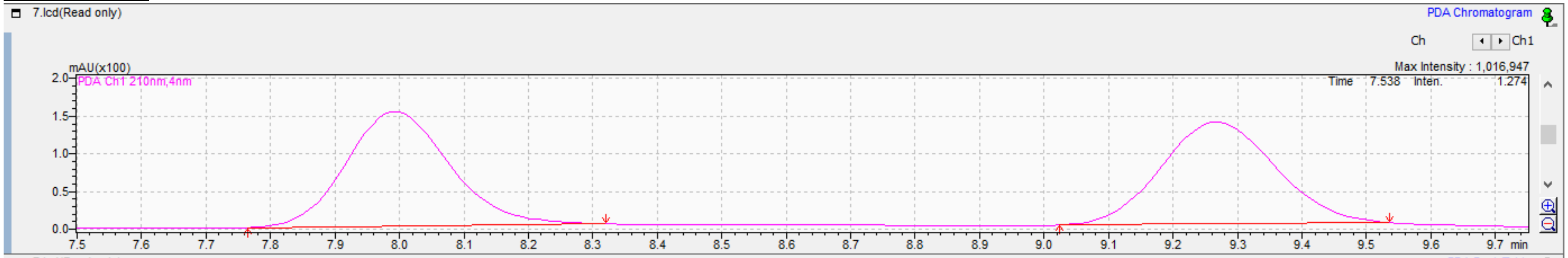

7.lcd(Read only) PDA Peak Table

Peak#	Ret. Time	Area	Area%
1	7.993	1696215	50.442
2	9.267	1666486	49.558
Total		3362701	100.000

CHIRALCEL® OX-3R column, 45% acetonitrile, 55% water; t_R1 = 8.00 min, t_R2 = 9.27 min.

<u>Procedure B:</u>

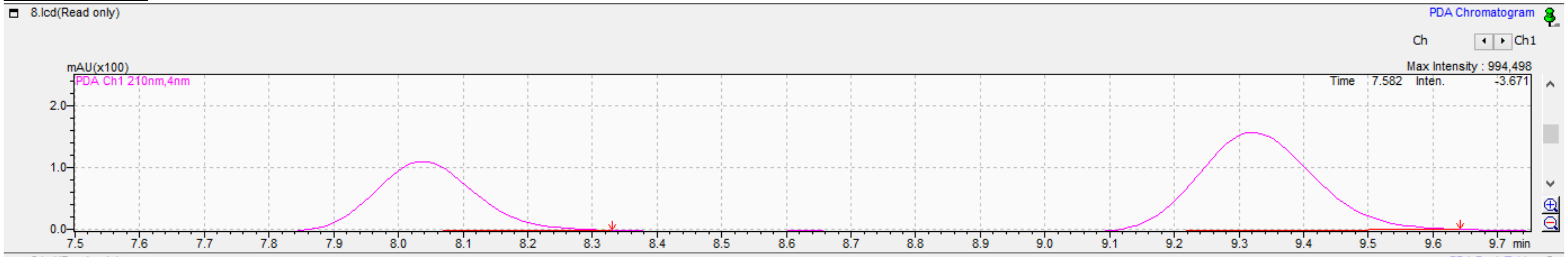

8.lcd(Read only) PDA Peak Table

Peak#	Ret. Time	Area	Area%
1	8.037	1224373	38.073
2	9.321	1991503	61.927
Total		3215876	100.000

CHIRALCEL® OX-3R column, 45% acetonitrile, 55% water; t_R1 = 8.04 min, t_R2 = 9.32 min.

tert-butyl 5-amino-2-(4-methoxyphenyl)-5-oxopentanoate (**31**):

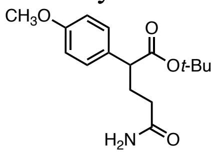

31

Procedure A:

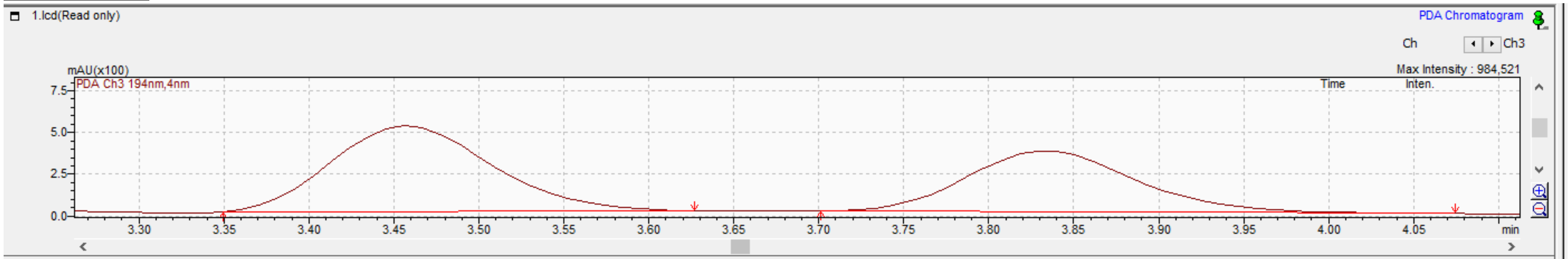

1.lcd(Read only) PDA Peak Table

Peak#	Ret. Time	Area	Area%
1	3.457	3353259	57.122
2	3.833	2517058	42.878
Total		5870317	100.000

CHIRALCEL® OX-3R column, 55% acetonitrile, 45% water; t_R1 = 3.46 min, t_R2 = 3.83 min.

Procedure B:

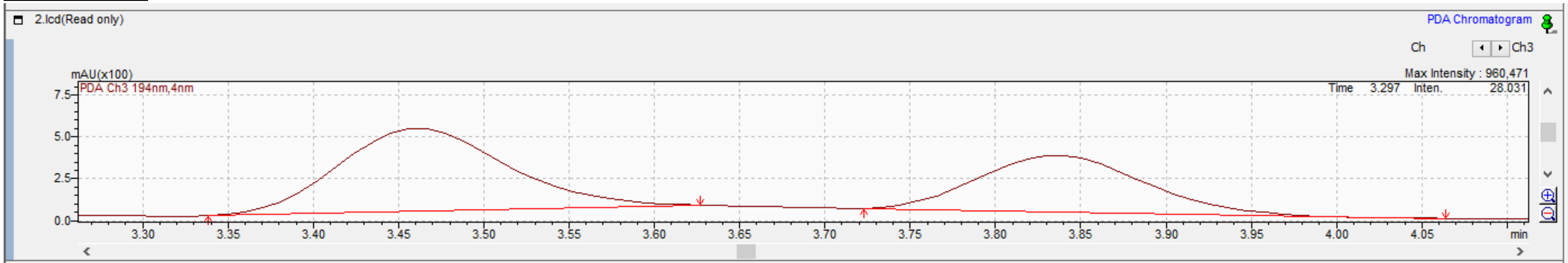

2.lcd(Read only) PDA Peak Table

Peak#	Ret. Time	Area	Area%
1	3.461	3405730	60.593
2	3.835	2214903	39.407
Total		5620633	100.000

CHIRALCEL® OX-3R column, 55% acetonitrile, 45% water; t_R1 = 3.46 min, t_R2 = 3.84 min.

tert-butyl 5-amino-2-(4-((*tert*-butoxycarbonyl)amino)phenyl)-5-oxopentanoate (**32**):

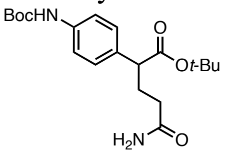

32

Procedure A:

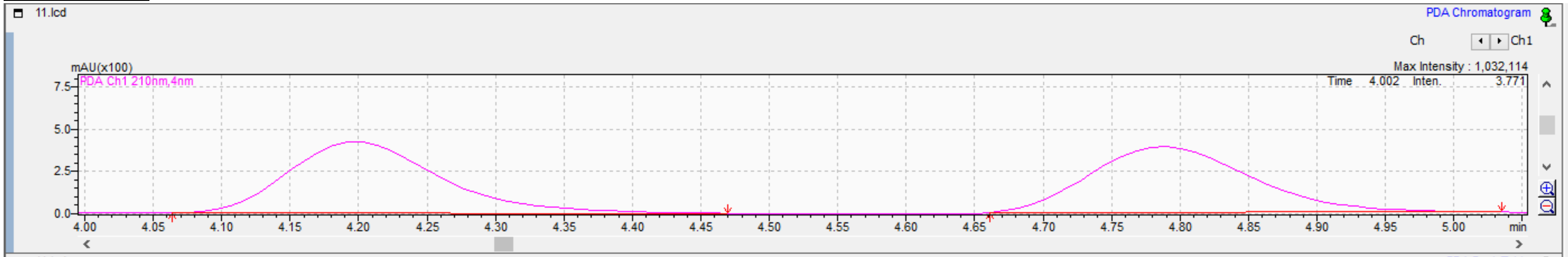

11.lcd PDA Peak Table

Peak#	Ret. Time	Area	Area%
1	4.197	3243919	50.381
2	4.787	3194826	49.619
Total		6438745	100.000

CHIRALCEL® OX-3R column, 55% acetonitrile, 45% water; t_R1 = 4.20 min, t_R2 = 4.79 min.

Procedure B:

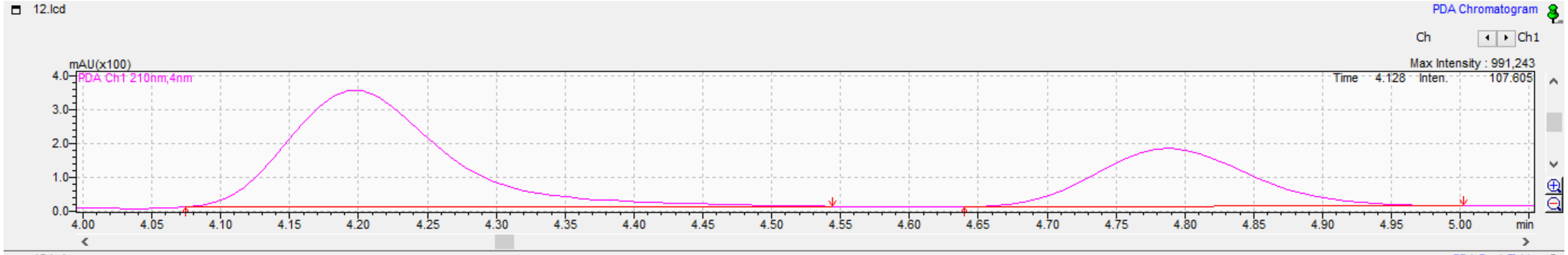

12.lcd PDA Peak Table

Peak#	Ret. Time	Area	Area%
1	4.197	2684349	66.800
2	4.788	1334145	33.200
Total		4018494	100.000

CHIRALCEL® OX-3R column, 55% acetonitrile, 45% water; t_R1 = 4.20 min, t_R2 = 4.79 min.

methyl-5-amino-5-oxo-2-phenylpentanoate (**33**):

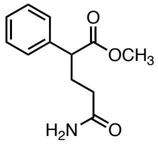

33

Procedure A:

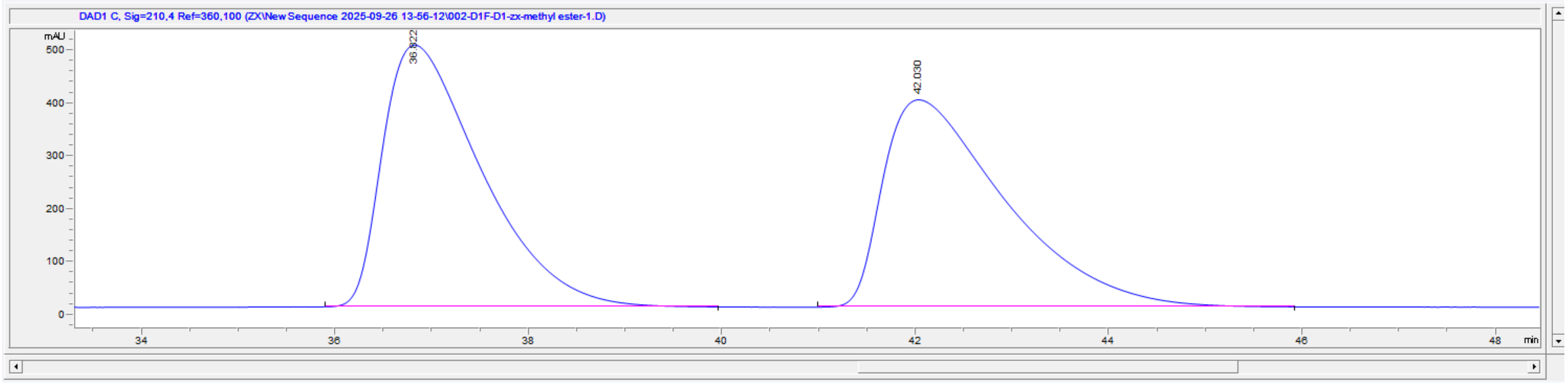

#	Time	Type	Area	Height	Width	Area%	Symmetry
1	36.822	BB	35250.3	496.4	0.9745	50.516	0.451
2	42.03	BB	34530.8	393.1	1.0479	49.484	0.385

CHIRALPAK® IG column, 5% *iso*-propanol, 95% hexane; t_R1 = 36.82 min, t_R2 = 42.03 min.

ethyl-5-amino-5-oxo-2-phenylpentanoate (**34**):

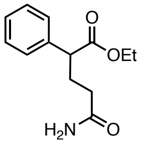

34

<u>Procedure A</u>:

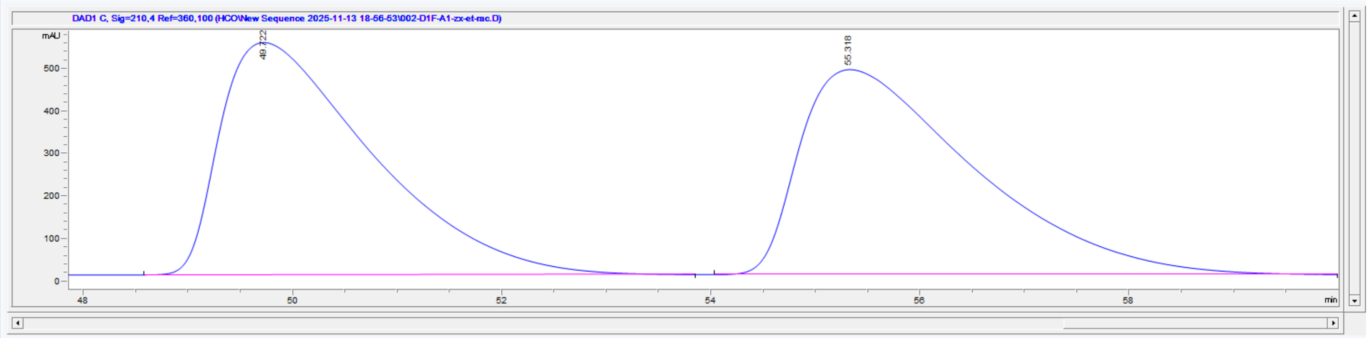

#	Time	Type	Area	Height	Width	Area%	Symmetry
1	49.722	BB	54958.3	545.4	1.2176	49.654	0.377
2	55.318	BBA	55723.9	481.3	1.3579	50.346	0.385

CHIRALPAK® IG column, 5% *iso*-propanol, 95% hexane; t_R1 = 49.72 min, t_R2 = 55.32 min.

<u>Procedure B</u>:

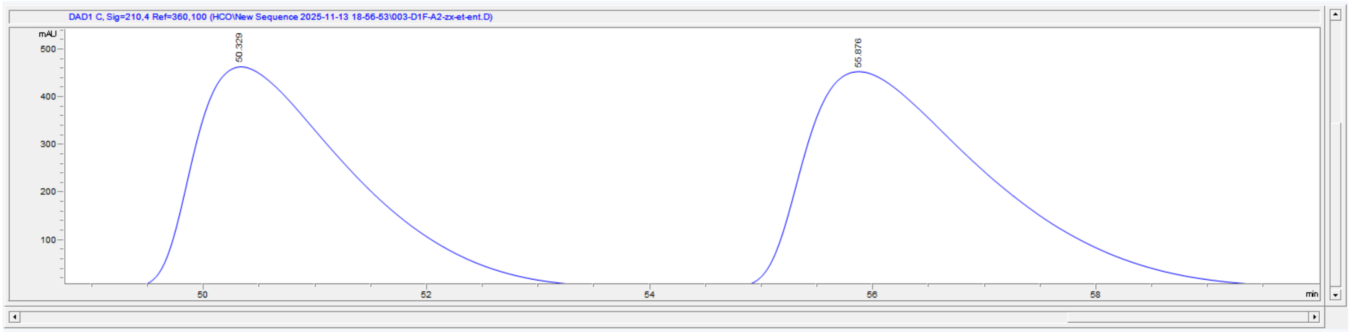

#	Time	Type	Area	Height	Width	Area%	Symmetry
1	50.329	BB R	45354.3	463.1	1.2805	46.872	0.376
2	55.876	BBA	51408.4	452.5	1.3444	53.128	0.4

CHIRALPAK® IG column, 5% *iso*-propanol, 95% hexane; t_R1 = 50.33 min, t_R2 = 55.88 min.

benzyl 5-amino-5-oxo-2-phenylpentanoate (**35**):

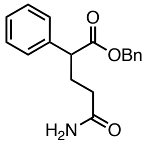

35

<u>Procedure A</u>:

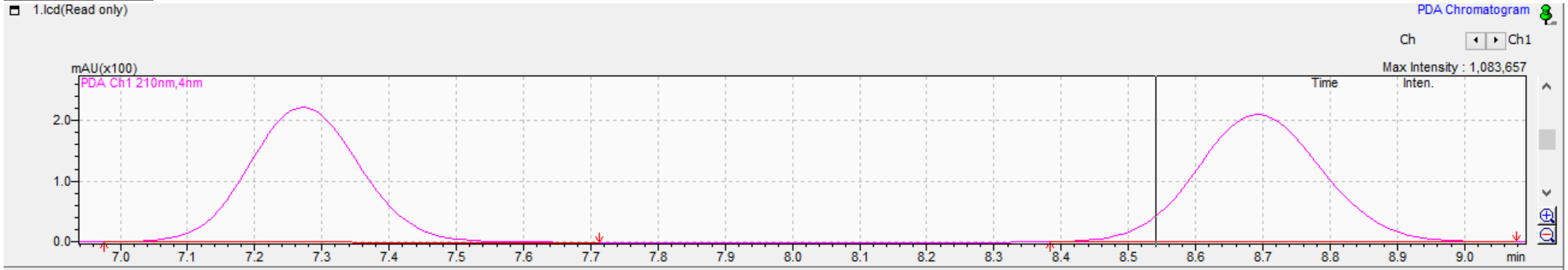

1.lcd(Read only) PDA Peak Table

Peak#	Ret. Time	Area	Area%
1	7.272	2569706	48.121
2	8.692	2770439	51.879
Total		5340145	100.000

Chiral HPLC method: CHIRALCEL® OX-3R column, 45% acetonitrile, 55% water; t_R1 = 7.27 min, t_R2 = 8.69 min.

<u>Procedure B</u>:

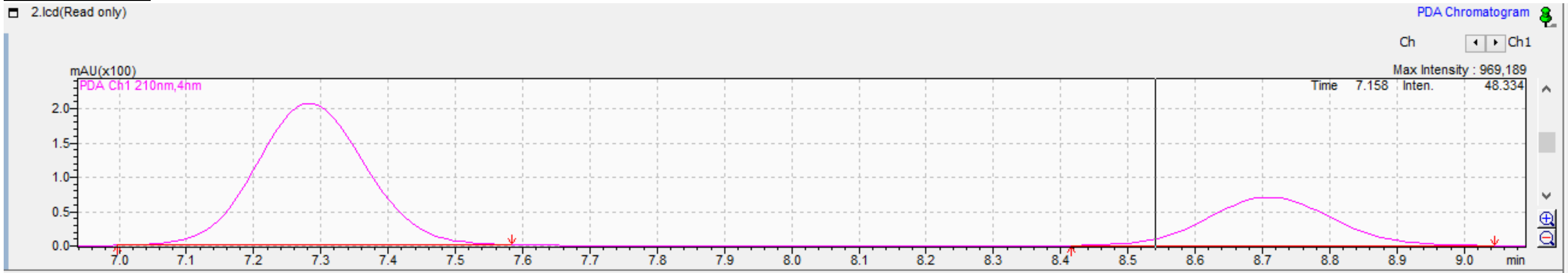

2.lcd(Read only) PDA Peak Table

Peak#	Ret. Time	Area	Area%
1	7.283	2352965	71.988
2	8.710	915606	28.012
Total		3268571	100.000

Chiral HPLC method: CHIRALCEL® OX-3R column, 45% acetonitrile, 55% water; t_R1 = 7.28 min, t_R2 = 8.71 min.

tert-pentyl 5-amino-5-oxo-2-phenylpentanoate (**36**):

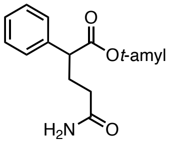

36

Procedure A:

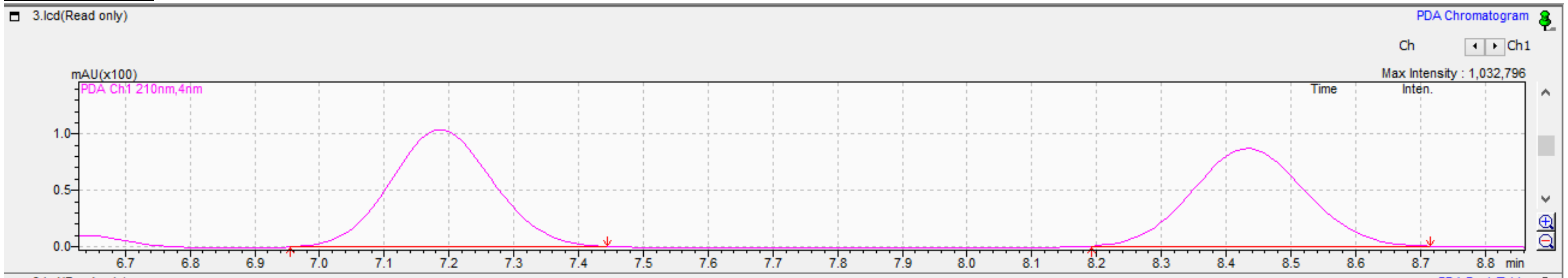

3.lcd(Read only) PDA Peak Table

Peak#	Ret. Time	Area	Area%
1	7.187	1150131	52.005
2	8.433	1061440	47.995
Total		2211571	100.000

Chiral HPLC method: CHIRALCEL® OX-3R column, 45% acetonitrile, 55% water; t_R1 = 7.19 min, t_R2 = 8.43 min.

Procedure B:

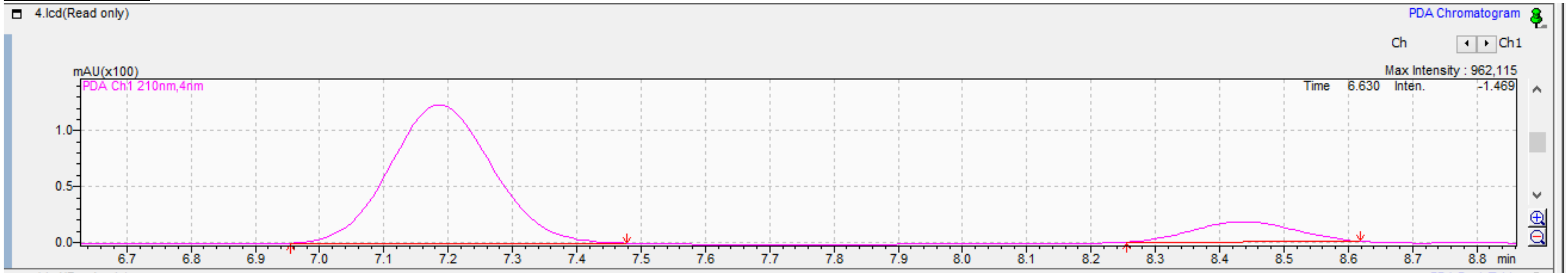

4.lcd(Read only) PDA Peak Table

Peak#	Ret. Time	Area	Area%
1	7.187	1371074	87.613
2	8.438	193844	12.387
Total		1564917	100.000

Chiral HPLC method: CHIRALCEL® OX-3R column, 45% acetonitrile, 55% water; t_R1 = 7.19 min, t_R2 = 8.44 min.

tert-butyl 5-amino-4-methyl-5-oxo-2-phenylpentanoate (**37**):

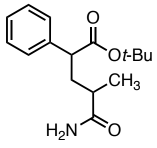

37

<u>Procedure A</u>:

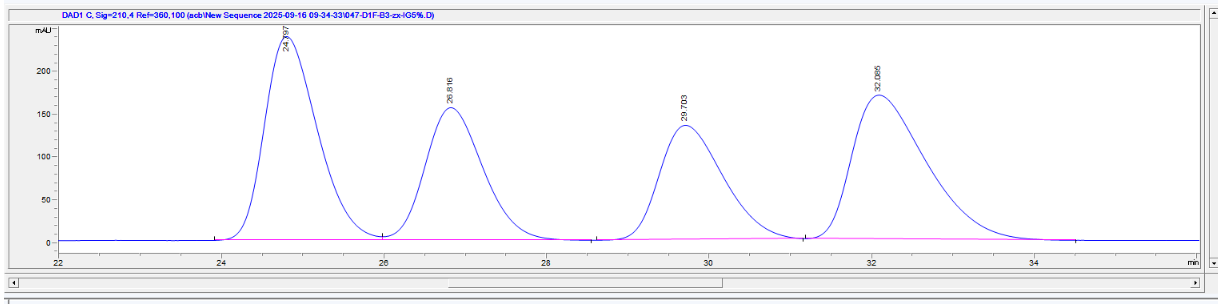

#	Time	Type	Area	Height	Width	Area%	Symmetry
1	24.797	BV	10767.6	237.8	0.6983	29.562	0.686
2	26.816	VB	7691.2	154.7	0.7309	21.116	0.775
3	29.703	BB	7266.7	133.4	0.7547	19.950	0.662
4	32.085	BB	10698.2	168	0.9221	29.372	0.544

CHIRALPAK® IG column, 5% *iso*-propanol, 95% hexane; t_R1 = 24.80 min, t_R2 = 26.82 min, t_R1^* = 29.70 min, t_R2^* = 32.09 min.

<u>Procedure B</u>:

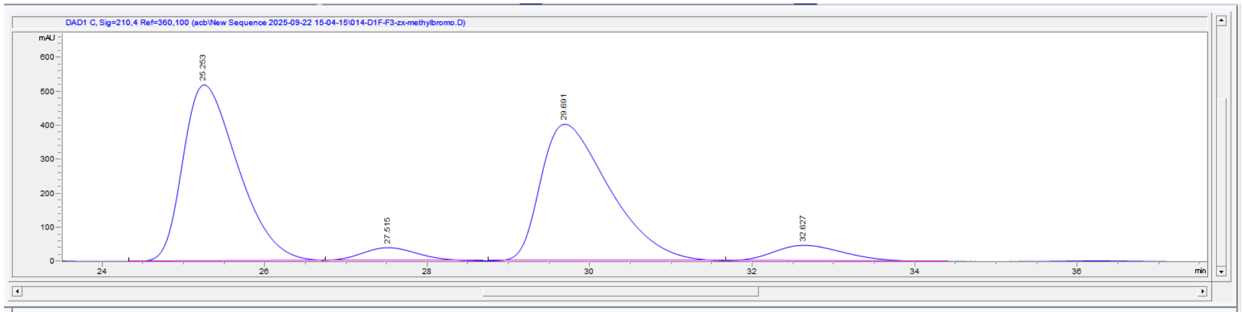

#	Time	Type	Area	Height	Width	Area%	Symmetry
1	25.253	BB	24218.9	517	0.7015	46.353	0.601
2	27.515	BB	1750.6	37.5	0.5558	3.351	0.845
3	29.691	BB	23544.8	401.1	0.8639	45.063	0.52
4	32.627	BB	2734.7	45.7	0.7099	5.234	0.714

CHIRALPAK® IG column, 5% *iso*-propanol, 95% hexane; t_R1 = 25.25 min, t_R2 = 27.52 min, t_R1^* = 29.69 min, t_R2^* = 32.63 min.

tert-butyl 4-carbamoyl-2-phenylhexanoate (**38**):

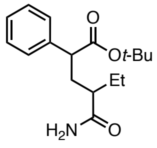

38

Procedure A:

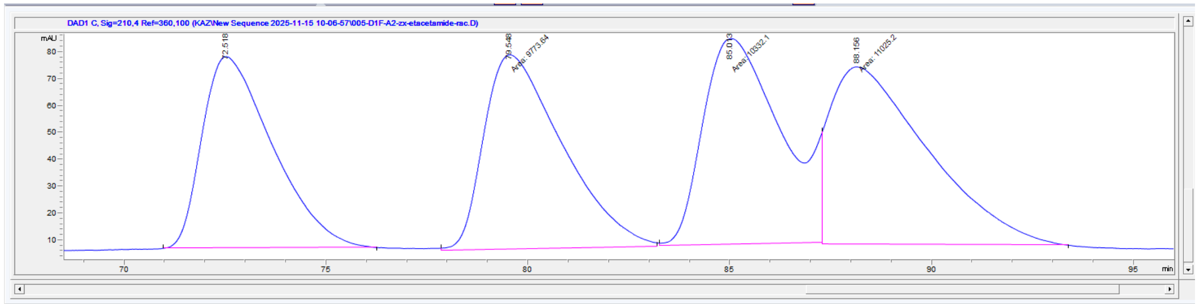

#	Time	Type	Area	Height	Width	Area%	Symmetry
1	72.518	BB	8606.9	71.1	1.4205	21.659	0.51
2	79.548	MM	9773.6	72.3	2.2544	24.595	0.487
3	85.013	MM	10332.1	76.4	2.2542	26.001	0.532
4	88.156	FM	11025.2	65.8	2.7934	27.745	0.37

CHIRALPAK® IE column, 2% *iso*-propanol, 98% hexane; t_R1 = 72.52 min, t_R2 = 79.55 min, t_R1^* = 85.01 min, t_R2^* = 88.16 min.

Procedure B:

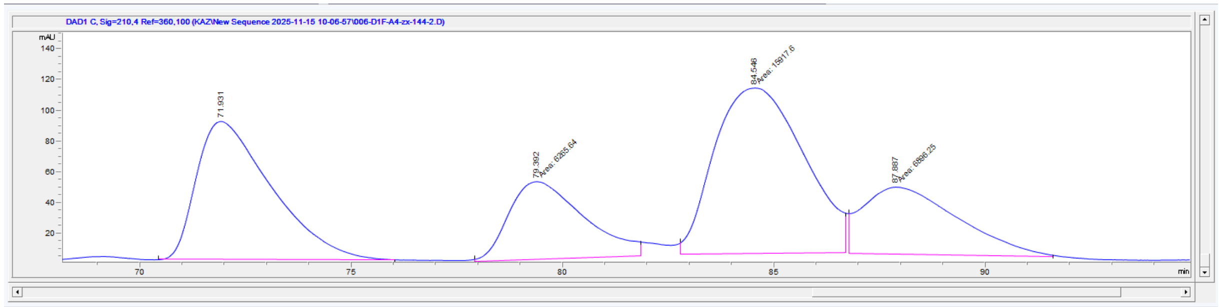

#	Time	Type	Area	Height	Width	Area%	Symmetry
1	71.931	BB	11093.9	90	1.4446	27.615	0.493
2	79.392	MM	6265.6	50.7	2.0604	15.596	0.59
3	84.546	MM	15917.6	108	2.4572	39.622	0.79
4	87.887	MM	6896.3	43.8	2.6231	17.166	0.523

CHIRALPAK® IE column, 2% *iso*-propanol, 98% hexane; t_R1 = 71.93 min, t_R2 = 79.39 min, t_R1^* = 84.55 min, t_R2^* = 87.89 min.

tert-butyl 5-amino-4-chloro-5-oxo-2-phenylpentanoate (**39**):

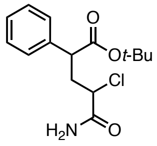

39

Procedure A:

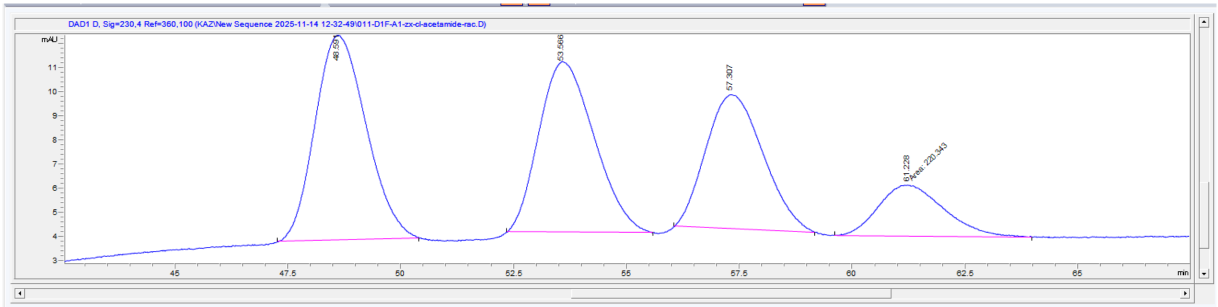

#	Time	Type	Area	Height	Width	Area%	Symmetry
1	48.591	BB	672.8	8.5	0.9234	33.795	0.769
2	53.566	BB	604.2	7.1	0.9958	30.349	0.709
3	57.307	BB	493.5	5.6	1.0383	24.788	0.759
4	61.228	MM	220.3	2.1	1.7158	11.068	0.771

CHIRALPAK® IG column, 2% *iso*-propanol, 98% hexane; t_R1 = 48.59 min, t_R2 = 53.57 min, t_R1^* = 57.31 min, t_R2^* = 61.23 min.

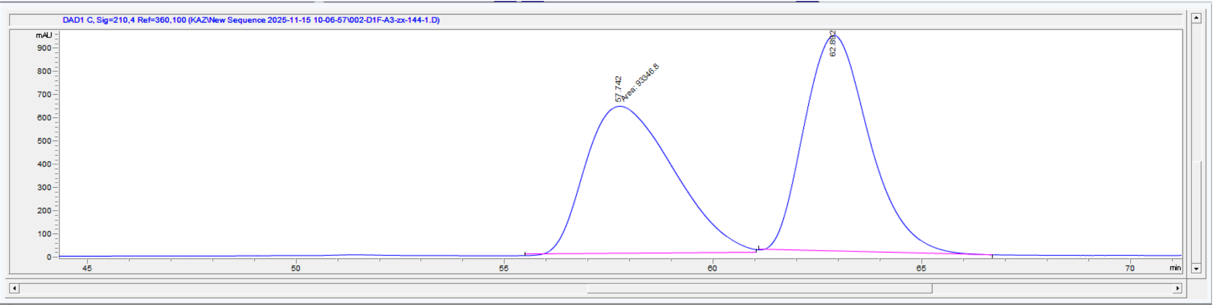

#	Time	Type	Area	Height	Width	Area%	Symmetry
1	57.742	MM	93346.8	634.9	2.4505	48.153	0.604
2	62.892	BB	100508.6	929.7	1.2803	51.847	0.796

CHIRALPAK® IG column, 2% *iso*-propanol, 98% hexane; t_R1^* = 57.74 min, t_R2^* = 62.89 min.

tert-butyl 5-amino-5-oxo-2-(pyridin-3-yl)pentanoate (**40**):

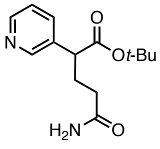

40

<u>Procedure A</u>:

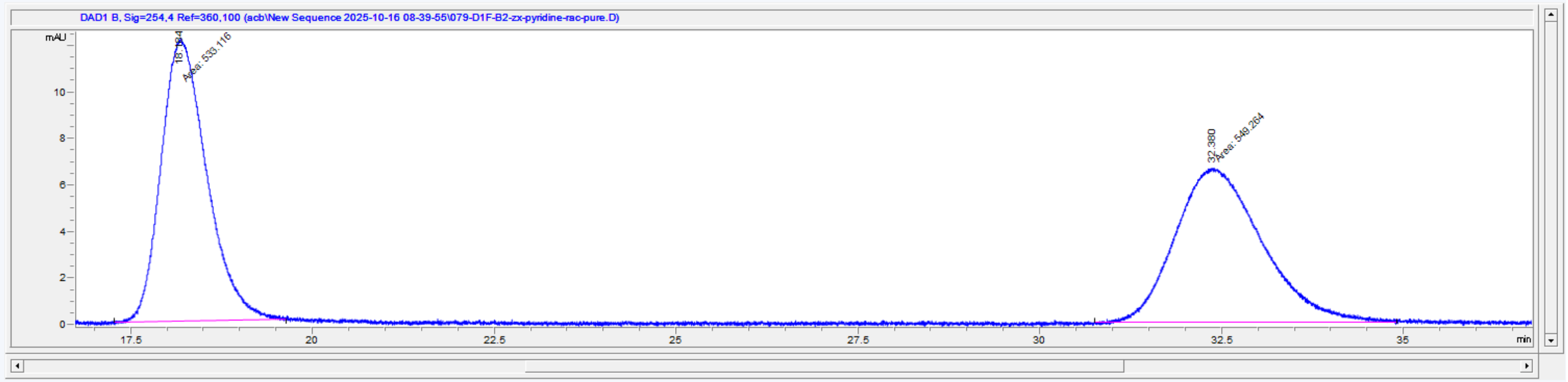

#	Time	Type	Area	Height	Width	Area%	Symmetry
1	18.184	MM	533.1	12.2	0.7296	49.254	0.742
2	32.38	MM	549.3	6.7	1.3761	50.746	0.713

CHIRALPAK® IG column, 20% *iso*-propanol, 80% hexane; t_R1 = 18.18 min, t_R2 = 32.38 min.

tert-butyl 5-amino-2-(6-methylpyridin-3-yl)-5-oxopentanoate (**41**):

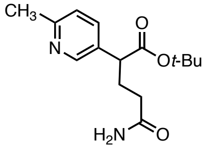

41

<u>Procedure A</u>:

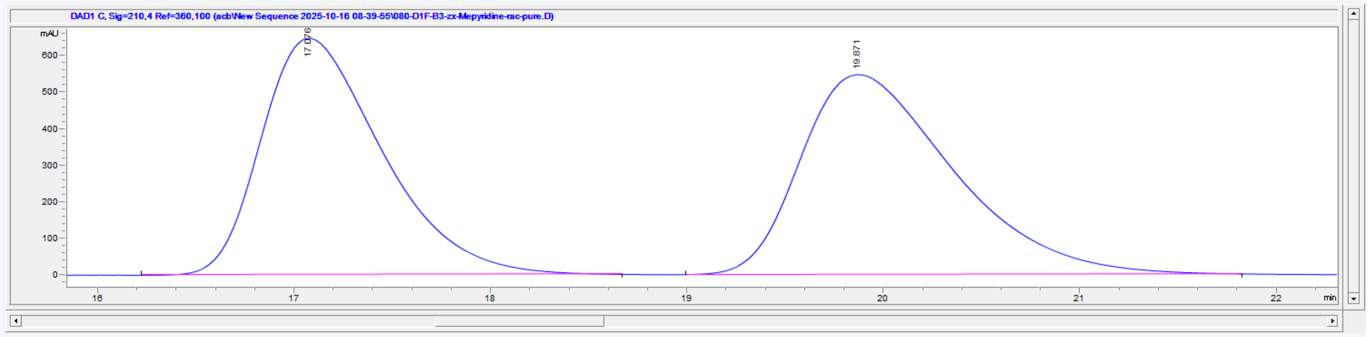

#	Time	Type	Area	Height	Width	Area%	Symmetry
1	17.076	BB R	27780.3	650.6	0.4992	48.428	0.649
2	19.871	BB R	29584.3	549.1	0.6301	51.572	0.602

CHIRALPAK® IG column, 20% *iso*-propanol, 80% hexane; t_R1 = 17.08 min, t_R2 = 19.87 min.

<u>Procedure B</u>:

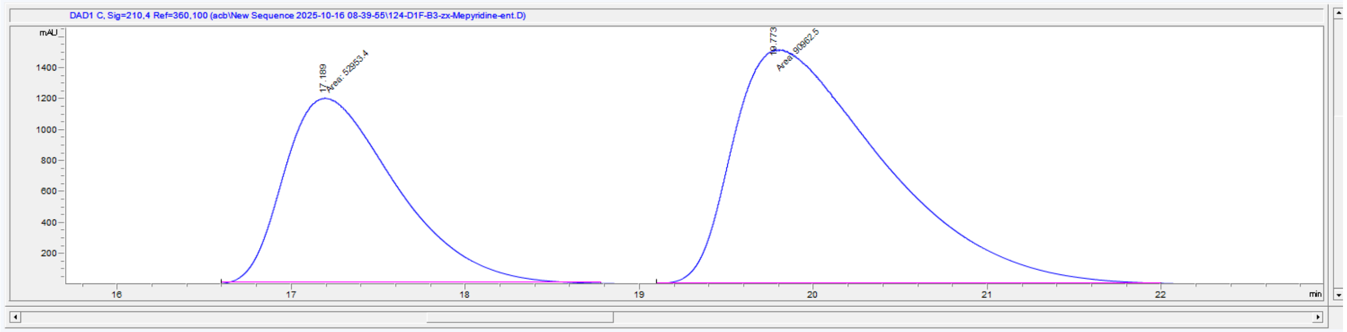

#	Time	Type	Area	Height	Width	Area%	Symmetry
1	17.189	MM	52953.4	1190.9	0.7411	36.795	0.556
2	19.773	MM	90962.5	1513.6	1.0016	63.205	0.381

CHIRALPAK® IG column, 20% *iso*-propanol, 80% hexane; t_R1 = 17.19 min, t_R2 = 19.77 min.

tert-butyl 5-amino-2-(6-methoxypyridin-3-yl)-5-oxopentanoate (**42**):

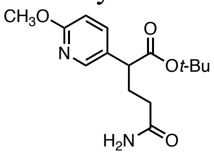

42

<u>Procedure A</u>:

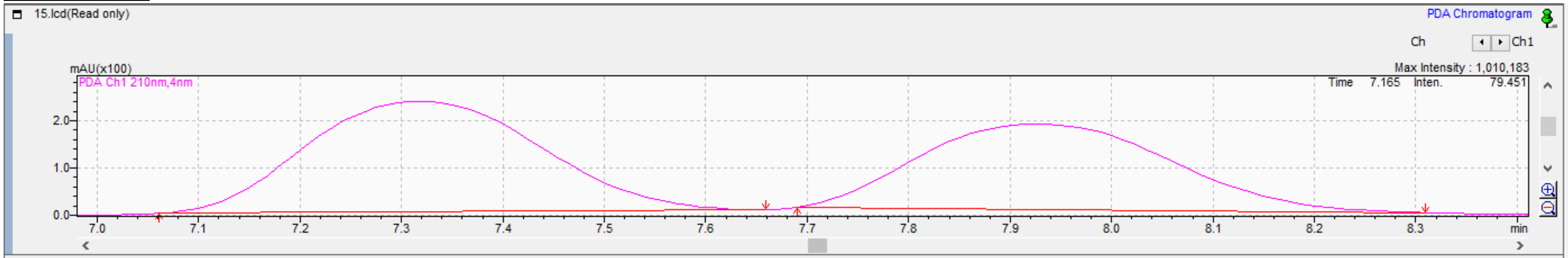

15.lcd(Read only) PDA Peak Table

Peak#	Ret. Time	Area	Area%
1	7.319	3738594	54.729
2	7.927	3092502	45.271
Total		6831095	100.000

CHIRALCEL® OX-3R column, 30% acetonitrile, 70% water; t_R1 = 7.32 min, t_R2 = 7.93 min.

<u>Procedure B</u>:

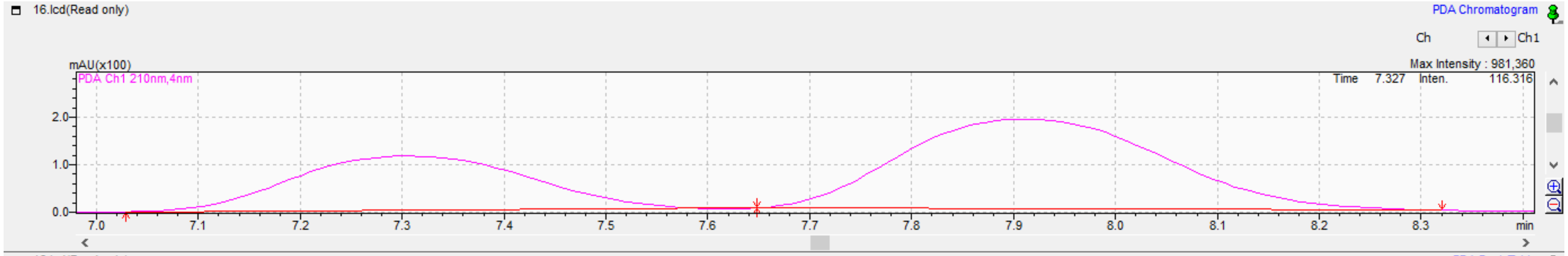

16.lcd(Read only) PDA Peak Table

Peak#	Ret. Time	Area	Area%
1	7.304	1816877	35.147
2	7.911	3352521	64.853
Total		5169399	100.000

CHIRALCEL® OX-3R column, 30% acetonitrile, 70% water; t_R1 = 7.30 min, t_R2 = 7.91 min.

tert-butyl 5-amino-2-(6-chloropyridin-3-yl)-5-oxopentanoate (**43**):

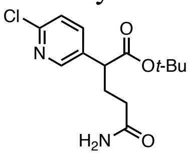

43

<u>Procedure A</u>:

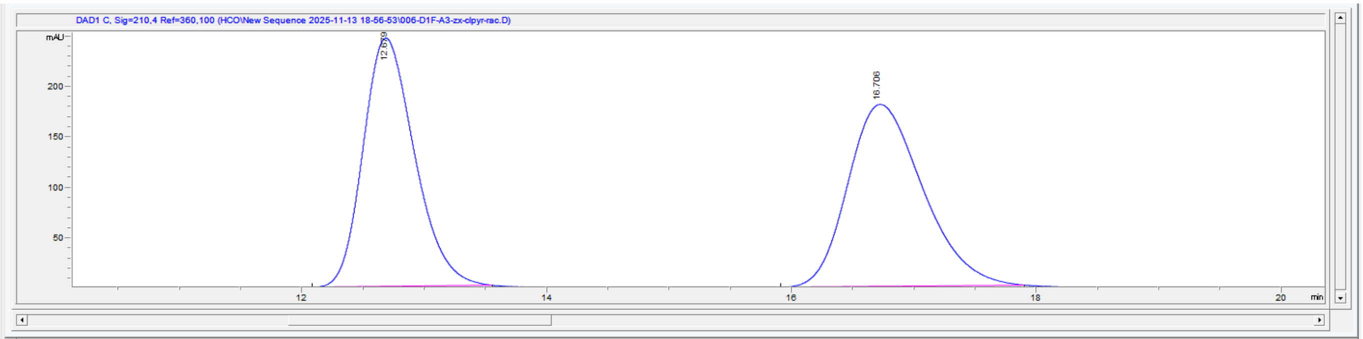

#	Time	Type	Area	Height	Width	Area%	Symmetry
1	12.679	BB R	7171.8	246.3	0.3412	48.293	0.754
2	16.706	BB R	7678.7	180.4	0.4971	51.707	0.704

CHIRALPAK® IG column, 20% *iso*-propanol, 80% hexane; t_R1 = 12.68 min, t_R2 = 16.71 min.

<u>Procedure B</u>:

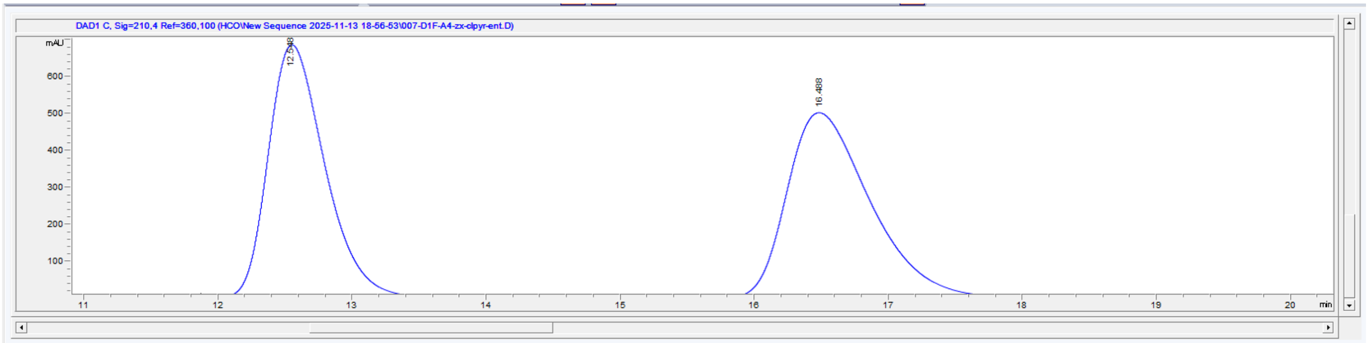

#	Time	Type	Area	Height	Width	Area%	Symmetry
1	12.548	BB R	20153.1	687.9	0.3433	48.180	0.685
2	16.488	BB R	21675.9	504.4	0.5028	51.820	0.631

CHIRALPAK® IG column, 20% *iso*-propanol, 80% hexane; t_R1 = 12.55 min, t_R2 = 16.49 min.

tert-butyl 5-amino-5-oxo-2-(pyrimidin-2-yl)pentanoate (**44**):

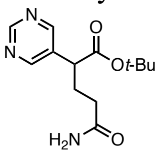

44

Procedure A:

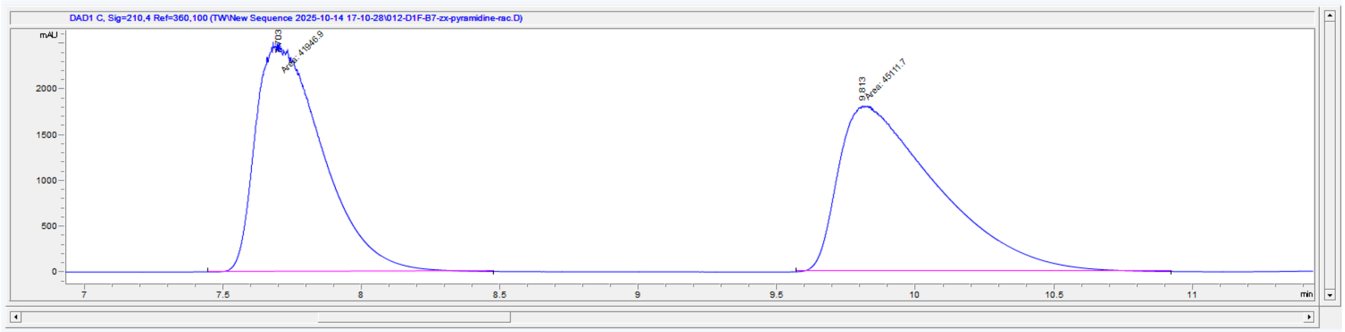

#	Time	Type	Area	Height	Width	Area%	Symmetry
1	7.703	MM	41946.9	2511.5	0.2784	48.182	0.33
2	9.813	MM	45111.7	1817.4	0.4137	51.818	0.289

CHIRALPAK® IE column, 50% *iso*-propanol, 50% hexane; t_R1 = 7.70 min, t_R2 = 9.81 min.

Procedure B:

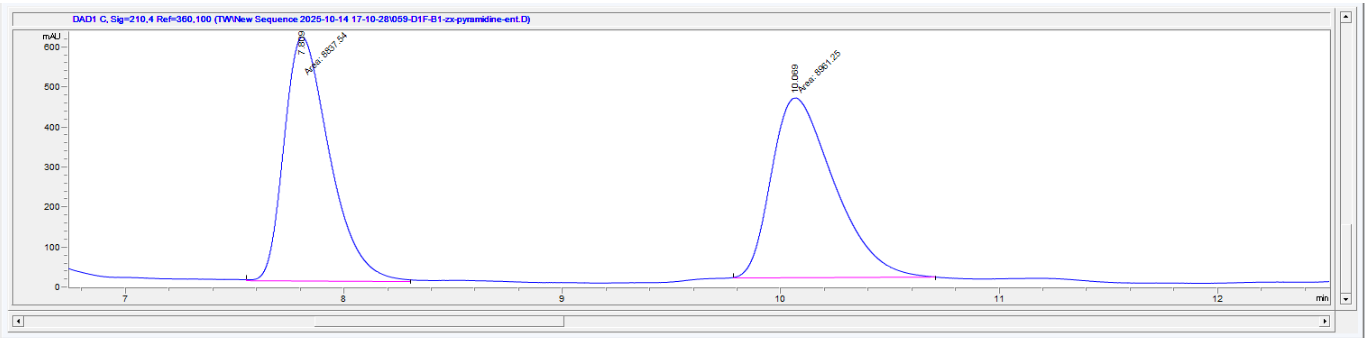

#	Time	Type	Area	Height	Width	Area%	Symmetry
1	7.809	MM	8837.5	609.4	0.2417	49.652	0.585
2	10.069	MM	8961.3	448.9	0.3327	50.348	0.581

CHIRALPAK® IE column, 50% *iso*-propanol, 50% hexane; t_R1 = 7.81 min, t_R2 = 10.07 min.

tert-butyl 5-amino-2-(naphthalen-2-yl)-5-oxopentanoate (**45**):

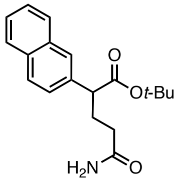

45

Procedure A:

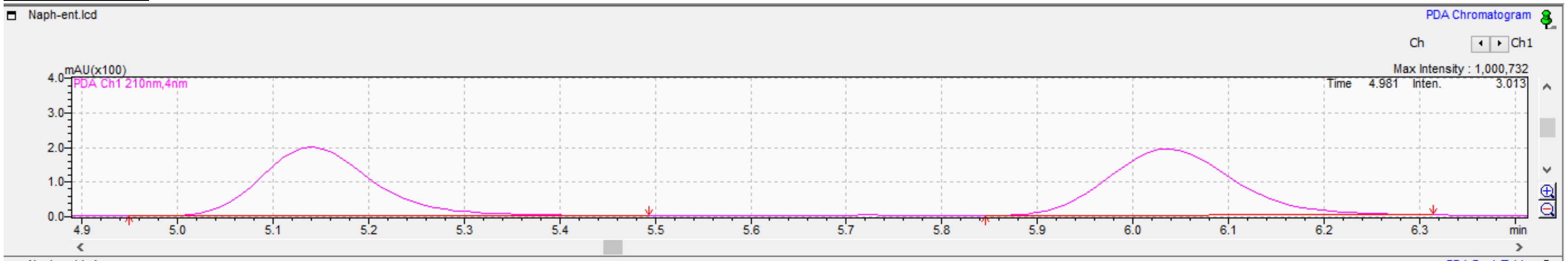

Naph-ent.lcd PDA Peak Table

Peak#	Ret. Time	Area	Area%
1	5.140	1624777	47.800
2	6.036	1774365	52.200
Total		3399142	100.000

CHIRALCEL® OX-3R column, 30% acetonitrile, 70% water; t_R1 = 5.14 min, t_R2 = 6.04 min.

Procedure B:

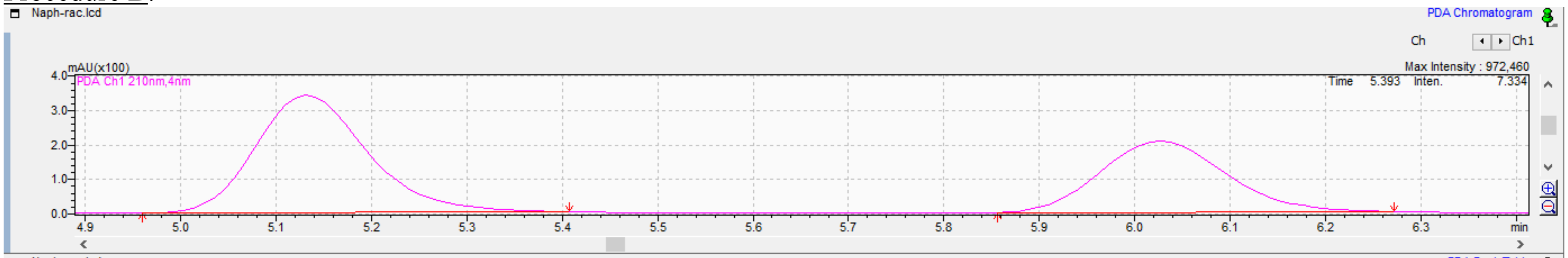

Naph-rac.lcd PDA Peak Table

Peak#	Ret. Time	Area	Area%
1	5.132	2795605	59.611
2	6.026	1894105	40.389
Total		4689711	100.000

CHIRALCEL® OX-3R column, 30% acetonitrile, 70% water; t_R1 = 5.13 min, t_R2 = 6.03 min.

tert-butyl 5-amino-5-oxo-2-(quinolin-3-yl)pentanoate (**46**):

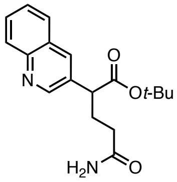

46

Procedure A:

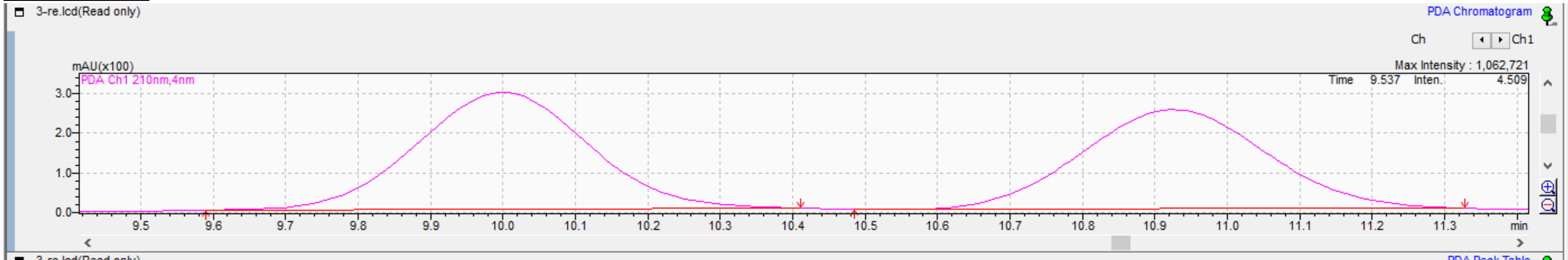

3-re.lcd(Read only) PDA Peak Table

Peak#	Ret. Time	Area	Area%
1	10.000	4864278	52.244
2	10.926	4446428	47.756
Total		9310706	100.000

Chiral HPLC method: CHIRALCEL® OX-3R column, 20% acetonitrile, 80% water; t_R1 = 10.00 min, t_R2 = 10.93 min.

Procedure B:

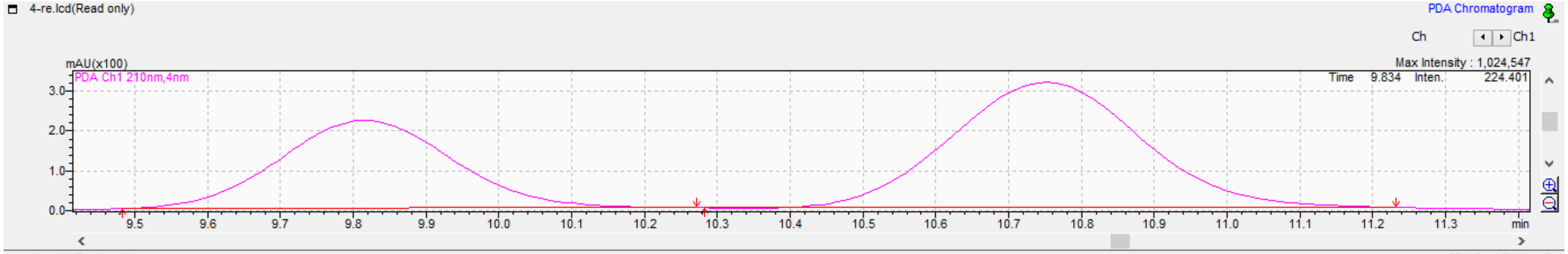

4-re.lcd(Read only) PDA Peak Table

Peak#	Ret. Time	Area	Area%
1	9.816	3669795	38.948
2	10.752	5752588	61.052
Total		9422383	100.000

Chiral HPLC method: CHIRALCEL® OX-3R column, 20% acetonitrile, 80% water; t_R1 = 9.82 min, t_R2 = 10.75 min.

tert-butyl 5-amino-5-oxo-2-(quinolin-4-yl)pentanoate (**47**):

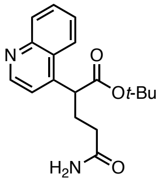

47

<u>Procedure A</u>:

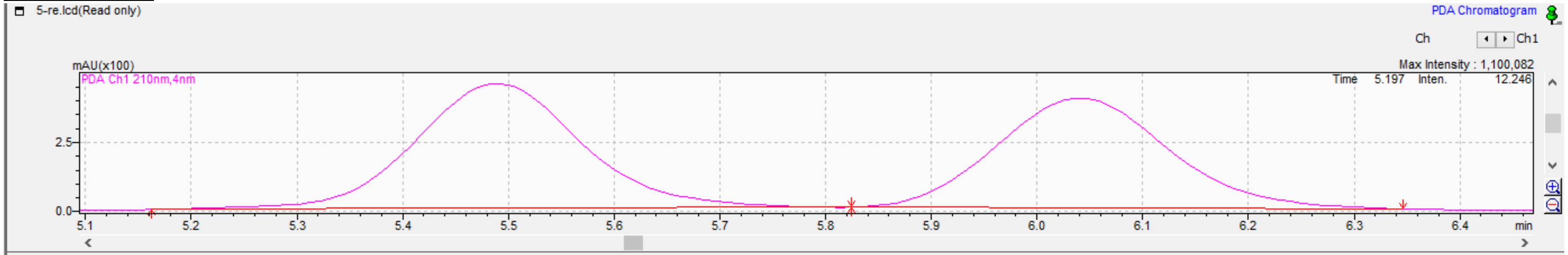

5-re.lcd(Read only) PDA Peak Table

Peak#	Ret. Time	Area	Area%
1	5.489	4919897	52.434
2	6.041	4463196	47.566
Total		9383092	100.000

CHIRALCEL® OX-3R column, 20% acetonitrile, 80% water; t_R1 = 5.49 min, t_R2 = 6.04 min.

<u>Procedure B</u>:

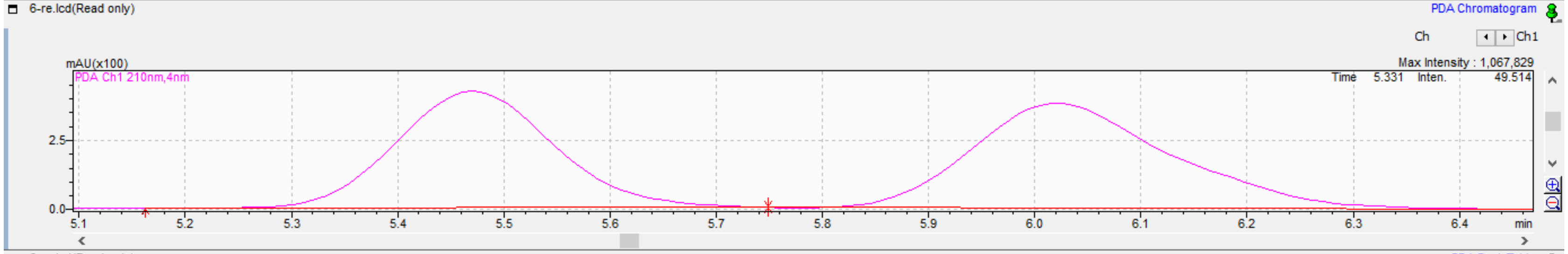

6-re.lcd(Read only) PDA Peak Table

Peak#	Ret. Time	Area	Area%
1	5.471	4331905	46.936
2	6.022	4897513	53.064
Total		9229418	100.000

CHIRALCEL® OX-3R column, 20% acetonitrile, 80% water; t_R1 = 5.47 min, t_R2 = 6.02 min.

tert-butyl 5-amino-5-oxo-2-(quinolin-5-yl)pentanoate (**48**):

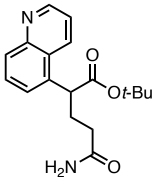

48

Procedure A:

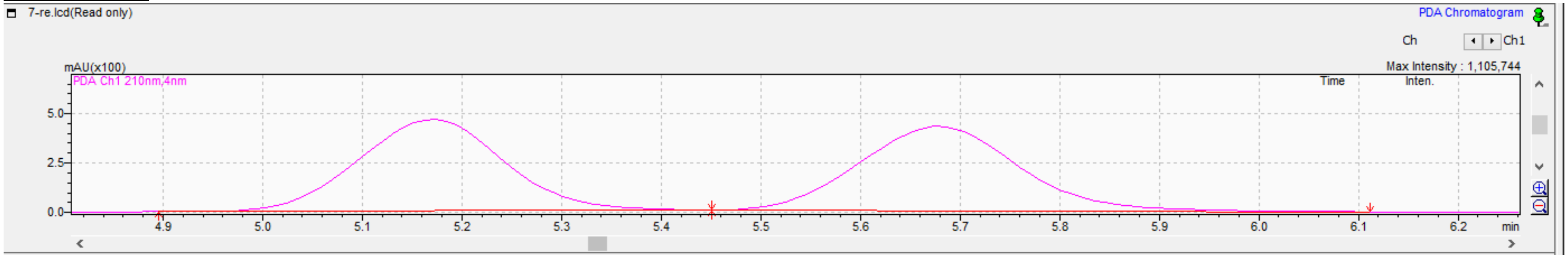

7-re.lcd(Read only) PDA Peak Table

Peak#	Ret. Time	Area	Area%
1	5.170	4695753	49.635
2	5.679	4764837	50.365
Total		9460590	100.000

CHIRALCEL® OX-3R column, 20% acetonitrile, 80% water; t_R1 = 5.17 min, t_R2 = 5.68 min.

Procedure B:

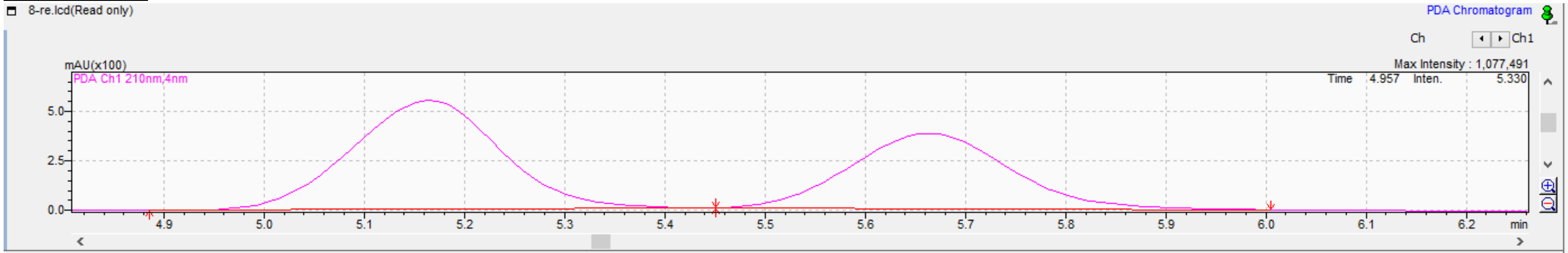

8-re.lcd(Read only) PDA Peak Table

Peak#	Ret. Time	Area	Area%
1	5.165	5668583	57.605
2	5.664	4171866	42.395
Total		9840449	100.000

CHIRALCEL® OX-3R column, 20% acetonitrile, 80% water; t_R1 = 5.17 min, t_R2 = 5.66 min.

tert-butyl 5-amino-2-(1-methyl-1*H*-indol-6-yl)-5-oxopentanoate (**49**):

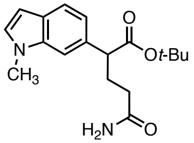

49

Procedure A:

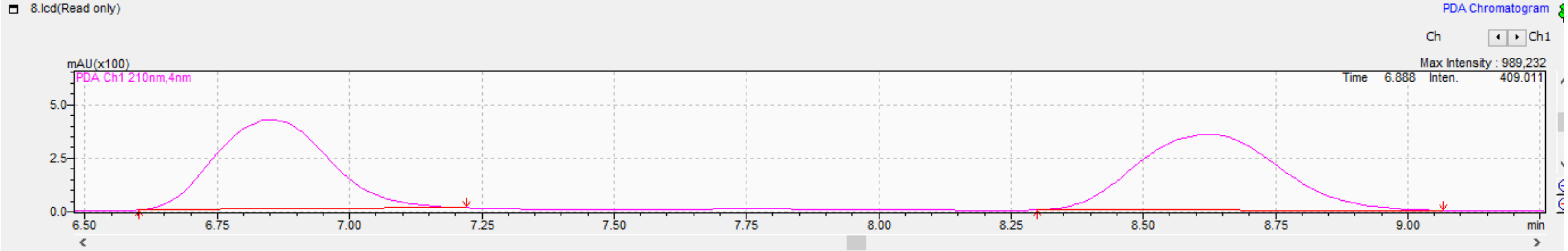

8.lcd(Read only) PDA Peak Table

Peak#	Ret. Time	Area	Area%
1	6.853	6235553	48.511
2	8.622	6618256	51.489
Total		12853809	100.000

CHIRALCEL® OX-3R column, 45% acetonitrile, 55% water; t_R1 = 6.85 min, t_R2 = 8.62 min.

Procedure B:

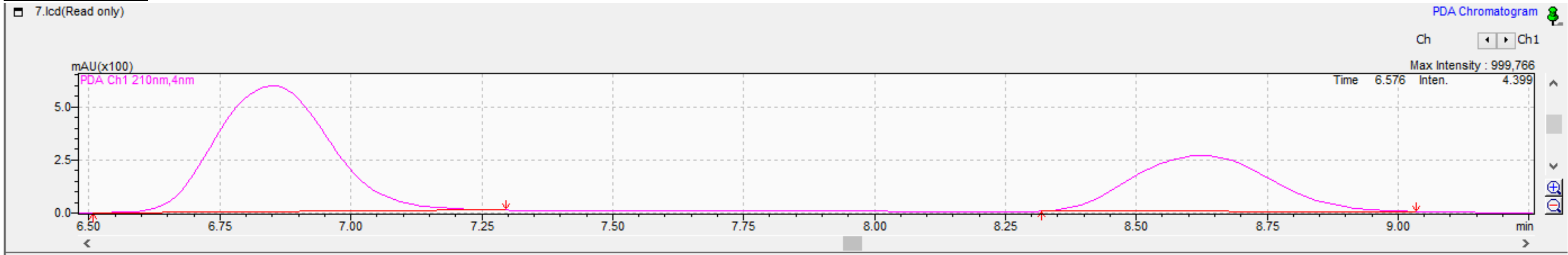

7.lcd(Read only) PDA Peak Table

Peak#	Ret. Time	Area	Area%
1	6.850	8986011	65.042
2	8.624	4829779	34.958
Total		13815790	100.000

CHIRALCEL® OX-3R column, 45% acetonitrile, 55% water; t_R1 = 6.85 min, t_R2 = 8.62 min.

tert-butyl 5-amino-2-(1-methyl-1*H*-indazol-6-yl)-5-oxopentanoate (**50**):

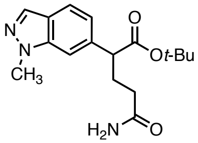

50

Procedure A:

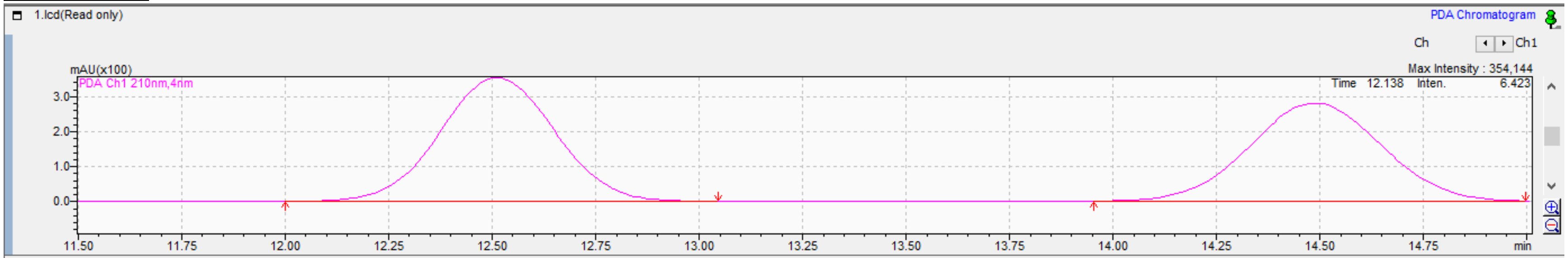

1.lcd(Read only) PDA Peak Table

Peak#	Ret. Time	Area	Area%
1	12.511	6852258	52.343
2	14.487	6238917	47.657
Total		13091176	100.000

CHIRALCEL® OX-3R column, 30% acetonitrile, 70% water; t_R1 = 12.51 min, t_R2 = 14.49 min.

Procedure B:

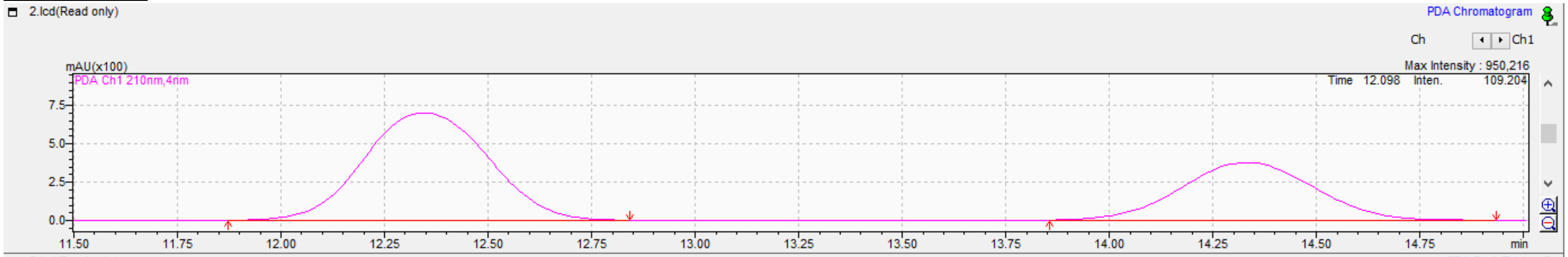

2.lcd(Read only) PDA Peak Table

Peak#	Ret. Time	Area	Area%
1	12.345	14595814	63.759
2	14.331	8296252	36.241
Total		22892067	100.000

CHIRALCEL® OX-3R column, 30% acetonitrile, 70% water; t_R1 = 12.35 min, t_R2 = 14.33 min.

tert-butyl 5-amino-2-(3-methyl-2-oxo-2,3-dihydrobenzo[*d*]oxazol-6-yl)-5-oxopentanoate (**51**):

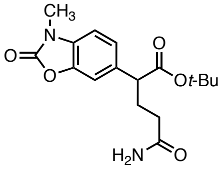

51

Procedure A:

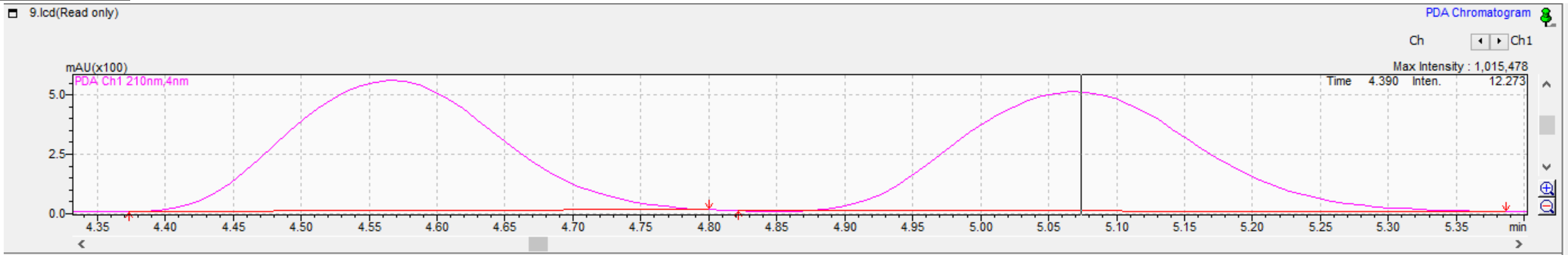

9.lcd(Read only) PDA Peak Table

Peak#	Ret. Time	Area	Area%
1	4.566	5949394	49.465
2	5.069	6077992	50.535
Total		12027386	100.000

CHIRALCEL® OX-3R column, 45% acetonitrile, 55% water; t_R1 = 4.57 min, t_R2 = 5.07 min.

Procedure B:

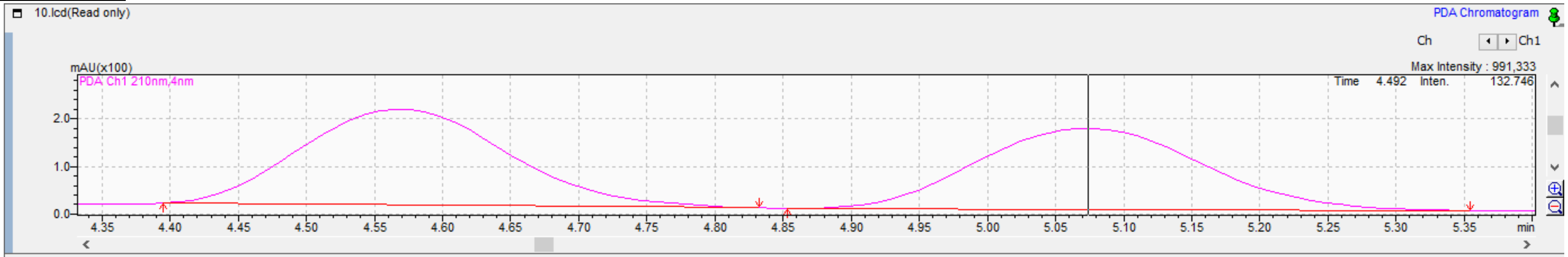

10.lcd(Read only) PDA Peak Table

Peak#	Ret. Time	Area	Area%
1	4.570	2123508	52.029
2	5.074	1957853	47.971
Total		4081361	100.000

CHIRALCEL® OX-3R column, 45% acetonitrile, 55% water; t_R1 = 4.57 min, t_R2 = 5.07 min.

tert-butyl 5-amino-5-oxo-2-(1,3,3-trimethyl-2-oxoindolin-6-yl)pentanoate (**52**):

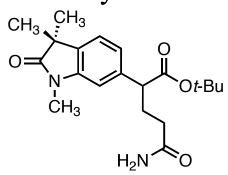

52

Procedure A:

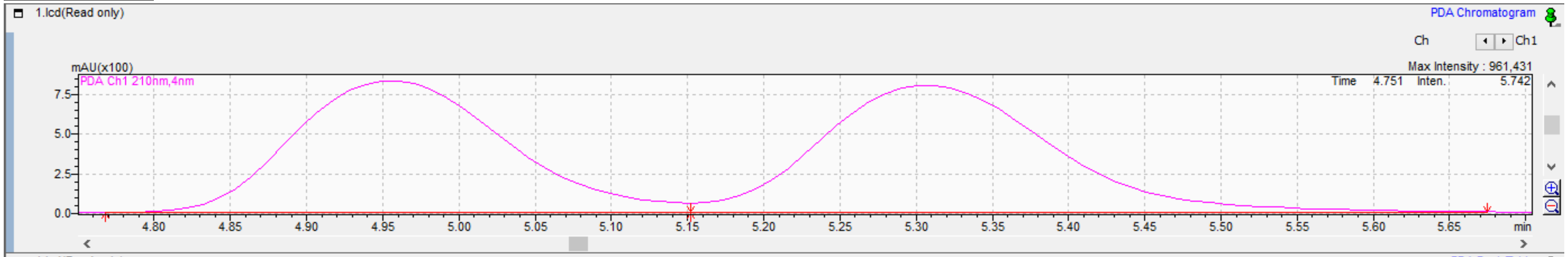

1.lcd(Read only) PDA Peak Table

Peak#	Ret. Time	Area	Area%
1	4.955	8063624	48.783
2	5.307	8465850	51.217
Total		16529473	100.000

Chiral HPLC method: CHIRALCEL® OX-3R column, 45% acetonitrile, 55% water; t_R1 = 4.96 min, t_R2 = 5.31 min.

Procedure B:

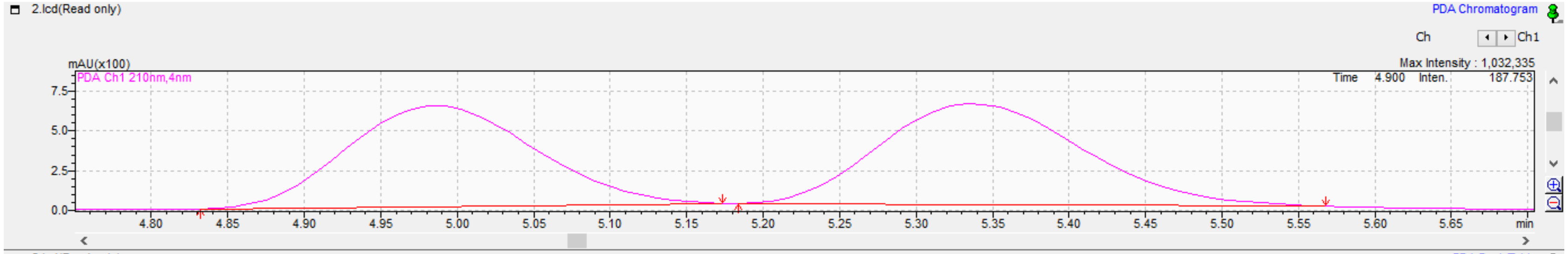

2.lcd(Read only) PDA Peak Table

Peak#	Ret. Time	Area	Area%
1	4.986	5465874	48.260
2	5.337	5859946	51.740
Total		11325820	100.000

Chiral HPLC method: CHIRALCEL® OX-3R column, 45% acetonitrile, 55% water; t_R1 = 4.99 min, t_R2 = 5.34 min.

3-phenylpiperidine-2,6-dione (**5**).

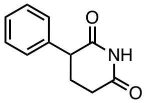

5

<u>Procedure A</u>:

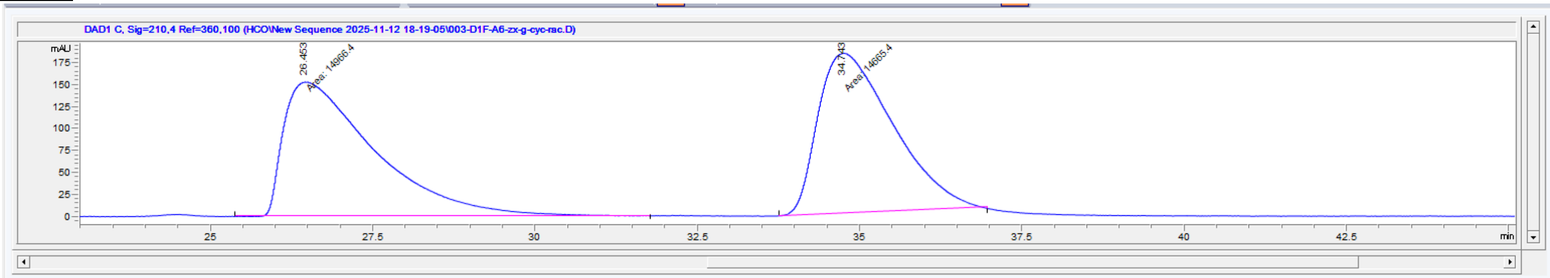

#	Time	Type	Area	Height	Width	Area%	Symmetry
1	26.453	MM	14966.4	154.5	1.6143	50.508	0.272
2	34.743	MM	14665.4	183.6	1.3311	49.492	0.483

CHIRALPAK® IH column, 20% *iso*-propanol, 80% hexane; t_R1 = 26.45 min, t_R2 = 34.74 min

<u>Procedure B</u>:

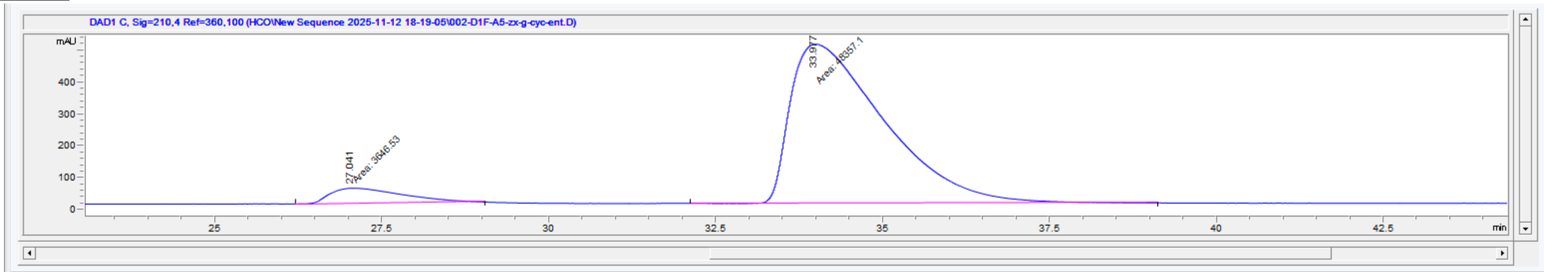

#	Time	Type	Area	Height	Width	Area%	Symmetry
1	27.041	MM	3646.5	48.2	1.2607	7.012	0.404
2	33.977	MM	48357.1	497.7	1.6194	92.988	0.305

CHIRALPAK® IH column, 20% *iso*-propanol, 80% hexane; t_R1 = 27.04 min, t_R2 = 33.98 min

12. Catalog of nuclear magnetic resonance spectra.

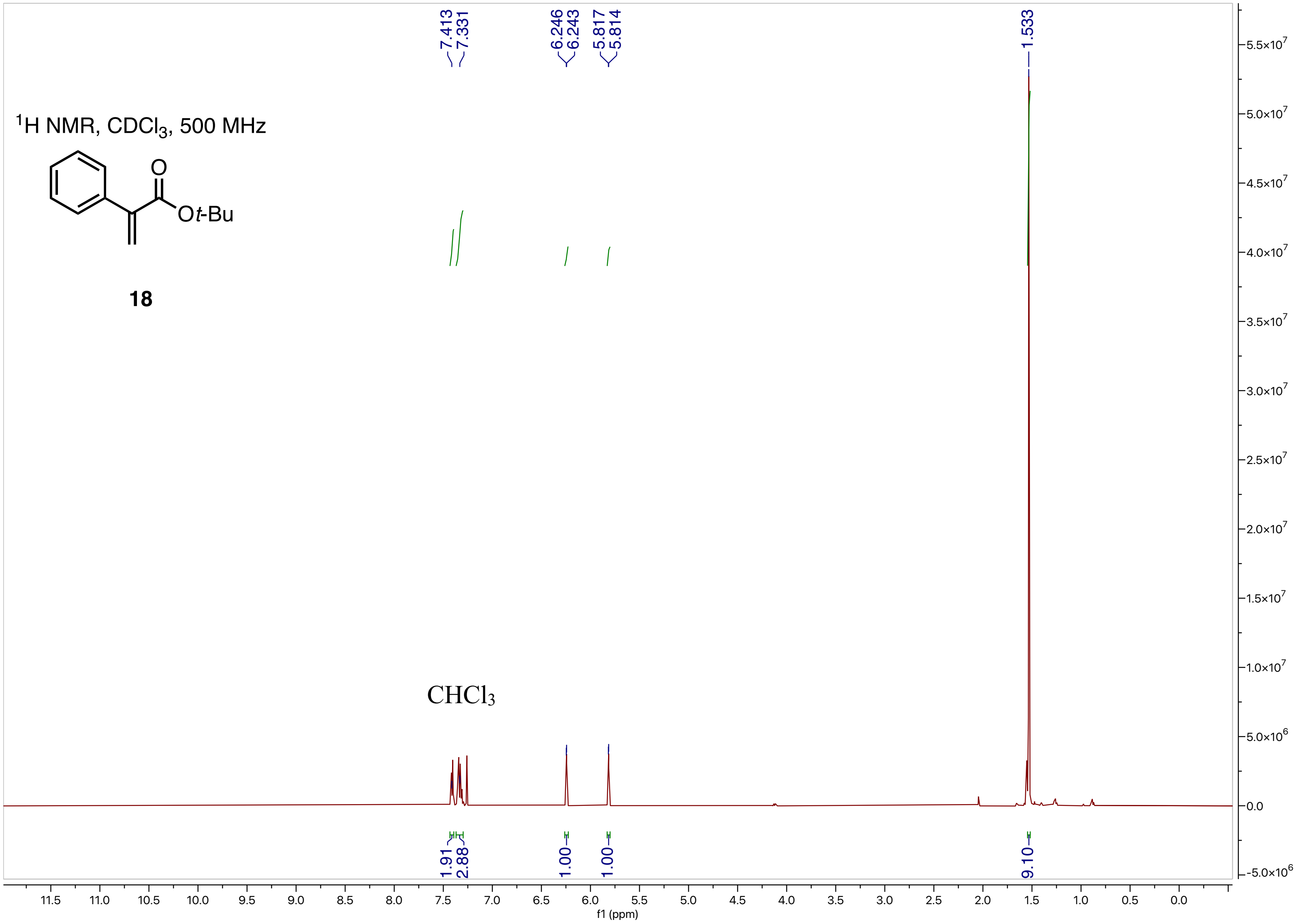

^1H NMR, $CDCl_3$, 500 MHz

21-s

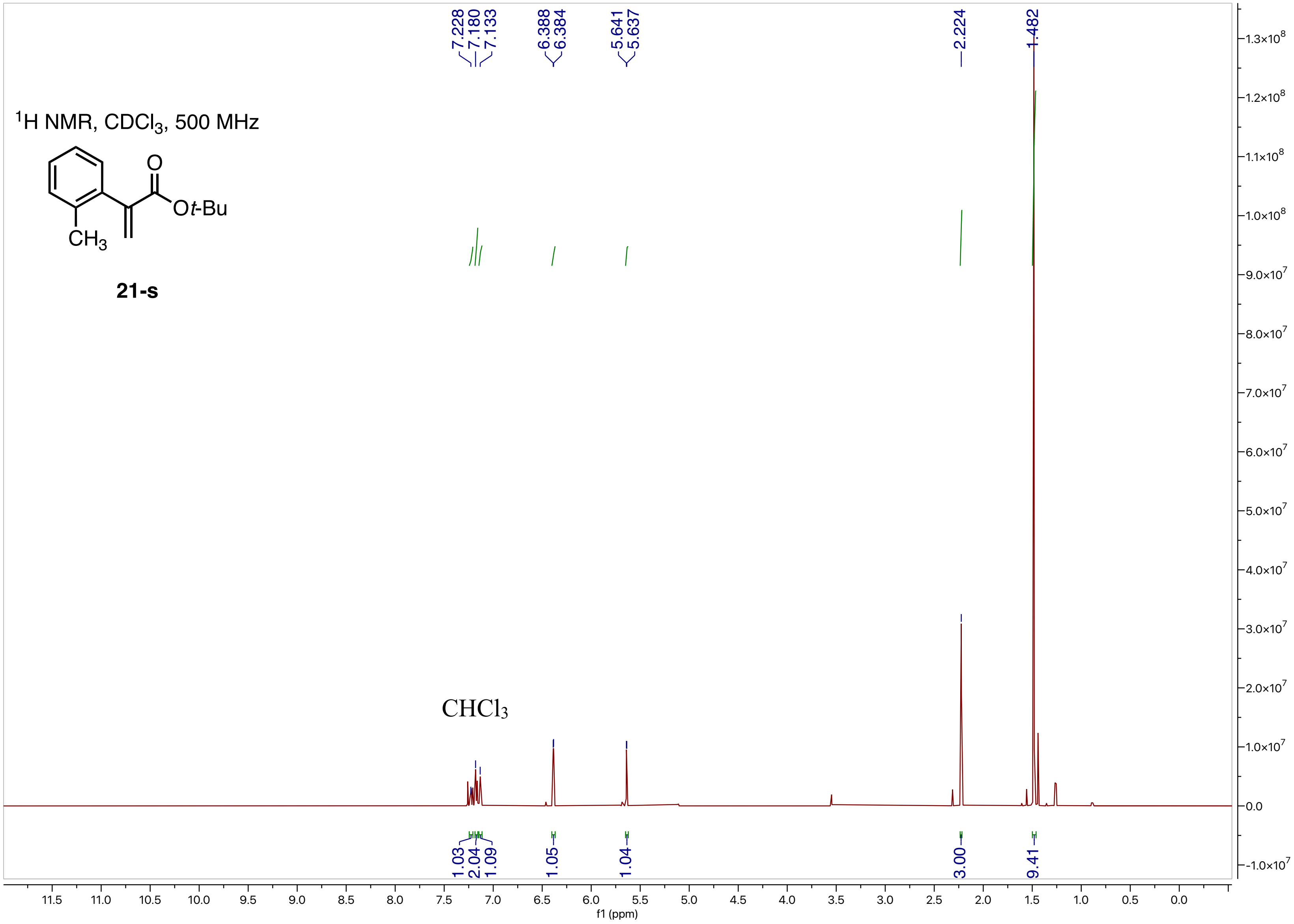

$^{13}C\{^{1}H\}$NMR, $CDCl_3$, 126 MHz

21-s

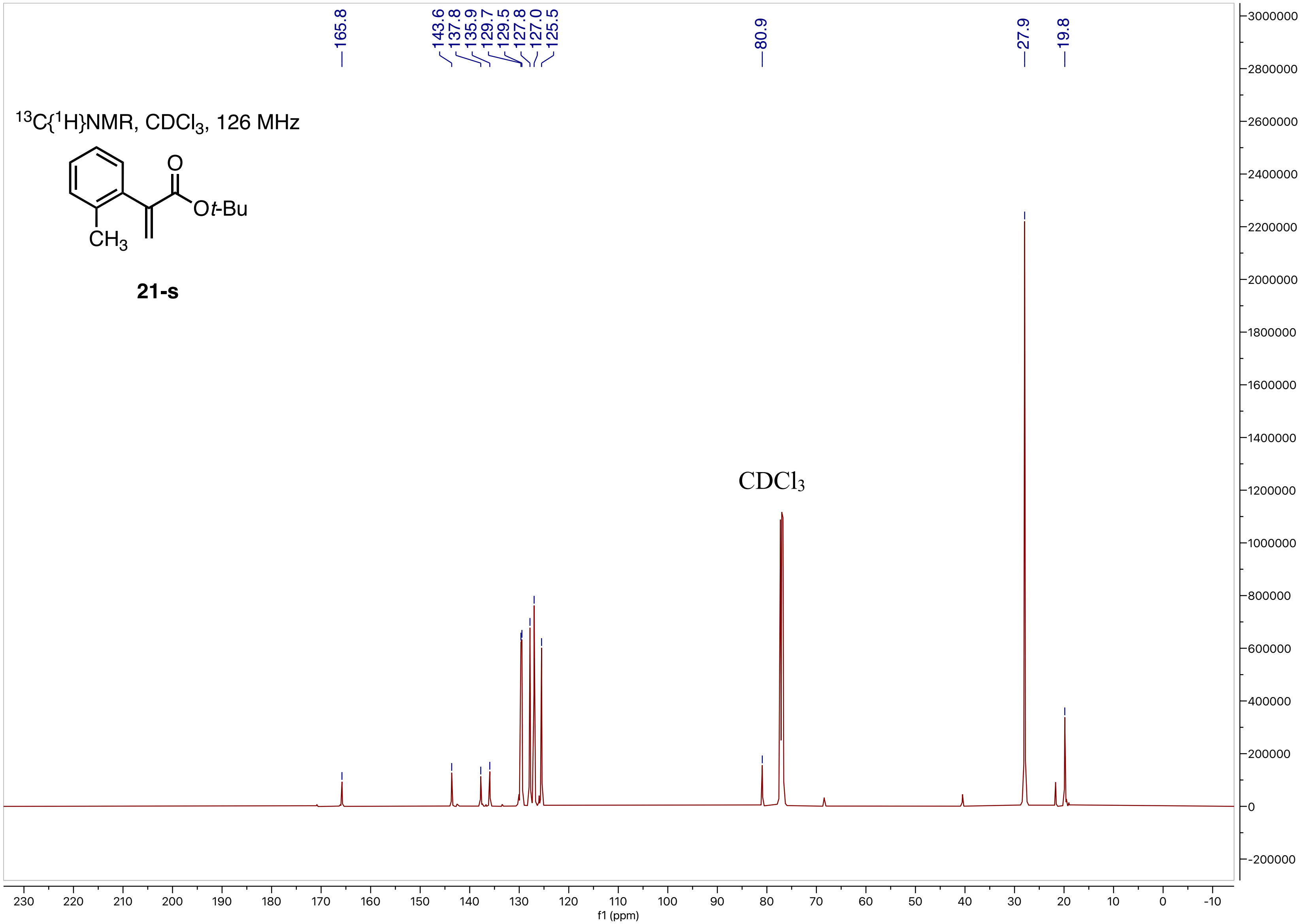

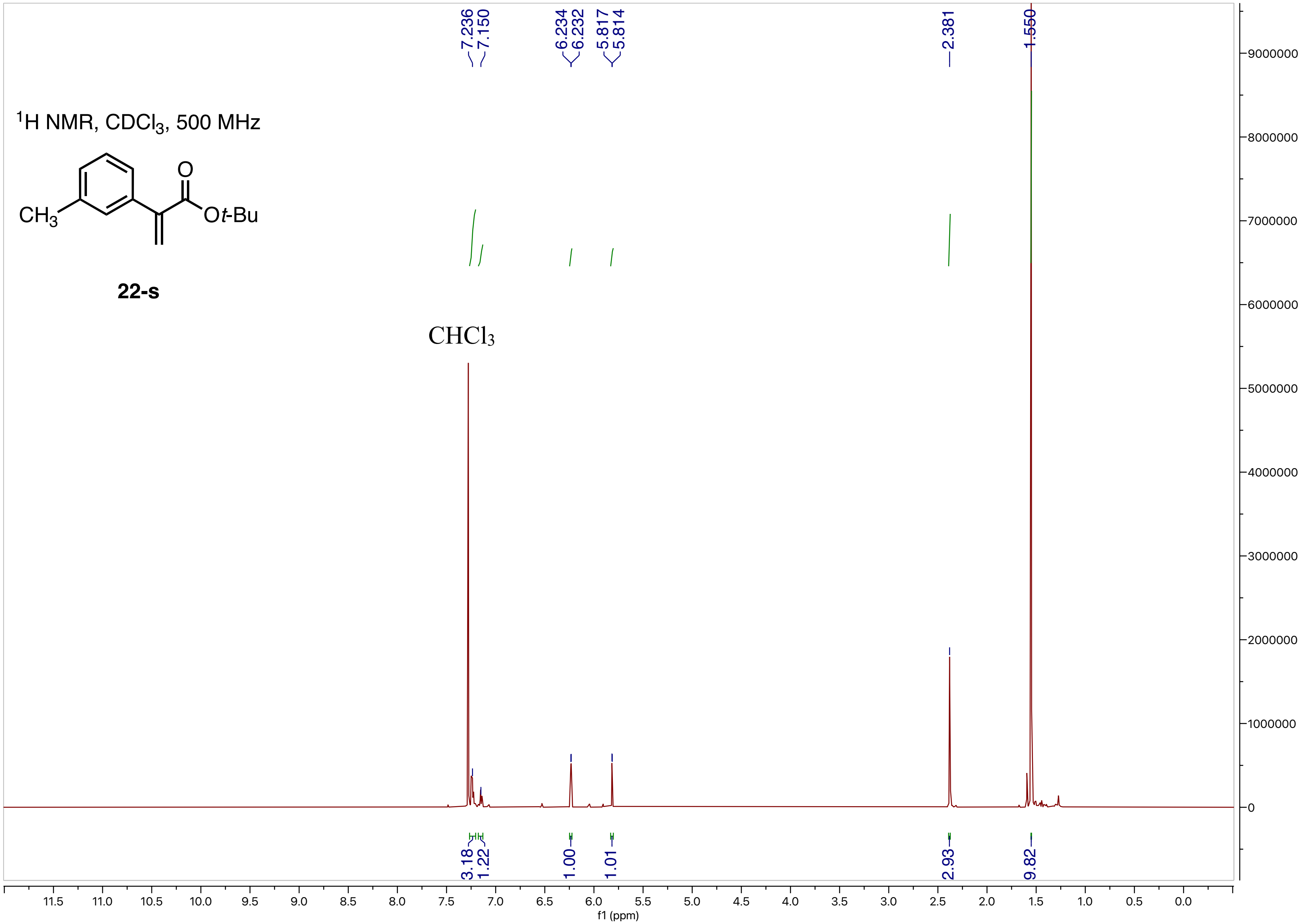

1H NMR, CDCl3, 500 MHz
CH3
O
Ot-Bu
22-s
CHCl3
7.236
7.150
6.234
6.232
5.817
5.814
2.381
1.550
3.18
1.22
1.00
1.01
2.93
9.82
f1 (ppm)

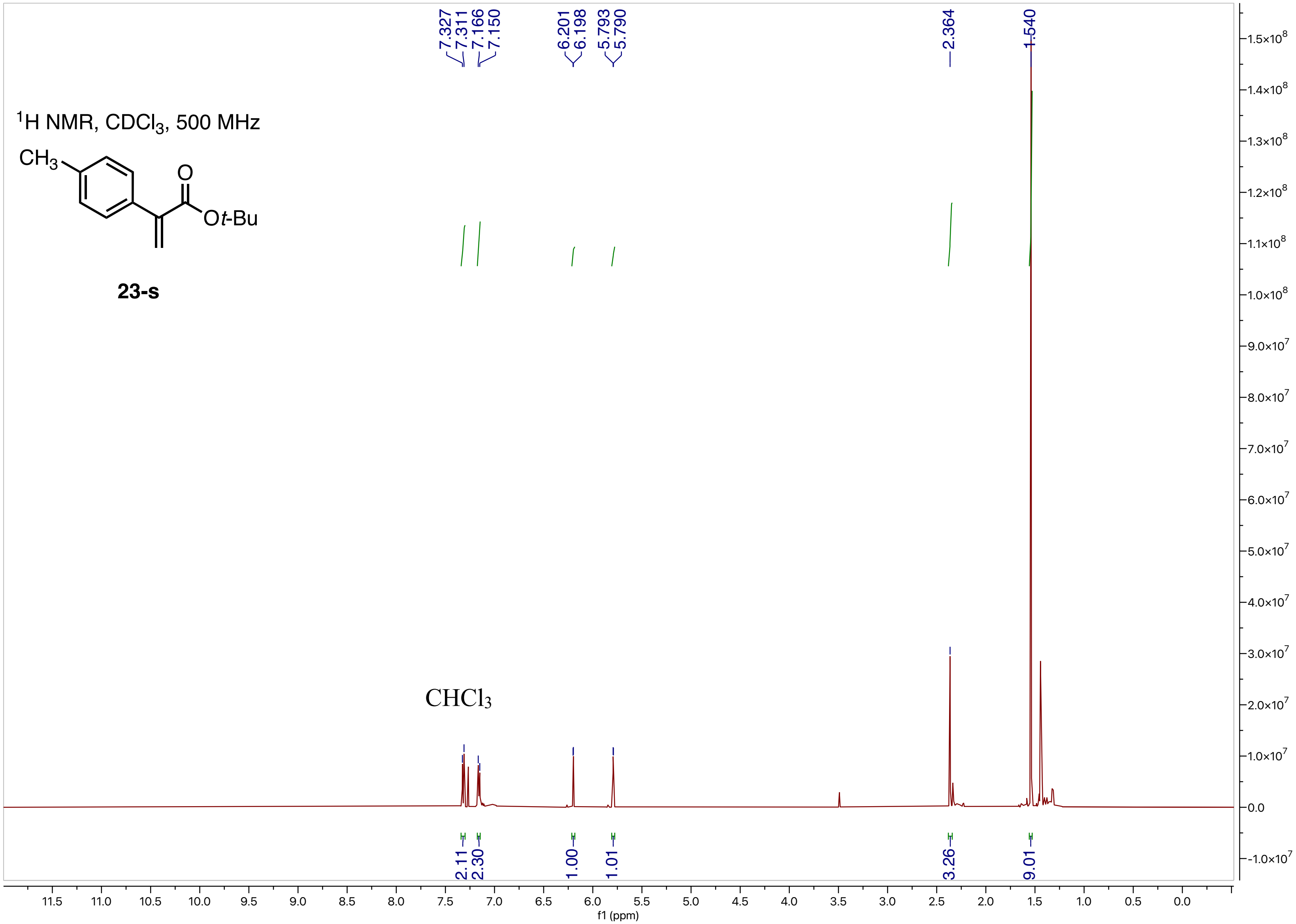

7.327
7.311
7.166
7.150
6.201
6.198
5.793
5.790
2.364
1.540
^{1}H NMR, CDCl3, 500 MHz
CH3
O
Ot-Bu
23-s
CHCl3
2.11
2.30
1.00
1.01
3.26
9.01
1.5x10[8]
1.4x10[8]
1.3x10[8]
1.2x10[8]
1.1x10[8]
1.0x10[8]
9.0x10[7]
8.0x10[7]
7.0x10[7]
6.0x10[7]
5.0x10[7]
4.0x10[7]
3.0x10[7]
2.0x10[7]
1.0x10[7]
0.0
-1.0x10[7]
11.5 11.0 10.5 10.0 9.5 9.0 8.5 8.0 7.5 7.0 6.5 6.0 5.5 5.0 4.5 4.0 3.5 3.0 2.5 2.0 1.5 1.0 0.5 0.0
f1 (ppm)

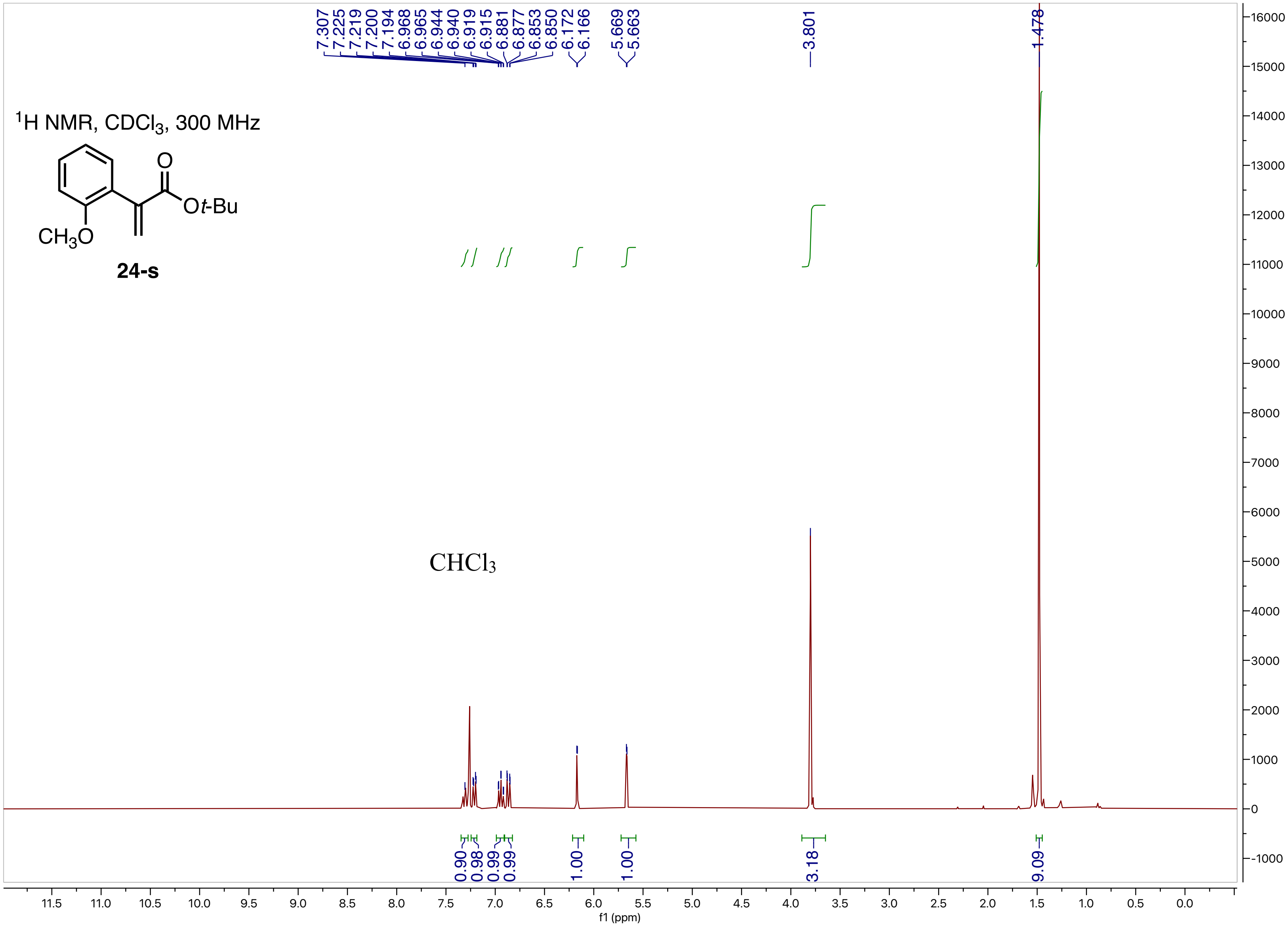

^{1}H NMR, $CDCl_3$, 300 MHz
O
O*t*-Bu
CH_3O
24-s
$CHCl_3$
7.307
7.225
7.219
7.200
7.194
6.968
6.965
6.944
6.940
6.919
6.915
6.881
6.877
6.853
6.850
6.172
6.166
5.669
5.663
3.801
1.478
0.90
0.98
0.99
0.99
1.00
1.00
3.18
9.09
f1 (ppm)

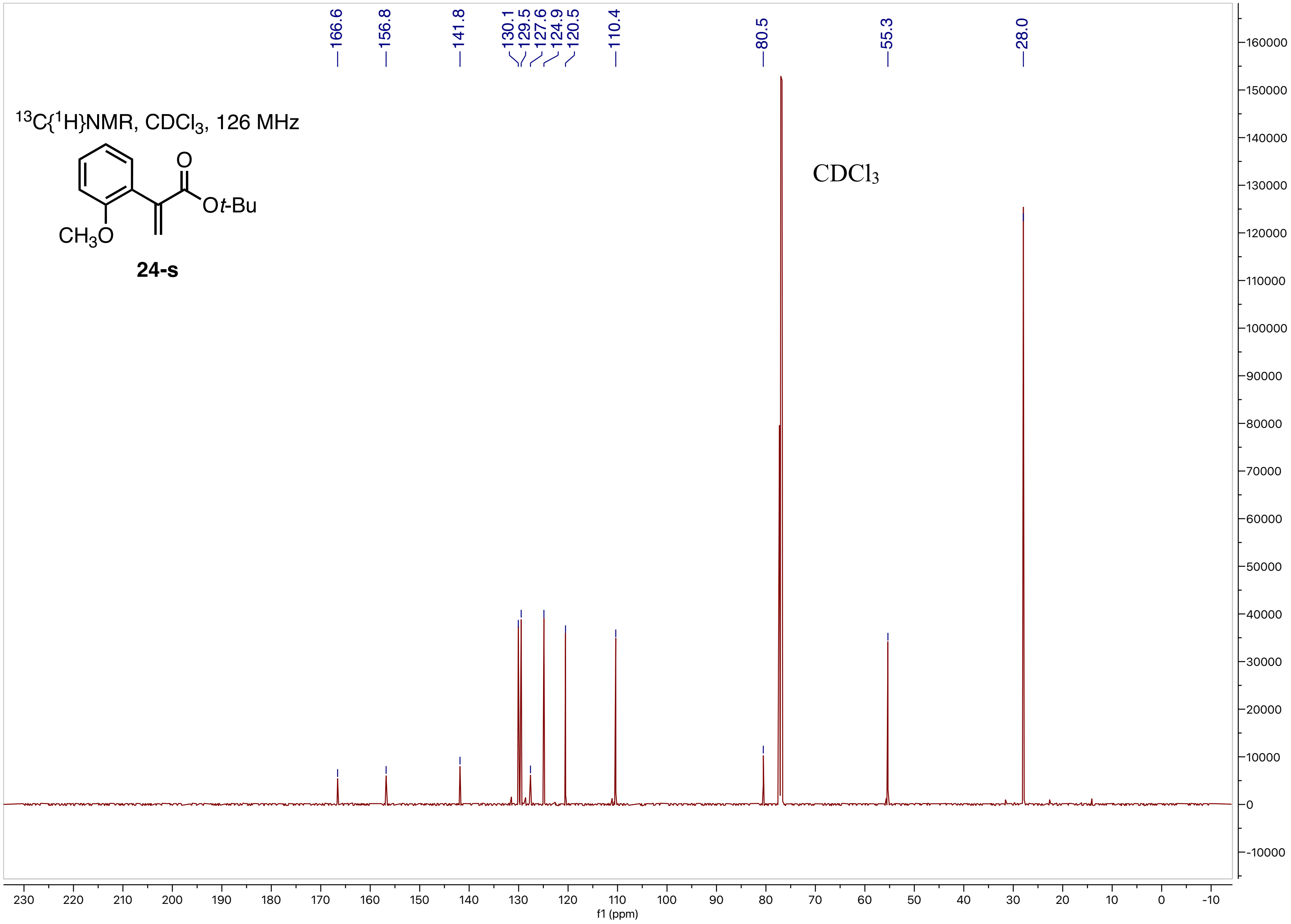

166.6
156.8
141.8
130.1
129.5
127.6
124.9
120.5
110.4
80.5
55.3
28.0
$^{13}C\{^1H\}$NMR, $CDCl_3$, 126 MHz
O
O*t*-Bu
CH_3O
24-s
$CDCl_3$
f1 (ppm)

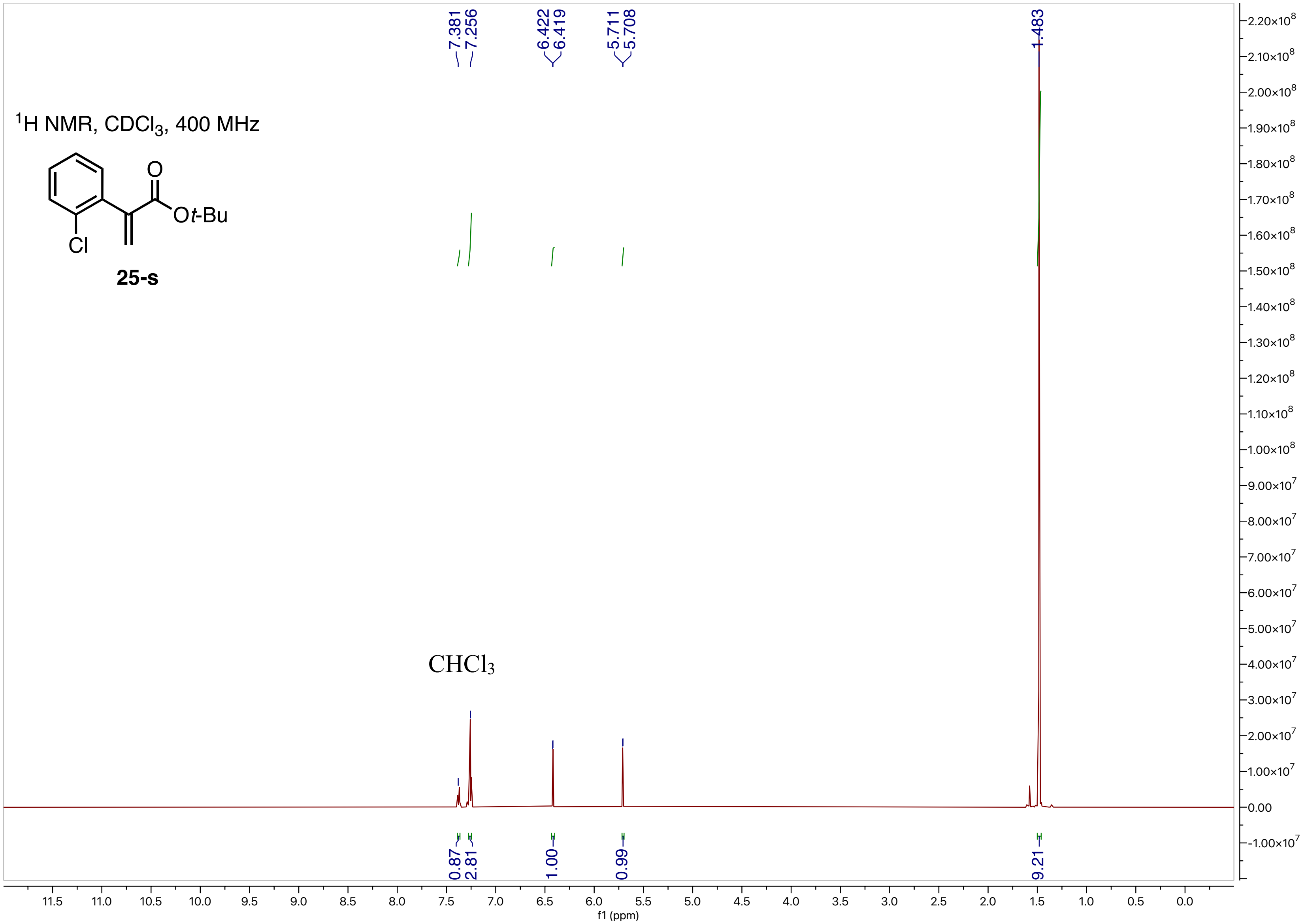

1H NMR, CDCl3, 400 MHz
O
Ot-Bu
Cl
25-s
CHCl3
7.381
7.256
6.422
6.419
5.711
5.708
1.483
0.87
2.81
1.00
0.99
9.21
f1 (ppm)

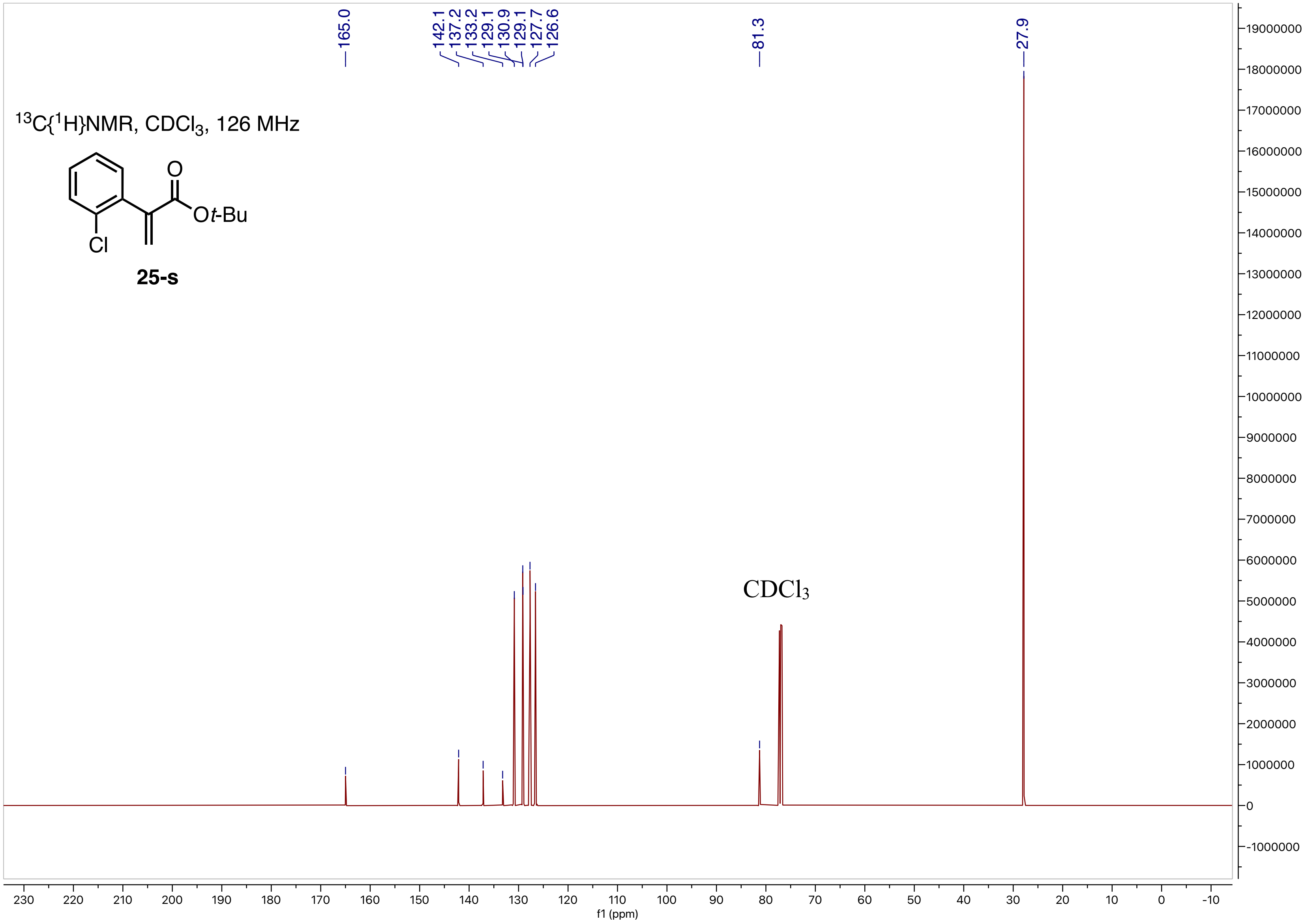

165.0
142.1
137.2
133.2
129.1
130.9
129.1
127.7
126.6
81.3
27.9
$^{13}C\{^{1}H\}$NMR, $CDCl_3$, 126 MHz
O
Ot-Bu
Cl
25-s
$CDCl_3$
f1 (ppm)

^1H NMR, $CDCl_3$, 500 MHz

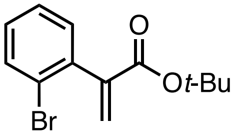

26-s

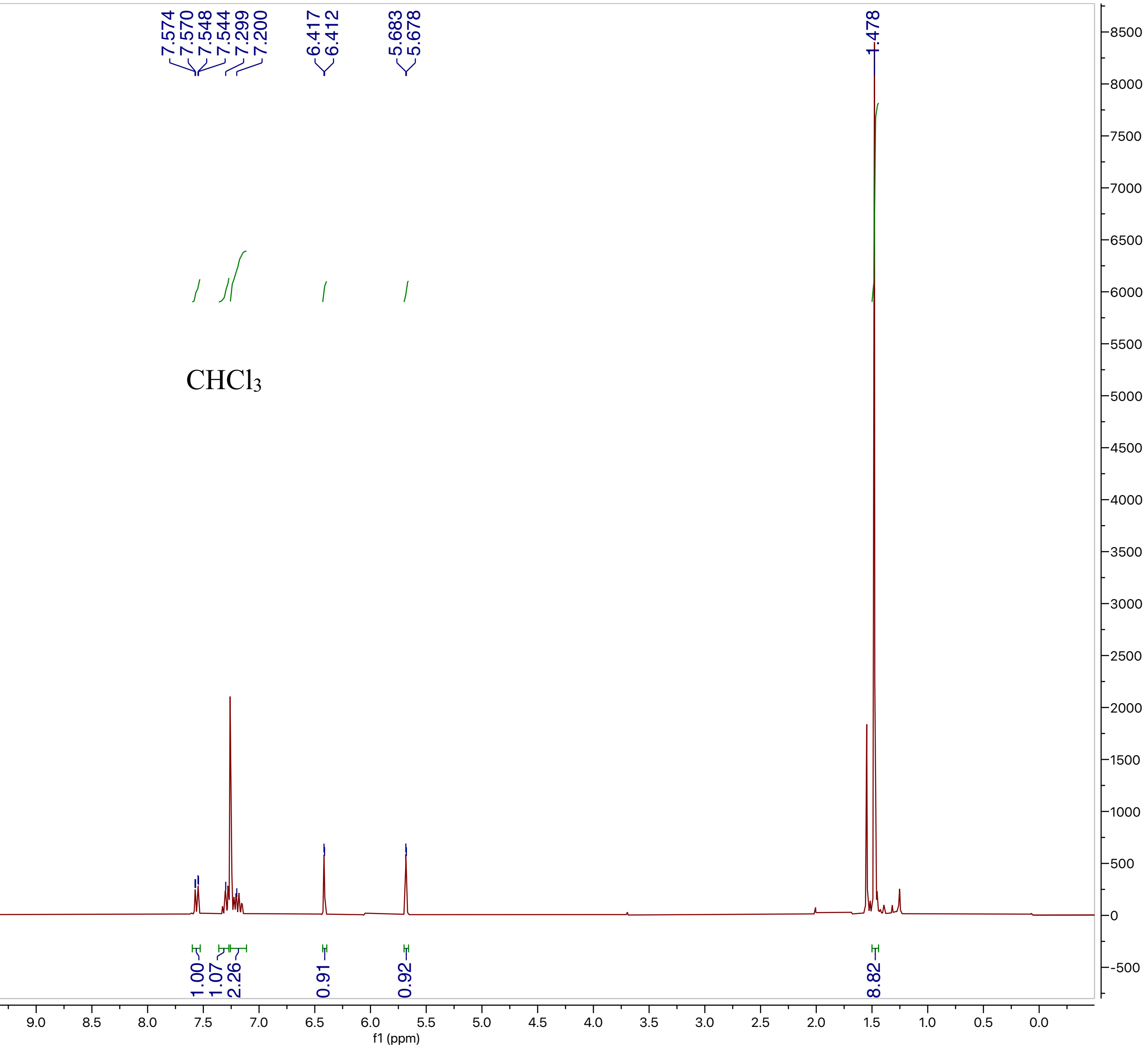

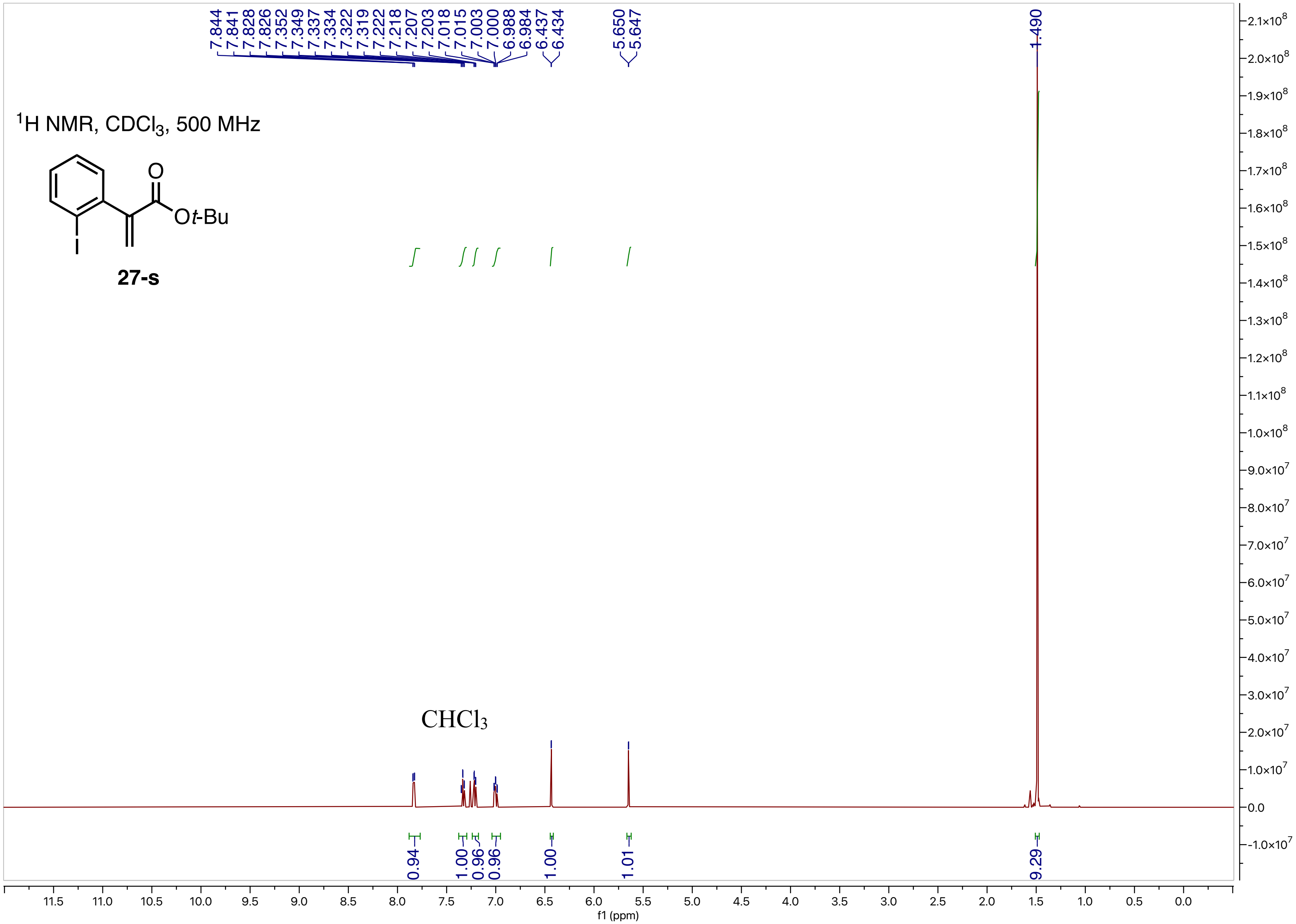

^{1}H NMR, CDCl$_3$, 500 MHz
27-s
CHCl$_3$

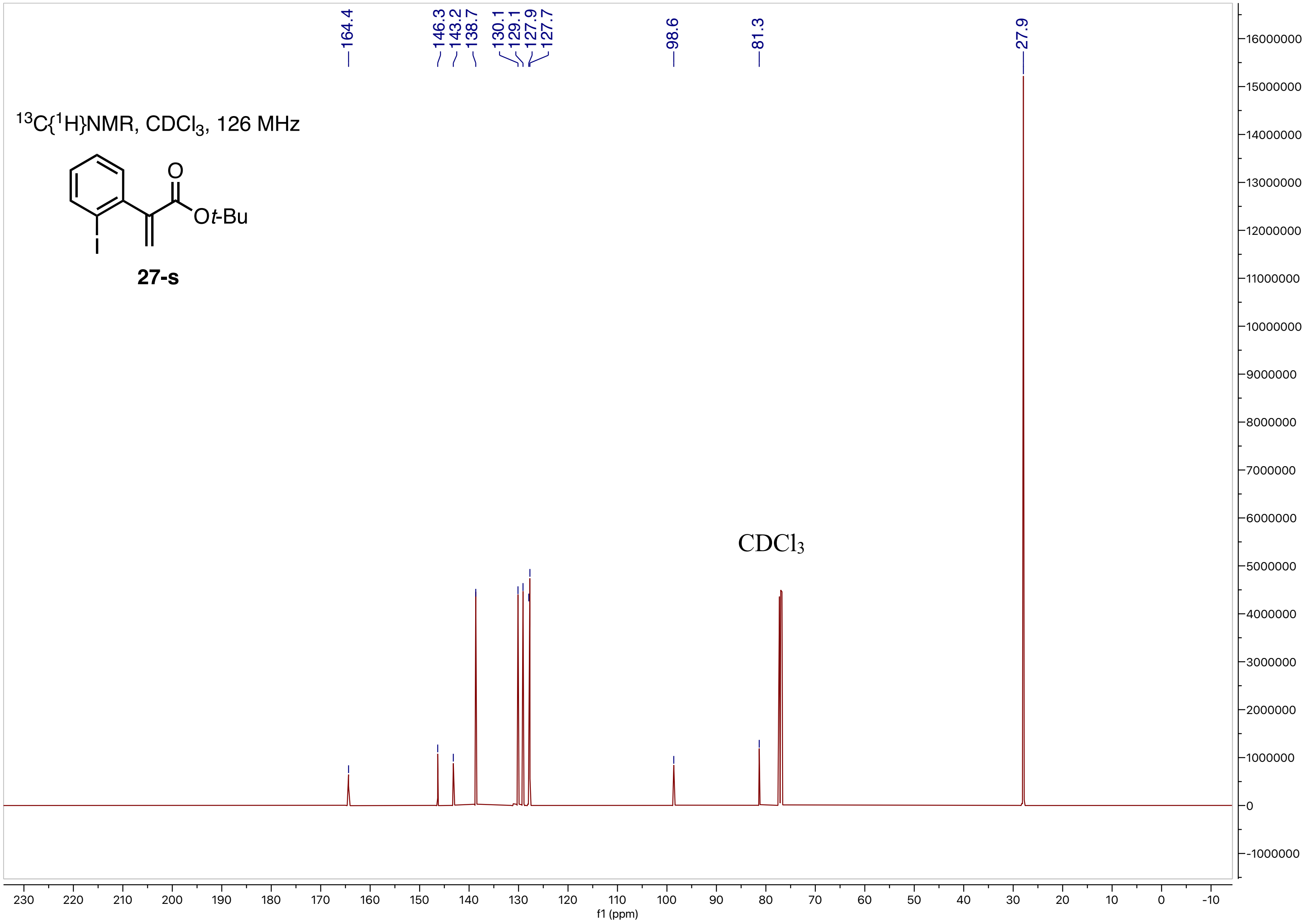

164.4
146.3
143.2
138.7
130.1
129.1
127.9
127.7
98.6
81.3
27.9
$^{13}C\{^1H\}$NMR, $CDCl_3$, 126 MHz
O
O*t*-Bu
I
27-s
$CDCl_3$
f1 (ppm)

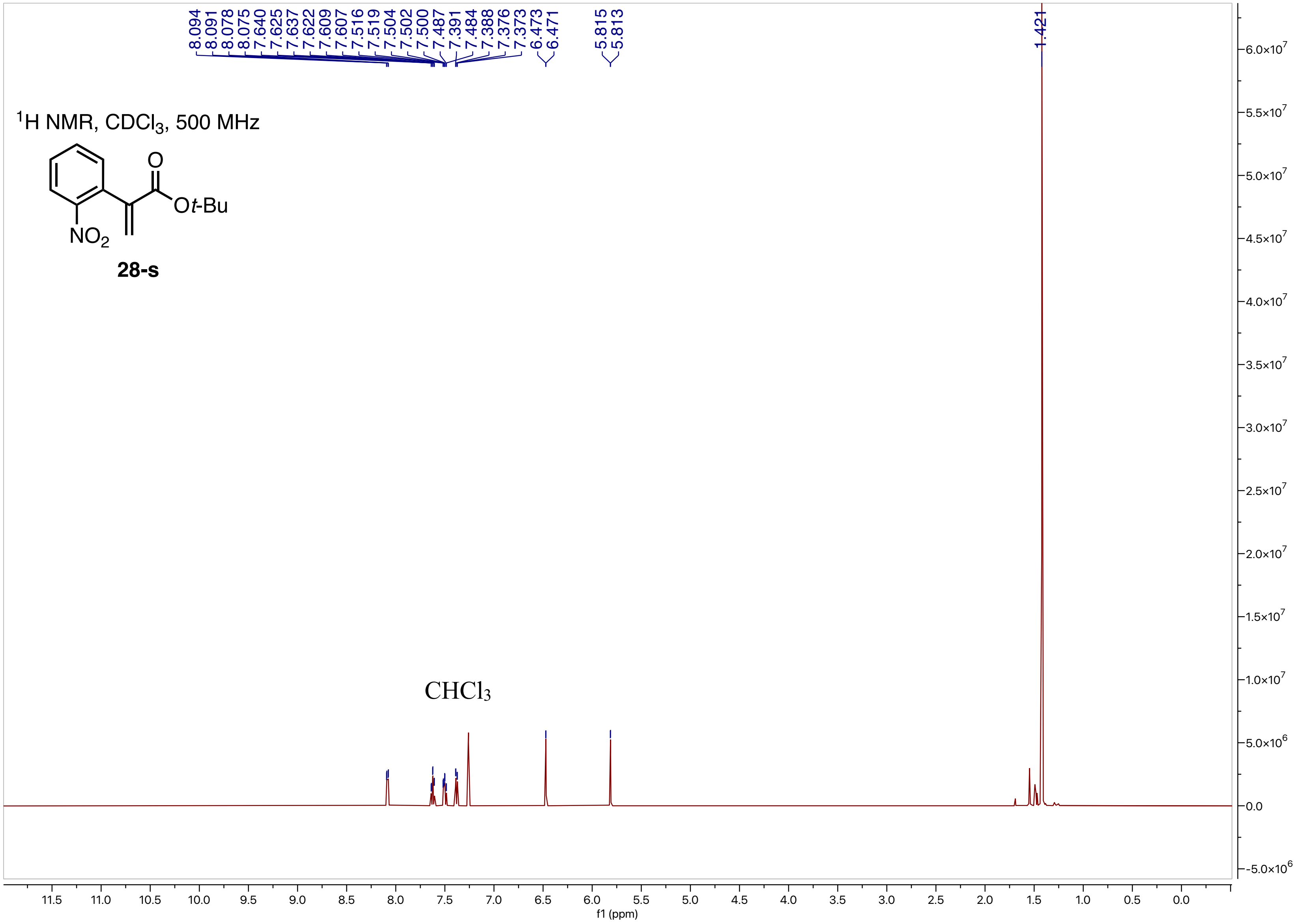

8.094
8.091
8.078
8.075
7.640
7.625
7.637
7.622
7.609
7.607
7.516
7.519
7.504
7.502
7.500
7.487
7.391
7.484
7.388
7.376
7.373
6.473
6.471
5.815
5.813
1.421
^{1}H NMR, $CDCl_3$, 500 MHz
O
O*t*-Bu
NO_2
28-s
$CHCl_3$
f1 (ppm)

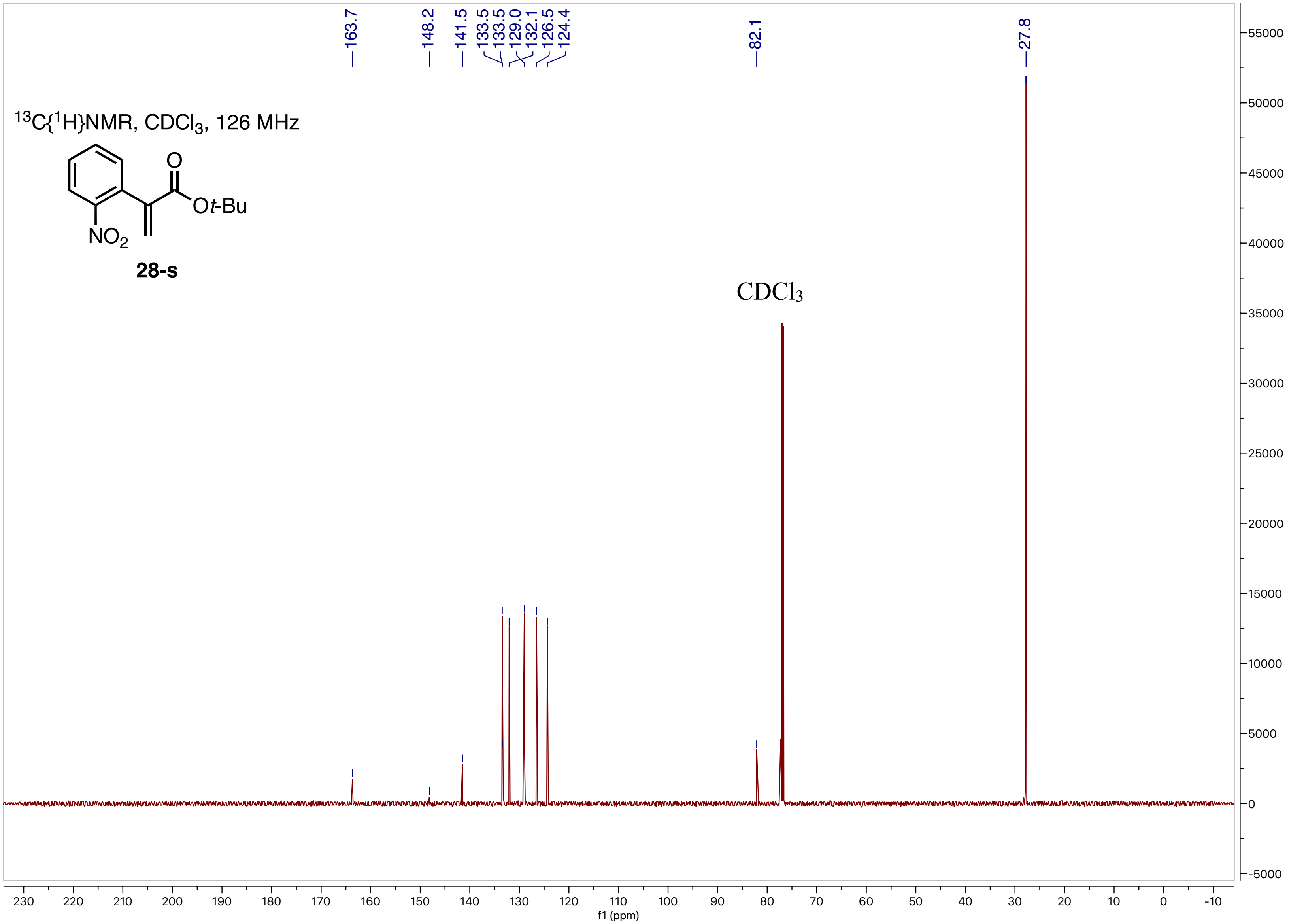

163.7
148.2
141.5
133.5
133.5
129.0
132.1
126.5
124.4
82.1
27.8
13C{1H}NMR, CDCl3, 126 MHz
O
Ot-Bu
NO2
28-s
CDCl3
f1 (ppm)

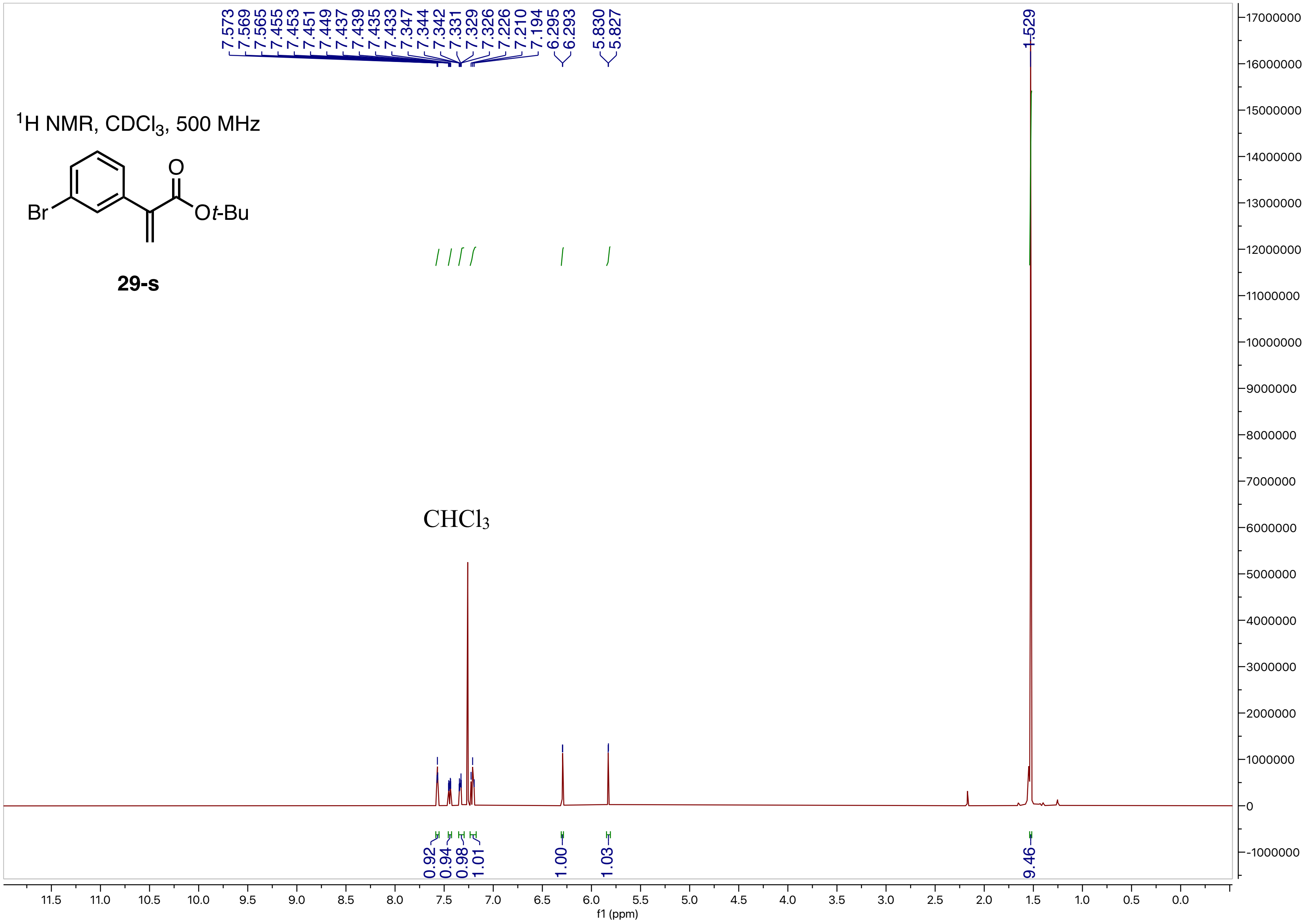

7.573
7.569
7.565
7.455
7.453
7.451
7.449
7.439
7.437
7.435
7.433
7.347
7.344
7.342
7.331
7.329
7.326
7.226
7.210
7.194
6.295
6.293
5.830
5.827
1.529
^1H NMR, $CDCl_3$, 500 MHz
O
Br
O*t*-Bu
29-s
$CHCl_3$
0.92
0.94
0.98
1.01
1.00
1.03
9.46
f1 (ppm)

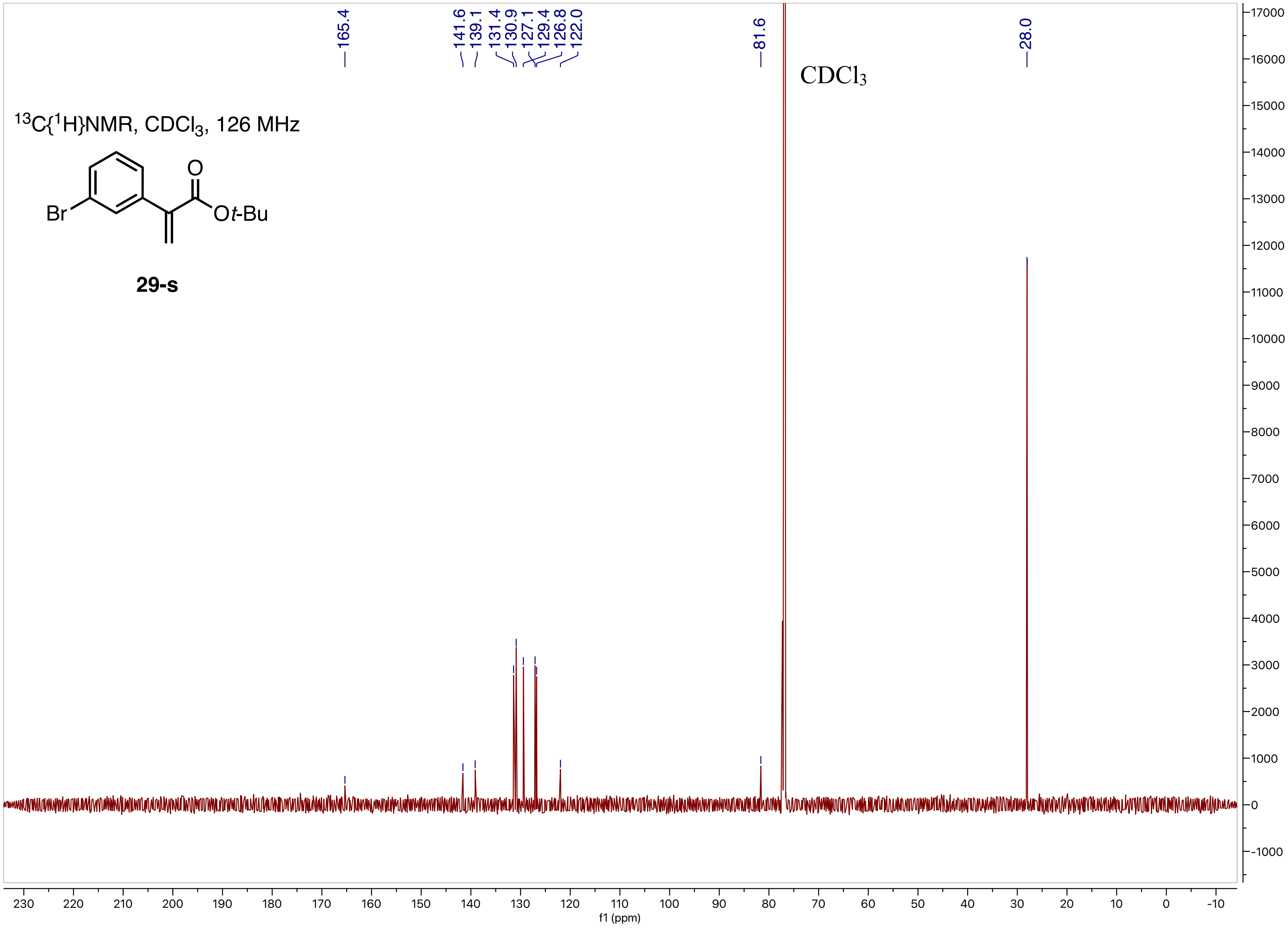

13C{1H}NMR, CDCl3, 126 MHz
29-s
Br
O
Ot-Bu
165.4
141.6
139.1
131.4
130.9
127.1
129.4
126.8
122.0
81.6
28.0
CDCl3
f1 (ppm)

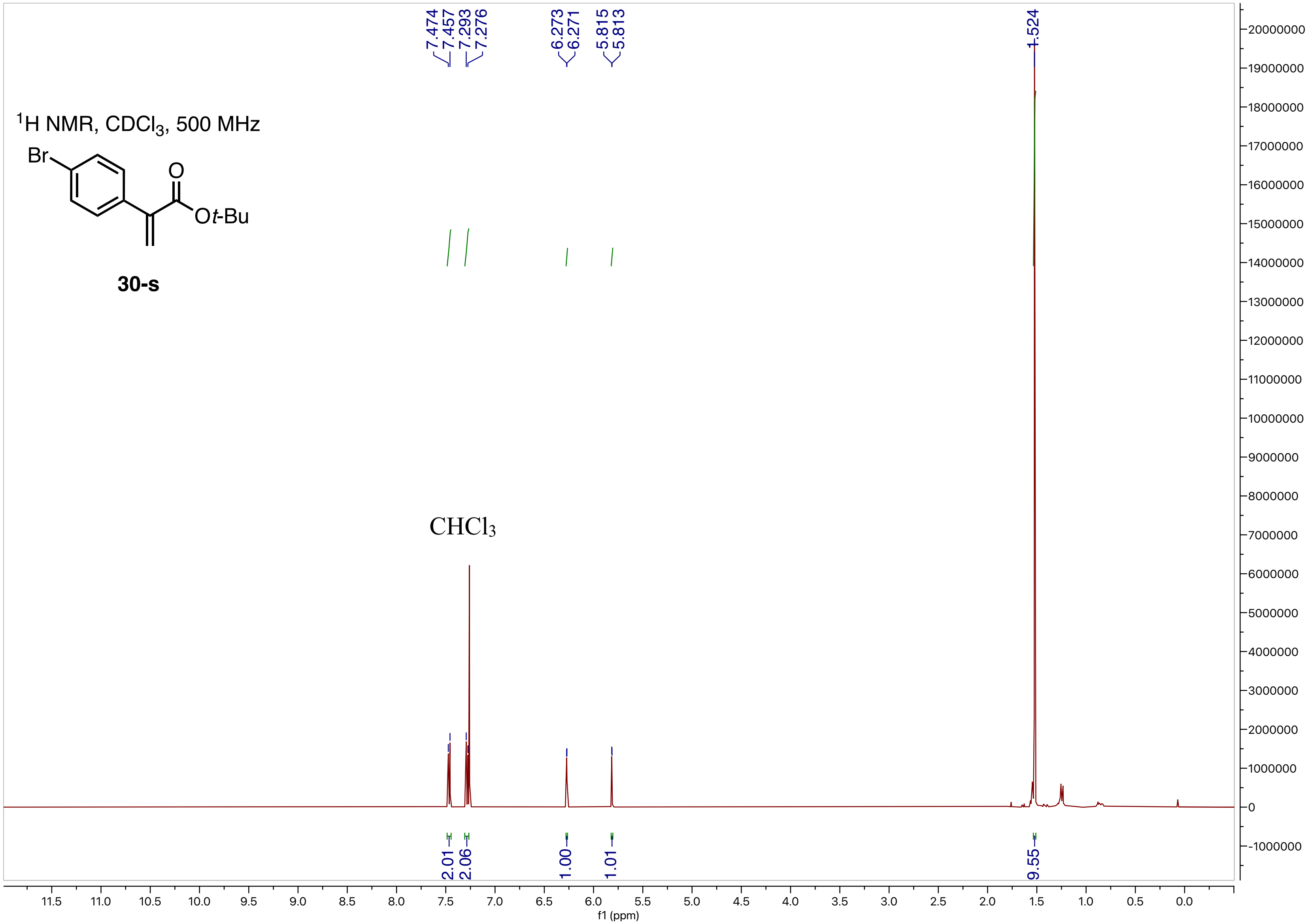

7.474
7.457
7.293
7.276
6.273
6.271
5.815
5.813
1.524
¹H NMR, CDCl3, 500 MHz
Br
O
Ot-Bu
30-s
CHCl3
2.01
2.06
1.00
1.01
9.55
f1 (ppm)

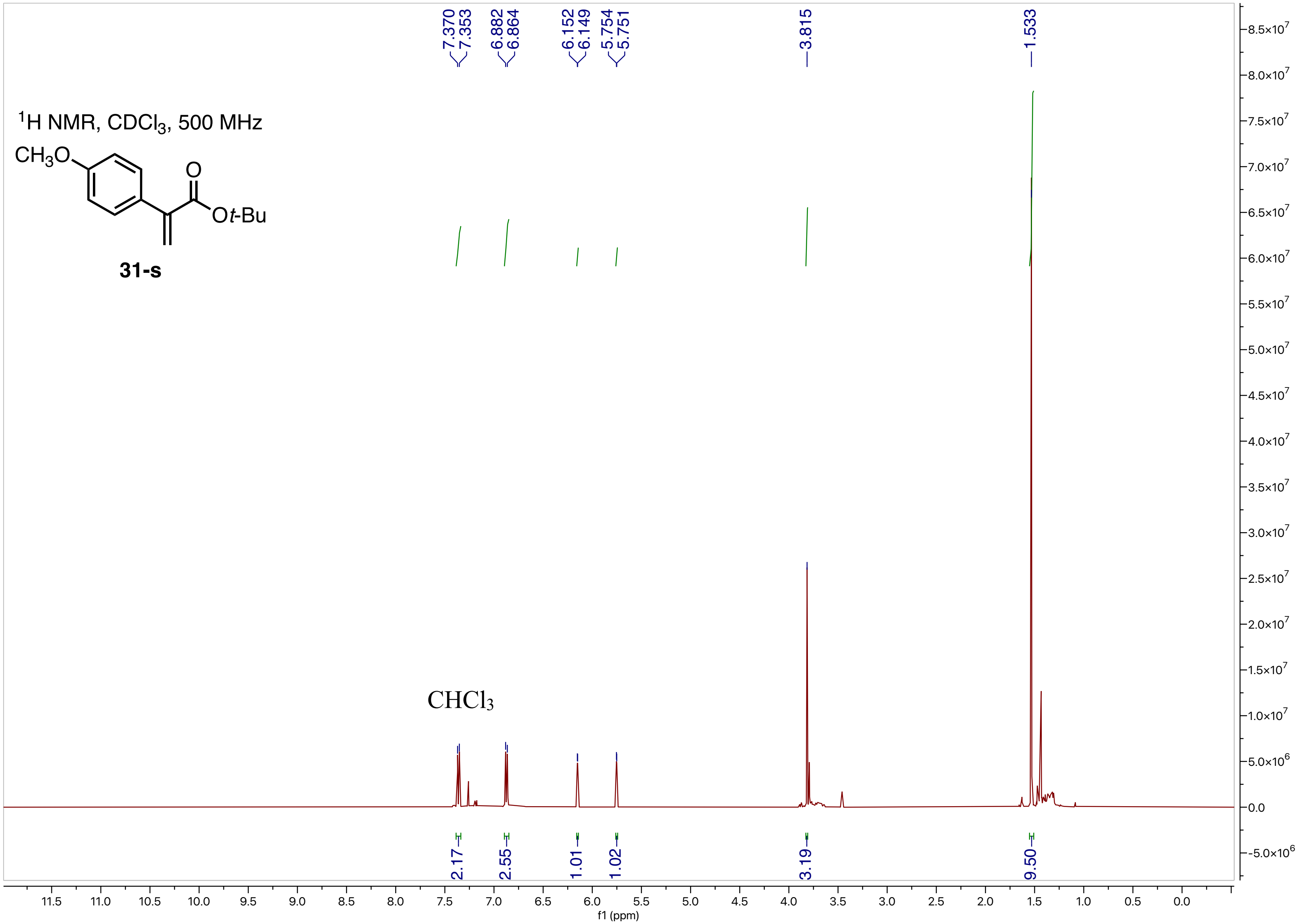

1H NMR, CDCl3, 500 MHz
CH3O
O
Ot-Bu
31-s
CHCl3
7.370
7.353
6.882
6.864
6.152
6.149
5.754
5.751
3.815
1.533
2.17
2.55
1.01
1.02
3.19
9.50
f1 (ppm)

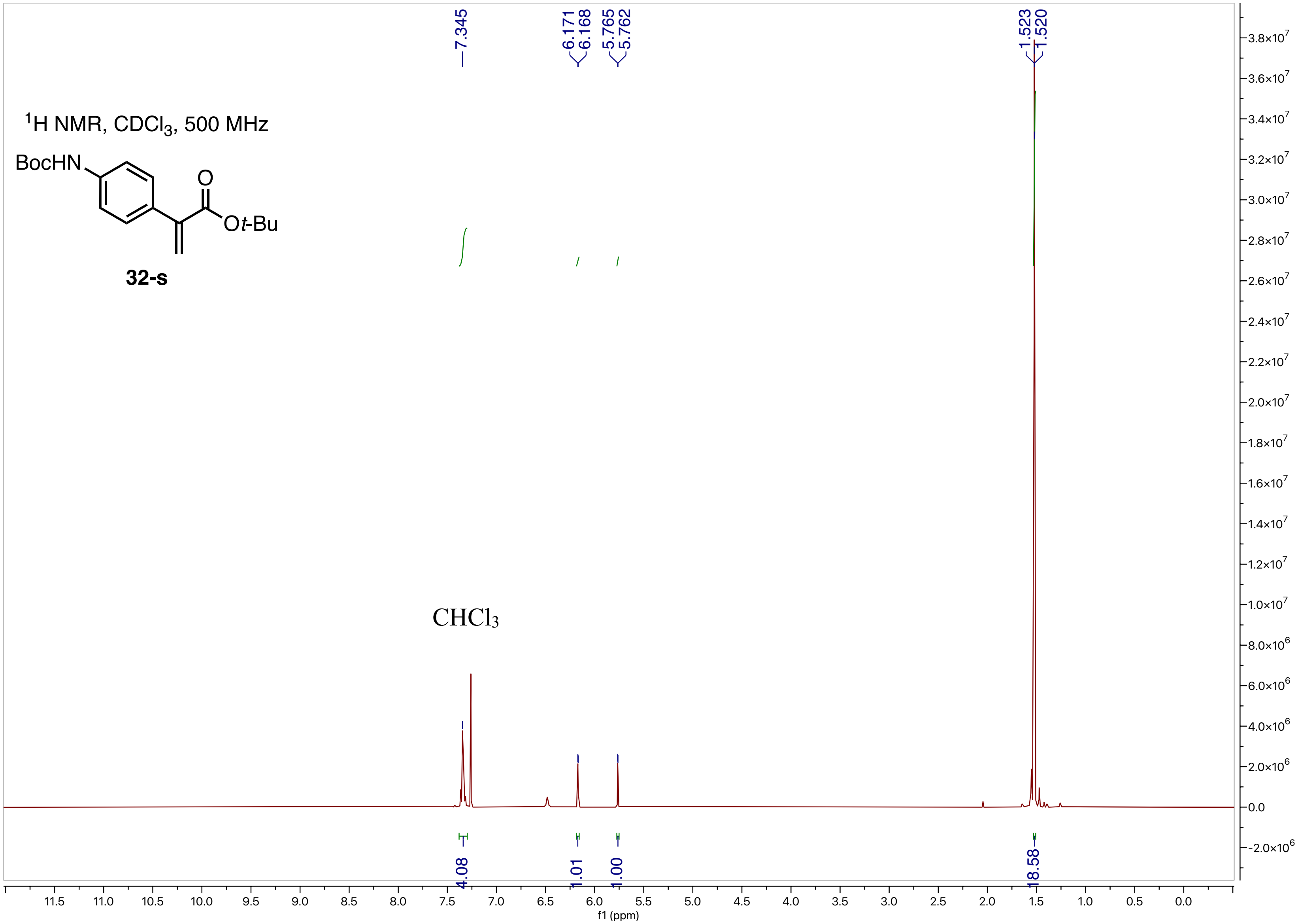

7.345
6.171
6.168
5.765
5.762
1.523
1.520
1H NMR, CDCl3, 500 MHz
BocHN
O
Ot-Bu
32-s
CHCl3
4.08
1.01
1.00
18.58
f1 (ppm)

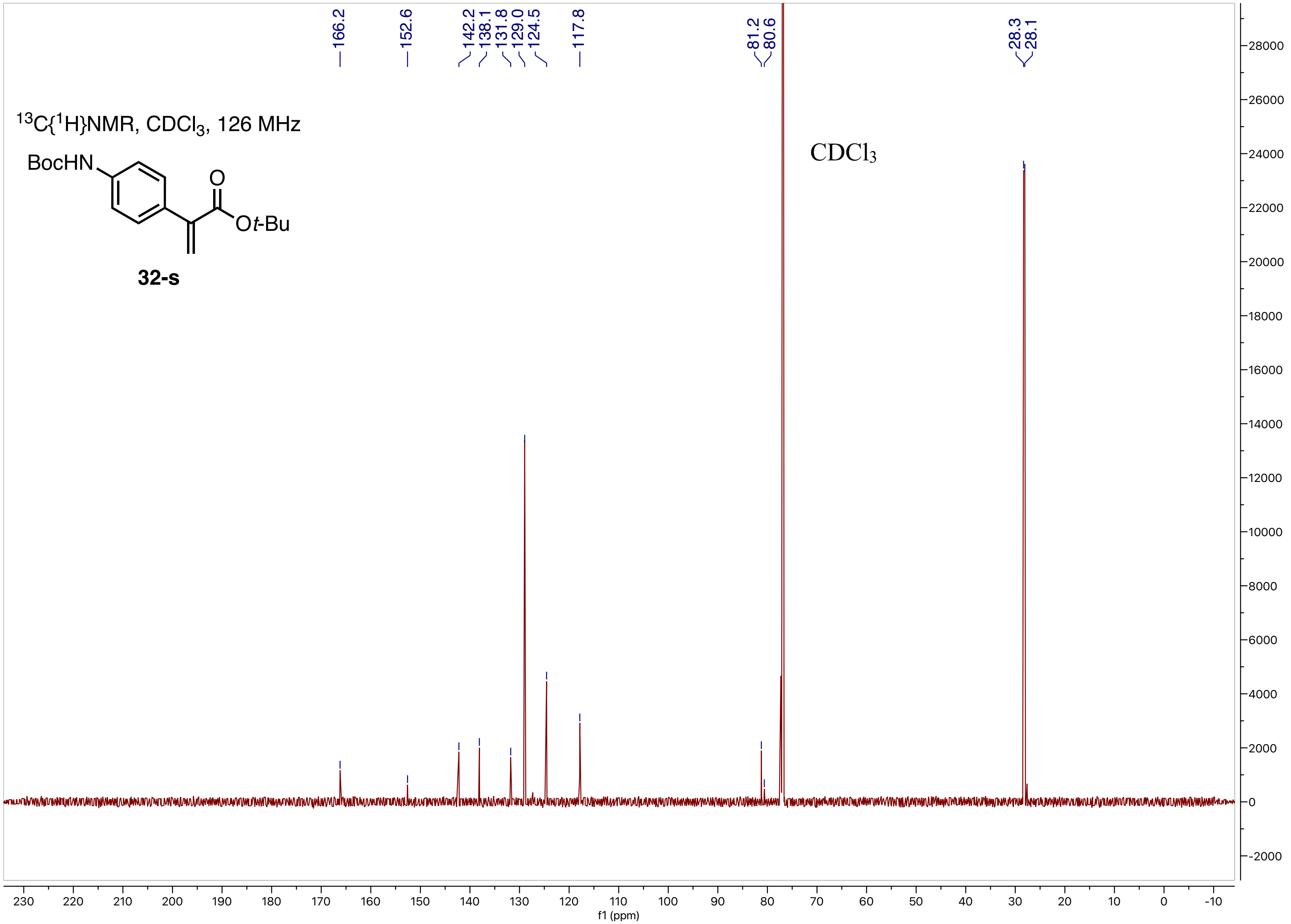

$^{13}C\{^1H\}$NMR, $CDCl_3$, 126 MHz
BocHN
O
Ot-Bu
32-s
166.2
152.6
142.2
138.1
131.8
129.0
124.5
117.8
81.2
80.6
28.3
28.1
$CDCl_3$
f1 (ppm)

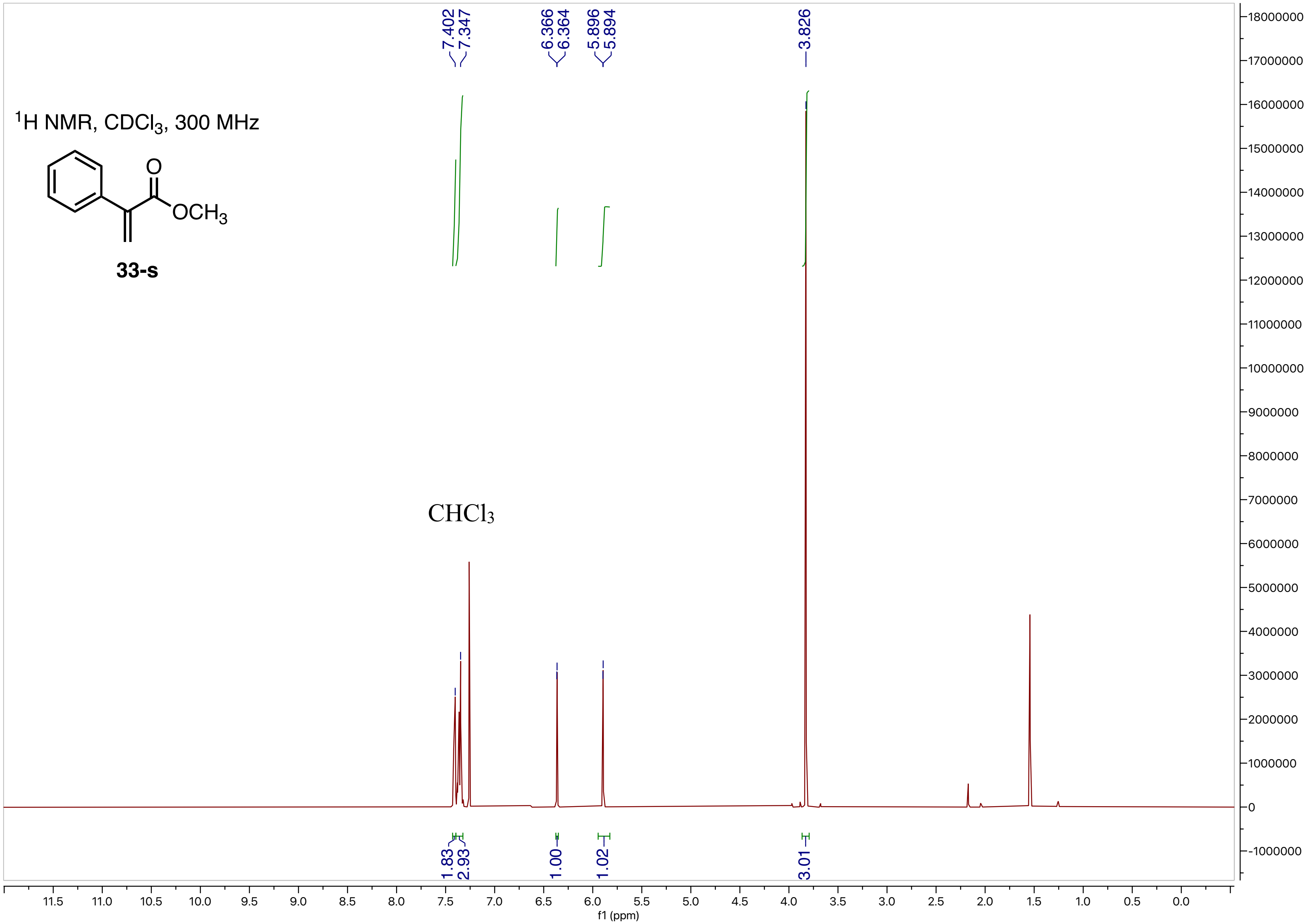

^{1}H NMR, $CDCl_3$, 300 MHz
O
OCH_3
33-s
7.402
7.347
6.366
6.364
5.896
5.894
3.826
CHCl3
1.83
2.93
1.00
1.02
3.01
f1 (ppm)

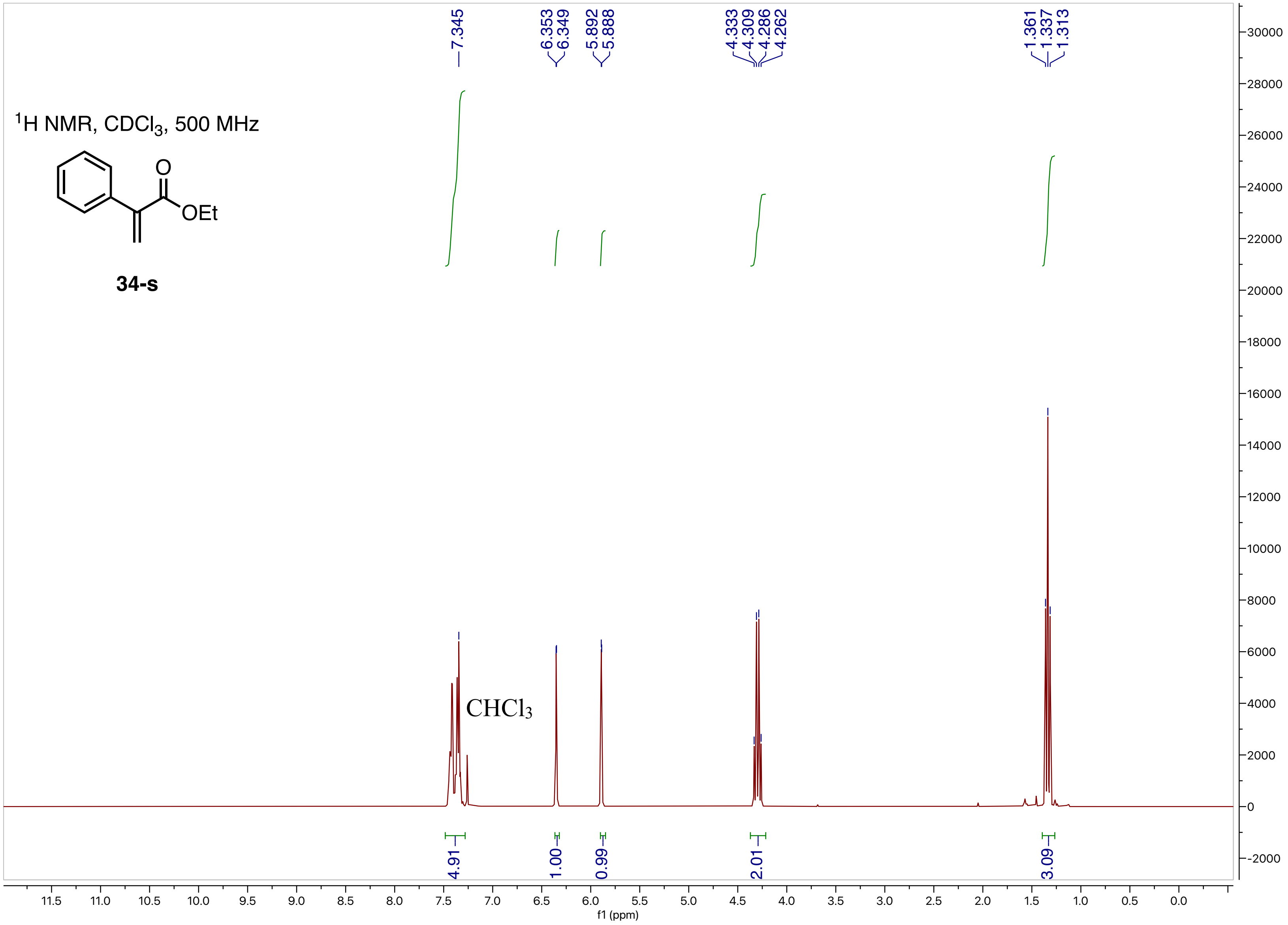

^{1}H NMR, $CDCl_3$, 500 MHz
34-s
OEt
O
CHCl3
7.345
6.353
6.349
5.892
5.888
4.333
4.309
4.286
4.262
1.361
1.337
1.313
4.91
1.00
0.99
2.01
3.09
f1 (ppm)

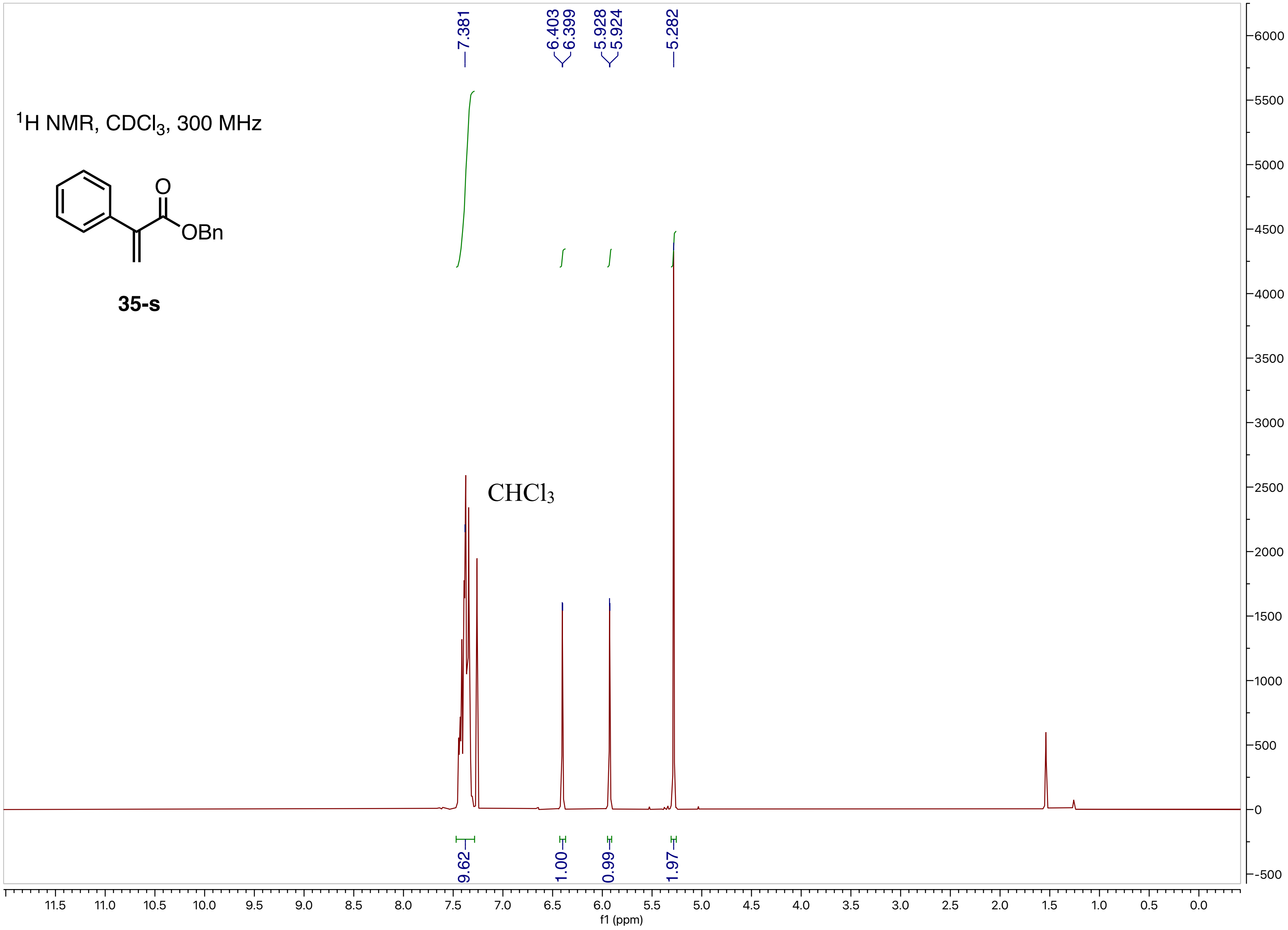

7.381
6.403
6.399
5.928
5.924
5.282
1H NMR, CDCl3, 300 MHz
O
OBn
35-s
CHCl3
9.62
1.00
0.99
1.97
f1 (ppm)

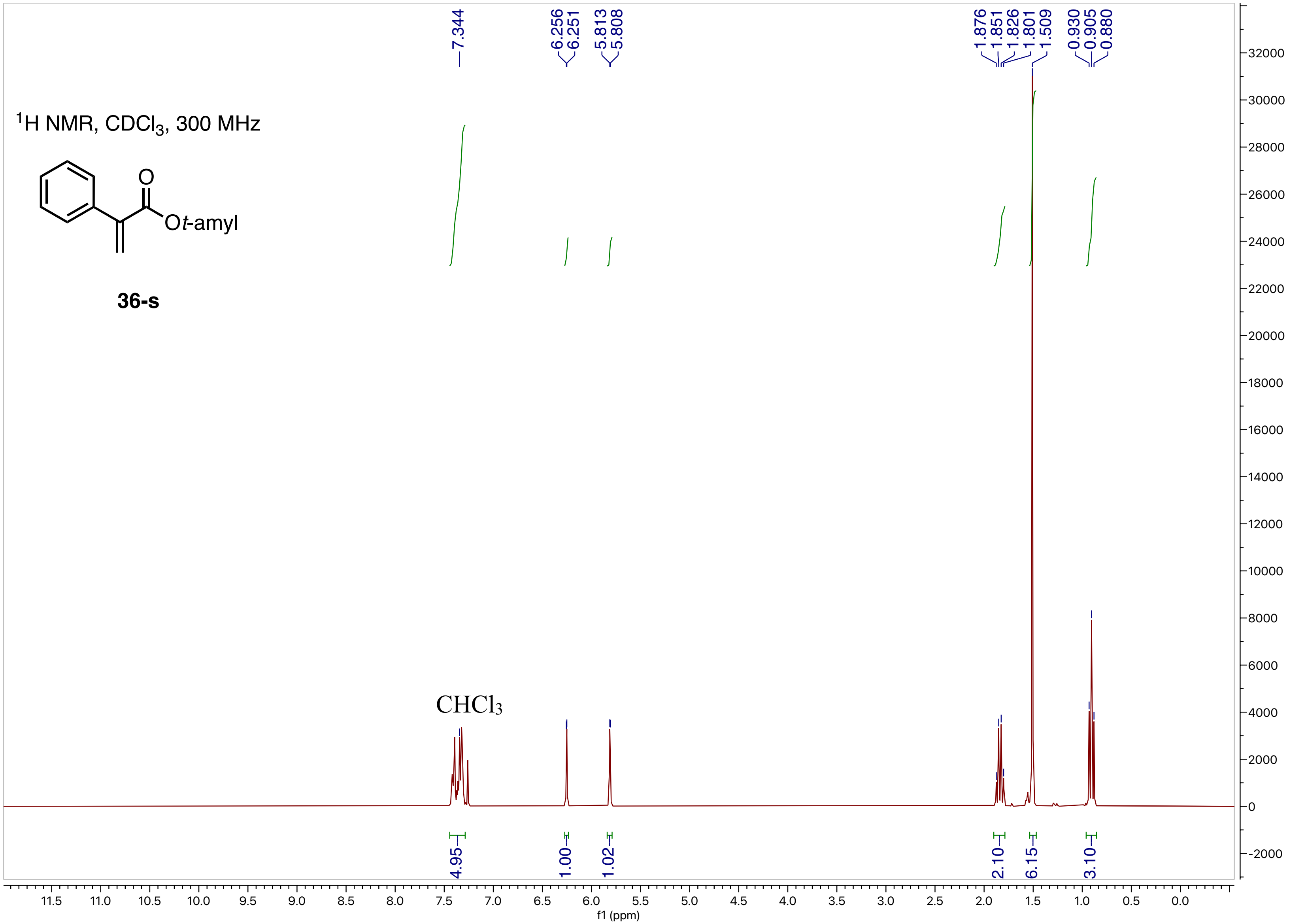

^{1}H NMR, $CDCl_3$, 300 MHz
O
Ot-amyl
36-s
CHCl3
7.344
6.256
6.251
5.813
5.808
1.876
1.851
1.826
1.801
1.509
0.930
0.905
0.880
4.95
1.00
1.02
2.10
6.15
3.10
f1 (ppm)

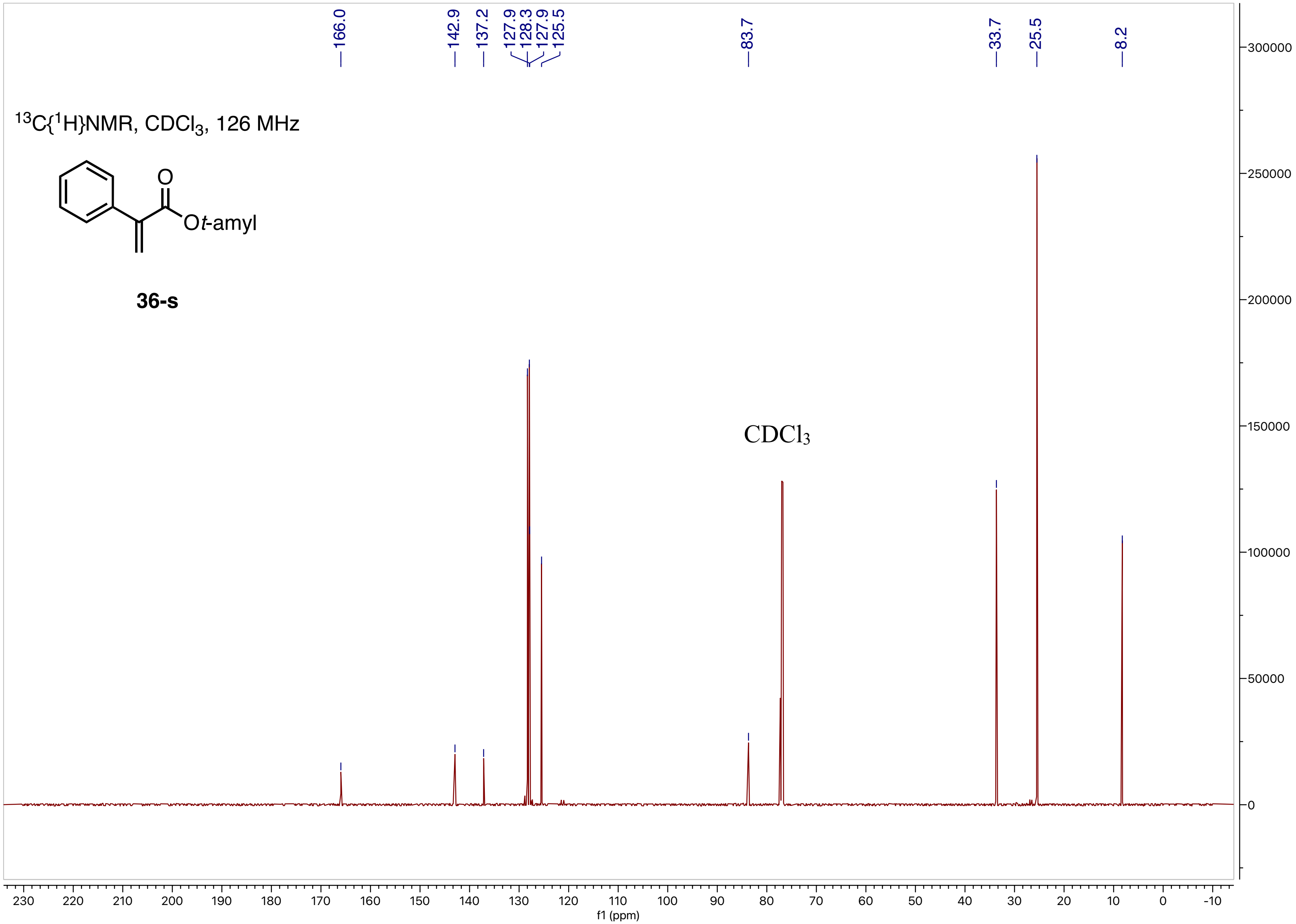
166.0
142.9
137.2
127.9
128.3
127.9
125.5
83.7
33.7
25.5
8.2
$^{13}C\{^1H\}$NMR, $CDCl_3$, 126 MHz
O
Ot-amyl
36-s
$CDCl_3$
f1 (ppm)

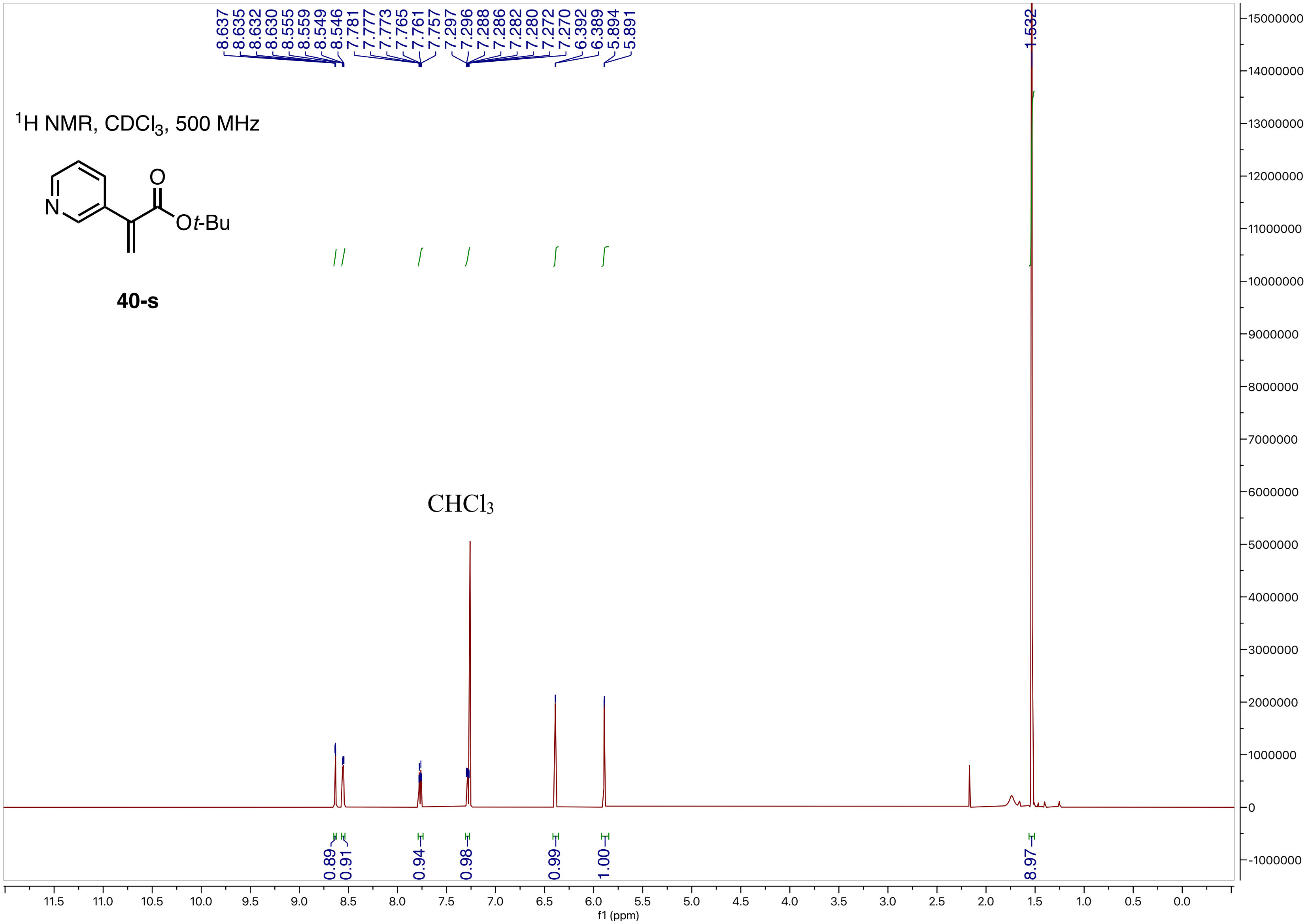

8.637
8.635
8.632
8.630
8.555
8.559
8.549
8.546
7.781
7.777
7.773
7.765
7.761
7.757
7.297
7.296
7.288
7.286
7.282
7.280
7.272
7.270
6.392
6.389
5.894
5.891
1.532
^{1}H NMR, $CDCl_3$, 500 MHz
O
N
Ot-Bu
40-s
$CHCl_3$
0.89
0.91
0.94
0.98
0.99
1.00
8.97
f1 (ppm)

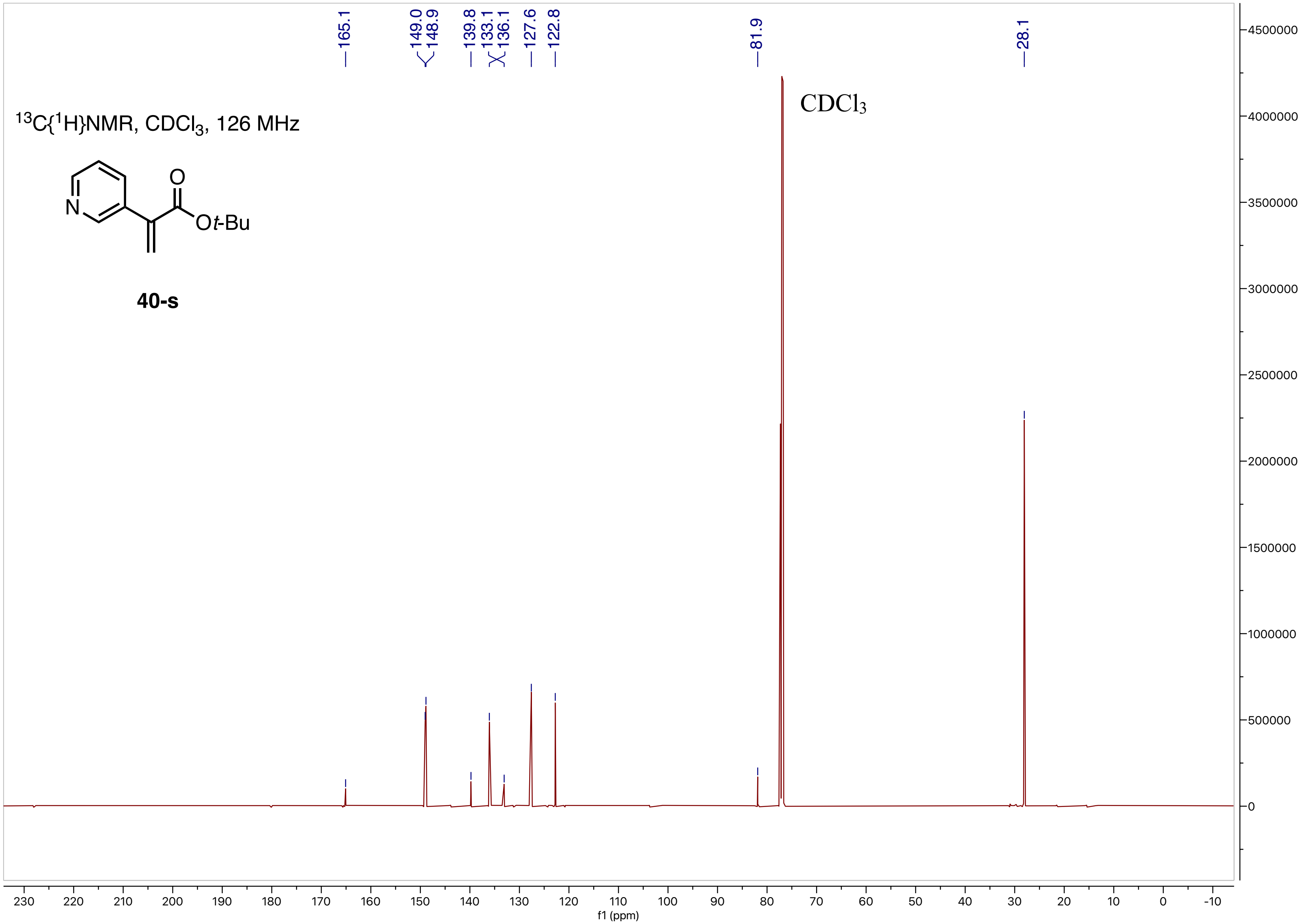
165.1
149.0
148.9
139.8
133.1
136.1
127.6
122.8
81.9
28.1
CDCl3
13C{1H}NMR, CDCl3, 126 MHz
O
N
Ot-Bu
40-s
4500000
4000000
3500000
3000000
2500000
2000000
1500000
1000000
500000
0
230 220 210 200 190 180 170 160 150 140 130 120 110 100 90 80 70 60 50 40 30 20 10 0 -10
f1 (ppm)

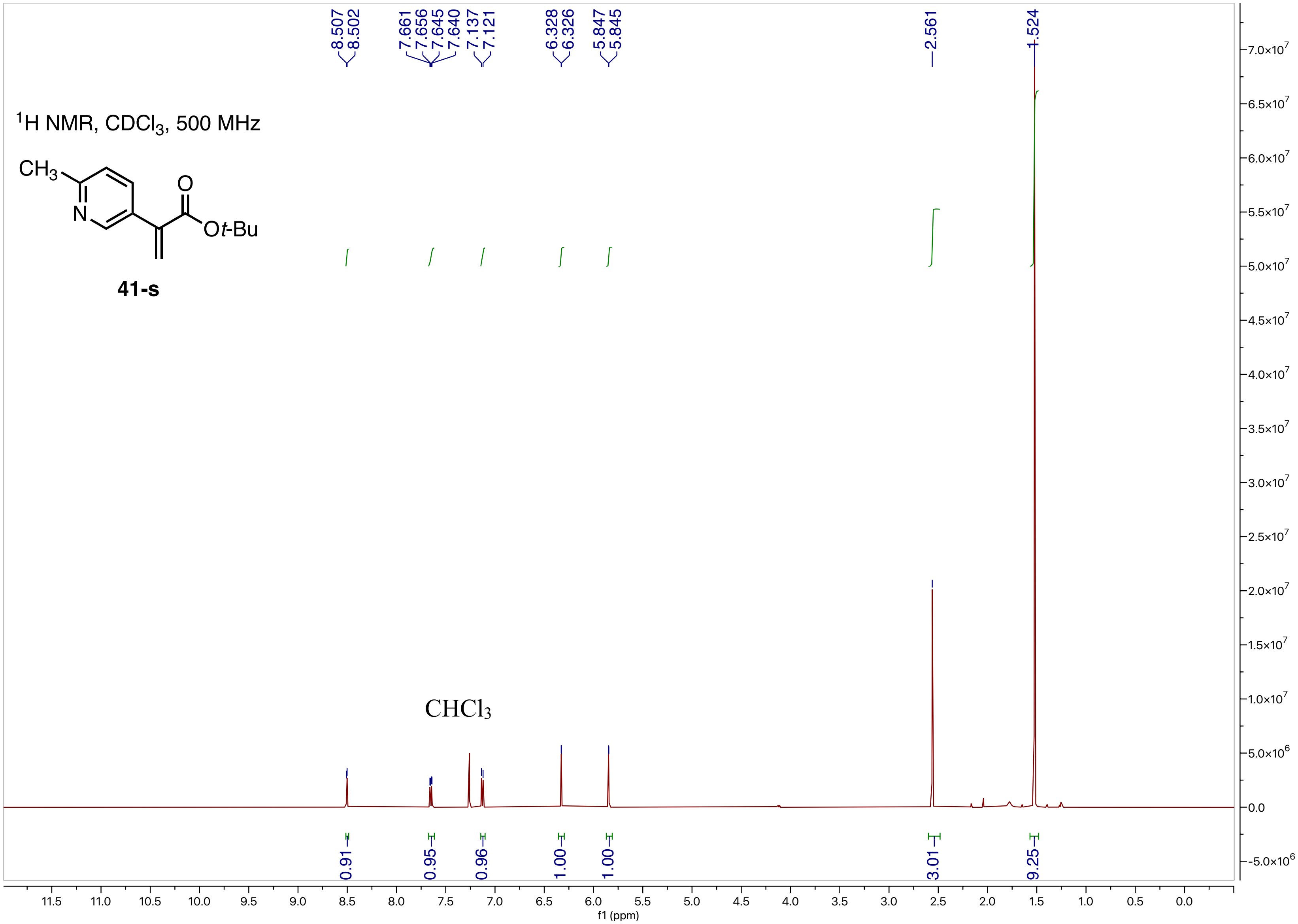

8.507
8.502
7.661
7.656
7.645
7.640
7.137
7.121
6.328
6.326
5.847
5.845
2.561
1.524
1H NMR, CDCl3, 500 MHz
CH3
N
O
Ot-Bu
41-s
CHCl3
0.91
0.95
0.96
1.00
1.00
3.01
9.25
f1 (ppm)

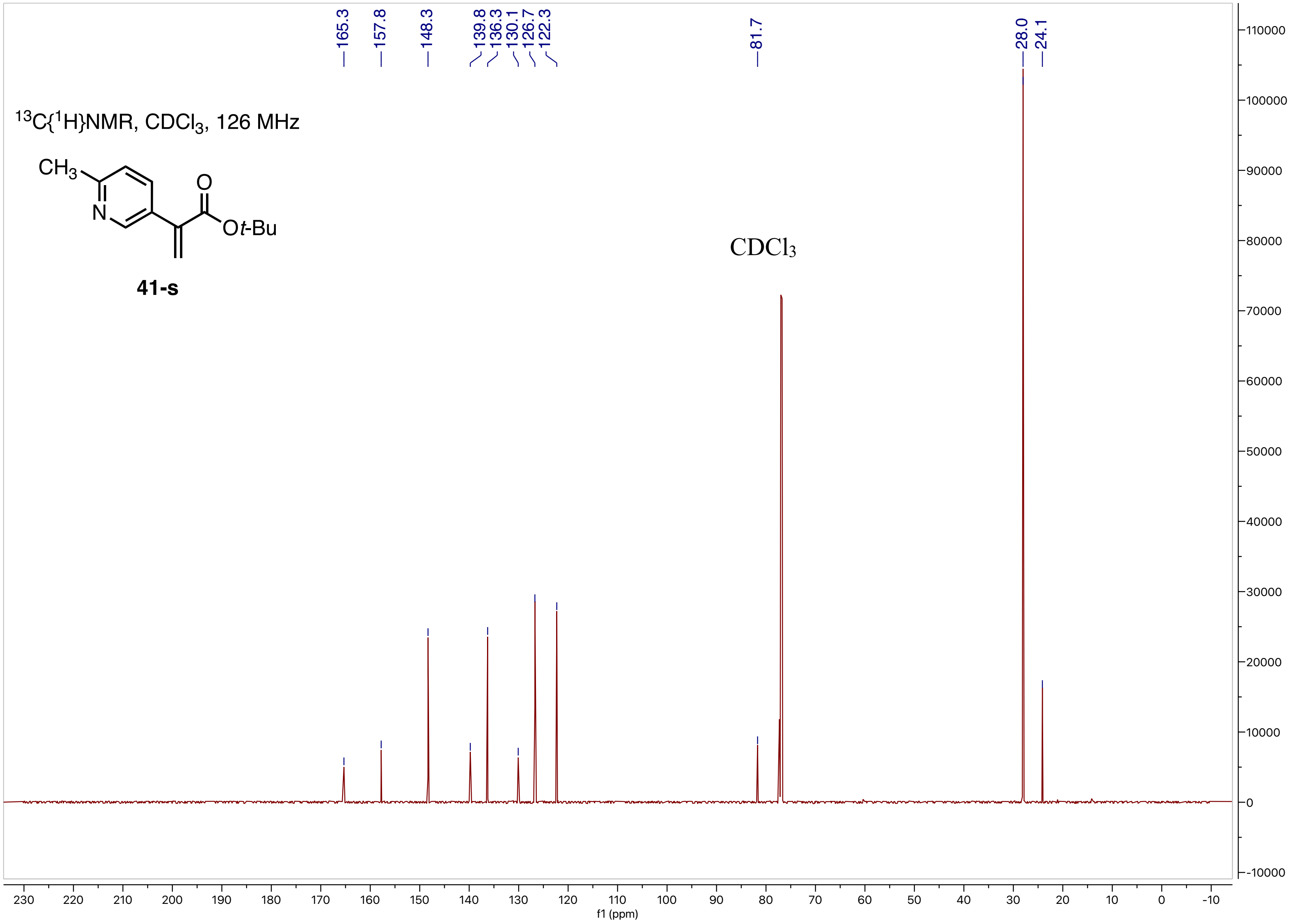

165.3
157.8
148.3
139.8
136.3
130.1
126.7
122.3
81.7
28.0
24.1
$^{13}C\{^1H\}$NMR, $CDCl_3$, 126 MHz
CH_3
N
O
O*t*-Bu
41-s
$CDCl_3$
f1 (ppm)

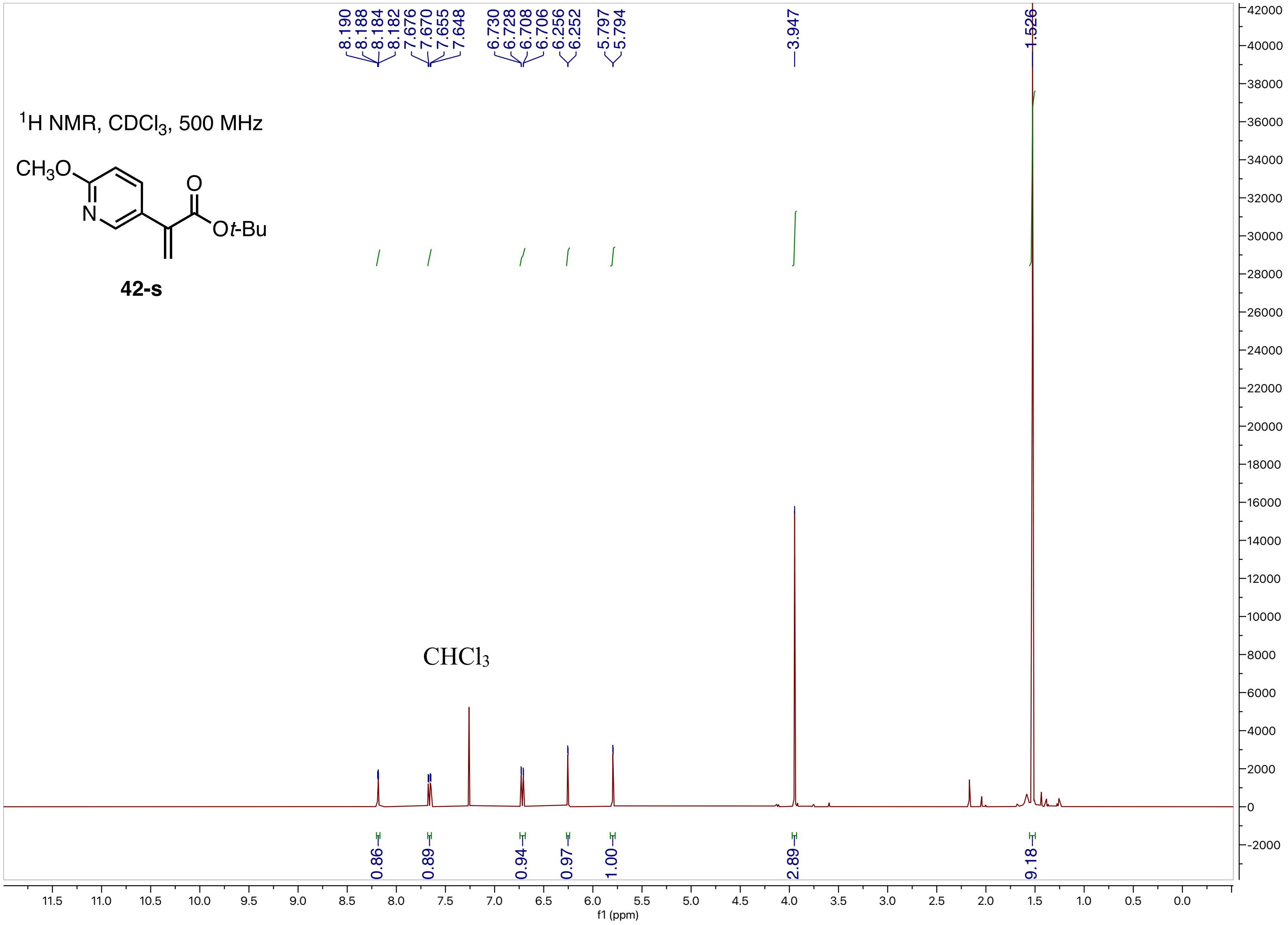

1H NMR, CDCl3, 500 MHz
CH3O
N
O
Ot-Bu
42-s
CHCl3
8.190
8.188
8.184
8.182
7.676
7.670
7.655
7.648
6.730
6.728
6.708
6.706
6.256
6.252
5.797
5.794
3.947
1.526
0.86
0.89
0.94
0.97
1.00
2.89
9.18
f1 (ppm)

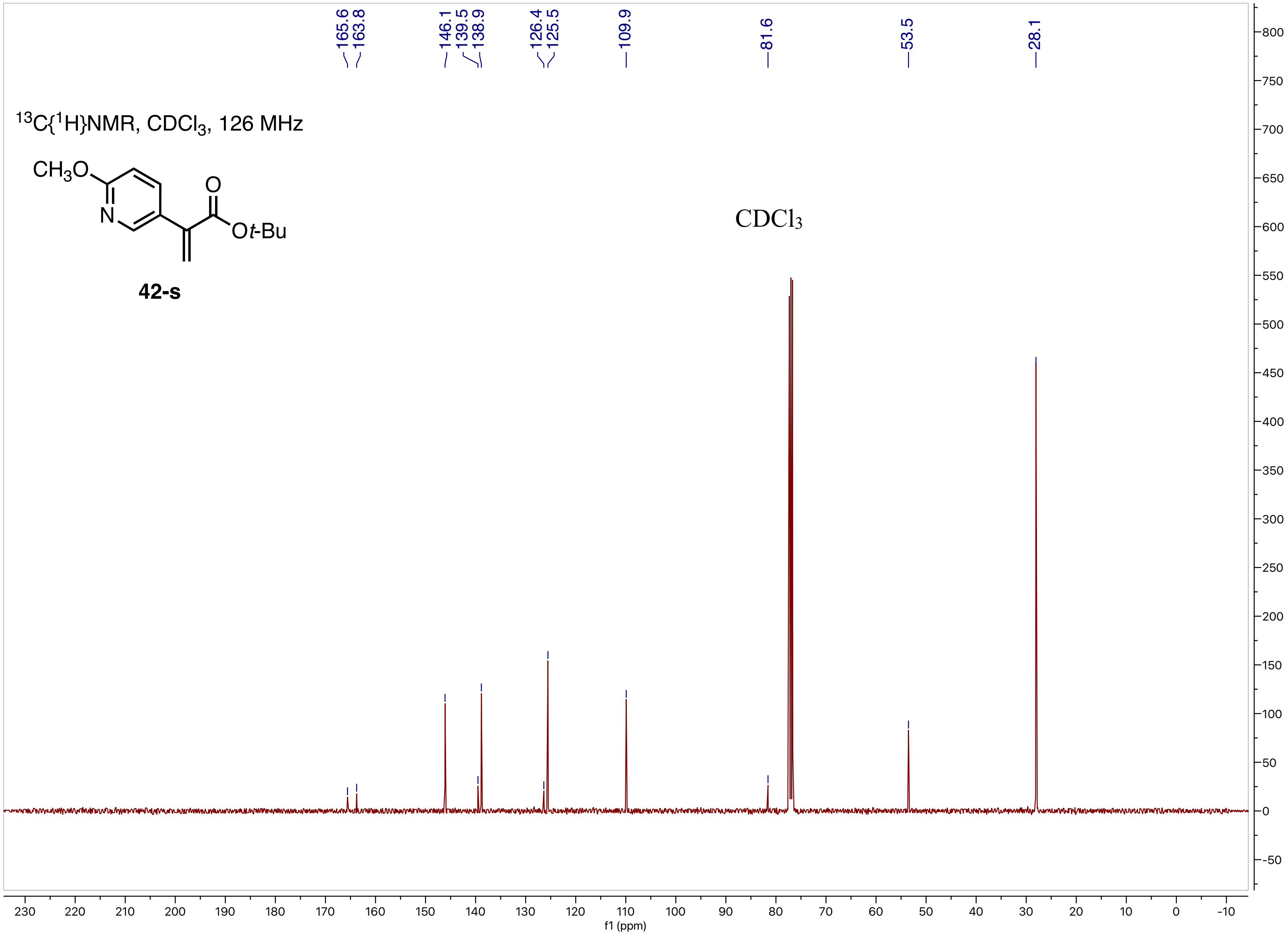
165.6
163.8
146.1
139.5
138.9
126.4
125.5
109.9
81.6
53.5
28.1
$^{13}C\{^1H\}$NMR, $CDCl_3$, 126 MHz
CH_3O
N
O
O*t*-Bu
42-s
$CDCl_3$
f1 (ppm)

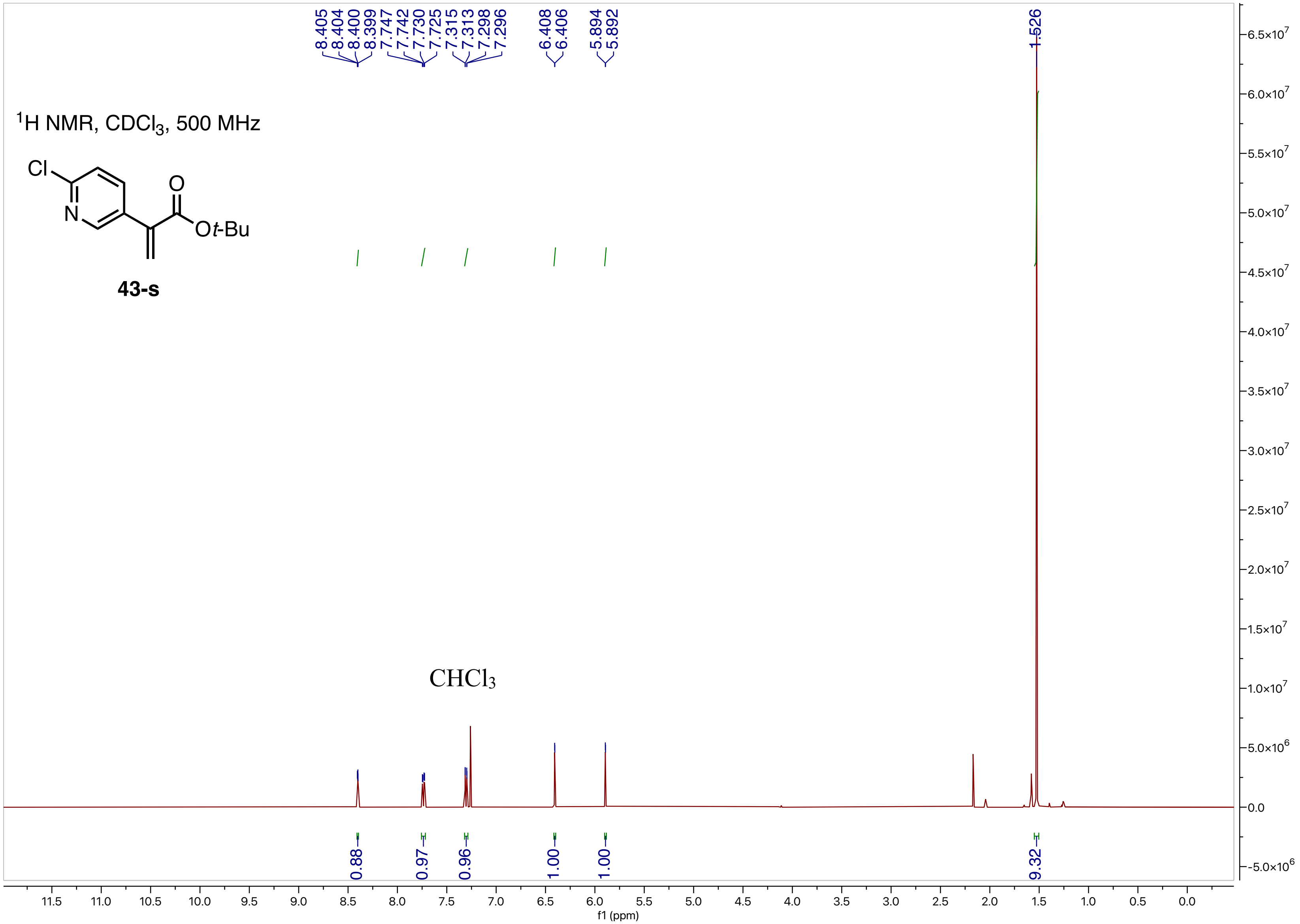

8.405
8.404
8.400
8.399
7.747
7.742
7.730
7.725
7.315
7.313
7.298
7.296
6.408
6.406
5.894
5.892
1.526
¹H NMR, CDCl₃, 500 MHz
Cl
N
O
Ot-Bu
43-s
CHCl₃
0.88
0.97
0.96
1.00
1.00
9.32
f1 (ppm)

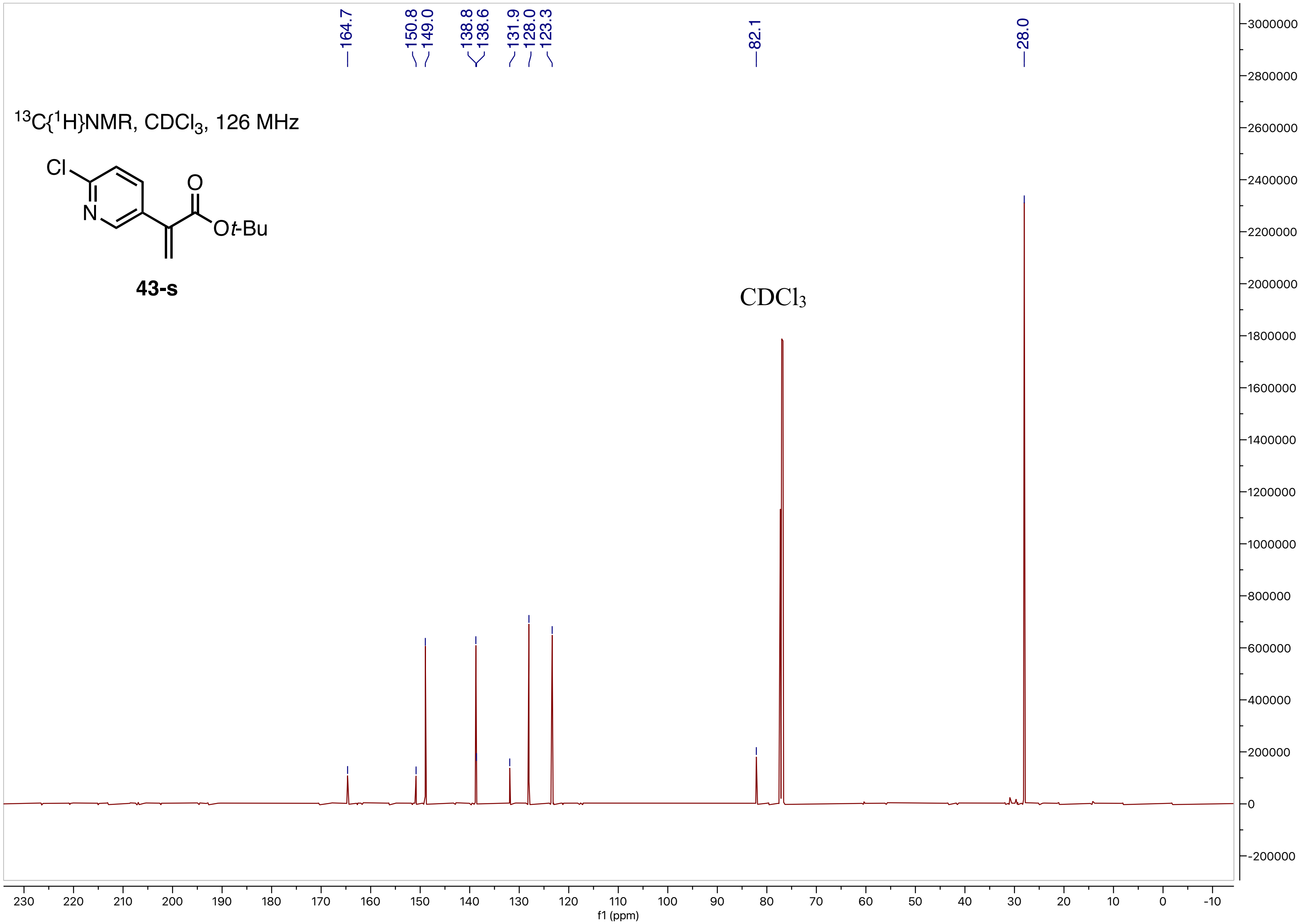

164.7
150.8
149.0
138.8
138.6
131.9
128.0
123.3
82.1
28.0
$^{13}C\{^1H\}$NMR, $CDCl_3$, 126 MHz
Cl
N
O
Ot-Bu
43-s
$CDCl_3$
f1 (ppm)

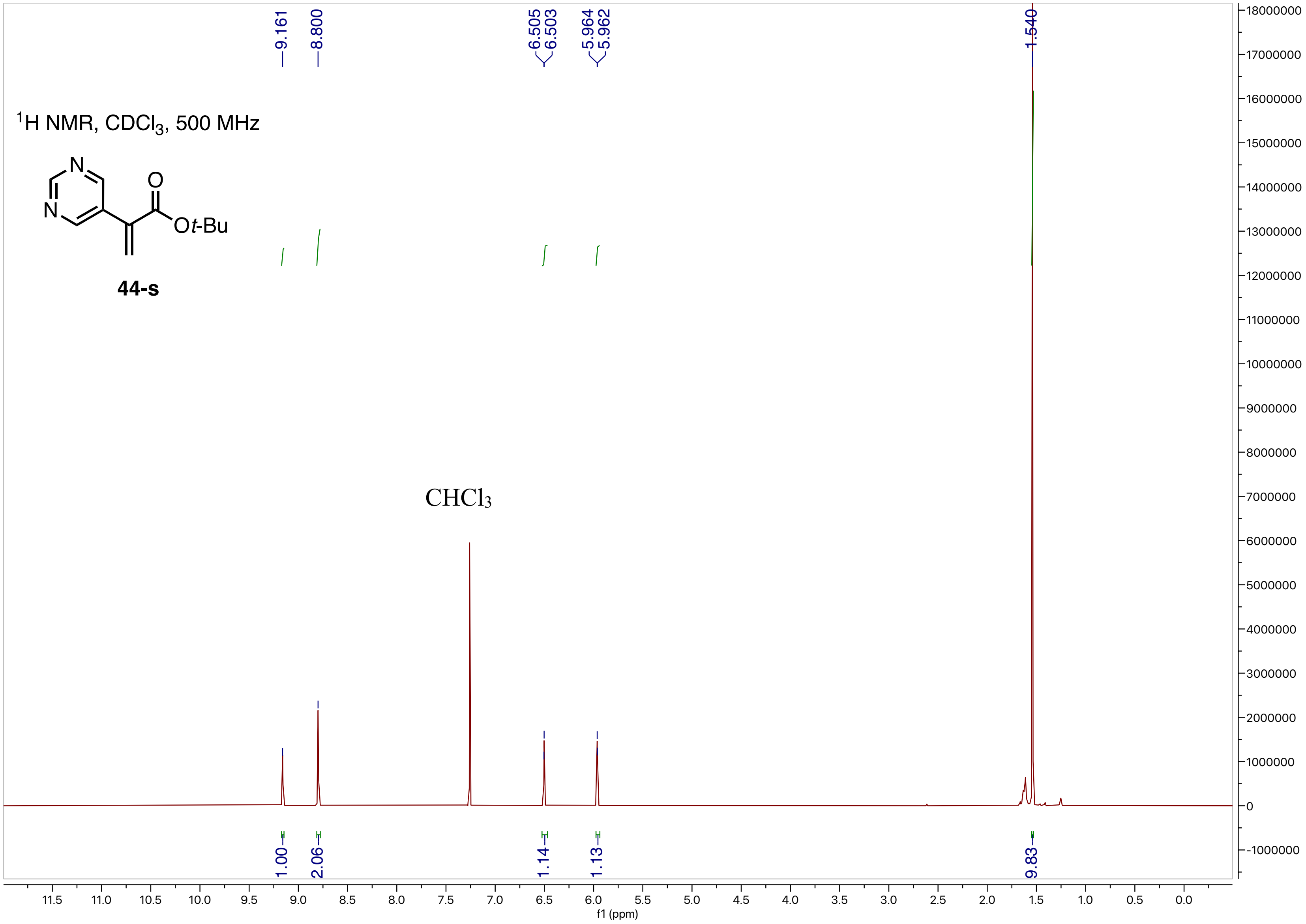

1H NMR, CDCl3, 500 MHz
44-s
CHCl3
9.161
8.800
6.505
6.503
5.964
5.962
1.540
1.00
2.06
1.14
1.13
9.83
f1 (ppm)

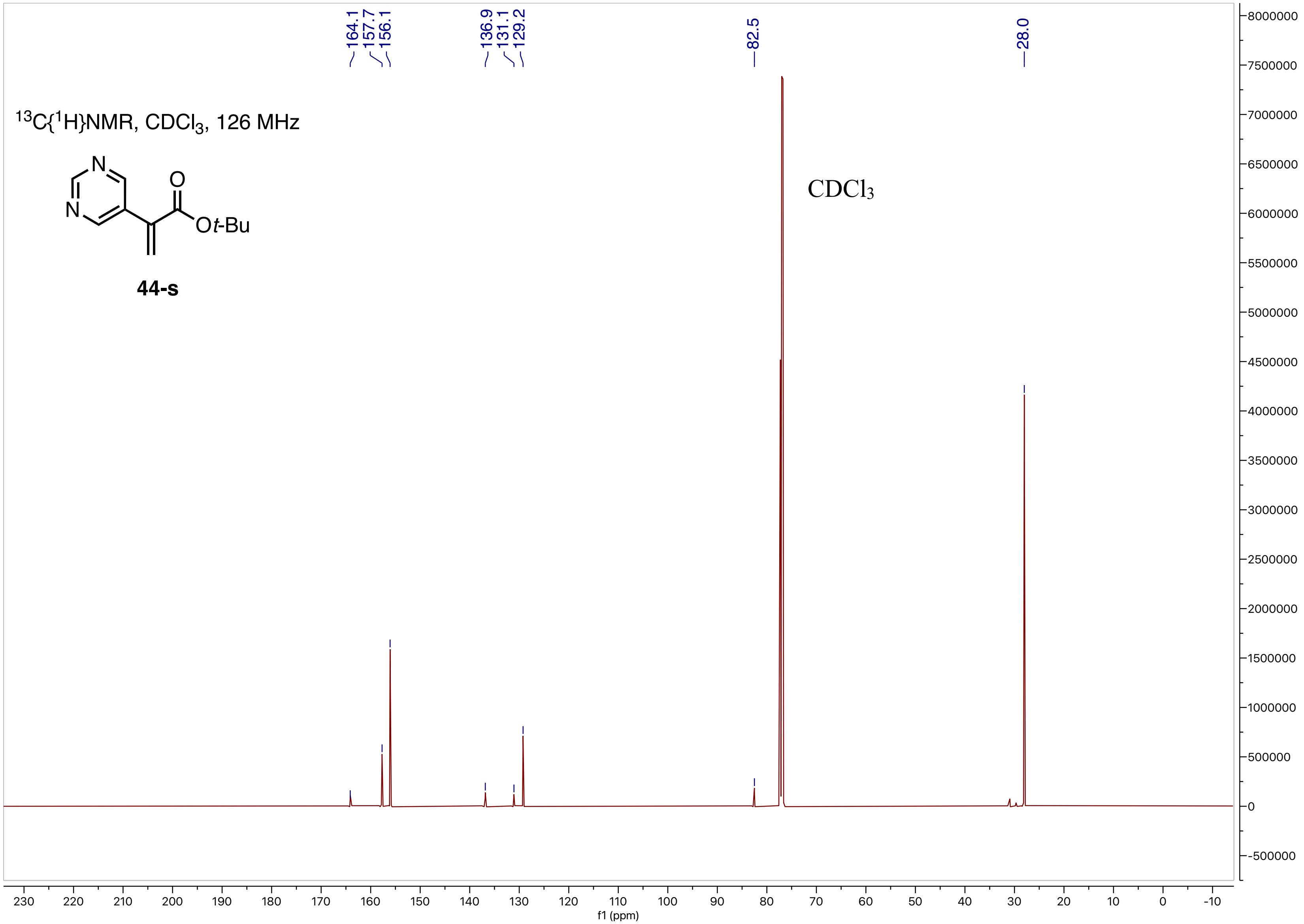

164.1
157.7
156.1
136.9
131.1
129.2
82.5
28.0
$^{13}C\{^1H\}NMR$, $CDCl_3$, 126 MHz
N
N
O
Ot-Bu
44-s
CDCl3
8000000
7500000
7000000
6500000
6000000
5500000
5000000
4500000
4000000
3500000
3000000
2500000
2000000
1500000
1000000
500000
0
-500000
230 220 210 200 190 180 170 160 150 140 130 120 110 100 90 80 70 60 50 40 30 20 10 0 -10
f1 (ppm)

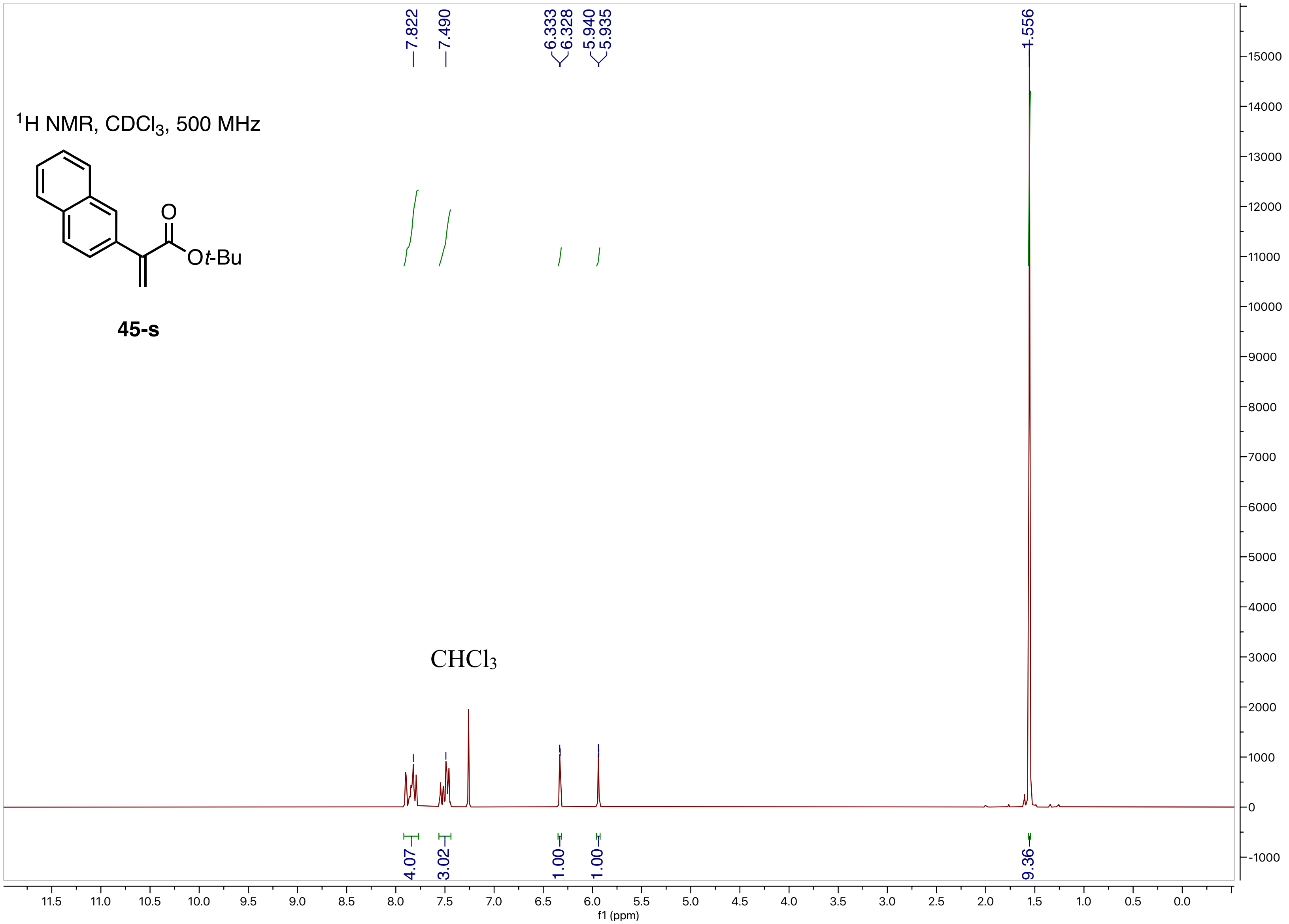

7.822
7.490
6.333
6.328
5.940
5.935
1.556
1H NMR, CDCl3, 500 MHz
O
Ot-Bu
45-s
CHCl3
4.07
3.02
1.00
1.00
9.36
f1 (ppm)

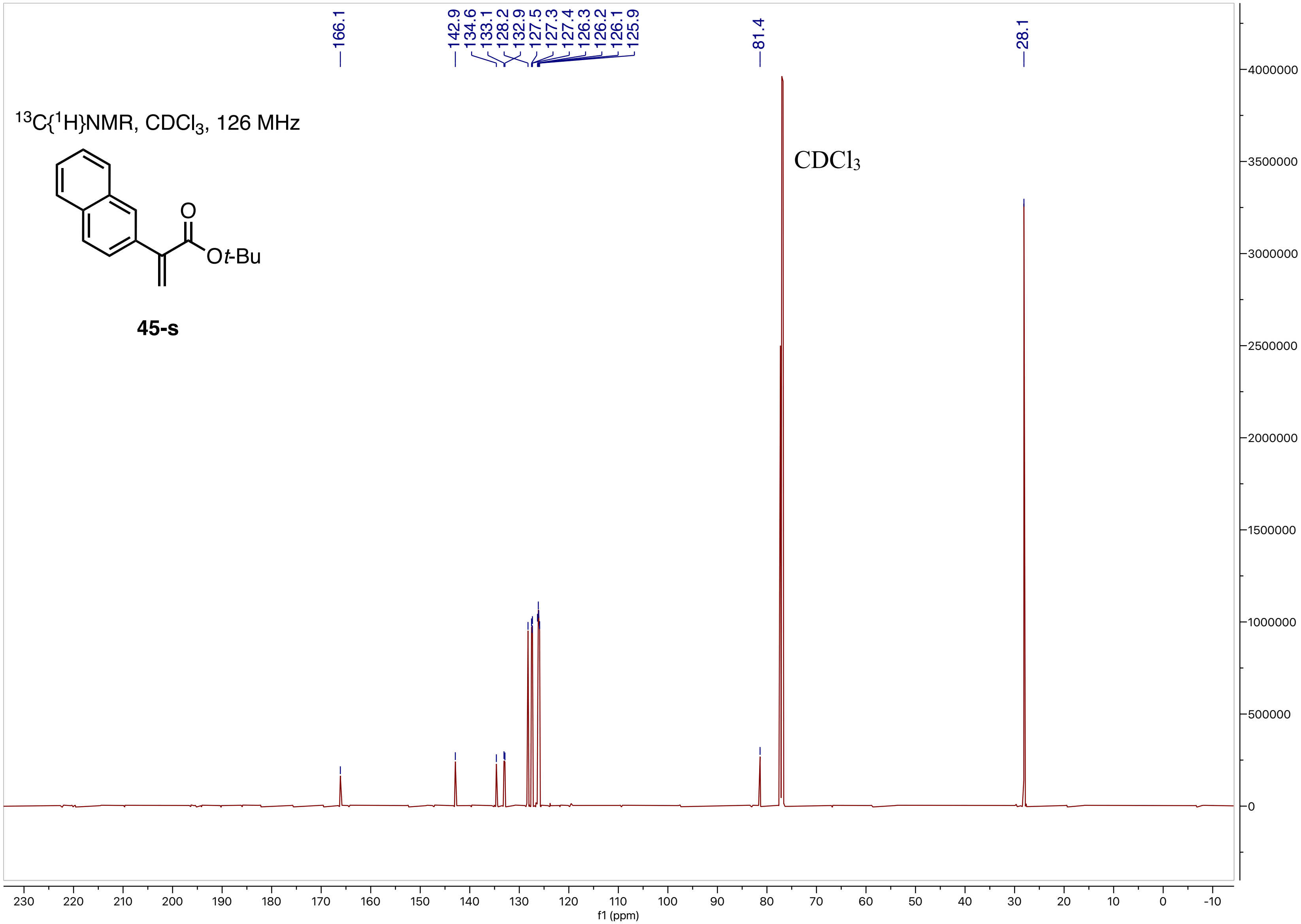

166.1
142.9
134.6
133.1
128.2
132.9
127.5
127.3
127.4
126.3
126.2
126.1
125.9
81.4
28.1
13C{1H}NMR, CDCl3, 126 MHz
CDCl3
f1 (ppm)
O
Ot-Bu

45-s

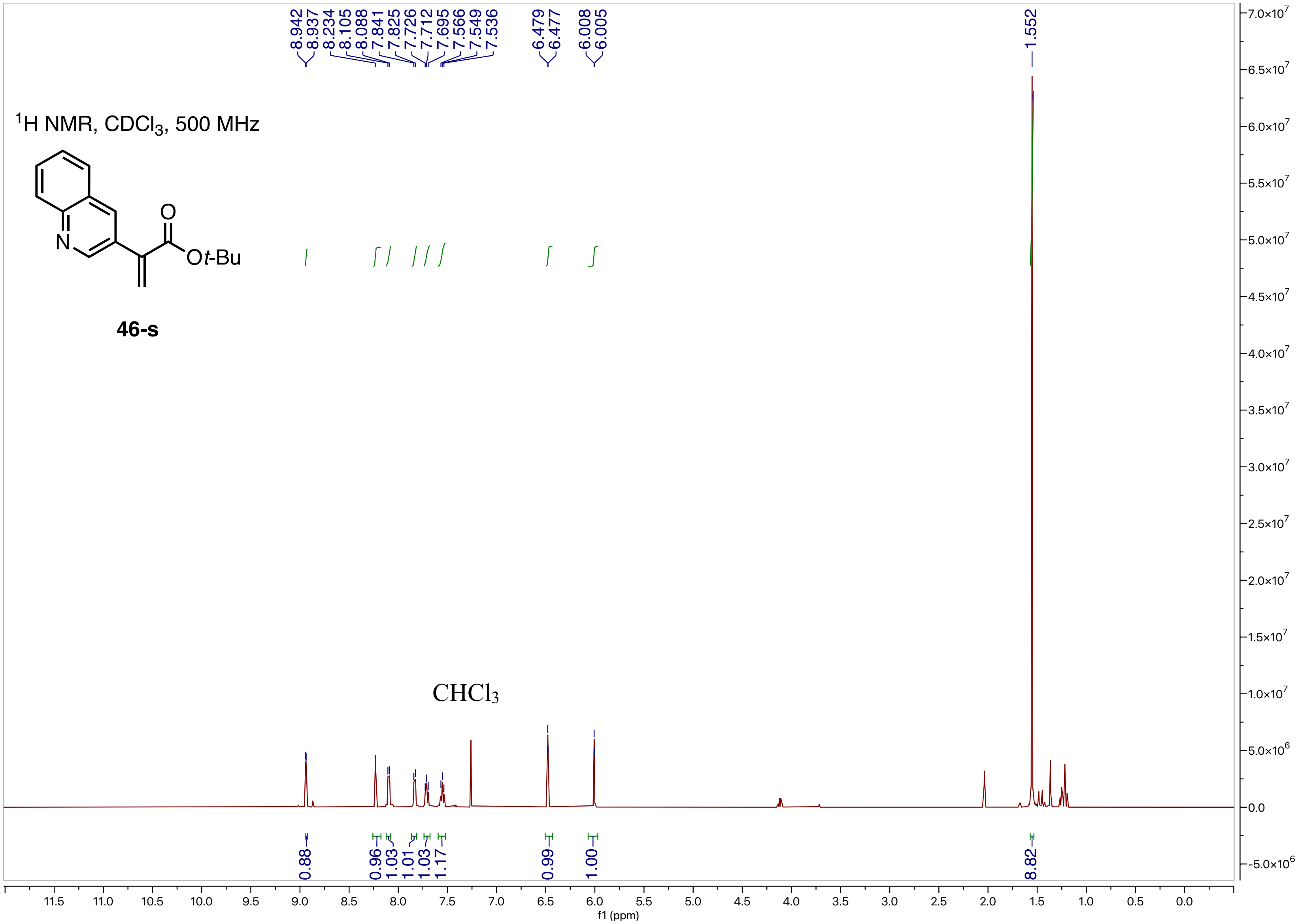

^{1}H NMR, CDCl$_3$, 500 MHz
N
O
O*t*-Bu
46-s
8.942
8.937
8.234
8.105
8.088
7.841
7.825
7.726
7.712
7.695
7.566
7.549
7.536
6.479
6.477
6.008
6.005
1.552
CHCl$_3$
0.88
0.96
1.03
1.01
1.03
1.17
0.99
1.00
8.82
f1 (ppm)

$^{13}C\{^1H\}$NMR, $CDCl_3$, 126 MHz

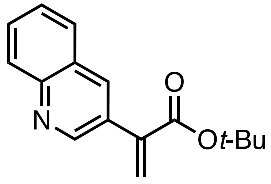

46-s

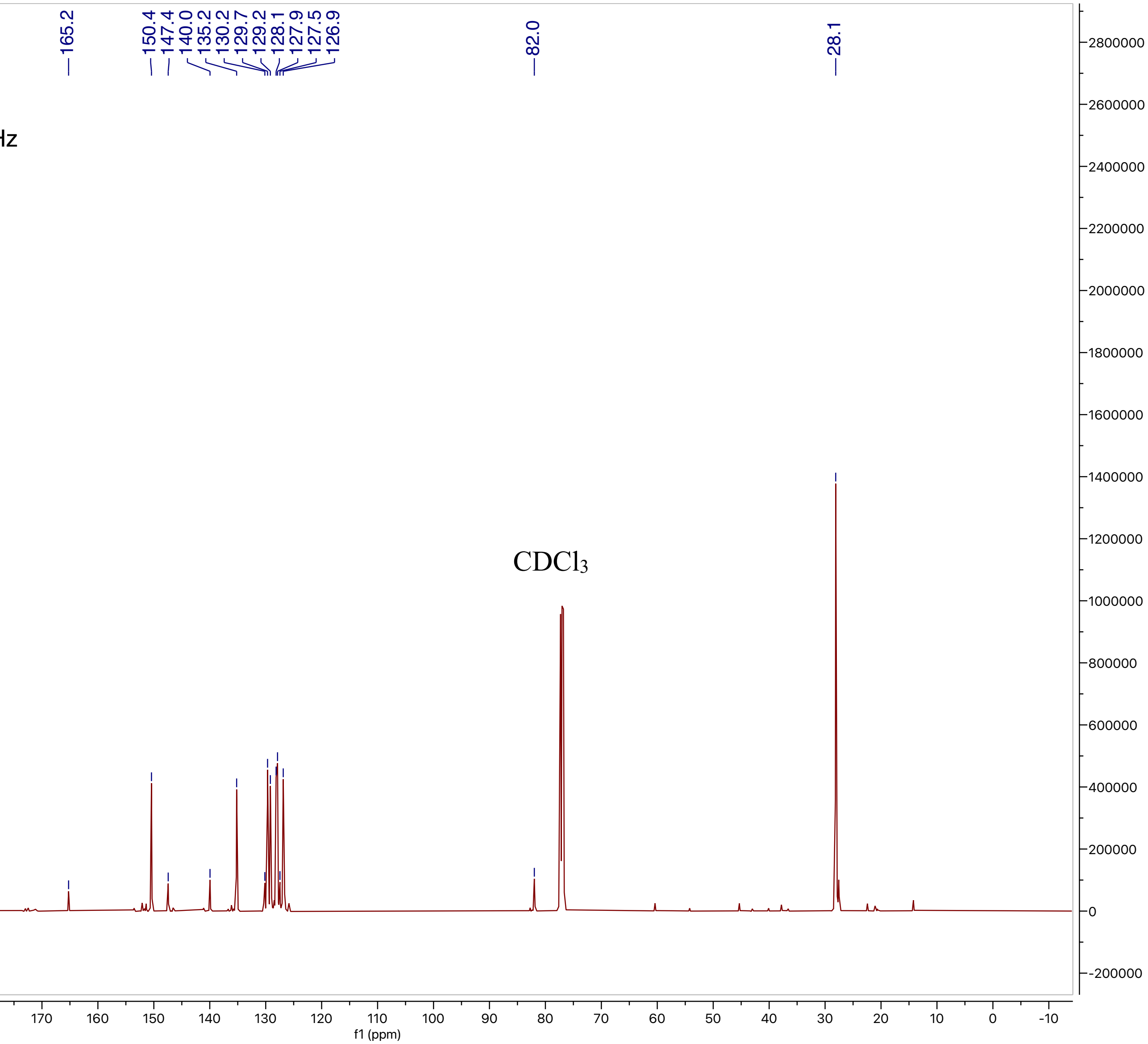

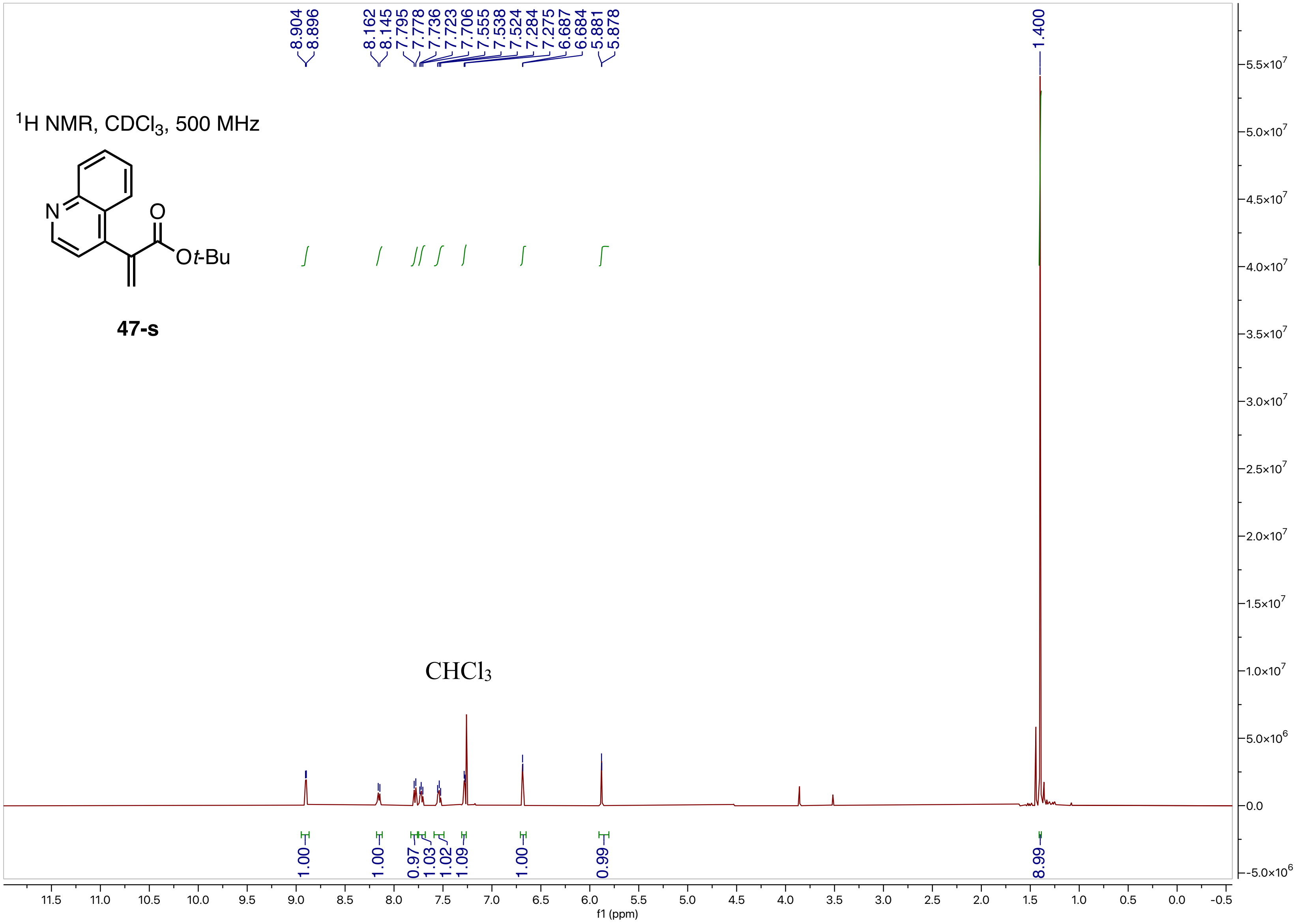

1H NMR, CDCl3, 500 MHz
47-s
CHCl3
f1 (ppm)

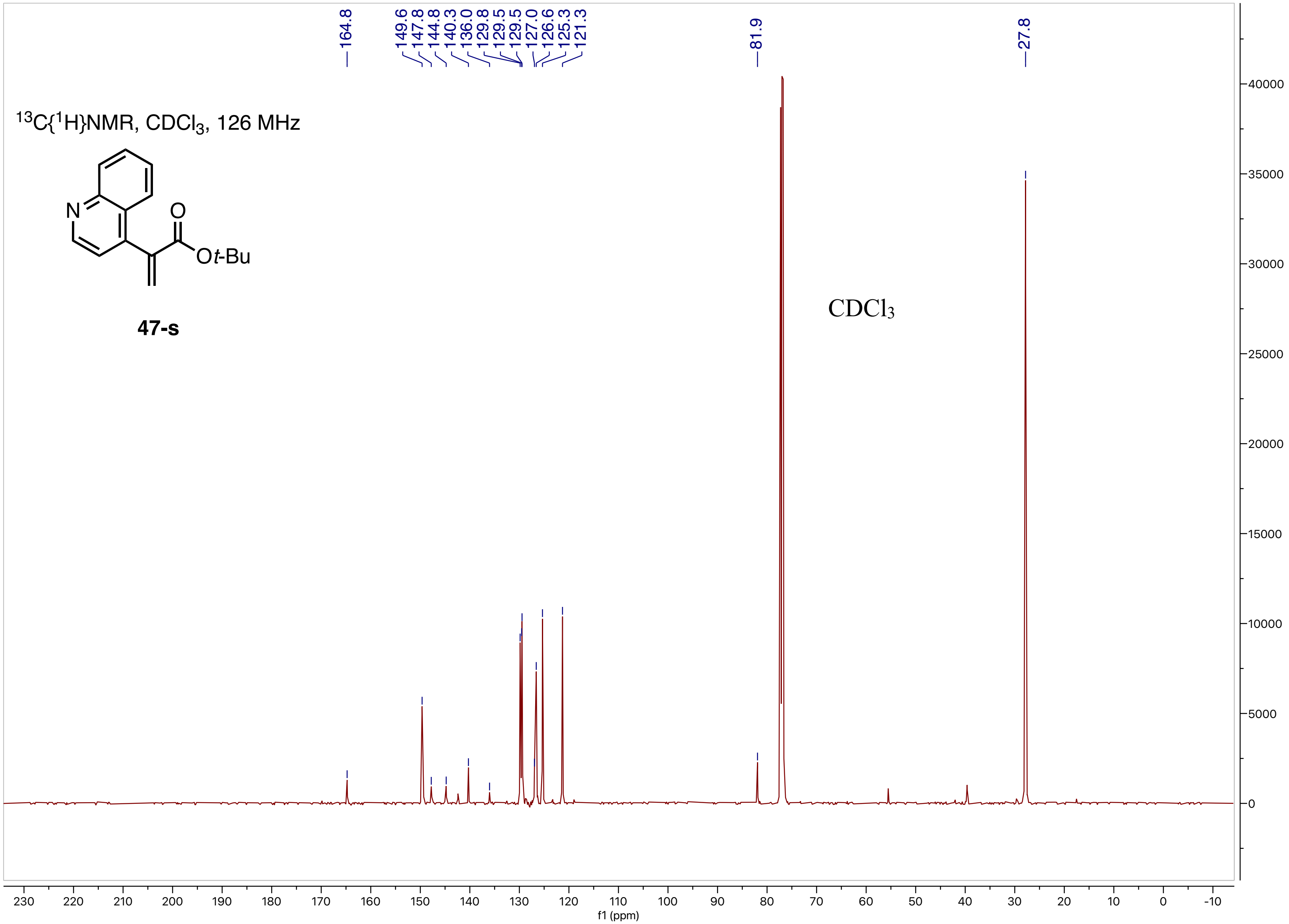

$^{13}C\{^{1}H\}$NMR, $CDCl_3$, 126 MHz
N
O
Ot-Bu
47-s
164.8
149.6
147.8
144.8
140.3
136.0
129.8
129.5
129.5
127.0
126.6
125.3
121.3
81.9
27.8
CDCl3
f1 (ppm)

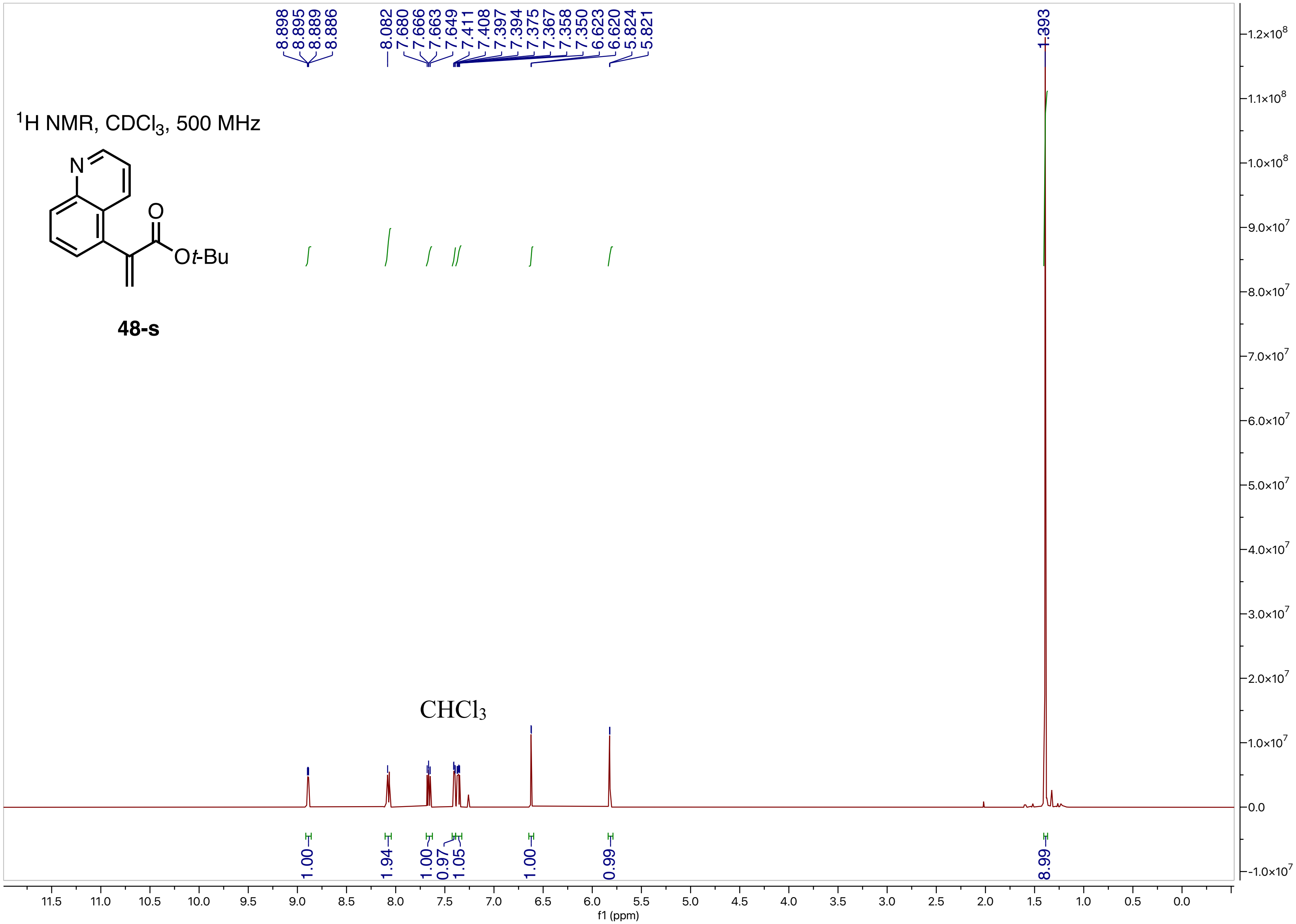

1H NMR, CDCl3, 500 MHz
N
O
Ot-Bu
48-s
CHCl3
8.898
8.895
8.889
8.886
8.082
7.680
7.666
7.663
7.649
7.411
7.408
7.397
7.394
7.375
7.367
7.358
7.350
6.623
6.620
5.824
5.821
1.393
1.00
1.94
1.00
0.97
1.05
1.00
0.99
8.99
f1 (ppm)

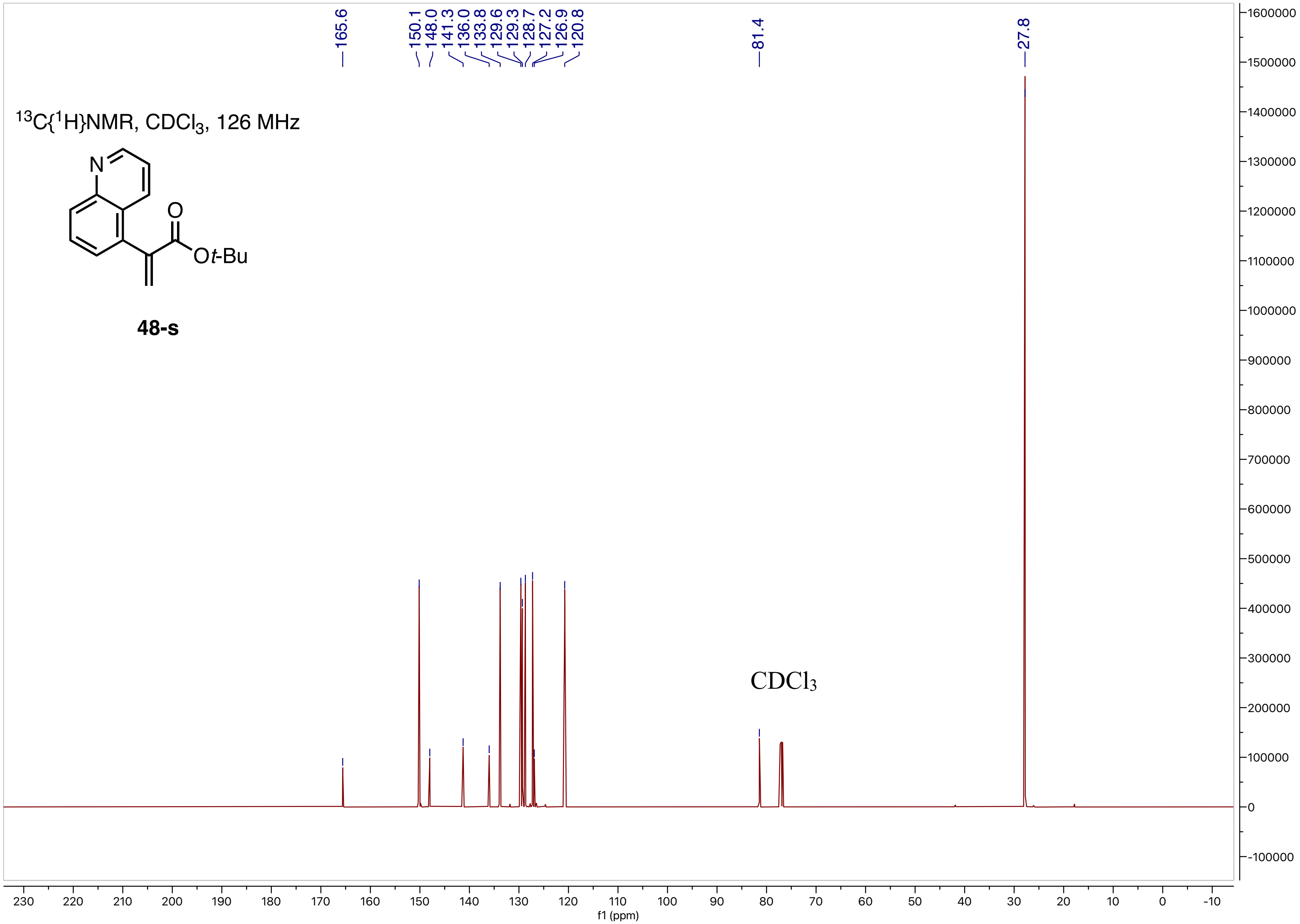

$^{13}C\{^1H\}$NMR, $CDCl_3$, 126 MHz
48-s
165.6
150.1
148.0
141.3
136.0
133.8
129.6
129.3
128.7
127.2
126.9
120.8
81.4
27.8
CDCl3
f1 (ppm)

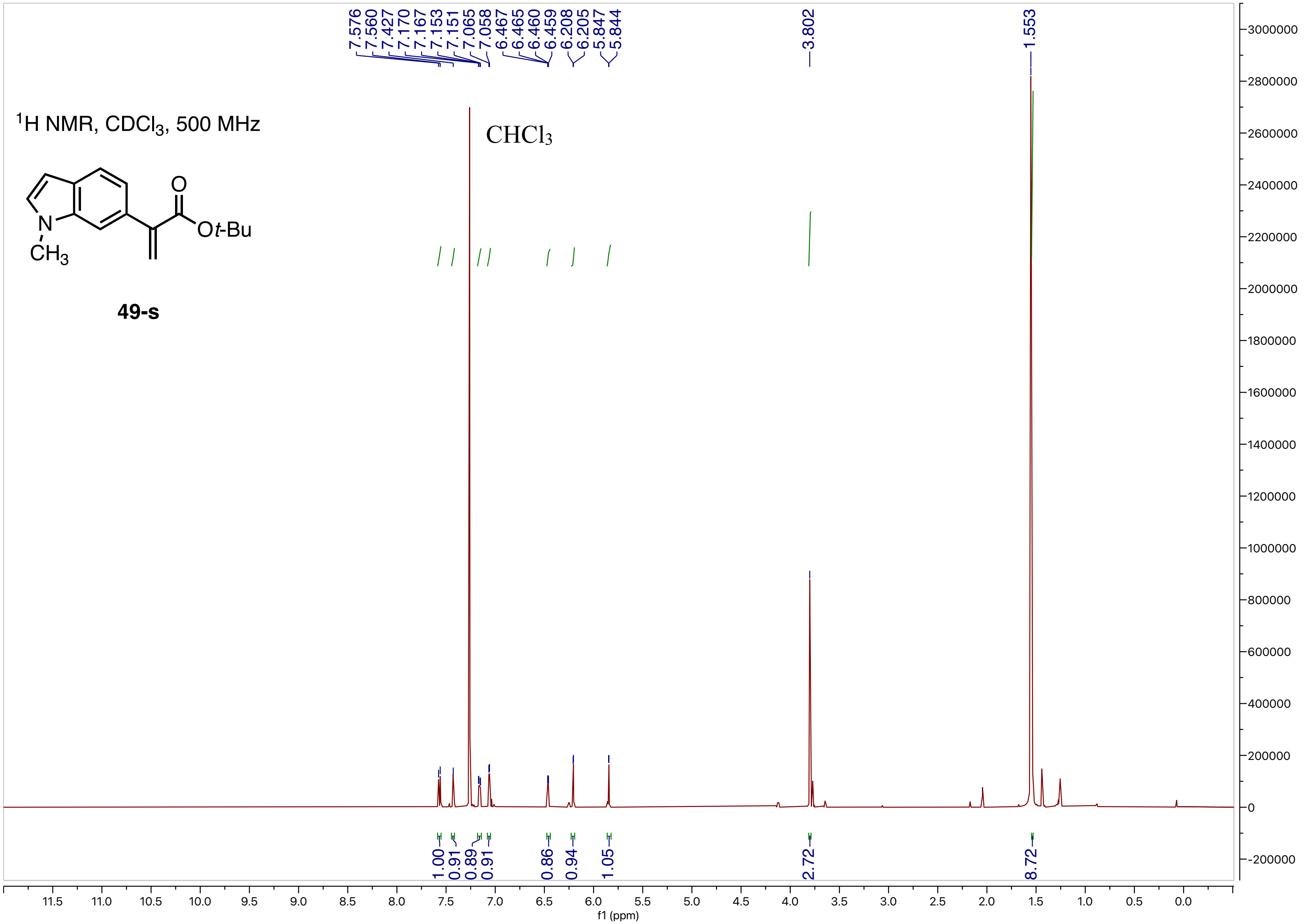

1H NMR, CDCl3, 500 MHz
49-s
O
Ot-Bu
N
CH3
CHCl3
7.576
7.560
7.427
7.170
7.167
7.153
7.151
7.065
7.058
6.467
6.465
6.460
6.459
6.208
6.205
5.847
5.844
3.802
1.553
1.00
0.91
0.89
0.91
0.86
0.94
1.05
2.72
8.72
f1 (ppm)

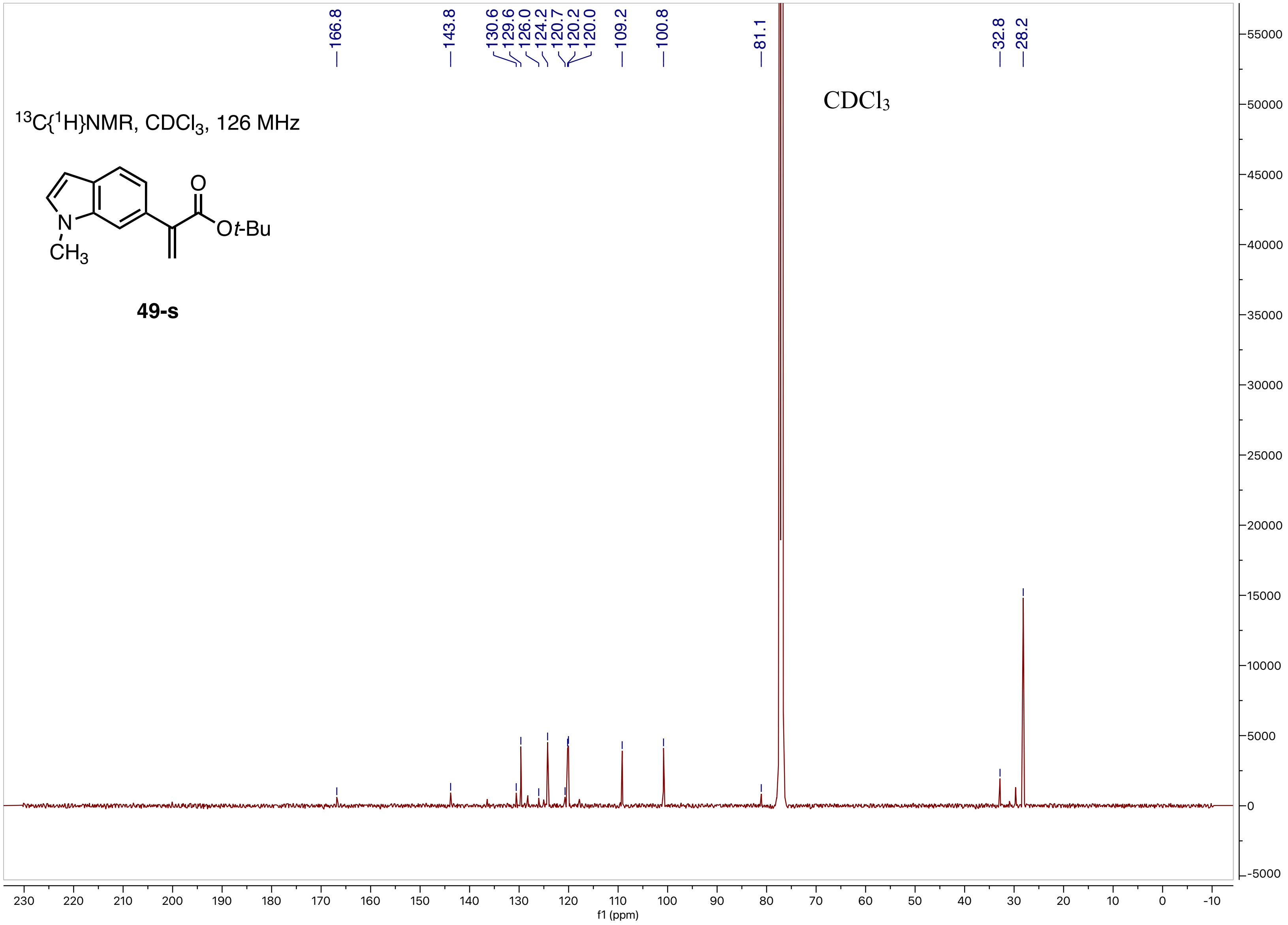
166.8
143.8
130.6
129.6
126.0
124.2
120.7
120.2
120.0
109.2
100.8
81.1
32.8
28.2
CDCl3
13C{1H}NMR, CDCl3, 126 MHz
O
Ot-Bu
N
CH3
49-s
f1 (ppm)

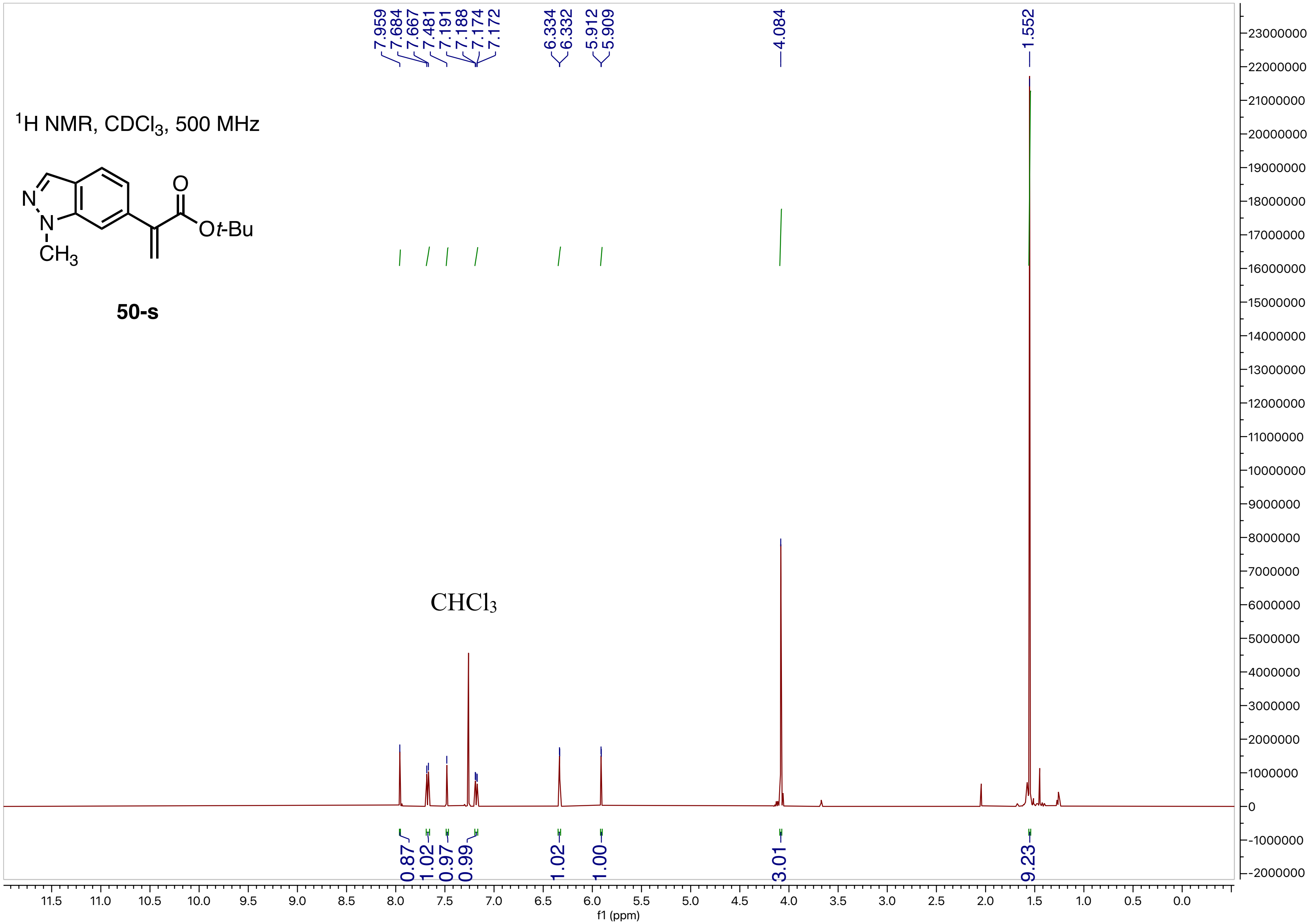

1H NMR, CDCl3, 500 MHz
50-s
CHCl3
7.959
7.684
7.667
7.481
7.191
7.188
7.174
7.172
6.334
6.332
5.912
5.909
4.084
1.552
0.87
1.02
0.97
0.99
1.02
1.00
3.01
9.23
f1 (ppm)

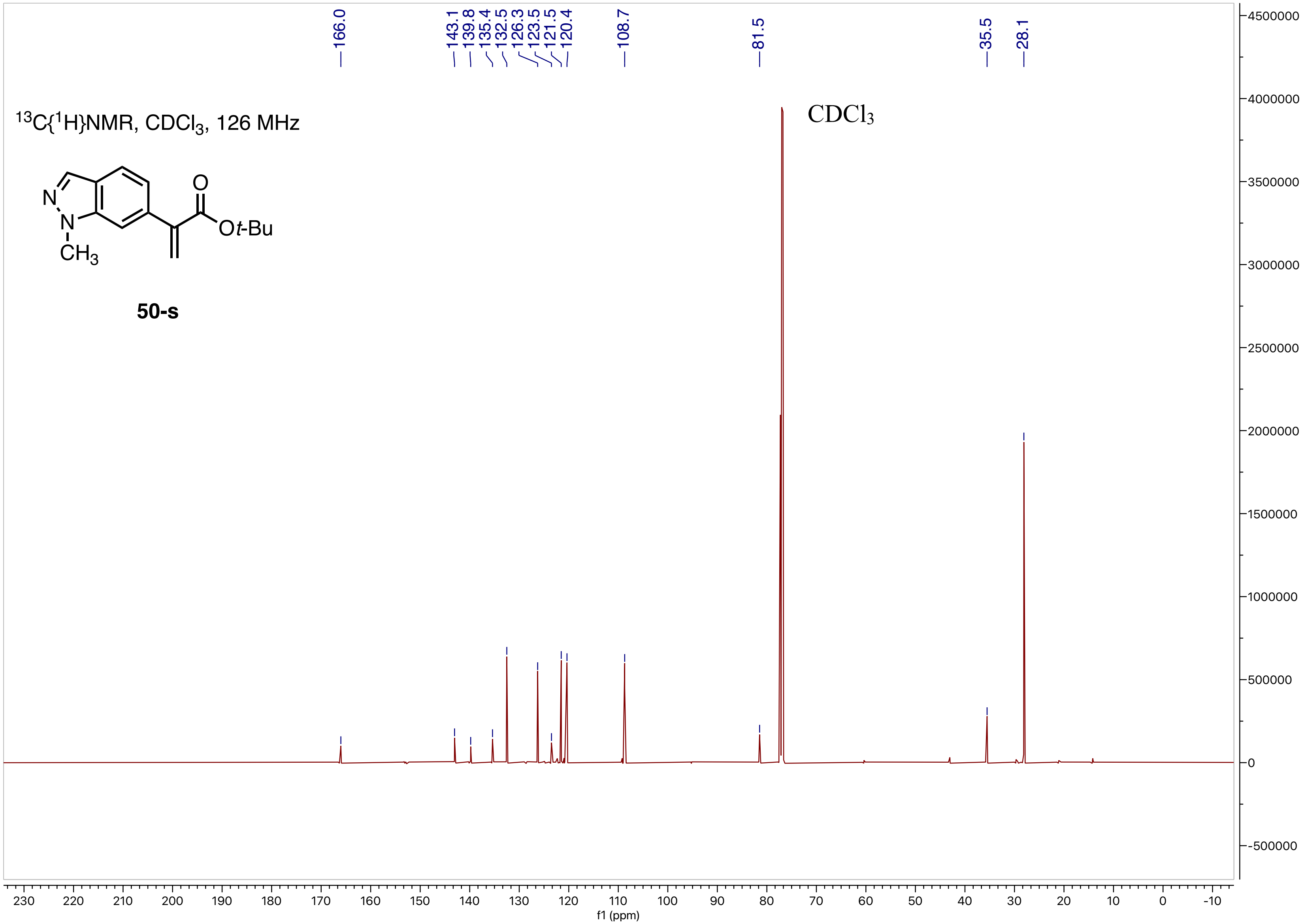

166.0
143.1
139.8
135.4
132.5
126.3
123.5
121.5
120.4
108.7
81.5
35.5
28.1
CDCl3
13C{1H}NMR, CDCl3, 126 MHz
N
N
CH3
O
Ot-Bu
50-s
f1 (ppm)

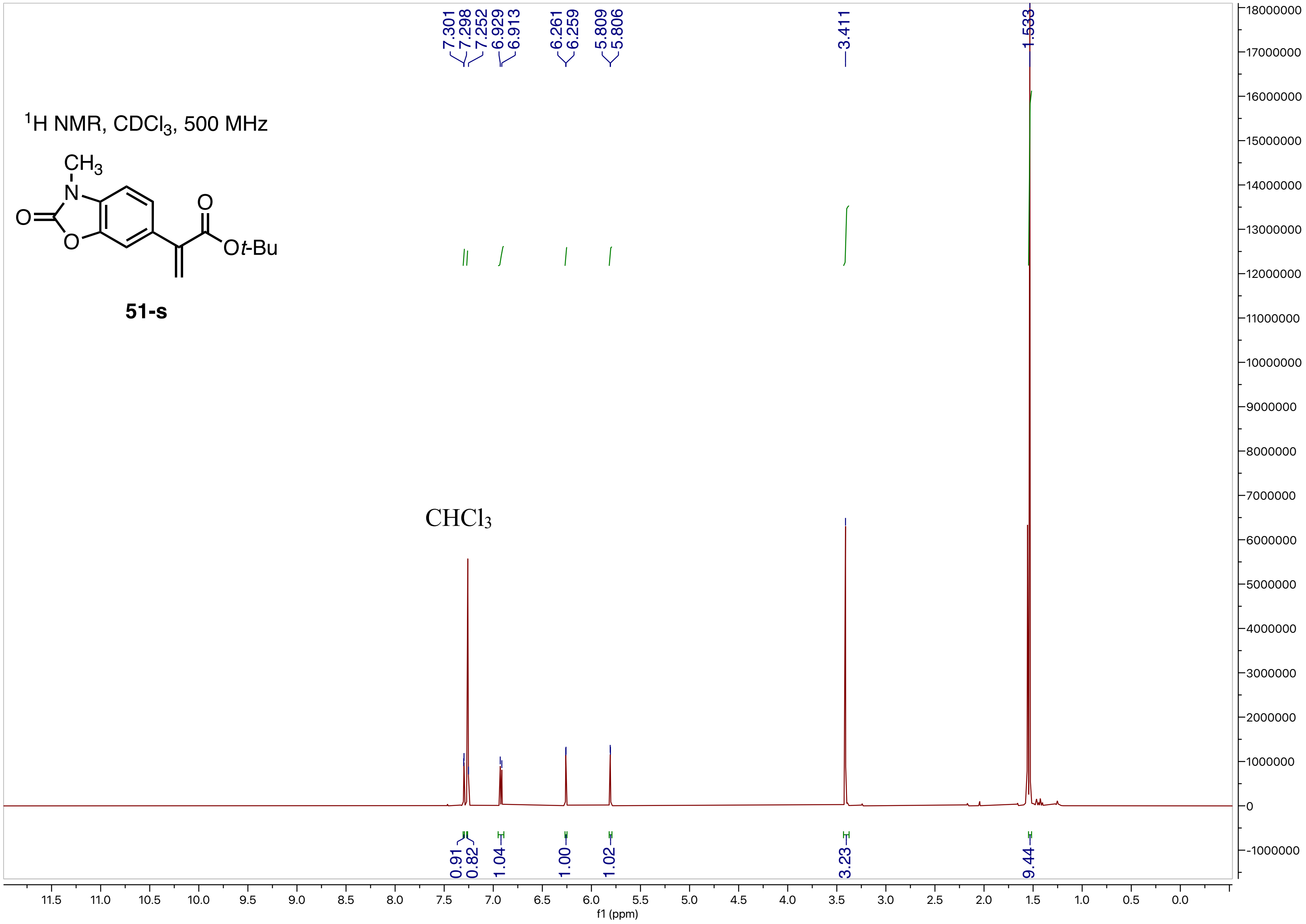

^1H NMR, $CDCl_3$, 500 MHz
51-s
CHCl3
7.301
7.298
7.252
6.929
6.913
6.261
6.259
5.809
5.806
3.411
1.533
0.91
0.82
1.04
1.00
1.02
3.23
9.44
f1 (ppm)

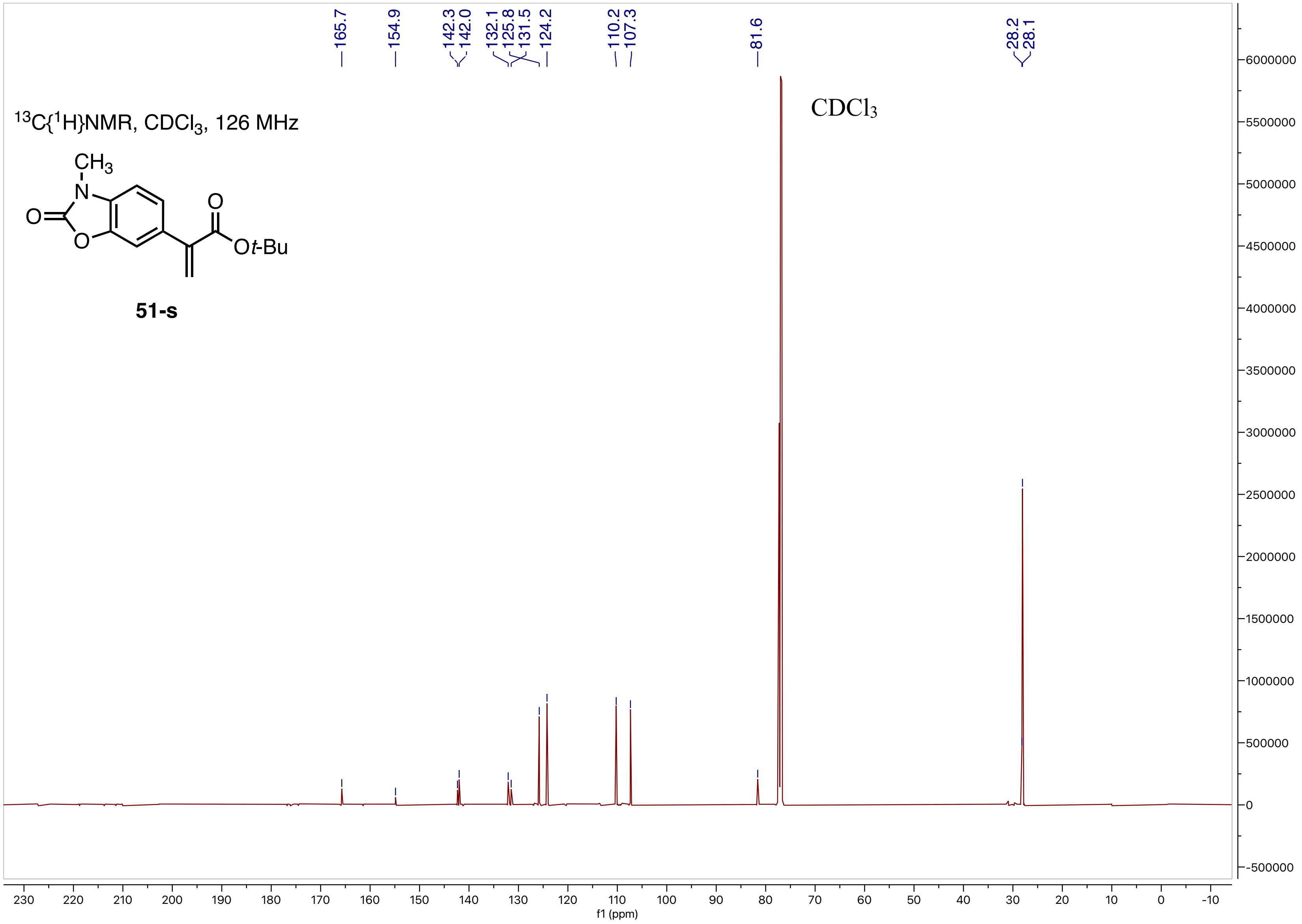

165.7
154.9
142.3
142.0
132.1
125.8
131.5
124.2
110.2
107.3
81.6
28.2
28.1
$^{13}C\{^1H\}$NMR, $CDCl_3$, 126 MHz
CH3
N
O
O
O
Ot-Bu
51-s
CDCl3
6000000
5500000
5000000
4500000
4000000
3500000
3000000
2500000
2000000
1500000
1000000
500000
0
-500000
230
220
210
200
190
180
170
160
150
140
130
120
110
100
90
80
70
60
50
40
30
20
10
0
-10
f1 (ppm)

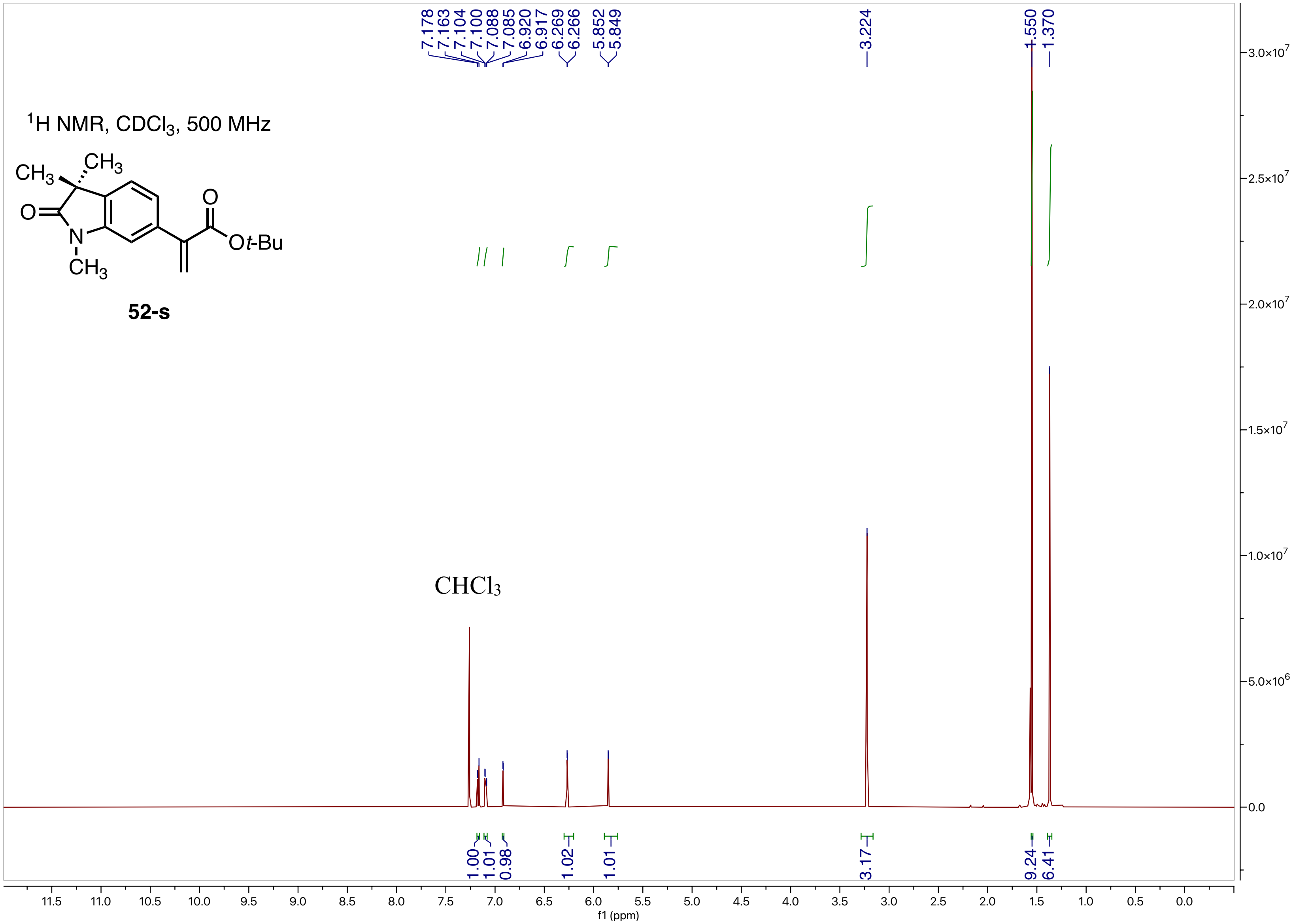

^{1}H NMR, $CDCl_3$, 500 MHz
52-s
CHCl3
7.178
7.163
7.104
7.100
7.088
7.085
6.920
6.917
6.269
6.266
5.852
5.849
3.224
1.550
1.370
1.00
1.01
0.98
1.02
1.01
3.17
9.24
6.41
f1 (ppm)

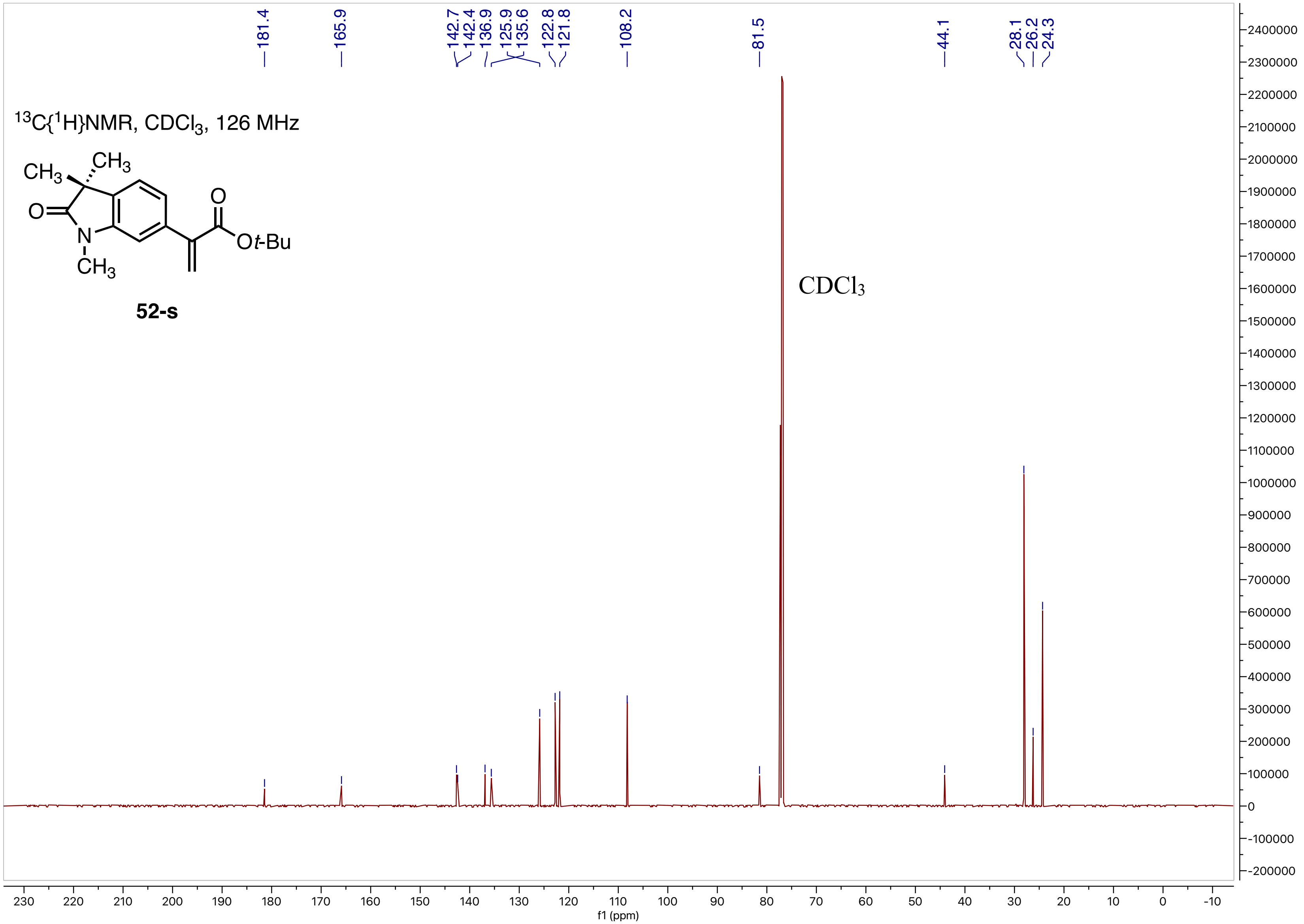
181.4
165.9
142.7
142.4
136.9
125.9
135.6
122.8
121.8
108.2
81.5
44.1
28.1
26.2
24.3
13C{1H}NMR, CDCl3, 126 MHz
CH3
CH3
O
N
CH3
O
Ot-Bu
52-s
CDCl3
f1 (ppm)

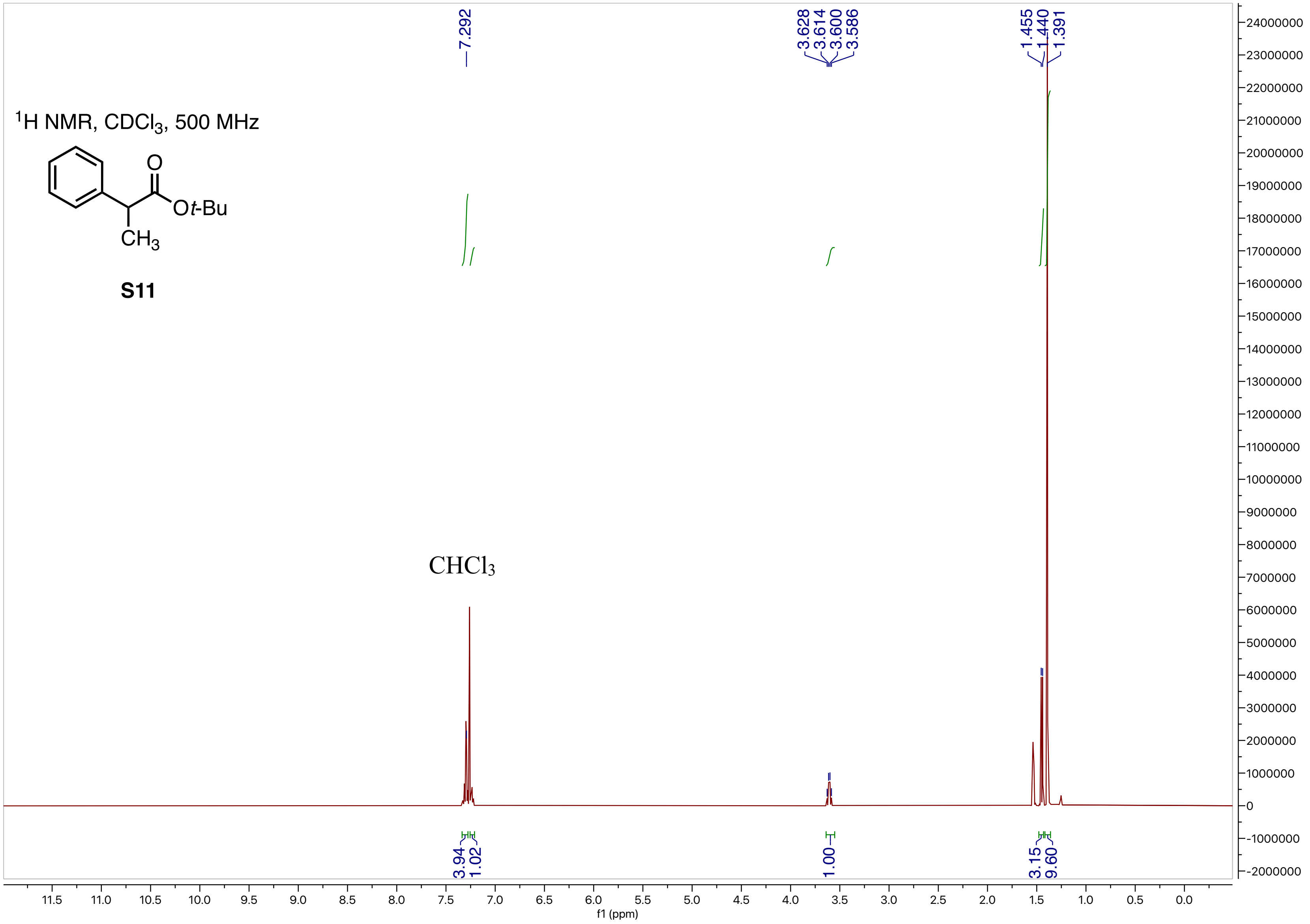
^{1}H NMR, $CDCl_3$, 500 MHz
O
Ot-Bu
CH_3
S11
7.292
3.628
3.614
3.600
3.586
1.455
1.440
1.391
$CHCl_3$
3.94
1.02
1.00
3.15
9.60
f1 (ppm)

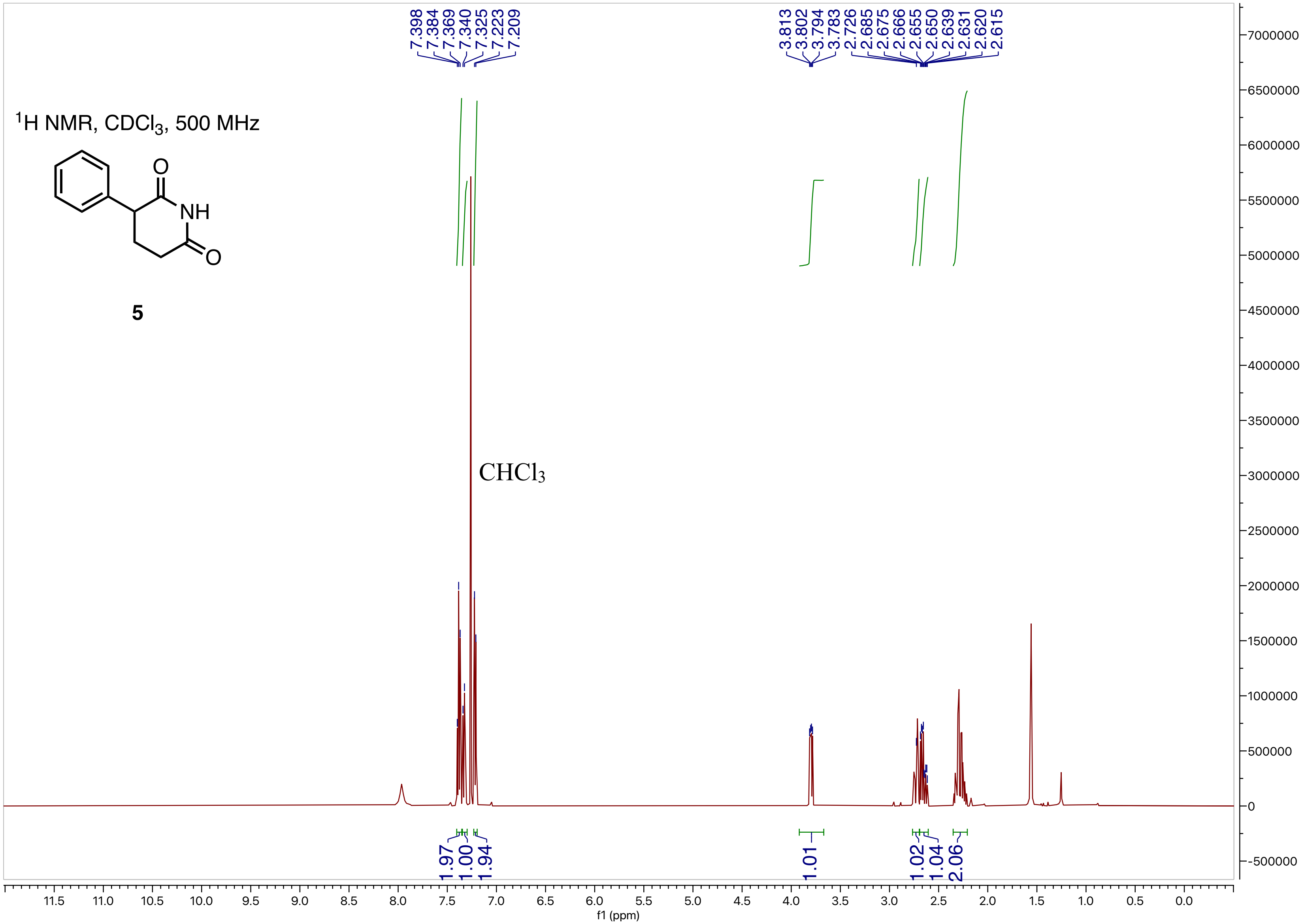

^{1}H NMR, CDCl$_3$, 500 MHz
5
CHCl$_3$
7.398
7.384
7.369
7.340
7.325
7.223
7.209
3.813
3.802
3.794
3.783
2.726
2.685
2.675
2.666
2.655
2.650
2.639
2.631
2.620
2.615
1.97
1.00
1.94
1.01
1.02
1.04
2.06
f1 (ppm)

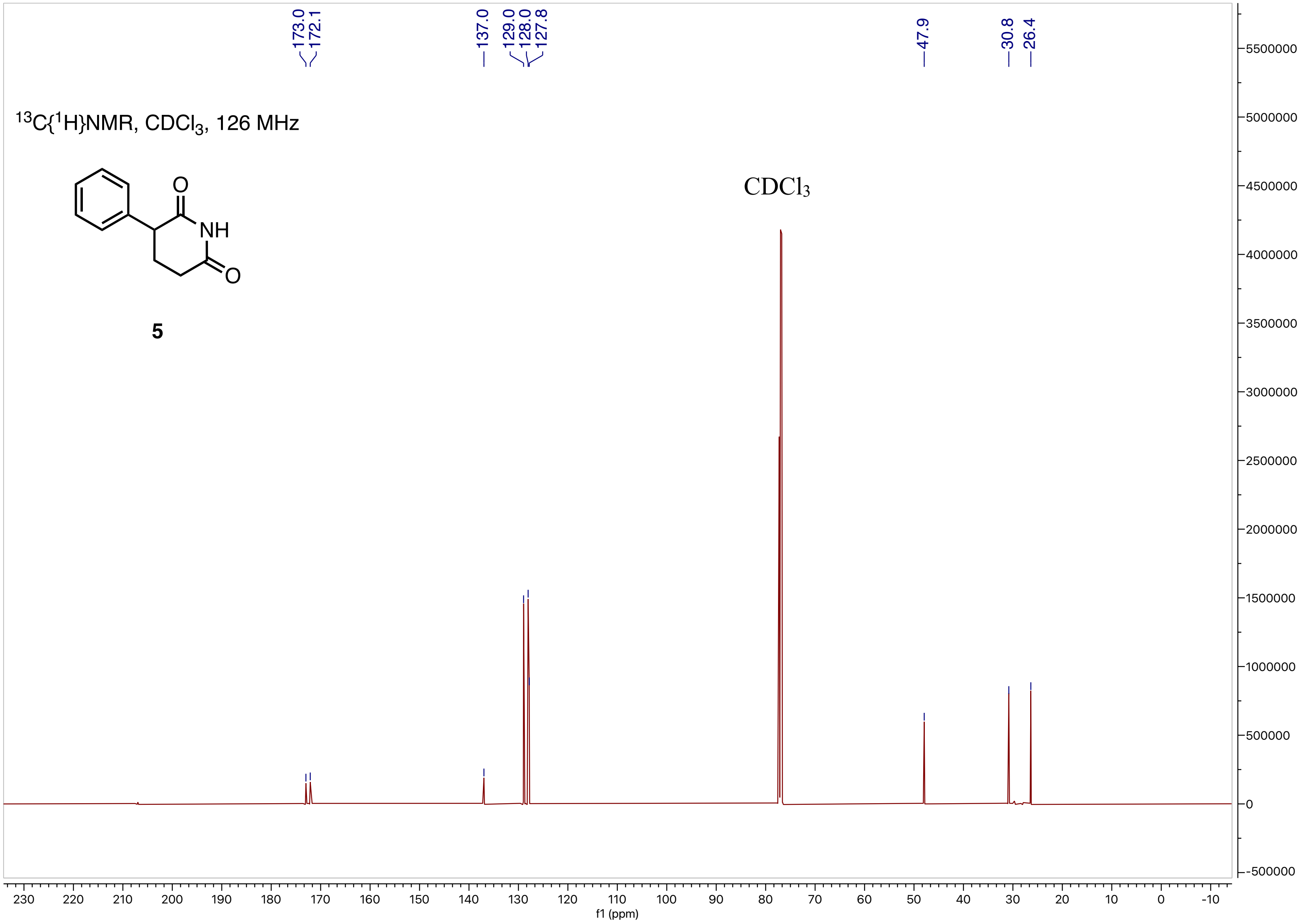

173.0
172.1
137.0
129.0
128.0
127.8
47.9
30.8
26.4
$^{13}C\{^{1}H\}$NMR, $CDCl_3$, 126 MHz
O
NH
O
5
$CDCl_3$
5500000
5000000
4500000
4000000
3500000
3000000
2500000
2000000
1500000
1000000
500000
0
-500000
230 220 210 200 190 180 170 160 150 140 130 120 110 100 90 80 70 60 50 40 30 20 10 0 -10
f1 (ppm)

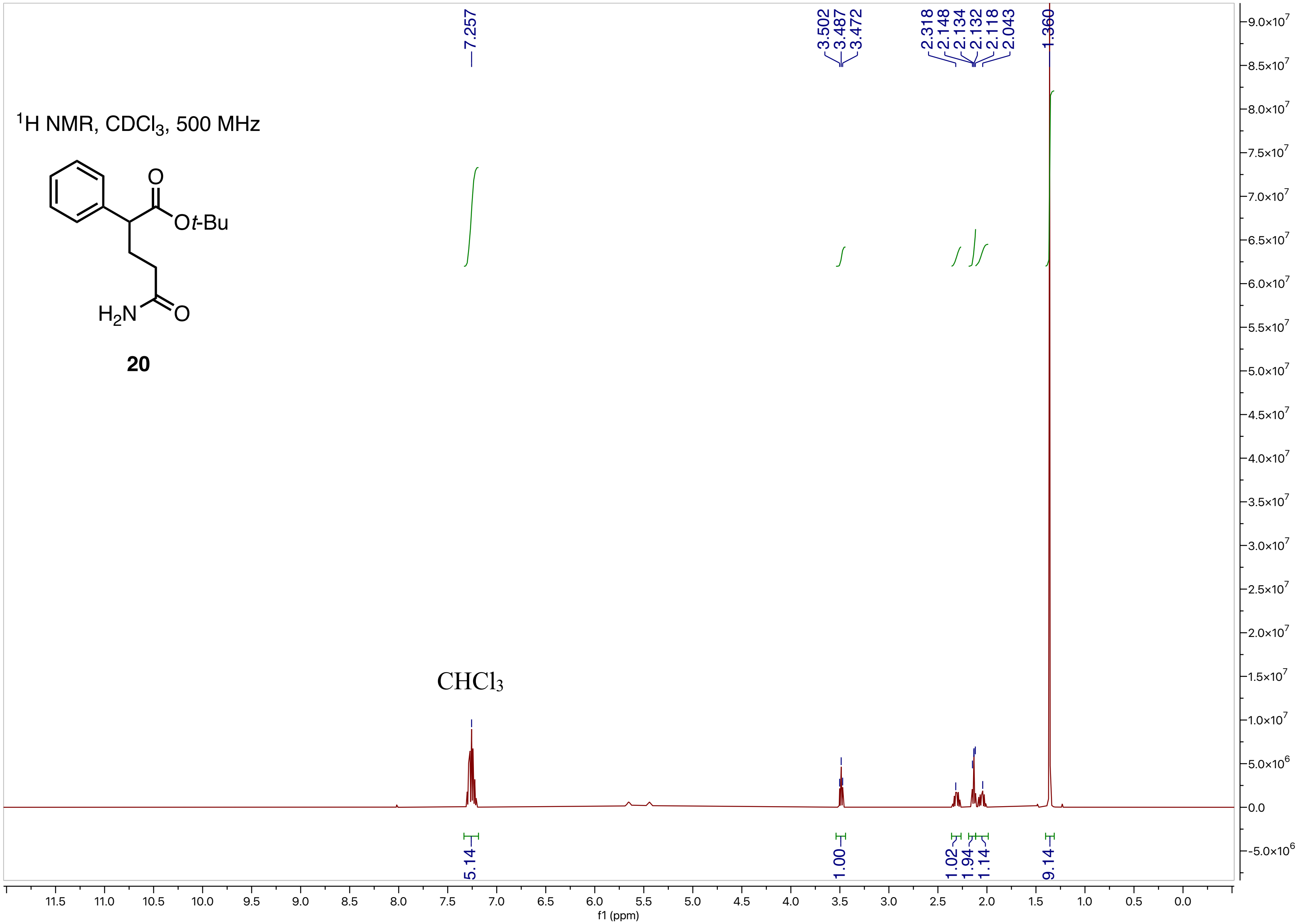

^{1}H NMR, $CDCl_3$, 500 MHz
O
Ot-Bu
H_2N
O
20
7.257
3.502
3.487
3.472
2.318
2.148
2.134
2.132
2.118
2.043
1.360
CHCl3
5.14
1.00
1.02
1.94
1.14
9.14
f1 (ppm)

$^{13}C\{^1H\}$NMR, $CDCl_3$, 126 MHz

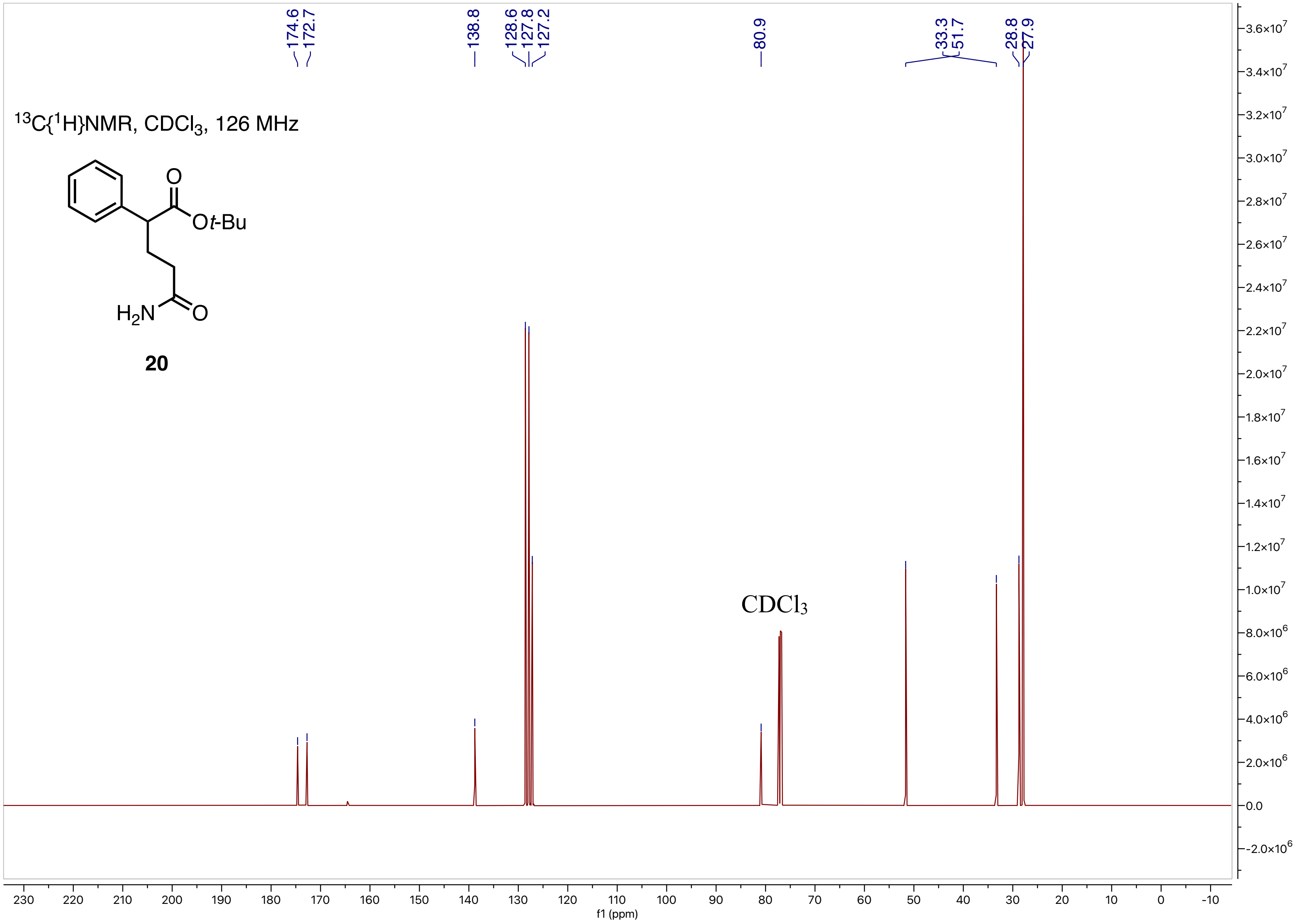

20

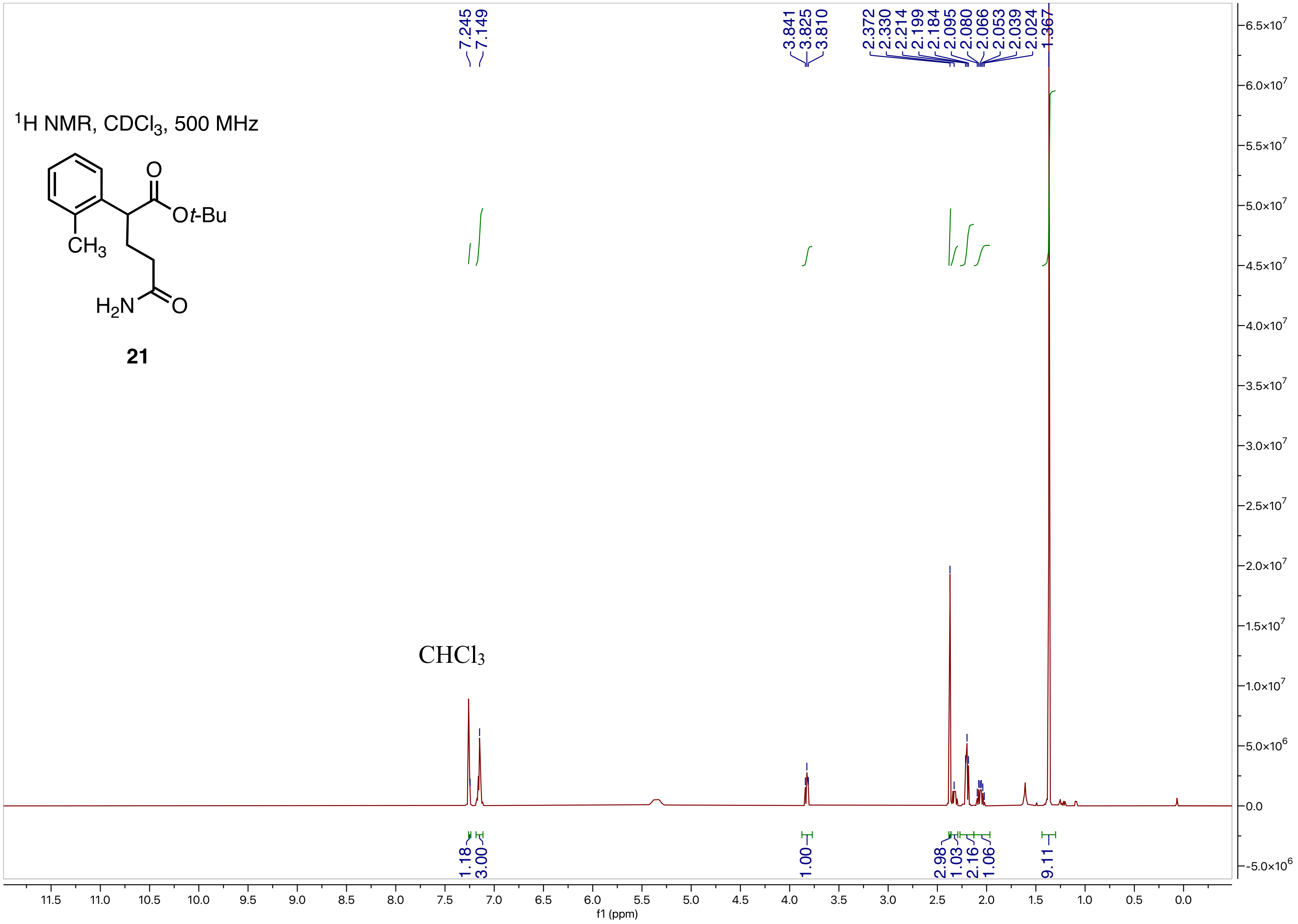

^{1}H NMR, CDCl$_3$, 500 MHz
O
O*t*-Bu
CH$_3$
H$_2$N
O
21
CHCl$_3$
7.245
7.149
3.841
3.825
3.810
2.372
2.330
2.214
2.199
2.184
2.095
2.080
2.066
2.053
2.039
2.024
1.367
1.18
3.00
1.00
2.98
1.03
2.16
1.06
9.11
f1 (ppm)

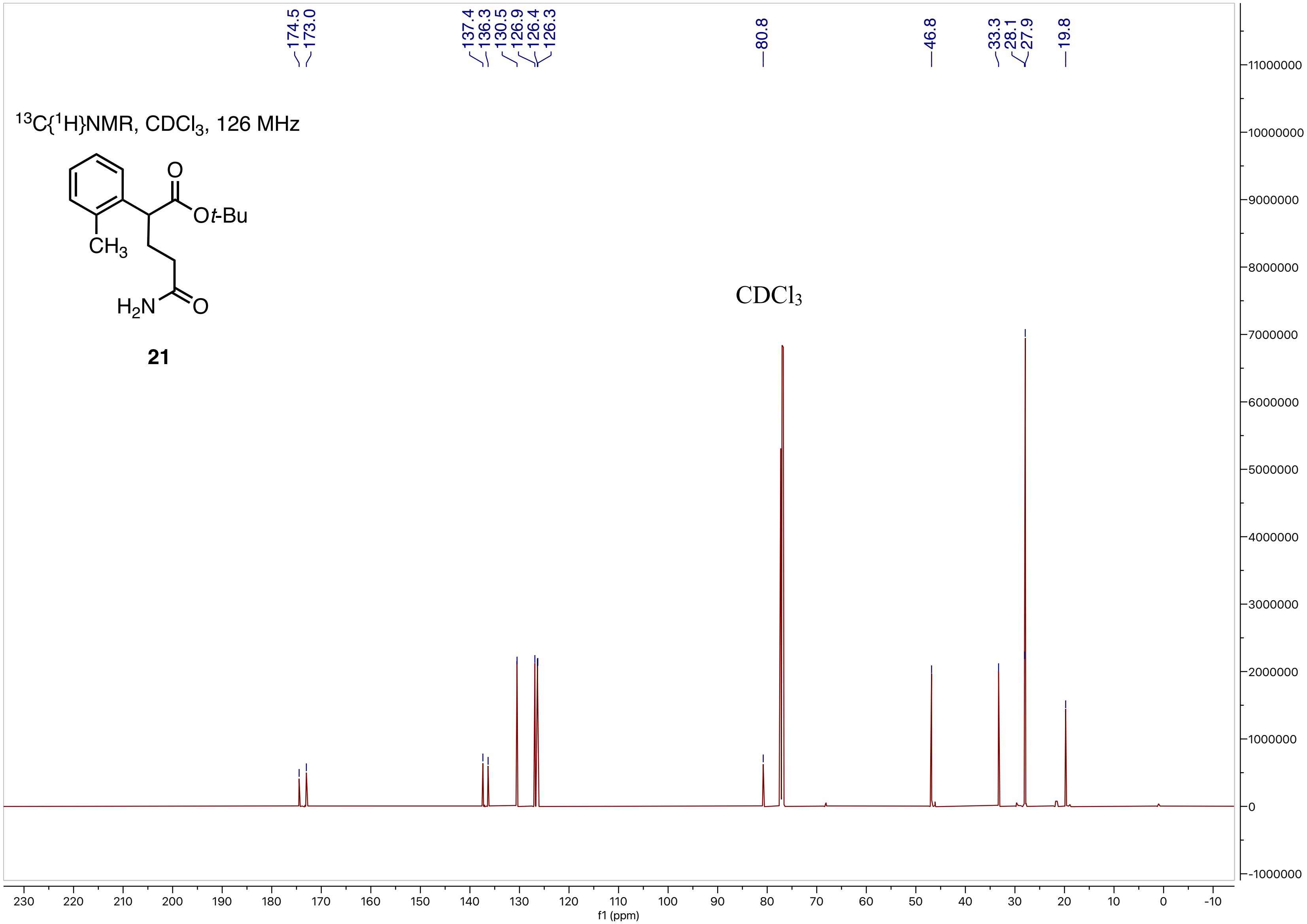

174.5
173.0
137.4
136.3
130.5
126.9
126.4
126.3
80.8
46.8
33.3
28.1
27.9
19.8
$^{13}C\{^1H\}$NMR, $CDCl_3$, 126 MHz
O
O*t*-Bu
CH_3
H_2N
O
21
$CDCl_3$
f1 (ppm)

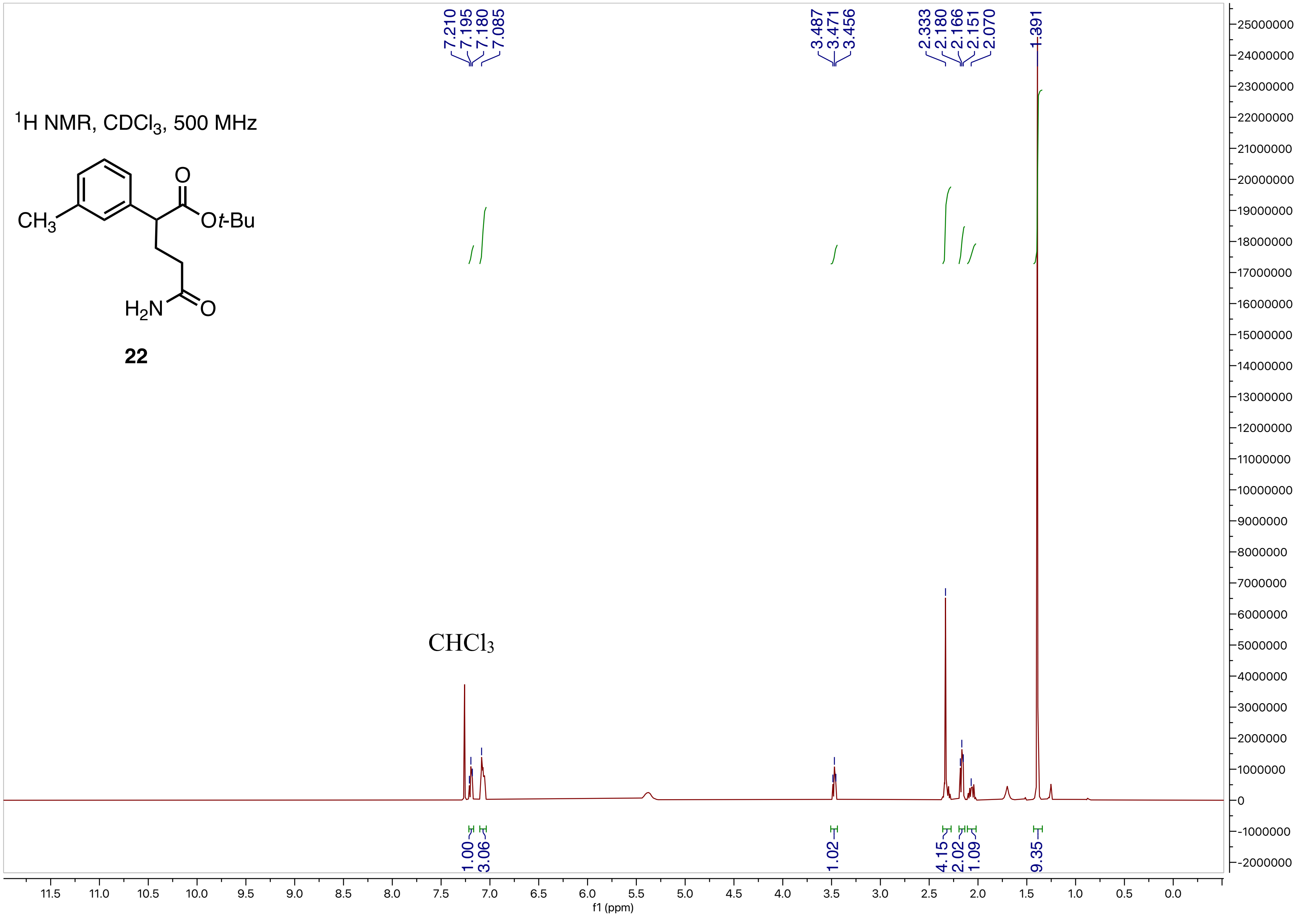

^{1}H NMR, $CDCl_3$, 500 MHz
CH_3
O
O*t*-Bu
H_2N
O
22
CHCl3
7.210
7.195
7.180
7.085
3.487
3.471
3.456
2.333
2.180
2.166
2.151
2.070
1.391
1.00
3.06
1.02
4.15
2.02
1.09
9.35
f1 (ppm)

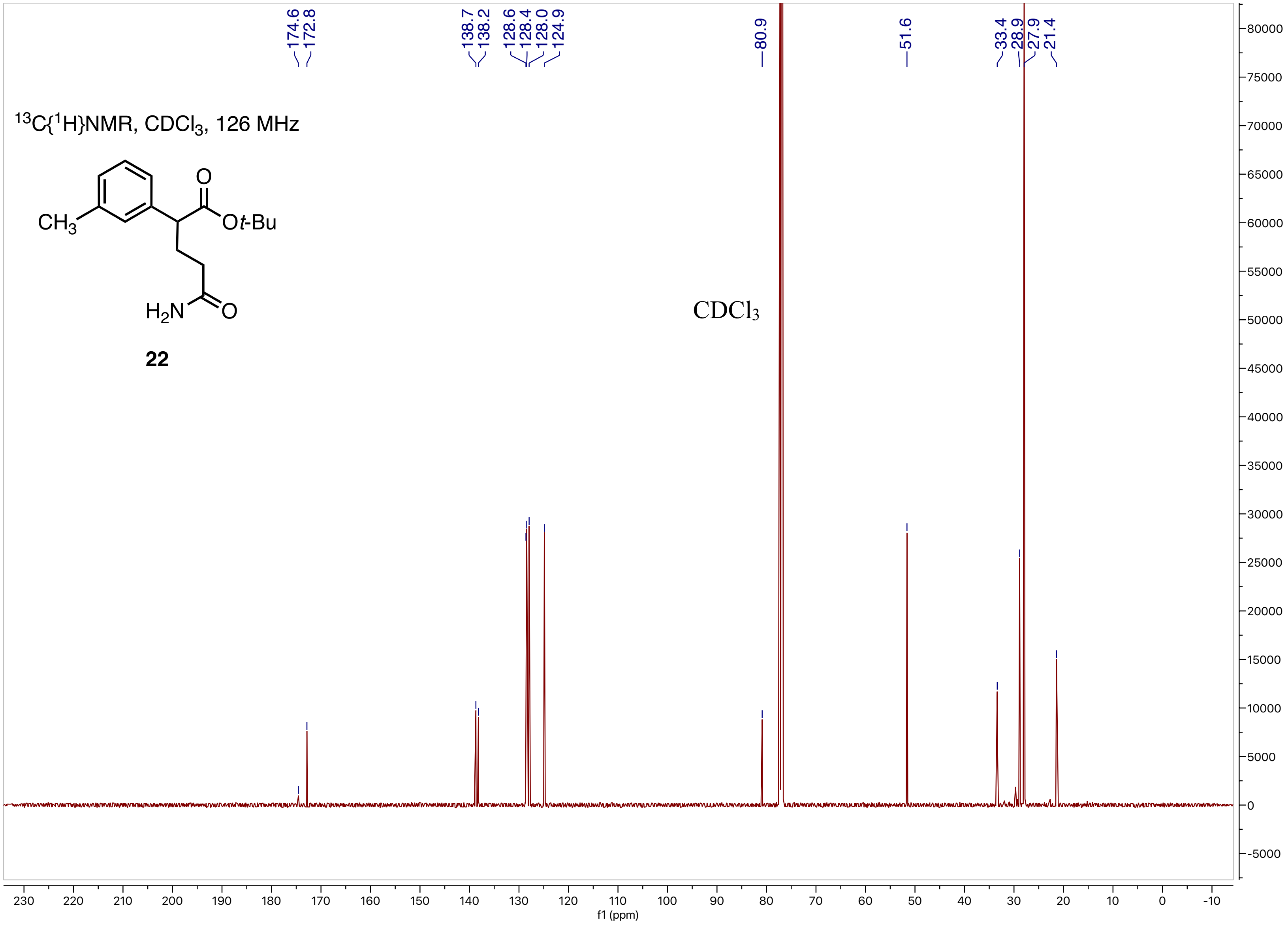

174.6
172.8
138.7
138.2
128.6
128.4
128.0
124.9
80.9
51.6
33.4
28.9
27.9
21.4
$^{13}C\{^1H\}$NMR, $CDCl_3$, 126 MHz
CH3
O
Ot-Bu
H2N
O
22
CDCl3
f1 (ppm)

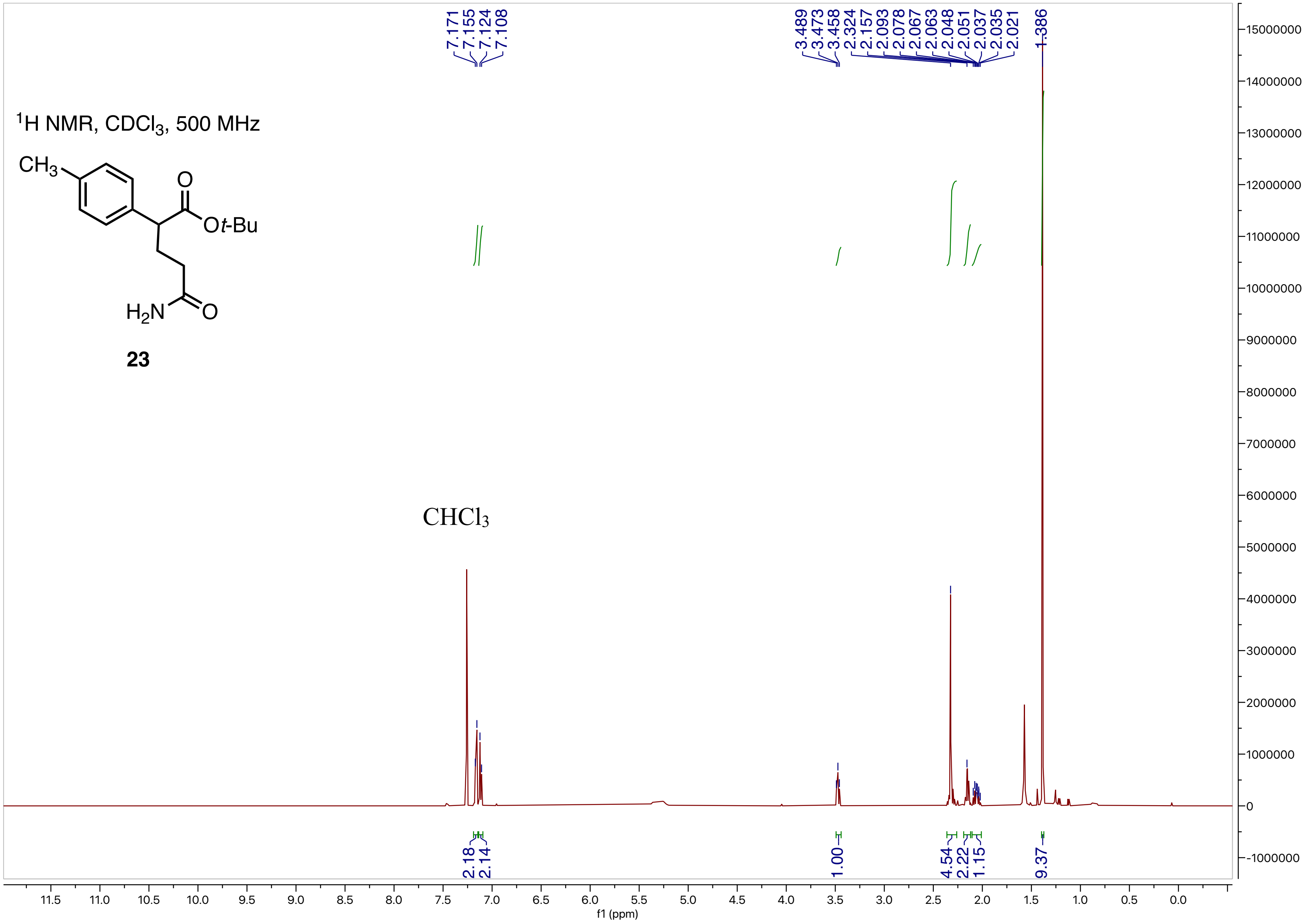

1H NMR, CDCl3, 500 MHz
CH3
O
Ot-Bu
H2N
O
23
CHCl3
7.171
7.155
7.124
7.108
3.489
3.473
3.458
2.324
2.157
2.093
2.078
2.067
2.063
2.048
2.051
2.037
2.035
2.021
1.386
2.18
2.14
1.00
4.54
2.22
1.15
9.37
f1 (ppm)

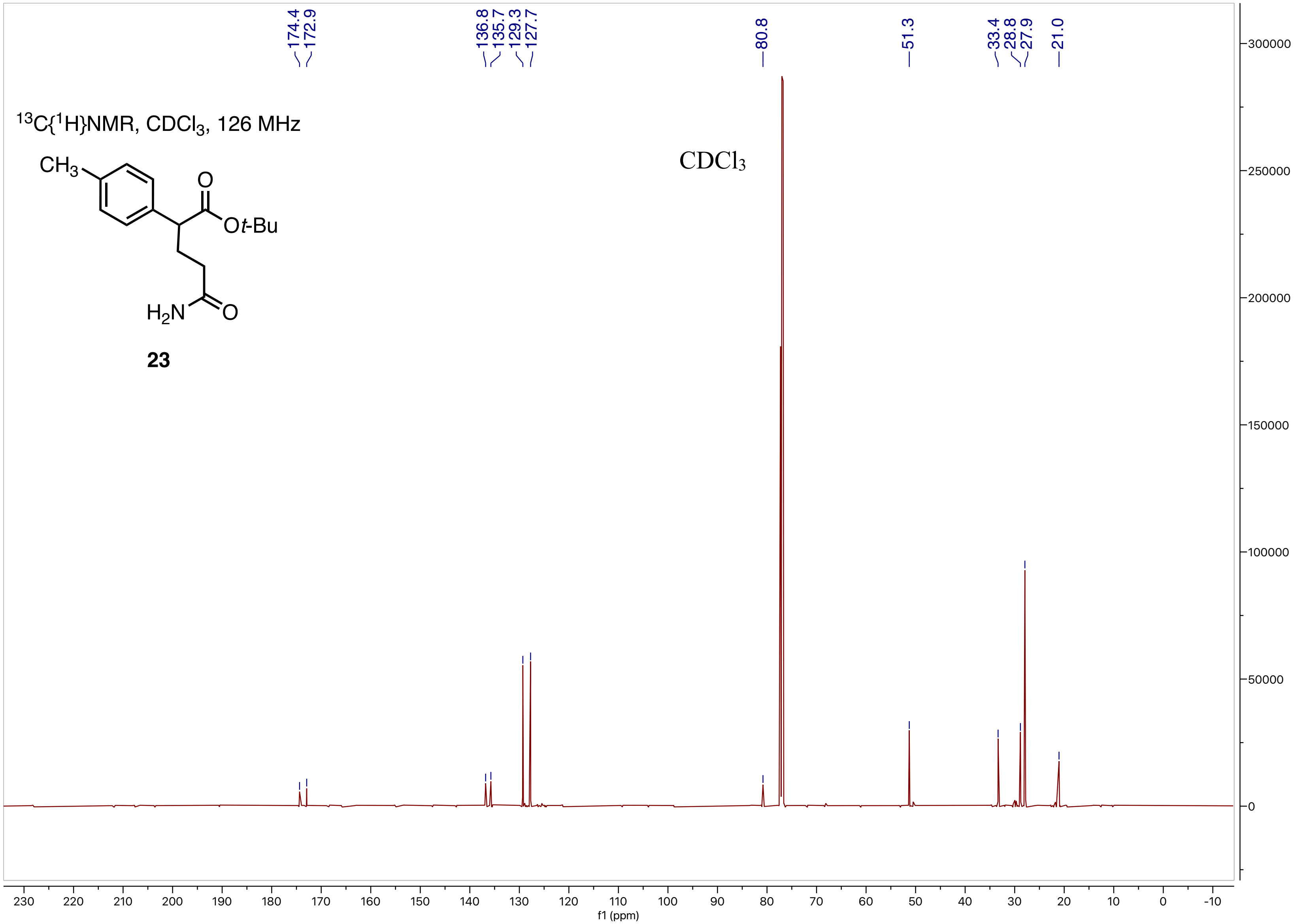
174.4
172.9
136.8
135.7
129.3
127.7
80.8
51.3
33.4
28.8
27.9
21.0
13C{1H}NMR, CDCl3, 126 MHz
CH3
O
Ot-Bu
H2N
O
23
CDCl3
f1 (ppm)

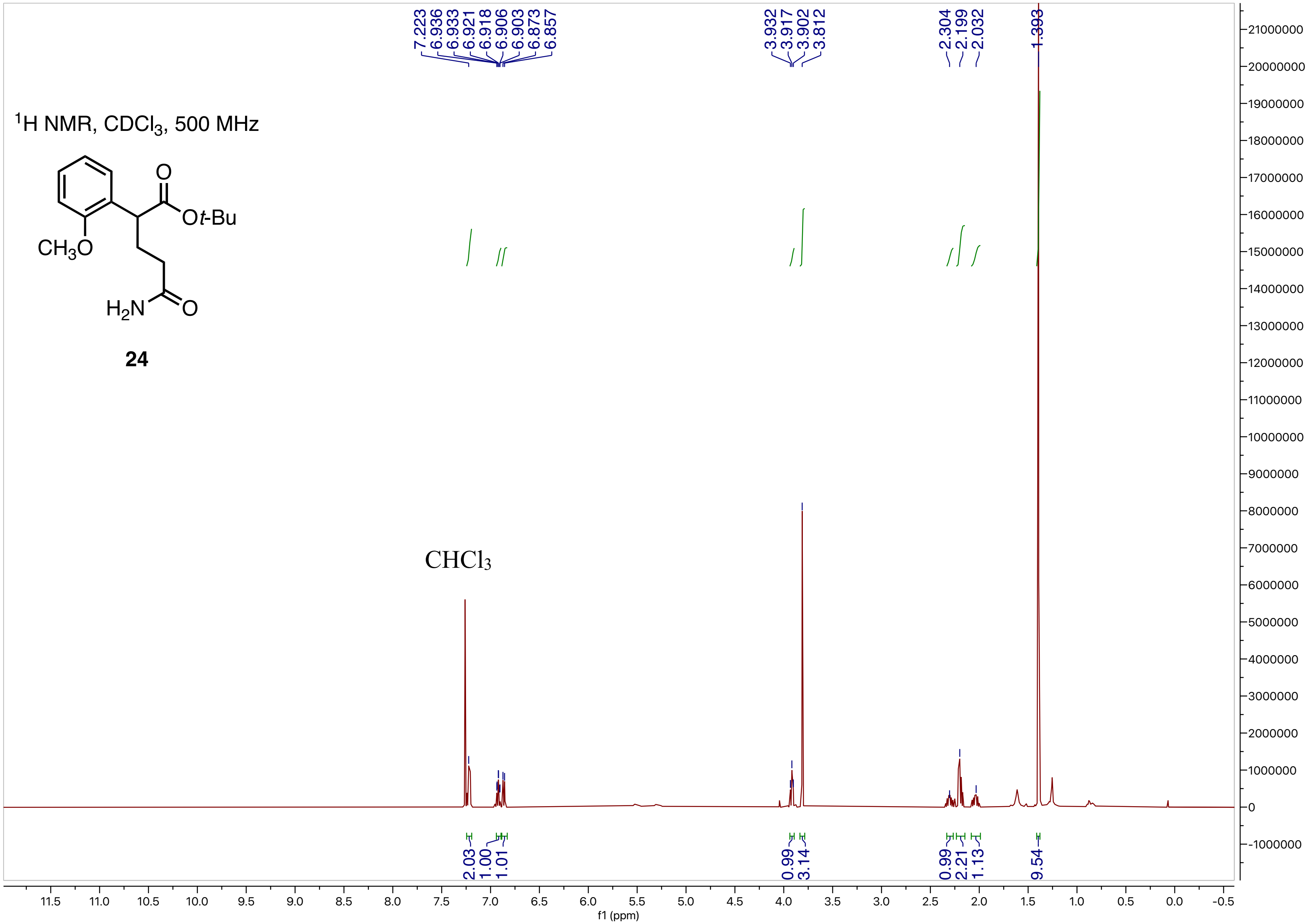
1H NMR, CDCl3, 500 MHz
OMe
CH3O
Ot-Bu
H2N
24
CHCl3
7.223
6.936
6.933
6.921
6.918
6.906
6.903
6.873
6.857
3.932
3.917
3.902
3.812
2.304
2.199
2.032
1.393
2.03
1.00
1.01
0.99
3.14
0.99
2.21
1.13
9.54
f1 (ppm)

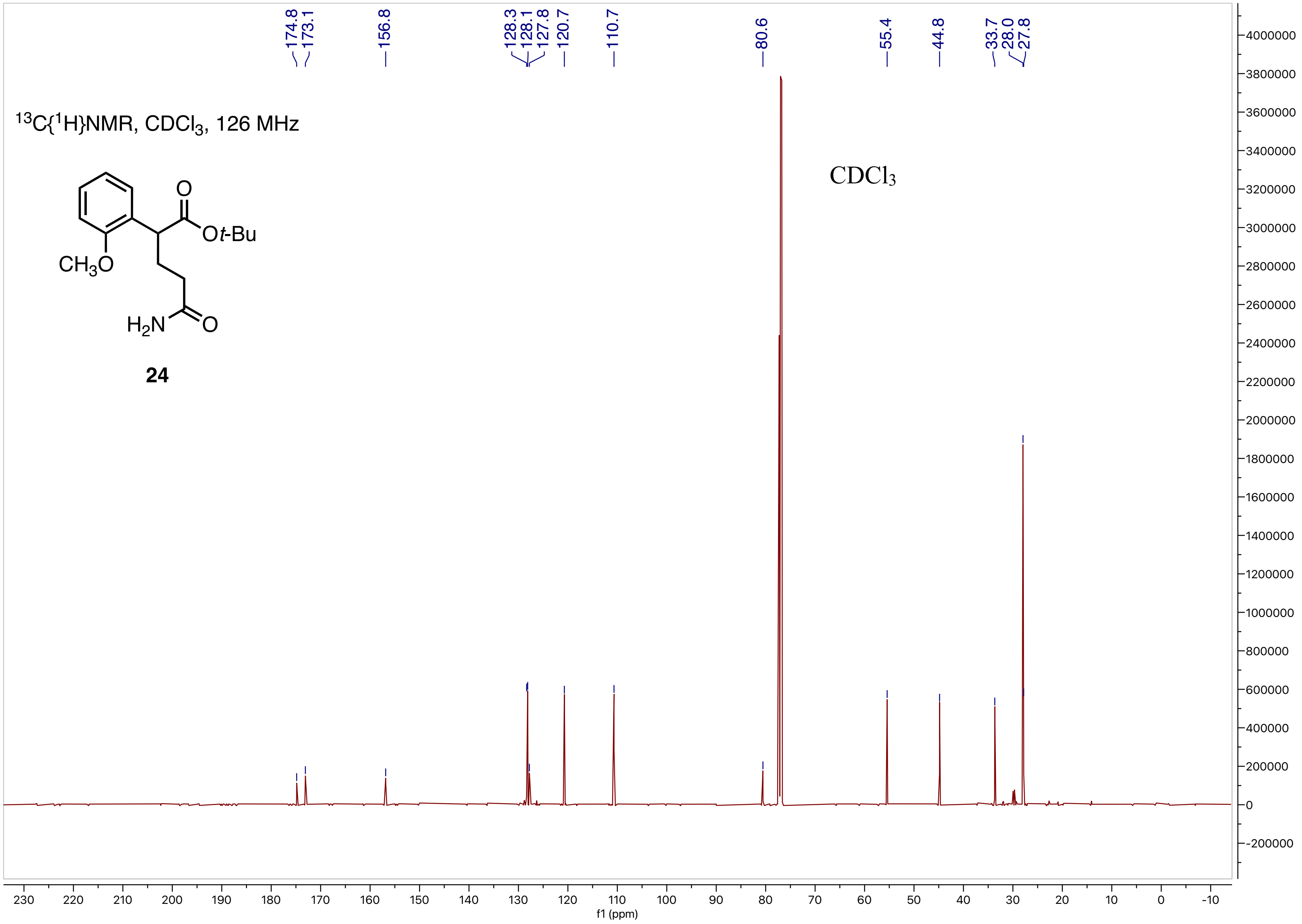

174.8
173.1
156.8
128.3
128.1
127.8
120.7
110.7
80.6
55.4
44.8
33.7
28.0
27.8
$^{13}C\{^1H\}$NMR, $CDCl_3$, 126 MHz
O
O*t*-Bu
CH_3O
H_2N
O
$CDCl_3$
f1 (ppm)

24

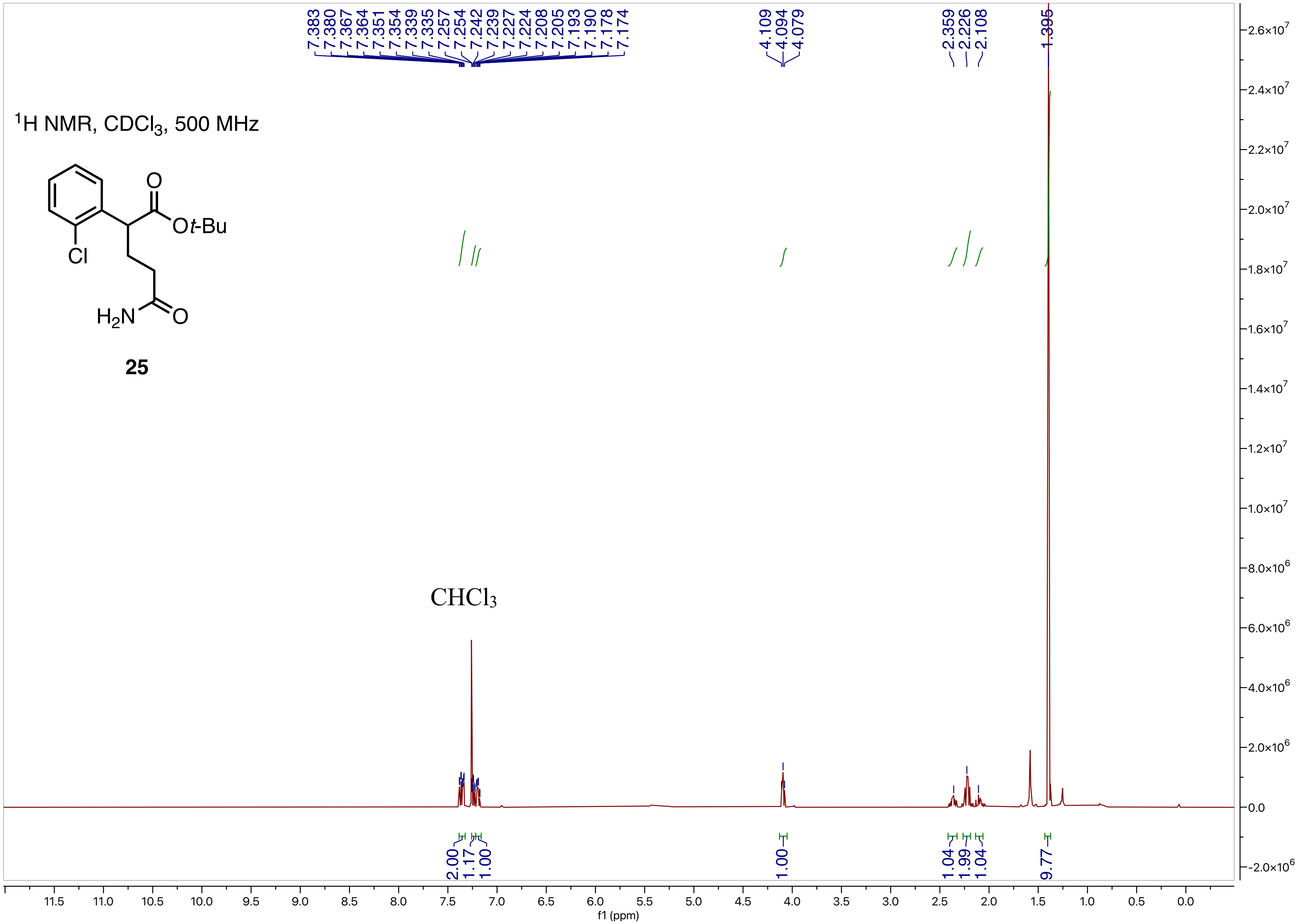
1H NMR, CDCl3, 500 MHz
O
Ot-Bu
Cl
H2N
O
25
CHCl3
7.383
7.380
7.367
7.364
7.351
7.354
7.339
7.335
7.257
7.254
7.242
7.239
7.227
7.224
7.208
7.205
7.193
7.190
7.178
7.174
4.109
4.094
4.079
2.359
2.226
2.108
1.395
2.00
1.17
1.00
1.00
1.04
1.99
1.04
9.77
f1 (ppm)

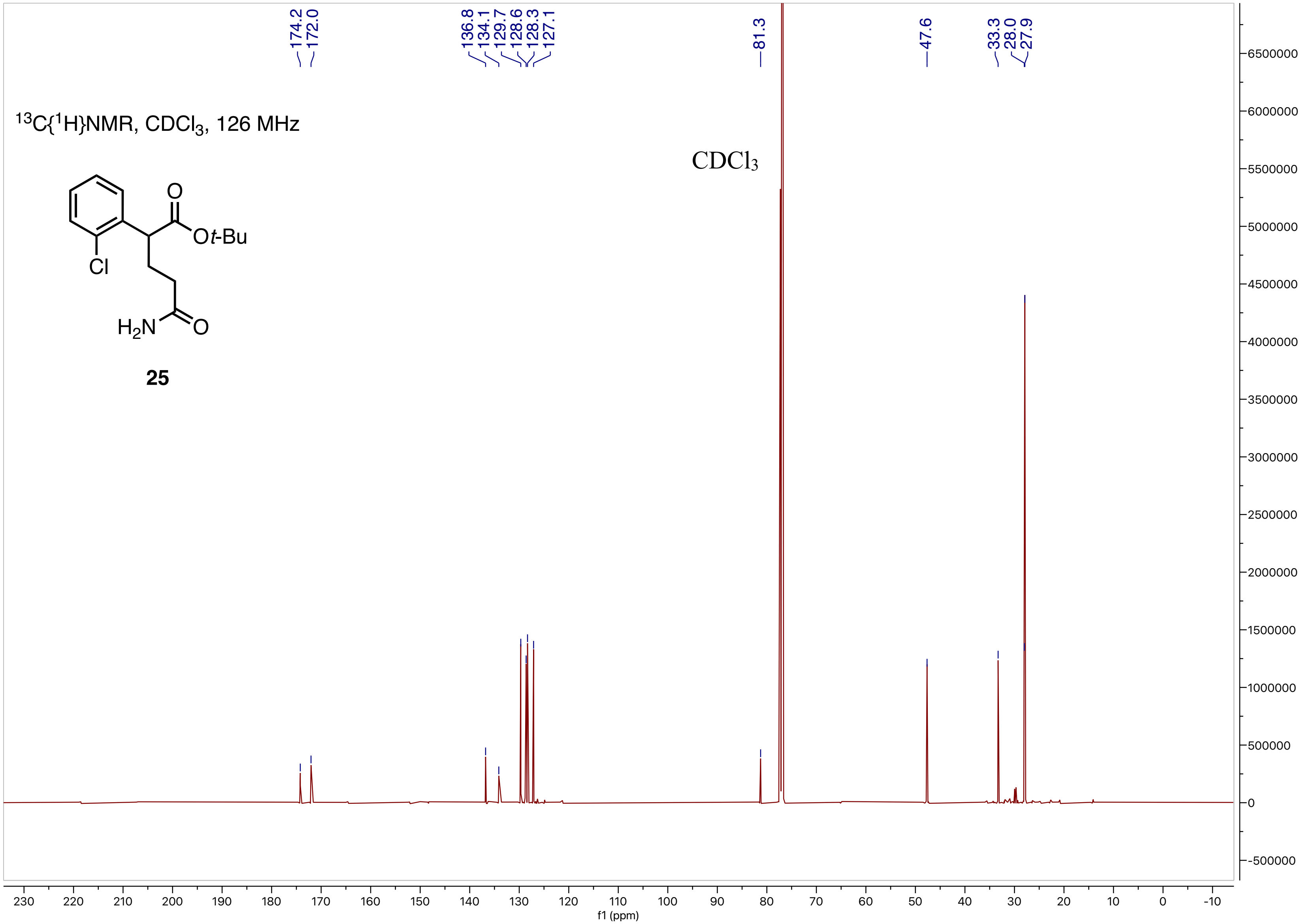

174.2
172.0
136.8
134.1
129.7
128.6
128.3
127.1
81.3
47.6
33.3
28.0
27.9
$^{13}C\{^{1}H\}$NMR, $CDCl_3$, 126 MHz
O
Ot-Bu
Cl
H_2N
O
25
$CDCl_3$
f1 (ppm)

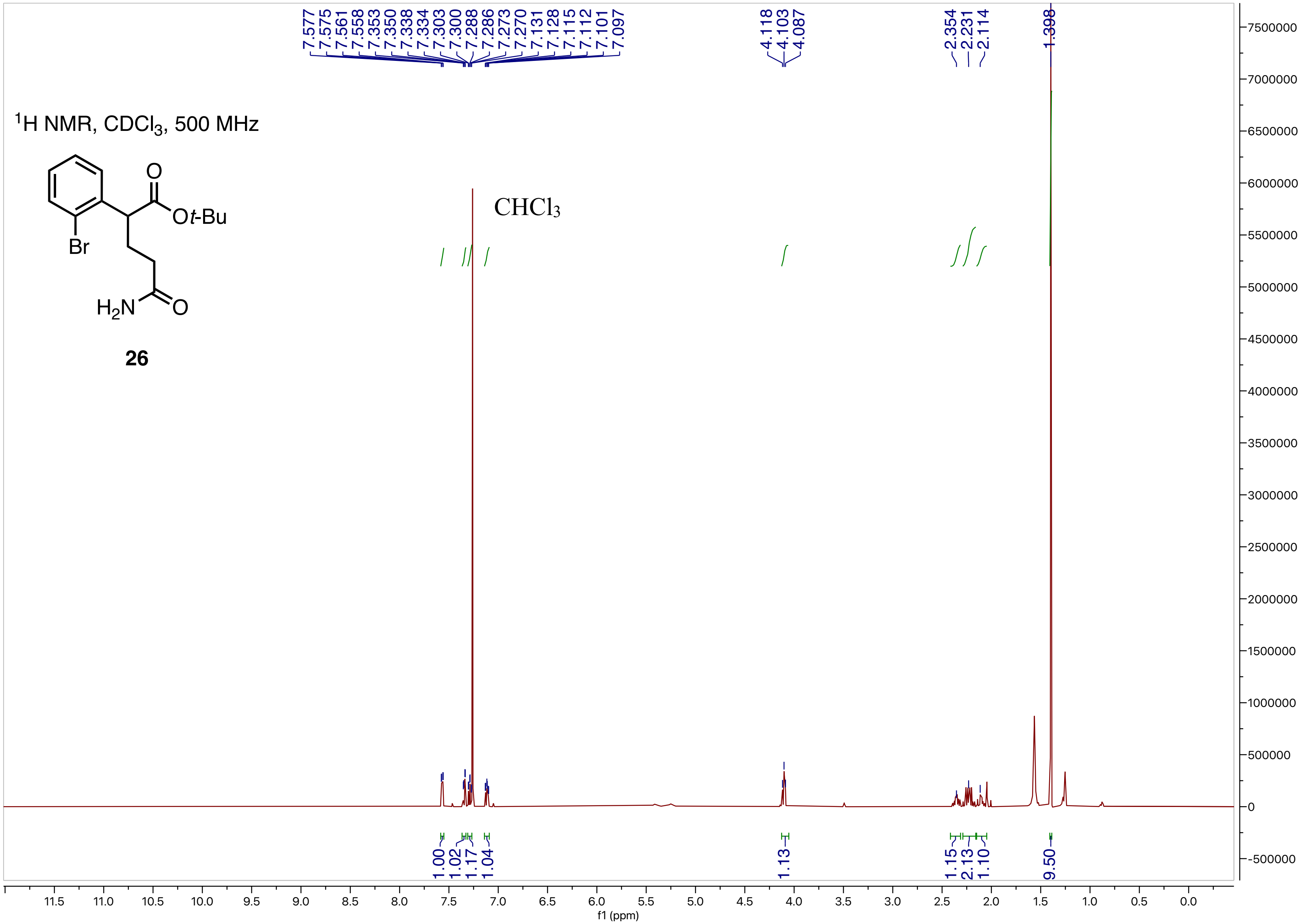

^1H NMR, $CDCl_3$, 500 MHz
O
Ot-Bu
Br
H_2N
O
26
CHCl3
7.577
7.575
7.561
7.558
7.353
7.350
7.338
7.334
7.303
7.300
7.288
7.286
7.273
7.270
7.131
7.128
7.115
7.112
7.101
7.097
4.118
4.103
4.087
2.354
2.231
2.114
1.398
1.00
1.02
1.17
1.04
1.13
1.15
2.13
1.10
9.50
f1 (ppm)

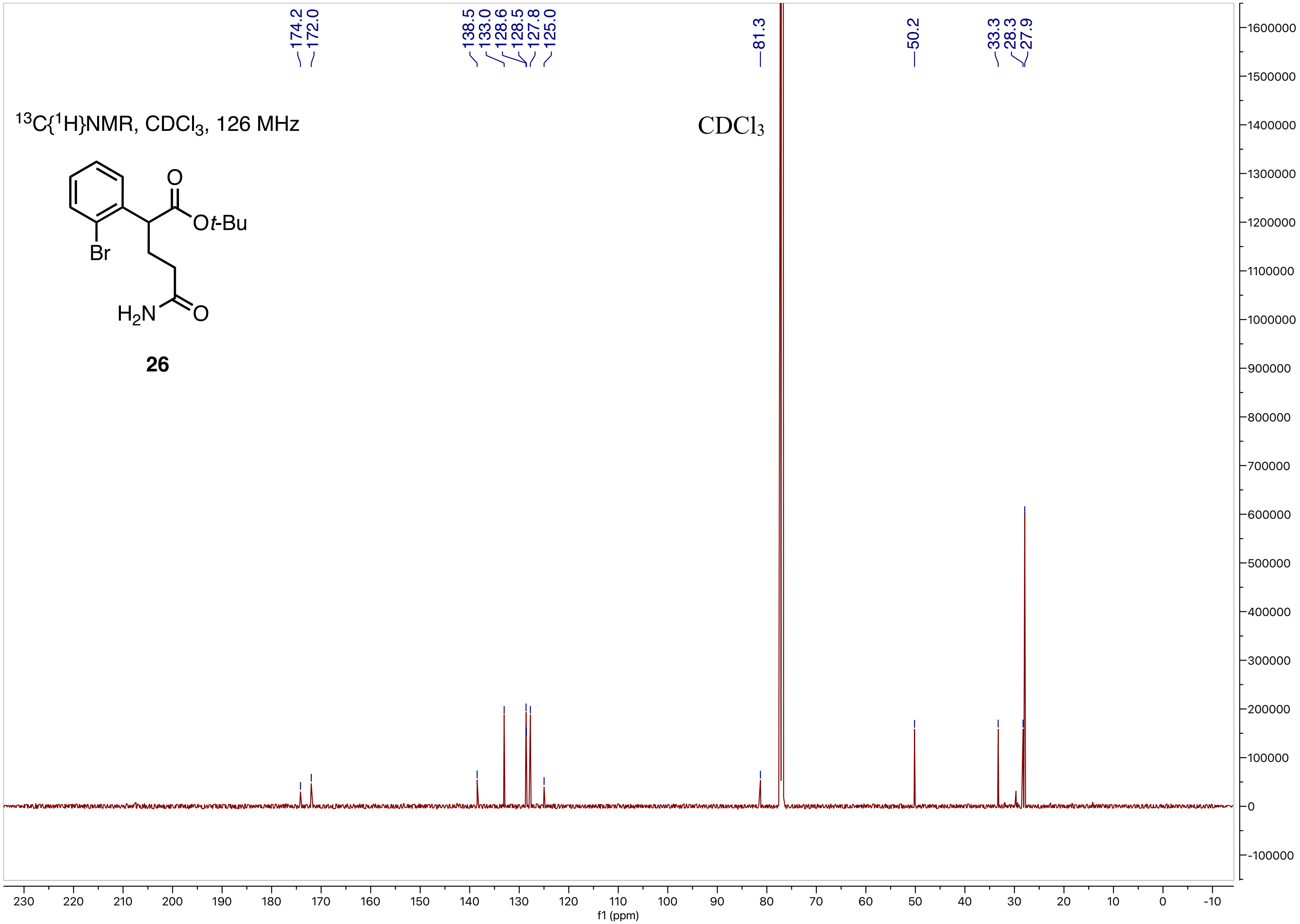
174.2
172.0
138.5
133.0
128.6
128.5
127.8
125.0
81.3
50.2
33.3
28.3
27.9
^{13}C{^{1}H}NMR, CDCl$_3$, 126 MHz
CDCl$_3$
O
O*t*-Bu
Br
H_2N
O
26
f1 (ppm)

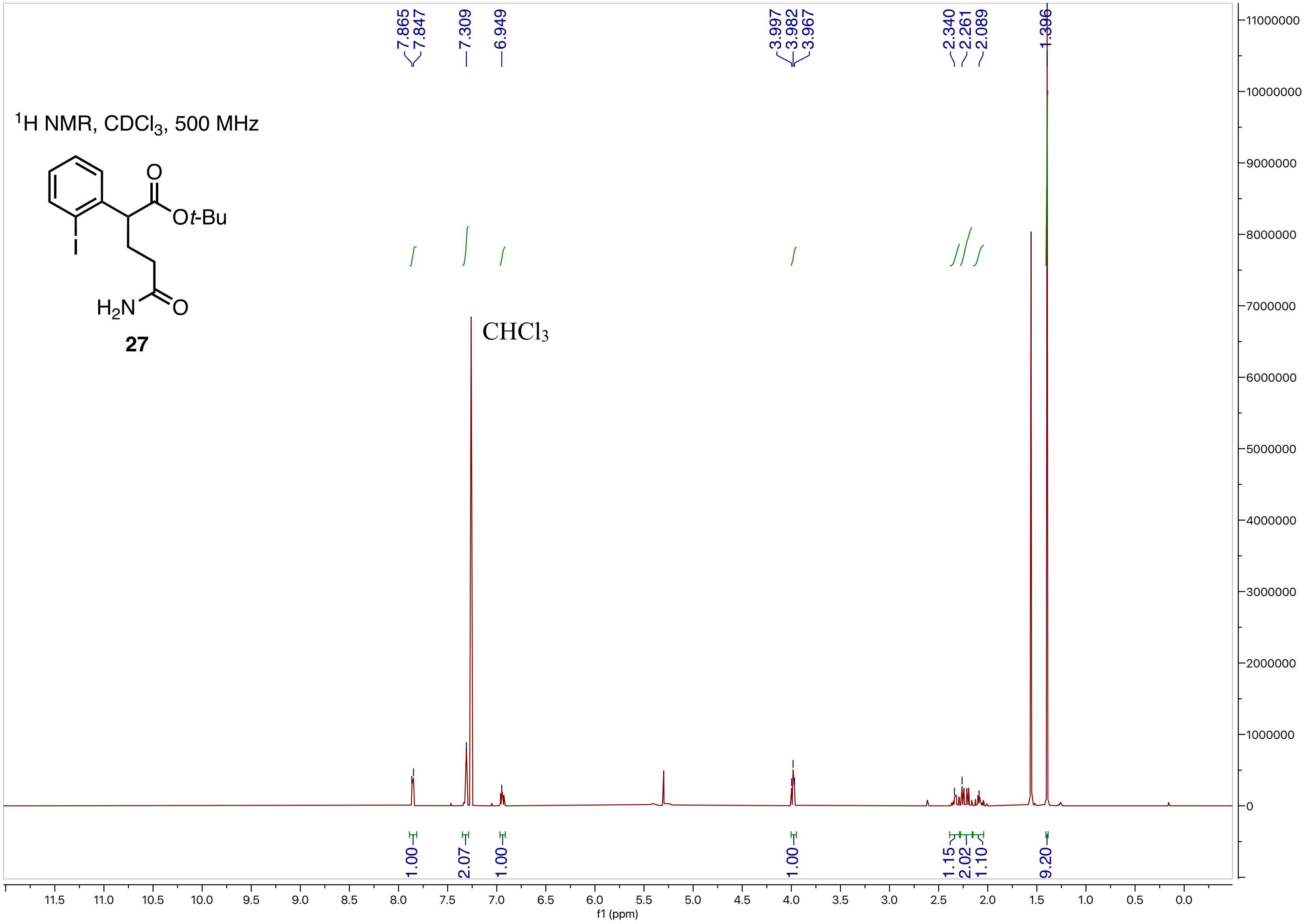

1H NMR, CDCl3, 500 MHz
Ot-Bu
H2N
27
CHCl3
7.865
7.847
7.309
6.949
3.997
3.982
3.967
2.340
2.261
2.089
1.396
1.00
2.07
1.00
1.00
1.15
2.02
1.10
9.20
f1 (ppm)

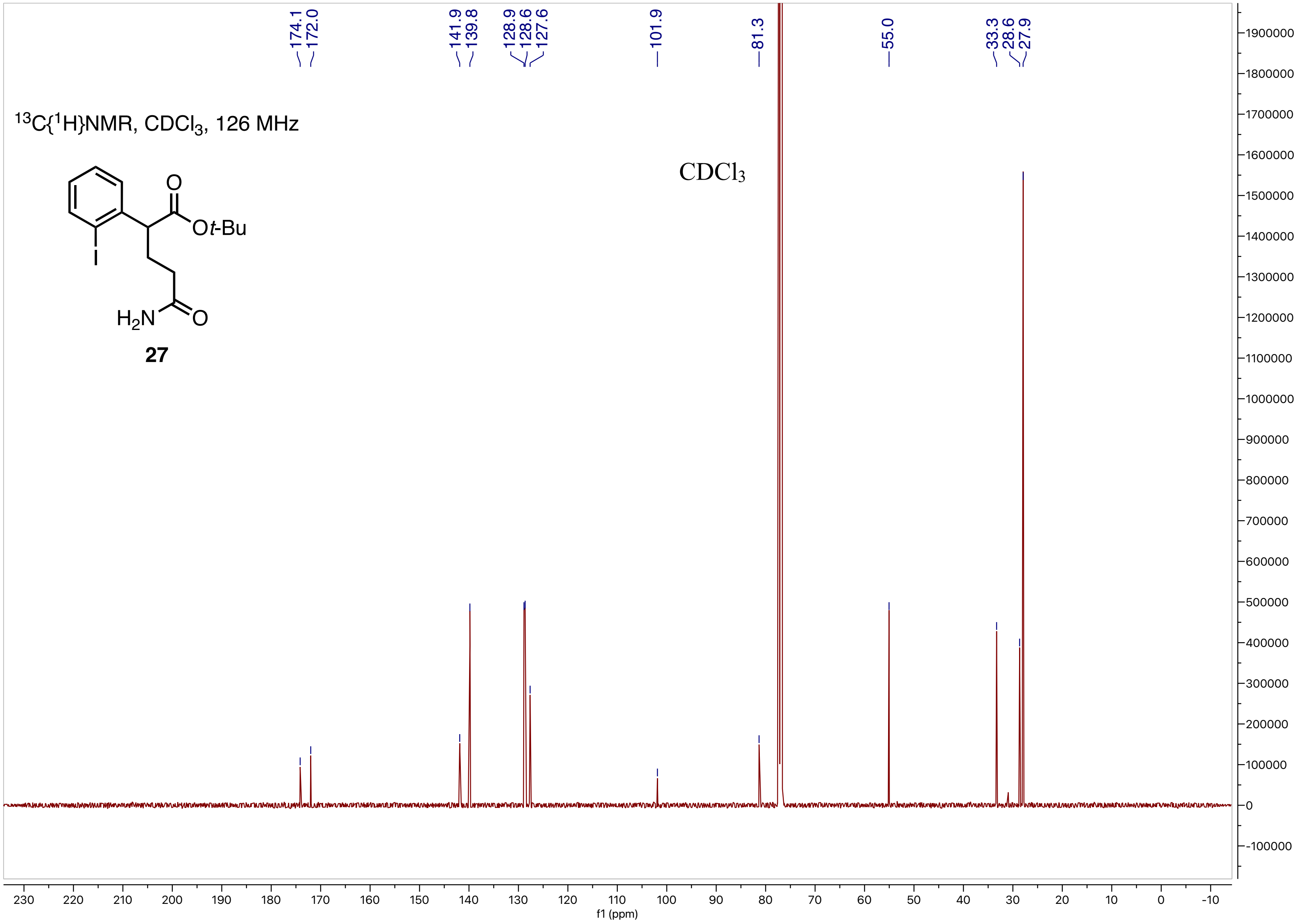

174.1
172.0
141.9
139.8
128.9
128.6
127.6
101.9
81.3
55.0
33.3
28.6
27.9
13C{1H}NMR, CDCl3, 126 MHz
O
Ot-Bu
I
H2N
O
27
CDCl3
f1 (ppm)

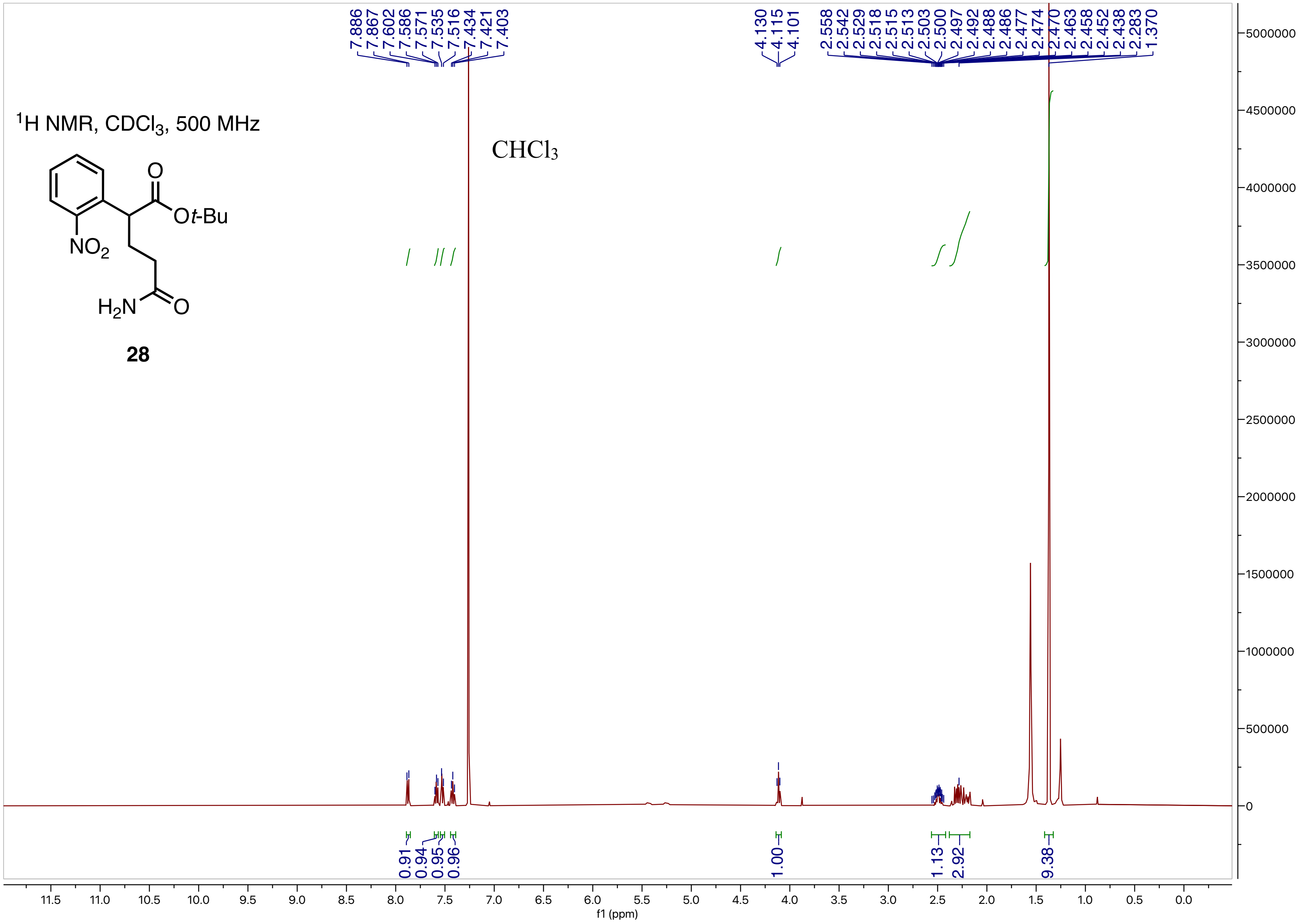

¹H NMR, CDCl₃, 500 MHz
NO₂
Ot-Bu
O
H₂N
O
28
CHCl₃
7.886
7.867
7.602
7.586
7.571
7.535
7.516
7.434
7.421
7.403
4.130
4.115
4.101
2.558
2.542
2.529
2.518
2.515
2.513
2.503
2.500
2.497
2.492
2.488
2.486
2.477
2.474
2.470
2.463
2.458
2.452
2.438
2.283
1.370
0.91
0.94
0.95
0.96
1.00
1.13
2.92
9.38
f1 (ppm)

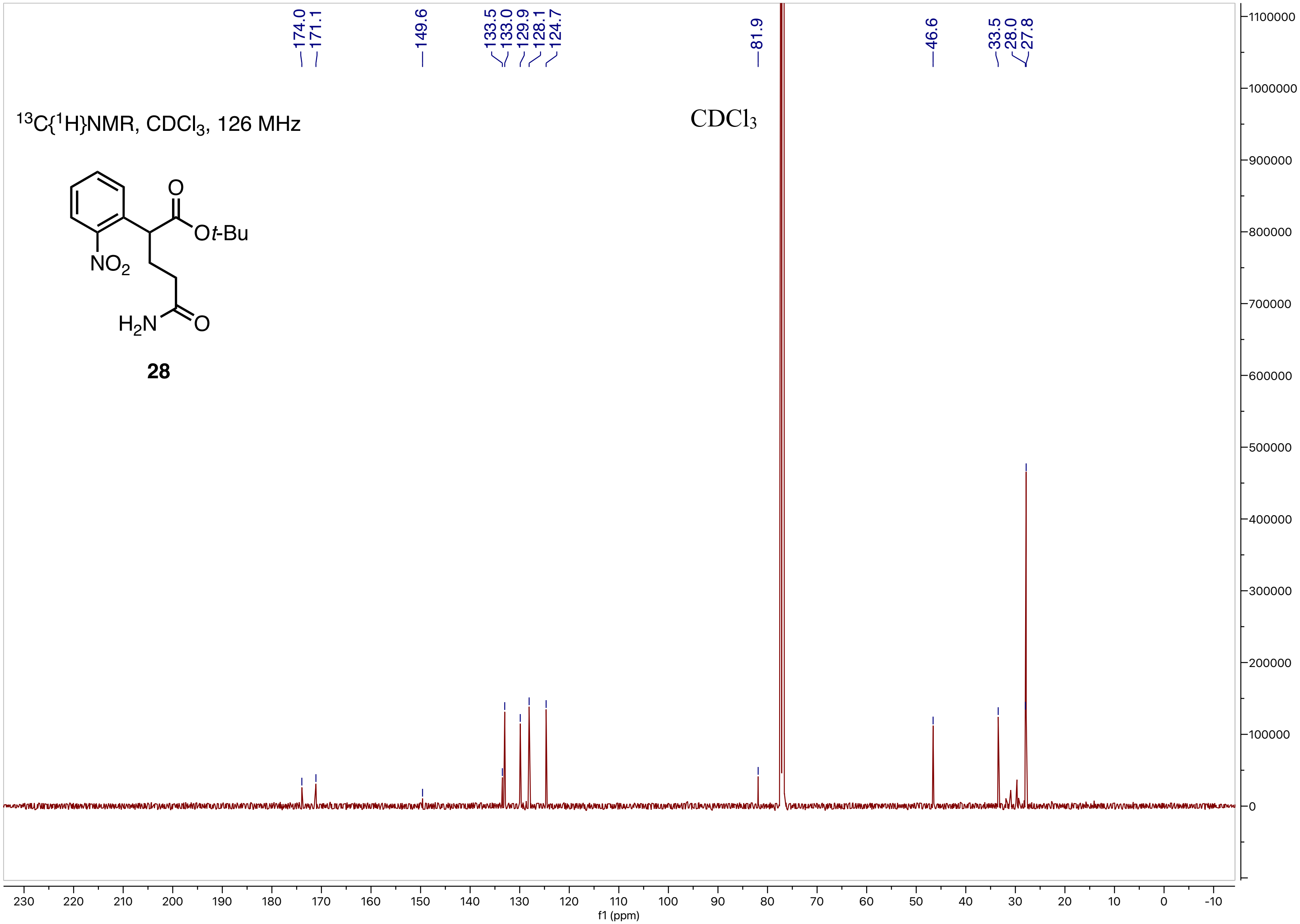

$^{13}C\{^1H\}$NMR, $CDCl_3$, 126 MHz
NO2
O
Ot-Bu
H2N
O
28
CDCl3
174.0
171.1
149.6
133.5
133.0
129.9
128.1
124.7
81.9
46.6
33.5
28.0
27.8
f1 (ppm)

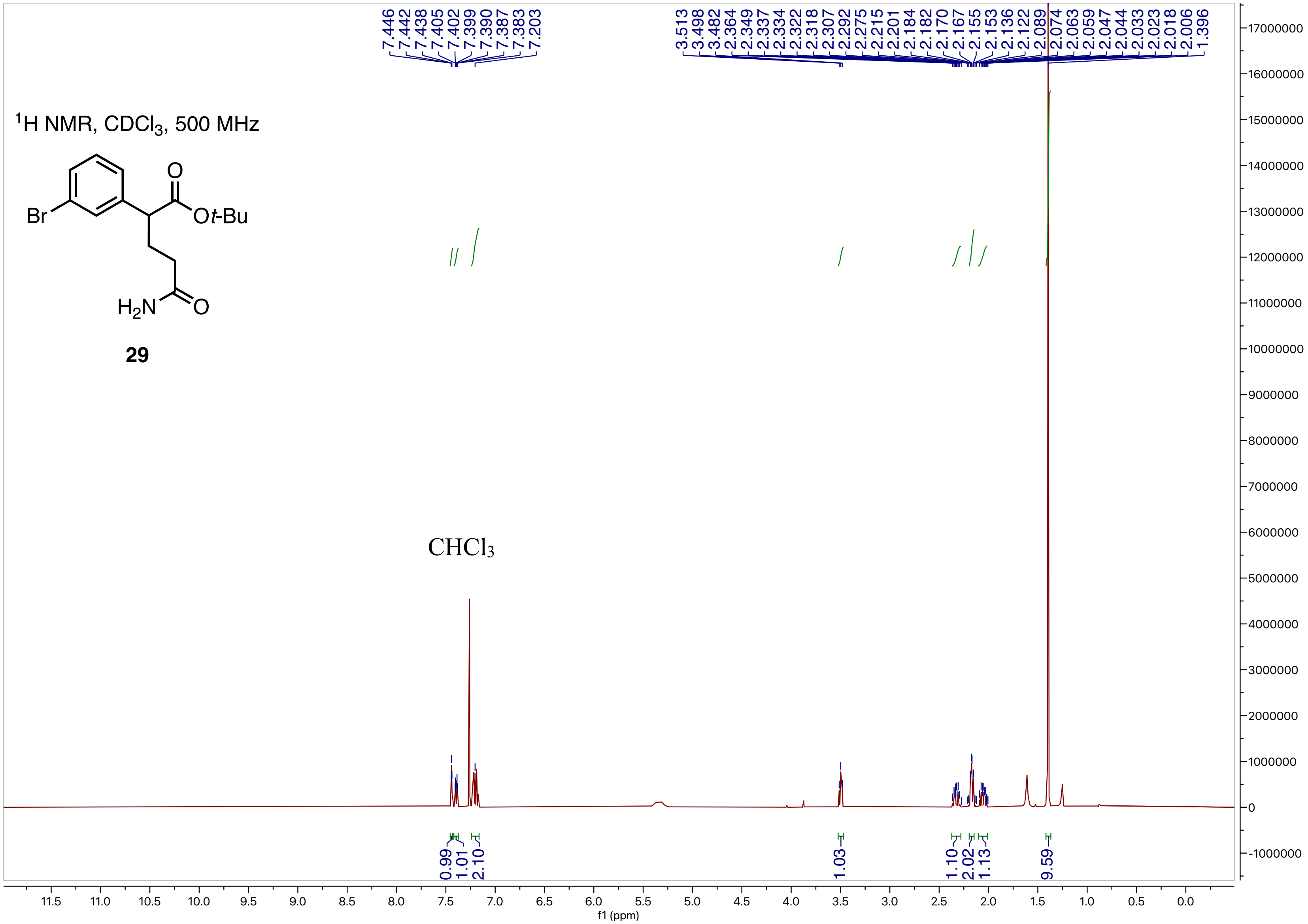
1H NMR, CDCl3, 500 MHz
29
CHCl3
7.446 7.442 7.438 7.405 7.402 7.399 7.390 7.387 7.383 7.203
3.513 3.498 3.482 2.364 2.349 2.337 2.334 2.322 2.318 2.307 2.292 2.275 2.215 2.201 2.184 2.182 2.170 2.167 2.155 2.153 2.136 2.122 2.089 2.074 2.063 2.059 2.047 2.044 2.033 2.023 2.018 2.006 1.396
0.99 1.01 2.10 1.03 1.10 2.02 1.13 9.59
f1 (ppm)

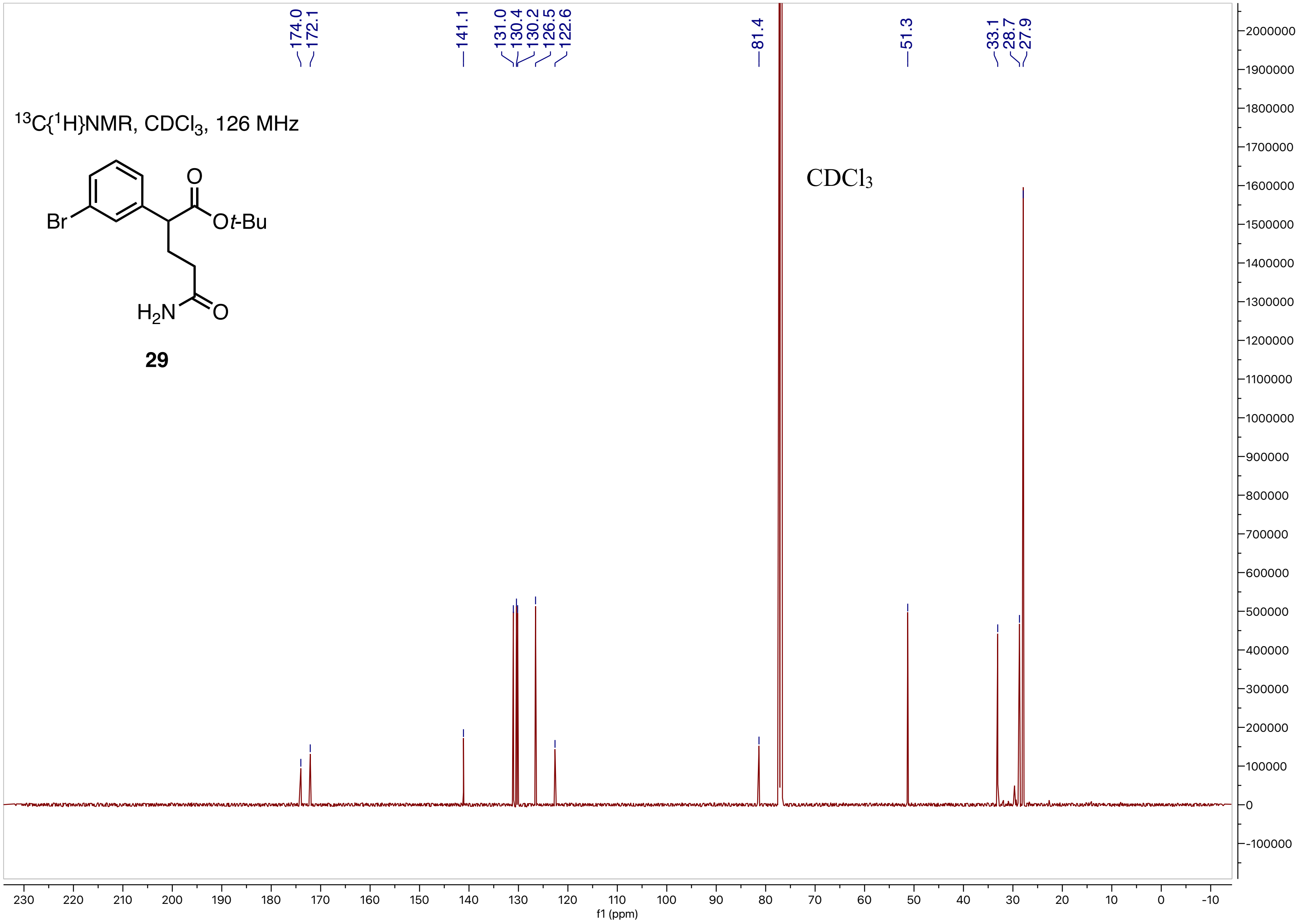

174.0
172.1
141.1
131.0
130.4
130.2
126.5
122.6
81.4
51.3
33.1
28.7
27.9
$^{13}C\{^{1}H\}$NMR, $CDCl_3$, 126 MHz
Br
O
O*t*-Bu
H_2N
O
29
CDCl3
f1 (ppm)

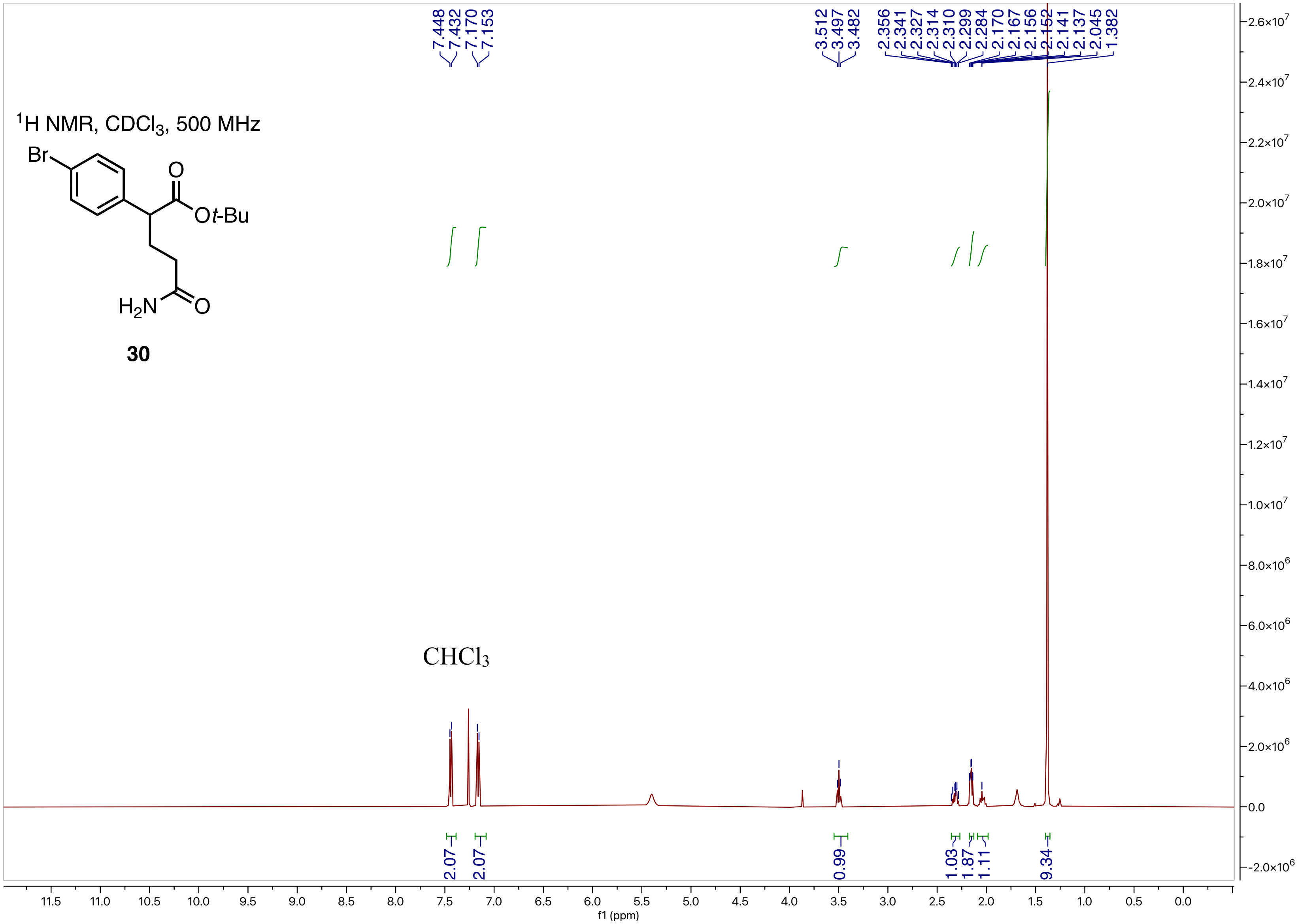

1H NMR, CDCl3, 500 MHz
Br
O
Ot-Bu
H2N
O
30
CHCl3
7.448
7.432
7.170
7.153
3.512
3.497
3.482
2.356
2.341
2.327
2.314
2.310
2.299
2.284
2.170
2.167
2.156
2.152
2.141
2.137
2.045
1.382
2.07
2.07
0.99
1.03
1.87
1.11
9.34
f1 (ppm)

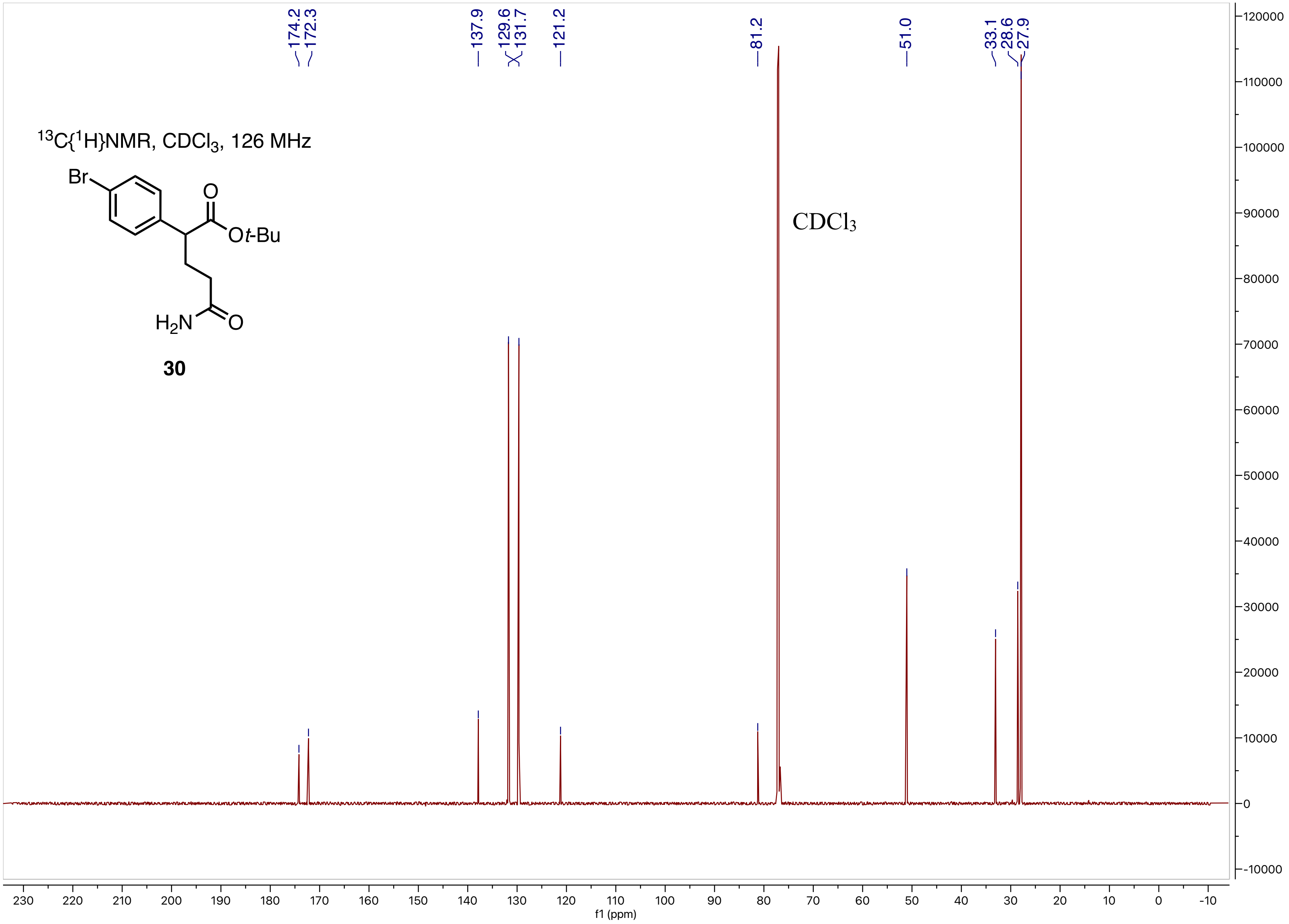

174.2
172.3
137.9
129.6
131.7
121.2
81.2
51.0
33.1
28.6
27.9
$^{13}C\{^1H\}$NMR, $CDCl_3$, 126 MHz
Br
O
O*t*-Bu
H_2N
O
30
$CDCl_3$
120000
110000
100000
90000
80000
70000
60000
50000
40000
30000
20000
10000
0
-10000
230
220
210
200
190
180
170
160
150
140
130
120
110
100
90
80
70
60
50
40
30
20
10
0
-10
f1 (ppm)

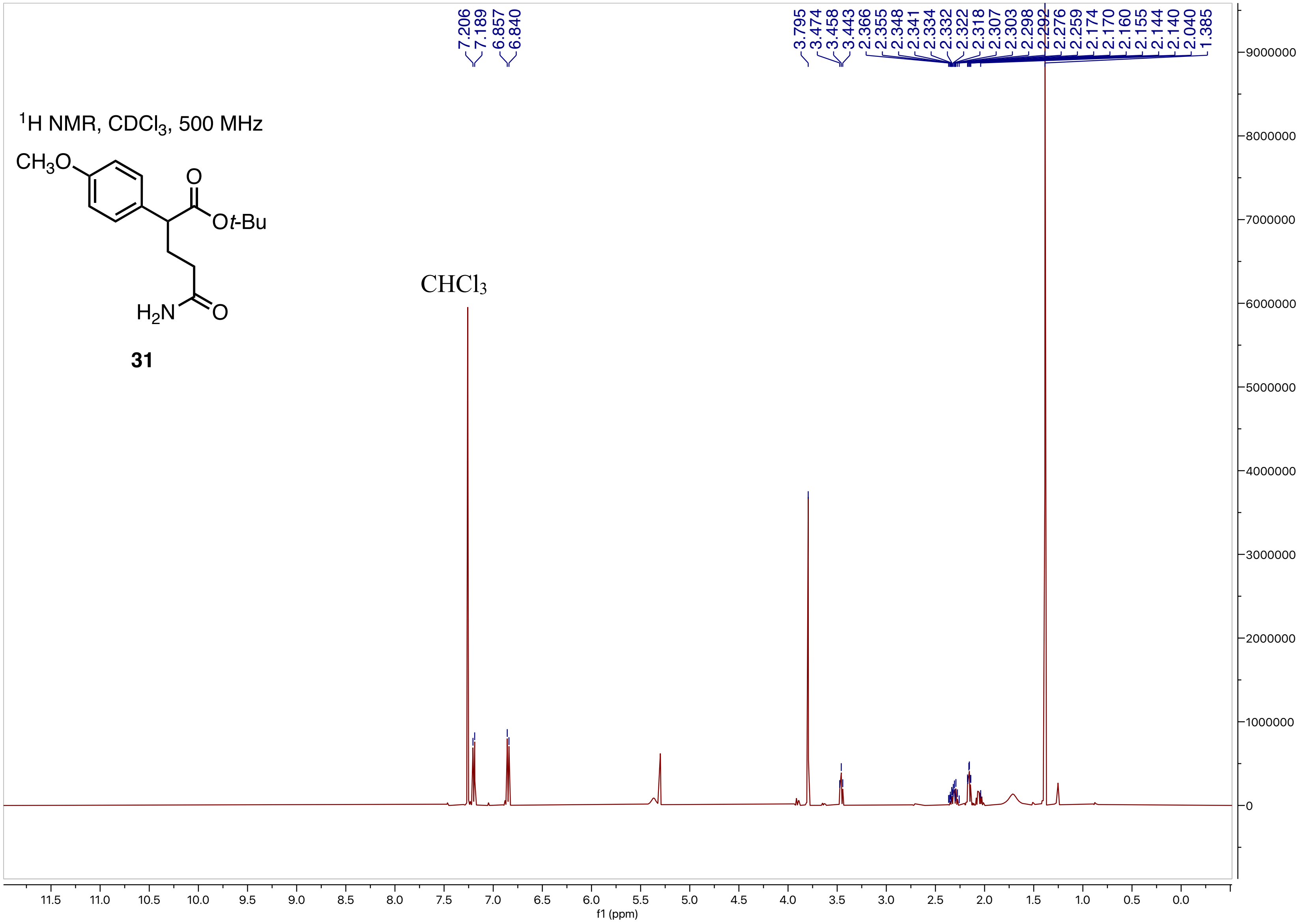
1H NMR, CDCl3, 500 MHz
CH3O
O
Ot-Bu
H2N
O
31
CHCl3
7.206
7.189
6.857
6.840
3.795
3.474
3.458
3.443
2.366
2.355
2.348
2.341
2.334
2.332
2.322
2.318
2.307
2.303
2.298
2.292
2.276
2.259
2.174
2.170
2.160
2.155
2.144
2.140
2.040
1.385
f1 (ppm)

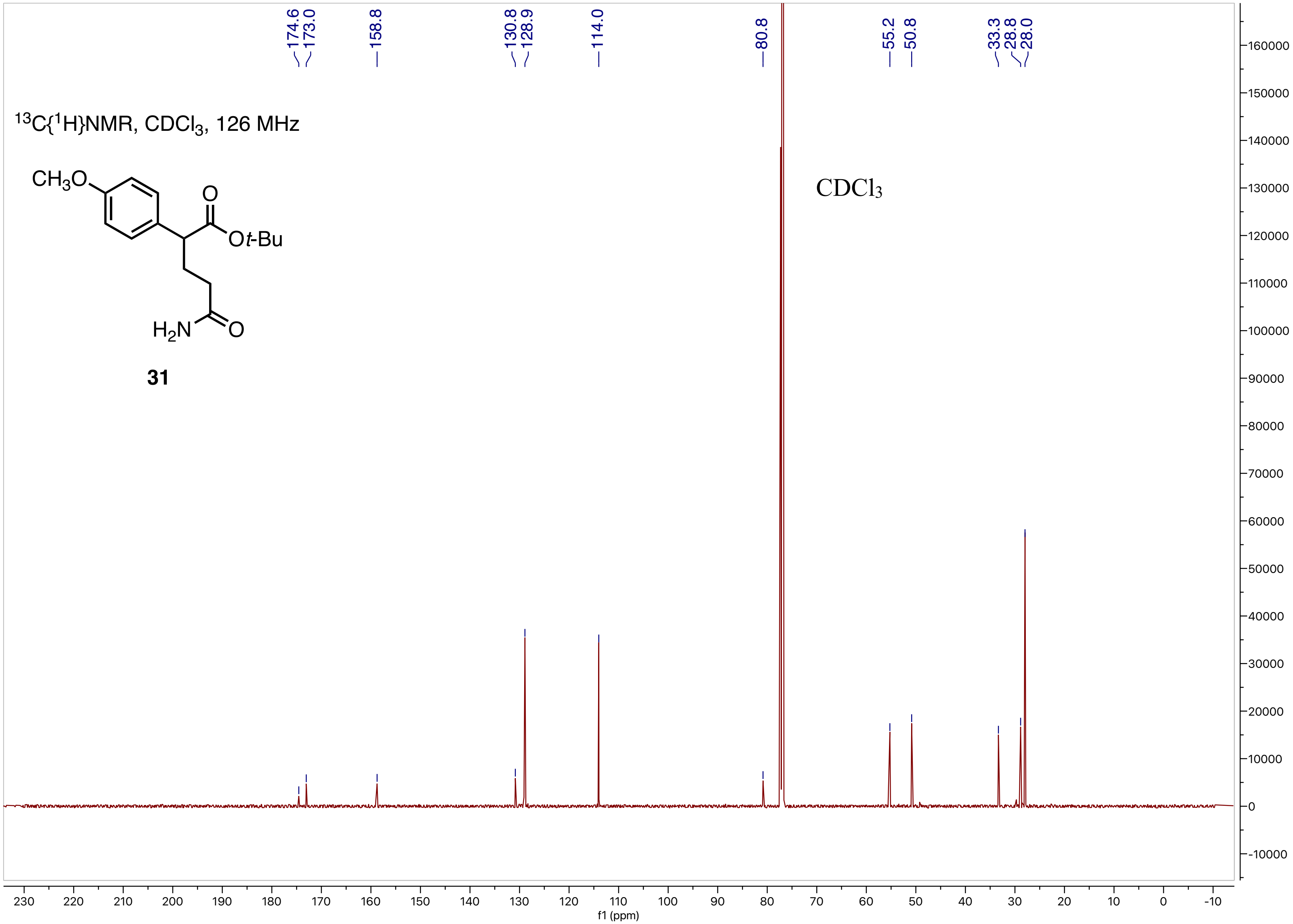

174.6
173.0
158.8
130.8
128.9
114.0
80.8
55.2
50.8
33.3
28.8
28.0
$^{13}C\{^1H\}$NMR, $CDCl_3$, 126 MHz
CH_3O
O
O*t*-Bu
H_2N
O
31
$CDCl_3$
f1 (ppm)

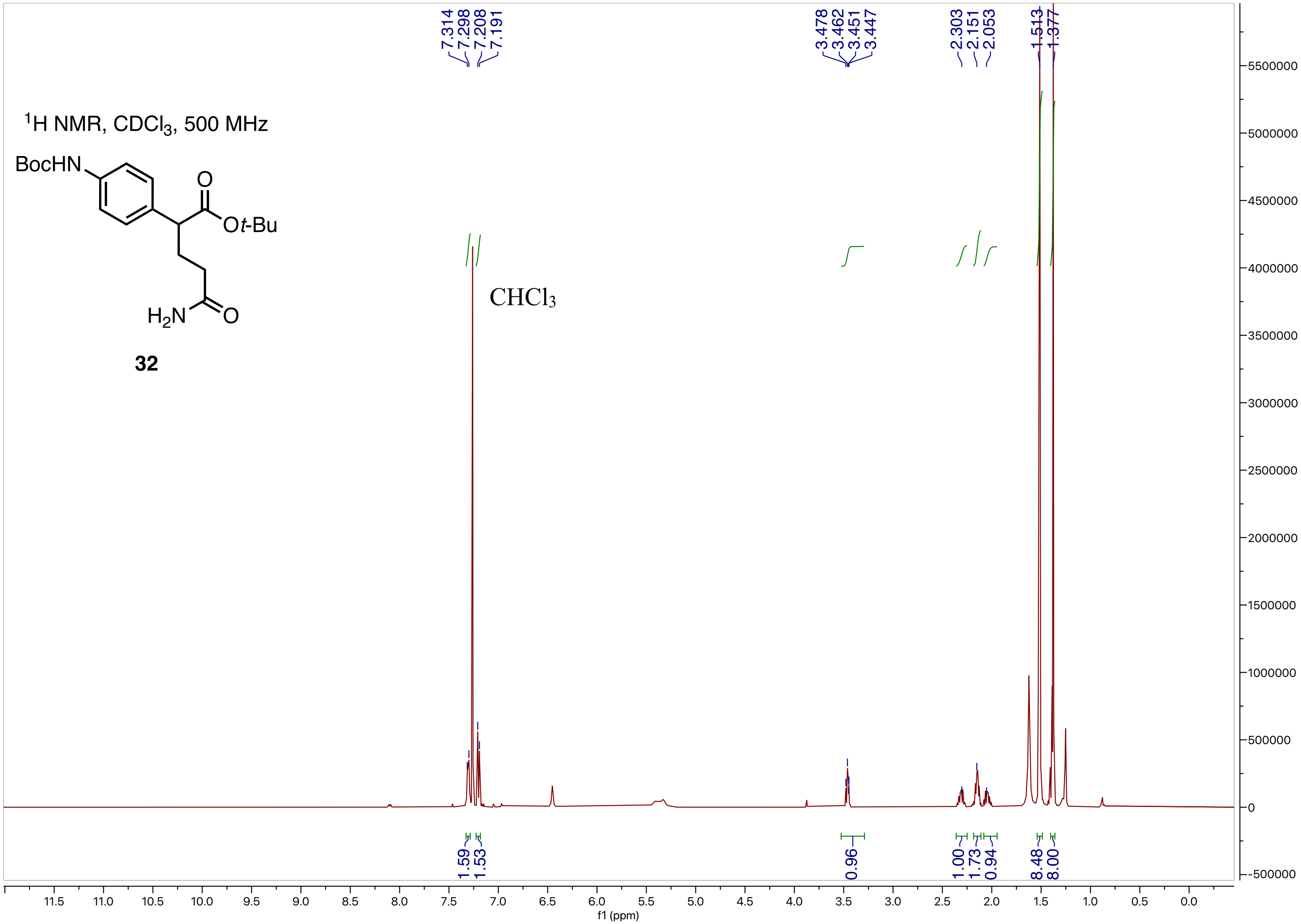

1H NMR, CDCl3, 500 MHz
BocHN
O
Ot-Bu
H2N
O
32
CHCl3
7.314
7.298
7.208
7.191
3.478
3.462
3.451
3.447
2.303
2.151
2.053
1.513
1.377
1.59
1.53
0.96
1.00
1.73
0.94
8.48
8.00
f1 (ppm)

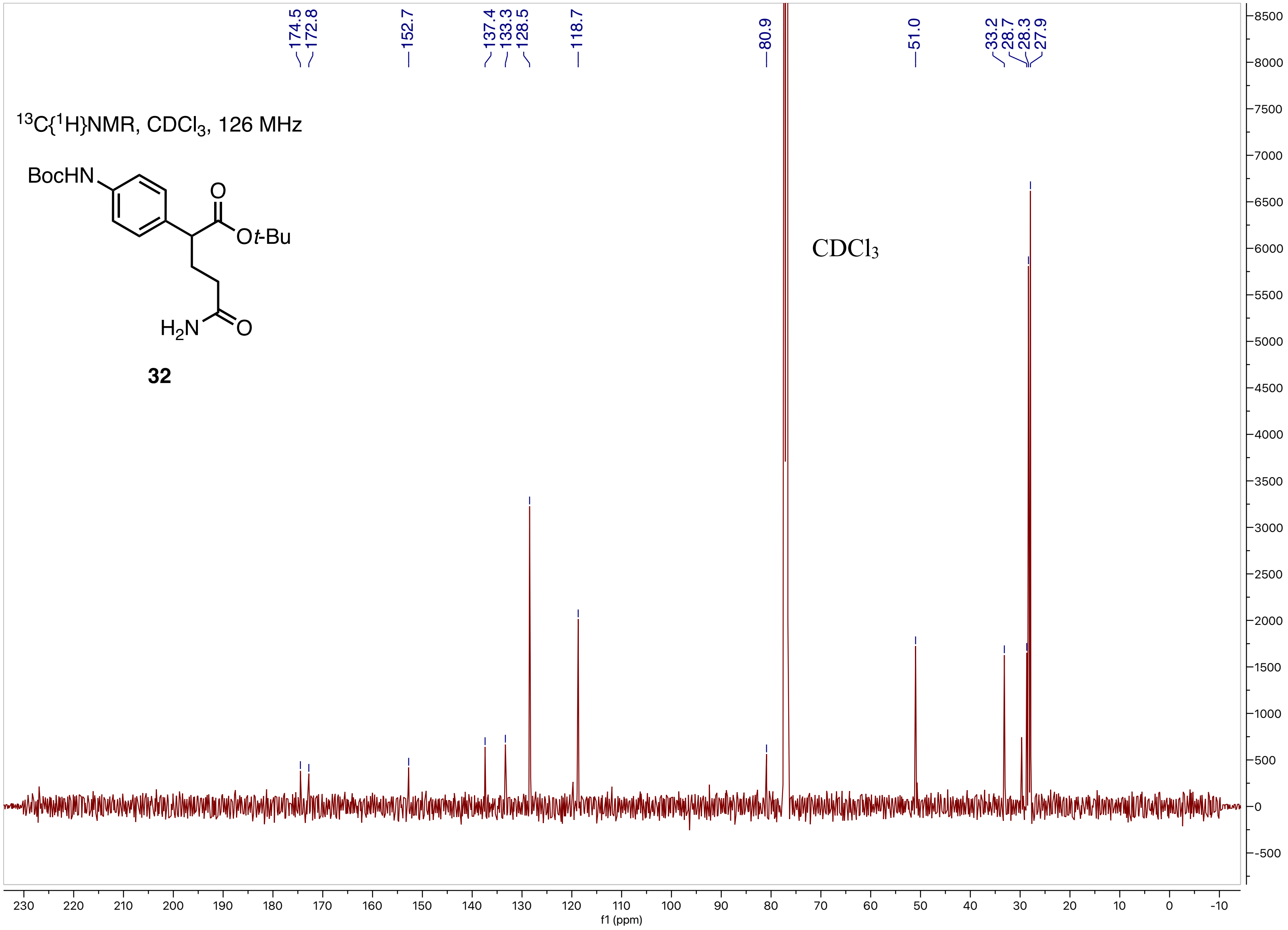

$^{13}C\{^1H\}$NMR, $CDCl_3$, 126 MHz
BocHN
O
Ot-Bu
H_2N
O
174.5
172.8
152.7
137.4
133.3
128.5
118.7
80.9
51.0
33.2
28.7
28.3
27.9
$CDCl_3$
f1 (ppm)

32

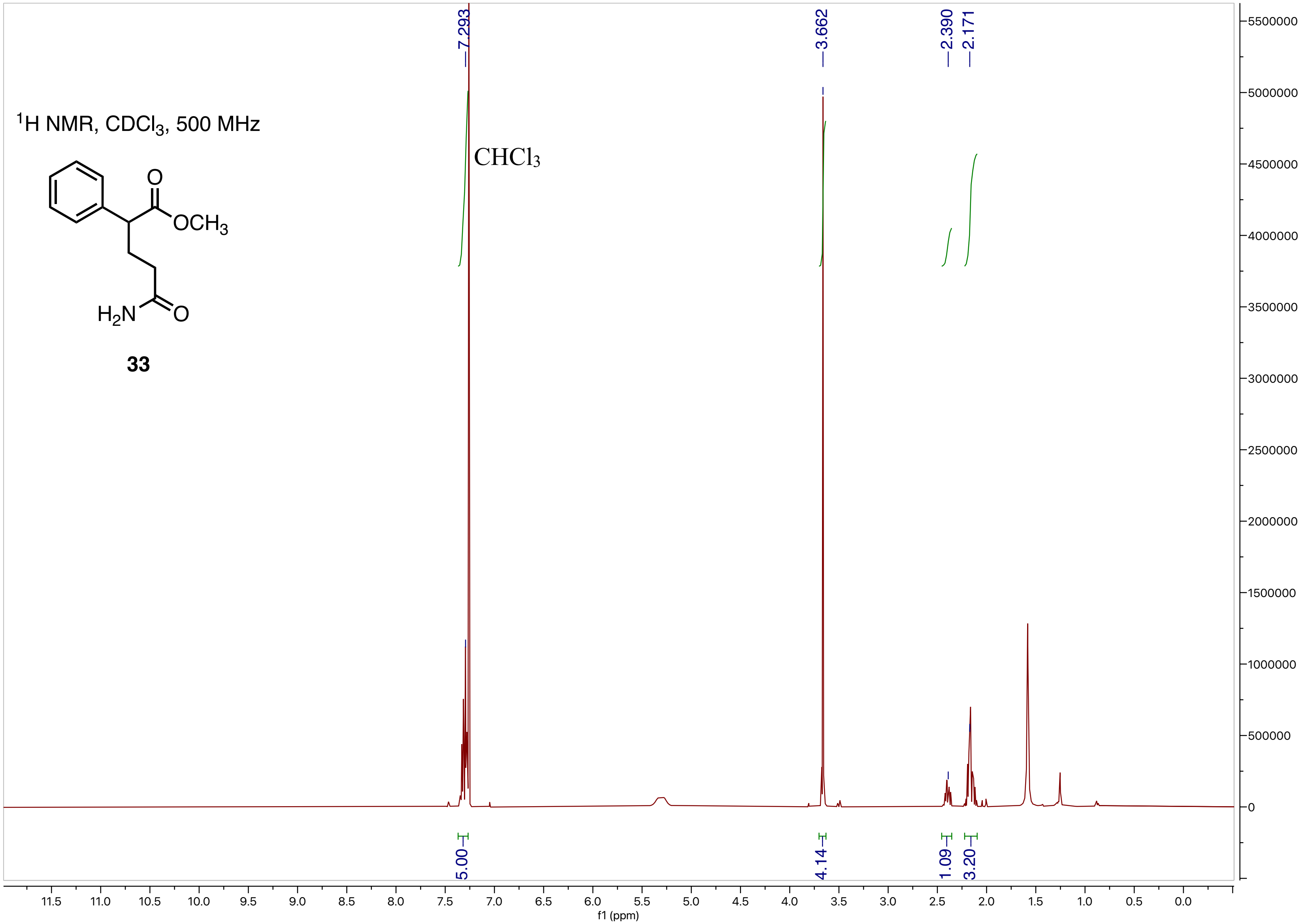

^1H NMR, $CDCl_3$, 500 MHz
O
OCH3
H2N
O
33
CHCl3
7.293
3.662
2.390
2.171
5.00
4.14
1.09
3.20
f1 (ppm)

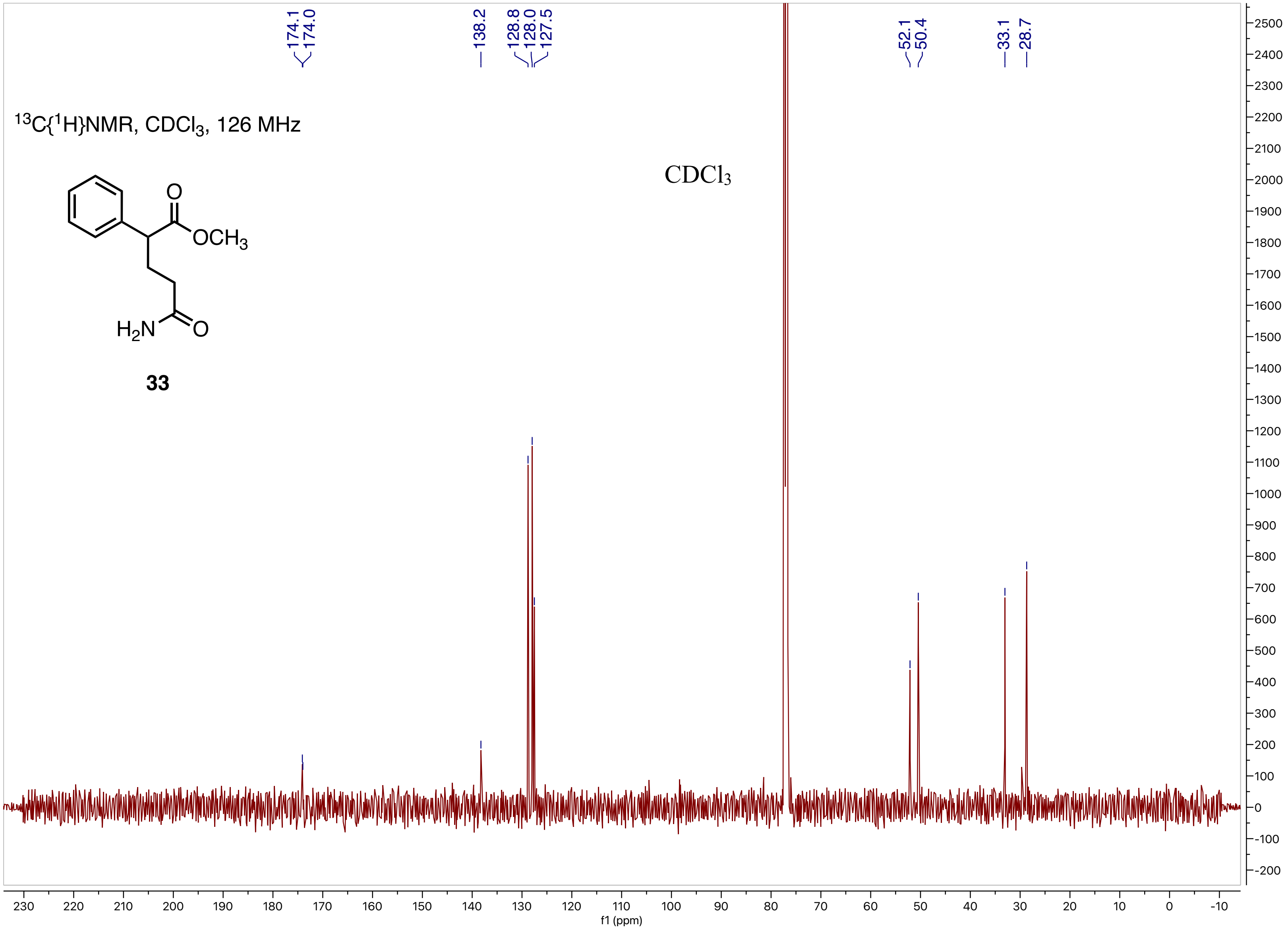

174.1
174.0
138.2
128.8
128.0
127.5
52.1
50.4
33.1
28.7
$^{13}C\{^1H\}$NMR, $CDCl_3$, 126 MHz
O
OCH_3
H_2N
O
33
$CDCl_3$
f1 (ppm)

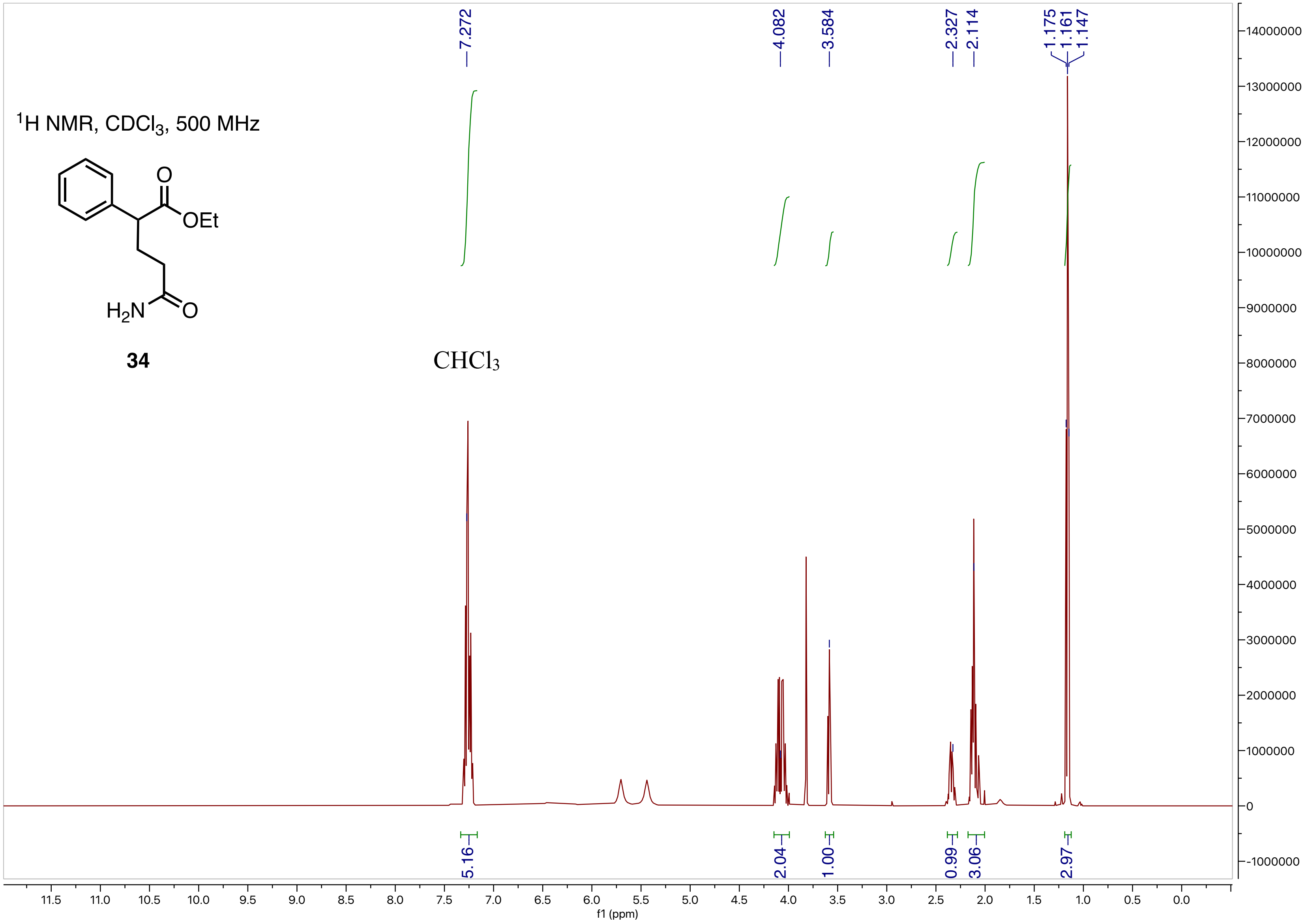

1H NMR, CDCl3, 500 MHz
34
CHCl3
7.272
4.082
3.584
2.327
2.114
1.175
1.161
1.147
5.16
2.04
1.00
0.99
3.06
2.97
f1 (ppm)

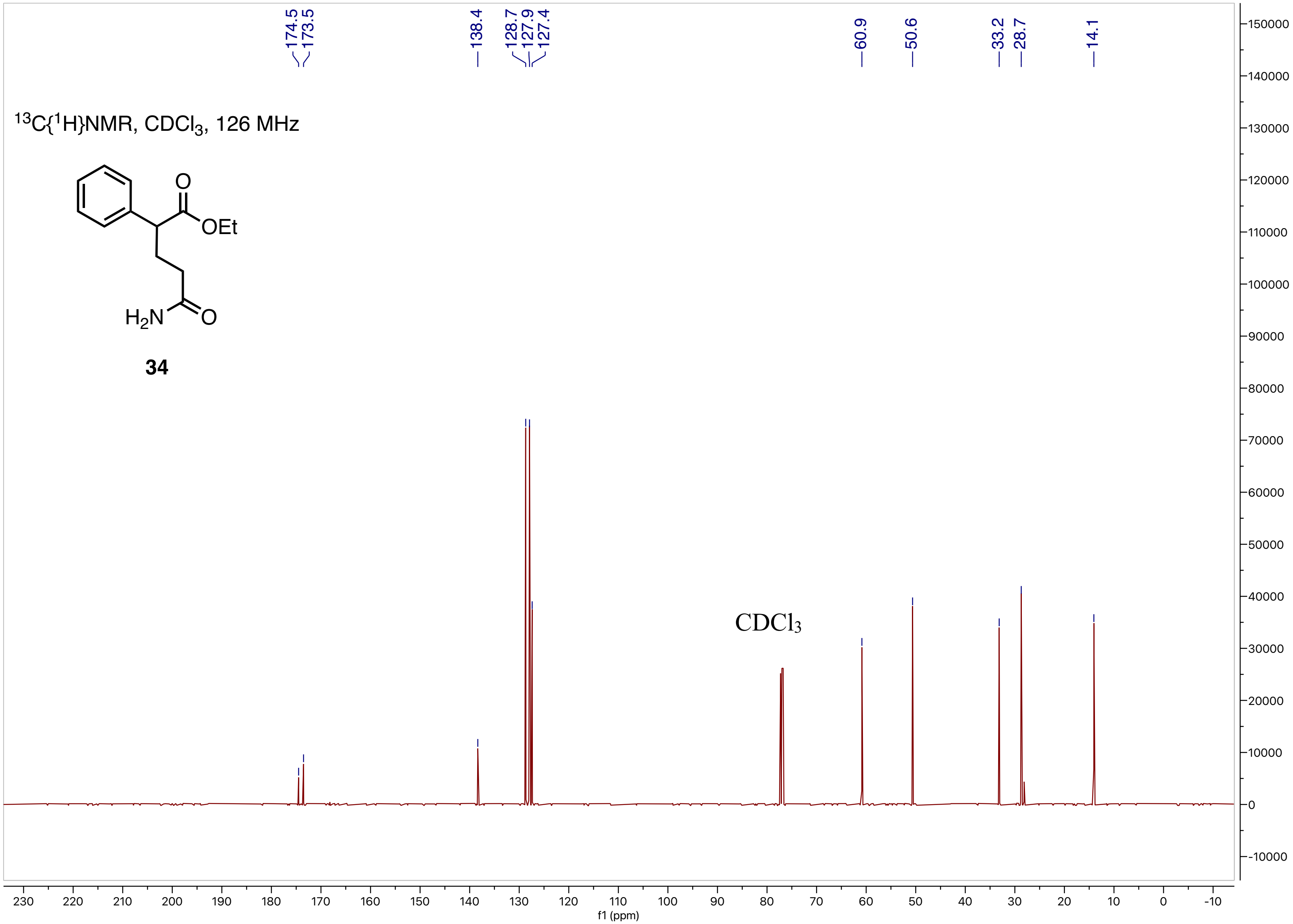

174.5
173.5
138.4
128.7
127.9
127.4
60.9
50.6
33.2
28.7
14.1
^{13}C{^{1}H}NMR, CDCl$_3$, 126 MHz
O
OEt
H$_2$N
O
34
CDCl$_3$
150000
140000
130000
120000
110000
100000
90000
80000
70000
60000
50000
40000
30000
20000
10000
0
-10000
230
220
210
200
190
180
170
160
150
140
130
120
110
100
90
80
70
60
50
40
30
20
10
0
-10
f1 (ppm)

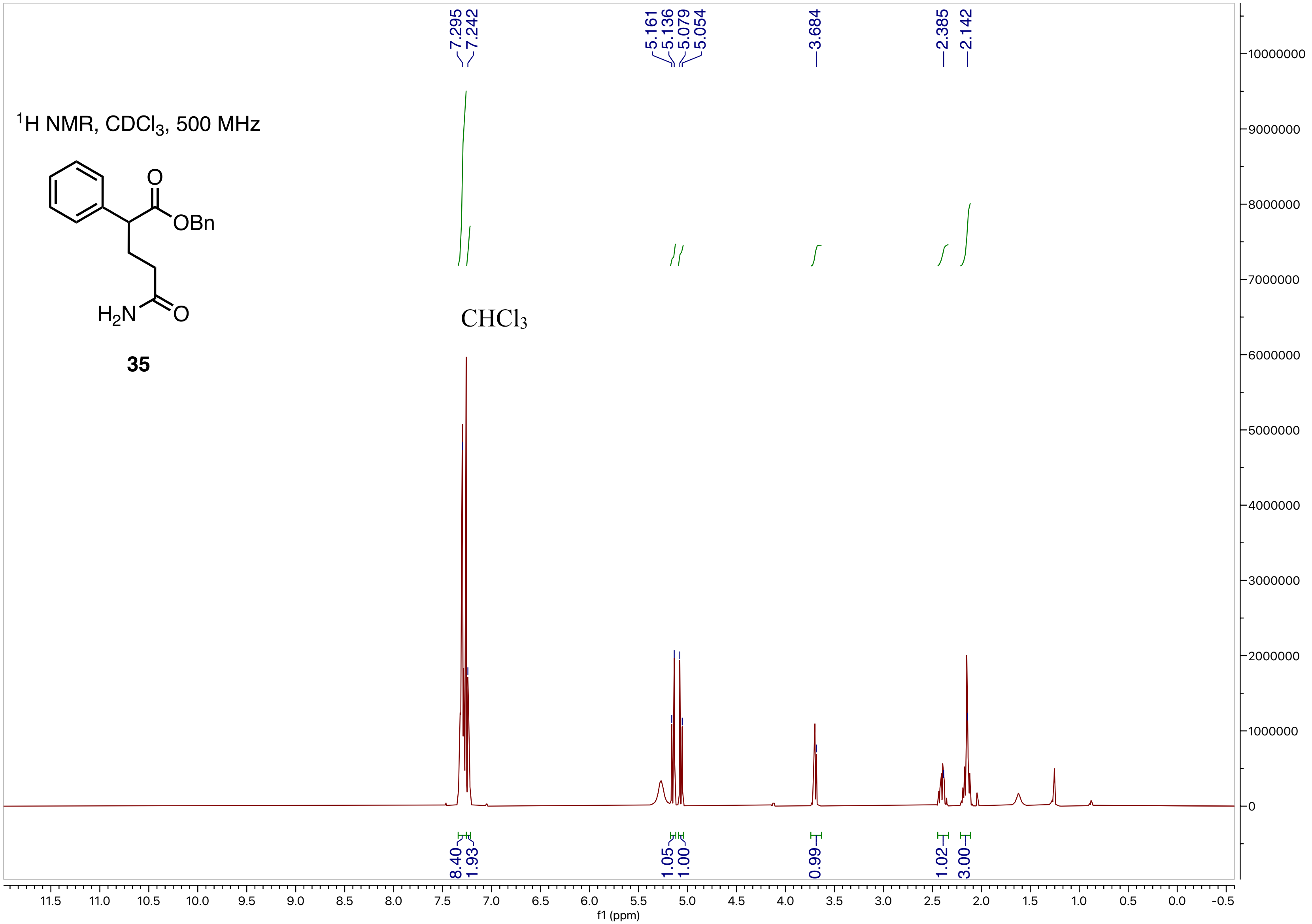

^{1}H NMR, $CDCl_3$, 500 MHz
O
OBn
H_2N
O
35
$CHCl_3$
7.295
7.242
5.161
5.136
5.079
5.054
3.684
2.385
2.142
8.40
1.93
1.05
1.00
0.99
1.02
3.00
f1 (ppm)

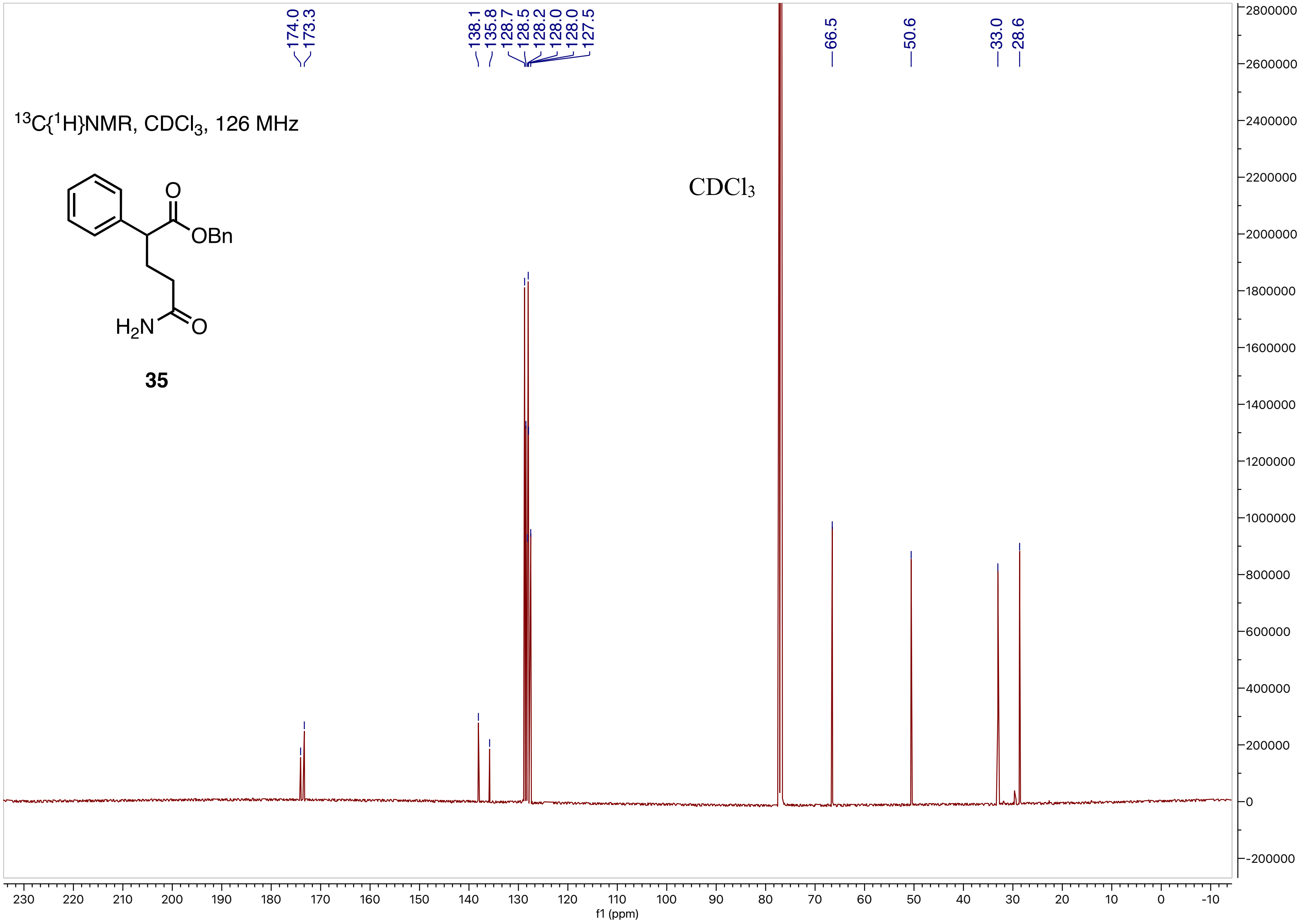

174.0
173.3
138.1
135.8
128.7
128.5
128.2
128.0
128.0
127.5
66.5
50.6
33.0
28.6
13C{1H}NMR, CDCl3, 126 MHz
O
OBn
H2N
O
35
CDCl3
f1 (ppm)

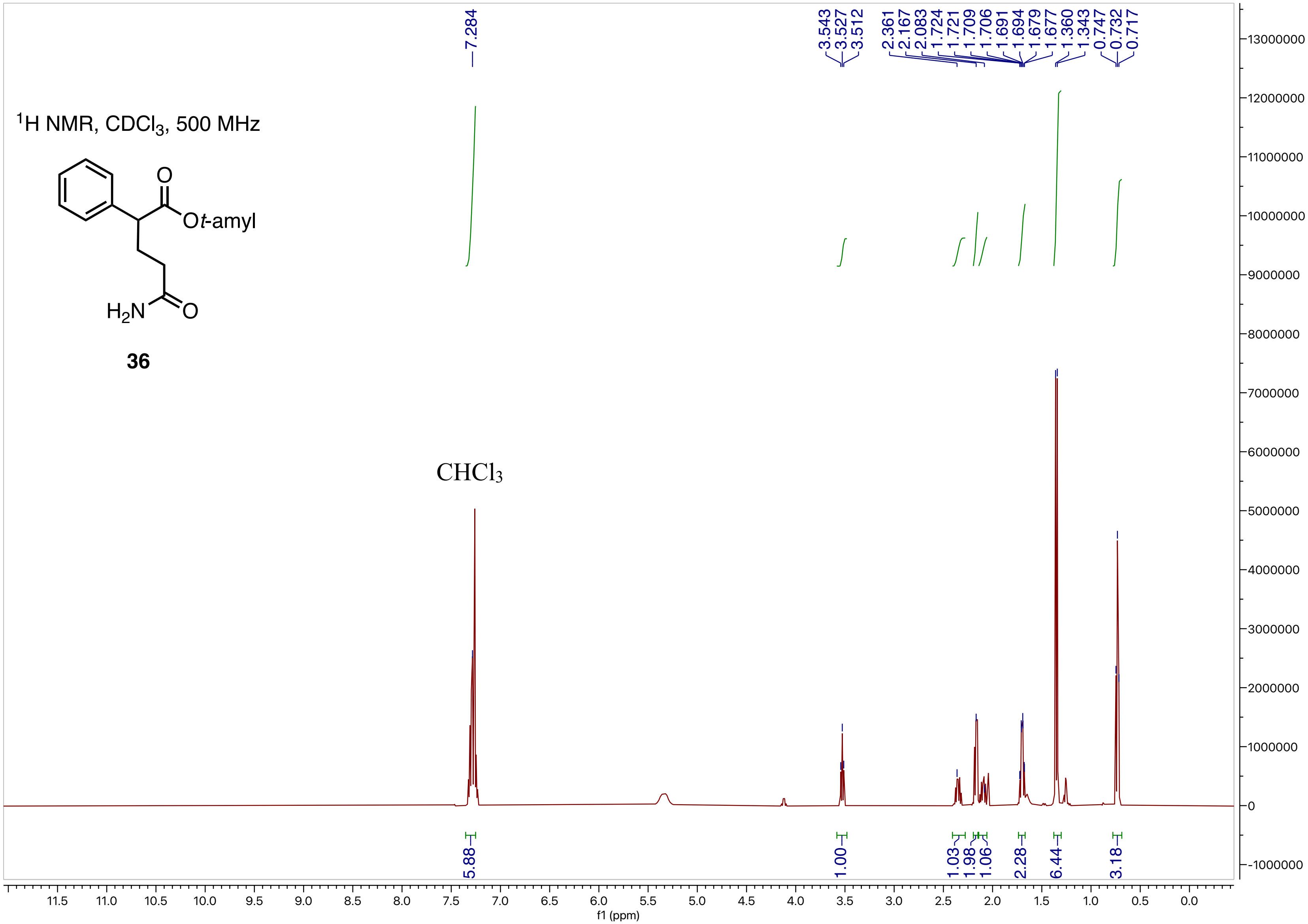

1H NMR, CDCl3, 500 MHz
Ot-amyl
H2N
O
36
CHCl3
7.284
3.543
3.527
3.512
2.361
2.167
2.083
1.724
1.721
1.709
1.706
1.691
1.694
1.679
1.677
1.360
1.343
0.747
0.732
0.717
5.88
1.00
1.03
1.98
1.06
2.28
6.44
3.18
f1 (ppm)

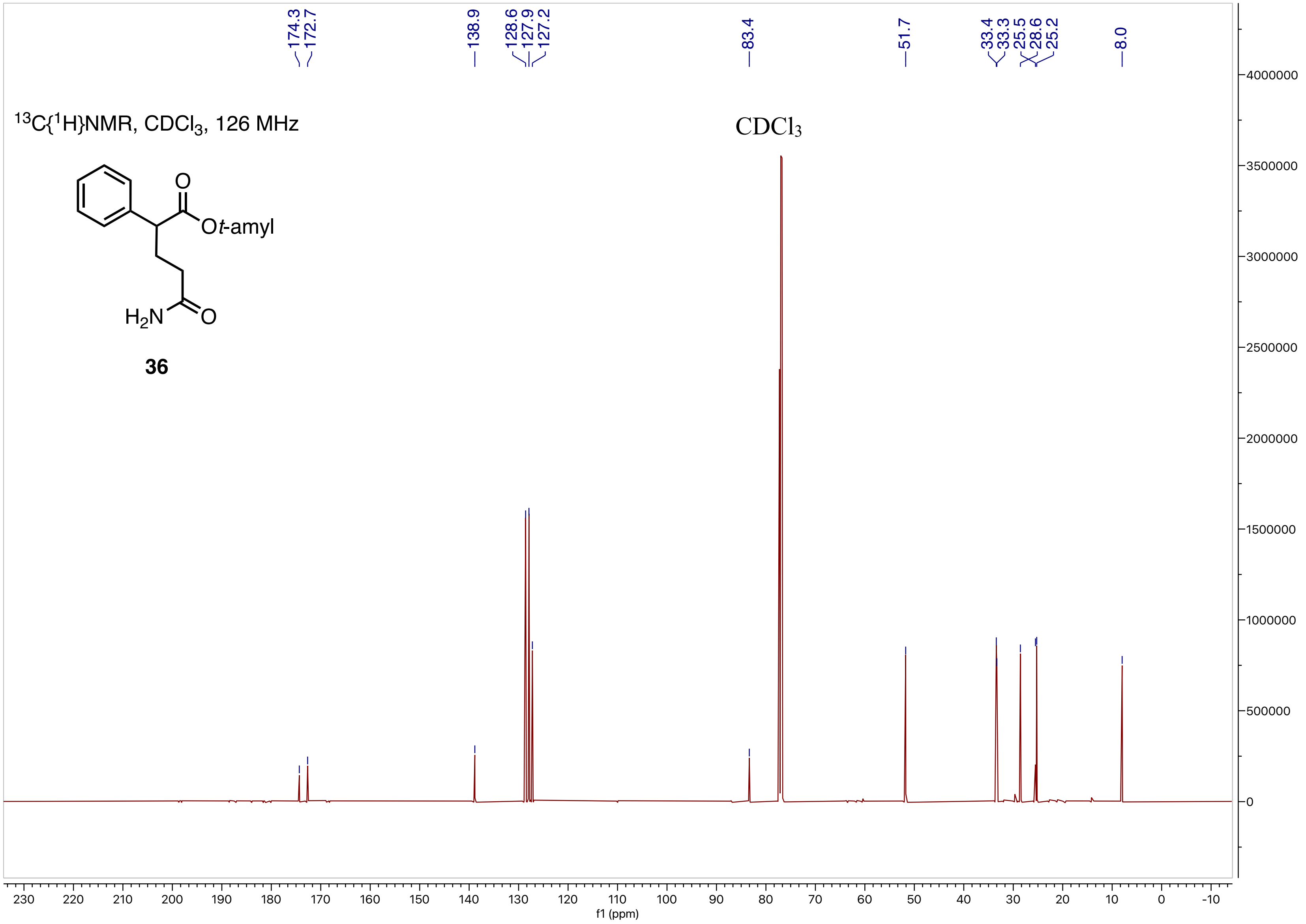
174.3
172.7
138.9
128.6
127.9
127.2
83.4
51.7
33.4
33.3
25.5
28.6
25.2
8.0
13C{1H}NMR, CDCl3, 126 MHz
O
Ot-amyl
H2N
O
36
CDCl3
f1 (ppm)

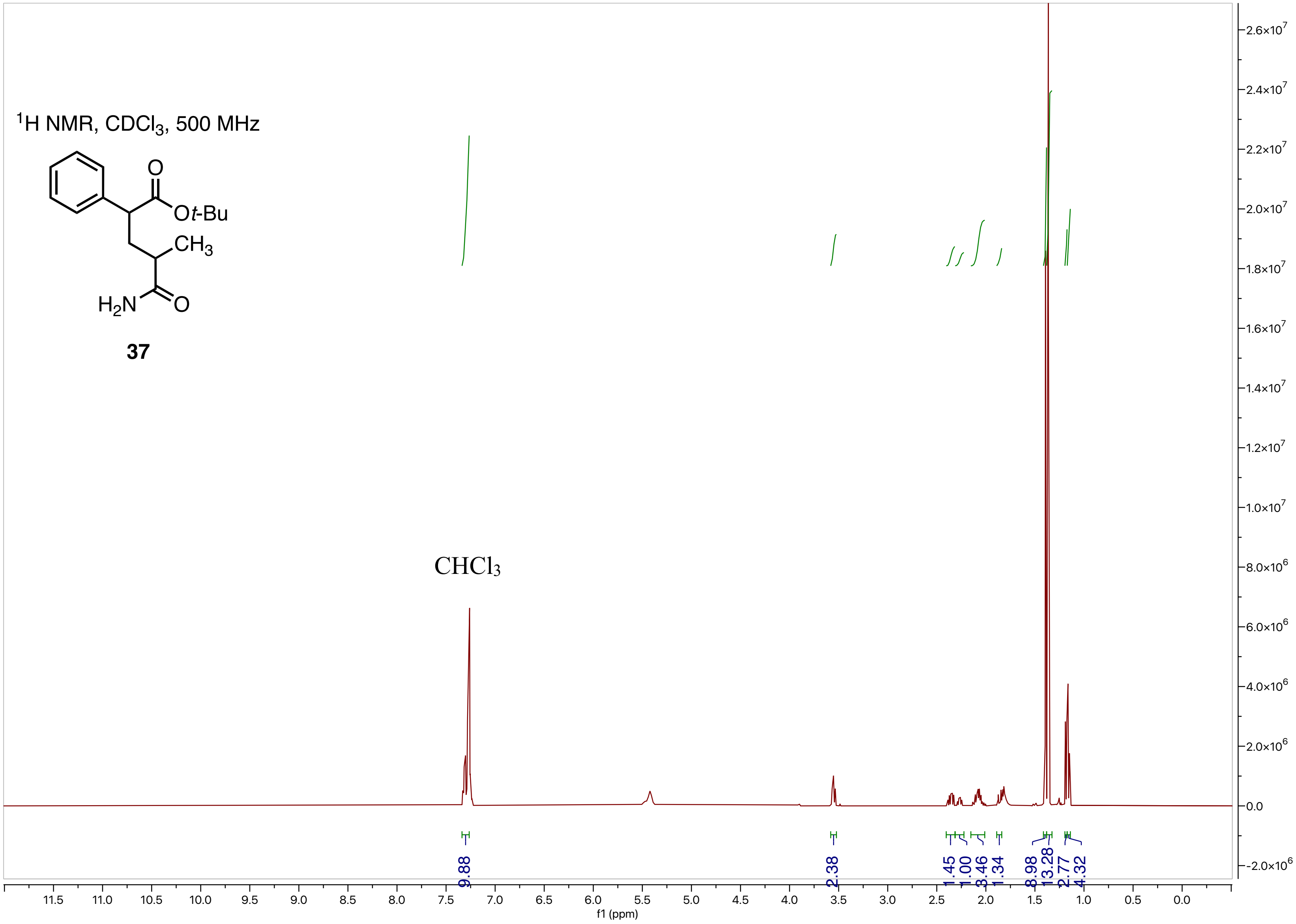

^1H NMR, $CDCl_3$, 500 MHz
O
Ot-Bu
CH_3
H_2N
O
37
$CHCl_3$
9.88
2.38
1.45
1.00
3.46
1.34
8.98
13.28
2.77
4.32
f1 (ppm)

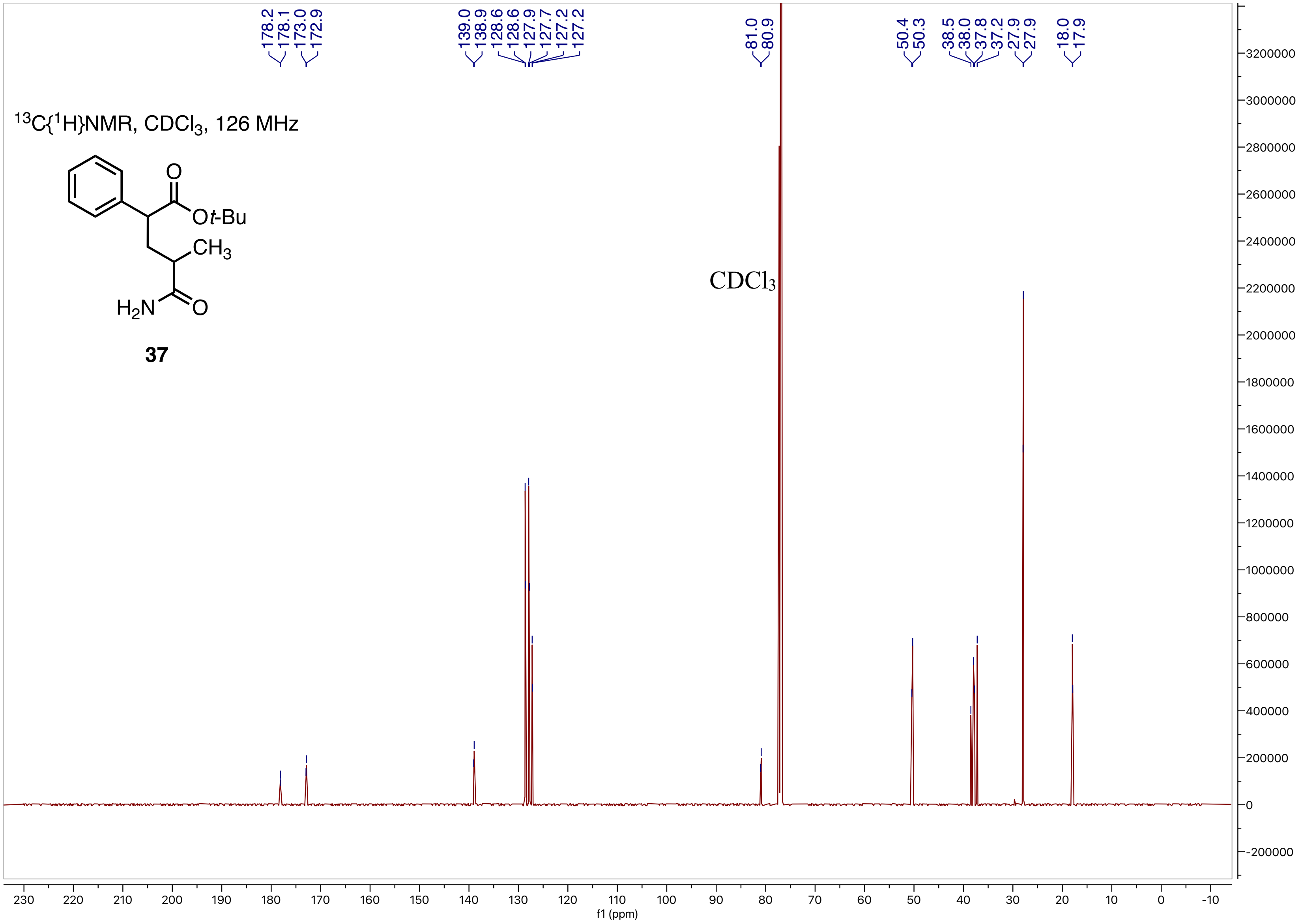
178.2
178.1
173.0
172.9
139.0
138.9
128.6
128.6
127.9
127.7
127.2
127.2
81.0
80.9
50.4
50.3
38.5
38.0
37.8
37.2
27.9
27.9
18.0
17.9
13C{1H}NMR, CDCl3, 126 MHz
O
Ot-Bu
CH3
H2N
O
37
CDCl3
f1 (ppm)

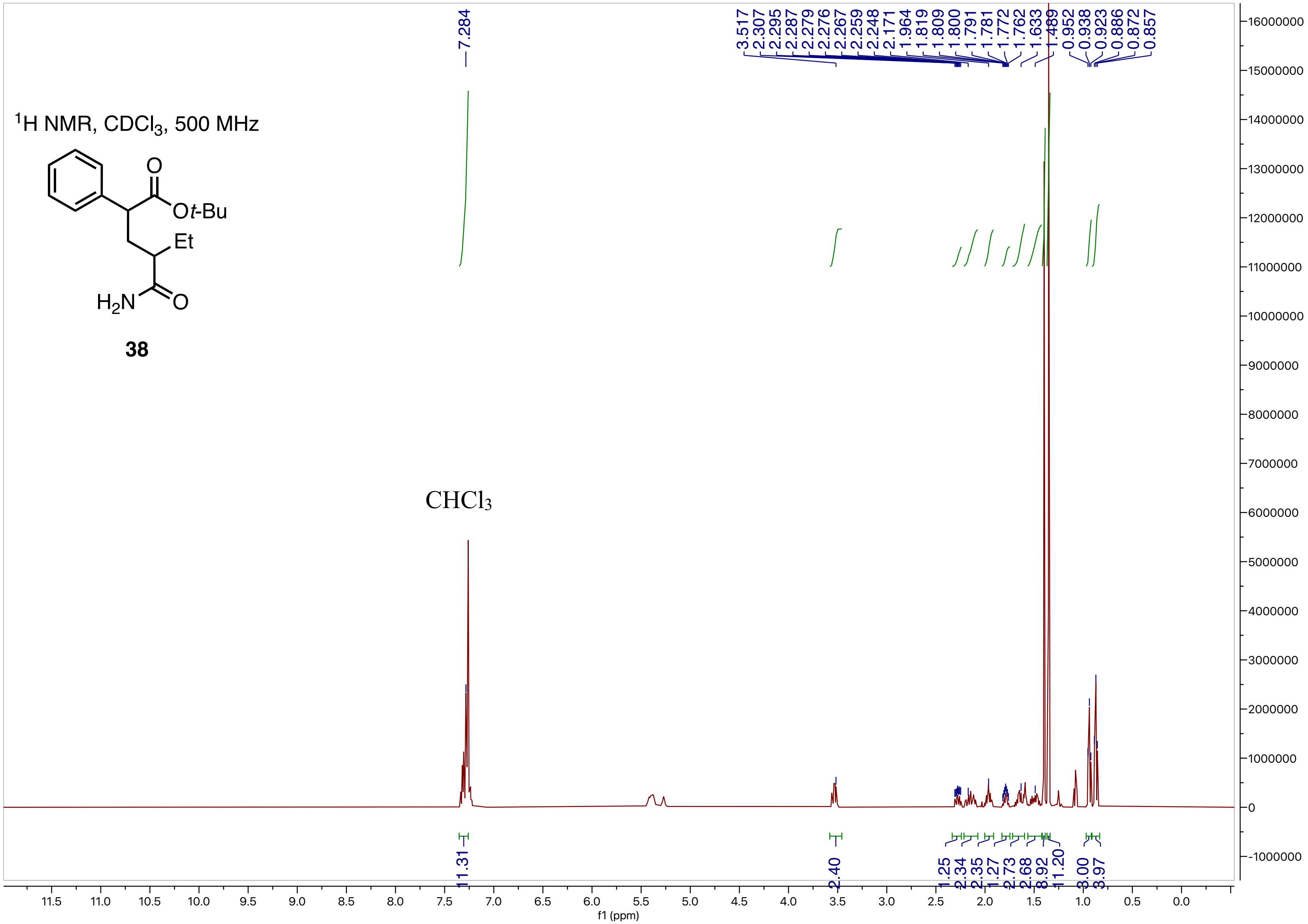

^{1}H NMR, $CDCl_3$, 500 MHz
38
CHCl3
7.284
3.517
2.307
2.295
2.287
2.279
2.276
2.267
2.259
2.248
2.171
1.964
1.819
1.809
1.800
1.791
1.781
1.772
1.762
1.633
1.489
0.952
0.938
0.923
0.886
0.872
0.857
11.31
2.40
1.25
2.34
2.35
1.27
2.73
2.68
8.92
11.20
3.00
3.97
f1 (ppm)

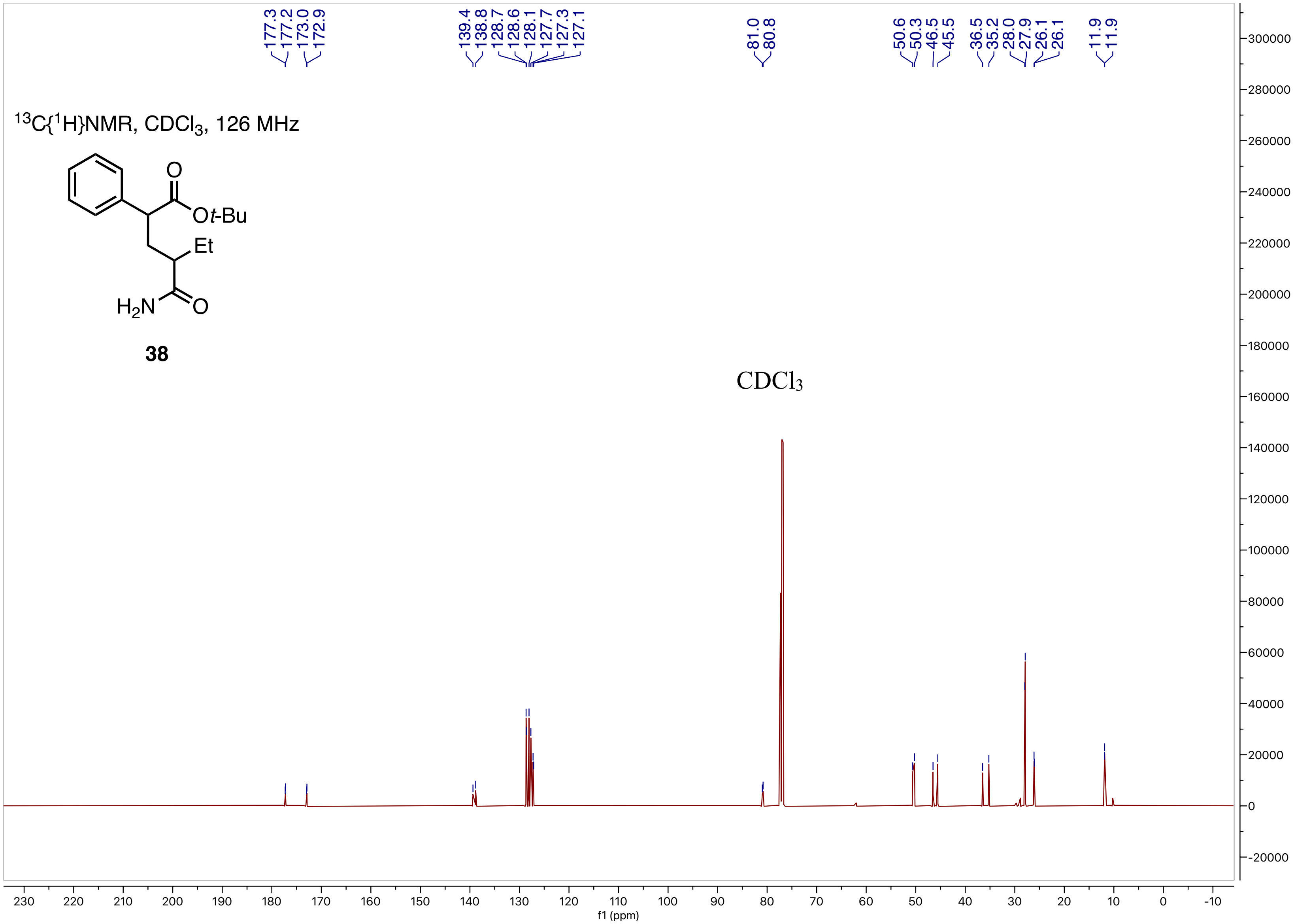
177.3
177.2
173.0
172.9
139.4
138.8
128.7
128.6
128.1
127.7
127.3
127.1
81.0
80.8
50.6
50.3
46.5
45.5
36.5
35.2
28.0
27.9
26.1
26.1
11.9
11.9
$^{13}C\{^1H\}$NMR, $CDCl_3$, 126 MHz
O
O*t*-Bu
Et
H_2N
O
38
$CDCl_3$
f1 (ppm)

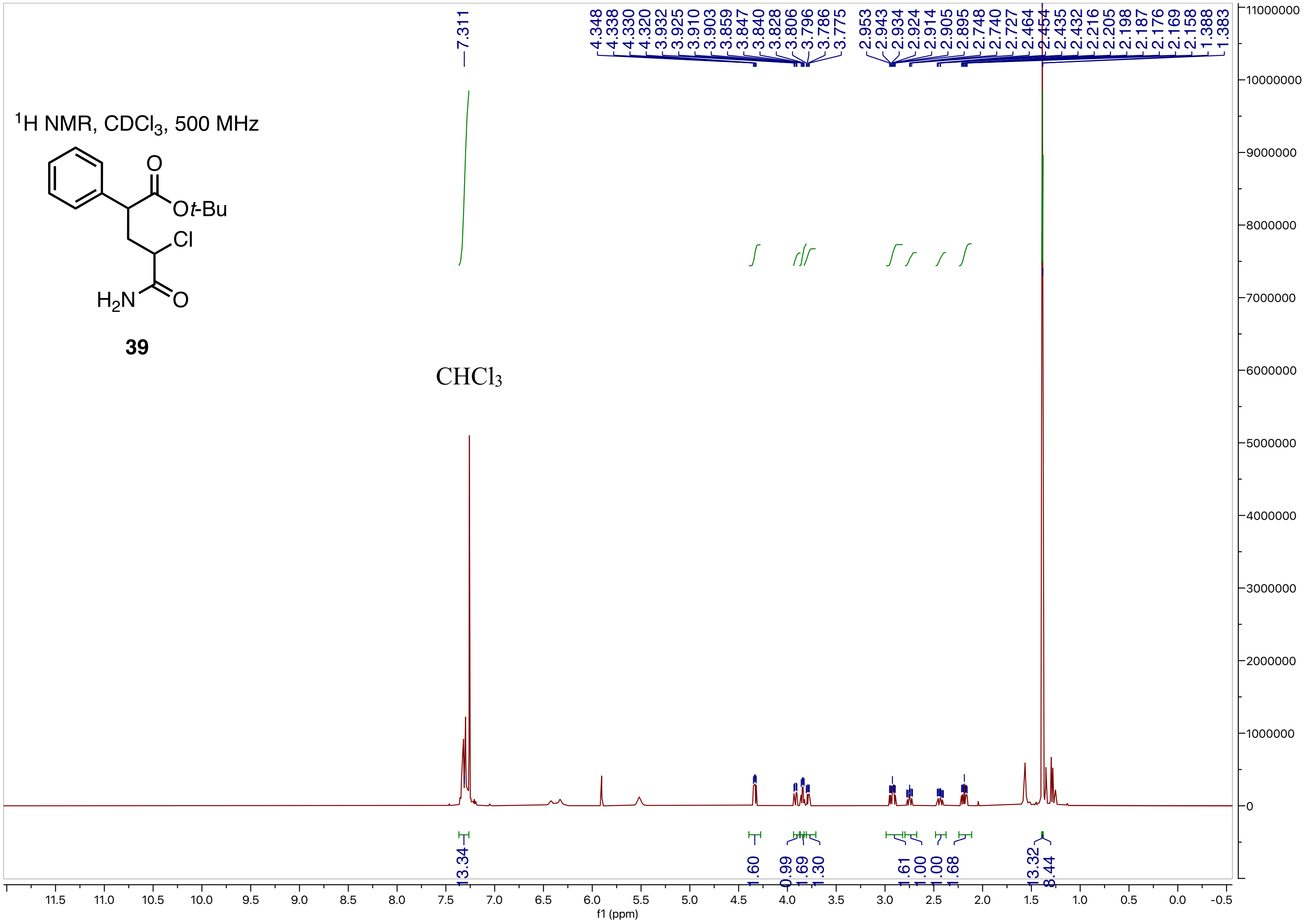

^{1}H NMR, $CDCl_3$, 500 MHz
39
CHCl3
f1 (ppm)

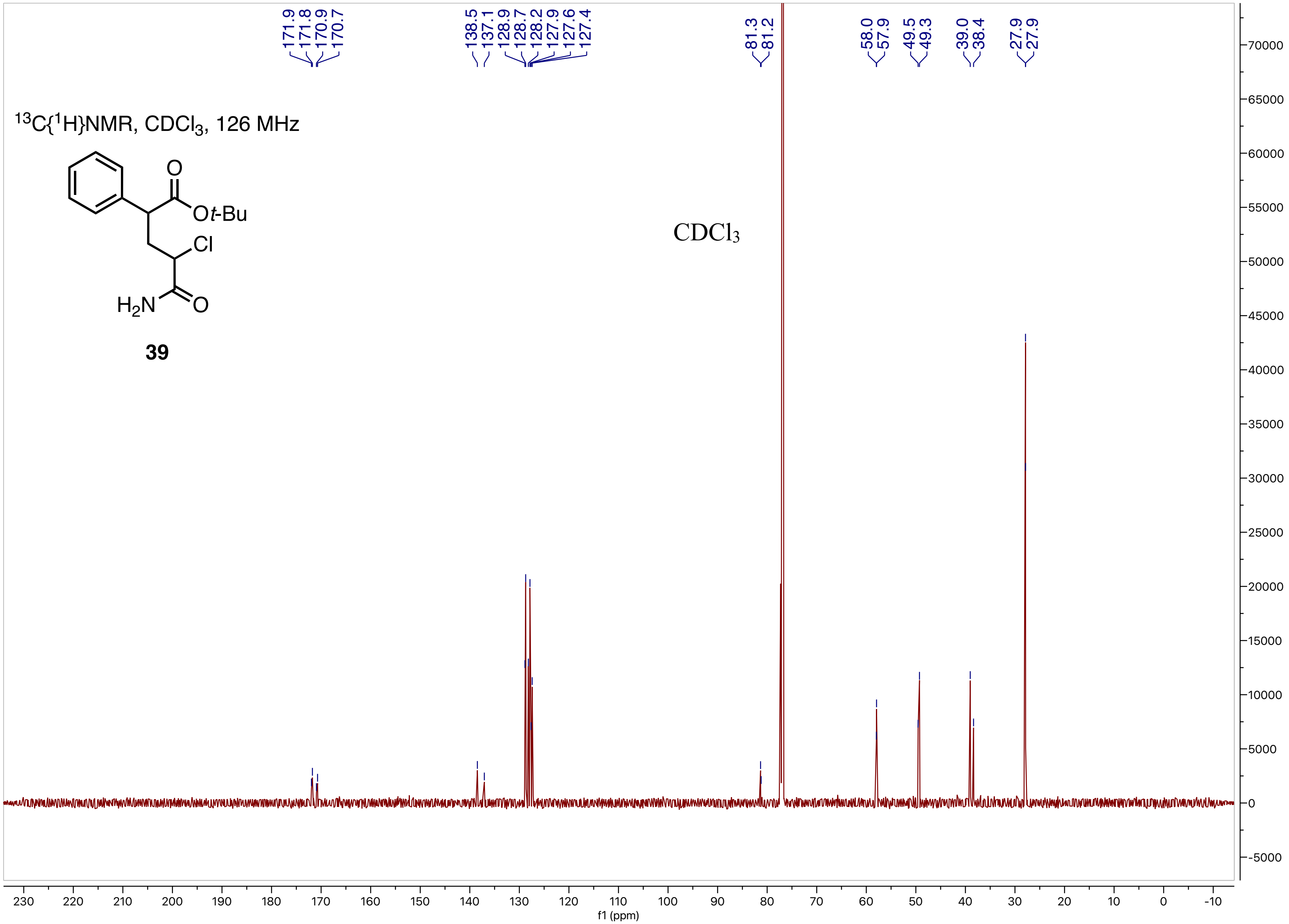
171.9
171.8
170.9
170.7
138.5
137.1
128.9
128.7
128.2
127.9
127.6
127.4
81.3
81.2
58.0
57.9
49.5
49.3
39.0
38.4
27.9
27.9
13C{1H}NMR, CDCl3, 126 MHz
O
Ot-Bu
Cl
H2N
O
39
CDCl3
f1 (ppm)

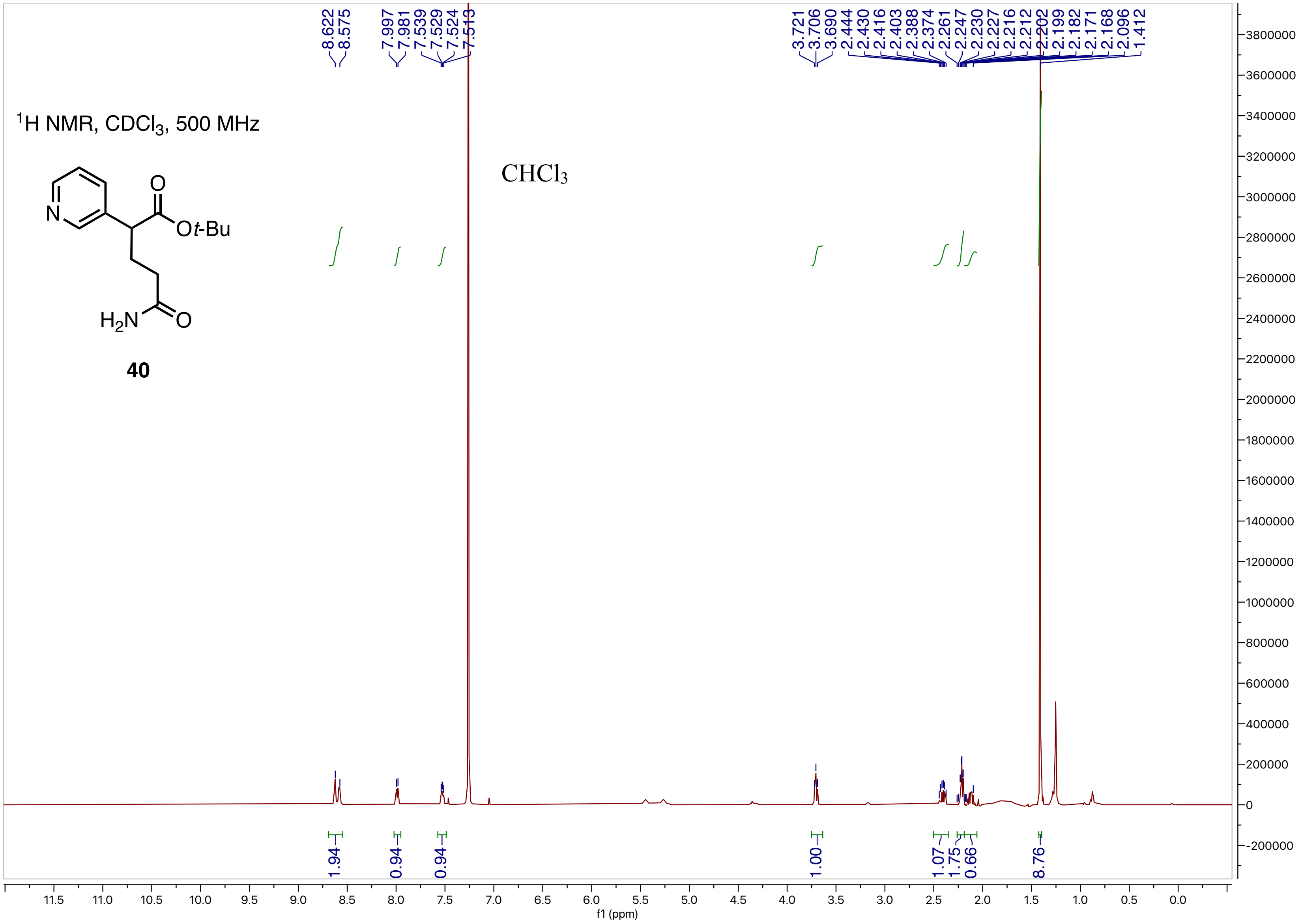

^{1}H NMR, $CDCl_3$, 500 MHz
N
O
O*t*-Bu
H_2N
O
40
CHCl3
8.622
8.575
7.997
7.981
7.539
7.529
7.524
7.513
3.721
3.706
3.690
2.444
2.430
2.416
2.403
2.388
2.374
2.261
2.247
2.230
2.227
2.216
2.212
2.202
2.199
2.182
2.171
2.168
2.096
1.412
1.94
0.94
0.94
1.00
1.07
1.75
0.66
8.76
f1 (ppm)

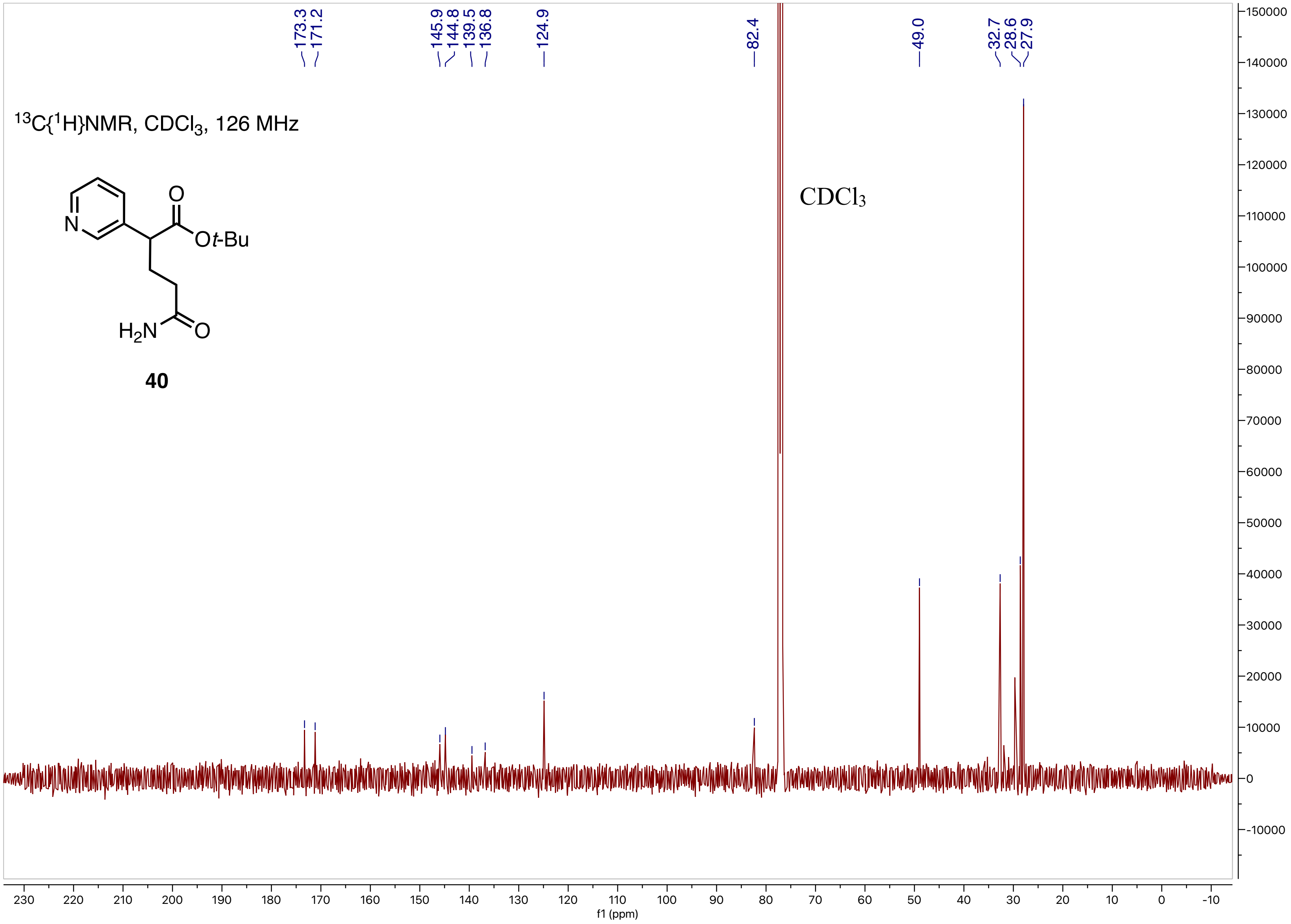
173.3
171.2
145.9
144.8
139.5
136.8
124.9
82.4
49.0
32.7
28.6
27.9
13C{1H}NMR, CDCl3, 126 MHz
N
O
Ot-Bu
H2N
O
40
CDCl3
150000
140000
130000
120000
110000
100000
90000
80000
70000
60000
50000
40000
30000
20000
10000
0
-10000
230
220
210
200
190
180
170
160
150
140
130
120
110
100
90
80
70
60
50
40
30
20
10
0
-10
f1 (ppm)

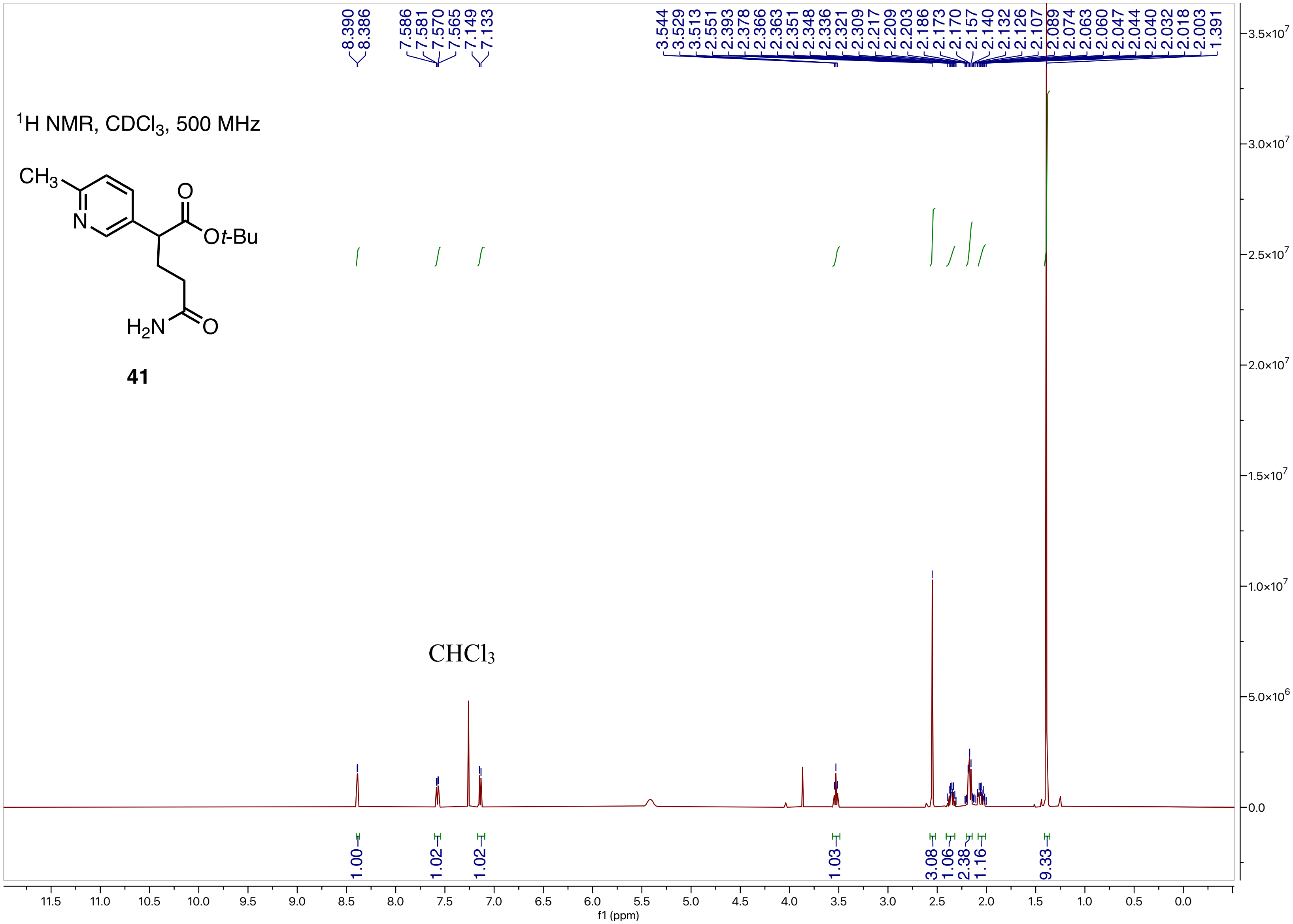

8.390
8.386
7.586
7.581
7.570
7.565
7.149
7.133
3.544
3.529
3.513
2.551
2.393
2.378
2.366
2.363
2.351
2.348
2.336
2.321
2.309
2.217
2.209
2.203
2.186
2.173
2.170
2.157
2.140
2.132
2.126
2.107
2.089
2.074
2.063
2.060
2.047
2.044
2.040
2.032
2.018
2.003
1.391
1H NMR, CDCl3, 500 MHz
CH3
N
O
Ot-Bu
H2N
O
41
CHCl3
1.00
1.02
1.02
1.03
3.08
1.06
2.38
1.16
9.33
f1 (ppm)

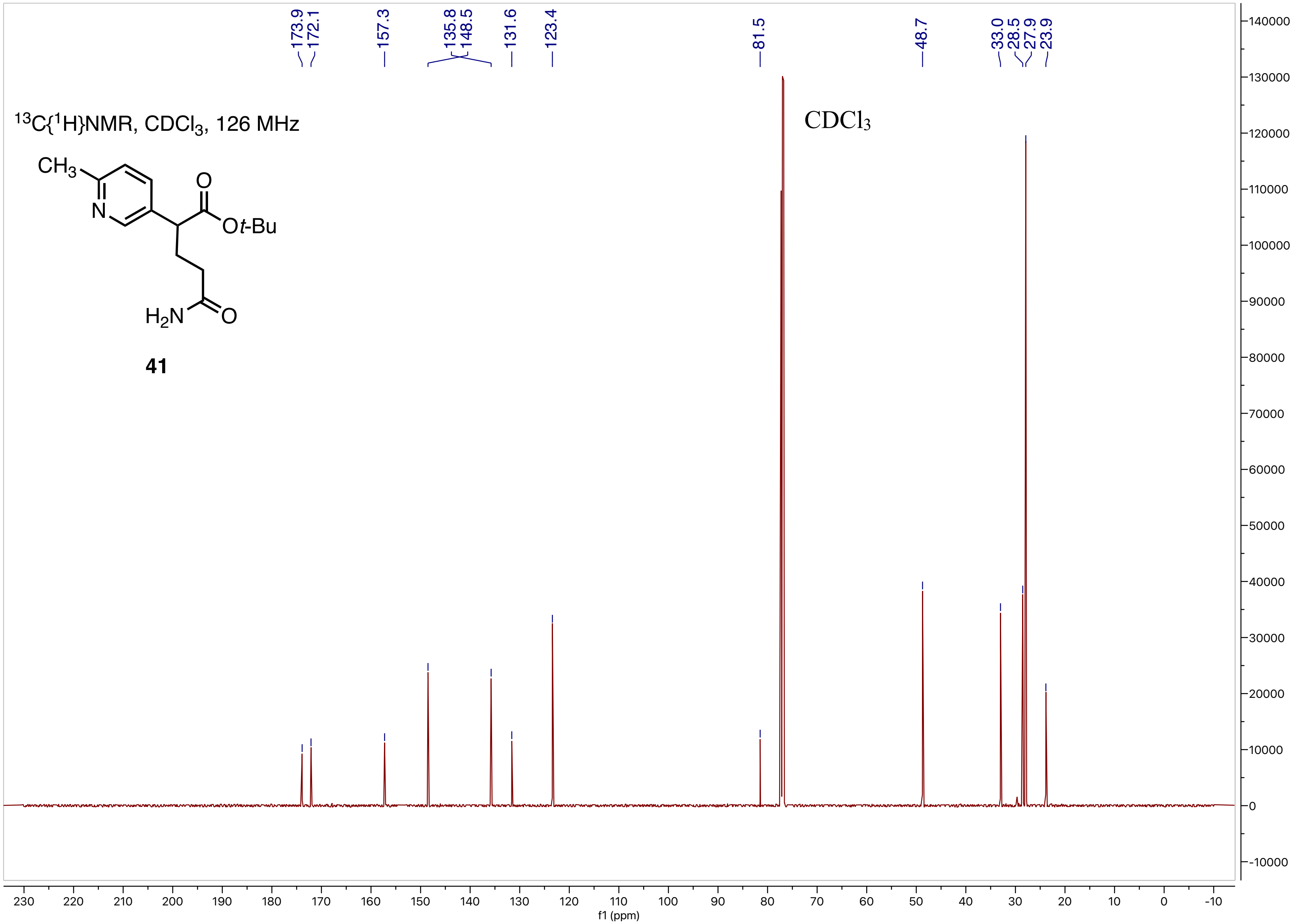

173.9
172.1
157.3
135.8
148.5
131.6
123.4
81.5
48.7
33.0
28.5
27.9
23.9
13C{1H}NMR, CDCl3, 126 MHz
CH3
N
O
Ot-Bu
H2N
O
41
CDCl3
140000
130000
120000
110000
100000
90000
80000
70000
60000
50000
40000
30000
20000
10000
0
-10000
230
220
210
200
190
180
170
160
150
140
130
120
110
100
90
80
70
60
50
40
30
20
10
0
-10
f1 (ppm)

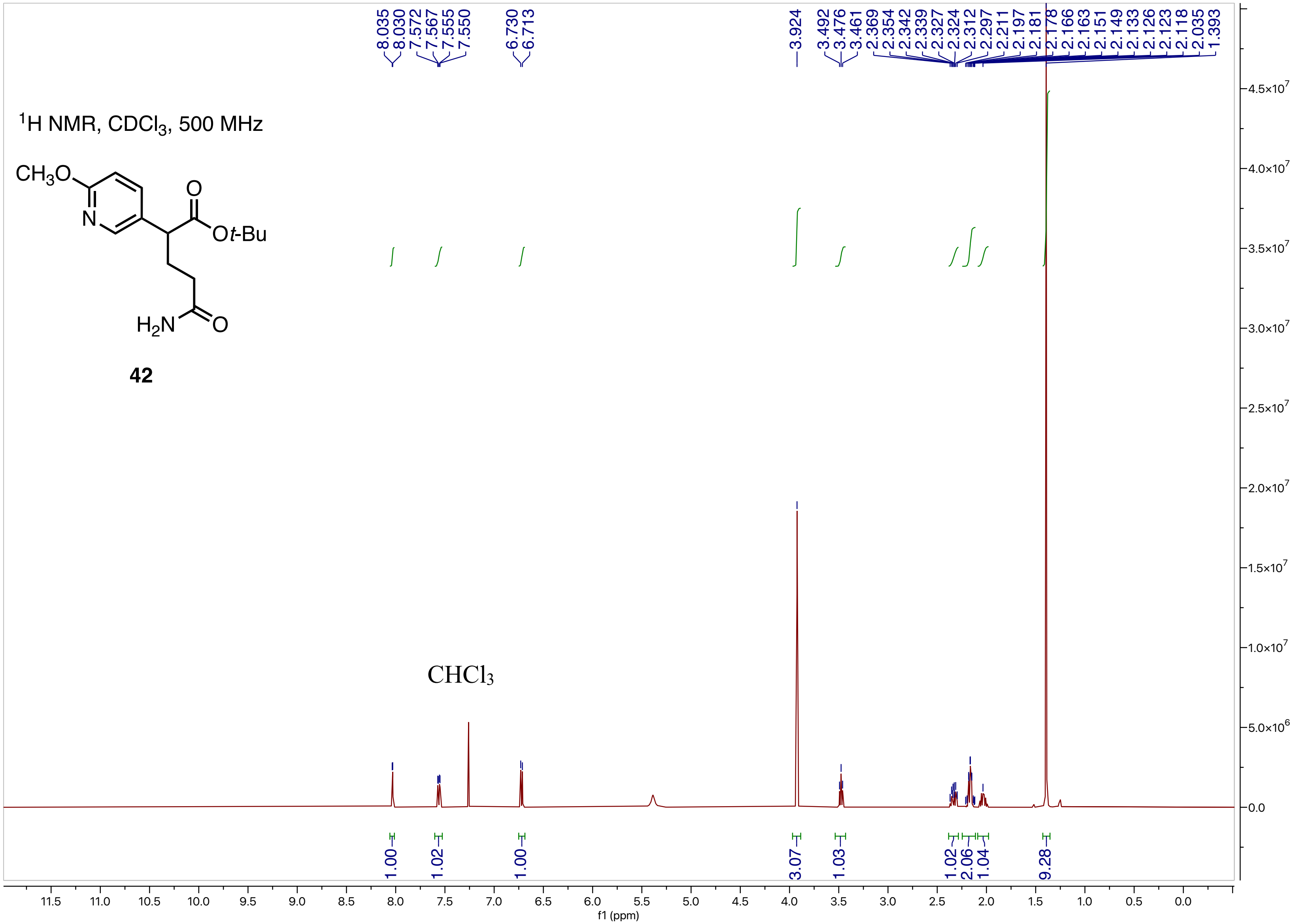

8.035
8.030
7.572
7.567
7.555
7.550
6.730
6.713
3.924
3.492
3.476
3.461
2.369
2.354
2.342
2.339
2.327
2.324
2.312
2.297
2.211
2.197
2.181
2.178
2.166
2.163
2.151
2.149
2.133
2.126
2.123
2.118
2.035
1.393
^{1}H NMR, $CDCl_3$, 500 MHz
CH_3O
N
O
O*t*-Bu
H_2N
O
42
CHCl3
1.00
1.02
1.00
3.07
1.03
1.02
2.06
1.04
9.28
f1 (ppm)

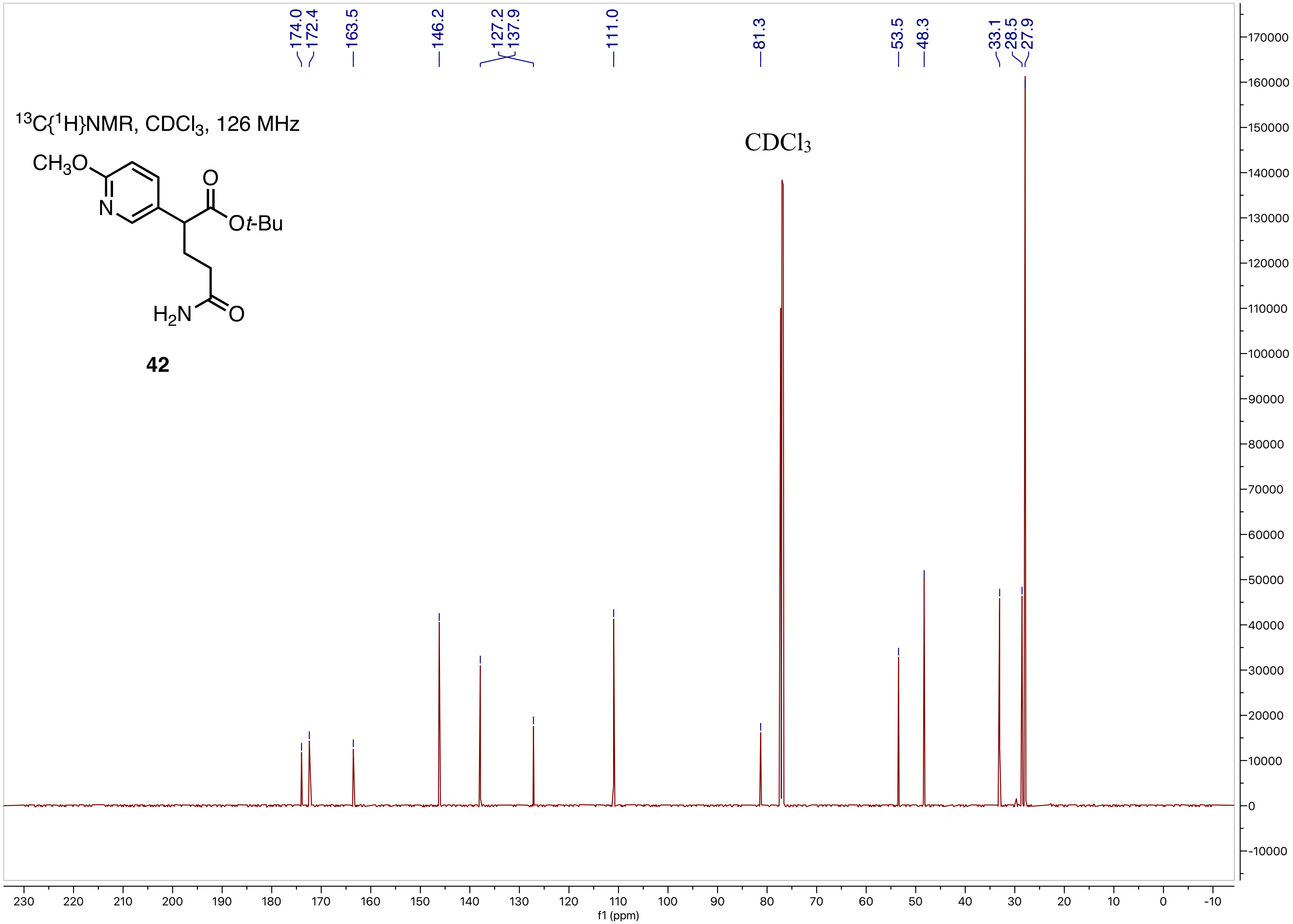
13C{1H}NMR, CDCl3, 126 MHz
CH3O
N
O
Ot-Bu
H2N
O
42
CDCl3
174.0
172.4
163.5
146.2
127.2
137.9
111.0
81.3
53.5
48.3
33.1
28.5
27.9
f1 (ppm)

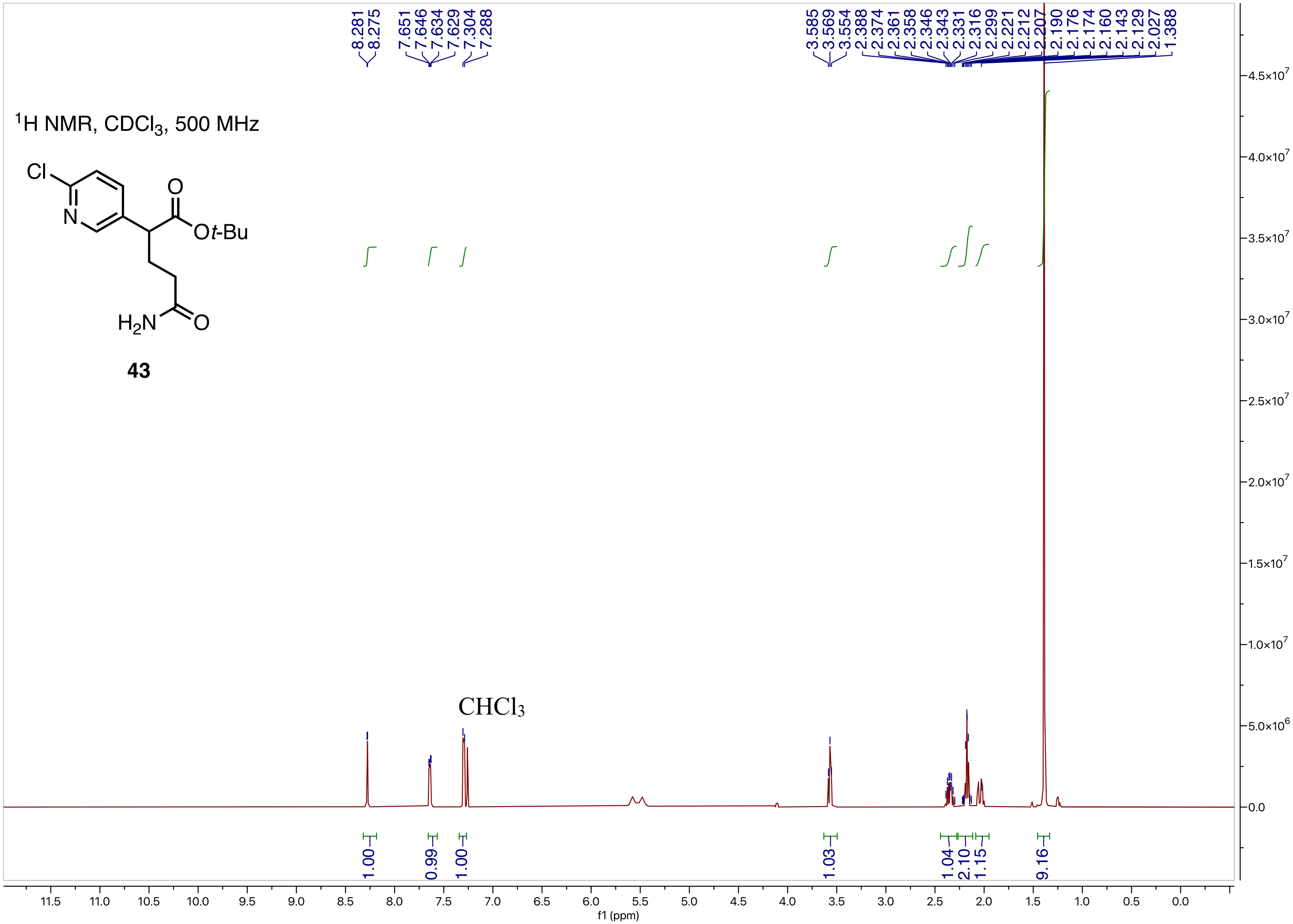

1H NMR, CDCl3, 500 MHz
Cl
N
O
Ot-Bu
H2N
O
43
CHCl3
8.281
8.275
7.651
7.646
7.634
7.629
7.304
7.288
3.585
3.569
3.554
2.388
2.374
2.361
2.358
2.346
2.343
2.331
2.316
2.299
2.221
2.212
2.207
2.190
2.176
2.174
2.160
2.143
2.129
2.027
1.388
1.00
0.99
1.00
1.03
1.04
2.10
1.15
9.16
f1 (ppm)

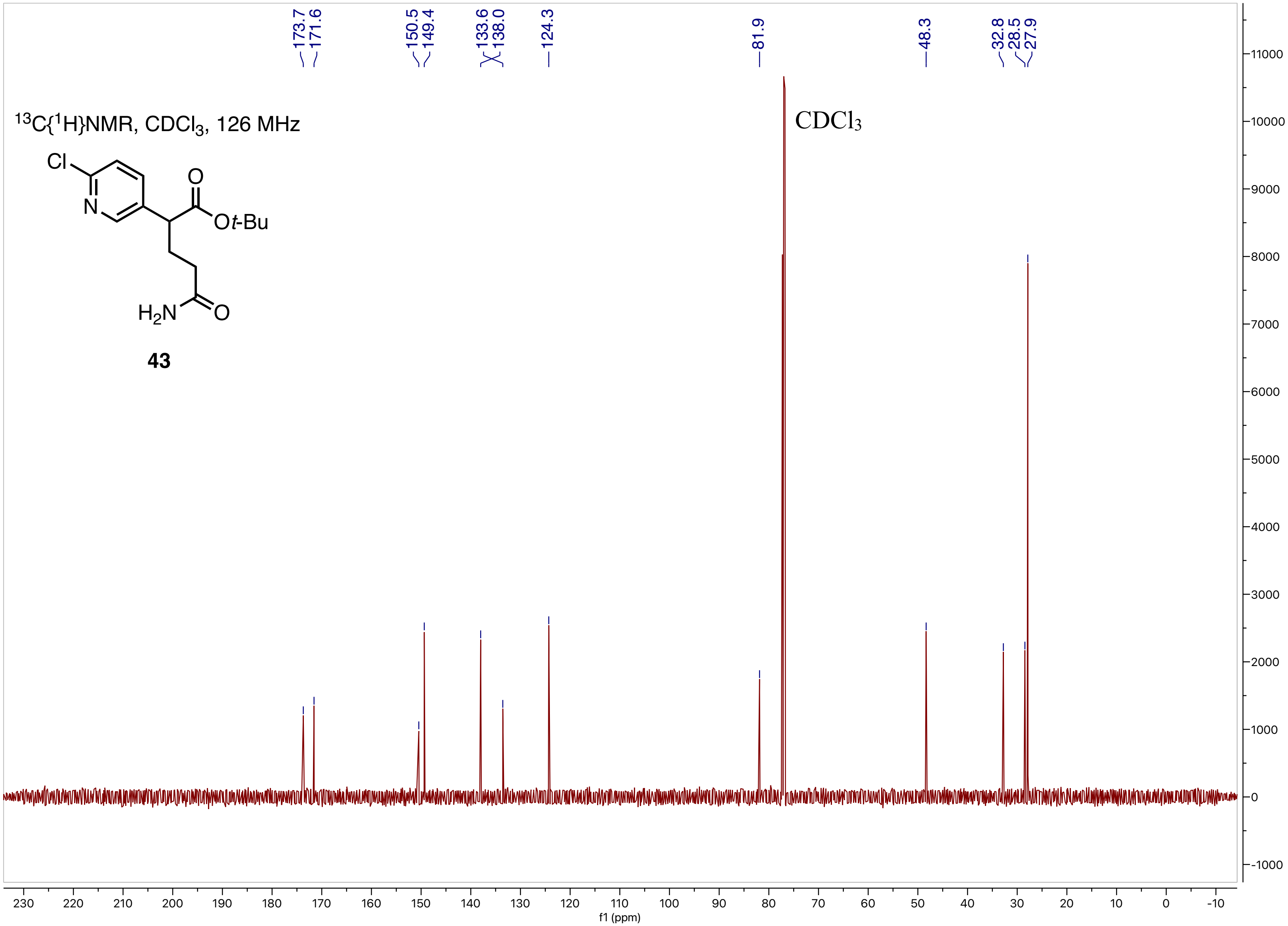
173.7
171.6
150.5
149.4
133.6
138.0
124.3
81.9
48.3
32.8
28.5
27.9
$^{13}C\{^1H\}NMR$, $CDCl_3$, 126 MHz
Cl
N
O
Ot-Bu
H_2N
O
43
$CDCl_3$
11000
10000
9000
8000
7000
6000
5000
4000
3000
2000
1000
0
-1000
230 220 210 200 190 180 170 160 150 140 130 120 110 100 90 80 70 60 50 40 30 20 10 0 -10
f1 (ppm)

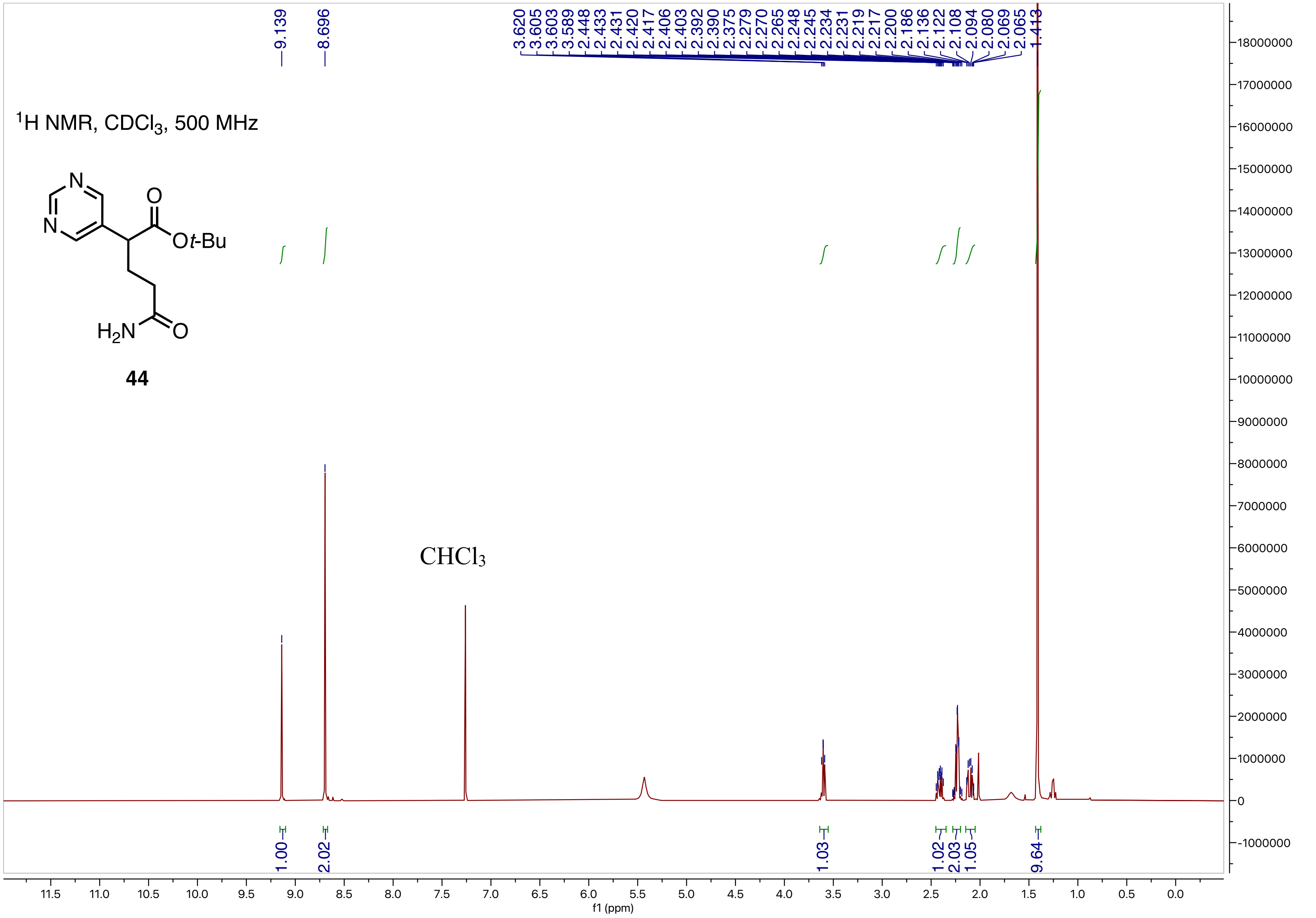

1H NMR, CDCl3, 500 MHz
44
CHCl3
9.139
8.696
3.620
3.605
3.603
3.589
2.448
2.433
2.431
2.420
2.417
2.406
2.403
2.392
2.390
2.375
2.279
2.270
2.265
2.248
2.245
2.234
2.231
2.219
2.217
2.200
2.186
2.136
2.122
2.108
2.094
2.080
2.069
2.065
1.413
1.00
2.02
1.03
1.02
2.03
1.05
9.64
f1 (ppm)

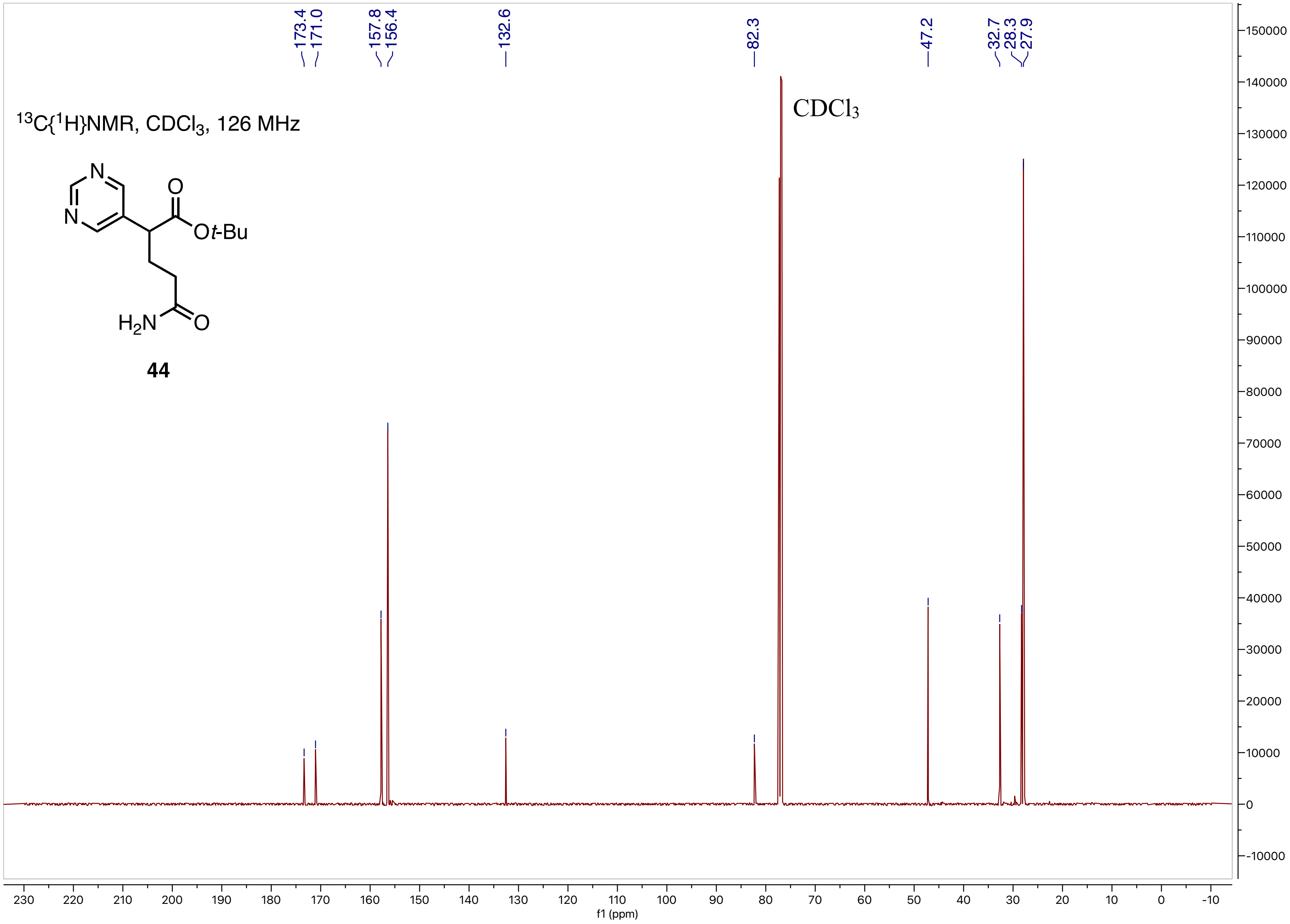
173.4
171.0
157.8
156.4
132.6
82.3
47.2
32.7
28.3
27.9
CDCl3
13C{1H}NMR, CDCl3, 126 MHz
N
N
O
Ot-Bu
H2N
O
44
f1 (ppm)

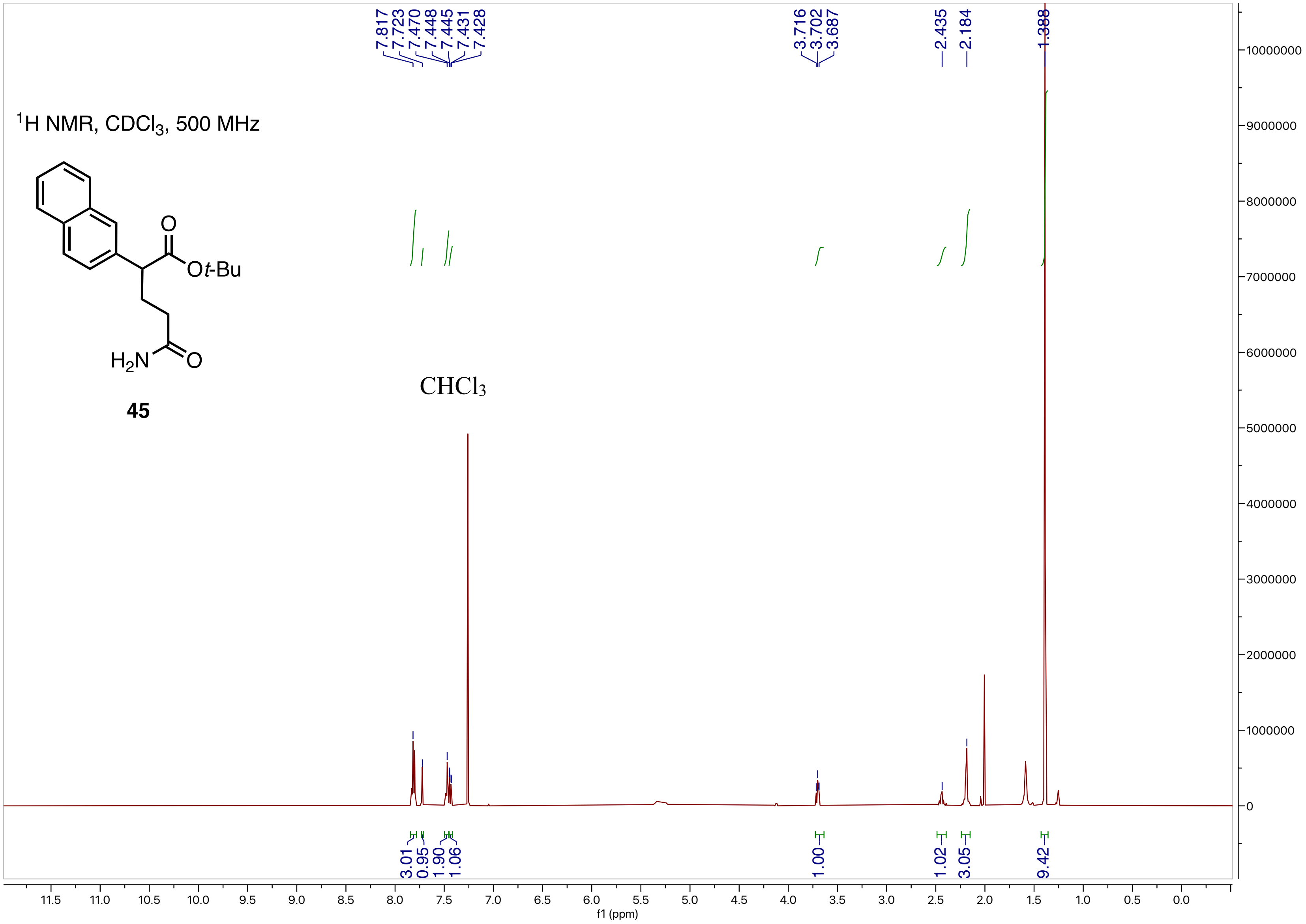

7.817
7.723
7.470
7.448
7.445
7.431
7.428
3.716
3.702
3.687
2.435
2.184
1.388
1H NMR, CDCl3, 500 MHz
O
Ot-Bu
H2N
O
45
CHCl3
3.01
0.95
1.90
1.06
1.00
1.02
3.05
9.42
f1 (ppm)

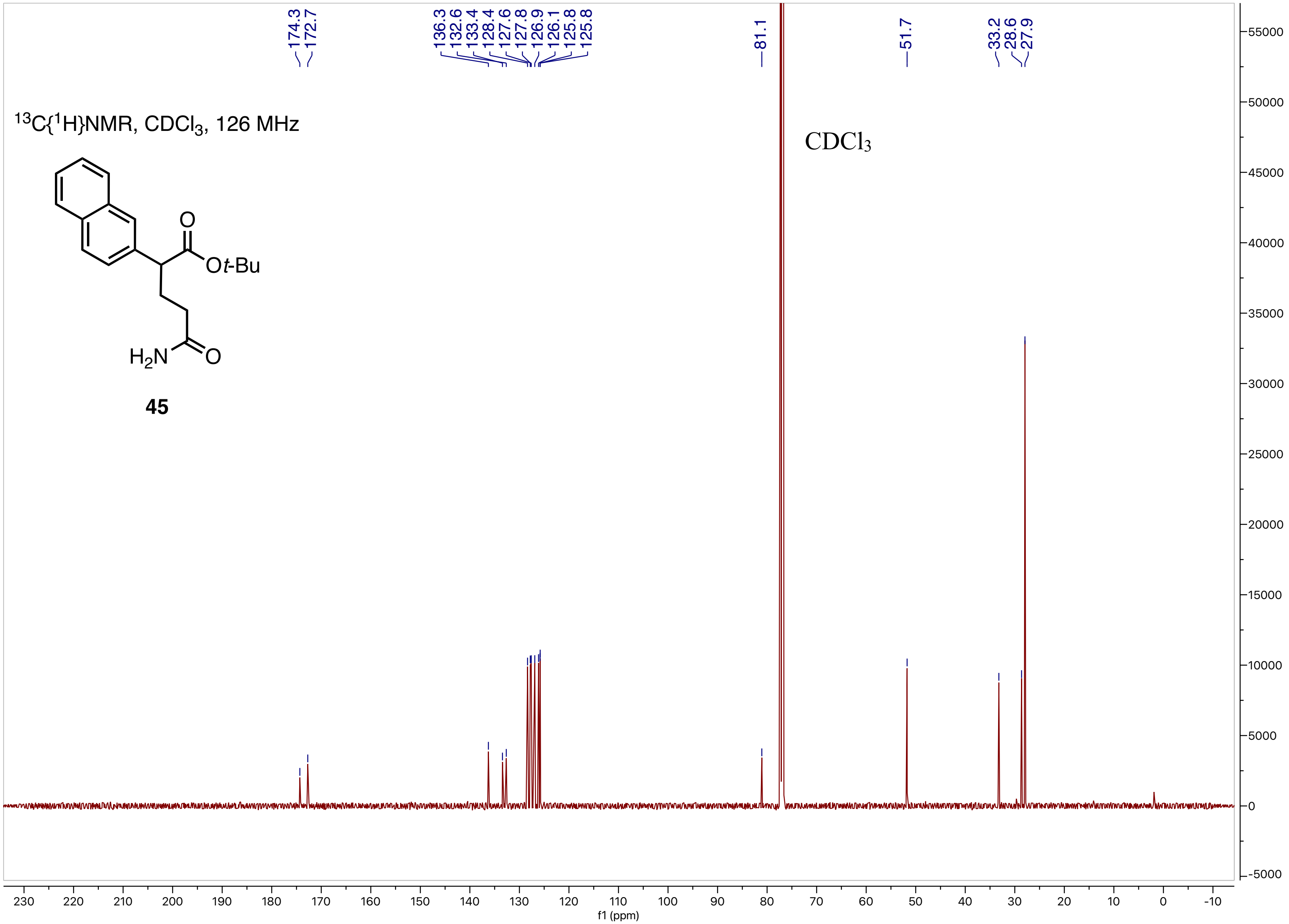

174.3
172.7
136.3
132.6
133.4
128.4
127.6
127.8
126.9
126.1
125.8
125.8
81.1
51.7
33.2
28.6
27.9
$^{13}C\{^1H\}$NMR, $CDCl_3$, 126 MHz
O
O*t*-Bu
H_2N
O
45
CDCl₃
f1 (ppm)

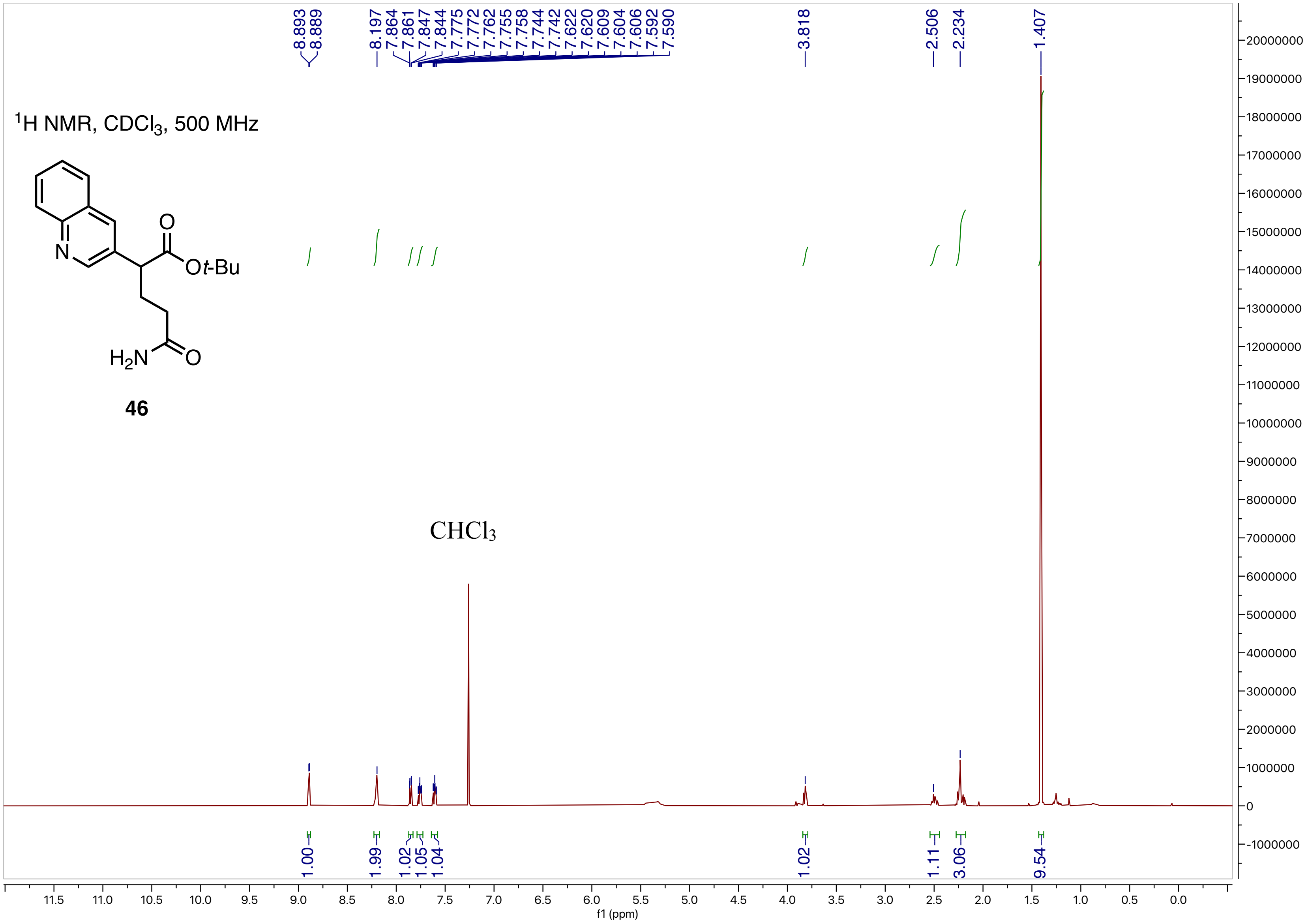

^{1}H NMR, $CDCl_3$, 500 MHz
46
Ot-Bu
H_2N
CHCl₃
8.893
8.889
8.197
7.864
7.861
7.847
7.844
7.775
7.772
7.762
7.755
7.758
7.744
7.742
7.622
7.620
7.609
7.604
7.606
7.592
7.590
3.818
2.506
2.234
1.407
1.00
1.99
1.02
1.05
1.04
1.02
1.11
3.06
9.54
f1 (ppm)

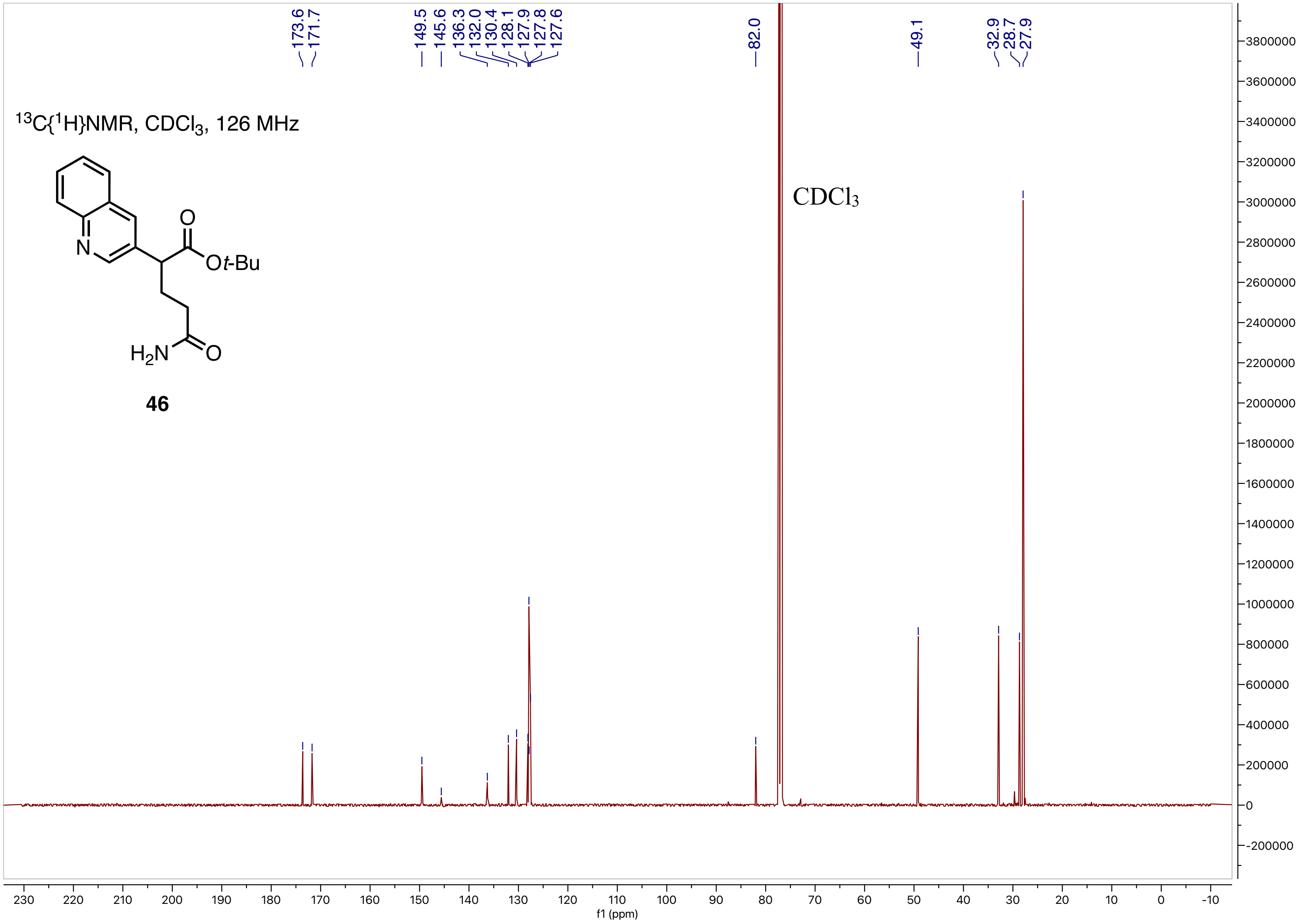

173.6
171.7
149.5
145.6
136.3
132.0
130.4
128.1
127.9
127.8
127.6
82.0
49.1
32.9
28.7
27.9
$^{13}C\{^1H\}$NMR, $CDCl_3$, 126 MHz
O
N
Ot-Bu
H_2N
O
46
CDCl3
3800000
3600000
3400000
3200000
3000000
2800000
2600000
2400000
2200000
2000000
1800000
1600000
1400000
1200000
1000000
800000
600000
400000
200000
0
-200000
230 220 210 200 190 180 170 160 150 140 130 120 110 100 90 80 70 60 50 40 30 20 10 0 -10
f1 (ppm)

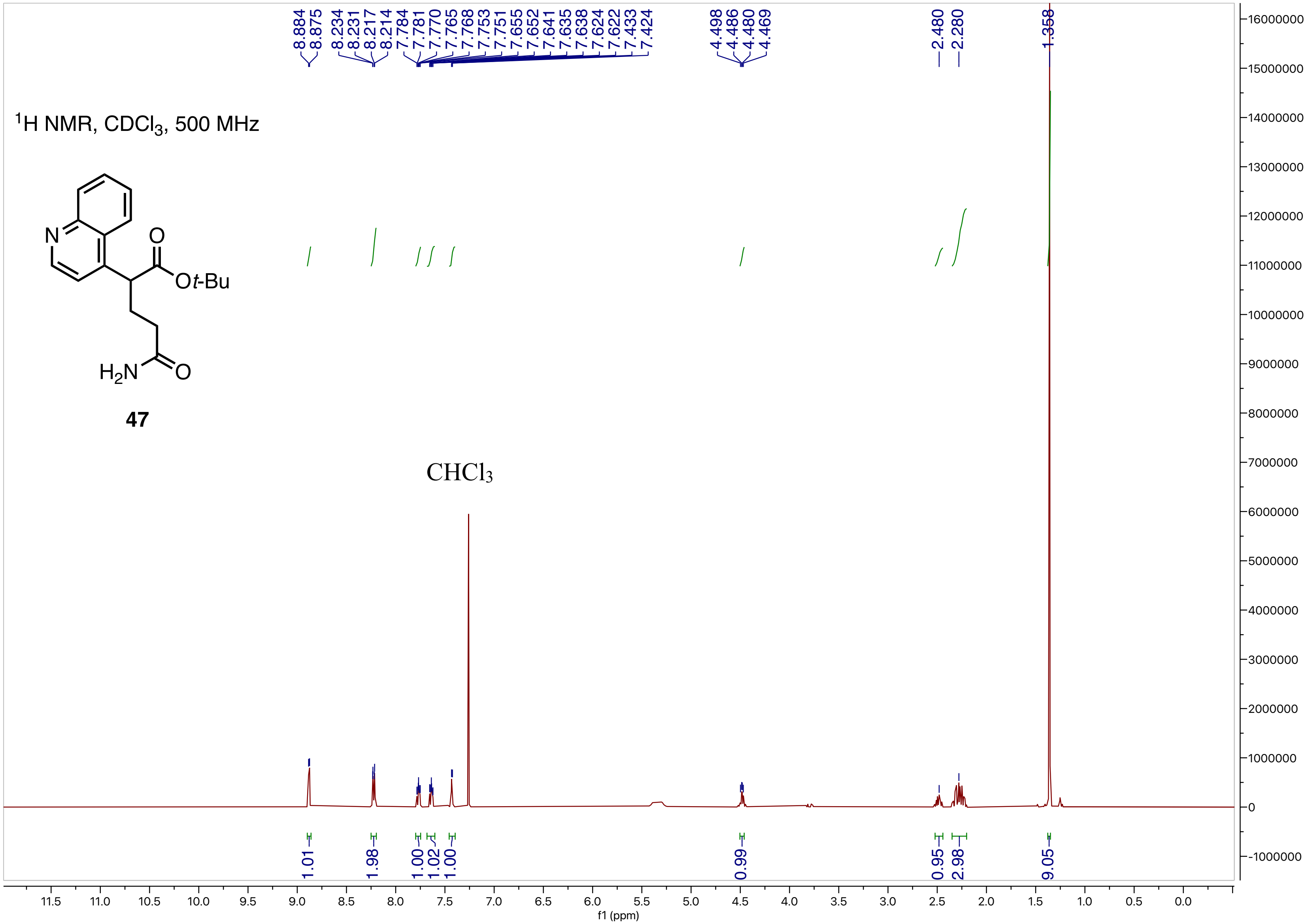
1H NMR, CDCl3, 500 MHz
47
CHCl3
8.884
8.875
8.234
8.231
8.217
8.214
7.784
7.781
7.770
7.765
7.768
7.753
7.751
7.655
7.652
7.641
7.635
7.638
7.624
7.622
7.433
7.424
4.498
4.486
4.480
4.469
2.480
2.280
1.358
1.01
1.98
1.00
1.02
1.00
0.99
0.95
2.98
9.05
f1 (ppm)

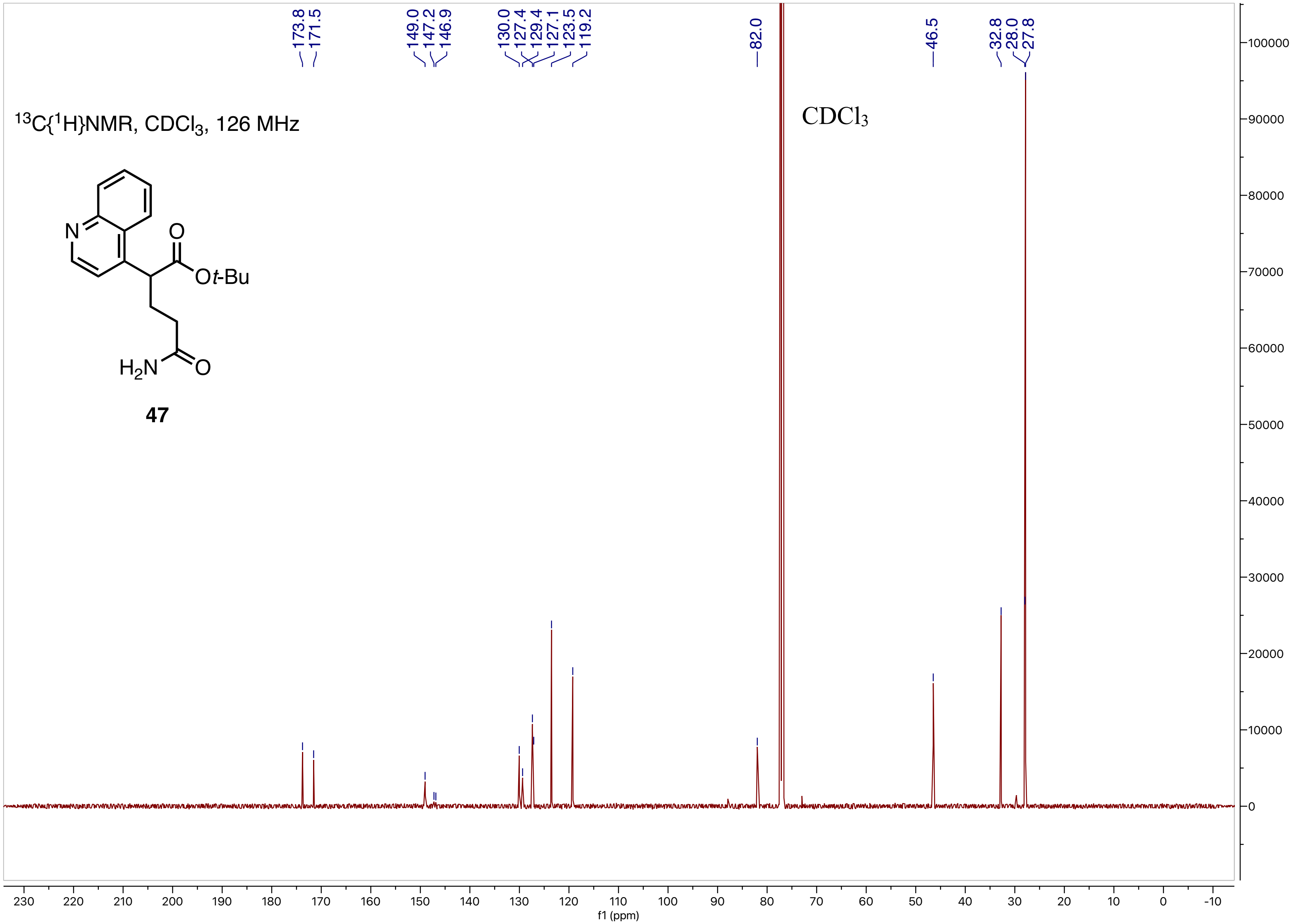
173.8
171.5
149.0
147.2
146.9
130.0
127.4
129.4
127.1
123.5
119.2
82.0
46.5
32.8
28.0
27.8
$^{13}C\{^1H\}$NMR, $CDCl_3$, 126 MHz
$CDCl_3$
N
O
O*t*-Bu
H_2N
O
47
f1 (ppm)

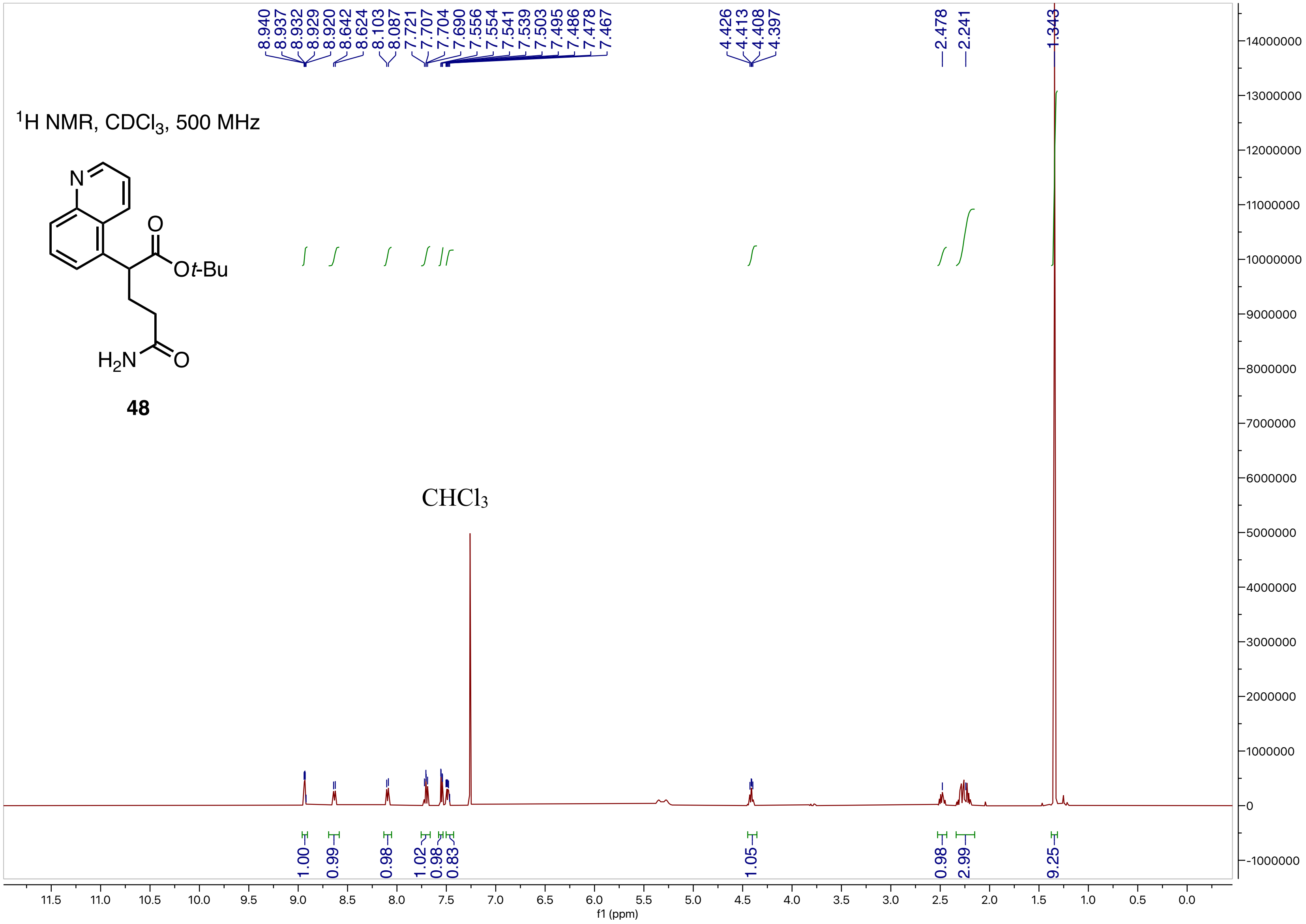

^{1}H NMR, $CDCl_3$, 500 MHz
Ot-Bu
H_2N
O
N
48
$CHCl_3$
8.940 8.937 8.932 8.929 8.920 8.642 8.624 8.103 8.087 7.721 7.707 7.704 7.690 7.556 7.554 7.541 7.539 7.503 7.495 7.486 7.478 7.467
4.426 4.413 4.408 4.397
2.478 2.241
1.343
1.00 0.99 0.98 1.02 0.98 0.83 1.05 0.98 2.99 9.25
f1 (ppm)

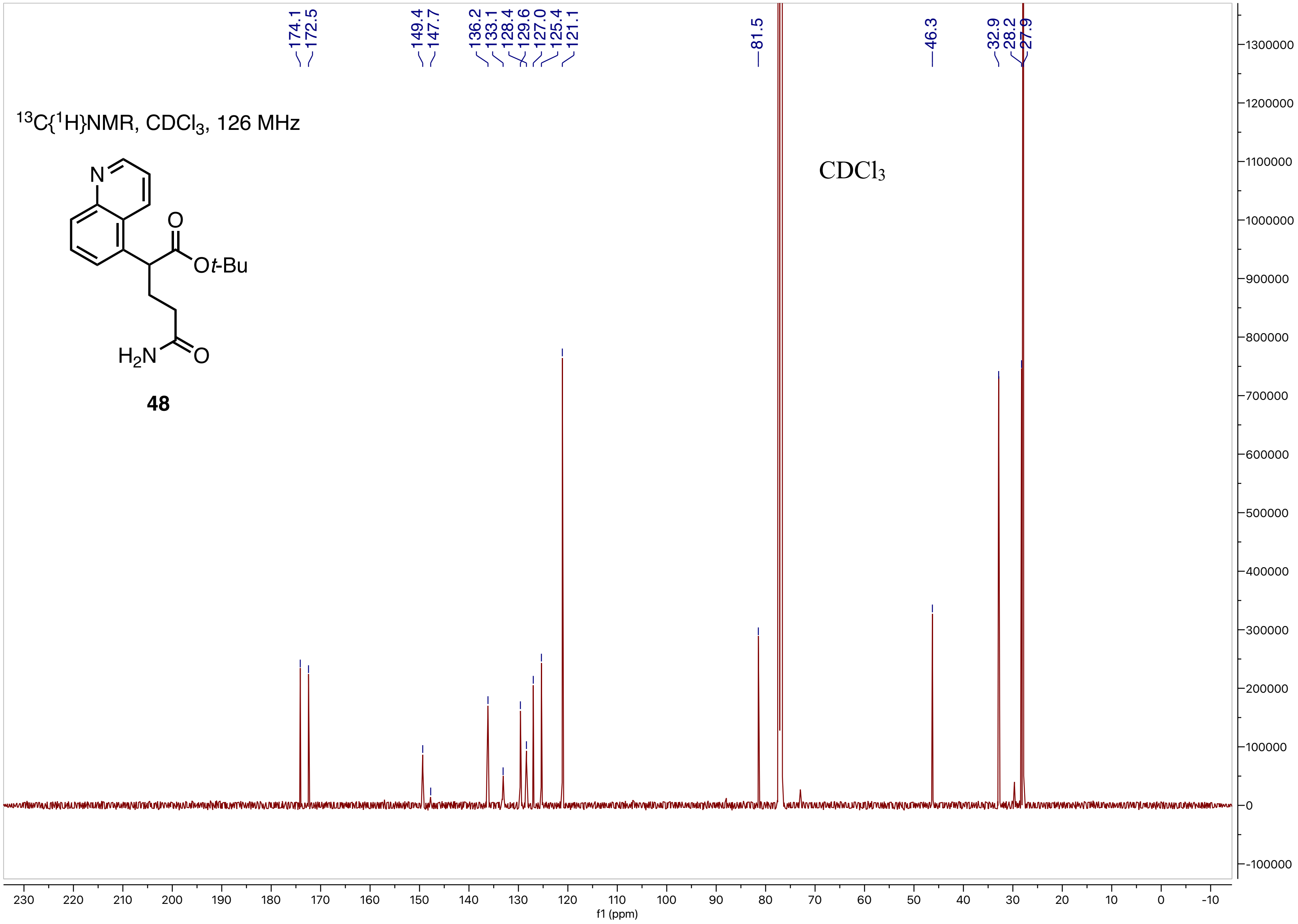

174.1
172.5
149.4
147.7
136.2
133.1
128.4
129.6
127.0
125.4
121.1
81.5
46.3
32.9
28.2
27.9
$^{13}C\{^1H\}$NMR, $CDCl_3$, 126 MHz
N
O
O*t*-Bu
H_2N
O
48
$CDCl_3$
f1 (ppm)

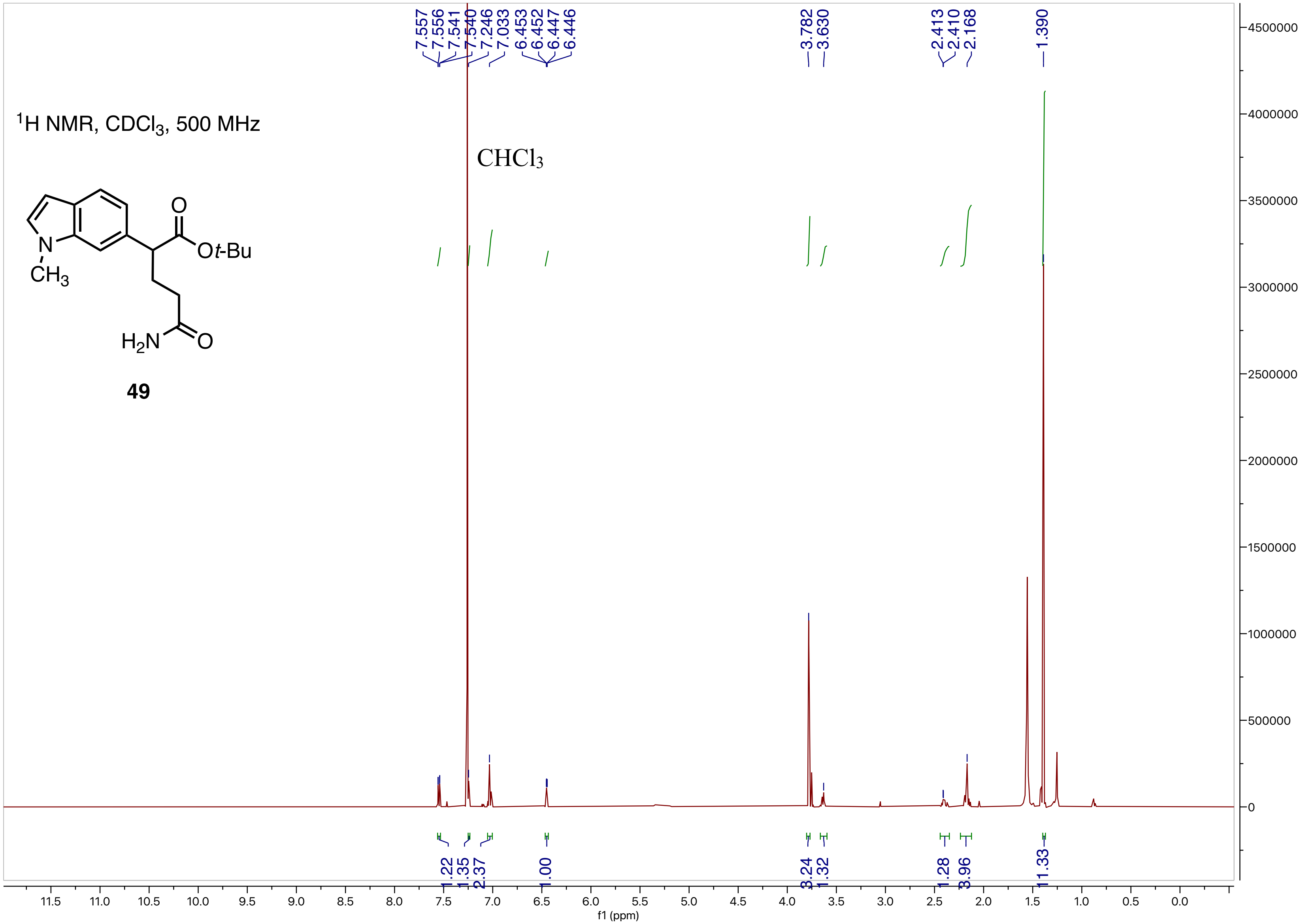

7.557
7.556
7.541
7.540
7.246
7.033
6.453
6.452
6.447
6.446
3.782
3.630
2.413
2.410
2.168
1.390
^1H NMR, $CDCl_3$, 500 MHz
$CHCl_3$
O
O*t*-Bu
N
CH_3
H_2N
O
49
1.22
1.35
2.37
1.00
3.24
1.32
1.28
3.96
11.33
f1 (ppm)

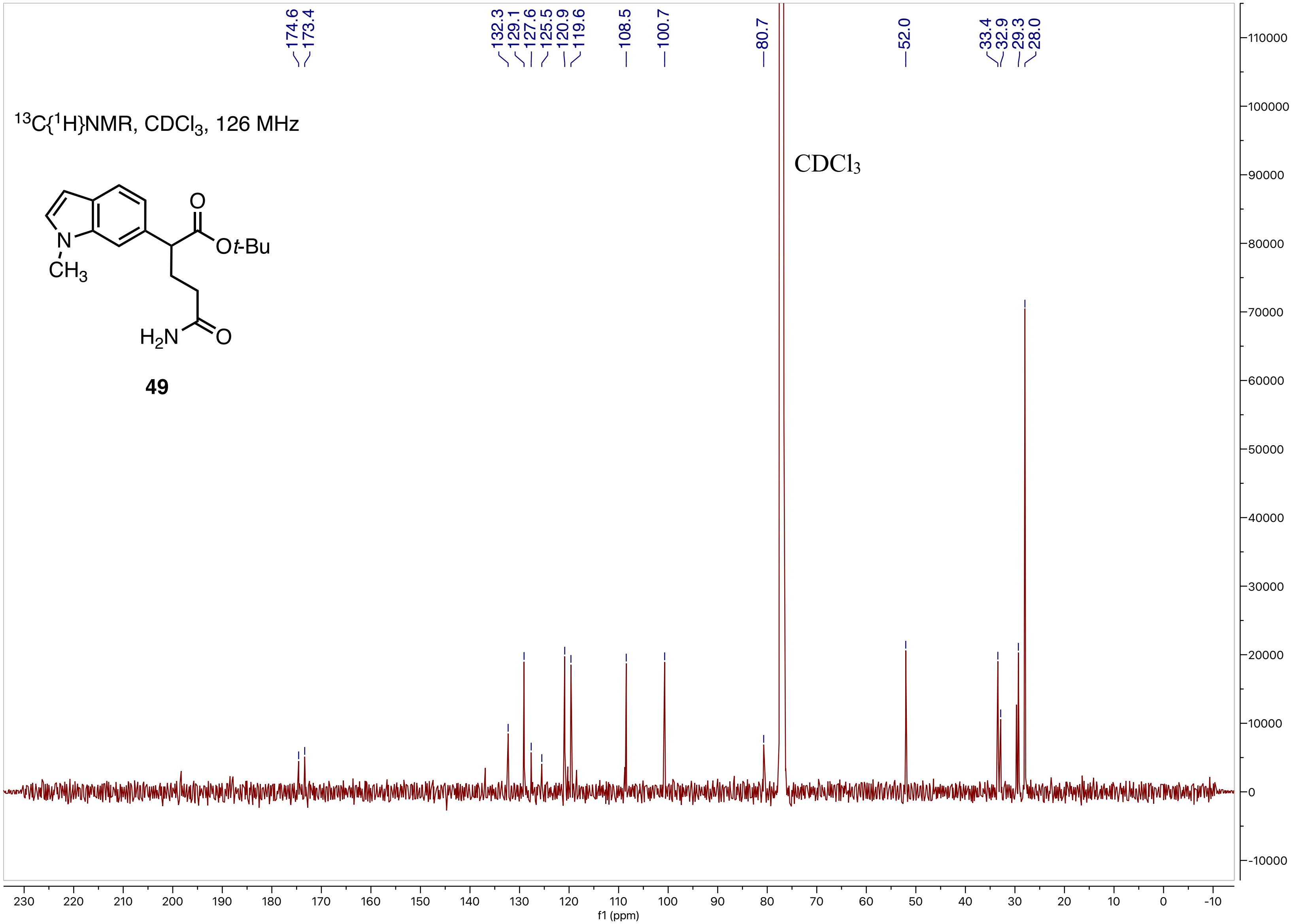

174.6
173.4
132.3
129.1
127.6
125.5
120.9
119.6
108.5
100.7
80.7
52.0
33.4
32.9
29.3
28.0
$^{13}C\{^1H\}NMR$, $CDCl_3$, 126 MHz
CDCl3
O
Ot-Bu
N
CH3
H2N
O
49
f1 (ppm)

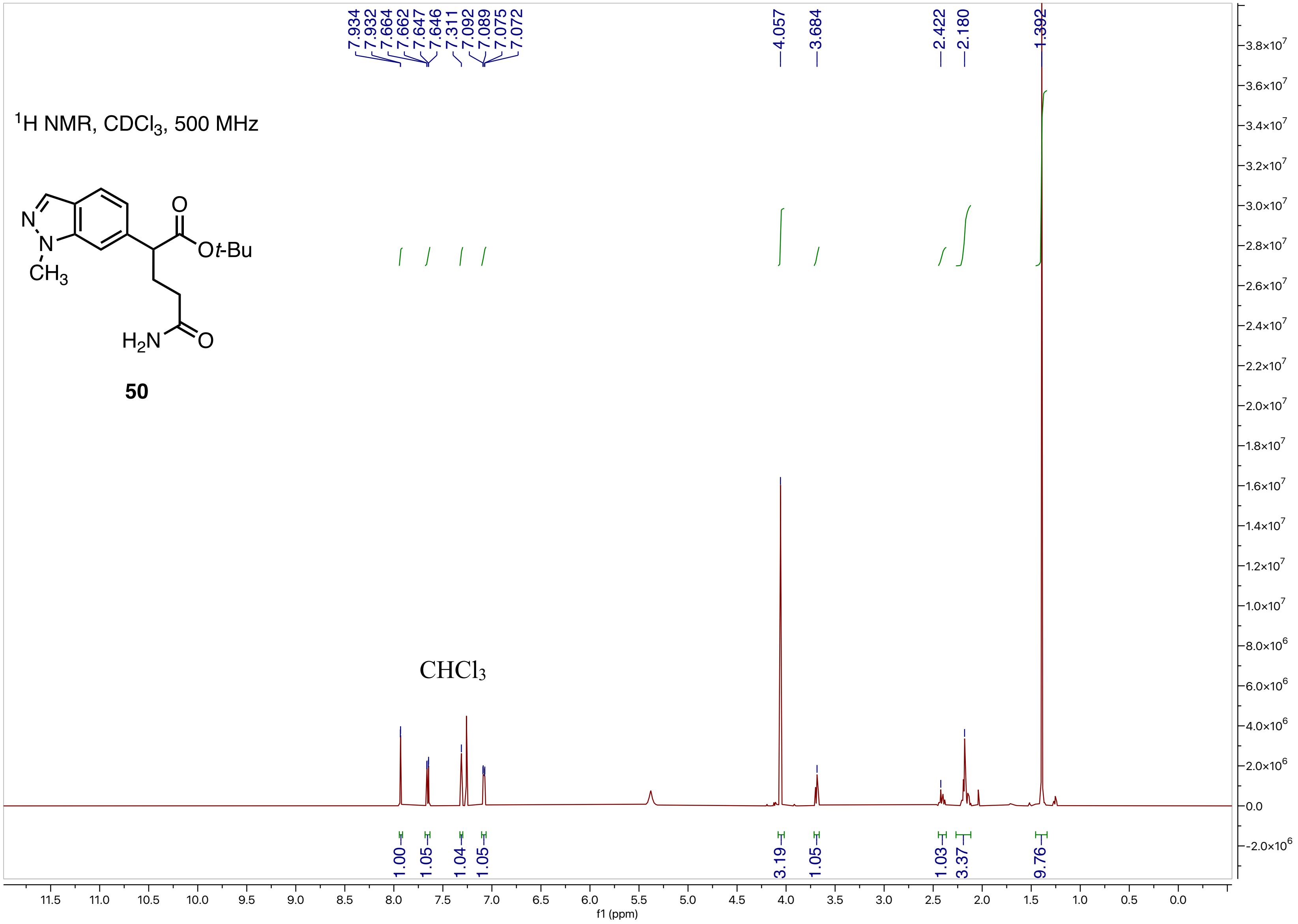

1H NMR, CDCl3, 500 MHz
Ot-Bu
H2N
CH3
50
CHCl3
7.934 7.932 7.664 7.662 7.647 7.646 7.311 7.092 7.089 7.075 7.072
4.057
3.684
2.422
2.180
1.392
1.00 1.05 1.04 1.05 3.19 1.05 1.03 3.37 9.76
f1 (ppm)

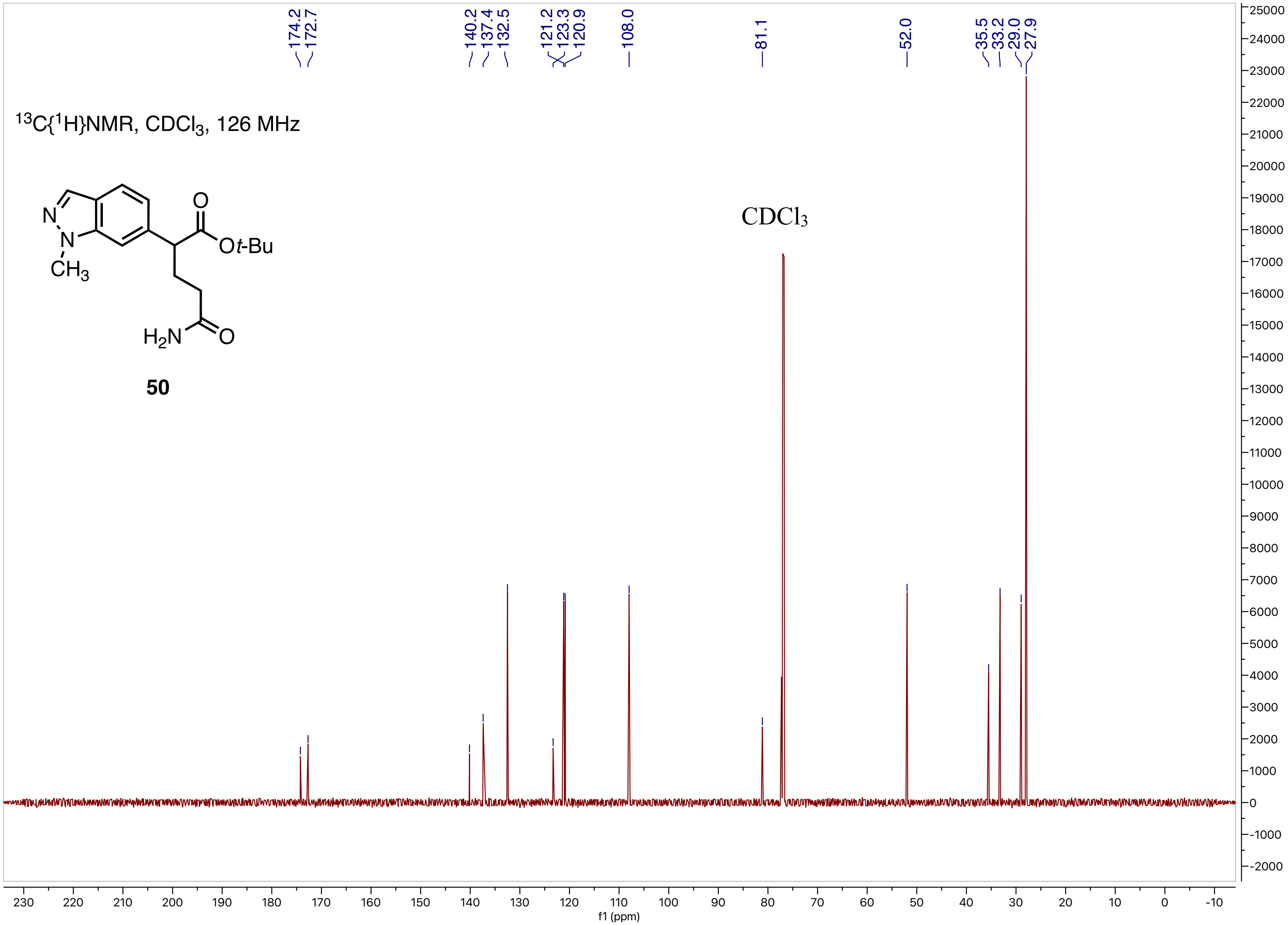
174.2
172.7
140.2
137.4
132.5
121.2
123.3
120.9
108.0
81.1
52.0
35.5
33.2
29.0
27.9
13C{1H}NMR, CDCl3, 126 MHz
N
N
CH3
O
Ot-Bu
H2N
O
50
CDCl3
f1 (ppm)

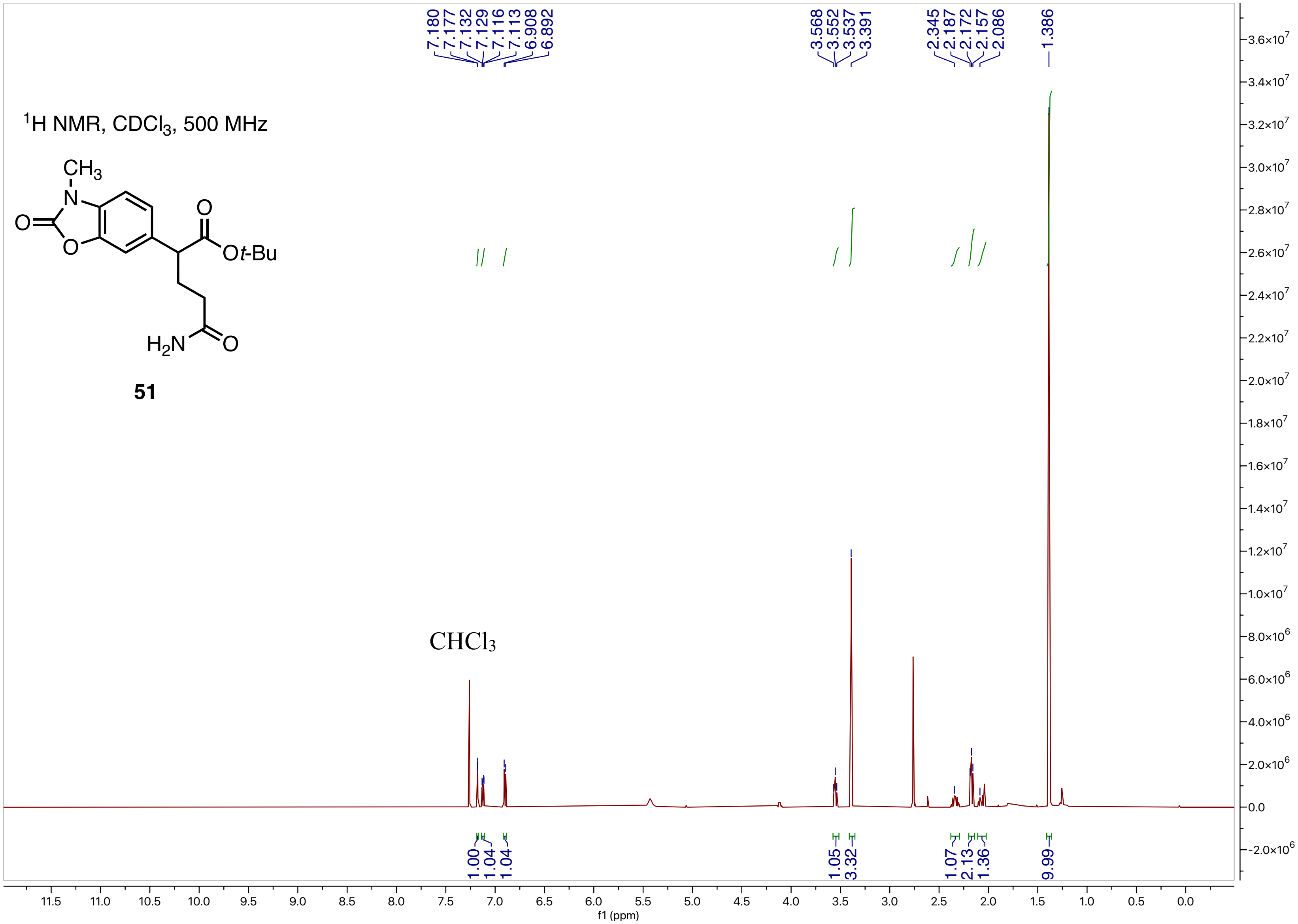
¹H NMR, CDCl3, 500 MHz
CH3
N
O
O
O
Ot-Bu
H2N
O
51
CHCl3
7.180
7.177
7.132
7.129
7.116
7.113
6.908
6.892
3.568
3.552
3.537
3.391
2.345
2.187
2.172
2.157
2.086
1.386
1.00
1.04
1.04
1.05
3.32
1.07
2.13
1.36
9.99
f1 (ppm)

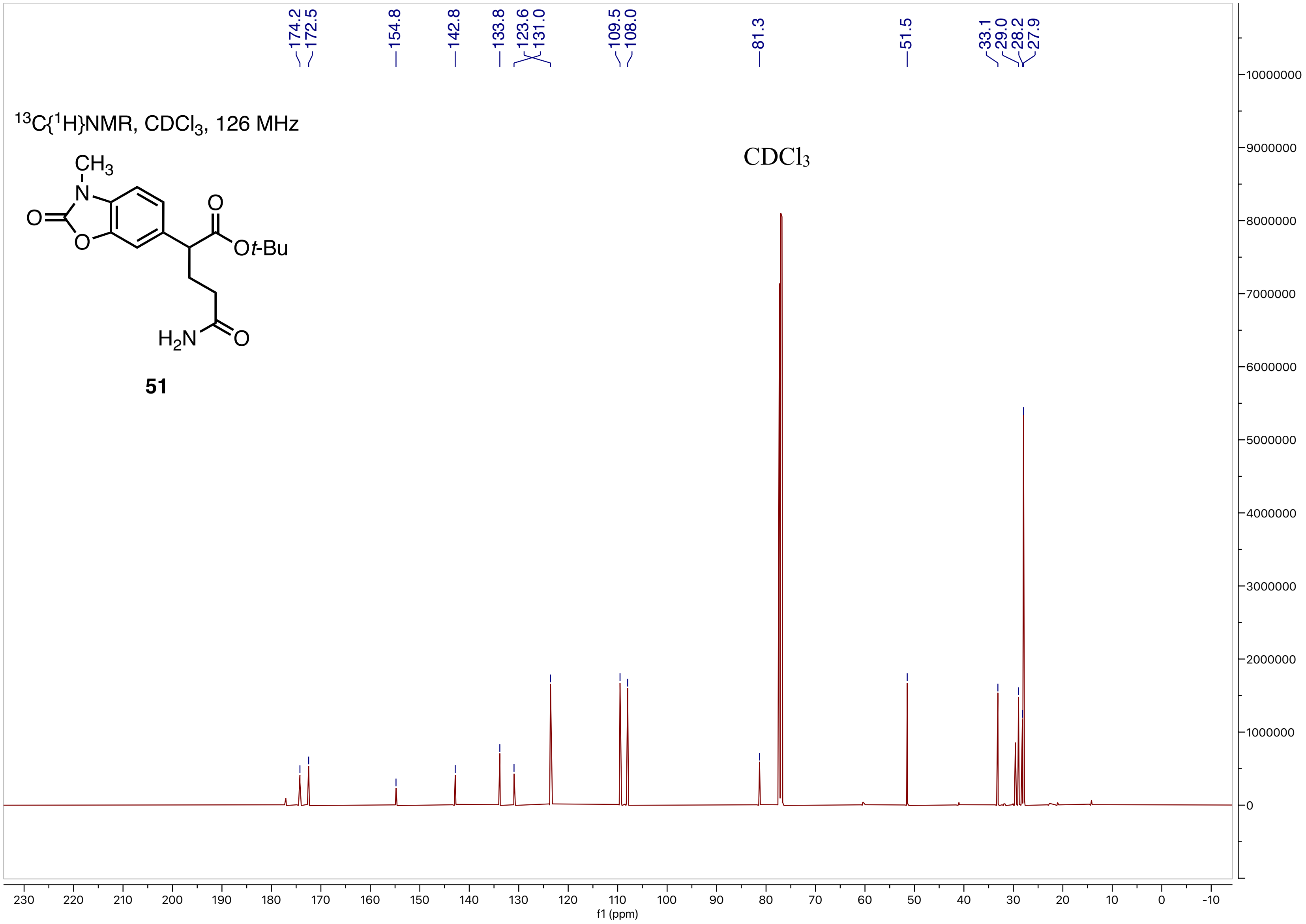

174.2
172.5
154.8
142.8
133.8
123.6
131.0
109.5
108.0
81.3
51.5
33.1
29.0
28.2
27.9
^{13}C{^{1}H}NMR, CDCl$_3$, 126 MHz
CDCl$_3$
CH$_3$
N
O
O
O
O*t*-Bu
H$_2$N
O
51
f1 (ppm)

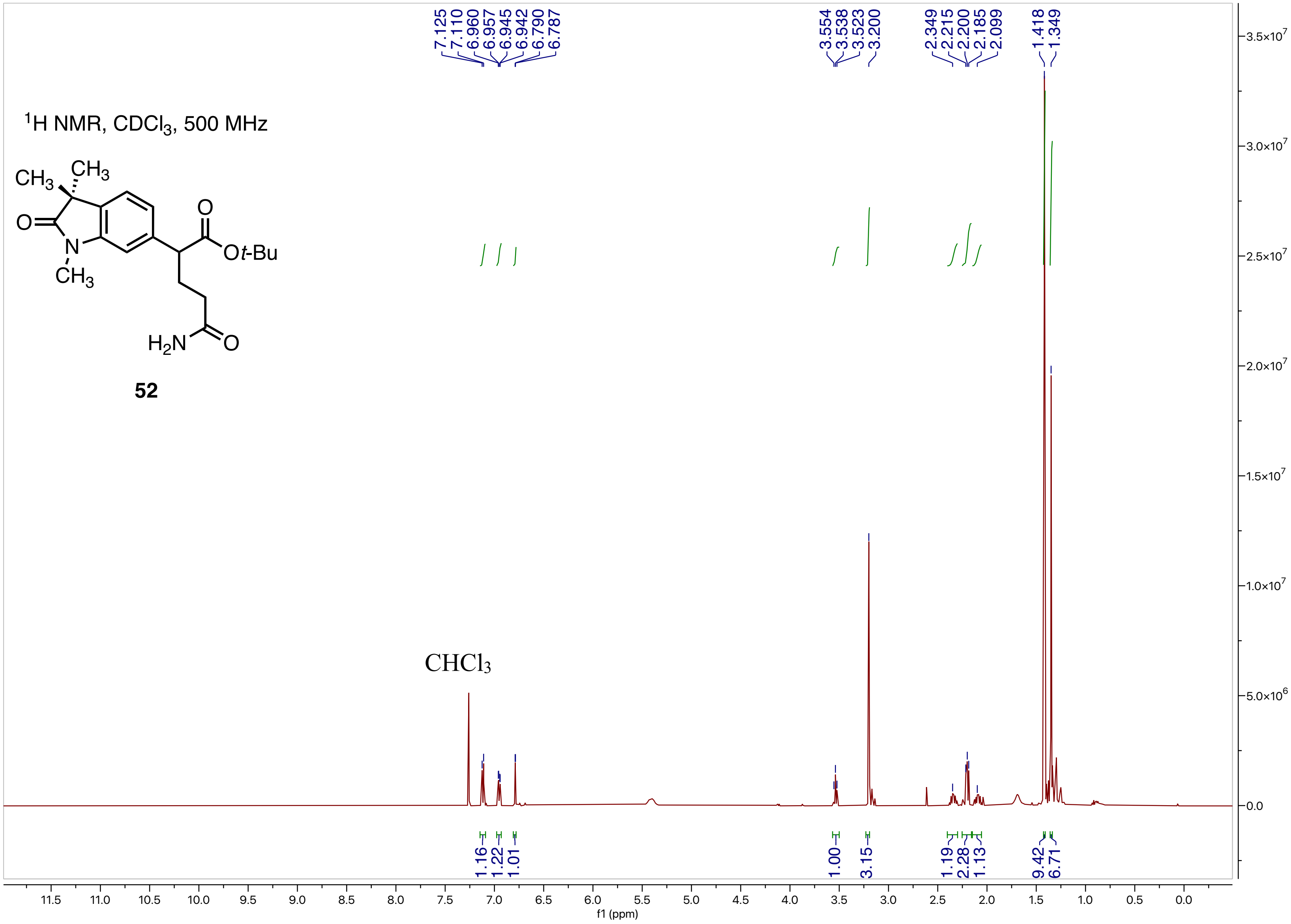

1H NMR, CDCl3, 500 MHz
52
CHCl3
7.125
7.110
6.960
6.957
6.945
6.942
6.790
6.787
3.554
3.538
3.523
3.200
2.349
2.215
2.200
2.185
2.099
1.418
1.349
1.16
1.22
1.01
1.00
3.15
1.19
2.28
1.13
9.42
6.71
f1 (ppm)

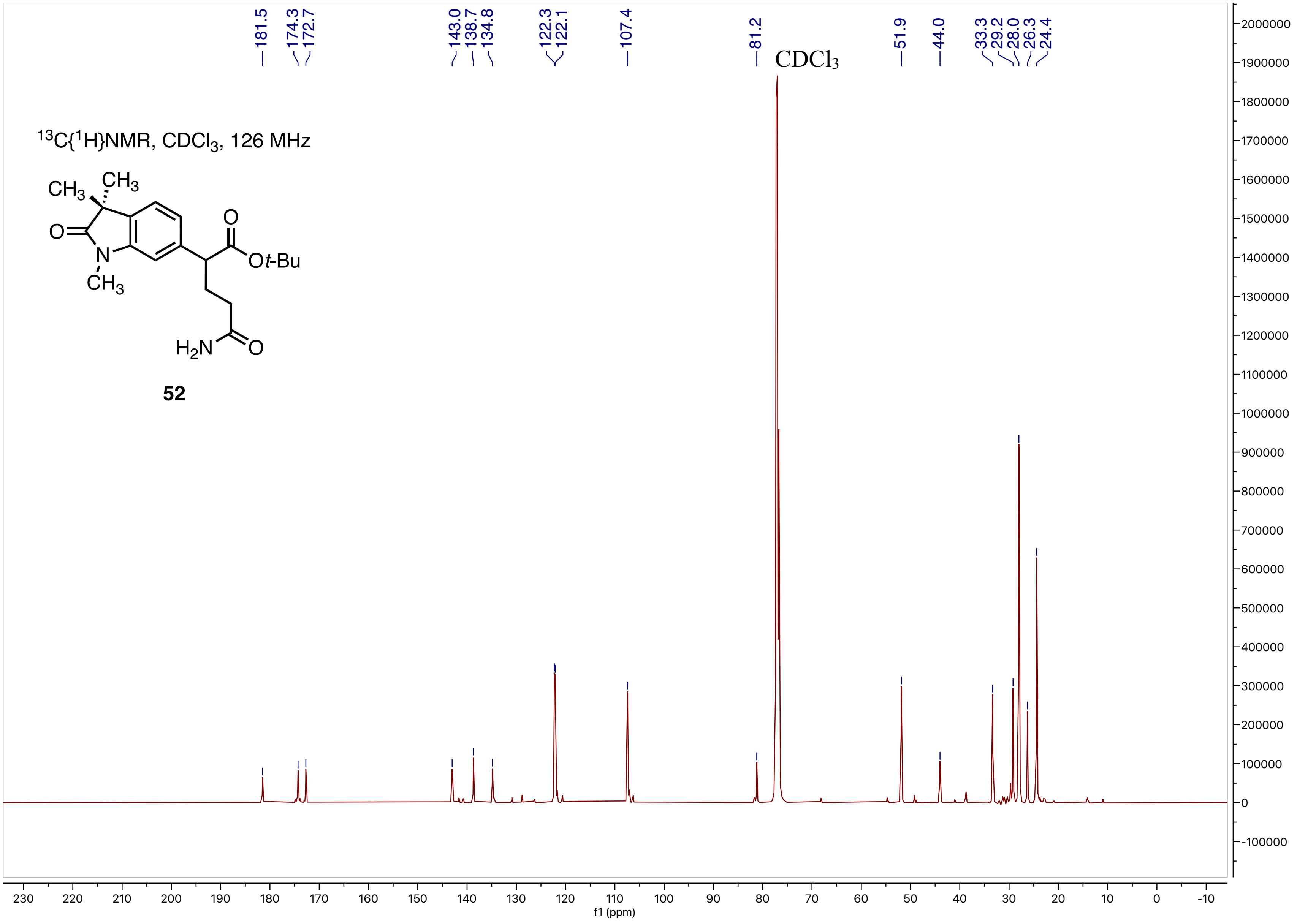
181.5
174.3
172.7
143.0
138.7
134.8
122.3
122.1
107.4
81.2
CDCl3
51.9
44.0
33.3
29.2
28.0
26.3
24.4
13C{1H}NMR, CDCl3, 126 MHz
52
f1 (ppm)

13. Bibliography.

[1] W. C. Still, M. Kahn, A. Mitra, "Rapid chromatographic technique for preparative separations with moderate resolution" *J. Org. Chem.* **1978**, *43*, 2923.

[2] D. G. Gibson, L. Young, R.-Y. Chuang, J. C. Venter, C. A. Hutchison, H. O. Smith, "Enzymatic assembly of DNA molecules up to several hundred kilobases" *Nat. Methods* **2009**, *6*, 343.

[3] K. F. Biegasiewicz, S. J. Cooper, X. Gao, D. G. Oblinsky, J. H. Kim, S. E. Garfinkle, L. A. Joyce, B. A. Sandoval, G. D. Scholes, T. K. Hyster, "Photoexcitation of flavoenzymes enables a stereoselective radical cyclization" *Science* **2019**, *364*, 1166.

[4] M. J. Black, K. F. Biegasiewicz, A. J. Meichan, D. G. Oblinsky, B. Kudisch, G. D. Scholes, T. K. Hyster, "Asymmetric redox-neutral radical cyclization catalysed by flavin-dependent 'ene'-reductases" *Nat. Chem.* **2020**, *12*, 71.

[5] S. Kille, C. G. Acevedo-Rocha, L. P. Parra, Z.-G. Zhang, D. J. Opperman, M. T. Reetz, J. P. Acevedo, "Reducing Codon Redundancy and Screening Effort of Combinatorial Protein Libraries Created by Saturation Mutagenesis" *ACS Synth. Biol.* **2013**, *2*, 83.

[6] T. Hama, D. A. Culkin, J. F. Hartwig, "Palladium-Catalyzed Intermolecular α-Arylation of Zinc Amide Enolates under Mild Conditions" *J. Am. Chem. Soc.* **2006**, *128*, 4976.

[7] C. Wang, L. Zong, C.-H. Tan, "Enantioselective Oxidation of Alkenes with Potassium Permanganate Catalyzed by Chiral Dicationic Bisguanidinium" *J. Am. Chem. Soc.* **2015**, *137*, 10677.

[8] Z. Chen, W. Zhong, S. Liu, T. Zou, K. Zhang, C. Gong, W. Guo, F. Kong, L. Nie, S. Hu, H. Wang, "Highly Stereodivergent Synthesis of Chiral C4-Ester-Quaternary Pyrrolidines: A Strategy for the Total Synthesis of Spirotryprostatin A" *Org. Lett.* **2023**, *25*, 3391.

[9] X. Yang, S. Lu, Y. Zhang, H. Xu, X. Cai, C. Shu, "DABSO-Mediated Hydrosulfonylation of Activated Alkenes" *Org. Lett.* **2025**, *27*, 4927.

[10] J. Kim, S. Chang, "Iridium-Catalyzed Direct C–H Amidation with Weakly Coordinating Carbonyl Directing Groups under Mild Conditions" *Angew. Chem. Int. Ed.* **2014**, *53*, 2203.

[11] C. S. Buxton, D. C. Blakemore, J. F. Bower, "Reductive Coupling of Acrylates with Ketones and Ketimines by a Nickel-Catalyzed Transfer-Hydrogenative Strategy" *Angew. Chem. Int. Ed.* **2017**, *56*, 13824.

[12] D.-D. Chen, B.-Y. Zhang, X.-X. Liu, X.-Q. Li, X.-J. Yang, L. Zhou, "Bioactivity and structure–activity relationship of cinnamic acid derivatives and its heteroaromatic ring analogues as potential high-efficient acaricides against Psoroptes cuniculi" *Bioorg. Med. Chem. Lett.* **2018**, *28*, 1149.

[13] T. Hama, J. F. Hartwig, "Palladium-Catalyzed α-Arylation of Esters with Chloroarenes" *Org. Lett.* **2008**, *10*, 1549.

[14] L.-M. Chen, C. Shin, T. J. DeLano, A. Carretero-Cerdán, G. Gheibi, S. E. Reisman, "Ni-Catalyzed Asymmetric Reductive Arylation of α-Substituted Imides" *J. Am. Chem. Soc.* **2024**, *146*, 29523.

[15] P. Macheroux, S. Ghisla, C. Sanner, H. Rüterjans, F. Müller, "Reduced Flavin: NMR investigation of N(5)-H exchange mechanism, estimation of ionisation constants and assessment of properties as biological catalyst" *BMC Biochemistry* **2005**, *6*, 26.

[16] C. G. Page, S. J. Cooper, J. S. DeHovitz, D. G. Oblinsky, K. F. Biegasiewicz, A. H. Antropow, K. W. Armbrust, J. M. Ellis, L. G. Hamann, E. J. Horn, K. M. Oberg, G. D.

Scholes, T. K. Hyster, “Quaternary Charge-Transfer Complex Enables Photoenzymatic Intermolecular Hydroalkylation of Olefins” *J. Am. Chem. Soc.* **2021**, *143*, 97.

[17] C. Hansch, A. Leo, R. W. Taft, “A survey of Hammett substituent constants and resonance and field parameters” *Chem. Rev.* **1991**, *91*, 165.

[18] H. Fu, T. K. Hyster, “From Ground-State to Excited-State Activation Modes: Flavin-Dependent “Ene”-Reductases Catalyzed Non-natural Radical Reactions” *Acc. Chem. Res.* **2024**, *57*, 1446.

[19] C. Wagen, A. Wagen, *ChemRxiv* preprint **2024**, DOI: 10.26434/chemrxiv-2024-8489b.

[20] S. Riniker, G. A. Landrum, “Better Informed Distance Geometry: Using What We Know To Improve Conformation Generation” *J. Chem. Inf. Model.* **2015**, *55*, 2562.

[21] C. Bannwarth, S. Ehlert, S. Grimme, “GFN2-xTB—An Accurate and Broadly Parametrized Self-Consistent Tight-Binding Quantum Chemical Method with Multipole Electrostatics and Density-Dependent Dispersion Contributions” *J. Chem. Theory Comput.* **2019**, *15*, 1652.

[22] D. Anstine, R. Zubatyuk, O. Isayev O, *ChemRxiv* preprint **2024**, DOI:10.26434/chemrxiv-2023-296ch-v3.

[23] M. Stahn, S. Ehlert, S. Grimme, “Extended Conductor-like Polarizable Continuum Solvation Model (CPCM-X) for Semiempirical Methods” *J. Phys. Chem. A* **2023**, *127*, 7036.

[24] S. Grimme, A. Hansen, S. Ehlert, J.-M. Mewes, “r2SCAN-3c: A “Swiss army knife” composite electronic-structure method” *J. Chem. Phys.* **2021**, *154*.

[25] M. J. Abraham, T. Murtola, R. Schulz, S. Páll, J. C. Smith, B. Hess, E. Lindahl, “GROMACS: High performance molecular simulations through multi-level parallelism from laptops to supercomputers” *SoftwareX* **2015**, *1-2*, 19.

[26] Molecular Operating Environment (MOE), 2024.0601 Chemical Computing Group ULC, 910-1010 Sherbrooke St. W., Montreal, QC H3A 2R7, 2025.

[27] S. Somani, S. Jo, R. Thirumangalathu, D. Rodrigues, L. M. Tanenbaum, K. Amin, A. D. MacKerell, S. V. Thakkar, “Toward Biotherapeutics Formulation Composition Engineering using Site-Identification by Ligand Competitive Saturation (SILCS)” *J. Pharm. Sci.* **2021**, *110*, 1103.

[28] V. D. Ustach, S. K. Lakkaraju, S. Jo, W. Yu, W. Jiang, A. D. MacKerell, Jr., “Optimization and Evaluation of Site-Identification by Ligand Competitive Saturation (SILCS) as a Tool for Target-Based Ligand Optimization” *J. Chem. Inf. Model.* **2019**, *59*, 3018.

[29] E. P. Raman, W. Yu, S. K. Lakkaraju, A. D. MacKerell, Jr., “Inclusion of Multiple Fragment Types in the Site Identification by Ligand Competitive Saturation (SILCS) Approach” *J. Chem. Inf. Model.* **2013**, *53*, 3384.

[30] E. P. Raman, W. Yu, O. Guvench, A. D. MacKerell, Jr., “Reproducing Crystal Binding Modes of Ligand Functional Groups Using Site-Identification by Ligand Competitive Saturation (SILCS) Simulations” *J. Chem. Inf. Model.* **2011**, *51*, 877.

[31] R. B. Best, X. Zhu, J. Shim, P. E. M. Lopes, J. Mittal, M. Feig, A. D. MacKerell, Jr., “Optimization of the Additive CHARMM All-Atom Protein Force Field Targeting Improved Sampling of the Backbone ϕ, ψ and Side-Chain $\chi 1$ and $\chi 2$ Dihedral Angles” *J. Chem. Theory Comput.* **2012**, *8*, 3257.

[32] J. Huang, S. Rauscher, G. Nawrocki, T. Ran, M. Feig, B. L. de Groot, H. Grubmüller, A. D. MacKerell, "CHARMM36m: an improved force field for folded and intrinsically disordered proteins" *Nat. Methods* **2017**, *14*, 71.
[33] K. Vanommeslaeghe, E. Hatcher, C. Acharya, S. Kundu, S. Zhong, J. Shim, E. Darian, O. Guvench, P. Lopes, I. Vorobyov, A. D. Mackerell Jr., "CHARMM general force field: A force field for drug-like molecules compatible with the CHARMM all-atom additive biological force fields" *J. Comput. Chem.* **2010**, *31*, 671.
[34] S. R. Durell, B. R. Brooks, A. Ben-Naim, "Solvent-Induced Forces between Two Hydrophilic Groups" *J. Phys. Chem.* **1994**, *98*, 2198.
